# Age-related changes after intracerebral hemorrhage: a comparative proteomics analysis of perihematomal tissue

**DOI:** 10.3389/ebm.2024.10117

**Published:** 2024-03-25

**Authors:** Xinhui Li, Zhongsong Xiao, Peizheng Li, Wensong Yang, Yiqing Shen, Fangyu Liu, Xin Xiong, Qingyuan Wu, Peng Wang, Ruozhi Dang, Siwen Gui, Lan Deng, Anatol Manaenko, Peng Xie, Qi Li

**Affiliations:** ^1^ Department of Neurology, The First Affiliated Hospital of Chongqing Medical University, Chongqing, China; ^2^ NHC Key Laboratory of Diagnosis and Treatment on Brain Functional Diseases, The First Affiliated Hospital of Chongqing Medical University, Chongqing, China; ^3^ Department of Neurology, Chongqing Hospital of Traditional Chinese Medicine, Chongqing, China; ^4^ Department of Neurology, Chongqing University Three Gorges Hospital, Chongqing, China; ^5^ Department of Neurology, The Second Affiliated Hospital of Anhui Medical University, Hefei, Anhui, China

**Keywords:** proteomics, intracerebral hemorrhage, aging, protein-protein interaction, tandem mass tag

## Abstract

The risk factors and causes of intracerebral hemorrhage (ICH) and the degree of functional recovery after ICH are distinct between young and elderly patients. The increasing incidence of ICH in young adults has become a concern; however, research on the molecules and pathways involved ICH in subjects of different ages is lacking. In this study, tandem mass tag (TMT)-based proteomics was utilized to examine the protein expression profiles of perihematomal tissue from young and aged mice 24 h after collagenase-induced ICH. Among the 5,129 quantified proteins, ICH induced 108 and 143 differentially expressed proteins (DEPs) in young and aged mice, respectively; specifically, there were 54 common DEPs, 54 unique DEPs in young mice and 89 unique DEPs in aged mice. In contrast, aging altered the expression of 58 proteins in the brain, resulting in 39 upregulated DEPs and 19 downregulated DEPs. Bioinformatics analysis indicated that ICH activated different proteins in complement pathways, coagulation cascades, the acute phase response, and the iron homeostasis signaling pathway in mice of both age groups. Protein–protein interaction (PPI) analysis and ingenuity pathway analysis (IPA) demonstrated that the unique DEPs in the young and aged mice were related to lipid metabolism and carbohydrate metabolism, respectively. Deeper paired-comparison analysis demonstrated that apolipoprotein M exhibited the most significant change in expression as a result of both aging and ICH. These results help illustrate age-related protein expression changes in the acute phase of ICH.

## Impact statement

The increasing incidence of intracerebral hemorrhage (ICH) in young adults has become a concern; however, research on susceptible molecules and pathways specifically targeting ICH in subjects of different ages remains lacking. We aimed to investigate the underlying interaction relationship between aging and ICH by proteomics approaches. In this study, we performed tandem mass tag (TMT)-based proteomics on young and aged mice after constructing collagenase-induced ICH models to screen protein expression in brain perihematomal tissue among groups.

## Introduction

Intracerebral hemorrhage (ICH) is a devastating stroke subtype that is associated with low treatable rates and high mortality rates [1]. Approximately 50% of patients die within the first 48 h, and most patients suffer from a severe disability after being discharged from the hospital [2]. Previous studies have revealed that young adult and elderly patients with acute ICH exhibit distinct incidence rates, causes and functional recoveries [3]. Recently, the increasing incidence of ICH in young adults has become a major concern, and permanent disability and death caused by ICH in this demographic bring heavy economic burdens to society and public health organizations [4, 5]. To optimize preventive and protective approaches for young adults with ICH, the pathological changes that occur in young subjects after ICH need to be investigated.

Recently, genome-wide studies of ICH populations have been carried out [6–8]. High-throughput sequencing has provided evidence that age-specific changes occur after ICH events. A proteomics analysis detected differentially expressed proteins (DEPs) in plasma among young, middle-aged, and older healthy individuals and confirmed that insulin-like growth factor 1 is a promising novel approach for ameliorating aging-associated acute brain injury after ICH [9]. The other transcriptomic analysis distinguished the expression pattern of oxylipin enzymes between middle-aged and aged rats 3 days after experimental ICH induction and revealed that among the specific differentially expressed genes (DEGs), Cyp1a1 and Cyp2e1 were the most significant DEGs in the two age groups [10]. In addition, in pathological findings of animals with ICH, more severe brain swelling was observed in aged animals than in young animals, and aged animals exhibited different glial responses from young animals, impaired lesion resolution, and neuronal death [11]. Interestingly, a recent study based on another classic stroke model, middle cerebral artery occlusion mice, found that O-GlcNAcylation was a pro-survival pathway that was impaired in the stroke penumbra in aged mice but was activated in young mice, and this study introduced a therapeutic strategy for treating ischemic stroke in an age-specific manner [12]. However, relatively little is known about the different responses to ICH in aged and young subjects. Therefore, understanding the discrepancies in the mechanisms of internal molecular injury in young and aged patients after ICH is highly desirable.

In ICH, primary injury occurs due to the mechanical compression of the brain parenchyma caused by an expanding intracranial hematoma, resulting in brain edema and neuron damage in the perihematomal area [13]. Furthermore, local ischemia and hypoxia in brain tissue, along with the release of blood components from ruptured blood vessels, initiate secondary brain injury, activating microglia and resident macrophages within the brain and recruiting peripheral leukocytes through the compromised blood‒brain barrier to the perihematomal area, further exacerbating inflammatory injury [14]. Therefore, the perihematomal region is the most commonly studied region in the field of ICH [15–18]. Experimental analysis of perihematomal tissue can provide an in-depth understanding of the mechanisms of secondary injury, the related role of inflammatory mediators, and the processes of neuroprotection and neuronal repair, yielding valuable evidence for the development of strategies for treating brain injury, inhibiting inflammatory responses, and promoting neuroprotection.

This study aimed to explore the differences in protein expression in perihematomal tissue between young and aged mice using proteomics data. The functions and pathways of common and unique DEPs that were identified by comparative proteomics between young and aged mice were investigated, and possible targets and pathways for follow-up studies across different life periods were identified.

## Materials and methods

### Animals

Specific-pathogen-free 10-week-old and 22-month-old male C57BL/6 mice were purchased from the Laboratory Animal Center of Chongqing Medical University (CQMU). Animals were maintained in a pathogen-free facility and were fed *ad libitum* in a 12/12-h light-dark environment at 25°C. All experimental protocols were approved by the Ethical Committee of CQMU. All efforts were made to reduce the number of animals and the amount of suffering they endured.

### Animal models

The collagenase IV-induced ICH mouse model was established based on a previous study [19]. Anaesthesia was administered to the mice through the inhalation of 4% isoflurane. After remaining under anaesthesia with 1.5% isoflurane, the mice were placed on a stereotaxic apparatus (Rivard, China) in a prone position with a rectal temperature that was maintained at 37.5°C. A 26-gauge microsyringe (Hamilton, the United States) was inserted into the right striatum through an eyehole that was drilled in the skull (the anterior fontanelle was the origin of the coordinate with 0.6 mm anterior and 2.3 mm lateral directions and a 3.7 mm depth). ICH models or sham controls were induced by 0.075 U collagenase (Sigma, the United States) in 0.4 µL of 0.9% physiological saline or an equivalent dose of saline at a rate of 0.5 μL/min. The mice were placed on a warming pad until consciousness was recovered.

### Sampling

Mice were randomly divided into a young sham group, a young ICH group, an aged sham group, and an aged ICH group. Twenty-four hours after ICH, the mice were anaesthetized with pentobarbital sodium. Approximately 2 minutes later, after the mice were successfully anaesthetized, they were perfused transcardially with 30 mL sterile phosphate-buffered saline within 6 min. Immediately after perfusion, the brains of the mice were removed, and 1 mm coronal brain sections of perihematomal tissue from the basal ganglia were prepared according to our previous study [20]. The tissues were shock frozen in liquid nitrogen for 30 min and stored at −80°C until use.

### Protein extraction and peptide fractionation

Eight mice in each group were used for the proteomic analysis, and 2 or 3 brain samples were pooled as one biological replicate (n = 3 in each group). The samples were lysed in 4% SDS, 10 mM Tris-HCl, and 1 mM DTT (pH = 7.6). Then, the lysates were transferred to a 2 mL centrifuge tube prefilled with an appropriate amount of quartz sand and a 1/4-inch ceramic bead (MP 6540-424), homogenized twice for 120 s (24 × 2, 6.0 m/s) by a FastPrep-24 instrument (MP Biomedicals, the United States) and ultrasonicated 10 times for 100 s (100 W, sonication for 10 s, rest for 5 s). Next, the samples were boiled in water for 15 min and centrifuged at 14,000 *g* for 30 min, and the supernatant was filtered via 0.22 μm filters. The concentration of the final extracted proteins was quantified using a BCA Protein Assay Kit (Bio-Rad, the United States). Protein digestion and peptide fractionation were performed based on a filter-aided sample preparation (FASP) protocol [21]. In brief, 200 µg of protein sample was incubated with the detergent dithiothreitol to remove residual low-molecular-weight molecules, and then iodoacetamide was added for the alkylation reaction. Peptide concentrations were measured using the ultraviolet spectral density method at 280 nm.

### Tandem mass tag (TMT)-based liquid chromatography–mass spectrometry (LC–MS) proteomics analysis

The digested peptides were labelled by the Tandem Mass Tags (TMT) reagent kit (Thermo Scientific, the United States). The label information of 12 samples is shown in Table 1. After TMT labeling, the samples were specifically identified and analyzed using database search software. LC/MS-MS analysis was carried out using Easy nLC Proxeon Biosystems coupled with a Q Exactive mass spectrometer (Thermo Scientific, the United States) for 60/90 min. The digested peptides were loaded onto a nano Viper C18 trap column (100 μm i.d. ×2 cm, Acclaim PepMap100) (Thermo Scientific, the United States) and separated on a capillary C18-reversed-phase analytical column (75 μm i.d. ×10 cm, 3 μm resin, Easy Column) (Thermo Scientific, the United States) with an eluent buffer composed of 0.1% formic acid (FA) and a linear gradient buffer composed of 0.1% FA and 84% acetonitrile at a flow rate of 0.300 μL/min. The 10 most abundant precursor ions were processed during each scan cycle by higher energy collision-induced dissociation (HCD) fragmentation. The automatic gain control (AGC) target value was 3 x e6, and the mass range was 300–1800 m/z. The duration time for the dynamic exclusion was 2/3 min, and the injection time did not exceed 10 ms. The resolutions for the survey scans and HCD spectra were set to 70,000 at m/z 200 and 30,000 at m/z 200, respectively. The underfill rate was 0.1%, the normalized collision energy was adjusted to 30 eV, and the width parameter of isolation was 2 m/z. The mass spectrometer was run in positive mode, and the apparatus was operated in peptide recognition mode.

**TABLE 1:** Label information of 12 samples.

TMT label	126	127N	127C	128N	128C	129N	129C	130N	130C	131N	131C	132N	No.
sample	aged-ICH-1	aged-ICH-2	aged-ICH-3	aged-sham-1	aged-sham-2	aged-sham-3	young-ICH-1	young-ICH-2	young-ICH-3	young-sham-1	young-sham-2	young-sham-3	1

### Protein identification

The raw proteomics data were analyzed by Proteome Discoverer software (v.1.4) using the MASCOT engine (v.2.2) (Matrix Science, Britain). Tandem mass spectra data were matched against the UniProt database^1^. Trypsin was defined as the cleavage enzyme, and the maximum missing cleavage was 2. The peptide ion mass tolerance was ±20 ppm, and the fragment ion mass tolerance was 0.1 Da. The false discovery rate (FDR) for protein and peptide identification was ≤0.01. Protein quantitative analysis was performed according to the median of the exclusive specific peptides in the MS/MS spectra. The carbamidomethylation of cysteine and the oxidation of methionine were defined as fixed modification and variable modification, respectively. TMT-16plex was used in the quantitation process.

### Bioinformatics

**FIGURE 1:**
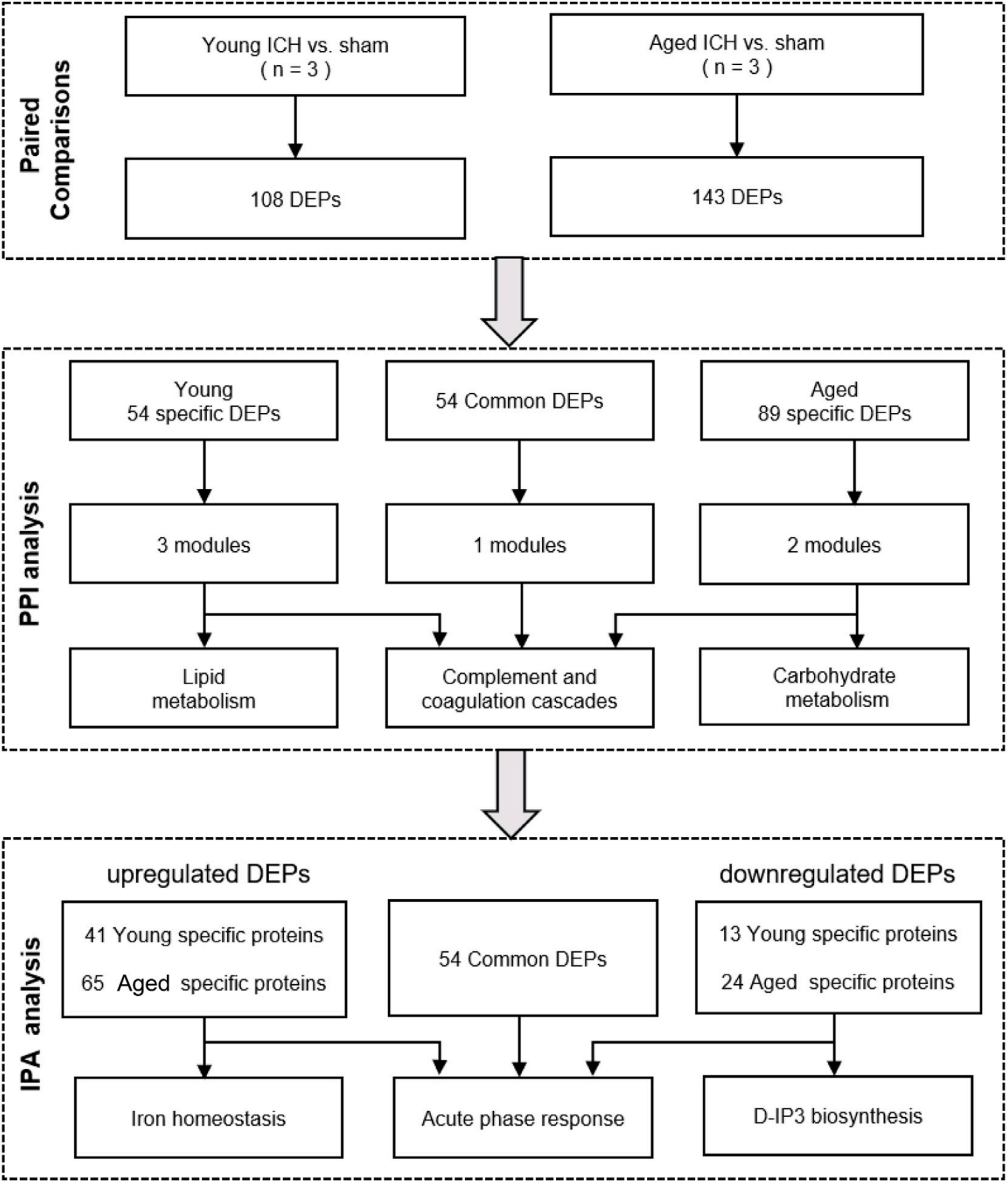
Bioinformatics analysis procedure.

The bioinformatics analysis procedure is shown in Figure 1. DEPs between the ICH and sham groups were identified by an FDR <0.05. The subcellular localization of DEPs was searched on CELLO^2^. The *p* values and fold change (FC) values of the DEPs were visualized in volcano plots. Hierarchical clustering analyses were conducted by cluster 3.0^3^ and Java Treeview^4^ and were visualized in heatmaps. The specific and common DEPs between different paired comparisons were identified by Venn plots^5^. DEP functional analysis was conducted by Gene Ontology (GO) analysis, and pathway analysis was performed by Kyoto Encyclopedia of Genes and Genomes (KEGG)^6^ and Ingenuity Pathway Analysis (IPA) software (IPA®, Qiagen, the United States)^7^. Protein–protein interaction (PPI) networks were constructed based on STRING^8^ and were further analyzed with Cytoscape software by the MCODE and CytoNCA plug-ins^9^.

## Results

### Identification of DEPs in young and aged mice after ICH

**FIGURE 2:**
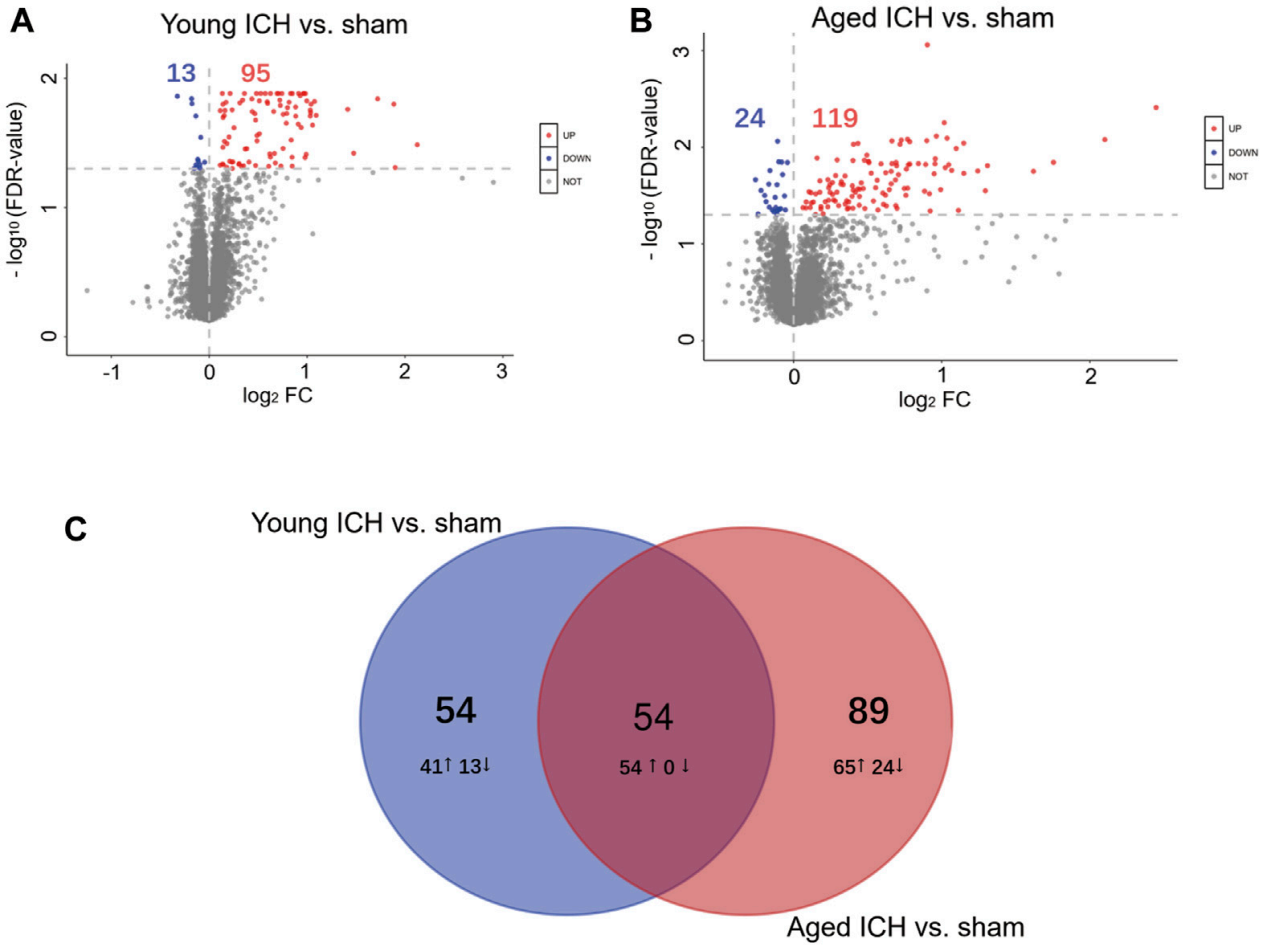
Identification of DEPs and PPI analysis. Volcano plots of DEPs between the ICH and sham groups in **(A)** young and **(B)** aged mice. **(C)** Venn diagram analysis of the ICH-induced DEPs between different age-matched comparisons.

The number of obtained spectra, peptides, and proteins are presented in Table 2. Specifically, the expression of a total of 5,129 proteins in the brains of young and aged mice was quantified, and the accession numbers, expression levels, sequence coverages and number of peptides identified are listed in Supplementary Tables S1, S2. Next, we examined the differences in the protein expression profiles of perihematomal tissues after ICH between young and aged mice by performing DEP analysis at 24 h after ICH and comparing the spectra with those of age-matched sham controls. When the DEPs were screened by FC > 1 and <1 and FDR <0.05, the FCs in DEPs between the ICH mice and sham controls in both age groups displayed significant discrepancies, as seen in the hierarchical clustering heatmaps, which grouped the samples from the ICH group and sham controls into two different clusters and enabled us to perform the following analysis (Supplementary Figures S1A, S1B). The full names of the DEPs in the heatmaps are presented in Supplementary Table S3. As depicted in the volcano plot (Figure 2A), ICH upregulated 95 and downregulated 13 proteins in young mice. In contrast, there were 119 upregulated proteins and 24 downregulated proteins in aged mice after ICH (Figure 2B). As shown in Figure 2C, common and unique DEPs were identified by a Venn diagram and included 54 unique DEPs in young mice (41 upregulated and 13 downregulated in the ICH group relative to controls), 89 unique DEPs in aged mice (65 upregulated and 24 downregulated in the ICH group relative to controls), and 54 common DEPs (all upregulated in the ICH group relative to controls) in the two age groups. To reduce the number of DEPs, FC > 2, FDR <0.05 was set as a stricter threshold to identify the most significant DEPs. The subcellular localization and biological function of the DEPs are described in detail in Supplementary Table S4.

**TABLE 2:** The identification and quantitation results.

Spectrum		Peptides		Proteins	
Total spectrum	Matched spectrum	Peptides	Unique peptides	Identified	Quantified
520,445	65,279	29,892	27,287	5,135	5,129

### Identification of specific and common DEP-related functions and pathways

To gain precise and thorough insight into the effect of aging and ICH, we explored the PPIs and performed module cluster analysis by GO enrichment and KEGG pathway analysis for specific and common DEPs in aged and young mice. To prevent the possibility of omitting meaningful functions and pathways due to false-negative results, the criterion for the identification of DEPs was once again set as FC > 1 and <1 and FDR <0.05.

**FIGURE 3:**
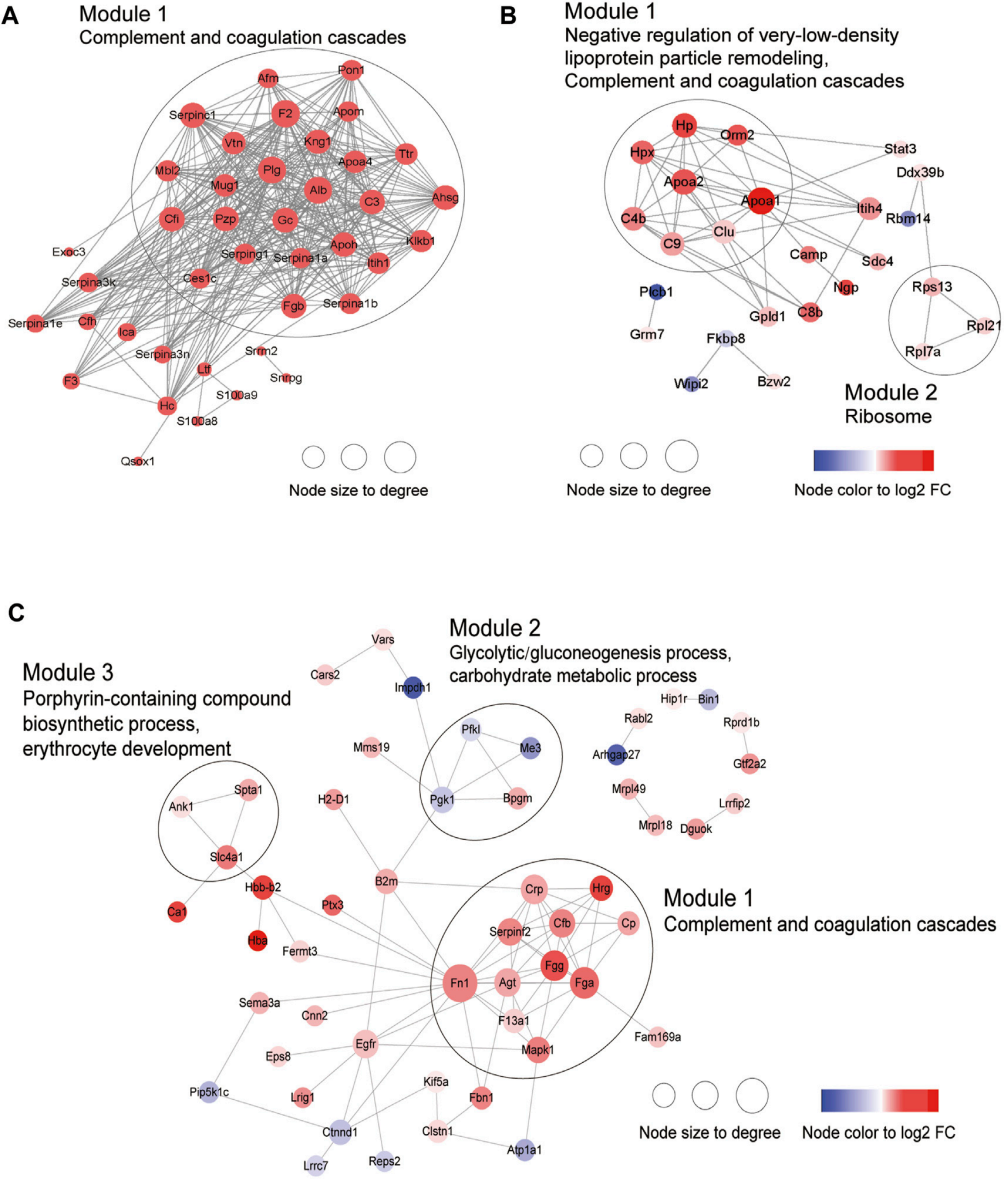
Construction of PPI network. **(A)** Common DEPs and **(B)** Specific DEPs in young and **(C)** aged mice after ICH.

Next, PPI analysis was conducted. As shown in Figure 3A, most of the common DEPs were clustered in one module, and their functions were associated with complement and coagulation cascades (Supplementary Table S5). The two modules in the PPI network constructed from unique DEPs in young mice were associated with negative regulation of the remodeling of very-low-density lipoprotein particles and ribosomal subunits (Figure 3B; Supplementary Table S5). In contrast, the DEPs constructed from unique DEPs in aged mice were divided into 3 modules, which were related to complement and coagulation cascades, glycolytic processes, and the biosynthetic processes of porphyrin-containing compounds (Figure 3C; Supplementary Table S5).

### Identification of specific and common up- or downregulated DEP-related functions and pathways

**FIGURE 4:**
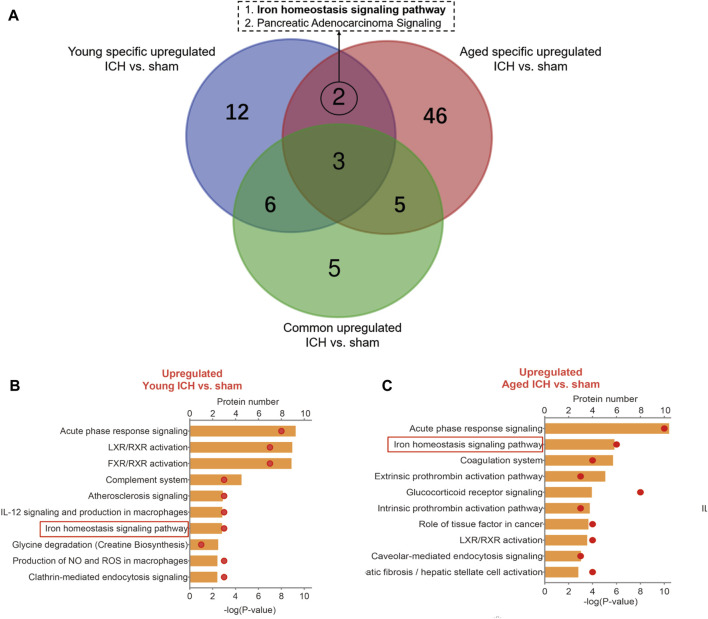
IPA analysis of specific upregulated DEPs. **(A)** The number of IPA pathways between specific and common upregulated DEPs in aged or young mice after ICH. **(B)** The top 10 IPA pathways in young and **(C)** aged mice after ICH compared with the corresponding sham controls.

**FIGURE 5:**
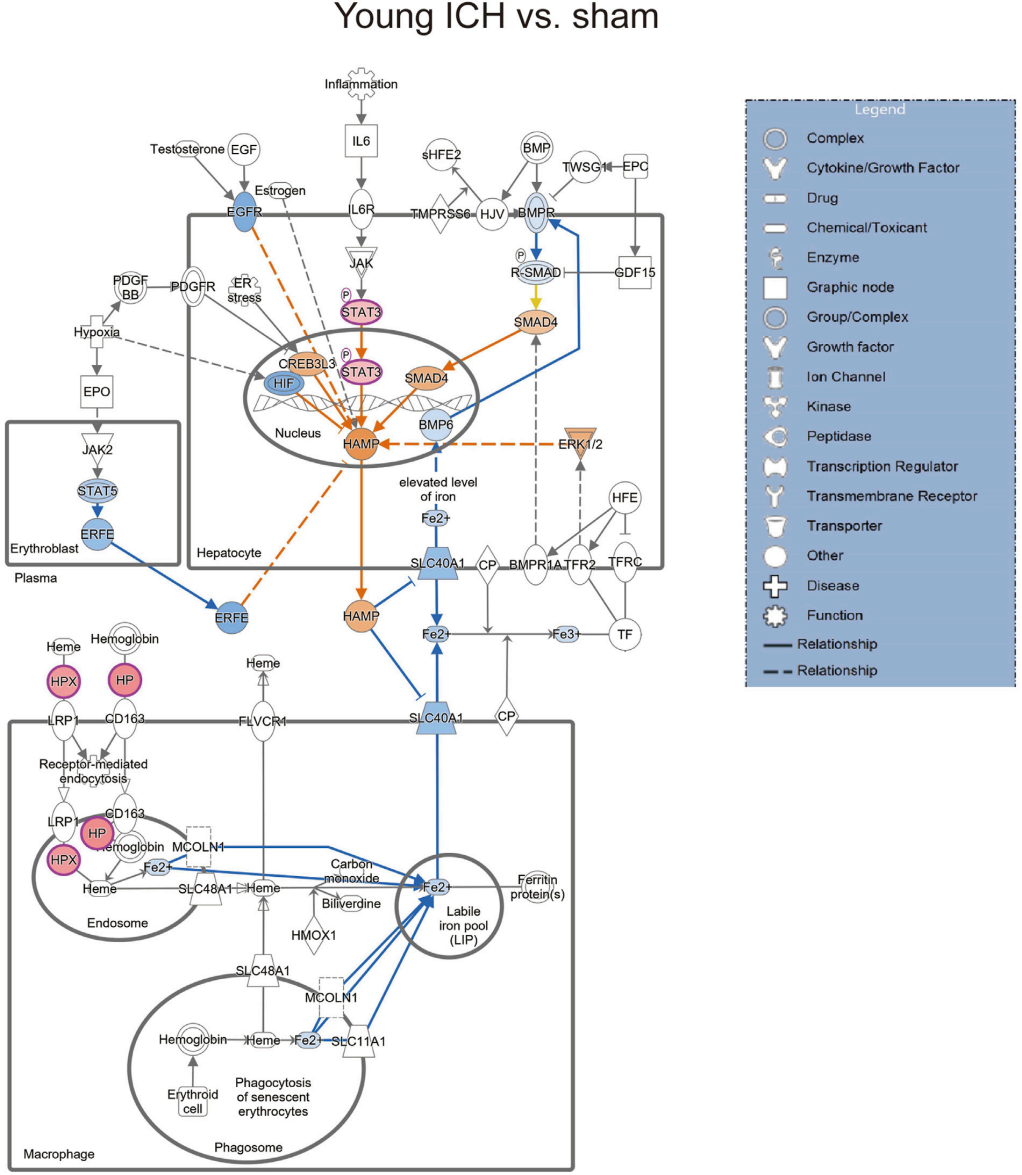
The iron homeostasis signaling pathway in young mice after ICH compared with the corresponding sham controls (by IPA software).

**FIGURE 6:**
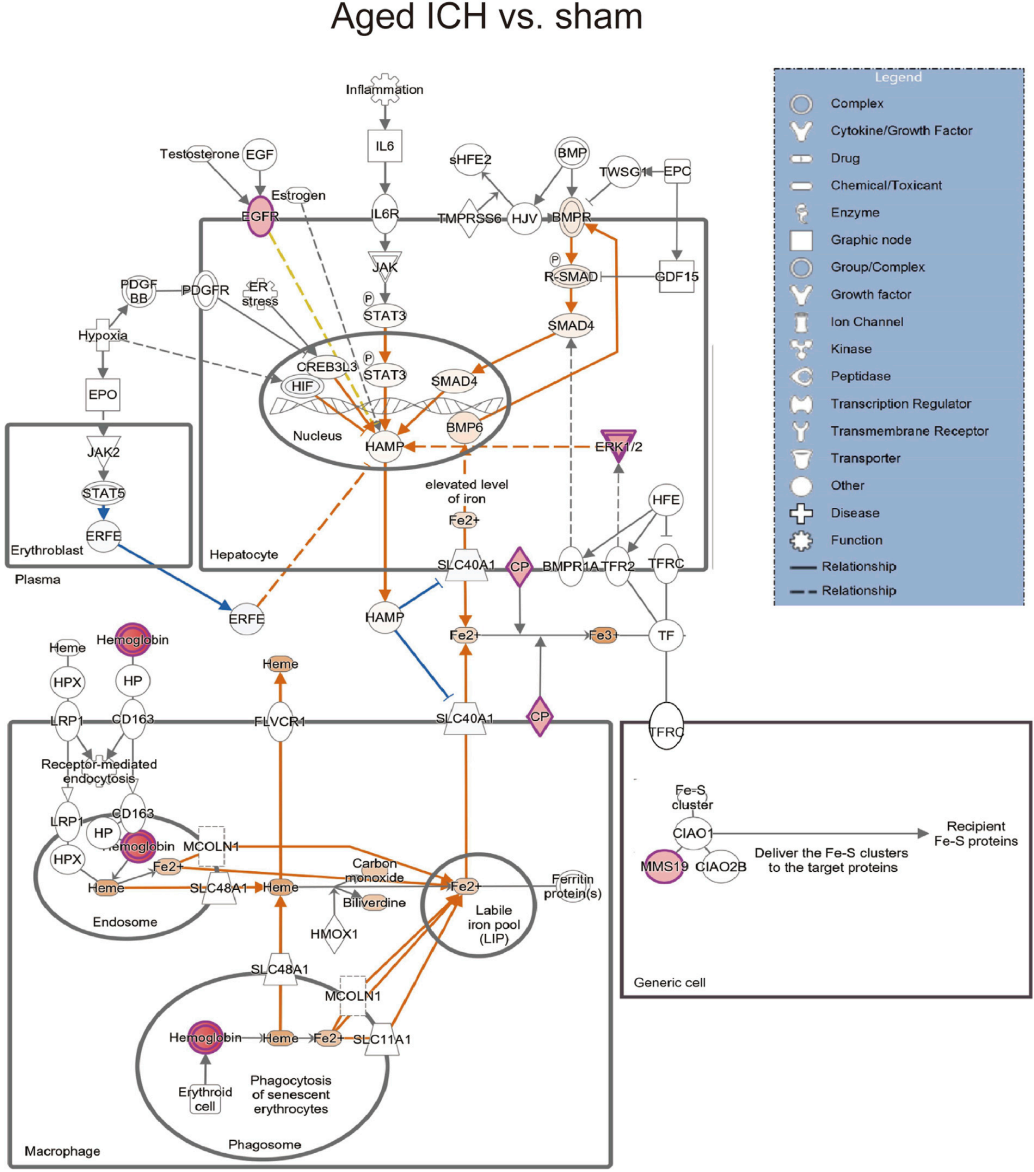
The iron homeostasis signaling pathway in aged mice after ICH compared with the corresponding sham controls (by IPA software).

Then, the ICH-induced DEPs in the two age groups were further divided into upregulated and downregulated DEPs for IPA. Consistent with the functional analysis of module clusters from PPI networks, biofunctional analysis conducted via IPA software demonstrated that lipid metabolism and carbohydrate embolism were significantly activated after ICH in young and aged mice, respectively (Supplementary Figures S2A, S2B). In the results for the IPA pathway enrichment of upregulated DEPs, the signaling pathways for iron homeostasis and pancreatic adenocarcinoma were the common activated pathways (Figures 4A–C; Supplementary Table S6). However, the proteins involved in the iron homeostasis signaling pathway were different. The proteins that participated in this pathway included STAT3, HPX, and HP from young mice and CP, EGFR, Hbb-b1, Hbb-b2, MAPK1, and MMS19 from aged mice (Figures 5, 6). In addition, regarding neurodegenerative diseases (NDDs), several functions, including Huntington’s disease signaling (EGFR, MAPK1, PSMB3), neuroprotection by THOP1 in Alzheimer’s disease (AGT, MAPK1) and Parkinson’s signaling (MAPK1), were activated only in aged mice (Supplementary Table S6). The shared pathways among the three clusters of DEPs included the complement and coagulation system and acute phase response, and this finding was consistent with the PPI analysis (Figures 4A–C; Supplementary Table S6). For the downregulated DEPs, D-IP3 biosynthesis and autophagy were the common functional pathways that were activated (Supplementary Table S6).

**FIGURE 7:**
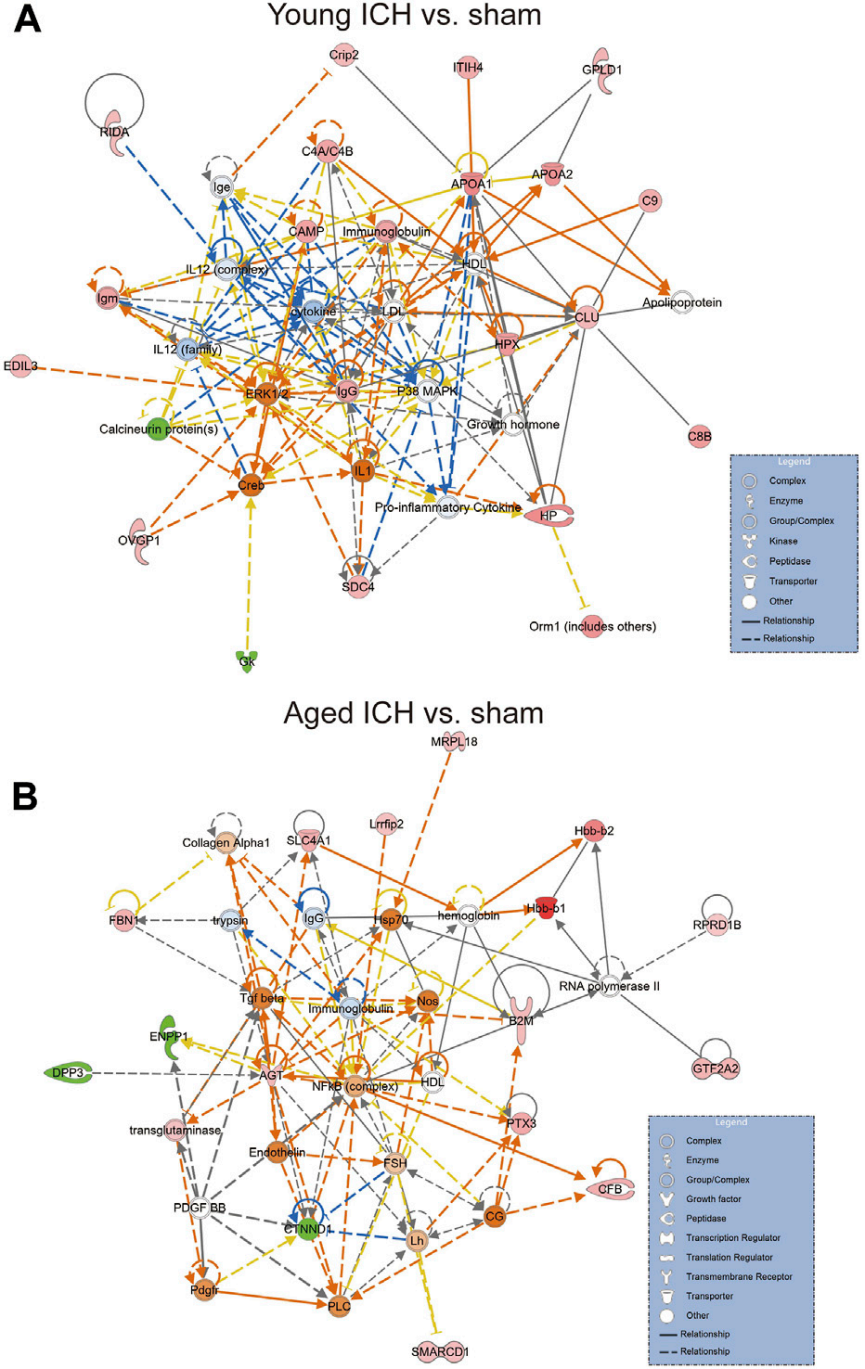
The internet interaction of DEPs in young and aged mice after ICH (by IPA software). **(A)** Young ICH vs. Young sham. **(B)** Aged ICH vs. Aged sham.

We next explored the proinflammatory factors that correlated with proteins of the iron homeostasis signaling pathway in both age groups. The networks obtained with the IPA software and constructed based on the direct and indirect relationships among the unique upregulated DEPs in the two age groups demonstrated that IL1 was the upstream molecule of HP in the young mice after ICH (Figure 7A) and that the NF-κB complex interacted with Hbb-b1 in aged mice after ICH (Figure 7B).

### Specific markers between aged and young mice after ICH

**FIGURE 8:**
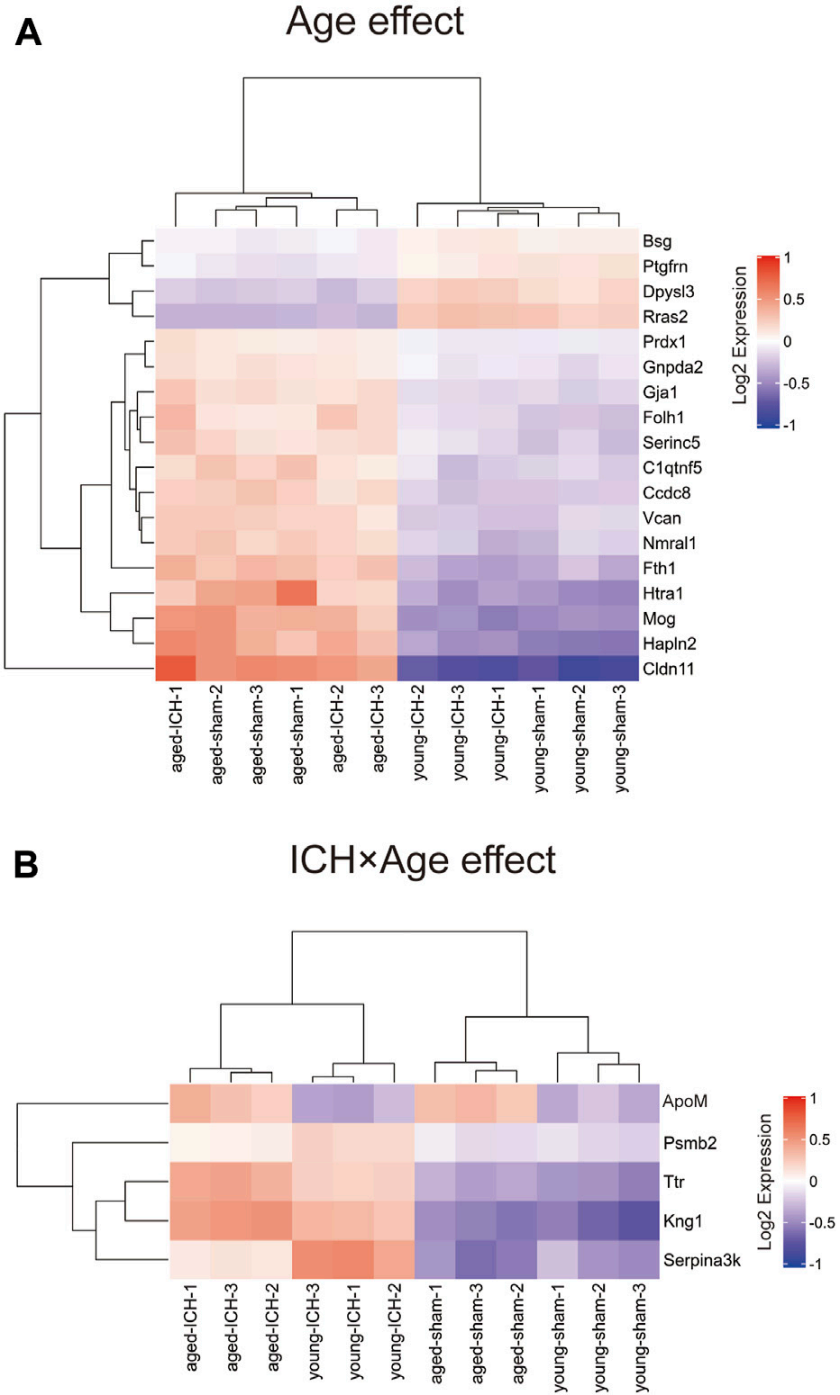
DEPs affected by age or ICH. **(A)** DEPs that were affected by age only. **(B)** DEPs that were affected by age and ICH.

Considering that the previous comparison was limited to ICH vs. sham, some important markers may have been missed. Thus, we explored different pairwise-comparison groups to explore the proteins affected by age alone and those affected by both age and ICH. As shown in Figures 8A, B, the expression of Bsg, Ptgfrn, Dpysl3, Rras2, Prdx1, Gnpda2, Gja1, Folh1, Serinc5, C1qtnf5, Ccdc8, Vcan, Nmral1, Fth1, Hlta1, Mog, Hapln2, and Cldn11 was affected by age only (18 in total), and that of ApoM, Psmb2, Ttr, Kng1, and Serpina3k was affected by both age and ICH (5 in total).

### DEP analysis between the aged and young sham groups

**FIGURE 9:**
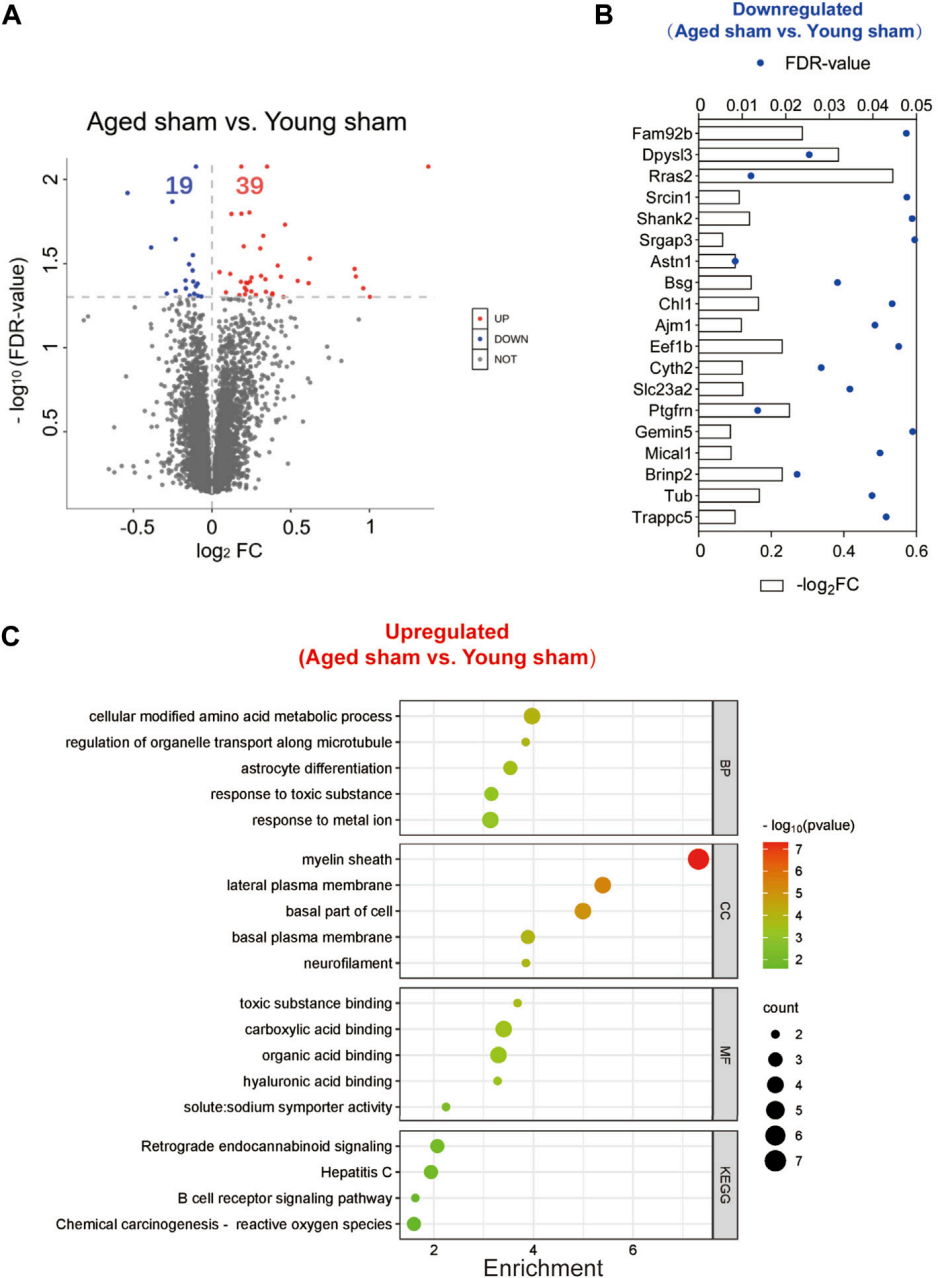
Identification of DEPs between the aged and young sham groups and functional enrichment analysis. **(A)** Volcano plot of the 58 DEPs in the aged sham group versus the young sham group. **(B)** Expression of the 19 downregulated DEPs between the two sham groups. **(C)** Top GO terms and KEGG pathways enriched in the 39 upregulated DEPs between the two sham groups.

To exclude the inherent differences between aged and young mice, we further compared the DEPs between the two sham groups. A total of 58 DEPs were identified between the aged and young sham groups of mice (Figure 9A; Supplementary Figure S3). The full names of the DEPs in the heatmap are presented in Supplementary Table S3. Aging downregulated 19 proteins in the basal ganglia, including the SH3 domain binding proteins Dpysl3, Shank2, and Mical1, the dendrite development-related proteins Srcin1 and Cyth2 and the neuron differentiation-related proteins Dpysl3, Shank2, Slc23a2 and Brinp2 (Figure 9B). Moreover, 39 proteins were upregulated in the brains of aged mice compared to those of young controls, and the functions of these proteins were identified by KEGG and GO enrichment analysis^11^ (last accessed on 10 November 2023, an online platform for data analysis and visualization) (Figure 9C; Supplementary Table S7) [22–24]. According to GO analysis, the DEPs were significantly enriched in 392 biological process (BP) terms (*p* < 0.05), with cellular modified amino acid metabolicprocess, regulation of organelle transport along microtubule, and astrocyte differentiation being the top BP terms. Furthermore, these DEPs were significantly enriched in 61 GO cellular component (CC) terms. Notably, the top CC terms included myelin sheath, lateral plasma membrane, and basal part of cell. Moreover, 71 molecular function (MF) terms were significantly overrepresented, including toxic substance binding, carboxylic acid binding, and organic acid binding. Additionally, KEGG pathway analysis revealed 11 significantly enriched pathways, including alanine, aspartate and glutamate metabolism, retrograde endocannabinoid signaling, and hepatitis C.

**FIGURE 10:**
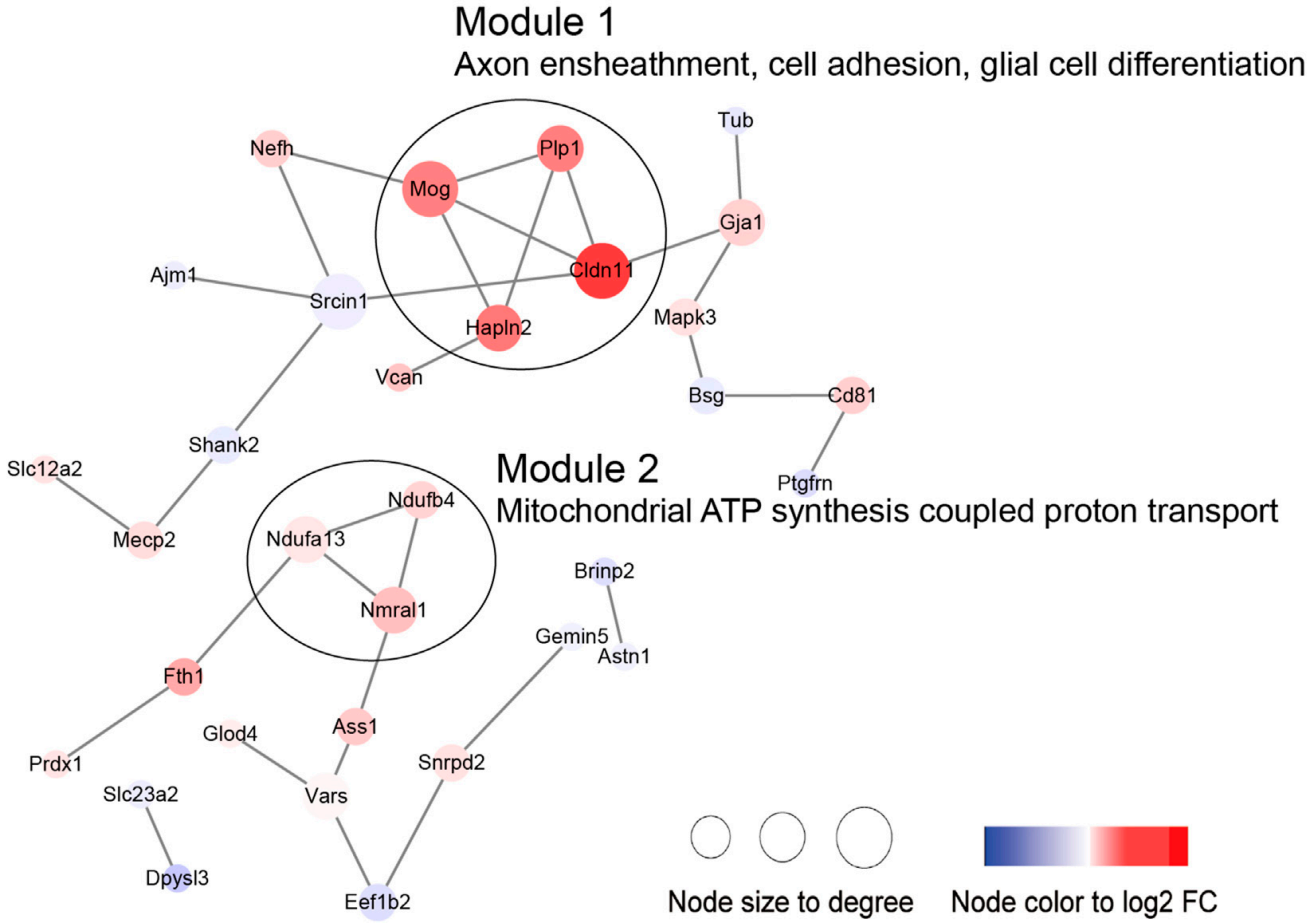
PPI network including the DEPs in the aged sham group versus the young sham group.

Then, we performed PPI and module analysis of all DEPs (Figure 10; Supplementary Table S5). The proteins in module 1 were primarily associated with GO-BP terms such as axon ensheathment, cell adhesion, and glial cell differentiation. On the other hand, the proteins in module 2 were mainly involved in mitochondrial ATP synthesis-coupled proton transport.

## Discussion

In the current study, we conducted LC–MS/MS analysis to examine changes in the protein profile of perihematomal tissue in response to ICH in the brains of aged and young mice. The functional analysis of age-specific DEPs and common DEPs in different ages implied that different age-related response patterns occurred at the acute phase of brain injury after ICH. Decoding aberrant protein expression patterns between young and aged groups of ICH mice can provide age-related internal vulnerable molecular mechanisms.

Specific and common DEPs in both age groups were associated with complement and coagulation cascades and the acute phase response, and these pathways are known as the vital BP that occurs with brain injury after ICH. Surprisingly, we found that the clustered DEPs that were specific to aged and young mice were enriched in glycolysis/gluconeogenesis processes and ribosomal subunits, respectively. Enhanced glycolysis/gluconeogenesis could promote the proinflammatory function of macrophages [25, 26]. In aged mice after ICH, decreased Pfkl (ATP-dependent 6-phosphofructokinase, liver type) and Pgk1 (phosphoglycerate kinase 1) proteins and increased Bpgm (bisphosphoglycerate mutase) protein were clustered together, and their functions involved glycolysis/gluconeogenesis and carbohydrate metabolic processes. Pfkl is a subtype of Pfk, and in a previous transcriptomics study, the elevated level of Pfk gene expression in CD14^+^ monocytes/macrophages from hematoma tissue was reported to be significantly associated with good outcomes for ICH patients at the subacute stage [27]. Clinical and preclinical data have shown that increased Pgk1 activity and glycolysis ameliorate the neurodegeneration and progression of Parkinson’s disease [28]. Bpgm plays an essential role in reversing aging-related cognitive impairment and auditory dysfunction [29]. Based on these findings, the significant change in glycolysis protein levels in the acute stage of ICH may be a specific predictor of poor outcomes. In addition, the other module from the aged-specific DEP PPI network included Spta1 (spectrin alpha chain, erythrocytic 1), Slc4a1 (band 3 anion transport protein), and Ank1 (ankyrin-1). In clinical research, the Slc4a1 and Ank1 genes from the peripheral blood of ICH patients were verified as hub genes by RNA sequence analysis, and this result implied that these genes have the potential to be used as key intervention targets in translational research [30].

In contrast, lipid metabolism was significantly affected in the young mice. Oxylipin profiling at 12 h, 24 h, and 72 h after ICH in adult mouse brains was explored in a recent study, and the number of changed oxylipins was the highest on the first day of ICH [10]. In the present study, the expression of the apolipoprotein A-I (Apoa1) protein differed between young and aged ICH model mice, being significantly elevated exclusively in young mice. This discrepancy may be associated with the weaker response to ICH in aged mice. Apoa1, as a component of high-density lipoprotein, may exert a protective effect by reducing cholesterol deposition and inhibiting inflammatory responses. Previous studies have also identified it as a protective factor in ICH, supporting this hypothesis [31, 32]. Consequently, in aged mice and even patients with ICH, greater attention should be paid to changes in the level of this indicator and related treatments. However, the interaction between lipid metabolism and ICH is still worthy of further exploration. In addition, the levels of Rps13 (40S ribosomal protein S13), Rpl21 (60S ribosomal protein L21), and Rpl7a (60S ribosomal protein L7a) genes were elevated, and these genes interacted with each other in the PPI network that was constructed by DEPs from young mice after ICH. The genes are all components of ribosomal subunits. Ribosomal damage could cause the TP53 pathway to be disrupted and increase ROS [33], which have a close link with secondary brain injury after ICH [34]. Significantly, Rpl7a was identified as a hub gene in a murine model of traumatic brain injury [35], and increased Rps13 gene levels in microglia and epithelial cells were found to be aging-related in a single-cell sequence study on murine brains [36]. In our study, the elevated ribosomal-associated proteins functioned as transcriptional proteins, and they may play a role in brain injury in young mice after ICH.

Secondary brain injury includes erythrocyte lysis with the production of neurotoxic iron ions, immune-inflammatory reactions, and coagulation cascades, which result in nerve cell death and brain tissue necrosis [37]. Previous studies have shown that the distribution of iron in the healthy human brain is region-specific and that its concentration increases with age [38, 39]. The putamen nucleus, caudate nucleus, and globus pallidus in the basal ganglia are the brain regions with the highest concentration of iron [40]. The increase in the number of glial cells and the permeability of the blood–brain barrier with age may explain the accumulation of iron in the basal ganglia. In the present proteomics analysis, we found that the signaling pathway of iron homeostasis was activated after ICH in both aged and young mice. Interestingly, the number and type of proteins that were activated in the pathway were significantly higher in the aged mice after ICH than in the young mice. Following ICH, the proteins involved in iron metabolism in young mice include HP (haptoglobin), HPX (hemopexin), and STAT3 (signal transducer and activator of transcription 3). On the other hand, in aged mice, the proteins related to iron metabolism were CP (ceruloplasmin), EGFR (epidermal growth factor receptor), Hbb-b1 (hemoglobin subunit beta-1), Hbb-b2 (hemoglobin subunit beta-2), MAPK1 (mitogen-activated protein kinase 1), and MMS19 (MMS19 nucleotide excision repair protein). HP and HPX and CP are plasma proteins synthesized by the liver that can bind to hemoglobin, heme and copper, respectively. Previous studies have suggested that increasing HPX and CP levels or reducing HP levels could be potential strategies for the treatment of experimental ICH [41–43]. Specifically, the therapeutic effect of HP gene knockout on ICH was age dependent, with Hp depletion resulting in a better neurological outcome in young ICH model mice but being ineffective in aged ICH model mice. Similarly, another experiment on age-related memory decline showed that specific knockout of CP in astrocytes had a beneficial effect on aged mice but had detrimental effects on young mice [44]. These results emphasize the importance of considering age-related factors when developing strategies for protecting the brain and indicate that the underlying mechanisms of ICH and response to treatment may differ between young and aged individuals. When iron metabolism becomes dysfunctional, excess iron and hydroxyl radicals are produced, resulting in OS damage and neuronal injury or death [45]. Previous studies based on an ICH model with aged murine and aged rats indicated that mediating iron metabolism and reducing iron accumulation could alleviate neuronal death and neurological deficits [46, 47]. The present results suggest that after ICH in aged individuals, iron metabolism is a crucial process in the brain injury mechanism. In addition, several DEPs in the aged mice were enriched in NDD-related pathways, which suggests a possible interactive correlation between ICH and NDD in pathological mechanisms.

To select meaningful molecular markers for validation, we performed additional pairwise comparisons. Among the proteins altered by age alone, Cldn11 (claudin-11) and Mog(myelinoligodendrocyte glycoprotein are myelin-associated proteins. ApoM (apolipoprotein M), Psmb2 (proteasome subunit beta type-2), Ttr (transthyretin), Kng1 (kininogen-1) and Serpina3k (serine protease inhibitor A3K) were altered by both age and ICH. Indeed, disruption of myelin-related molecules with age has been demonstrated in previous experiments [48]. ApoM binding to sphingosine-1-phosphate has been reported to have inhibitory effects on lymphopoiesis and neuroinflammation [49]. Treatments targeting Ttr and Serpina3k exerted significant protective effects against experimental traumatic brain injury [50, 51]. Kng1 is involved in regulating vasodilation and inflammatory responses [52], while Psmb1 functions in the degradation and processing of intracellular proteins [53]. The dysregulation of these two proteins may indicate a pronounced change in the inflammatory response due to age and ICH. Considering these findings together, the higher Serpina3k protein level in younger mice may explain the better long-term prognosis of younger subjects after ICH, and the higher ApoM and Ttr levels in elderly subjects with ICH may be due to a compensatory mechanism for protecting against acute brain injury after ICH.

The inclusion of the two sham groups in this study has further improved our understanding of the impact of aging on the brain. Aging is a complex BP involving changes at multiple levels, including disruptions of cellular metabolism [54]. According to our results, the dysregulation of modified amino acid metabolism within cells is associated with aging. During the aging process, the metabolic activity and regulatory capacity of cells gradually decline, which can lead to abnormalities in the synthesis, degradation, and conversion of modified amino acids [55]. For instance, research has found that in aging cells, there is an abnormal increase or decrease in processes such as methylation and acetylation, which can result in changes in protein function and aberrant cellular metabolism [56]. Additionally, alterations in the expression of myelin proteins and changes in the regulation of proteins involved in myelination during aging were observed. Myelin plays a crucial role in facilitating efficient nerve signal transmission, and any disturbance of or deterioration in its structure or composition may impact neural communication [57]. A previous study demonstrated that significant alterations occurred in sphingolipid expression patterns associated with myelin remodeling during the aging process [58]. With advancing age, mitochondrial ATP synthesis coupled proton transport was also affected. This could be attributed to changes in the internal structure and function of mitochondria, including mitochondrial DNA damage, membrane instability, and increased oxidative stress [59–61]. These factors may contribute to a decrease in the mitochondrial membrane potential, consequently affecting the efficiency of proton pump function and ATP synthesis.

Taken together, the results of our current study provide information on specific DEPs and their functions in mice of different ages after ICH, contribute to the exploration of the age-specific approach for ICH and provide foundations for future research on the subject.

## Data Availability

The original contributions presented in the study are included in the article/Supplementary Material, further inquiries can be directed to the corresponding authors. The MS proteomics data have been deposited in the ProteomeXchange Consortium http://proteomecentral.proteomexchange.org via the iProX partner repository with the dataset identifier PXD033791 [62].

## Footnotes

1
https://www.uniprot.org/

2
http://cello.life.nctu.edu.tw/

3
http://bonsai.hgc.jp/∼mdehoon/software/cluster/software.htm

4
http://jtreeview.sourceforge.net

5
http://bioinformatics.psb.ugent.be/webtools/Venn/

6
https://david.ncifcrf.gov/summary.jsp

7
https://www.qiagenbioinformatics.com

8
https://string-db.org

9
https://apps.cytoscape.org/apps/

11
https://www.bioinformatics.com.cn
