# Supplementary material for: Age-related changes after intracerebral hemorrhage: a comparative proteomics analysis of perihematomal tissue
Source: Exp Biol Med (Maywood). 2024 Mar 25;249:10117. doi: 10.3389/ebm.2024.10117 (PMC11001198; doi:10.3389/ebm.2024.10117)
Supplement: Supplementary file 1 [file DataSheet1.pdf]

Supplementary Table 1 | The overview of all the proteins quantified. (Coverage, the percentage of the protein sequence covered by identified peptides; Unique Peptides, the number of peptide sequences unique to a protein group; PSMs, peptide spectrum matches, the total number of identified peptide sequences for the protein, including those redundantly identified; AAs, the number of amino acids in the protein; WM, calculated molecular weight of the protein; calc. pI, calculated protein isoelectric point.)

Accession	Gene Name	Coverage	Unique Peptides	Peptides	PSMs	AAs	MW [kDa]	calc. pI
P16546	Sptan1	65	151	152	613	2472	284.4	5.33
Q9QXS1	Plec	38	170	173	360	4691	533.9	5.96
Q62261	Sptbn1	55	116	119	402	2363	274.1	5.58
Q9JHU4	Dync1h1	31	130	130	286	4644	531.7	6.42
Q6PIC6	Atp1a3	58	33	51	848	1013	111.6	5.41
Q9QYR6	Map1a	38	100	100	294	2776	300	5
P20357	Map2	50	80	80	254	1828	199	4.91
O88737	Bsn	34	100	103	211	3942	418.6	7.71
Q68FD5	Cltc	50	64	64	352	1675	191.4	5.69
Q8C8R3	Ank2	29	85	90	179	3898	426	5.17
Q61879	Myh10	47	73	90	209	1976	228.9	5.54
Q8VDN2	Atp1a1	43	27	45	599	1023	112.9	5.45
Q6PIE5	Atp1a2	54	33	52	545	1020	112.1	5.55
P14873	Map1b	36	68	68	179	2464	270.1	4.83
Q9QXZ0	Macf1	14	87	90	146	7354	831.4	5.43
P07724	Alb	71	41	41	288	608	68.6	6.07
Q9R0K7	Atp2b2	43	36	48	200	1198	132.5	5.96
Q8VDD5	Myh9	36	47	63	125	1960	226.2	5.66
P60710	Actb	68	12	23	620	375	41.7	5.48
P52480	Pkm	60	33	34	315	531	57.8	7.47
G5E829	Atp2b1	33	25	42	181	1220	134.7	5.91
Q99104	Myo5a	30	56	56	134	1853	215.4	8.63
Q9QYX7	Pclo	17	65	68	118	5068	550.5	6.51
Q7TPR4	Actn1	54	27	43	167	892	103	5.38
P56480	Atp5f1b	66	27	27	178	529	56.3	5.34
Q03265	Atp5f1a	56	31	31	440	553	59.7	9.19
Q8CAQ8	Immt	57	45	45	119	757	83.8	6.61
P46460	Nsf	49	39	40	191	744	82.6	6.95
O08553	Dpysl2	72	24	28	348	572	62.2	6.38
O08599	Stxbp1	59	36	36	183	594	67.5	6.96
Q7TMM9	Tubb2a	61	1	24	479	445	49.9	4.89
P17182	Eno1	66	23	27	415	434	47.1	6.8
P63017	Hspa8	63	23	38	183	646	70.8	5.52
O55143	Atp2a2	33	33	33	147	1044	114.8	5.34
Q9CWF2	Tubb2b	61	1	24	456	445	49.9	4.89
Q99KI0	Aco2	53	34	34	171	780	85.4	7.93

P99024	Tubb5	62	4	24	450	444	49.6	4.89
P07901	Hsp90aa1	50	24	37	193	733	84.7	5.01
P08553	Nefm	51	42	47	167	848	95.9	4.77
P39053	Dnm1	45	27	37	216	867	97.7	7.74
Q7TSJ2	Map6	64	47	47	127	906	96.4	9.5
P68369	Tuba1a	66	4	25	460	451	50.1	5.06
P68372	Tubb4b	58	1	24	411	445	49.8	4.89
Q71LX4	Tln2	30	45	52	85	2375	253.5	5.8
P68368	Tuba4a	65	6	24	394	448	49.9	5.06
P46660	Ina	69	33	38	226	501	55.3	5.4
P17710	Hk1	40	36	39	150	974	108.2	6.8
P11499	Hsp90ab1	51	21	36	182	724	83.2	5.03
Q9D6F9	Tubb4a	59	8	23	377	444	49.6	4.88
P05063	Aldoc	73	21	28	164	363	39.4	7.12
P63038	Hspd1	72	32	32	114	573	60.9	6.18
P05064	Aldoa	74	21	28	248	364	39.3	8.09
P17426	Ap2a1	41	22	34	120	977	107.6	7.03
Q9ERD7	Tubb3	57	8	22	349	450	50.4	4.93
Q01853	Vcp	48	32	32	120	806	89.3	5.26
P60879	Snap25	74	19	20	124	206	23.3	4.77
P19096	Fasn	23	45	45	87	2504	272.3	6.58
Q9Z1B3	Plcb1	34	38	38	84	1216	138.3	6.13
P08551	Nefl	64	32	36	143	543	61.5	4.64
P16330	Cnp	57	25	26	207	420	47.1	8.97
Q61316	Hspa4	59	39	41	100	841	94.1	5.24
P57780	Actn4	44	24	36	122	912	104.9	5.41
P05214	Tuba3a	54	0	20	349	450	49.9	5.1
P63101	Ywhaz	80	14	21	348	245	27.8	4.79
Q8CHC4	Synj1	26	36	36	90	1574	172.5	6.89
Q6Q477	Atp2b4	22	14	27	124	1205	133	6.13
Q02053	Uba1	34	31	31	98	1058	117.7	5.66
Q99P72	Rtn4	43	32	32	85	1162	126.5	4.54
Q8BPN8	Dmxl2	17	44	45	79	3032	338	6.42
P12960	Cntn1	37	30	30	97	1020	113.3	6.16
O08788	Dctn1	33	37	37	72	1281	141.6	5.9
P48722	Hspa4l	52	34	35	79	838	94.3	5.74
Q9WV92	Epb41l3	52	34	39	96	929	103.3	5.31
P19246	Nefh	31	35	39	93	1090	116.9	5.81
Q04447	Ckb	63	19	19	390	381	42.7	5.67
P38647	Hspa9	51	32	33	90	679	73.4	6.07
P17183	Eno2	71	18	24	227	434	47.3	5.11
P62259	Ywhae	80	19	22	194	255	29.2	4.74
P13595	Ncam1	41	33	33	110	1115	119.4	4.83
Q3UHJ0	Aak1	42	27	27	64	959	103.3	6.7

P50516	Atp6v1a	47	27	27	112	617	68.3	5.58
Q91XV3	Basp1	93	22	22	125	226	22.1	4.51
P16858	Gapdh	67	17	17	326	333	35.8	8.25
P17427	Ap2a2	38	21	33	81	938	104	6.93
Q921I1	Tf	50	33	34	99	697	76.7	7.18
P62737	Acta2	54	7	18	300	377	42	5.39
P15508	Sptb	23	40	43	67	2128	245.1	5.33
P09411	Pgk1	63	21	21	129	417	44.5	7.9
Q9JMH9	Myo18a	23	40	40	66	2050	232.6	6.28
P11798	Camk2a	42	14	20	176	478	54.1	7.08
Q64727	Vcl	45	40	40	67	1066	116.6	6
P26443	Glud1	46	25	25	112	558	61.3	8
Q9QWI6	Srcin1	33	34	34	64	1250	134.8	9.32
P03995	Gfap	70	28	30	112	430	49.9	5.34
D3YVF0	Akap5	57	26	26	70	745	79.4	4.75
Q91VD9	Ndufs1	41	26	26	86	727	79.7	5.72
Q61644	Pacsin1	55	24	26	114	441	50.5	5.24
Q4KMM3	Oxr1	38	27	27	66	866	95.9	5.33
O88935	Syn1	45	21	23	137	706	74.1	9.8
Q3UNH4	Gprin1	47	45	45	85	932	95.4	7.93
Q9JI91	Actn2	36	18	29	83	894	103.8	5.45
Q8BL66	Eea1	34	43	43	63	1411	160.8	5.77
P58281	Opa1	35	31	31	72	960	111.3	7.55
P42932	Cct8	56	28	28	75	548	59.5	5.62
Q9DBG3	Ap2b1	34	15	31	81	937	104.5	5.38
P26039	Tln1	18	32	39	58	2541	269.7	6.18
P01027	C3	22	34	34	66	1663	186.4	6.73
Q9ES97	Rtn3	37	27	27	63	964	103.8	4.92
P70336	Rock2	30	39	44	70	1388	160.5	5.99
Q80TJ1	Cadps	28	25	31	64	1355	153	5.74
P20029	Hspa5	40	22	25	95	655	72.4	5.16
Q60932	Vdac1	75	18	18	212	296	32.3	8.43
P61982	Ywhag	84	12	20	184	247	28.3	4.89
Q8CI94	Pygb	41	26	32	74	843	96.7	6.73
Q8K1M6	Dnm1l	46	30	30	71	742	82.6	7.05
Q8BYI9	Tnr	24	27	27	65	1358	149.5	4.94
P28652	Camk2b	50	13	21	91	542	60.4	7.28
P16125	Ldhb	50	16	18	114	334	36.5	6.05
P08249	Mdh2	68	19	19	152	338	35.6	8.68
Q8BH59	Slc25a12	43	23	23	73	677	74.5	8.25
Q6ZWR6	Syne1	6	46	46	53	8799	1009.3	5.59
P62814	Atp6v1b2	54	23	23	112	511	56.5	5.81
P68510	Ywhah	81	13	19	161	246	28.2	4.89
O55131	Septin7	49	23	24	86	436	50.5	8.57

P48678	Lmna	45	31	32	65	665	74.2	6.98
Q8BZ98	Dnm3	30	16	27	91	863	97.1	8.35
P63328	Ppp3ca	48	16	22	118	521	58.6	5.86
Q9Z2I9	Sucla2	50	20	20	70	463	50.1	7.01
Q9CQV8	Ywhab	69	7	16	139	246	28.1	4.83
P50396	Gdi1	54	17	19	80	447	50.5	5.08
O08532	Cacna2d1	28	26	27	57	1103	124.6	5.3
P14824	Anxa6	43	26	28	63	673	75.8	5.5
O70318	Epb41l2	31	29	33	61	988	109.9	5.43
Q9DBJ1	Pgam1	76	16	16	82	254	28.8	7.18
Q9Z1G4	Atp6v0a1	21	18	18	67	839	96.4	6.76
P28738	Kif5c	36	18	30	60	956	109.2	6.19
Q810U3	Nfasc	24	26	26	70	1240	137.9	6.19
Q91ZU6	Dst	6	39	41	55	7393	833.7	5.31
Q60864	Stip1	52	31	31	74	543	62.5	6.8
P18872	Gnao1	56	16	17	106	354	40.1	5.53
Q9CZ13	Uqcrc1	41	16	17	69	480	52.8	6.21
P28663	Napb	58	15	17	67	298	33.5	5.47
P20152	Vim	54	21	27	78	466	53.7	5.12
P60469	Ppfia3	31	30	32	54	1194	133.3	5.64
Q8QZT1	Acat1	50	17	17	57	424	44.8	8.51
Q9QXS6	Dbn1	35	22	22	67	706	77.2	4.49
Q7TQF7	Amph	33	20	22	70	686	75	4.63
Q05920	Pc	28	30	30	67	1178	129.6	6.71
Q6ZQ38	Cand1	23	27	27	70	1230	136.2	5.78
O08638	Myh11	15	13	26	47	1972	226.9	5.45
Q8BGQ7	Aars	34	25	25	50	968	106.8	5.67
Q6URW6	Myh14	19	27	32	43	2000	228.4	5.55
P68254	Ywhaq	67	12	18	123	245	27.8	4.78
P47857	Pfkm	33	18	20	42	780	85.2	8
Q61301	Ctnna2	30	17	22	46	953	105.2	5.71
P63318	Prkcg	37	22	25	71	697	78.3	7.46
Q62108	Dlg4	28	21	24	67	724	80.4	5.87
P11983	Tcp1	47	20	20	54	556	60.4	6.16
P28660	Nckap1	27	25	25	62	1128	128.7	6.62
P43006	Slc1a2	30	16	16	118	572	62	6.68
Q922F4	Tubb6	39	4	16	219	447	50.1	4.89
P11881	Itpr1	10	23	30	57	2749	313	6.04
Q91V92	Acly	25	24	24	52	1091	119.7	7.44
Q9JJZ2	Tuba8	48	3	16	136	449	50	5.1
P15105	Glul	46	16	16	75	373	42.1	7.08
Q8C419	Gpr158	27	25	25	43	1200	134.3	8.09
O35643	Ap1b1	26	8	24	70	943	103.9	5.17
Q9CZW5	Tomm70	41	25	25	59	611	67.5	7.53

Q91V14	Slc12a5	22	20	24	55	1138	126.2	6.74
Q9R111	Gda	41	19	19	57	454	51	5.53
P40142	Tkt	37	18	18	52	623	67.6	7.5
P97427	Crmp1	47	16	20	76	572	62.1	7.12
P0DP27	Calm2	77	13	13	238	149	16.8	4.22
G5E8K5	Ank3	18	22	27	47	1961	213.9	6.7
Q3UHL1	Camkv	50	19	19	65	512	54.8	5.54
P80315	Cct4	42	17	17	43	539	58	8.02
P68404	Prkcb	35	16	23	63	671	76.7	7.01
P48962	Slc25a4	64	9	20	122	298	32.9	9.72
Q9DB77	Uqcrc2	44	16	16	50	453	48.2	9.25
Q91XM9	Dlg2	36	25	28	52	852	94.8	6.24
Q76MZ3	Ppp2r1a	49	23	23	57	589	65.3	5.11
Q8K2B3	Sdha	39	20	20	59	664	72.5	7.37
P17751	Tpi1	59	14	14	70	299	32.2	5.74
Q9EPN1	Nbea	12	25	28	48	2936	326.5	6.2
P05202	Got2	42	19	19	121	430	47.4	9
P10126	Eef1a1	43	10	17	74	462	50.1	9.01
P97807	Fh	47	20	20	66	507	54.3	9.04
P51881	Slc25a5	74	10	21	117	298	32.9	9.73
P58252	Eef2	29	22	23	64	858	95.3	6.83
P80314	Cct2	49	21	21	53	535	57.4	6.4
Q91ZX7	Lrp1	8	36	36	53	4545	504.4	5.36
P21619	Lmnb2	42	25	26	57	596	67.3	5.5
Q8K0U4	Hspa12a	37	22	22	57	675	74.8	6.77
P10637	Mapt	34	20	20	59	733	76.2	6.79
Q8BRT1	Clasp2	26	27	30	51	1286	140.7	8.63
P23819	Gria2	30	19	25	48	883	98.6	7.39
P61264	Stx1b	47	14	16	90	288	33.2	5.38
Q9QYC0	Add1	32	18	19	49	735	80.6	5.9
Q8R0Y6	Aldh1l1	36	27	27	48	902	98.6	5.91
E9Q3L2	Pi4ka	15	26	26	43	2105	236.9	7.06
P05201	Got1	56	21	21	62	413	46.2	7.14
Q64332	Syn2	43	14	17	66	586	63.3	8.43
Q62420	Sh3gl2	43	11	15	77	352	39.9	5.39
Q9WUA3	Pfkp	35	21	23	54	784	85.4	7.11
P56399	Usp5	31	22	22	55	858	95.8	5.01
P46096	Syt1	48	13	19	69	421	47.4	8.53
Q9WV27	Atp1a4	13	2	12	190	1032	114.8	5.71
Q61768	Kif5b	30	16	26	42	963	109.5	6.44
Q8BWF0	Aldh5a1	47	19	19	41	523	55.9	8.25
Q8CGC7	Eprs	20	25	25	36	1512	170	7.66
Q8BFR5	Tufm	46	19	19	48	452	49.5	7.56
Q61699	Hsph1	36	25	27	53	858	96.3	5.53

Q91VR2	Atp5f1c	47	12	12	75	298	32.9	9.01
Q9CWZ7	Napg	61	18	18	54	312	34.7	5.41
Q6PH08	Erc2	26	24	27	44	957	110.6	7.03
Q61292	Lamb2	17	26	27	45	1799	196.5	6.67
D3Z7P3	Gls	35	20	20	48	674	73.9	7.99
A2AGT5	Ckap5	13	25	25	49	2032	225.5	7.96
Q99KJ8	Dctn2	49	17	17	46	402	44.1	5.26
Q61553	Fscn1	50	21	21	59	493	54.5	6.89
F6SEU4	Syngap1	20	22	24	50	1340	148.1	8.98
P17156	Hspa2	34	7	23	84	633	69.6	5.67
Q6P9K8	Caskin1	24	23	24	39	1431	150.4	9.17
Q9Z2Q6	Septin5	37	14	16	47	369	42.7	6.67
Q8K596	Slc8a2	20	14	14	52	921	100.6	5.12
P14733	Lmnb1	41	23	24	43	588	66.7	5.16
P14094	Atp1b1	42	16	16	116	304	35.2	8.65
P62874	Gnb1	49	7	13	81	340	37.4	6
Q64521	Gpd2	31	21	21	53	727	80.9	6.61
P08113	Hsp90b1	24	18	20	65	802	92.4	4.82
Q9Z0H8	Clip2	24	20	24	41	1047	115.8	6.48
Q60598	Cttn	38	22	22	44	546	61.2	5.4
Q62188	Dpysl3	41	14	17	71	570	61.9	6.49
Q61595	Ktn1	19	23	23	37	1327	152.5	5.86
Q9DB05	Napa	57	12	14	44	295	33.2	5.45
P06151	Ldha	49	13	15	83	332	36.5	7.74
Q8BKX1	Baiap2	45	21	21	39	535	59.2	9.04
P62631	Eef1a2	43	11	18	67	463	50.4	9.03
P80313	Cct7	41	17	17	48	544	59.6	7.84
Q9Z0E0	Ncdn	31	17	17	59	729	78.8	5.54
Q6PHZ2	Camk2d	33	7	14	77	499	56.3	7.25
Q9Z2H5	Epb41l1	31	21	23	49	879	98.3	5.62
Q61753	Phgdh	30	13	13	43	533	56.5	6.54
O08539	Bin1	39	19	21	47	588	64.4	5.03
P62880	Gnb2	42	4	12	84	340	37.3	6
P14152	Mdh1	42	16	16	87	334	36.5	6.58
P40124	Cap1	46	19	19	48	474	51.5	7.52
Q8BWT1	Acaa2	63	15	15	27	397	41.8	8.09
E9Q401	Ryr2	7	32	32	42	4966	564.5	6.09
Q8BTM8	Flna	13	27	28	36	2647	281	6.04
Q9QYB8	Add2	27	14	15	42	725	80.6	6.21
Q11011	Npepps	23	21	21	47	920	103.3	5.9
P27773	Pdia3	38	18	18	46	505	56.6	6.21
Q80SW1	Ahcyl1	34	9	17	48	530	58.9	6.89
P33173	Kif1a	17	16	26	42	1695	191.6	6.2
Q8K310	Matr3	29	22	22	44	846	94.6	6.25

P18760	Cfl1	71	11	13	79	166	18.5	8.09
Q9Z0P4	Palm	50	17	18	52	383	41.6	4.84
P84091	Ap2m1	37	17	17	97	435	49.6	9.54
Q7TQD2	Tppp	56	13	13	60	218	23.6	9.42
Q9WV34	Mpp2	42	21	21	39	552	61.5	6.44
P70296	Pebp1	69	8	8	46	187	20.8	5.4
Q01065	Pde1b	35	16	17	45	535	61.2	5.72
Q6R891	Ppp1r9b	28	19	19	39	817	89.5	4.92
Q9D6R2	Idh3a	42	17	17	61	366	39.6	6.73
Q9D051	Pdhb	35	11	11	49	359	38.9	6.87
O35526	Stx1a	58	13	15	57	288	33	5.24
P48453	Ppp3cb	27	8	14	66	525	59.1	5.91
Q8R0S2	Iqsec1	23	18	21	41	961	107.9	7.14
Q91WC3	Acsl6	34	21	21	42	697	78	7.34
Q4ACU6	Shank3	18	22	22	36	1730	185.3	8.88
Q3TXS7	Psmd1	30	19	19	29	953	105.7	5.39
P61922	Abat	42	16	16	46	500	56.4	8.09
Q9JLM8	Dclk1	28	15	16	34	756	84.1	8.87
Q61548	Snap91	18	12	13	41	901	91.8	4.88
P80316	Cct5	37	19	19	39	541	59.6	6.02
Q923T9	Camk2g	34	8	15	64	529	59.6	7.58
Q3UM45	Ppp1r7	46	16	16	35	361	41.3	4.92
O88342	Wdr1	35	17	17	46	606	66.4	6.6
Q9CZC8	Scrn1	31	12	12	44	414	46.3	4.79
Q91YT0	Ndufv1	38	14	14	45	464	50.8	8.21
P30275	Ckmt1	48	15	15	75	418	47	8.16
Q5SQX6	Cyfip2	18	11	22	40	1253	145.6	7.05
Q8VEK3	Hnrnpu	28	18	18	44	800	87.9	6.24
Q8BMJ2	Lars	19	20	20	31	1178	134.1	7.05
P09405	Ncl	27	22	22	44	707	76.7	4.75
Q9CPY7	Lap3	33	15	15	36	519	56.1	7.72
Q80Z38	Shank2	23	23	23	33	1476	158.9	7.18
P02088	Hbb-b1	69	4	10	180	147	15.8	7.65
Q8C1B7	Septin11	38	5	14	44	431	49.7	6.68
P06837	Gap43	79	18	18	67	227	23.6	4.73
Q60597	Ogdh	20	21	21	39	1023	116.4	6.83
Q9D6M3	Slc25a22	52	11	13	36	323	34.6	9.09
P47738	Aldh2	35	14	15	32	519	56.5	7.62
P17742	Ppia	63	13	13	110	164	18	7.9
Q9R1T4	Septin6	40	8	15	40	434	49.6	6.43
Q80TL4	Phf24	46	14	14	39	400	45.2	5.77
Q6PB66	Lrpprc	17	22	22	38	1392	156.5	6.83
P06745	Gpi	29	18	18	78	558	62.7	8.13
Q9R1Q8	Tagln3	72	11	13	37	199	22.5	7.33

Q9JME5	Ap3b2	22	20	20	39	1082	119.1	5.63
Q00PI9	Hnrnpul2	23	15	15	31	745	84.9	4.89
P47708	Rph3a	24	13	14	39	681	75.4	8.27
P62821	Rab1A	55	7	12	44	205	22.7	6.21
O35098	Dpysl4	32	12	14	54	572	61.9	6.98
O54774	Ap3d1	18	18	18	35	1199	135	7.37
Q8VD37	Sgip1	31	16	16	27	806	86	7.87
O88569	Hnrnpa2b1	51	14	15	61	353	37.4	8.95
P27546	Map4	25	21	21	33	1125	117.4	4.98
Q8VD04	Gripap1	27	18	18	28	806	92.7	5.25
Q9R0P9	Uchl1	71	14	14	49	223	24.8	5.24
Q9CYT6	Cap2	41	16	16	39	476	52.8	6.43
Q9Z2Y3	Homer1	48	16	16	39	366	41.4	5.53
P09103	P4hb	40	19	19	52	509	57	4.88
Q7TMB8	Cyfip1	17	9	20	35	1253	145.1	6.9
P26040	Ezr	34	16	21	43	586	69.4	6.1
Q9CZU6	Cs	32	14	14	72	464	51.7	8.57
Q8K0T0	Rtn1	22	11	11	28	780	83.5	4.58
Q9D0F9	Pgm1	32	16	16	43	562	61.4	6.57
Q8BMF4	Dlat	27	16	16	48	642	67.9	8.57
Q9WU78	Pdcd6ip	23	17	17	36	869	96	6.52
P49025	Cit	11	23	23	35	2055	235.2	6.54
P26638	Sars	36	14	14	42	512	58.4	6.3
P60202	Plp1	36	11	11	152	277	30.1	8.35
Q61598	Gdi2	48	15	17	36	445	50.5	6.25
P08752	Gnai2	43	7	11	34	355	40.5	5.45
P47754	Capza2	63	9	11	33	286	32.9	5.85
Q9CS84	Nrxn1	19	17	20	30	1514	166.1	6.09
Q99PL5	Rrbp1	33	28	28	36	1605	172.8	9.33
Q60930	Vdac2	63	14	14	71	295	31.7	7.49
P07759	Serpina3k	32	6	11	101	418	46.9	5.16
Q8R081	Hnrnpl	38	15	15	40	586	63.9	8.1
Q6ZPE2	Sbf1	13	17	17	24	1867	208.6	7.12
P39447	Tjp1	13	18	18	28	1745	194.6	6.64
O55042	Snca	86	9	11	74	140	14.5	4.77
Q9JIS5	Sv2a	20	14	15	61	742	82.6	5.57
Q8K0E8	Fgb	44	18	18	34	481	54.7	7.08
P99029	Prdx5	52	10	10	31	210	21.9	8.85
P61979	Hnrnpk	38	15	15	49	463	50.9	5.54
P62761	Vsnl1	62	10	12	63	191	22.1	5.15
Q9WUB3	Pygm	28	16	22	35	842	97.2	7.11
Q8CC35	Synpo	26	15	15	32	929	99.5	9.42
P70398	Usp9x	9	20	20	34	2559	290.5	5.87

P48036	Anxa5	52	16	17	41	319	35.7	4.96
P28665	Mug1	15	19	19	33	1476	165.2	6.42
P10630	Eif4a2	35	6	13	37	407	46.4	5.48
P23818	Gria1	19	11	16	35	907	101.5	7.69
Q8BVE3	Atp6v1h	32	13	13	31	483	55.8	6.61
P39054	Dnm2	21	5	16	60	870	98.1	7.43
P50518	Atp6v1e1	39	12	12	49	226	26.1	8.43
Q60902	Eps15l1	28	20	20	31	907	99.2	5.02
P31324	Prkar2b	52	16	18	32	416	46.1	4.98
P67778	Phb	54	13	13	47	272	29.8	5.76
Q8BMS1	Hadha	25	16	16	27	763	82.6	9.14
Q7TMY8	Huwe1	7	22	22	27	4377	482.3	5.22
Q91V61	Sfxn3	51	12	12	39	321	35.4	9.51
Q8CA95	Pde10a	25	19	19	28	790	89.4	6.6
P42669	Pura	45	9	9	22	321	34.9	6.44
P26043	Rdx	35	16	22	37	583	68.5	6.2
O89053	Coro1a	34	16	17	45	461	51	6.48
O08749	Dld	34	12	12	45	509	54.2	7.9
Q9D0K2	Oxct1	37	13	13	43	520	56	8.53
Q922J3	Clip1	15	15	19	37	1391	155.7	5.24
Q61696	Hspa1a	26	4	14	57	641	70	5.72
P12382	Pfkl	32	13	17	27	780	85.3	7.17
Q61361	Bcan	20	14	14	42	883	95.8	4.93
Q9CZ44	Nsfl1c	54	17	17	32	370	40.7	5.15
Q810U4	Nrcam	18	17	17	30	1256	138.4	5.91
Q61792	Lasp1	52	15	15	45	263	30	7.05
P97300	Nptn	29	12	12	55	397	44.3	7.74
Q3THK7	Gmps	27	16	16	30	693	76.7	6.73
P63330	Ppp2ca	42	2	12	39	309	35.6	5.54
Q7TQI3	Otub1	41	8	8	29	271	31.3	4.94
P14211	Calr	43	17	17	52	416	48	4.49
O08709	Prdx6	56	10	10	42	224	24.9	6.01
Q8VDQ8	Sirt2	37	13	13	39	389	43.2	5.35
P02468	Lamc1	14	22	22	32	1607	177.2	5.21
P70333	Hnrnph2	35	4	12	39	449	49.2	6.3
Q91WQ3	Yars	36	18	18	38	528	59.1	7.01
Q61838	Pzp	18	19	20	37	1495	165.7	6.68
P28740	Kif2a	29	19	19	32	705	79.7	6.73
Q8CGK3	Lonp1	19	15	15	33	949	105.8	6.57
P10852	Slc3a2	26	14	14	44	526	58.3	5.91
Q8CGY8	Ogt	20	16	16	27	1046	116.9	6.7
P61205	Arf3	52	6	7	43	181	20.6	7.43
Q922S4	Pde2a	23	19	19	41	939	105.6	5.41
P08228	Sod1	57	10	10	60	154	15.9	6.51

P62715	Ppp2cb	42	2	12	35	309	35.6	5.43
Q63810	Ppp3r1	69	11	11	31	170	19.3	4.81
P80318	Cct3	42	19	19	30	545	60.6	6.7
P70704	Atp8a1	12	14	14	25	1164	131.3	6.84
O35737	Hnrnph1	33	2	11	39	449	49.2	6.3
Q9Z1G3	Atp6v1c1	41	16	16	46	382	43.9	7.46
Q3U1J4	Ddb1	22	24	24	38	1140	126.8	5.26
P33175	Kif5a	23	10	20	31	1027	116.9	5.94
P56564	Slc1a3	16	7	7	35	543	59.6	8.4
D3YZU1	Shank1	10	17	17	26	2167	226.2	8.34
Q2PFD7	Psd3	12	17	17	35	1037	114.7	6.24
O08917	Flot1	43	15	15	29	428	47.5	7.15
Q5DTL9	Slc4a10	16	12	17	29	1118	125.7	6.51
Q3U0V1	Khsrp	27	14	16	34	748	76.7	7.33
O35551	Rabep1	22	17	17	29	862	99.5	5.01
Q61490	Alcam	23	12	12	23	583	65.1	6.15
Q9JII6	Akr1a1	45	14	14	45	325	36.6	7.39
P80317	Cct6a	39	18	18	28	531	58	7.08
Q68FL4	Ahcyl2	26	7	15	37	613	66.9	7.36
Q6NZL0	Soga3	21	17	19	30	945	103.4	6.14
P63005	Pafah1b1	39	15	15	32	410	46.6	7.37
P47757	Capzb	48	13	13	31	277	31.3	5.74
Q91V41	Rab14	67	12	12	32	215	23.9	6.21
P46097	Syt2	32	6	12	34	422	47.2	7.99
P35486	Pdha1	37	16	16	58	390	43.2	8.19
Q8BVI4	Qdpr	38	7	7	28	241	25.6	7.81
P48758	Cbr1	51	10	10	27	277	30.6	8.31
Q8R071	Itpka	30	11	11	23	459	50.9	7.72
Q99PU5	Acsbg1	25	13	13	27	721	80.4	5.94
Q8BJ42	Dlgap2	18	14	16	25	1059	119	6.81
Q60875	Arhgef2	23	17	17	31	985	111.9	7.25
Q9JKY5	Hip1r	19	19	20	27	1068	119.4	6.52
Q9QZQ1	Afdn	13	22	23	32	1820	206.4	6.32
P46471	Psmc2	36	14	14	33	433	48.6	5.95
Q9EQ20	Aldh6a1	35	14	14	32	535	57.9	8.07
Q9Z1S5	Septin3	42	12	12	40	350	40	6.81
Q91ZA3	Pcca	26	15	15	29	724	79.9	7.25
Q8R570	Snap47	39	13	13	22	413	46.5	5.76
P16054	Prkce	24	15	16	31	737	83.5	7.03
O70133	Dhx9	15	15	15	24	1380	149.4	6.83
Q9CVB6	Arpc2	40	14	14	39	300	34.3	7.36
Q64487	Ptprd	11	12	15	23	1912	214.3	6.55
Q05793	Hspg2	7	21	21	30	3707	398	6.32
P97315	Csrp1	60	10	10	29	193	20.6	8.57

Q91V12	Acot7	36	12	12	45	381	42.5	8.68
Q922D8	Mthfd1	22	17	18	29	935	101.1	7.14
Q920I9	Wdr7	14	18	18	26	1489	163.3	6.9
P70175	Dlg3	22	18	19	28	849	93.4	6.79
A2AN08	Ubr4	4	18	18	26	5180	571.9	6.06
Q78PY7	Snd1	23	17	17	29	910	102	7.43
Q811D0	Dlg1	18	12	17	36	905	100.1	5.8
Q04690	Nf1	8	22	22	28	2841	319.4	7.39
Q9D1A2	Cndp2	29	10	10	27	475	52.7	5.66
P42859	Htt	7	15	15	20	3119	344.5	6.29
O70161	Pip5k1c	23	13	14	26	661	72.4	5.59
P26041	Msn	36	15	20	35	577	67.7	6.6
Q80TE7	Lrrc7	15	16	17	24	1490	166.8	6.61
Q9Z218	Dpp6	22	14	14	31	804	91.2	6.68
Q80TV8	Clasp1	12	15	18	30	1535	169.1	9.03
Q64514	Tpp2	21	20	20	28	1262	139.8	6.58
A2AQ25	Skt	11	18	18	25	1946	212.9	7.77
Q9QXY6	Ehd3	29	10	13	29	535	60.8	6.46
P63011	Rab3a	44	6	8	59	220	25	5.03
P10649	Gstm1	36	9	10	51	218	26	7.94
P61164	Actr1a	40	7	13	32	376	42.6	6.64
Q9CWS0	Ddah1	52	12	13	29	285	31.4	5.97
Q6ZPJ3	Ube2o	12	12	12	25	1288	140.7	5.06
Q8K212	Pacs1	18	15	15	30	961	104.8	7.74
P04370	Mbp	41	10	10	128	250	27.2	9.58
Q8BGD9	Eif4b	33	19	19	30	611	68.8	5.67
P70168	Kpnb1	20	15	15	20	876	97.1	4.78
Q3UTJ2	Sorbs2	20	20	20	30	1180	132.3	8.53
Q99JY9	Actr3	47	12	16	34	418	47.3	5.88
Q9CR68	Uqcrfs1	34	11	11	43	274	29.3	8.7
P08003	Pdia4	27	15	15	30	638	71.9	5.31
P00920	Ca2	63	11	11	36	260	29	7.01
O35136	Ncam2	19	17	17	38	837	93.1	6.07
Q921M7	Fam49b	39	10	11	40	324	36.8	6.06
Q60625	Icam5	19	16	16	30	917	96.9	6.32
Q3URD3	Slmap	19	16	16	28	845	96.9	5.33
Q9JLB0	Mpp6	33	17	17	31	553	62.6	6.4
P20444	Prkca	20	7	14	41	672	76.8	7.14
Q922B2	Dars	32	13	13	24	501	57.1	6.49
P13707	Gpd1	43	15	15	29	349	37.5	7.17
Q80TB8	Vat1l	43	15	15	27	417	45.8	5.06
Q91YQ5	Rpn1	28	17	17	33	608	68.5	6.46
Q9D394	Rufy3	31	11	12	28	469	53	5.49
Q9D8Y0	Efhd2	42	11	11	34	240	26.8	5.06

P50247	Ahcy	31	12	12	27	432	47.7	6.54
Q812A2	Srgap3	15	14	15	22	1099	124.3	6.67
P10605	Ctsb	36	10	10	30	339	37.3	5.91
O88643	Pak1	29	8	11	30	545	60.7	5.74
P60843	Eif4a1	38	7	14	29	406	46.1	5.48
Q02248	Ctnnb1	17	11	11	33	781	85.4	5.86
Q91ZZ3	Sncb	51	3	6	51	133	14	4.37
Q99JR1	Sfxn1	41	9	9	25	322	35.6	9.23
P11276	Fn1	11	20	20	27	2477	272.4	5.59
O55106	Strn	23	13	14	21	780	85.9	5.27
Q9EQH3	Vps35	17	13	13	39	796	91.7	5.44
Q8BKZ9	Pdhx	26	11	11	33	501	54	7.75
P28661	Septin4	29	12	15	33	478	54.9	5.87
Q9Z0X1	Aifm1	24	13	13	25	612	66.7	9.17
P51863	Atp6v0d1	31	13	13	36	351	40.3	5
Q8C0T5	Sipa1l1	12	18	19	25	1782	196.9	8.13
Q99LX0	Park7	47	7	7	51	189	20	6.77
P12367	Prkar2a	37	10	12	33	401	45.4	4.89
P84309	Adcy5	14	16	16	24	1262	139	7.06
Q0KL02	Trio	6	15	18	24	3102	347.6	6.35
Q9QWY8	Asap1	14	15	15	21	1147	127.3	7.64
Q61885	Mog	34	11	11	58	246	28.3	7.96
Q3UHD9	Agap2	16	20	20	26	1186	124.4	9.89
Q62059	Vcan	4	12	12	24	3357	366.6	4.64
Q5SVL6	Rap1gap2	21	11	11	23	712	78.2	6.43
P54227	Stmn1	62	12	13	56	149	17.3	5.97
Q8BG39	Sv2b	15	10	12	39	683	77.4	5.57
Q9JLV5	Cul3	19	16	16	26	768	88.9	8.46
Q9Z0R4	Itsn1	12	17	20	27	1714	194.2	7.91
P16627	Hspa1l	22	2	12	49	641	70.6	6.24
Q9QUR6	Prep	20	12	12	28	710	80.7	5.73
P62823	Rab3c	37	6	8	33	227	25.9	5.24
P97351	Rps3a	56	15	15	38	264	29.9	9.73
O88485	Dync1i1	27	14	14	22	628	70.7	5.12
Q5SYD0	Myo1d	16	18	18	32	1006	116	9.41
F6ZDS4	Tpr	10	23	23	26	2431	273.8	5.03
Q60634	Flot2	31	12	12	29	428	47	5.2
Q91VR5	Ddx1	28	16	16	23	740	82.4	7.21
B2RSH2	Gnai1	35	7	11	30	354	40.3	5.97
Q9ESJ4	Nckipsd	25	14	14	22	714	78.5	6.05
Q9WVK8	Cyp46a1	31	14	14	26	500	56.8	8.82
Q9JKK7	Tmod2	44	12	12	27	351	39.5	5.35
Q9JHK4	Rabggta	33	16	16	26	567	64.9	5.8
Q8BUV3	Gphn	20	14	14	22	769	83.2	5.6

Q9Z1Z0	Uso1	17	14	14	28	959	106.9	4.93
Q91WD5	Ndufs2	27	9	9	31	463	52.6	6.99
Q8C0M9	Asrgl1	29	9	9	28	326	33.9	7.65
Q9DC69	Ndufa9	33	11	11	22	377	42.5	9.74
Q80U63	Mfn2	19	12	12	27	757	86.1	6.77
Q8R5H6	Wasf1	19	7	9	21	559	61.5	6.37
Q91ZJ5	Ugp2	36	15	15	21	508	56.9	7.61
Q99PT1	Arhgdia	49	8	8	27	204	23.4	5.2
Q99PV0	Prpf8	8	19	19	27	2335	273.4	8.84
Q99L47	St13	21	8	8	28	371	41.6	5.26
P51150	Rab7a	61	11	11	27	207	23.5	6.7
O88844	Idh1	36	16	16	26	414	46.6	7.17
E9PV24	Fga	19	16	16	33	789	87.4	6.11
Q8CHH9	Septin8	38	10	14	41	429	49.8	6
B9EKR1	Ptprz1	6	13	13	39	2312	254.2	4.88
Q9WTX5	Skp1	47	7	7	22	163	18.7	4.54
Q8CHG7	Rapgef2	13	14	14	23	1496	166.3	6.61
P49443	Ppm1a	32	11	11	20	382	42.4	5.36
P35235	Ptpn11	33	18	18	25	593	68	7.3
Q8BLQ9	Cadm2	24	9	10	34	435	47.5	5.41
Q8BIJ6	Iars2	18	14	14	23	1012	112.7	6.81
Q9CWJ9	Atic	27	13	13	28	592	64.2	6.76
P55012	Slc12a2	15	16	16	25	1205	131	7.33
Q5DU25	Iqsec2	10	11	14	27	1478	161.7	8.56
Q62419	Sh3gl1	33	7	11	41	368	41.5	5.72
P19536	Cox5b	59	9	9	45	128	13.8	8.38
Q80TK0	Kiaa1107	14	15	15	18	1239	134	6.61
Q8BKC5	Ipo5	11	11	11	22	1097	123.5	4.93
Q9QYB5	Add3	18	10	10	31	706	78.7	5.95
Q8CJ19	Mical3	10	18	18	22	1993	223.6	5.47
O54983	Crym	42	12	12	31	313	33.5	5.67
Q99KK7	Dpp3	24	15	15	29	738	82.8	5.38
Q9D8N0	Eef1g	23	12	12	27	437	50	6.74
Q5SWU9	Acaca	9	19	19	24	2345	265.1	6.39
Q07076	Anxa7	24	10	10	26	463	49.9	6.18
Q9JJK2	Lancl2	28	8	8	16	450	50.7	7.28
Q9QXL2	Kif21a	11	15	15	18	1672	186.4	6.27
Q8BGT8	Phyhipl	26	8	9	28	375	42.3	6.35
P63044	Vamp2	35	4	5	28	116	12.7	8.13
Q9JLJ2	Aldh9a1	28	12	12	21	494	53.5	6.98
Q6P9K9	Nrxn3	12	11	16	25	1571	173.3	6.14
P61161	Actr2	26	9	9	38	394	44.7	6.74
Q9DCT2	Ndufs3	41	11	11	30	263	30.1	7.17
O88487	Dync1i2	19	11	11	25	612	68.4	5.29

Q8R5C5	Actr1b	36	6	12	25	376	42.3	6.4
Q6NZJ6	Eif4g1	11	12	15	24	1600	176	5.4
Q6NXK7	Dpp10	17	13	13	23	797	90.8	6.48
P01942	Hba	51	9	9	187	142	15.1	8.22
Q80YN3	Bcas1	28	16	16	28	633	67.3	6.21
A2ALS5	Rap1gap	30	14	14	18	663	73.4	5.87
Q93092	Taldo1	29	10	10	28	337	37.4	7.03
P32921	Wars	29	13	13	23	481	54.3	6.89
Q7TT50	Cdc42bpb	10	16	17	25	1713	194.6	6.46
O54991	Cntnap1	9	12	12	27	1385	156.2	7.06
Q3UVX5	Grm5	13	16	17	21	1203	131.8	7.84
Q99MN9	Pccb	22	10	10	21	541	58.4	7.66
Q8JZQ2	Afg3l2	25	17	17	22	802	89.5	8.6
P23953	Ces1c	16	9	9	26	554	61	5.06
Q62418	Dbnl	31	12	12	27	436	48.7	4.92
Q8BGZ1	Hpcal4	58	7	9	27	191	22.2	4.89
Q921G7	Etfdh	28	15	15	24	616	68	7.58
Q9CRB9	Chchd3	42	11	11	31	227	26.3	8.37
P62137	Ppp1ca	36	3	10	25	330	37.5	6.33
Q9CR62	Slc25a11	32	8	8	37	314	34.1	9.94
Q99LC5	Etfa	33	8	8	19	333	35	8.38
Q99K85	Psat1	35	12	12	28	370	40.4	8.03
Q91W50	Csde1	18	16	16	29	798	88.7	6.37
O55023	Impa1	38	12	12	31	277	30.4	5.19
P60335	Pcbp1	36	5	9	27	356	37.5	7.09
P26645	Marcks	45	9	9	19	309	29.6	4.34
Q9D0M3	Cyc1	30	7	7	19	325	35.3	9.16
P55066	Ncan	14	18	19	27	1268	137.1	5.72
A2CG49	Kalrn	6	14	17	22	2964	336.8	6.07
Q99LF4	Rtcb	28	12	12	20	505	55.2	7.23
P12023	App	16	11	12	26	770	86.7	4.79
Q9JKR6	Hyou1	16	13	13	23	999	111.1	5.19
Q9EQF6	Dpysl5	33	14	14	28	564	61.5	7.09
Q8CJ40	Crocc	9	17	17	19	2009	226.8	5.55
Q8BP47	NARS1	20	11	11	21	559	64.2	5.86
P53810	Pitpna	38	9	10	24	271	31.9	6.37
P26231	Ctnna1	18	7	12	21	906	100	6.23
P28271	Aco1	20	13	13	21	889	98.1	7.5
Q99JF8	Psip1	32	14	15	21	528	59.7	9.13
P50544	Acadvl	27	12	12	18	656	70.8	8.75
Q9QVP9	Ptk2b	17	16	16	25	1009	115.7	6.35
Q8BYR5	Cadps2	11	8	14	22	1297	147.7	6.14
Q8K010	Oplah	14	13	13	18	1288	137.5	6.28
Q3UH60	Dip2b	10	12	12	19	1574	171	8.09

P58871	Tnks1bp1	12	13	13	17	1720	181.7	4.88
Q61656	Ddx5	23	11	15	23	614	69.2	8.92
Q3UPL0	Sec31a	13	14	14	18	1230	133.5	6.76
Q99LC3	Ndufa10	26	11	11	27	355	40.6	7.78
E9PUL5	Prrt2	40	9	9	25	346	35.9	4.63
O35129	Phb2	47	14	14	29	299	33.3	9.83
Q60676	Ppp5c	26	12	12	28	499	56.8	6.2
Q9Z2W9	Gria3	17	9	12	22	888	100.5	8.38
Q9WTQ5	Akap12	10	12	12	19	1684	180.6	4.44
P62908	Rps3	50	11	11	27	243	26.7	9.66
O88712	Ctbp1	23	4	9	28	441	47.7	6.77
Q8R3V5	Sh3glb2	30	11	12	24	400	44.5	5.82
Q8R464	Cadm4	31	9	9	21	388	42.7	6.3
Q60829	Ppp1r1b	64	8	8	27	194	21.8	4.65
P46664	Adss2	29	10	11	22	456	50	6.38
Q5SRX1	Tom1l2	19	9	9	19	507	55.6	4.82
Q9EPJ9	Arfgap1	37	12	12	28	414	45.3	5.57
Q99MK8	Grk2	23	13	13	21	689	79.6	7.28
Q80U49	Cep170b	11	14	16	22	1574	170.7	6.87
P62141	Ppp1cb	31	2	8	28	327	37.2	6.19
Q68FF6	Git1	22	13	13	21	770	85.2	6.93
Q6P1F6	Ppp2r2a	34	6	12	25	447	51.7	6.2
O70443	Gnaz	34	10	11	34	355	40.8	7.61
Q9JIF7	Copb1	15	11	11	20	953	107	6
Q80TZ3	Dnajc6	15	11	13	22	938	102.2	7.23
Q9D819	Ppa1	49	11	11	23	289	32.6	5.6
P97797	Sirpa	28	11	11	29	513	56.4	8.28
P19783	Cox4i1	45	8	8	39	169	19.5	9.23
Q8VDJ3	Hdlbp	15	19	19	28	1268	141.7	6.87
O55234	Psmb5	41	12	12	22	264	28.5	7.02
Q8VEM8	Slc25a3	30	11	11	42	357	39.6	9.26
Q8CIE6	Copa	12	15	15	20	1224	138.3	7.65
Q3V3R1	Mthfd1l	15	13	14	23	977	105.7	7.02
Q9Z2I0	Letm1	18	13	13	26	738	82.9	6.52
Q9R0P5	Dstn	55	9	10	30	165	18.5	7.97
O08529	Capn2	18	10	11	25	700	79.8	4.96
Q61171	Prdx2	35	7	7	44	198	21.8	5.41
Q99PJ0	Ntm	33	9	9	33	344	38	7.81
Q61081	Cdc37	36	14	14	32	379	44.6	5.34
Q9DBF1	Aldh7a1	24	12	12	24	539	58.8	7.47
P21550	Eno3	19	1	6	136	434	47	7.18
Q61035	Hars	24	12	12	27	509	57.4	6
P63087	Ppp1cc	31	1	8	23	323	37	6.54
Q9EPK7	Xpo7	11	8	8	15	1087	123.7	6.38

O88685	Psmc3	34	13	13	24	442	49.5	5.19
Q8BH95	Echs1	33	8	8	23	290	31.5	8.48
P08032	Spta1	7	12	13	16	2415	279.7	5.03
Q9EQZ6	Rapgef4	17	16	16	22	1011	115.4	6.92
B1AWN6	Scn2a	7	8	12	19	2006	227.8	5.67
Q9CWK8	Snx2	22	8	10	17	519	58.4	5.12
Q9Z1Z2	Strap	39	10	10	23	350	38.4	5.12
P27612	Plaa	18	12	12	18	794	87.2	6.14
Q99K51	Pls3	25	9	11	20	630	70.7	5.62
A2RT62	Fbxl16	26	9	9	17	479	51.8	6.6
P62962	Pfn1	66	7	7	24	140	14.9	8.28
Q9D0R2	Tars1	23	15	15	20	722	83.3	7.36
Q99N28	Cadm3	41	13	13	29	396	42.9	5.8
Q8BSS9	Ppfia2	15	14	16	19	1257	143.1	6.11
Q99KY4	Gak	14	13	15	21	1305	143.6	5.73
Q64525	Hist2h2bb	53	4	8	51	126	13.9	10.32
Q03137	Epha4	15	11	11	23	986	109.7	6.51
P57746	Atp6v1d	42	10	10	27	247	28.4	9.45
Q2M3X8	Phactr1	28	15	15	26	580	66.2	6.96
O35295	Purb	44	6	6	12	324	33.9	5.43
Q00897	Serpina1d	27	3	9	24	413	46	5.44
Q99NE5	Rims1	12	12	12	16	1463	163.1	9.42
P35700	Prdx1	60	11	12	37	199	22.2	8.12
Q06890	Clu	25	11	11	19	448	51.6	5.67
Q9WUM4	Coro1c	34	12	14	21	474	53.1	7.08
P54071	Idh2	32	11	11	19	452	50.9	8.69
Q9R0Y5	Ak1	48	8	8	30	194	21.5	5.81
Q02357	Ank1	11	16	16	18	1862	204.1	6.55
Q9Z204	Hnrnpc	35	11	12	28	313	34.4	5.05
P62702	Rps4x	37	9	9	22	263	29.6	10.15
Q62433	Ndrg1	26	8	8	28	394	43	6.1
P48318	Gad1	22	12	12	18	593	66.6	7.17
Q9CQD1	Rab5a	53	5	8	20	215	23.6	8.15
P68181	Prkacb	31	4	10	26	351	40.7	8.56
Q8K183	Pdxk	32	8	8	21	312	35	6.29
Q8BK64	Ahsa1	32	9	9	19	338	38.1	5.53
Q91X72	Hpx	33	14	14	30	460	51.3	7.8
Q9DC07	Nebl	47	13	13	18	270	31.1	8.31
P62897	Cycs	56	7	7	15	105	11.6	9.58
O88543	Cops3	27	10	10	19	423	47.8	6.65
Q6ZPQ6	Pitpnm2	10	11	12	19	1335	147.9	7.93
Q05816	Fabp5	73	9	9	18	135	15.1	6.54

Q8VDM4	Psmd2	14	12	12	26	908	100.1	5.17
Q80XI3	Eif4g3	11	13	16	24	1579	174.8	5.53
Q4KUS2	Unc13a	9	15	15	20	1712	193.7	5.25
Q64010	Crk	41	9	9	19	304	33.8	5.55
Q00898	Serpina1e	27	4	10	29	413	45.9	5.73
Q9WTX6	Cul1	16	10	10	17	776	89.6	8
Q7TMK9	Syncrip	17	9	9	21	623	69.6	8.59
Q8R0S4	Cacnb4	20	6	9	17	519	57.9	9.28
P62983	Rps27a	46	7	7	148	156	17.9	9.64
Q6IRU5	Cltb	38	10	10	33	229	25.2	4.63
Q61543	Glg1	13	14	14	18	1175	133.6	6.84
P62881	Gnb5	32	11	11	23	395	43.5	6.46
Q9EPW0	Inpp4a	16	14	14	20	939	105.5	7.05
Q8BLK3	Lsamp	37	11	11	38	341	38.1	6.65
P53994	Rab2a	39	4	7	15	212	23.5	6.54
Q8VHJ5	Mark1	20	9	13	14	795	88.3	9.39
Q9DCD0	Pgd	22	8	8	20	483	53.2	7.23
Q60668	Hnrnpd	22	7	7	28	355	38.3	7.81
P13020	Gsn	16	13	13	28	780	85.9	6.18
Q91VM5	Rbmxl1	39	5	18	33	388	42.1	9.99
Q9CPV4	Glod4	37	9	9	20	298	33.3	5.47
P29341	Pabpc1	20	13	13	23	636	70.6	9.5
P84096	Rhog	50	8	9	21	191	21.3	8.12
P07758	Serpina1a	25	3	9	24	413	46	5.72
O09061	Psmb1	47	8	8	18	240	26.4	7.81
P37040	Por	18	11	11	20	678	77	5.53
Q78ZA7	Nap1l4	26	7	8	17	375	42.7	4.67
Q64436	Atp4a	7	2	7	48	1033	113.9	5.88
O54988	Slk	13	14	14	21	1233	141.4	5.14
Q9WVE8	Pacsin2	29	12	14	25	486	55.8	5.2
P61226	Rap2b	52	5	8	17	183	20.5	4.81
E9PVA8	Gcn1	7	14	14	18	2671	292.8	7.36
Q91VI7	Rnh1	36	12	12	19	456	49.8	4.78
P70441	Slc9a3r1	40	11	11	26	355	38.6	5.9
Q01097	Grin2b	10	13	13	18	1482	165.9	6.87
Q99LD4	Gps1	19	7	7	18	471	53.4	6.84
P23506	Pcmt1	41	10	10	17	227	24.6	7.65
P21107	Tpm3	33	7	11	24	285	33	4.72
P22892	Ap1g1	17	12	12	19	822	91.3	6.8
Q9R1P0	Psma4	35	9	9	20	261	29.5	7.72
Q8JZW4	Cpne5	18	6	10	20	593	65.6	5.77
Q5H8C4	Vps13a	6	14	14	18	3166	359.2	6.19
P43277	H1-3	25	1	8	31	221	22.1	11.03

Q99JB2	Stoml2	36	10	10	16	353	38.4	8.87
F8VPU2	Farp1	12	12	12	21	1048	118.8	7.88
Q8K0S0	Phyhip	27	8	9	24	330	37.5	7.01
Q9CXS4	Cenpv	45	8	8	18	252	27.5	9.79
O54829	Rgs7	20	5	8	19	469	54.8	8.13
P56695	Wfs1	12	9	9	17	890	100.5	7.97
P07356	Anxa2	35	12	12	22	339	38.7	7.69
Q00623	Apoa1	44	13	13	27	264	30.6	5.73
Q64331	Myo6	13	14	14	19	1265	146.3	8.85
Q9D8W5	Psmd12	27	12	12	19	456	52.9	7.06
Q91YM2	Arhgap35	11	14	14	16	1499	170.3	6.61
P22599	Serpina1b	22	2	8	25	413	45.9	5.54
P05132	Prkaca	30	5	11	25	351	40.5	8.79
P48774	Gstm5	31	8	10	26	224	26.6	7.21
Q9JJV2	Pfn2	55	6	6	31	140	15	6.99
O88447	Klc1	20	8	10	22	541	61.4	5.68
Q80UG5	Septin9	19	12	12	25	583	65.5	8.9
P28474	Adh5	25	9	9	20	374	39.5	7.25
E9Q6P5	Ttc7b	18	14	14	18	843	94.1	6.9
Q80UG2	Plxna4	8	11	15	25	1893	212.4	6.83
P70206	Plxna1	7	10	13	19	1894	211	6.9
P20918	Plg	18	12	12	17	812	90.7	6.6
O35685	Nudc	33	13	13	23	332	38.3	5.26
Q62421	Sh3gl3	38	11	11	16	347	38.9	5.12
Q9CQN1	Trap1	16	9	9	26	706	80.2	6.68
P62806	H4c1	58	7	7	39	103	11.4	11.36
Q99KK2	Cmas	22	10	10	18	432	48	8.1
O08547	Sec22b	34	6	6	17	215	24.7	8.51
Q9CY58	Serbp1	39	12	12	19	407	44.7	8.54
Q99LI8	Hgs	17	12	12	27	775	86	6.16
Q61102	Abcb7	14	9	9	14	752	82.5	9.32
O54865	Gucy1b1	17	10	10	22	620	70.6	5.31
Q6PCP5	Mff	42	7	7	11	291	32.9	6.83
O70589	Cask	16	13	13	18	926	105	6.43
O70172	Pip4k2a	26	6	10	21	405	46.1	6.99
Q6PGN3	Dclk2	18	10	11	16	756	82.9	8.44
Q9WTT4	Atp6v1g2	44	6	6	21	118	13.6	10.26
Q9CZD3	Gars1	17	12	12	19	729	81.8	6.65
Q9DCW4	Etfb	40	8	8	20	255	27.6	8.1
Q8CI71	Vps50	13	11	11	15	964	111.1	6.07
Q9D6J6	Ndufv2	47	10	10	26	248	27.3	7.4
Q9Z0S1	Bpnt1	35	9	9	19	308	33.2	5.82

Q9ER72	Cars	17	12	12	16	831	94.8	6.76
Q62417	Sorbs1	12	13	13	19	1290	143	8.25
Q811P8	Arhgap32	8	13	14	20	2089	229.6	6.86
Q8CAA7	Pgm2l1	21	12	13	21	621	70.2	6.49
Q3ULJ0	Gpd1l	33	11	11	22	351	38.2	6.77
Q9JIF0	Prmt1	30	8	10	19	371	42.4	5.43
Q9D1G1	Rab1b	45	3	8	26	201	22.2	5.73
P46638	Rab11b	42	8	8	28	218	24.5	5.94
P55937	Golga3	9	10	10	14	1487	167.1	5.4
Q3USB7	Plcl1	16	15	15	17	1096	122.6	5.64
P23116	Eif3a	11	17	17	24	1344	161.8	6.77
Q80U28	Madd	9	13	13	26	1577	175.1	6.04
Q8VCM7	Fgg	23	9	9	23	436	49.4	5.86
Q8BW75	Maob	22	9	9	16	520	58.5	8.29
Q91VM9	Ppa2	30	10	10	20	330	38.1	6.98
E9Q7G0	Numa1	8	16	16	17	2094	235.5	5.87
Q9EPU0	Upf1	13	12	12	16	1124	123.9	6.61
Q9JIA1	Lgi1	12	7	7	20	557	63.6	8.02
Q8R0A7	Kiaa0513	21	10	10	21	407	46.3	5.02
P35564	Canx	19	11	11	25	591	67.2	4.64
P30416	Fkbp4	25	13	13	21	458	51.5	5.72
Q9D2U9	Hist3h2ba	45	3	7	49	126	14	10.37
Q9WVJ2	Psmd13	28	10	10	20	376	42.8	5.71
Q9CR95	Necap1	28	6	6	20	275	29.6	6.38
Q58A65	Spag9	10	9	13	20	1321	146.1	5.15
Q8BHN3	Ganab	14	12	12	21	944	106.8	6.06
Q9DCX2	Atp5pd	52	7	7	31	161	18.7	5.69
P23927	Cryab	47	7	7	16	175	20.1	7.33
Q8C0C7	Farsa	18	8	8	18	508	57.6	8.28
Q8K3J1	Ndufs8	29	7	7	43	212	24	6.21
Q60575	Kif1b	8	2	12	19	1816	204	5.62
O88990	Actn3	10	1	9	22	900	103	5.45
P70663	Sparcl1	25	11	11	15	650	72.2	4.6
P43274	H1-4	22	3	7	27	219	22	11.11
Q9WV02	Rbmx	35	3	16	28	391	42.3	10.05
Q5SSL4	Abr	18	13	14	19	859	97.6	6.58
P24529	Th	28	9	9	13	498	56	6.15
P06801	Me1	27	11	11	18	572	63.9	7.44
B9EJA2	Cttnbp2	8	14	14	21	1648	178.7	8.19
Q5U3K5	Rabl6	16	8	8	12	725	79.8	5.53
Q60931	Vdac3	43	10	10	28	283	30.7	8.79

Q8VIJ6	Sfpq	16	11	12	25	699	75.4	9.44
P63168	Dynll1	33	2	2	20	89	10.4	7.4
P62334	Psmc6	25	9	9	17	389	44.1	7.49
Q8VDP4	Ccar2	17	12	12	15	922	102.9	5.25
Q9DCL9	Paics	24	9	9	17	425	47	7.23
O35927	Ctnnd2	13	14	14	16	1247	134.9	7.65
P15864	H1-2	24	1	7	29	212	21.3	11
Q148V7	Relch	11	12	12	18	1216	134.5	5.34
Q8VCT3	Rnpep	16	9	9	17	650	72.4	5.35
Q5EBJ4	Ermn	44	9	9	17	281	32.1	4.59
P19157	Gstp1	20	2	4	41	210	23.6	7.87
Q9R1V6	Adam22	16	11	11	21	904	99.7	7.83
P12658	Calb1	31	8	9	27	261	30	4.83
P24527	Lta4h	20	11	11	23	611	69	6.42
Q99JY0	Hadhb	23	9	9	25	475	51.4	9.38
Q9CQW1	Ykt6	44	8	8	20	198	22.3	6.35
Q8K3H0	Appl1	21	10	10	16	707	79.3	5.41
Q6PAJ1	Bcr	12	11	12	15	1270	143	6.92
P14685	Psmd3	18	9	9	23	530	60.7	8.44
Q9QYJ0	Dnaja2	32	11	11	17	412	45.7	6.48
Q9R1T2	Sae1	30	10	10	18	350	38.6	5.36
Q61481	Pde1a	24	10	11	20	545	62.3	5.91
P20917	Mag	20	10	10	21	627	69.3	5.1
P63040	Cplx1	56	4	6	21	134	15.1	4.97
O88343	Slc4a4	11	11	11	17	1079	121.4	6.84
Q64105	Spr	42	8	8	16	261	27.9	5.74
Q08331	Calb2	44	10	11	19	271	31.4	5.02
Q61990	Pcbp2	25	2	8	23	362	38.2	6.79
P09528	Fth1	48	8	8	22	182	21.1	5.88
Q64133	Maoa	20	11	11	22	526	59.6	7.81
Q3TDD9	Ppp1r21	19	11	11	15	780	88.3	6.9
Q9Z2D6	Mecp2	29	11	11	18	484	52.3	9.96
Q9JHQ5	Lztfl1	32	8	8	14	299	34.8	5.17
Q9D0M5	Dynll2	38	3	3	34	89	10.3	7.37
Q921F2	Tardbp	26	8	8	18	414	44.5	6.7
Q9Z140	Cpne6	14	8	9	23	557	61.7	5.59
Q8BMK4	Ckap4	21	12	12	14	575	63.7	5.64
Q3TXX4	Slc17a7	15	9	9	22	560	61.6	7.34
Q3B7Z2	Osbp	16	9	10	16	805	88.7	7.2
Q8C522	Endod1	23	8	8	16	501	55.2	6.16
Q9CR16	Ppid	26	8	9	20	370	40.7	7.43
Q9CPW0	Cntnap2	10	11	11	22	1332	148.1	6.77
P21279	Gnaq	25	8	9	27	359	42.1	5.68
P99026	Psmb4	40	6	6	10	264	29.1	5.64

P14869	Rplp0	32	6	6	11	317	34.2	6.25
O88544	Cops4	35	12	12	18	406	46.3	5.83
Q9Z2U0	Psma7	38	9	9	22	248	27.8	8.46
Q64516	Gk	18	10	10	20	559	61.2	5.87
Q99JI4	Psmd6	31	13	13	19	389	45.5	5.52
Q61137	Astn1	13	13	13	18	1302	144.8	5.21
Q8CGU1	Calcoco1	19	11	11	15	691	77.2	4.82
P61089	Ube2n	71	8	8	21	152	17.1	6.57
Q8BJY1	Psmd5	30	10	10	13	504	55.9	5.21
Q8VCW8	Acsf2	14	8	8	17	615	67.9	8.18
Q8BG05	Hnrnpa3	23	9	9	26	379	39.6	9.01
Q8C1A5	Thop1	20	12	12	21	687	78	6.06
P09671	Sod2	30	7	7	18	222	24.6	8.62
Q9Z2H2	Rgs6	19	6	9	19	472	54.5	7.42
P35278	Rab5c	52	5	8	17	216	23.4	8.41
Q91WJ8	Fubp1	18	9	11	20	651	68.5	7.93
Q641P0	Actr3b	24	6	10	18	418	47.5	6.02
Q91WS0	Cisd1	44	4	4	25	108	12.1	9.06
Q9DBR7	Ppp1r12a	16	14	15	17	1029	114.9	5.49
Q60605	Myl6	44	7	7	18	151	16.9	4.65
Q8BIZ1	Anks1b	8	8	9	23	1259	139	6.29
P45591	Cfl2	41	4	6	27	166	18.7	7.88
Q91WP6	Serpina3n	21	6	9	16	418	46.7	5.82
Q8R1Q8	Dync1li1	27	10	10	15	523	56.6	6.42
A2A5R2	Arfgef2	8	12	14	16	1792	202.1	6.55
Q922R8	Pdia6	19	7	7	14	440	48.1	5.14
P18572	Bsg	25	9	9	24	389	42.4	5.85
P52503	Ndufs6	58	5	5	14	116	13	8.65
Q9D2G2	Dlst	21	8	8	31	454	49	8.95
P84075	Hpca	52	6	9	29	193	22.4	4.97
P62192	Psmc1	25	9	9	21	440	49.2	6.21
P62827	Ran	28	7	7	14	216	24.4	7.49
Q80XI4	Pip4k2b	21	4	8	18	416	47.3	7.33
Q8QZS1	Hibch	25	8	8	15	385	43	8.06
O88448	Klc2	20	8	11	17	599	66.6	7.21
P35762	Cd81	21	3	3	25	236	25.8	5.83
Q505F5	Lrrc47	17	8	8	16	581	63.6	8.1
Q8BU30	Iars	11	13	13	18	1262	144.2	6.55
O88910	Mpp3	22	9	9	15	568	64.4	6.34
P70404	Idh3g	32	9	9	17	393	42.8	9.01
A2AWA9	Rabgap1	13	13	13	21	1064	120.7	5.25
Q6GQT9	Nomo1	10	12	12	16	1214	133.3	6.09
P01872	Ighm	19	8	8	15	454	49.9	7.01
Q9CZ42	Naxd	30	8	8	14	343	36.7	7.77

Q8VE47	Uba5	21	6	6	15	403	44.8	4.96
P63037	Dnaja1	30	10	10	19	397	44.8	7.08
Q7TMF3	Ndufa12	59	6	6	19	145	17.1	9.36
P61082	Ube2m	47	10	10	19	183	20.9	7.69
Q8BHZ0	Fam49a	25	5	6	19	323	37.3	6.01
Q9DB20	Atp5po	55	10	10	24	213	23.3	9.99
Q80YA9	Cnksr2	11	9	9	15	1032	117.3	6.79
Q9D2V7	Coro7	18	12	12	15	922	100.7	5.77
Q8BGH2	Samm50	29	10	10	14	469	51.8	6.8
Q6PB44	Ptpn23	9	13	13	17	1692	185.1	6.8
O35593	Psmd14	33	6	6	12	310	34.6	6.52
O88532	Zfr	11	9	9	11	1074	116.8	9.04
Q9R1V7	Adam23	10	7	7	14	829	91.5	7.68
Q8JZS0	Lin7a	42	4	8	16	233	26	8.72
P11627	L1cam	9	7	7	12	1260	140.9	5.97
P28656	Nap1l1	18	4	5	14	391	45.3	4.46
Q9CXW3	Cacybp	39	7	7	12	229	26.5	7.87
Q9D0L7	Armc10	32	6	6	10	306	33.3	7.99
Q62167	Ddx3x	18	2	11	18	662	73.1	7.18
Q6IR34	Gpsm1	20	9	9	13	673	74.3	6.23
								10.5
P12970	Rpl7a	28	8	8	14	266	30	6
Q9D0L8	Rnmt	27	9	9	13	465	53.3	6.48
Q9Z1R2	Bag6	11	10	10	15	1154	121	5.71
Q9Z1L5	Cacna2d3	11	9	9	15	1091	122.7	5.73
O35857	Timm44	23	9	9	22	452	51.1	8.13
Q0GNC1	Inf2	12	12	12	14	1273	138.5	5.21
Q61771	Kif3b	16	10	13	17	747	85.2	7.69
P01864		17	3	4	14	335	36.6	8.22
Q8R366	Igsf8	18	9	9	18	611	65	7.99
Q8K021	Scamp1	34	8	8	17	338	38	7.71
A2ALU4	Shroom2	8	9	9	15	1481	164.6	6.7
Q9DBC7	Prkar1a	25	7	10	17	381	43.2	5.35
P97445	Cacna1a	6	12	12	13	2368	267.5	8.85
Q9QUP5	Hapln1	25	7	7	17	356	40.5	7.8
Q8R5M8	Cadm1	20	6	7	15	456	49.8	5.03
Q8CGK7	Gnal	26	9	9	17	381	44.3	6.65
Q80Z24	Negr1	28	8	8	16	348	37.9	6.52
P84086	Cplx2	52	4	6	13	134	15.4	5.08
Q8CC88	Vwa8	7	13	13	16	1905	213.3	6.6
Q8JZN5	Acad9	16	9	9	14	625	68.7	7.46
Q9Z2W0	Dnpep	26	10	10	17	473	52.2	7.25
Q61330	Cntn2	14	12	12	12	1040	113.1	7.62
P51830	Adcy9	11	13	13	15	1353	150.9	7.21

Q9WV60	Gsk3b	27	7	10	14	420	46.7	8.78
Q9WVK4	Ehd1	19	6	9	17	534	60.6	6.83
Q99KE1	Me2	24	10	11	19	589	65.8	7.61
P62196	Psmc5	21	8	8	13	406	45.6	7.55
Q80TS3	Adgrl3	7	10	10	12	1537	171	6.71
Q61584	Fxr1	17	7	8	14	677	76.2	6.98
Q8BW96	Camk1d	27	6	9	12	385	42.9	7.17
Q9JHI5	Ivd	22	10	10	18	424	46.3	8.29
Q9ES28	Arhgef7	11	11	11	14	862	97	6.8
Q9QUM9	Psma6	43	9	9	16	246	27.4	6.76
P41216	Acsl1	17	10	11	16	699	77.9	7.15
Q05512	Mark2	15	6	10	15	776	86.3	9.67
Q62095	Ddx3y	18	2	11	15	658	73.4	7.53
P35436	Grin2a	8	10	10	14	1464	165.3	7.01
Q9D1E6	Tbcb	36	9	9	24	244	27.4	5.24
P63085	Mapk1	30	8	10	24	358	41.2	6.98
Q80UW2	Fbxo2	31	7	7	15	297	33.7	4.28
P23242	Gja1	21	7	7	19	382	43	8.76
Q9CPP6	Ndufa5	58	5	5	12	116	13.4	8.1
Q8VHL1	Setd7	25	7	7	15	366	40.5	4.65
P08556	Nras	44	1	7	16	189	21.2	5.17
Q9QZB7	Actr10	29	10	10	16	417	46.2	7.61
P27601	Gna13	27	9	10	22	377	44	8.21
Q8BJI1	Slc6a17	13	7	7	16	727	81	6.23
Q61937	Npm1	29	8	8	15	292	32.5	4.77
Q6A065	Cep170	7	10	12	17	1588	174.9	7.17
Q64433	Hspe1	56	6	6	26	102	11	8.35
P31650	Slc6a11	11	7	7	13	627	69.9	6.98
Q8CBE3	Wdr37	22	11	11	19	496	55	7.23
Q8K394	Plcl2	10	10	10	16	1128	125.7	6.92
A2ASQ1	Agrn	7	11	11	12	1950	207.4	6.32
Q8BR92	Palm2	23	8	9	20	376	42.1	5.15
Q5RJI5	Brsk1	17	12	12	15	778	85.1	9.32
Q9CQQ7	Atp5pb	31	9	9	31	256	28.9	9.06
Q925I1	Atad3	17	10	10	17	591	66.7	9.29
Q80WJ7	Mtdh	18	9	9	19	579	63.8	9.33
Q99MR8	Mccc1	13	8	8	14	717	79.3	7.83
Q9D1K2	Atp6v1f	48	7	7	23	119	13.4	5.82
P14231	Atp1b2	33	9	9	21	290	33.3	8.31
Q6PGF7	Exoc8	17	10	10	12	716	81	5.4
Q9QZE5	Copg1	15	9	10	17	874	97.5	5.35
Q6PDM2	Srsf1	36	9	9	22	248	27.7	10.3<br>6
Q9CZW4	Acsl3	19	9	10	12	720	80.4	8.54

Q5PR73	Diras2	48	6	9	21	199	22.5	8.76
Q9ERK4	Cse1l	13	11	11	15	971	110.4	5.77
Q3UHB1	Nt5dc3	19	11	11	18	546	63.1	8.56
P35802	Gpm6a	15	5	5	26	278	31.1	5.27
Q63844	Mapk3	27	7	9	20	380	43	6.61
Q9CQF9	Pcyox1	24	7	7	12	505	56.5	6.92
Q6P4T2	Snrnp200	6	12	12	13	2136	244.4	6.06
P30999	Ctnnd1	13	10	10	14	938	104.9	6.87
P60487	Pdxp	30	7	7	13	292	31.5	5.74
Q80U23	Snph	27	9	9	14	495	53.7	5.9
Q8BMA6	Srp68	20	12	12	16	625	70.5	8.57
Q9WUK2	Eif4h	37	10	10	35	248	27.3	7.23
P62754	Rps6	27	7	7	21	249	28.7	10.84
O35465	Fkbp8	21	7	7	25	402	43.5	5.16
Q99020	Hnrnpab	22	4	5	21	285	30.8	7.91
Q91Z53	Grhpr	24	6	6	19	328	35.3	7.65
Q3UHD6	Snx27	17	9	9	17	539	61	6.4
Q9D172	Gatd3a	32	7	7	20	266	28.1	8.78
Q61001	Lama5	3	7	7	11	3718	403.8	6.73
Q80ZJ1	Rap2a	44	4	7	15	183	20.6	4.82
P14148	Rpl7	29	8	8	14	270	31.4	10.89
Q9EPL8	Ipo7	10	9	9	13	1038	119.4	4.82
P40630	Tfam	31	9	9	19	243	28	9.69
O09111	Ndufb11	50	6	6	21	151	17.4	5.22
Q9WTP7	Ak3	43	10	10	18	227	25.4	8.84
Q9ESW4	Agk	17	7	7	15	421	46.9	8.4
P57722	Pcbp3	22	1	7	21	371	39.3	7.52
O55029	Copb2	14	12	12	15	905	102.4	5.3
B0V2N1	Ptprs	7	7	9	11	1907	211.8	7.23
P02089	Hbb-b2	44	1	7	36	147	15.9	8.05
Q80VP0	Tecpr1	10	10	10	16	1166	130.2	6.48
Q8BX70	Vps13c	4	13	13	18	3748	419.8	6.81
Q9CQ60	Pgls	36	6	6	10	257	27.2	5.85
Q9Z2X1	Hnrnpf	20	5	7	14	415	45.7	5.49
P32037	Slc2a3	14	6	6	14	493	53.4	4.98
Q7TME0	Plppr4	17	11	11	16	766	83.2	8.84
O55091	Impact	33	9	9	18	318	36.3	5.05
Q03734	Serpina3m	22	2	8	14	418	47	6.1
Q9WV55	Vapa	39	7	8	23	249	27.8	8.4
Q9Z0P5	Twf2	25	6	7	17	349	39.4	6.8
P38060	Hmgcl	32	9	9	12	325	34.2	8.41

Q9CQC6	Bzw1	18	7	7	11	419	48	5.92
Q9D5V5	Cul5	15	12	12	17	780	90.9	7.81
P62264	Rps14	40	8	8	19	151	16.3	10.0<br>5
O35639	Anxa3	24	7	7	16	323	36.4	5.76
P34152	Ptk2	9	9	9	14	1052	119.2	6.62
Q99L13	Hibadh	20	6	6	12	335	35.4	8.13
Q9CZ30	Ola1	26	9	9	18	396	44.7	7.81
Q8CBW3	Abi1	22	9	11	19	481	52.3	7.64
Q9JLN9	Mtor	5	14	14	18	2549	288.6	7.17
Q61029	Tmpo	28	4	9	11	452	50.3	9.45
O35874	Slc1a4	18	8	8	15	532	56	5.87
P49722	Psma2	42	9	9	13	234	25.9	7.43
Q9Z1Q9	Vars	12	13	13	16	1263	140.1	7.77
Q80X50	Ubap2l	9	8	8	14	1107	116.7	7.11
Q9CXZ1	Ndufs4	31	5	5	21	175	19.8	9.99
P68040	Rack1	22	7	7	13	317	35.1	7.69
Q80UJ7	Rab3gap1	15	12	12	15	981	110.1	5.73
Q8BRF7	Scfd1	20	9	9	13	639	72.3	6.38
Q8BH66	Atl1	18	8	9	17	558	63.3	6.49
Q922Q1	Marc2	23	8	8	18	338	38.2	8.68
Q9CZT8	Rab3b	30	5	6	17	219	24.7	5.11
Q3THE2	Myl12b	40	2	6	17	172	19.8	4.84
P59999	Arpc4	43	7	7	21	168	19.7	8.43
Q9EPL2	Clstn1	11	11	11	16	979	108.8	4.92
Q80TQ2	Cyld	15	12	12	14	952	106.5	5.63
Q9Z2I8	Suclg2	28	8	8	11	433	46.8	7.02
Q9WV69	Dmtn	26	9	10	13	405	45.4	8.41
Q5DQR4	Stxbp5l	9	8	8	13	1185	131.8	6.86
O70435	Psma3	21	7	7	21	255	28.4	5.44
Q60771	Cldn11	15	4	4	43	207	22.1	7.91
P62746	Rhob	39	6	7	16	196	22.1	5.24
P29699	Ahsg	27	7	7	12	345	37.3	6.51
Q9QY76	Vapb	34	7	8	21	243	26.9	7.78
Q9QUI0	Rhoa	42	5	6	16	193	21.8	6.1
P70670	Naca	3	4	4	10	2187	220.4	9.35
Q9CYH2	Prxl2a	32	8	8	14	218	24.4	9.17
P28028	Braf	12	5	8	11	804	88.7	7.65
Q61151	Ppp2r5e	16	7	8	13	467	54.7	6.95
P04925	Prnp	20	6	6	17	254	28	9.33
Q02819	Nucb1	22	9	9	14	459	53.4	5.07
P49312	Hnrnpa1	23	5	6	14	320	34.2	9.23
Q9CRB6	Tppp3	38	7	7	15	176	19	9.11
Q5DTY9	Kctd16	24	11	11	16	427	48.9	8.19

Q8R326	Pspc1	13	5	6	12	523	58.7	6.67
Q921M3	Sf3b3	10	11	11	15	1217	135.5	5.26
Q8CDG3	Vcpip1	9	9	9	14	1220	134.4	7.17
Q6PDG5	Smarcc2	11	12	12	15	1213	132.5	5.59
P46061	Rangap1	20	10	10	14	589	63.5	4.68
O35633	Slc32a1	20	8	8	12	525	57.3	6.64
P50580	Pa2g4	20	8	8	17	394	43.7	6.86
Q9Z268	Rasal1	16	12	12	19	799	89.3	6.37
Q8BVQ5	Ppme1	25	8	8	10	386	42.2	5.97
Q8VHI6	Wasf3	15	4	6	10	501	55.2	6.37
P63325	Rps10	39	7	8	18	165	18.9	10.15
Q64523	Hist2h2ac	58	3	6	21	129	14	10.9
O88741	Gdap1	16	7	7	12	358	41.3	8.37
Q6A0A9	FAM120A	13	10	11	12	1112	121.6	8.92
Q8CDN6	Txnl1	29	6	6	12	289	32.2	4.96
Q9Z0U1	Tjp2	9	11	11	14	1167	131.2	6.79
P84084	Arf5	32	3	5	16	180	20.5	6.79
Q6NS52	Dgkb	15	10	10	12	802	90.2	7.93
P61027	Rab10	33	7	7	16	200	22.5	8.38
Q8R3S6	Exoc1	9	8	8	11	894	101.8	6.52
P58771	Tpm1	29	4	10	18	284	32.7	4.74
Q6P8X1	Snx6	27	10	11	15	406	46.6	6.16
P51174	Acadl	20	7	7	14	430	47.9	8.31
Q9D7G0	Prps1	27	7	7	11	318	34.8	6.98
A2RSJ4	Uhrf1bp1l	8	11	11	13	1457	161.8	6.61
Q14BI2	Grm2	13	11	11	20	872	95.8	8.06
Q8CGF6	Wdr47	12	11	11	14	920	102.2	5.97
Q62442	Vamp1	34	3	4	12	118	12.9	6.65
P99027	Rplp2	68	4	4	11	115	11.6	4.54
D3YXK2	Safb	10	6	9	16	937	105	5.35
O08759	Ube3a	11	8	8	15	870	99.8	5.08
Q9JM52	Mink1	9	9	11	17	1308	147.2	7.44
P48320	Gad2	14	5	5	12	585	65.2	6.9
O70325	Gpx4	32	6	6	16	197	22.2	8.46
Q61411	Hras	42	2	7	15	189	21.3	5.31
A2AQ07	Tubb1	12	1	6	115	451	50.4	5.07
Q9D832	Dnajb4	38	10	11	13	337	37.8	8.59
Q8C854	Myef2	15	9	9	18	591	63.3	8.87
O35864	Cops5	29	6	6	12	334	37.5	6.54
Q920E5	Fdps	25	8	8	12	353	40.6	5.66
Q8VD75	Hip1	9	8	9	11	1029	115.1	5.43

P16332	Mmut	10	6	6	12	748	82.8	6.89
Q9D7N9	Apmap	21	8	8	12	415	46.4	6.32
Q8BMF3	Me3	18	10	11	20	604	67.1	7.83
Q9WUA2	Farsb	15	8	8	14	589	65.7	7.12
P62071	Rras2	25	4	5	14	204	23.4	6.01
Q9JMA1	Usp14	16	8	8	13	493	56	5.24
Q9R1P4	Psma1	23	6	6	18	263	29.5	6.46
Q3UTQ8	Cdkl5	11	11	11	12	938	105.4	9.58
P58404	Strn4	14	8	9	11	760	81.6	5.38
P63137	Gabrb2	19	6	8	11	512	59.2	9.31
Q9CXJ4	Abcb8	10	6	6	12	717	78	9.07
P35279	Rab6a	43	6	11	18	208	23.6	5.54
P60122	Ruvbl1	18	6	6	10	456	50.2	6.42
Q9R0Q6	Arpc1a	19	8	8	15	370	41.6	8.18
Q8BMG7	Rab3gap2	8	10	10	13	1366	152.4	6.23
Q9DCJ5	Ndufa8	36	5	5	15	172	20	8.46
Q3THS6	Mat2a	17	8	8	17	395	43.7	6.48
P18242	Ctsd	20	7	7	16	410	44.9	7.15
Q9D0E1	Hnrnpm	17	11	11	14	729	77.6	8.63
P62281	Rps11	33	6	6	16	158	18.4	10.3
Q8R550	Sh3kbp1	17	9	9	14	709	78.1	7.55
Q80X90	Flnb	5	9	10	11	2602	277.7	5.71
P51859	Hdgf	31	6	7	14	237	26.3	4.83
Q80XN0	Bdh1	37	9	9	13	343	38.3	9.01
P32883	Kras	38	3	6	11	189	21.6	6.77
Q9QYS2	Grm3	10	9	9	21	879	99.1	7.75
Q8C5H8	Nadk2	22	7	7	10	452	50.8	8.07
O08795	Prkcsh	14	9	9	15	521	58.8	4.46
Q9CTY5	Micu3	18	9	9	13	523	59.8	7.15
P28650	Adss1	28	9	10	14	457	50.2	8.43
Q8K4G5	Ablim1	13	11	12	13	861	96.7	8.63
Q01405	Sec23a	15	8	9	11	765	86.1	7.08
Q6ZQ08	Cnot1	5	10	10	10	2375	266.6	7.11
Q60780	Gas7	18	7	7	11	421	48.1	7.83
Q99L45	Eif2s2	30	8	8	15	331	38.1	5.8
P23198	Cbx3	40	6	7	17	183	20.8	5.22
Q99MN1	Kars1	16	10	10	16	595	67.8	5.94
Q62277	Syp	14	5	5	41	314	34	4.94
P45376	Akr1b1	26	7	7	10	316	35.7	7.18
Q9WU79	Prodh	19	9	9	11	599	68	8.24
O89112	Lancl1	24	9	9	19	399	45.3	7.77
P56959	Fus	13	6	6	11	518	52.6	9.36
Q9ESN6	Trim2	16	7	10	14	744	81.4	6.96
Q8VCD6	Reep2	34	7	7	13	254	28.4	9.41

P42567	Eps15	9	6	6	12	897	98.4	4.6
Q62318	Trim28	12	10	10	22	834	88.8	5.77
Q9Z1P6	Ndufa7	48	5	5	11	113	12.6	10.17
Q8C437	Pex5l	16	8	8	11	567	63.1	5.33
Q91Z67	Srgap2	11	10	11	14	1071	120.7	6.64
Q3ULD5	Mccc2	14	5	5	10	563	61.3	8
Q3UJU9	Rmdn3	21	9	9	14	470	52	5.21
Q80UM3	Naa15	13	11	11	15	865	100.9	7.62
P31648	Slc6a1	12	4	4	17	599	67	7.96
Q9Z1W9	Stk39	20	7	8	13	556	60.3	6.29
Q05BC3	Eml1	12	10	10	16	814	89.6	7.02
P21460	Cst3	51	6	6	17	140	15.5	9
Q3UUG6	Tbc1d24	13	7	7	12	561	63.2	7.24
Q9R0P3	Esd	27	5	5	13	282	31.3	7.12
Q00612	G6pdx	18	9	9	14	515	59.2	6.49
Q9R0N7	Syt7	14	7	7	13	403	45.4	9.28
P56391	Cox6b1	71	7	7	28	86	10.1	8.72
P31938	Map2k1	21	7	7	14	393	43.4	6.7
O08585	Clta	24	8	8	22	235	25.6	4.58
Q8CCB4	Vps53	12	9	9	14	832	94.4	6.61
P28741	Kif3a	15	9	10	14	701	80.1	6.54
P47934	Crat	14	8	8	13	626	70.8	8.44
Q9Z2U1	Psma5	22	5	5	16	241	26.4	4.79
Q8CHT1	Ngef	13	11	11	18	710	82.1	5.99
Q99MI1	Erc1	10	9	12	16	1120	128.3	5.87
Q9JKB1	Uchl3	27	6	6	16	230	26.1	5.05
P31230	Aimp1	28	6	6	9	310	34	8.35
P70195	Psmb7	21	5	5	19	277	29.9	7.99
P32067	Ssb	19	9	9	16	415	47.7	9.77
Q9JMH6	Txnrd1	12	6	6	11	613	67	7.44
P28867	Prkcd	14	9	10	18	674	77.5	7.39
Q99JI6	Rap1b	54	3	8	16	184	20.8	5.78
Q9Z1J3	Nfs1	24	9	9	11	459	50.5	8.16
Q8BG32	Psmd11	23	10	10	12	422	47.4	6.48
P59281	Arhgap39	9	8	8	10	1107	125.1	7.97
Q8BNU0	Armc6	19	7	7	11	468	50.7	6
Q99KN9	Clint1	17	9	9	13	631	68.5	6.25
Q3TES0	Iqsec3	9	7	9	12	1195	129	6.19
P08226	Apoe	29	9	9	18	311	35.8	5.68
Q9Z130	Hnrnpdl	27	7	8	24	301	33.5	7.31
P29758	Oat	23	7	7	12	439	48.3	6.62
P27659	Rpl3	16	6	6	16	403	46.1	10.21

Q9ESN9	Mapk8ip3	6	4	8	15	1337	147.5	5.45
O08756	Hsd17b10	26	4	4	10	261	27.4	8.41
Q8VHH5	Agap3	9	6	8	16	910	97.9	7.75
Q9D0S9	Hint2	45	5	5	7	163	17.3	9.82
Q6ZQ18	Efr3b	14	8	8	12	817	92.3	6.54
P21614	Gc	20	8	8	13	476	53.6	5.5
P35803	Gpm6b	20	6	6	20	328	36.2	6.14
Q9QZD9	Eif3i	18	6	6	13	325	36.4	5.64
Q91WK5	Gcsh	18	2	2	10	170	18.6	4.75
P24549	Aldh1a1	21	8	9	14	501	54.4	7.8
Q6PHN9	Rab35	38	6	7	15	201	23	8.29
Q8CCT4	Tceal5	36	8	8	25	200	22	6.2
Q71M36	Cspg5	15	6	6	11	566	60.4	4.54
P21836	Ache	14	6	6	14	614	68.1	6.33
Q640R3	Hepacam	25	7	7	15	418	46.3	9.42
O35382	Exoc4	11	8	8	12	975	110.5	6.49
Q62443	Nptx1	23	9	9	11	432	47.1	6.44
Q8C878	Uba3	20	7	7	10	462	51.7	5.45
Q7M6Y3	Picalm	16	7	8	17	660	71.5	7.91
P15532	Nme1	46	4	7	16	152	17.2	7.37
Q99M71	Epdr1	21	4	4	15	224	25.5	7.58
Q6DFV3	Arhgap21	5	9	9	12	1944	215.6	7.64
P63094	Gnas	27	10	10	17	394	45.6	5.96
Q8VBY2	Camkk1	22	9	9	14	505	55.8	5.99
Q61335	Bcap31	18	4	4	12	245	27.9	8.7
P12787	Cox5a	28	6	6	15	146	16.1	6.54
Q3TVA9	Ccdc136	5	4	4	7	1136	131.8	4.84
Q6Y685	Tacc1	11	6	7	12	774	83.9	5.03
Q9ERI6	Rdh14	20	6	6	14	334	36.3	8.18
Q3UMR5	Mcu	23	9	9	14	350	39.7	8.56
O35343	Kpna4	10	3	4	11	521	57.9	4.94
Q00493	Cpe	20	8	8	10	476	53.2	5.19
Q91VN4	Chchd6	22	5	5	13	273	29.8	8.41
Q9CZY3	Ube2v1	51	3	6	21	147	16.3	7.96
Q9D154	Serpinb1a	22	7	7	13	379	42.5	6.21
P01869	Ighg1	17	5	5	9	393	43.4	6.44
Q80TR1	Adgrl1	5	5	5	11	1466	161.6	6.48
P60904	Dnajc5	21	7	7	10	198	22.1	5.07
Q921J2	Rheb	27	6	6	12	184	20.4	5.92
P47199	Cryz	26	7	7	10	331	35.2	8.07
Q9D1G5	Lrrc57	44	10	10	14	239	26.7	8.35
Q99K48	Nono	19	8	10	19	473	54.5	8.95
Q921Q7	Rin1	14	7	7	9	763	83	8.72
Q99LR1	Abhd12	17	6	6	11	398	45.2	8.72

Q8BP92	Rcn2	29	7	7	14	320	37.2	4.42
Q07417	Acads	17	5	5	9	412	44.9	8.47
Q9Z1F9	Uba2	13	8	8	16	638	70.5	5.24
Q6GQS1	Slc25a23	16	5	6	12	467	52.5	7.59
Q924M7	Mpi	10	3	3	15	423	46.5	5.95
P97450	Atp5pf	50	6	6	11	108	12.5	9.36
P01837	Igkc	49	5	5	10	107	11.9	5.9
Q6PFD5	Dlgap3	8	8	9	15	977	105.8	8.76
P61294	Rab6b	47	4	9	17	208	23.4	5.53
Q3UHB8	Ccdc177	11	6	6	9	706	79.8	10.8
Q8CBY8	Dctn4	21	6	6	9	467	53	7.72
Q8BUK6	Hook3	15	9	9	17	718	83.2	5.19
Q9CQA3	Sdhb	27	10	10	17	282	31.8	8.68
Q9WV80	Snx1	14	5	7	11	522	58.9	5.22
P60766	Cdc42	36	5	6	17	191	21.2	6.55
P20060	Hexb	12	7	7	10	536	61.1	8.12
O08677	Kng1	9	6	6	16	661	73.1	6.54
Q501J6	Ddx17	16	5	9	13	650	72.4	8.59
Q9CX86	Hnrnpa0	22	6	6	14	305	30.5	9.31
Q9Z110	Aldh18a1	12	8	8	11	795	87.2	7.55
Q9JI46	Nudt3	40	5	6	18	168	19	6.34
Q3TEA8	Hp1bp3	11	7	7	11	554	60.8	9.7
								11.5
P47963	Rpl13	26	7	7	16	211	24.3	5
Q2NL51	Gsk3a	14	3	6	10	490	51.6	8.81
O35344	Kpna3	16	4	5	11	521	57.7	4.94
Q9JL04	Fmn2	5	7	7	8	1578	167.3	5.48
O55126	Nipsnap2	17	6	6	16	281	32.9	9.26
Q6ZPU9	Kifbp	13	4	4	11	617	71	5.48
P01878		17	4	4	6	344	36.9	5.06
Q9Z0N1	Eif2s3x	20	8	8	14	472	51	8.4
Q9DBP5	Cmpk1	30	6	6	14	196	22.2	5.83
A2APV2	Fmnl2	8	8	9	11	1086	123	7.53
P35282	Rab21	27	5	5	11	222	24.1	7.94
P48193	Epb41	10	4	7	16	858	95.9	5.6
Q6ZQK5	Acap2	16	9	9	10	770	87.2	6.68
Q91W86	Vps11	9	9	9	15	941	107.7	7.01
Q5U458	Dnajc11	17	11	11	15	559	63.2	8.32
								10.7
P27661	H2afx	48	3	6	23	143	15.1	4
Q5F2E8	Taok1	9	9	10	17	1001	116	7.55
Q9CX34	Sugt1	28	9	9	13	336	38.1	5.45
P61750	Arf4	28	2	4	10	180	20.4	7.14
P63054	Pcp4	61	5	5	12	62	6.8	6.71

Q99PL6	Ubxn6	22	8	8	15	442	49.8	8.54
P84099	Rpl19	21	5	5	12	196	23.5	11.47
Q8BLR2	Cpne4	14	6	7	11	557	62.4	6.33
P97384	Anxa11	16	6	6	10	503	54	7.66
Q9D6Z1	Nop56	10	6	6	9	580	64.4	9.14
Q68FH0	Pkp4	8	9	9	13	1190	131.5	8.94
Q8R127	Sccpdh	17	6	6	10	429	47.1	8.6
P26883	Fkbp1a	25	2	2	46	108	11.9	8.16
P26350	Ptma	23	5	5	8	111	12.2	3.79
Q01768	Nme2	38	3	6	15	152	17.4	7.5
P54775	Psmc4	20	6	6	11	418	47.4	5.21
Q9WUM3	Coro1b	20	9	9	14	484	53.9	5.78
Q9CZX8	Rps19	37	6	6	12	145	16.1	10.4
P36916	Gnl1	17	9	9	15	607	68.7	5.68
P63158	Hmgb1	22	7	7	12	215	24.9	5.74
Q925N0	Sfxn5	23	7	7	13	342	37.3	9.44
Q6A4J8	Usp7	7	9	9	15	1103	128.4	5.5
P62812	Gabra1	15	6	7	13	455	51.7	9.31
Q69ZW3	Ehbp1	7	7	7	8	1231	139	5.38
Q9CU62	Smc1a	9	11	11	12	1233	143.1	7.64
P54830	Ptpn5	20	9	9	14	541	60.8	5.2
P35293	Rab18	40	7	7	10	206	23	5.36
Q7TPM6	Fsd1	19	6	6	10	496	55.5	6.67
P62830	Rpl23	49	6	6	8	140	14.9	10.51
Q3UEB3	Puf60	17	7	7	11	564	60.2	5.29
Q9QXK3	Copg2	11	6	7	8	871	97.6	5.8
Q8C1B1	Camsap2	6	8	9	11	1461	164.2	6.86
Q3UU96	Cdc42bpa	6	8	9	10	1719	195.4	6.51
Q9R0X4	Acot9	26	10	10	14	439	50.5	8.59
Q8BNY6	Ncs1	39	7	7	15	190	21.9	4.83
Q8BH04	Pck2	12	5	5	8	640	70.5	7.28
Q91VE0	Slc27a4	14	8	8	11	643	72.3	8.59
Q8R3B1	Plcd1	14	8	8	9	756	85.8	6.21
Q6NVE8	Wdr44	9	8	8	11	915	101.5	5.24
Q9JKC6	Cend1	35	5	5	17	149	15	8.97
P52760	Rida	35	4	4	11	135	14.2	8.68
P70232	Chl1	9	7	7	11	1209	135	5.57
Q8BLE7	Slc17a6	10	5	5	9	582	64.5	6.68
Q8K2K6	Agfg1	19	8	9	14	561	58	8.63
Q9Z2W8	Gria4	7	2	6	14	902	100.8	8.07
Q62425	Ndufa4	56	6	6	23	82	9.3	9.52
Q9DCS9	Ndufb10	35	6	6	15	176	21	8.03

Q08460	Kcnma1	7	6	6	9	1209	134.3	7.15
Q91VC3	Eif4a3	21	7	9	11	411	46.8	6.73
Q9CRC9	Gnpda2	24	5	6	8	276	31.1	6.9
A6X935	Itih4	10	7	7	10	942	104.6	6.4
Q99KQ4	Nampt	19	7	7	8	491	55.4	7.15
Q9CZR8	Tsfm	30	7	7	10	324	35.3	7.06
Q61655	Ddx19a	11	6	6	13	478	53.9	6.67
Q9D8E6	Rpl4	19	8	8	13	419	47.1	11
P47911	Rpl6	22	8	8	20	296	33.5	10.7
Q6WVG3	Kctd12	18	5	6	10	327	35.9	5.81
Q8BWS5	Gprin3	16	10	10	11	763	80.4	7.02
P62717	Rpl18a	34	6	6	16	176	20.7	10.71
Q03141	Mark3	12	4	8	9	753	84.3	9.51
Q8JZQ9	Eif3b	12	7	7	11	803	91.3	5.02
P28352	Apex1	26	6	6	10	317	35.5	7.91
Q8C8N2	Scai	14	7	7	13	606	70.2	8.6
Q9CXY6	Ilf2	21	5	5	7	390	43	5.26
P35438	Grin1	7	8	8	17	938	105.4	8.84
Q61233	Lcp1	14	5	7	14	627	70.1	5.33
Q62376	Snrnp70	20	9	9	10	448	52	9.94
Q9CQ19	Myl9	34	1	5	12	172	19.8	4.92
Q9D0I9	Rars	12	8	8	14	660	75.6	7.55
P34914	Ephx2	12	5	5	9	554	62.5	6.19
Q91WK2	Eif3h	22	7	7	11	352	39.8	6.67
Q9JJY3	Smpd3	15	8	8	11	655	71.2	5.88
Q03517	Scg2	16	9	9	14	617	70.6	4.75
Q91YJ2	Snx4	14	6	6	11	450	51.7	5.8
Q6P5E4	Uggt1	5	7	7	11	1551	176.3	5.62
Q9CQM9	Glrx3	17	5	5	18	337	37.8	5.59
P62242	Rps8	29	5	5	14	208	24.2	10.32
Q922E4	Pcyt2	18	7	7	13	404	45.2	6.58
Q80UP3	Dgkz	7	6	6	10	929	104	8.09
P10833	Rras	20	3	4	8	218	23.7	6.79
O35250	Exoc7	12	8	8	14	697	79.9	6.98
Q61735	Cd47	11	3	3	21	303	33.1	8.63
Q8C0E2	Vps26b	21	7	7	11	336	39.1	7.37
Q8VEH3	Arl8a	31	3	5	11	186	21.4	7.77
Q921F4	Hnrnpll	13	6	6	12	591	64.1	5.85
Q9DB72	Btbd17	17	6	6	11	478	52.6	9.42
P47791	Gsr	14	6	6	12	500	53.6	7.99
Q6P542	Abcf1	13	9	9	11	837	94.9	6.51
P15327	Bpgm	29	7	7	12	259	30	7.06

Q9ERS2	Ndufa13	42	5	5	13	144	16.8	9.48
P63028	Tpt1	22	5	5	13	172	19.5	4.86
Q61166	Mapre1	25	4	5	9	268	30	5.22
Q9ERU9	Ranbp2	4	8	9	11	3053	340.9	6.18
Q9DBG6	Rpn2	16	6	6	6	631	69	5.81
Q8BNW9	Kbtbd11	15	6	6	10	633	67.9	5.39
P59279	Rab2b	23	1	4	8	216	24.2	6.68
Q6P5F9	Xpo1	10	9	9	10	1071	123	6.07
Q61036	Pak3	11	2	6	11	559	62.4	5.4
P42125	Eci1	22	6	6	16	289	32.2	8.98
P29387	Gnb4	24	3	7	23	340	37.4	6.16
Q80TL0	Ppm1e	10	6	6	9	749	83.4	4.97
Q8C7R4	Uba6	8	8	8	12	1053	117.9	6.11
Q9QZ06	Tollip	18	4	4	12	274	30.3	5.17
P15116	Cdh2	9	6	8	15	906	99.7	4.78
Q8BPM0	Daam1	8	8	10	17	1077	123.3	7.46
Q3UHX2	Pdap1	37	5	5	11	181	20.6	7.39
Q9CQR4	Acot13	34	4	4	14	140	15.2	8.82
P54728	Rad23b	18	7	9	15	416	43.5	4.83
P24270	Cat	17	9	9	13	527	59.8	7.88
Q6ZWX6	Eif2s1	31	10	10	11	315	36.1	5.08
Q62077	Plcg1	7	9	9	10	1302	149.6	5.8
Q80ZI6	Lrsam1	13	8	8	8	727	83.9	6.14
Q64737	Gart	10	8	8	12	1010	107.4	6.68
P47962	Rpl5	23	7	7	14	297	34.4	9.77
Q8R4N0	Clybl	26	7	7	9	338	37.5	8.54
Q8BLF1	Nceh1	13	4	4	12	408	45.7	7.05
P47955	Rplp1	53	2	2	5	114	11.5	4.32
Q9JL62	Gltp	26	6	6	14	209	23.7	7.39
P60229	Eif3e	17	6	6	8	445	52.2	6.04
Q6P1B1	Xpnpep1	15	9	9	10	623	69.5	5.54
Q8R1B4	Eif3c	10	10	10	15	911	105.5	5.78
Q60737	Csnk2a1	15	5	5	7	391	45.1	7.74
Q80VD1	Fam98b	11	4	4	12	429	45.3	8.5
Q9R0N5	Syt5	8	1	3	18	386	43.1	9.6
P62774	Mtpn	33	4	4	14	118	12.9	5.52
Q6ZQ82	Arhgap26	12	10	10	13	814	92	6.68
Q62465	Vat1	13	5	5	12	406	43.1	6.37
Q68FL6	Mars	10	9	9	13	902	101.4	7.14
Q91Z61	Diras1	34	4	7	12	198	22.3	8.9
P46735	Myo1b	6	6	6	10	1107	128.5	9.26
O70439	Stx7	32	7	7	12	261	29.8	5.78
O88952	Lin7c	40	3	6	10	197	21.8	8.43
Q9JIX8	Acin1	6	6	6	9	1338	150.6	5.91

Q91X97	Ncald	46	4	7	20	193	22.2	5.35
Q99L04	Dhrs1	27	7	7	9	313	34	8.35
Q569Z6	Thrap3	8	7	7	12	951	108.1	10.17
Q9CQI3	Gmfb	26	4	4	14	142	16.7	5.16
Q61646	Hp	15	6	6	10	347	38.7	6.29
Q8VCI5	Pex19	25	5	5	7	299	32.7	4.34
Q8C754	Vps52	7	3	3	9	723	82	5.9
O35381	Anp32a	25	4	7	12	247	28.5	4.07
Q9DC70	Ndufs7	18	5	5	15	224	24.7	9.92
Q9Z329	Itpr2	3	1	8	12	2701	307.3	6.44
P35922	Fmr1	12	4	6	9	614	68.9	7.62
Q9D4H8	Cul2	10	8	8	18	745	86.8	7.01
Q9DC51	Gnai3	22	4	7	10	354	40.5	5.69
O88958	Gnpda1	20	4	5	7	289	32.5	6.6
Q3UH66	Wnk2	5	6	6	9	2149	227.4	5.91
Q8R001	Mapre2	17	5	6	12	326	36.9	5.38
Q56A07	Scn2b	29	6	6	12	215	24.2	6.54
Q8R151	Znfx1	6	8	8	10	1909	218.7	7.47
O88704	Hcn1	12	7	8	9	910	102.4	8.37
Q99L43	Cds2	13	5	5	14	444	51.3	7.05
P54823	Ddx6	19	7	7	9	483	54.2	8.66
Q9JKK1	Stx6	23	5	5	6	255	29	4.92
Q8BL65	Ablim2	12	5	5	10	612	68.1	8.02
Q9QZF2	Gpc1	15	6	6	7	557	61.3	7.05
Q61147	Cp	11	9	9	9	1061	121.1	5.85
P04627	Araf	9	2	5	9	604	67.5	9.11
Q80X80	C2cd2l	17	8	8	13	706	76.3	7.21
Q9WVA4	Tagln2	37	5	6	8	199	22.4	8.24
O08810	Eftud2	12	7	8	9	971	109.3	5
P67871	Csnk2b	20	3	3	9	215	24.9	5.55
P54822	Adsl	11	6	6	10	484	54.8	7.27
Q9QYG0	Ndrg2	26	7	7	11	371	40.8	5.4
Q6PER3	Mapre3	25	5	7	16	281	31.9	5.54
P97494	Gclc	17	8	8	10	637	72.5	5.9
Q78JE5	Fbxo22	21	7	7	8	402	44.2	7.61
Q8BYM5	Nlgn3	8	4	4	9	825	91.1	5.8
Q9CQI6	Cotl1	43	5	5	19	142	15.9	5.4
P32848	Pvalb	56	5	5	21	110	11.9	5.19
Q9D415	Dlgap1	9	7	9	12	992	110.3	7.09
P27671	Rasgrf1	7	7	7	9	1262	144	7.17
Q9D2M8	Ube2v2	36	1	4	18	145	16.4	8.09
P21981	Tgm2	13	9	9	12	686	77	5.1
Q8R164	Bphl	27	8	8	14	291	32.8	8.94

A2AJA9	Ajm1	12	10	10	12	974	107.1	9.11
P40237	Cd82	16	4	4	10	266	29.6	5.02
Q6ZQI3	Mlec	25	7	7	11	291	32.3	6.05
P10922	H1-0	23	4	4	10	194	20.8	10.9
Q99KV1	Dnajb11	26	6	6	8	358	40.5	6.32
Q8CHT0	Aldh4a1	13	8	8	10	562	61.8	8.24
Q9D6J5	Ndufb8	32	5	5	9	186	21.9	6.64
Q99MR6	Srrt	9	7	7	12	875	100.4	5.97
A2AG50	Map7d2	10	8	8	12	781	86	8.81
Q6PDY2	Ado	30	7	7	11	256	28.4	5.97
O88696	Clpp	25	5	5	11	272	29.8	7.47
P17563	Selenbp1	15	5	5	8	472	52.5	6.29
P01831	Thy1	31	4	4	17	162	18.1	8.97
Q9R1R2	Trim3	11	5	8	9	744	80.7	7.81
P29391	Ftl1	36	4	4	9	183	20.8	6
O88441	Mtx2	28	4	4	7	263	29.7	5.63
E9Q7X7	Nrxn2	5	6	8	9	1710	184.8	5.9
Q9CRD2	Emc2	19	4	4	9	297	34.9	6.81
P61087	Ube2k	24	4	4	10	200	22.4	5.44
Q8BHL5	Elmo2	13	7	9	14	732	83.8	5.95
Q80VP1	Epn1	12	6	6	11	575	60.2	4.81
O35954	Pitpnm1	6	6	7	11	1243	134.9	6.06
P61021	Rab5b	26	2	5	9	215	23.7	8.13
P51855	Gss	18	7	7	9	474	52.2	5.8
O35678	Mgll	20	6	6	11	303	33.4	7.15
P62082	Rps7	23	5	5	13	194	22.1	10.1
Q60973	Rbbp7	13	2	5	10	425	47.8	5.05
P51880	Fabp7	23	2	2	8	132	14.9	5.63
Q5DTT2	Psd	7	7	7	10	1024	109.6	7.05
Q80VC9	Camsap3	6	7	8	11	1252	135.1	8.43
Q8QZY1	Eif3l	16	7	7	9	564	66.6	6.44
P70414	Slc8a1	8	8	8	16	970	108	5
A2ASZ8	Slc25a25	14	5	6	10	469	52.6	8.54
Q91YP2	Nln	13	9	9	14	704	80.4	6.44
Q9D7A8	Armc1	19	5	5	8	282	31.2	5.57
Q9EQK5	Mvp	8	7	7	10	861	95.9	5.59
Q6PF93	Pik3c3	13	10	10	11	887	101.4	6.73
Q6PDL0	Dync1li2	19	7	7	10	492	54.2	6.28
Q9D164	Fxyd6	27	2	2	6	94	10.4	5.11
Q9CR26	Vta1	26	6	6	8	309	33.9	6.13
Q5FWK3	Arhgap1	17	8	8	11	439	50.4	6.44
Q8BWY3	Etf1	13	5	5	8	437	49	5.71
Q8BH57	Wdr48	13	8	8	11	676	76	7.17
Q9ESK9	Rb1cc1	6	7	7	9	1588	182.2	5.49

Q8K411	Pitrm1	8	9	9	12	1036	117.3	7.2
P97390	Vps45	11	8	8	11	570	65	8.25
O89084	Pde4a	11	5	8	12	844	93.5	5.26
Q8BWR2	Pithd1	29	4	4	10	211	24.2	5.74
O70305	Atxn2	7	8	8	10	1285	136.4	9.55
P07309	Ttr	31	4	4	13	147	15.8	6.16
P62482	Kcnab2	25	5	6	9	367	41	9
Q9Z2C4	Mtmr1	11	7	7	8	669	75.3	6.8
Q4V9Z5	Sez6l2	9	6	6	10	910	97.4	4.87
Q6PE13	Prrt3	9	7	7	10	971	101.2	7.52
Q6PDQ2	Chd4	4	6	6	7	1915	217.6	5.81
P59325	Eif5	19	6	6	9	429	48.9	5.52
O35286	Dhx15	10	8	8	17	795	90.9	7.46
Q9QXB9	Drg2	15	5	5	10	364	40.7	8.88
Q62448	Eif4g2	10	8	8	9	906	102	7.14
P22315	Fech	17	7	7	12	420	47.1	8.91
Q9DAK9	Phpt1	52	6	6	15	124	14	5.53
P14576	Srp54	18	8	8	8	504	55.7	8.75
P61028	Rab8b	27	4	6	14	207	23.6	9.07
O70166	Stmn3	38	7	7	12	180	20.9	7.49
Q8BFU3	Rnf214	14	9	9	15	668	73.6	6.23
B9EJ86	Osbpl8	7	7	7	13	889	101.2	6.96
Q8K012	Fnbp1l	9	6	6	13	605	69.8	6.64
Q99LD8	Ddah2	26	5	6	10	285	29.6	6.01
Q9Z1Q2	Abhd16a	15	7	7	10	558	63	8.25
Q69ZK9	Nlgn2	11	4	6	10	836	90.9	6.18
Q9QZE7	Tsnax	19	5	5	9	290	32.9	6.55
P29533	Vcam1	11	6	6	8	739	81.3	5.3
Q80T41	Gabbr2	10	8	8	11	940	105.6	8.72
P51660	Hsd17b4	9	6	6	10	735	79.4	8.57
P35979	Rpl12	39	5	5	15	165	17.8	9.42
Q7M750	Opalin	32	3	3	11	143	15.8	4.88
Q3UHC7	Dab2ip	5	5	7	14	1189	131.6	8.72
Q9D7P6	Iscu	40	6	6	9	168	18.1	9.29
O54946	Dnajb6	13	4	5	13	365	39.8	9.36
P24369	Ppib	27	6	6	12	216	23.7	9.55
Q9EP89	Lactb	10	6	6	7	551	60.7	8.9
Q9DB41	Slc25a18	20	5	7	11	320	34.1	9.19
Q9WUM5	Suclg1	19	6	6	12	346	36.1	9.39
Q9CQ65	Mtap	23	5	5	8	283	31	7.14
Q91Z31	Ptbp2	12	4	4	8	531	57.5	8.66
D3YZP9	Ccdc6	12	5	5	8	469	52.9	7.34
Q8VBW6	Nae1	17	6	6	12	534	60.2	5.52
Q80VL1	Tdrkh	13	6	6	8	560	62.1	4.94

Q8R5J9	Arl6ip5	24	5	5	9	188	21.5	9.61
Q11136	Pepd	17	7	7	9	493	55	5.78
P16045	Lgals1	51	6	6	12	135	14.9	5.49
Q922D4	Ppp6r3	12	7	7	10	844	94.6	4.58
Q80WC7	Agfg2	14	5	6	8	479	48.9	9.1
P58389	Ptpa	23	6	6	14	323	36.7	6.39
Q99K70	Rragc	16	2	3	6	398	44.1	5.1
A2A432	Cul4b	8	6	8	10	970	110.6	8.37
P34022	Ranbp1	29	5	5	9	203	23.6	5.22
Q9D4H1	Exoc2	9	6	6	8	924	103.9	7.18
Q791T5	Mtch1	15	7	7	13	389	41.5	9.32
Q9D8U8	Snx5	15	5	6	8	404	46.8	6.62
O70228	Atp9a	8	6	7	11	1047	118.5	7.69
O88322	Nid2	6	7	7	10	1403	153.8	5.38
Q9DD18	Dtd1	25	5	5	12	209	23.4	7.87
O35658	C1qbp	15	4	4	18	278	31	4.92
P63001	Rac1	31	5	6	15	192	21.4	8.5
O35495	Cdk14	12	5	5	9	469	53	8.92
P56546	Ctbp2	11	1	6	15	445	48.9	6.95
Q64336	Tbr1	14	7	7	8	681	73.9	7.33
Q01339	Apoh	19	6	6	10	345	38.6	8.22
Q1RLL3	Cpne9	9	1	5	8	553	61.8	5.4
O88738	Birc6	2	8	8	8	4882	531.8	6.07
Q6X893	Slc44a1	9	5	5	8	653	73	8.75
P51410	Rpl9	30	4	4	9	192	21.9	9.95
Q9EP69	Sacm1l	11	7	7	15	587	66.9	7.3
Q8C163	Exog	17	4	4	8	368	41.4	8.12
P08071	Ltf	11	7	7	9	707	77.8	8.53
Q9DBE8	Alg2	16	5	5	10	415	47.4	7.97
Q8CIN4	Pak2	18	4	6	9	524	57.9	5.77
Q8BG18	Necab1	11	2	2	6	352	40.9	4.89
Q925E7	Ppp2r2d	17	2	6	12	453	51.9	6.49
P97393	Arhgap5	4	5	5	7	1501	172	6.34
O35215	Ddt	55	5	5	10	118	13.1	6.54
Q6KAU4	Mvb12b	17	4	4	6	317	35.4	7.93
Q8R1N4	Nudcd3	25	7	7	7	363	40.9	5.26
Q8BMI3	Gga3	9	5	5	9	718	77.9	5.66
Q8BU31	Rap2c	27	1	4	8	183	20.7	4.94
Q8BHL3	Tbc1d10b	8	8	8	14	798	87.2	8.9
Q8BLY2	Tarsl2	11	8	8	12	790	91.3	7.53
Q61282	Acan	4	7	7	11	2132	221.8	4.3
Q6P4S6	Sik3	5	6	6	11	1311	145.7	6.92
O08848	RO60	10	7	7	12	538	60.1	7.9
P36993	Ppm1b	17	6	6	8	390	42.8	5.19

Q8BJU0	Sgta	14	4	4	13	315	34.3	5.06
Q8C052	Map1s	8	7	7	9	973	102.9	7.02
Q8BFZ9	Erlin2	15	4	5	8	340	37.8	5.5
Q99JR5	Tinagl1	11	5	5	9	466	52.6	6.77
Q9WUL7	Arl3	31	4	4	9	182	20.5	7.24
Q99K28	Arfgap2	13	6	6	7	520	56.6	8.18
Q8CG76	Akr7a2	11	4	4	9	367	40.6	8.12
A2A690	Tanc2	5	8	8	9	1994	220.1	7.97
P63073	Eif4e	17	4	4	12	217	25	6.15
Q8R3P0	Aspa	17	6	6	12	312	35.3	6.58
Q61033	Tmpo	14	2	7	11	693	75.1	8.05
O35841	Api5	10	4	4	7	504	56.8	5.92
P59017	Bcl2l13	15	5	5	10	434	46.7	4.59
Q9DBT5	Ampd2	11	8	8	10	798	92	6.23
Q99JP7	Ggt7	11	6	6	11	662	70.2	5.06
P58774	Tpm2	23	3	8	13	284	32.8	4.7
O70566	Diaph2	7	7	7	8	1098	124.8	6.92
O08914	Faah	9	4	4	6	579	63.2	7.87
Q99J85	Nptxr	17	8	8	8	493	52.3	6.13
Q91XU3	Pip4k2c	16	6	7	24	421	47.3	6.89
								11.1
Q9D2P8	Mobp	22	5	5	12	170	19.2	4
P54285	Cacnb3	9	3	4	10	484	54.5	6.27
B2RXS4	Plxnb2	4	7	7	9	1842	206.1	5.87
Q8JZW5	Sh2d5	23	7	7	13	429	47.4	8.47
Q0VBD0	Itgb8	9	6	6	9	767	84.5	7.09
P32261	Serpinc1	15	6	6	8	465	52	6.46
Q6PAK3	Prmt8	15	3	5	11	394	45.2	6.93
Q8CHP8	Pgp	21	5	5	10	321	34.5	5.35
Q6PHU5	Sort1	5	5	5	9	825	91.1	5.88
Q6I6G8	Hecw2	4	4	6	10	1578	176.1	5.41
P61202	Cops2	16	6	6	9	443	51.6	5.53
Q8VIM9	Irgq	15	7	7	9	583	59.3	4.83
Q9D855	Uqcrb	36	6	6	12	111	13.5	9.11
Q8R4U7	Luzp1	8	8	8	8	1068	119.2	7.99
Q8VBZ3	Clptm1	12	5	5	6	664	75.2	6.3
Q8K354	Cbr3	29	5	5	8	277	30.9	6.57
Q7TQH0	Atxn2l	8	8	8	11	1049	110.6	8.85
Q922H2	Pdk3	20	8	8	12	415	47.9	8.82
Q9JM96	Cdc42ep4	17	4	4	7	349	37.8	5.36
P70372	Elavl1	16	5	5	8	326	36.1	9.04
Q61941	Nnt	5	5	5	8	1086	113.8	7.64
Q8K386	Rab15	33	5	5	14	212	24.3	5.71
P10711	Tcea1	14	5	5	8	301	33.9	8.38

Q9ERG2	Strn3	7	5	6	12	796	87.1	5.29
Q80VQ0	Aldh3b1	14	5	5	8	468	52.3	7.55
Q6PDS3	Sarm1	12	8	8	8	724	79.6	6.27
Q61024	Asns	12	5	5	6	561	64.2	6.58
P21447	Abcb1a	7	9	9	11	1276	140.6	8.85
O55022	Pgrmc1	21	4	5	10	195	21.7	4.7
P62751	Rpl23a	25	5	5	12	156	17.7	10.45
Q91ZP9	Necab2	22	7	7	8	389	43.4	5.3
P31001	Des	11	3	7	19	469	53.5	5.27
Q6ZWV3	Rpl10	14	4	4	10	214	24.6	10.08
Q8R146	Apeh	8	6	6	8	732	81.5	5.59
Q8C2Q3	Rbm14	10	7	7	10	669	69.4	9.67
Q3THG9	Aarsd1	22	6	6	7	412	44.9	6.42
P52196	Tst	23	5	5	9	297	33.4	7.85
Q99K10	Nlgn1	6	2	4	8	843	94.1	5.99
P57759	Erp29	21	5	5	11	262	28.8	6.15
O54941	Smarce1	10	3	4	18	411	46.6	4.88
Q60870	Reep5	16	4	4	8	185	21	8.38
P63141	Kcna2	16	4	4	6	499	56.7	4.86
Q3U1F9	Pag1	16	4	4	5	429	46.5	4.81
O54781	Srpk2	11	4	7	9	681	76.7	4.91
O55125	Nipsnap1	22	5	5	11	284	33.3	9.44
Q60631	Grb2	28	6	6	10	217	25.2	6.32
Q8C0L0	Tmx4	18	6	6	9	335	37.1	4.37
Q8JZK9	Hmgcs1	14	5	6	8	520	57.5	5.99
Q8CHX7	Rftn2	17	6	6	7	500	54.9	5.5
A2AJI0	Map7d1	8	7	7	10	846	93.2	10.15
Q9D1P4	Chordc1	27	5	5	7	331	37.3	7.9
Q61290	Cacna1e	4	7	7	8	2272	257.1	8.18
Q3UGY8	Arfgef3	4	7	7	10	2170	239.9	5.88
Q9QYI3	Dnajc7	12	6	6	8	494	56.4	6.49
Q9WVA3	Bub3	18	4	4	9	326	36.9	6.84
Q4VAA2	Cdv3	21	6	6	9	281	29.7	6.1
Q6RHR9	Magi1	6	7	7	8	1471	161.9	7.36
Q60675	Lama2	2	7	7	9	3118	343.6	6.09
A2A699	Fam171a2	12	7	7	8	822	87.4	8.05
Q8VDC0	Lars2	9	7	7	8	902	101.4	8.19
P12849	Prkar1b	13	3	6	8	381	43.2	5.96
Q5SXY1	Specc1	7	7	7	9	1067	118	6.64

Q6ZWN5	Rps9	34	7	7	17	194	22.6	10.65
Q8K406	Lgi3	15	8	8	10	548	61.8	8.16
Q8C7X2	Emc1	5	4	4	10	997	111.5	7.43
Q9QZX7	Srr	24	5	5	8	339	36.3	6.02
Q6VNB8	Wdfy3	2	8	8	8	3508	392.1	6.8
Q9JLZ3	Auh	15	5	5	9	314	33.4	9.51
Q8K0D5	Gfm1	11	8	8	9	751	83.5	6.92
O35621	Pmm1	37	8	8	9	262	29.8	5.47
Q8R016	Blmh	20	6	6	9	455	52.5	6.48
Q8CCK0	H2afy2	20	5	5	7	372	40.1	9.69
Q9DAW9	Cnn3	18	3	4	9	330	36.4	5.72
P40336	Vps26a	21	6	6	9	327	38.1	6.57
Q9CQC7	Ndufb4	36	3	3	9	129	15.1	9.89
Q923D2	Blvrb	24	5	5	11	206	22.2	7.01
Q9JKV1	Adrm1	15	5	5	7	407	42	5.07
P47809	Map2k4	16	6	6	11	397	44.1	8.07
Q91WC0	Setd3	10	5	5	8	594	67.1	5.6
Q8CAY6	Acat2	20	6	6	9	397	41.3	7.5
Q68EF6	Begain	13	7	7	8	600	65.3	5.87
Q9R1P1	Psmb3	32	5	5	6	205	22.9	6.55
Q4VAE3	Tmem65	22	5	5	10	234	24.9	7.78
O09044	Snap23	19	2	3	18	210	23.2	4.98
Q60972	Rbbp4	12	2	5	10	425	47.6	4.89
Q5XJY5	Arcn1	15	7	7	9	511	57.2	6.21
A2APX8	Scn1a	4	4	8	8	2009	228.7	5.87
Q9DCM2	Gstk1	28	5	5	7	226	25.7	8.88
Q8BYK6	Ythdf3	11	2	5	5	585	63.9	9.04
P62835	Rap1a	33	1	6	14	184	21	6.67
P63276	Rps17	32	4	4	9	135	15.5	9.85
Q8C7M3	Trim9	9	7	7	10	817	90.8	7.24
P32233	Drg1	24	6	6	6	367	40.5	8.9
Q9JL26	Fmnl1	8	7	8	9	1094	122	5.82
Q9EQU5	Set	14	4	4	11	289	33.4	4.32
Q62093	Srsf2	14	3	3	9	221	25.5	11.85
Q9D8W7	Ociad2	39	6	6	12	154	16.9	9.41
P35980	Rpl18	20	3	3	9	188	21.6	11.78
P83510	Tnik	5	3	5	8	1323	150.3	7.27
Q66JS6	Eif3j2	23	6	6	11	263	29.5	4.81
Q8CHG3	Gcc2	3	5	5	6	1679	194.3	5.12
Q8K400	Stxbp5	6	5	5	8	1152	127.6	7.21
P49615	Cdk5	25	6	6	13	292	33.3	7.66

Q8VDK1	Nit1	21	5	5	8	323	35.7	7.94
Q9QZQ8	H2afy	20	4	4	8	372	39.7	9.8
P45952	Acadm	17	7	7	9	421	46.5	8.37
Q9CQ75	Ndufa2	41	5	5	14	99	10.9	9.99
Q8R3Q2	Ppp6r2	8	4	4	5	923	100.4	4.82
P70207	Plxna2	3	2	7	11	1894	211.4	6.54
Q62048	Pea15	23	4	4	15	130	15	5.02
Q5XG69	Fam169a	8	4	4	7	665	73.2	4.68
Q9WUT3	Rps6ka2	10	7	7	11	733	83.1	8.5
P50171	Hsd17b8	20	3	3	5	259	26.6	6.54
Q8BZN6	Dock10	4	7	7	8	2150	245.6	7.05
Q9QYB1	Clic4	37	6	6	8	253	28.7	5.59
Q8BG40	Katnb1	11	6	6	10	658	72.6	7.27
Q9WV18	Gabbr1	9	8	8	10	960	108.1	8.21
Q920N7	Syt12	14	6	6	8	421	46.7	5.64
Q9JHR7	Ide	6	6	6	10	1019	117.7	6.54
O35459	Ech1	16	4	4	6	327	36.1	7.71
Q8R361	Rab11fip5	8	5	5	9	645	69.5	9.07
O35435	Dhodh	16	5	5	7	395	42.7	9.55
P62301	Rps13	36	6	6	13	151	17.2	10.54
Q9JJC6	Rilpl1	17	5	5	6	406	47.3	5.16
Q9ESE1	Lrba	3	3	6	9	2856	316.9	5.69
Q9QUR8	Sema7a	12	6	6	8	664	74.9	7.74
O08915	Aip	18	5	5	8	330	37.6	6.4
Q8QZT2	Ccsap	24	6	6	9	252	28.4	9.22
Q80Y17	Llgl1	7	5	5	6	1036	112.5	6.48
Q8CCN5	Bcas3	7	5	5	9	928	101	6.7
Q80UU9	Pgrmc2	28	3	4	6	217	23.3	5.15
Q920Q4	Vps16	9	7	7	8	839	94.9	7.01
P36536	Sar1a	21	3	4	8	198	22.4	6.93
P00405	Mtco2	22	5	5	16	227	26	4.73
Q8C078	Camkk2	13	6	6	8	588	64.6	5.9
Q8BGN3	Enpp6	14	4	4	8	440	50.6	7.31
Q99LP6	Grpel1	29	6	6	11	217	24.3	8.38
Q99J99	Mpst	21	5	5	9	297	33.1	6.47
P42208	Septin2	19	6	6	9	361	41.5	6.55
P62743	Ap2s1	31	4	4	12	142	17	6.18
Q80TY0	Fnbp1	9	5	5	11	616	71.3	5.67
Q9CY64	Blvra	22	6	6	9	295	33.5	7.02
P70297	Stam	17	8	8	9	548	59.7	4.84
P47753	Capza1	24	3	5	10	286	32.9	5.55
Q7TPH6	Mycbp2	1	6	6	8	4749	520.9	7.09

Q9QZ88	Vps29	21	4	4	12	182	20.5	6.79
Q9DBS5	Klc4	15	5	8	9	619	68.6	6.09
Q8R2Y0	Abhd6	25	5	5	7	336	38.2	8.47
Q61702	Itih1	5	5	5	8	907	101	6.96
Q6ZWR4	Ppp2r2b	13	1	5	10	443	51.7	6.44
Q9D1X0	Nol3	22	4	4	6	220	24.6	4.07
Q99KX1	Mlf2	15	4	4	13	247	28	6.98
Q9DCZ4	Apoo	13	2	2	9	198	22.6	9.25
Q9EQQ9	Oga	5	6	6	9	916	103.1	4.92
Q8BY87	Usp47	7	7	7	9	1376	157.4	5.11
Q9QZS3	Numb	12	6	8	9	653	70.8	8.65
P28658	Atxn10	14	5	5	7	475	53.7	5.25
Q9D1T0	Lingo1	10	5	5	9	614	69.1	8.31
P05480	Src	14	7	7	10	541	60.6	7.84
Q9EQP2	Ehd4	8	3	4	9	541	61.4	6.76
P13634	Ca1	16	4	4	7	261	28.3	6.96
Q924N4	Slc12a6	6	3	6	11	1150	127.4	7.03
Q9WUQ2	Preb	11	3	3	5	417	45.4	8.76
Q69Z98	Brsk2	10	5	5	6	735	81.7	8.79
Q9CZ04	Cops7a	19	5	5	9	275	30.2	7.87
P63321	Rala	16	2	3	9	206	23.5	7.11
Q6A068	Cdc5l	9	5	5	6	802	92.1	8.02
P97765	Wbp2	26	4	4	7	261	28	6.33
Q8BIW1	Prune1	12	5	5	7	454	50.2	5.11
Q5SNZ0	Ccdc88a	4	8	8	8	1873	215.8	6.24
Q9D7X8	Ggct	22	4	4	10	188	21.2	5.67
Q3UV70	Pdp1	13	6	6	8	538	61.1	6.67
Q3UHK1	Slc2a13	8	5	5	9	637	69	6.86
Q8BX10	Pgam5	23	7	7	12	288	32	9.04
P62073	Timm10	71	7	7	11	90	10.3	6.29
Q9WVR4	Fxr2	6	1	3	5	673	73.7	6.23
Q9D8B7	Jam3	24	7	7	11	310	34.8	7.03
O88456	Capns1	24	5	5	7	269	28.4	5.63
Q8BVI5	Stx16	23	6	6	7	326	37.1	5.86
Q78IK2	Atp5md	28	2	2	10	58	6.4	9.83
Q99N96	Mrpl1	19	4	4	5	336	37.6	8.72
Q8JZU2	Slc25a1	18	6	6	8	311	33.9	9.89
Q8VD65	Pik3r4	5	6	6	9	1358	152.5	7.12
Q8VCX5	Micu1	16	8	8	9	477	54.3	8.59
Q6NVF9	Cpsf6	8	4	4	8	551	59.1	7.15
Q9CQE1	Nipsnap3b	16	3	3	6	247	28.3	9.48
P11404	Fabp3	23	3	3	10	133	14.8	6.57
Q62283	Tspan7	13	3	3	19	249	27.5	7.2

P80560	Ptprn2	8	5	6	6	1001	111.4	5.95
Q69Z26	Cntn4	6	5	5	7	1026	113.4	7.36
P06728	Apoa4	15	6	6	9	395	45	5.47
P55264	Adk	18	6	6	8	361	40.1	6.21
O54724	Cavin1	17	5	5	6	392	43.9	5.52
B2RUJ5	Apba1	11	6	7	9	842	92.9	4.88
Q6DFW4	Nop58	12	5	5	7	536	60.3	8.34
Q69ZH9	Arhgap23	6	5	5	8	1483	161.7	8.98
Q9EP53	Tsc1	7	6	6	6	1161	128.7	6.37
P62869	Elob	69	7	7	13	118	13.2	5.01
Q8K3G9	Appl2	10	5	5	6	662	73.8	5.03
Q8VD33	Sgtb	13	4	4	9	304	33.4	4.92
P10518	Alad	16	4	4	5	330	36	6.79
Q99KB8	Hagh	18	5	5	8	309	34.1	7.75
P50446	Krt6a	5	1	4	11	553	59.3	7.94
Q8CIQ7	Dock3	3	4	5	8	2027	232.8	6.99
Q03157	Aplp1	9	6	6	8	653	72.7	5.67
O08688	Capn5	12	7	7	8	640	72.9	7.36
Q9Z2M7	Pmm2	24	6	6	8	242	27.6	6.42
Q91YS8	Camk1	13	1	4	7	374	41.6	5.35
Q7TNC4	Luc7l2	12	3	5	11	392	46.6	10.1
P97379	G3bp2	15	8	8	11	482	54.1	5.62
P06684	C5	4	5	5	9	1680	188.8	6.81
P24668	M6pr	29	6	6	6	278	31.2	5.39
Q63912	Omg	11	5	5	12	440	49.3	8.41
Q8R574	Prpsap2	21	4	6	7	369	40.9	7.17
Q8K4P8	Hecw1	3	2	4	8	1604	179.4	5.39
Q99LY9	Ndufs5	34	4	4	8	106	12.6	8.92
P47740	Aldh3a2	17	6	6	6	484	53.9	8.35
Q9CYI4	Luc7l	13	2	4	9	371	43.9	9.88
P16283	Slc4a3	8	6	6	7	1227	135.3	6.51
Q8R010	Aimp2	16	3	3	6	320	35.4	7.83
P49813	Tmod1	20	6	6	7	359	40.4	5.1
Q9D024	Ccdc47	14	6	6	8	483	55.8	4.84
Q9WTR5	Cdh13	8	5	5	8	714	78.1	5.12
Q99M87	Dnaja3	14	6	6	7	480	52.4	9.22
Q9Z275	Rlbp1	19	5	5	7	317	36.4	5.06
P25444	Rps2	18	5	5	6	293	31.2	10.2<br>4
Q99JX3	Gorasp2	18	5	5	8	451	47	4.79
Q8JZR6	Slc4a8	5	2	6	8	1089	122.3	6.6
Q8R4H2	Arhgef12	5	5	5	7	1543	172.2	5.74
Q9Z1X4	Ilf3	6	5	5	7	898	96	8.76
Q8CC27	Cacnb2	6	1	4	8	655	73.1	8.47

P83093	Stim2	7	4	4	5	746	83.9	6.79
Q05D44	Eif5b	6	7	7	7	1216	137.5	5.59
Q5SUR0	Pfas	6	6	6	8	1337	144.5	5.67
O09131	Gsto1	24	5	5	10	240	27.5	7.36
Q9D906	Atg7	7	5	5	7	698	77.5	6.4
Q8BIJ7	Rufy1	7	4	5	7	712	80.3	5.68
Q80XR2	Atp2c1	8	6	6	8	918	100.2	6.83
Q9JMG7	Hdgfl3	26	3	4	6	202	22.4	8.4
P97429	Anxa4	21	5	6	7	319	35.9	5.57
P08414	Camk4	13	6	6	9	469	52.6	4.93
Q8CCJ3	Ufl1	8	4	5	6	793	89.5	6.67
Q8BK72	Mrps27	11	4	4	6	415	47.7	5.5
Q8C008	Dzank1	8	5	5	6	778	85	8.18
Q5DTX6	Jcad	6	6	6	7	1320	144.7	6.52
Q9Z0F7	Sncg	28	2	3	15	123	13.2	4.65
Q8K4Z0	Lgi2	7	4	4	10	550	63	6.54
Q99KH8	Stk24	17	4	6	7	431	47.9	5.43
P97370	Atp1b3	25	5	5	9	278	31.8	8.51
Q5M8N0	Cnrip1	23	2	2	7	164	18.6	7.96
Q8BJ71	Nup93	9	6	6	8	819	93.2	5.72
Q9D1Q6	Erp44	17	5	5	6	406	46.8	5.27
Q91VT4	Cbr4	19	3	3	6	236	25.4	9.69
Q8R1T1	Chmp7	12	4	4	6	451	50.6	5.22
Q9CQ62	Decr1	12	3	3	6	335	36.2	8.95
Q9ESM3	Hapln2	20	7	7	9	341	37.9	9.09
Q8BTI8	Srrm2	3	8	8	8	2703	294.7	12.03
P63323	Rps12	36	4	4	11	132	14.5	7.24
Q9CZS1	Aldh1b1	16	6	7	8	519	57.5	7.02
Q8BP67	Rpl24	29	5	5	10	157	17.8	11.25
O55017	Cacna1b	3	6	6	8	2327	261.3	8.59
P99028	Uqcrh	45	4	4	10	89	10.4	4.87
P83940	Eloc	47	4	4	12	112	12.5	4.78
Q9ER00	Stx12	23	5	5	7	274	31.2	5.44
Q7TNR6	Igsf21	9	4	4	7	468	51.9	6.95
Q9D7X3	Dusp3	35	5	5	8	185	20.5	6.54
O35066	Kif3c	8	4	6	7	796	89.9	8.06
Q8BI84	Mia3	3	6	6	7	1930	213.5	4.75
Q8BIK4	Dock9	3	5	5	6	2055	235.2	7.25
Q8WTY4	Ciapin1	16	4	4	6	309	33.4	5.2
Q9ERR1	Ndel1	14	5	5	8	345	38.3	5.24
Q9CPU0	Glo1	21	4	4	10	184	20.8	5.47
Q9D020	Nt5c3a	21	6	6	8	331	37.2	6.65

Q0VBF8	Stum	21	2	2	10	141	15	7.21
P56812	Pdcd5	31	4	4	9	126	14.3	5.68
Q8BH35	C8b	11	6	6	8	589	66.2	7.77
P28571	Slc6a9	8	4	4	7	692	76.5	7.81
Q62351	Tfrc	7	5	5	7	763	85.7	6.57
Q9Z0H4	Celf2	11	5	6	8	508	54.2	8.76
Q06335	Aplp2	11	6	7	9	707	80.4	4.7
Q9Z2V5	Hdac6	7	6	6	8	1149	125.7	5.78
Q8BGX2	Timm29	14	3	3	6	266	29.4	6.73
								10.9
P43276	H1-5	14	3	4	11	223	22.6	2
Q8BG51	Rhot1	9	5	5	6	631	72.2	6.49
P35585	Ap1m1	17	5	5	6	423	48.5	7.3
P01867	Igh-3	18	5	5	6	404	44.2	6.52
Q9CWE0	Mtfr1l	25	5	5	8	289	31.7	6.1
Q9D3D9	Atp5f1d	14	2	2	22	168	17.6	5.08
Q9CRA5	Golph3	15	3	3	4	298	33.7	6.44
P55288	Cdh11	8	5	5	6	796	88.1	4.89
Q9WVT6	Ca14	13	3	3	5	337	37.5	6.35
Q9D0M1	Prpsap1	21	4	6	6	356	39.4	7.2
P14206	Rpsa	26	5	5	6	295	32.8	4.87
Q9Z2Y8	Plpbp	24	6	6	10	274	30	8.27
Q60692	Psmb6	17	4	4	8	238	25.4	5.11
P11531	Dmd	2	6	6	9	3678	425.6	5.94
P55302	Lrpap1	17	5	5	7	360	42.2	7.87
B1AZP2	Dlgap4	6	4	4	5	992	108	7.08
Q9CT10	Ranbp3	14	5	5	7	491	52.5	5.12
Q69ZU6	Thsd7a	4	7	7	7	1645	183.4	7.2
Q6PAR5	Gapvd1	5	5	5	6	1458	162.3	5.19
Q64442	Sord	16	6	6	9	357	38.2	7.02
Q69ZK0	Prex1	4	6	6	10	1650	184.8	6.29
P62317	Snrpd2	40	4	4	8	118	13.5	9.91
P60867	Rps20	36	6	6	10	119	13.4	9.94
Q61315	Apc	2	6	6	7	2845	310.9	7.58
Q01063	Pde4d	10	4	7	8	747	84.5	4.91
Q8BXK8	Agap1	5	2	4	9	857	94.4	7.94
P21278	Gna11	11	3	4	15	359	42	5.97
Q9QUJ7	Acsl4	11	5	6	6	711	79	8.28
P47802	Mtx1	19	5	5	8	317	35.6	6.18
P59016	Vps33b	8	4	4	6	617	70.5	6.86
Q9JM76	Arpc3	24	5	5	8	178	20.5	8.59
Q62172	Ralbp1	9	5	5	6	648	75	5.92
Q9WUP7	Uchl5	19	5	5	7	329	37.6	5.33

Q9CR57	Rpl14	16	3	3	5	217	23.5	11.02
O70551	Srpk1	9	2	5	5	648	73	6.19
Q3URE1	Acsf3	11	6	6	6	583	65	8.02
Q91YE6	Ipo9	5	5	5	8	1041	116	4.81
P62858	Rps28	46	3	3	9	69	7.8	10.7
O88533	Ddc	14	5	5	6	480	53.8	6.6
Q8R3Z5	Cacnb1	7	1	4	8	597	65.4	6.62
Q9QUH0	Glrx	36	3	3	4	107	11.9	8.37
Q9Z0J4	Nos1	4	6	6	8	1429	160.4	7.15
P00493	Hprt1	22	4	4	6	218	24.6	6.68
Q9DC50	Crot	11	5	5	8	612	70.2	6.73
Q3UPH1	Prrc1	8	2	2	4	443	46.3	5.95
P83741	Wnk1	3	6	6	9	2377	250.8	6.43
Q8C080	Snx16	15	4	4	5	344	38.8	5.21
P08122	Col4a2	3	4	4	9	1707	167.2	8.48
Q9D7S9	Chmp5	21	4	4	7	219	24.6	4.79
P0C7L0	Wipf3	15	7	7	8	485	49.4	10.18
P97434	Mprip	8	7	7	9	1024	116.3	6.21
Q99J47	Dhrs7b	11	4	4	6	323	35	9.63
P01029	C4b	4	6	6	7	1738	192.8	7.53
P97492	Rgs14	10	4	4	5	547	59.8	7.43
Q9DBG5	Plin3	12	5	5	9	437	47.2	5.62
Q920P5	Ak5	14	6	6	7	562	63.3	5.29
Q60865	Caprin1	8	6	6	10	707	78.1	5.25
Q9CRY7	Gdpd1	13	3	3	7	314	35.8	8.31
Q9JKL5	Tesc	21	5	5	8	214	24.6	4.98
Q91XL9	Osbpl1a	5	5	5	7	950	107.7	6.44
P97355	Sms	11	5	5	10	366	41.3	5.06
Q8R1X6	Spart	9	4	4	4	671	72.6	5.86
Q9DAM7	Tmem263	42	3	3	4	115	11.5	9.32
Q8VE37	Rcc1	15	4	4	5	421	44.9	8.1
Q61464	Znf638	4	6	6	6	1960	218	6.89
A6H630	Armt1	8	5	5	10	439	50.5	5.92
P02535	Krt10	9	4	5	7	570	57.7	5.11
Q8BWG8	Arrb1	12	4	4	7	418	46.9	6.28
Q8K1Z0	Coq9	19	4	4	5	313	35.1	5.92
P35505	Fah	12	5	5	7	419	46.1	7.18
P97449	Anpep	6	5	5	7	966	109.6	5.9
Q9WTM5	Ruvbl2	11	4	4	6	463	51.1	5.64
P56395	Cyb5a	31	3	3	7	134	15.2	5.07
Q8R2R9	Ap3m2	8	3	4	7	418	46.9	7.56
Q9CQ54	Ndufc2	40	6	6	9	120	14.2	9.2

Q9DBS2	Tprg1l	24	3	3	4	266	29.8	7.37
P46935	Nedd4	7	6	6	8	887	102.6	5.26
Q8CJH3	Plxnb1	4	6	6	7	2119	231.2	5.55
P11031	Sub1	32	4	4	11	127	14.4	9.6
Q6NZB0	Dnajc8	17	4	4	10	253	29.8	9.06
Q6P2B1	Tnpo3	6	5	5	6	923	104.1	5.57
Q8R404	Micos13	50	3	3	6	119	13.4	8.63
Q62393	Tpd52	32	4	4	6	224	24.3	4.72
Q3UHD1	Adgrb1	5	5	5	7	1582	173.2	7.56
Q922P8	Tmem132 a	8	4	4	4	1018	110.2	5.63
Q9JLC8	Sacs	1	6	6	6	4582	520.4	7.11
P62889	Rpl30	22	2	2	6	115	12.8	9.63
Q8C167	Prepl	9	6	6	7	725	83.1	6.65
P11438	Lamp1	10	4	4	9	406	43.8	8.4
P16460	Ass1	13	6	6	11	412	46.6	8.22
Q9D892	Itpa	32	4	4	7	198	21.9	5.87
Q8BL86	Mblac2	21	5	5	7	279	31.2	6.86
Q8BGH4	Reep1	27	4	4	7	201	22.3	9.5
Q3UHH2	Slc22a23	7	5	5	7	689	74.3	7.83
P60521	Gabarapl 2	35	3	4	6	117	13.7	8.1
Q9CQJ6	Denr	25	4	4	7	198	22.2	5.3
P11352	Gpx1	28	4	4	9	201	22.3	7.21
P70392	Rasgrf2	6	6	6	6	1189	135.6	7.85
Q8R123	Flad1	11	4	4	5	492	54.7	6.64
Q91VW3	Sh3bgrl3	47	4	4	12	93	10.5	5.14
Q99JY8	Plpp3	17	5	5	8	312	35.2	9.07
P47941	Crkl	18	4	4	7	303	33.8	6.74
P49442	Inpp1	15	4	4	7	396	43.3	5.01
Q9D0G0	Mrps30	16	6	6	6	442	49.9	9.38
Q99NB9	Sf3b1	7	7	7	9	1304	145.7	7.09
Q9JM14	Nt5c	32	4	4	6	200	23.1	5.49
Q8BHG1	Nrdc	6	5	5	5	1161	132.8	4.87
Q9CXT8	Pmpcb	12	4	5	8	489	54.6	6.99
Q8BTZ7	Gmppb	8	3	3	6	360	39.9	6.74
Q9QXV0	Pcsk1n	23	4	4	6	258	27.3	5.85
O08808	Diaph1	4	4	4	6	1255	139.3	5.57
Q8JZP2	Syn3	11	4	6	10	579	63.3	9.38
P10107	Anxa1	14	4	4	6	346	38.7	7.37
P70335	Rock1	5	2	7	7	1354	158.1	5.86
P47856	Gfpt1	8	5	5	7	697	78.5	6.84
Q91W90	Txndc5	14	4	4	5	417	46.4	5.78
Q69ZZ6	Tmcc1	8	4	5	8	649	71.6	6.55

Q9Z0R6	Itsn2	4	5	8	10	1659	188.8	8.13
Q9WV98	Timm9	47	3	3	7	89	10.3	7.21
P61022	Chp1	20	3	3	6	195	22.4	5.1
P63242	Eif5a	31	3	3	8	154	16.8	5.24
O89051	Itm2b	11	2	2	8	266	30.2	5.3
O35988	Sdc4	22	4	4	6	198	21.5	4.41
E9Q8I9	Fry	2	5	5	7	3020	338.9	6.01
P62245	Rps15a	18	2	2	5	130	14.8	10.13
P27005	S100a8	30	2	2	4	89	10.3	5.68
P54763	Ephb2	6	4	5	6	986	109.8	5.71
Q9CPR4	Rpl17	22	4	4	10	184	21.4	10.18
Q8C7D2	Crbn	10	3	3	5	445	50.8	5.33
Q9WUC3	Ly6h	28	4	4	9	139	14.7	7.53
Q8BG02	Ppp2r2c	9	2	4	11	447	51.4	6.37
Q8R180	Ero1a	10	5	5	7	464	54.1	6.54
Q924C1	Xpo5	5	5	5	6	1204	136.9	5.82
Q8QZR5	Gpt	11	5	5	7	496	55.1	6.64
Q8VH51	Rbm39	9	4	4	7	530	59.4	10.1
Q8BJH1	Zc2hc1a	14	5	5	7	324	35.1	9.88
Q8K1S4	Unc5a	5	3	4	6	898	98.8	6.92
P97742	Cpt1a	6	4	4	6	773	88.2	8.62
P62484	Abi2	10	2	4	8	446	49.4	6.01
P46425	Gstp2	19	1	3	7	210	23.5	7.87
Q8VE33	Gdap1l1	10	3	3	9	370	42.3	6.6
Q60749	Khdrbs1	11	2	5	8	443	48.3	8.72
Q5SV85	Synrg	5	5	5	6	1306	139.5	5.03
O35350	Capn1	9	6	7	8	713	82.1	5.87
Q9EQ80	Nif3l1	14	6	6	9	376	41.7	6.76
Q64511	Top2b	4	6	6	7	1612	181.8	8.29
Q8R0F8	Fahd1	13	3	3	7	227	25.2	7.69
Q6KAR6	Exoc3	5	4	4	7	755	86.4	6.2
Q8VI75	Ipo4	5	4	4	7	1082	119.2	5.03
Q80XK6	Atg2b	3	5	5	5	2075	231.3	5.88
Q8VED9	Lgalsl	23	3	3	5	172	18.9	5.35
Q6PHS9	Cacna2d2	5	4	5	8	1154	130.3	5.66
A2AP18	Plch2	4	5	5	9	1501	164.2	7.49
Q9QX11	Cyth1	9	2	3	4	398	46.2	5.63
Q3UH93	Plxnd1	4	3	4	5	1925	211.5	7.11
Q7TN29	Smap2	13	5	5	8	428	46.5	8.87
Q9D2N4	Dtna	6	5	5	7	746	84	6.76
O54984	Asna1	11	4	4	7	348	38.8	4.91
Q5KU39	Vps41	6	4	4	6	853	98.5	5.81

Q8BML9	Qars	9	5	5	8	775	87.6	7.31
O08528	Hk2	5	1	4	13	917	102.5	6.11
O54734	Ddost	12	5	5	6	441	49	5.83
Q61207	Psap	8	5	5	7	557	61.4	5.19
Q9DBZ5	Eif3k	21	4	4	5	218	25.1	4.93
Q9EPE9	Atp13a1	4	5	5	6	1200	132.3	8.03
Q9CZX9	Emc4	28	4	4	5	183	20.1	8.62
Q9CR00	Psmd9	17	3	3	6	222	24.7	6.43
P10493	Nid1	5	6	6	7	1245	136.5	5.44
P32020	Scp2	9	4	4	6	547	59.1	7.44
P61222	Abce1	7	4	4	10	599	67.3	8.34
Q9CR51	Atp6v1g1	29	4	4	5	118	13.7	7.97
Q8VDM6	Hnrnpul1	8	6	6	6	859	95.9	6.58
A2ARS0	Ankrd63	11	4	4	7	390	41	10.83
P53026	Rpl10a	20	4	4	7	217	24.9	9.98
Q9CR61	Ndufb7	28	3	3	14	137	16.3	8.18
Q6NXK8	Asic1	6	4	4	7	526	59.6	5.58
P37804	Tagln	19	3	4	9	201	22.6	8.81
Q61037	Tsc2	3	4	4	5	1814	201.9	6.98
Q9CQZ5	Ndufa6	20	4	4	8	131	15.3	10.11
Q9DCB4	Arpp21	5	4	4	6	807	88.5	7.39
P70188	Kifap3	6	4	5	8	793	91.2	5.11
Q9CYR0	Ssbp1	21	3	3	6	152	17.3	9.92
P26450	Pik3r1	10	4	5	5	724	83.5	6.28
Q06185	Atp5me	37	3	3	17	71	8.2	9.35
Q8K0C9	Gmds	12	5	5	6	372	42	7.03
Q8K1A6	Cc2d1a	5	6	6	6	943	103.6	7.84
Q3TDN2	Faf2	10	4	4	5	445	52.4	5.47
Q8VHW2	Cacng8	9	4	4	9	423	43.4	9.2
O09167	Rpl21	29	5	5	8	160	18.6	10.49
Q3UJP5		26	5	5	6	209	23.8	6.86
Q6PHZ8	Kcnip4	21	4	4	5	250	28.7	5.21
P16014	Chgb	11	6	6	7	677	77.9	5.07
Q921H8	Acaa1a	12	4	4	5	424	43.9	8.44
Q8VEH5	Epm2aip1	8	4	4	5	606	70.1	5.87
Q61686	Cbx5	28	5	5	6	191	22.2	5.86
Q69ZS6	Sv2c	5	3	4	6	727	82.2	5.11
Q9D0J8	Ptms	23	3	3	30	101	11.4	4.22
Q6WQJ1	Dagla	6	5	5	7	1044	115.3	6.42
Q91V09	Wdr13	9	3	3	7	485	53.6	9.14
Q8VE70	Pdcd10	12	3	3	5	212	24.7	8.19

Q9QYR9	Acot2	10	2	4	6	453	49.6	7.36
O08919	Numbl	10	4	6	7	604	64.1	8.82
Q9JKN6	Nova1	6	3	3	6	507	51.7	8.72
Q91XD7	Creld1	14	6	6	7	420	45.7	5.02
A6H5Z3	Exoc6b	8	6	6	7	810	94.1	6.39
P17809	Slc2a1	9	3	3	7	492	53.9	8.87
P54754	Ephb3	5	3	4	4	993	109.6	6.28
Q80Y14	Glrx5	17	2	2	7	152	16.3	6.55
Q7TQ95	Lnpk	13	4	4	7	425	47.5	5.27
O35681	Syt3	7	4	4	9	587	63.2	6.65
Q3UVL4	Vps51	8	4	4	6	782	86.1	6.24
Q9EST5	Anp32b	9	1	4	8	272	31.1	4.01
Q8VHY0	Cspg4	3	5	5	5	2327	252.2	5.44
Q8BGD5	Cpt1c	9	5	5	7	798	90	7.49
Q9D6S7	Mrrf	14	2	2	5	262	29	9.85
Q3UKJ7	Smu1	14	5	5	7	513	57.5	7.18
Q9DC16	Ergic1	18	5	5	7	290	32.5	7.06
Q9EQ06	Hsd17b11	12	4	4	7	298	32.9	8.66
Q9JIG8	Praf2	20	3	3	6	178	19.5	9.6
O88545	Cops6	13	4	4	7	324	35.9	5.73
Q0VGU4	Vgf	9	5	5	6	617	68.2	4.7
P43275	H1-1	12	1	3	7	213	21.8	10.93
Q6P5H2	Nes	4	5	5	5	1864	207	4.34
Q9JJU8	Sh3bgrl	31	3	3	6	114	12.8	4.92
Q8VEK0	Tmem30a	17	4	4	6	364	41	8.37
O88951	Lin7b	29	3	6	10	207	22.9	8.68
Q9D0F3	Lman1	10	5	5	6	517	57.8	6.34
O09172	Gclm	14	3	3	5	274	30.5	5.52
Q8CFV4	Nrn1	19	3	3	5	142	15.3	6.99
P97352	S100a13	39	4	4	8	98	11.2	6.13
Q9R0P4	Smap	26	3	3	4	181	20	4.82
P61967	Ap1s1	32	5	5	7	158	18.7	5.73
Q8BFQ8	Gatd1	18	3	3	5	220	23.3	7.05
Q9CQ92	Fis1	24	3	3	5	152	17	8.53
P97411	Ica1	11	5	5	6	478	54.3	5.8
Q9D710	Tmx2	21	6	6	6	295	33.9	8.75
P01865	Igh-1a	8	1	2	5	398	43.9	6.29
Q9CY34	Ube2f	21	4	4	7	185	21.1	6.54
Q5SW19	Cluh	6	6	6	7	1315	148	6.02
P63216	Gng3	72	4	4	4	75	8.3	7.78
Q9D1C8	Vps28	15	3	3	7	221	25.4	5.54
P54923	Adprh	14	4	4	6	362	40	5.76
Q71RI9	Kyat3	7	3	3	7	455	51.1	8.37

Q3UHU5	Mtcl1	2	2	4	7	1945	213.7	6.14
Q62189	Snrpa	13	3	4	7	287	31.8	9.8
Q8BH44	Coro2b	11	6	6	10	480	54.9	8.27
P62918	Rpl8	18	5	5	11	257	28	11.03
Q9QYI5	Dnajb2	11	2	3	8	324	35.6	5.91
Q8CI32	Bag5	13	5	5	8	447	50.9	6.05
Q9CW03	Smc3	6	6	6	7	1217	141.5	7.18
P70699	Gaa	4	5	5	6	953	106.2	5.83
Q8BH58	Tiprl	14	4	4	12	271	31.2	5.63
Q99K01	Pdxdc1	7	6	6	6	787	87.3	5.48
Q923S9	Rab30	21	3	4	8	203	23	4.97
Q922J6	Tspan2	13	3	3	7	221	24.2	7.96
P10639	Txn	31	4	4	7	105	11.7	4.92
Q9CPW4	Arpc5	34	5	5	8	151	16.3	5.67
Q8JZR0	Acsl5	8	5	6	8	683	76.2	7.09
Q1HFZ0	Nsun2	6	3	3	6	757	85.4	6.58
Q8BFT9	Svop	8	3	3	4	548	60.7	5.85
Q8BWT5	Dip2a	4	6	6	9	1523	165.1	7.61
Q9QUN9	Dkk3	14	4	4	6	349	38.4	4.54
Q8CFE4	Scyl2	5	3	3	4	930	103.3	8.02
Q61704	Itih3	8	6	6	7	889	99.3	6.05
P53996	Cnbp	37	6	6	9	178	19.6	7.58
Q9WTR1	Trpv2	9	4	4	4	756	85.9	6.99
P70302	Stim1	7	6	6	8	685	77.5	6.54
P61358	Rpl27	30	3	3	12	136	15.8	10.56
Q3TCH7	Cul4a	7	3	5	7	759	87.7	8.35
Q3V0K9	Pls1	7	3	4	6	630	70.4	5.38
Q9D9V3	Echdc1	21	5	5	5	322	35.4	7.01
Q08274	Dmwd	9	4	4	4	665	69.8	7.24
Q6NS60	Fbxo41	5	5	5	7	873	94.3	8.24
Q9JHW2	Nit2	18	3	3	5	276	30.5	6.9
Q3U5Q7	Cmpk2	16	6	6	7	447	50	7.27
Q0VE82	Cpne7	7	2	3	5	557	61.9	5.4
Q8BRK8	Prkaa2	8	4	5	6	552	62	7.87
Q9CQJ8	Ndufb9	12	2	2	7	179	22	7.8
Q8BG95	Ppp1r12b	5	3	4	5	976	109	5.74
Q3UYG8	Macrod2	11	4	4	5	475	52.1	4.75
Q9DCT8	Crip2	22	3	3	7	208	22.7	8.63
Q9QYJ3	Dnajb1	16	3	4	6	340	38.1	8.63
Q69ZR2	Hectd1	2	5	5	6	2618	289.9	5.41
P63046	Sult4a1	13	4	4	6	284	33	5.53
P62069	Usp46	12	2	3	6	366	42.4	6.83

Q8C6E0	Cfap36	14	3	3	4	343	39.6	4.93
Q7TSC1	Prrc2a	2	4	5	7	2158	229.1	9.39
P0C192	Lrrc4b	4	2	3	6	709	76.1	7.24
Q8CIG8	Prmt5	6	4	4	8	637	72.6	6.42
Q9QUR7	Pin1	21	3	3	9	165	18.4	8.79
P15209	Ntrk2	8	3	5	9	821	92.1	6.55
Q3TKT4	Smarca4	4	4	5	5	1613	181.3	8
Q9ESX5	Dkc1	11	5	5	8	509	57.4	9.28
Q9DCJ1	Mlst8	17	4	4	5	326	35.8	5.86
Q9DAS9	Gng12	44	3	3	9	72	8	8.97
Q80U40	Rimbp2	6	5	5	5	1072	118.3	5.35
P61290	Psme3	14	4	4	5	254	29.5	5.95
Q8R5H1	Usp15	4	4	4	7	981	112.3	5.17
Q6GYP7	Ralgapa1	3	5	5	7	2035	229.2	6.1
D3Z6Q9	Bin2	9	3	5	7	489	52.5	5.34
Q3UIU2	Ndufb6	25	3	3	13	128	15.5	9.79
Q99JW2	Acy1	12	5	5	8	408	45.8	6.32
P04919	Slc4a1	8	5	5	5	929	103.1	5.45
P62270	Rps18	26	5	5	10	152	17.7	10.9<br>9
P22723	Gabrg2	11	5	5	9	474	55.1	8.66
Q5IRJ6	Slc30a9	10	6	6	7	567	62.8	8.69
Q9ET01	Pygl	6	3	6	7	850	97.4	7.09
P97772	Grm1	4	4	5	7	1199	133.1	6.86
Q3UTH8	Arhgef9	7	4	4	7	516	60.9	5.64
Q9QZM0	Ubqln2	8	2	3	13	638	67.3	5.22
Q9CQW2	Arl8b	24	2	4	6	186	21.5	8.43
Q9JK42	Pdk2	16	4	4	5	407	46	6.61
Q8R0H9	Gga1	6	4	4	6	635	69.9	5.27
P19221	F2	11	6	6	7	618	70.2	6.43
Q8BXR9	Osbpl6	6	5	5	5	959	108.9	7.27
Q8BGN8	Synpr	12	3	3	6	265	29.2	6.3
E9Q4S1	Pde8b	6	4	5	6	865	96.7	6.86
P68037	Ube2l3	16	3	3	9	154	17.9	8.51
Q9D6Y7	Msra	15	4	4	9	233	26	8.41
Q3TC72	Fahd2	20	4	4	6	313	34.7	8.16
P59764	Dock4	3	5	6	7	1978	226.4	7.65
P67984	Rpl22	30	3	3	6	128	14.8	9.19
Q8BMP6	Acbd3	13	5	5	6	525	60.1	5.11
Q9JJL8	Sars2	12	5	5	5	518	58.3	7.9
Q5SSZ5	Tns3	5	5	5	5	1440	155.5	6.65
Q8VC30	Tkfc	11	5	5	6	578	59.7	6.92
Q9ERL9	Gucy1a1	7	4	4	7	691	77.5	7.17
Q8VDQ1	Ptgr2	17	5	5	5	351	38	5.41

P19253	Rpl13a	15	3	3	8	203	23.4	11.02
Q62178	Sema4a	6	5	5	6	760	83.4	7.72
Q8C015	Pak5	8	5	5	7	719	80.9	8.21
P63080	Gabrb3	8	1	3	4	473	54.1	9.1
Q9WU28	Pfdn5	27	3	3	4	154	17.3	6.33
Q8C5W0	Clmn	5	6	6	6	1052	117.2	5.03
P20108	Prdx3	14	4	4	6	257	28.1	7.58
Q9CQ45	Nenf	26	4	4	4	171	18.9	5.27
Q80XA6	Reps2	12	4	5	6	521	57.7	7.53
Q6PEB6	Mob4	24	4	4	10	225	26	5.78
Q9CQU5	Zwint	22	4	4	5	252	28.7	8.43
Q3V1U8	Elmod1	11	3	3	7	326	38	8.4
Q62348	Tsn	11	2	2	5	228	26.2	6.44
Q9JK48	Sh3glb1	9	3	4	9	365	40.8	6.04
Q9QX47	Son	3	5	5	5	2444	265.5	5.6
P55088	Aqp4	6	2	2	21	323	34.4	7.42
O88703	Hcn2	5	3	4	7	863	94.7	8.73
Q9DB34	Chmp2a	15	3	3	5	222	25.1	5.97
Q9ER35	Fn3k	22	5	5	5	309	35	8.4
Q8C0D5	Efl1	4	5	5	6	1127	125.7	6.16
O08663	Metap2	10	4	4	5	478	52.9	5.82
Q60803	Traf3	8	5	5	8	567	64.3	8.05
O35226	Psmd4	13	3	3	3	376	40.7	4.79
Q69ZS8	Kazn	7	5	5	6	779	86.7	6.9
O70251	Eef1b	19	4	4	7	225	24.7	4.69
P60670	Nploc4	7	3	3	6	608	68	6.46
P06909	Cfh	3	4	4	5	1234	139	6.99
P70236	Map2k6	13	4	4	5	334	37.4	7.39
Q3TPX4	Exoc5	8	6	6	6	708	81.7	6.71
Q922Y1	Ubxn1	13	5	5	8	297	33.6	5.26
Q8CH25	Sltm	6	6	6	6	1031	116.9	7.72
O88998	Olfm1	10	5	5	11	485	55.4	6.95
Q9Z0V2	Kcnd2	8	4	4	5	630	70.5	8.07
P14115	Rpl27a	22	4	4	7	148	16.6	11.12
Q9R0M6	Rab9a	19	3	3	5	201	22.9	5.66
Q99J77	Nans	13	4	4	6	359	40	7.06
Q8K0G5	Eipr1	11	4	4	6	386	43.1	5.14
Q9QYH6	Maged1	4	3	3	5	775	85.6	7.5
Q61220	Nell2	5	5	5	6	819	91.4	5.82
Q6Y7W8	Gigyf2	4	4	5	7	1291	149.1	5.57
Q9JHS3	Lamtor2	34	3	3	8	125	13.5	5.4
Q80W21	Gstm7	11	1	3	7	218	25.7	6.8

A2BDX3	Mocs3	13	4	4	4	460	49.3	7.49
Q80W04	Tmcc2	5	2	3	6	706	77	6.84
Q6VNS1	Ntrk3	5	2	4	7	825	92.7	6.67
Q9WTL7	Lypla2	21	5	5	8	231	24.8	7.23
Q64012	Raly	15	5	5	6	312	33.2	8.84
P02469	Lamb1	3	4	5	8	1786	197	4.94
Q9CPT4	Mydgf	16	3	3	5	166	18	6.79
P51910	Apod	26	4	4	6	189	21.5	4.91
Q8CG72	Adprhl2	12	4	4	5	370	39.4	4.96
Q6PD03	Ppp2r5a	6	2	2	3	486	56.3	7.03
Q6PDI5	Ecpas	3	3	3	4	1840	203.6	7.06
Q6PA06	Atl2	8	3	4	6	583	66.2	5.43
Q9JMD3	Stard10	10	2	2	4	291	32.9	7.12
Q00915	Rbp1	23	3	3	5	135	15.8	5.25
Q9D1R9	Rpl34	28	4	4	11	117	13.3	11.47
Q9CWZ3	Rbm8a	27	4	4	6	174	19.9	5.72
Q8K0T4	Katnal1	10	4	4	4	488	55.1	7.09
Q9DB60	Prxl2b	18	4	4	6	201	21.7	6.74
Q922Q4	Pycr2	16	4	4	4	320	33.6	7.77
Q8K298	Anln	5	5	5	5	1121	122.7	6.98
Q8C3W1		16	4	4	5	322	35.3	5.24
Q03173	Enah	7	5	5	6	802	85.8	7.72
Q8BHC1	Rab39b	19	5	5	9	213	24.6	7.83
Q8CFI0	Nedd4l	6	6	6	6	1004	115.3	6.15
Q5SSM3	Arhgap44	6	4	4	5	814	88.9	6.6
Q9JI90	Rnf14	8	4	4	6	485	54.9	4.7
Q91VK4	Itm2c	23	4	4	4	269	30.5	8.59
Q9DC28	Csnk1d	9	3	4	6	415	47.3	9.74
Q3U9G9	Lbr	7	4	4	5	626	71.4	9.36
Q80YV4	Pank4	6	4	4	5	820	91.5	6.71
Q8BYN5	Fsd1l	9	3	3	4	507	56.9	6.7
Q05186	Rcn1	8	3	3	5	325	38.1	4.84
Q8R3F5	Mcat	10	3	3	4	381	41.9	8.1
Q9ESB3	Hrg	10	5	5	7	525	59.1	7.66
Q9R069	Bcam	7	4	4	6	622	67.6	6.25
Q9EPR5	Sorcs2	4	5	5	6	1159	128.8	7.34
P24288	Bcat1	9	3	3	7	386	42.8	5.39
Q5QNQ6	Osbp2	5	3	4	6	908	101.3	7.14
O70252	Hmox2	10	4	4	6	315	35.7	5.87
Q9QY42	Gpr37	7	2	2	5	600	66.7	8.12
Q59J78	Ndufaf2	28	4	4	7	168	19.6	8.25
Q9WVQ1	Magi2	4	5	5	5	1275	140.8	6.19
Q9JM13	Rabgef1	8	4	4	6	491	56.8	6.79

Q9CWM4	Pfdn1	29	4	4	8	122	14.2	8.32
Q99LE6	Abcf2	8	5	5	5	628	71.7	7.05
Q9DC61	Pmpca	8	4	4	4	524	58.2	6.83
Q60649	Clpb	8	5	5	6	677	76	8.51
Q8CHU3	Epn2	9	4	4	5	595	63.4	8.16
Q9D898	Arpc5l	13	2	2	8	153	17	6.8
Q810A7	Ddx42	9	4	4	5	929	101.9	6.98
Q80TH2	Erbin	4	4	5	9	1402	157.2	5.69
P60824	Cirbp	22	4	4	7	172	18.6	9.61
Q3V0G7	Garnl3	5	4	4	4	1038	115.4	7.17
Q921W4	Cryzl1	9	3	3	5	348	38.7	5.97
Q8K4X7	Agpat4	9	3	3	4	378	43.8	8.38
Q8VHR5	Gatad2b	8	3	3	3	594	65.4	9.7
P31725	S100a9	26	4	4	8	113	13	7.17
Q80WG5	Lrrc8a	8	5	5	5	810	94.1	7.94
P70290	Mpp1	12	4	4	6	466	52.2	7.2
Q9WUD1	Stub1	16	5	5	6	304	34.9	6.01
O35405	Pld3	6	2	2	6	488	54.4	6.52
Q91YD9	Wasl	9	3	3	4	501	54.2	7.93
Q8CI51	Pdlim5	9	5	5	5	591	63.3	8.25
P25976	Ubtf	8	5	5	8	765	89.5	5.76
Q80U19	Daam2	5	4	6	7	1115	128.3	6.92
P57776	Eef1d	17	4	4	5	281	31.3	5.02
Q91WU5	As3mt	13	3	3	4	376	41.8	5.86
Q68FD9	Kiaa1549	3	5	5	6	1940	209.1	6.1
P54726	Rad23a	13	3	5	6	363	39.7	4.58
Q8C3Q5	Shisa7	10	5	5	5	558	58.3	9.82
Q80TM9	Nisch	3	4	4	4	1593	174.9	5.16
Q9JIY5	Htra2	14	4	4	6	458	49.3	9.6
Q9WTQ8	Timm23	22	3	3	5	209	22	9.13
Q9D1D4	Tmed10	22	4	4	4	219	24.9	6.7
P45878	Fkbp2	19	2	2	4	140	15.3	8.88
P0C027	Nudt10	27	2	4	8	164	18.6	5.69
Q9R1K9	Cetn2	27	4	4	5	172	19.8	5
Q8R307	Vps18	3	3	3	4	973	110.1	6.09
Q61016	Gng7	54	3	3	5	68	7.5	8.51
P58021	Tm9sf2	7	4	4	11	662	75.3	7.43
Q9ERB0	Snap29	19	5	5	8	260	29.6	5.38
Q9DCM0	Ethe1	13	3	3	5	254	27.7	7.23
Q64444	Ca4	18	5	5	6	305	34.3	8.21
P17225	Ptbp1	6	2	2	4	527	56.4	8.34
O70311	Nmt2	8	4	4	6	529	60.4	7.8
Q922Q8	Lrrc59	15	6	6	9	307	34.9	9.52
Q8BQZ4	Ralgapb	3	5	5	5	1484	165.1	6.77

Q8R0G9	Nup133	5	4	4	5	1155	128.5	5.2
Q80XU3	Nucks1	15	3	3	4	234	26.3	5.14
P70202	Lxn	8	1	1	4	222	25.5	5.74
Q8BYW1	Arhgap25	5	4	4	5	648	73.3	6.38
P97371	Psme1	28	6	6	7	249	28.7	5.97
Q8VHK5	Mlc1	14	5	5	5	382	41.6	7.9
Q7SIG6	Asap2	3	3	3	6	958	106.7	6.65
Q505D1	Ankrd28	5	5	5	5	1053	112.8	6.27
Q8K0D0	Cdk17	9	4	5	7	523	59.5	9.03
								10.5
P62900	Rpl31	29	4	4	8	125	14.5	4
O54833	Csnk2a2	13	4	4	5	350	41.2	8.56
Q6NV83	U2surp	3	4	4	4	1029	118.2	8.47
Q921M4	Golga2	4	3	3	5	999	113.2	5
Q06138	Cab39	11	4	4	7	341	39.8	6.89
Q9CQR2	Rps21	37	3	3	9	83	9.1	8.51
Q3UDE2	Ttll12	5	3	3	5	639	74	5.63
Q9WTS4	Tenm1	2	4	4	4	2731	305.6	6.37
Q8BHE3	Atcay	12	3	3	6	372	42.2	4.68
Q80YX1	Tnc	3	5	5	5	2110	231.7	4.89
Q8BVL9	Jakmip1	6	4	4	5	626	73.1	6.09
Q99LS3	Psph	10	2	2	5	225	25.1	6.14
Q9CYN2	Spcs2	22	3	3	3	226	25	8.57
Q8BPQ7	Sgsm1	4	4	4	4	1093	123.1	5.66
O88271	Cfdp1	21	4	4	5	295	32.9	4.86
								11.2
P84244	H3-3a	20	1	4	20	136	15.3	7
Q8BMJ3	Eif1ax	19	3	3	6	144	16.5	5.24
Q9QXG2	Chm	5	4	4	6	665	73.9	4.68
Q3UMU9	Hdgfl2	6	3	4	6	669	74.2	8.66
O08692	Ngp	17	3	3	6	167	19.3	5.31
Q8BPU7	Elmo1	7	4	6	6	727	83.9	6.28
P14602	Hspb1	17	4	4	5	209	23	6.55
Q9EQZ7	Rims2	2	4	4	5	1530	172.8	9.25
Q91YH5	Atl3	7	4	4	6	541	60.5	6.1
Q9R0Q7	Ptges3	19	2	2	4	160	18.7	4.55
P11835	Itgb2	7	5	5	5	771	85	7.12
A2AHC3	Camsap1	3	5	5	7	1581	175.8	6.95
O55137	Acot1	8	1	3	4	419	46.1	6.58
Q9D404	Oxsm	11	4	4	4	459	48.6	7.06
O89086	Rbm3	22	4	4	8	153	16.6	7.5
P97441	Slc30a3	10	3	3	6	388	41.8	6.38
Q8K2C9	Hacd3	7	3	3	5	362	43.1	9.13
Q5XJV6	Lmtk3	3	3	3	4	1424	150.8	4.88

O08583	Alyref	14	3	3	8	255	26.9	11.15
P21126	Ubl4a	29	4	4	7	157	17.8	8.44
P59326	Ythdf1	7	1	4	4	559	60.8	8.95
Q9JLI8	Sart3	6	4	4	4	962	109.6	5.24
P70452	Stx4	13	4	4	6	298	34.1	6.14
Q6IRU2	Tpm4	13	3	4	8	248	28.5	4.68
Q04750	Top1	7	4	4	5	767	90.8	9.33
Q8CFI7	Polr2b	3	3	3	4	1174	133.8	6.87
Q60714	Slc27a1	4	2	2	4	646	71.2	8.38
Q99MR1	Gigyf1	5	5	5	5	1044	116.2	5.39
P51432	Plcb3	3	3	3	4	1234	139.4	5.94
P24547	Impdh2	7	3	4	7	514	55.8	7.28
Q8K4Z5	Sf3a1	8	5	5	5	791	88.5	5.22
Q80ZF8	Adgrb3	3	3	4	6	1522	171.2	7.02
Q91ZR1	Rab4b	16	2	3	4	213	23.6	6.06
P47915	Rpl29	11	2	2	4	160	17.6	11.84
P62075	Timm13	26	2	2	3	95	10.5	8.18
P17918	Pcna	13	2	2	4	261	28.8	4.77
Q9WU84	Ccs	18	3	3	4	274	28.9	6.1
Q61730	Il1rap	8	5	5	6	570	65.7	7.77
P62852	Rps25	30	4	4	6	125	13.7	10.11
P70280	Vamp7	19	3	3	5	220	25	8.6
P61514	Rpl37a	38	3	3	5	92	10.3	10.43
Q3TDK6	Rogdi	22	5	5	10	287	32.1	8.18
P97798	Neo1	2	4	4	5	1493	163.1	6.47
Q99KG3	Rbm10	4	3	3	3	930	103.4	5.92
O70591	Pfdn2	21	3	3	5	154	16.5	6.58
Q8BV13	Cops7b	12	2	2	4	264	29.7	6.32
O55142	Rpl35a	23	3	3	9	110	12.5	10.89
Q6ZQ29	Taok2	3	3	4	4	1240	139.2	7.06
Q9CRD0	Ociad1	28	5	5	8	247	27.6	7.81
Q8R2U6	Nudt4	17	1	3	6	179	20.1	6.35
Q5EG47	Prkaa1	8	3	4	7	559	63.9	8.12
Q9D8S4	Rexo2	12	2	2	4	237	26.7	7.15
P63034	Cyth2	6	1	2	5	400	46.6	5.63
O35691	Pnn	5	5	5	6	725	82.4	7.01
Q9QYY8	Spast	5	2	2	3	614	66.4	9.69
Q61627	Grid1	4	4	4	5	1009	112.1	6.7
Q9CY27	Tecr	8	3	3	6	308	36.1	9.55

P17665	Cox7c	29	2	2	8	63	7.3	11
A2AHG0	Lzts3	7	5	5	6	700	74.9	6.99
Q9Z2I2	Fkbp1b	25	2	2	5	108	11.8	8.47
Q7TT37	Elp1	5	5	5	6	1333	149.5	6
P68433	H3c1	20	1	4	19	136	15.4	11.12
Q9CQR6	Ppp6c	12	5	5	6	305	35.1	5.69
Q9JKX6	Nudt5	16	3	3	5	218	24	5.48
Q9WUA6	Akt3	10	3	4	6	479	55.7	6.02
P14106	C1qb	9	2	2	8	253	26.7	8.15
Q3THW5	H2afv	20	1	3	7	128	13.5	10.58
Q6P5F7	Ttyh3	7	3	3	4	524	57.7	5.85
Q9JKS5	Habp4	11	4	4	6	411	45.9	6.84
Q9JIS8	Slc12a4	5	1	5	8	1085	120.5	6.67
Q91YI0	Asl	9	4	4	5	464	51.7	6.96
Q91WG2	Rabep2	8	4	4	4	554	62.1	4.89
Q9CQS4	Slc25a46	15	4	4	4	418	46.2	7.64
O88845	Akap10	9	4	4	4	662	73.6	6.79
Q3TY60	Fam131b	11	3	3	5	332	35.9	4.45
P23492	Pnp	10	3	3	6	289	32.3	6.16
Q6P9R4	Arhgef18	5	6	6	6	1405	155.9	6.54
Q99KP6	Prpf19	9	4	4	6	504	55.2	6.61
Q8CJG0	Ago2	4	2	3	4	860	97.2	9.19
Q9R118	Htra1	5	2	2	4	480	51.2	7.65
P31750	Akt1	10	2	3	3	480	55.7	5.9
Q6P5U7	Nwd2	3	4	4	4	1742	197.3	6.15
Q14BB9	Map6d1	30	3	3	3	191	20.4	9.88
Q61187	Tsg101	11	5	5	6	391	44.1	6.71
Q7TNM2	Trim46	6	4	4	4	759	83.4	7.65
P70122	Sbds	10	3	3	5	250	28.8	8.76
Q3UXZ6	Fam81a	7	3	3	5	364	41.7	8.75
Q8BJZ4	Mrps35	13	4	4	7	320	36	8.59
Q9JME7	Trappc2l	19	3	3	6	139	16	6.77
Q9D880	Timm50	10	3	3	4	353	39.8	8.13
P97950	Rab33a	8	2	2	5	237	26.5	7.88
Q9CQ69	Uqcrq	46	4	4	4	82	9.8	10.26
Q9D964	Gatm	11	3	3	3	423	48.3	7.88
Q7TPB0	Plppr3	5	3	3	4	716	76.6	6.01
Q8BHG2	Czib	19	4	4	4	160	18	5.12
Q9D379	Ephx1	14	7	7	8	455	52.5	8.35
Q62446	Fkbp3	11	3	3	4	224	25.1	9.28
Q9CQD4	Chmp1b2	15	4	4	6	199	22.1	8.1

Q68FM6	Elfn2	4	4	4	6	823	90	7.52
Q9D9M2	Usp12	8	1	2	5	370	42.9	6.47
Q810B6	Ankfy1	5	5	5	6	1169	128.6	5.91
Q3UH68	Limch1	6	5	5	6	1057	118.1	5.48
Q9JHU9	Isyna1	6	4	4	5	557	60.9	6.42
Q61701	Elavl4	7	3	3	7	385	42.3	9.38
Q8BGQ1	Vipas39	10	3	3	3	491	56.6	7.17
O35609	Scamp3	8	2	2	5	349	38.4	7.64
O35449	Prrt1	8	2	2	4	306	31.4	7.65
Q8CCS6	Pabpn1	6	2	2	4	302	32.3	5.17
Q8CH72	Trim32	7	5	5	6	655	72	6.9
Q8CIP4	Mark4	7	4	5	5	752	82.6	9.67
Q80TI0	Gramd1b	6	5	5	6	738	85.3	6.14
Q3TTY5	Krt2	4	2	4	8	707	70.9	8.06
P55258	Rab8a	14	1	3	8	207	23.7	9.07
Q8R4C2	Rufy2	5	2	3	5	606	70	5.83
O55135	Eif6	19	3	3	4	245	26.5	4.74
Q8CGM1	Adgrb2	3	3	4	5	1561	169.8	7.36
P20065	Tmsb4x	40	2	4	12	50	5.7	5.06
O35345	Kpna6	9	3	3	3	536	59.9	4.94
Q91VR7	Map1lc3a	23	1	3	5	121	14.3	8.68
Q8CIB5	Fermt2	7	4	4	4	680	77.8	6.7
P12815	Pdcd6	24	3	3	3	191	21.9	5.4
Q8K1L5	Ppp1r11	12	2	2	5	131	14.5	6.09
Q7TNG5	Eml2	5	3	3	6	649	70.7	6.28
Q9DBL1	Acadsb	9	3	3	3	432	47.8	7.87
Q9DCB8	Isca2	16	2	2	3	154	16.7	5.6
Q61140	Bcar1	4	2	2	2	874	94.2	5.68
P06330		34	3	3	6	118	12.9	7.11
Q80TA6	Mtmr12	5	3	3	4	747	85.5	6.84
Q3TIR3	Ric8a	7	3	3	5	530	59.8	5.68
Q6ZWU9	Rps27	38	1	3	4	84	9.5	9.45
Q8BS95	Gpr89a	9	2	2	3	455	52.7	9.22
B2RQC6	Cad	2	4	4	7	2225	243.1	6.43
P16388	Kcna1	5	2	2	4	495	56.4	5.14
P40240	Cd9	8	3	3	10	226	25.2	7.23
Q8C3F2	Fam120c	4	3	4	5	1091	119.6	8.46
P62627	Dynlrb1	29	2	2	4	96	11	7.25
Q64152	Btf3	18	2	3	3	204	22	9.52
Q9JHG6	Rcan1	10	2	2	4	251	28.1	5.41
Q3UL36	Arglu1	11	4	4	6	271	32.9	10.36
Q8C166	Cpne1	7	3	4	5	536	58.8	5.66
Q80U95	Ube3c	2	2	2	6	1083	123.9	6.39

Q3TYX3	Smyd5	11	4	4	5	416	47.1	5.21
Q99KP3	Cryl1	14	4	4	7	319	35.2	5.86
Q9D0J4	Arl2	17	2	2	4	184	20.9	5.96
Q2L4X1	Bzw2	6	2	2	4	419	48	6.68
Q9WVL0	Gstz1	13	2	2	3	216	24.3	7.85
P28667	Marcksl1	19	3	3	5	200	20.2	4.61
Q9R0Q3	Tmed2	9	2	2	6	201	22.7	5.17
P18052	Ptpra	6	4	4	4	829	93.6	7.05
A2ADY9	Ddi2	13	4	4	6	399	44.6	5.05
Q99J39	Mlycd	6	3	3	4	492	54.7	8.94
Q9EPR4	Slc23a2	8	3	3	4	648	70	7.5
Q3UYV9	Ncbp1	6	4	4	4	790	91.9	6.64
P39688	Fyn	6	4	4	4	537	60.6	6.67
P01887	B2m	23	3	3	5	119	13.8	8.44
Q8CGF7	Tcerg1	5	5	5	7	1100	123.7	8.65
Q0KL01	Ubxn2b	10	3	3	5	331	37.4	5.94
Q9D7B6	Acad8	12	4	4	4	413	45	8.13
O08997	Atox1	59	3	3	4	68	7.3	6.51
Q01730	Rsu1	13	3	3	5	277	31.5	8.88
Q91ZW3	Smarca5	5	5	5	6	1051	121.6	8.15
P53811	Pitpnb	20	3	4	6	271	31.5	6.95
Q9QYE6	Golga5	5	4	4	6	729	82.3	6.23
P46978	Stt3a	5	3	3	4	705	80.5	8.1
Q7TPS5	C2cd5	4	3	3	3	1016	111.6	5.78
Q9CR98	Fam136a	19	3	3	5	138	15.7	7.61
Q61165	Slc9a1	5	3	3	4	820	91.4	7.12
Q8BNN1	Spata2l	13	4	4	4	426	46.7	5.2
Q61545	Ewsr1	5	3	3	5	655	68.4	9.33
Q08509	Eps8	6	4	4	5	821	91.7	7.65
Q920B9	Supt16h	3	3	3	5	1047	119.7	5.66
Q80TL1	Adcy2	3	4	4	6	1090	123.2	8.31
Q9Z1N5	Ddx39b	10	4	4	6	428	49	5.67
P26339	Chga	10	4	4	4	463	51.8	4.72
P63082	Atp6v0c	25	2	2	4	155	15.8	9.13
Q69ZF3	Gba2	6	5	5	6	918	103.2	5.59
Q8K0Z7	Taco1	9	2	2	5	294	32.3	8.12
Q62245	Sos1	4	5	5	7	1319	150.8	6.89
Q61234	Snta1	7	5	5	6	503	53.6	6.86
Q8JZZ7	Adgrl2	2	3	3	6	1487	166.5	6.71
Q9CX00	Ist1	12	4	4	5	362	39.4	5.44
Q9R0N0	Galk1	7	3	3	5	392	42.3	5.26
P97467	Pam	6	4	4	5	979	108.9	6.55
Q8CI75	Dis3l2	4	3	3	4	870	97.7	5.9
Q9Z172	Sumo3	23	2	2	5	110	12.4	5.96

Q99MB7	Rnf141	13	3	3	4	230	25.5	5.2
P29595	Nedd8	28	3	3	6	81	9	7.25
P84089	Erh	34	3	3	5	104	12.3	5.92
Q8VHQ9	Acot11	11	5	5	5	594	67.3	6.8
Q9JJ28	Flii	3	4	4	4	1271	144.7	6.06
O70378	Emc8	14	3	3	4	207	23.3	6.15
Q6NVF0	Ocrl	6	5	5	8	900	104.2	6.27
Q6NZC7	Sec23ip	4	4	4	4	998	110.7	5.94
Q9JIW9	Ralb	30	4	5	5	206	23.3	6.62
Q9DB29	Iah1	22	4	4	4	249	28	5.52
Q3UYC0	Ppm1h	8	3	3	3	513	56.3	6.73
Q9EQI8	Mrpl46	18	4	4	4	283	32.1	7.4
Q9DAR7	Dcps	17	4	4	5	338	39	6.48
Q9DCC4	Pycr3	13	3	3	4	274	28.7	7.27
Q922B1	Macrod1	9	3	3	5	323	35.3	8.85
O09164	Sod3	10	2	2	3	251	27.4	6.84
P31786	Dbi	32	2	2	5	87	10	8.82
Q61191	Hcfc1	2	4	4	6	2045	210.3	7.18
Q9DB73	Cyb5r1	13	3	3	5	305	34.1	8.87
P48771	Cox7a2	28	2	2	4	83	9.3	10.27
O08842	Gfra2	6	3	3	4	464	51.7	7.75
O70194	Eif3d	9	3	3	3	548	63.9	6.05
B1AXV0	Frrs1l	12	3	3	5	293	32.5	5.43
P09055	Itgb1	5	4	4	4	798	88.2	5.94
Q9ESZ8	Gtf2i	5	5	5	6	998	112.2	6.55
Q9CX56	Psmd8	7	3	3	5	353	39.9	9.58
Q9Z127	Slc7a5	6	2	2	6	512	55.8	7.9
Q8BFS6	Cpped1	15	4	4	5	312	35.2	5.34
Q9WUR2	Eci2	11	4	4	5	391	43.2	8.92
Q921T2	Tor1aip1	6	4	4	4	595	66.7	7.05
Q8C129	Lnpep	3	3	3	4	1025	117.2	5.96
Q6ZWY3	Rps27l	38	1	3	4	84	9.5	9.45
Q61772	Epha7	4	3	3	4	998	111.8	5.86
Q91XU0	Wrnip1	5	3	3	5	660	71.7	6.18
Q3UI43	Babam1	10	3	3	3	333	36.8	4.55
Q80WQ2	Vac14	6	4	4	6	782	88	6.13
Q70FJ1	Akap9	1	3	3	3	3797	435.9	5.03
Q8K2T1	Nmral1	11	3	3	3	309	34.4	6.86
A2A8L5	Ptprf	2	2	3	4	1898	211.4	6.65
P26049	Gabra3	7	3	4	7	492	55.4	8.94
Q6PGL7	Washc2	2	2	2	3	1334	145.2	4.77
Q9Z0E6	Gbp2	3	1	1	2	589	66.7	5.71
Q5NCE8	Mrs2	11	4	4	4	434	49.3	7.53

Q9CQE8	RTRAF	17	4	4	5	244	28.1	6.89
Q4VC33	Maea	11	3	3	3	396	45.3	8.69
Q9D967	Mdp1	15	2	2	4	164	18.6	6.8
P47746	Cnr1	8	4	4	5	473	52.8	8.27
P50136	Bckdha	11	4	4	4	442	50.3	8.06
Q9CYR6	Pgm3	8	5	5	6	542	59.4	6.2
Q9Z1D1	Eif3g	9	3	3	5	320	35.6	5.9
Q62165	Dag1	4	4	4	5	893	96.8	8.44
Q3TUH1	Tamm41	9	3	3	3	337	37.8	8.98
Q9CQV6	Map1lc3b	22	1	3	5	125	14.6	8.43
Q8R3Q6	Ccdc58	18	2	2	3	144	16.7	8.16
Q99P88	Nup155	3	3	3	3	1391	155	6.15
Q8JZL3	Thtpa	22	4	4	4	224	24.2	4.72
Q8BK30	Ndufv3	16	2	2	4	104	11.8	9.35
Q14CH7	Aars2	4	3	3	4	980	106.7	6.18
Q61831	Mapk10	5	2	3	6	464	52.5	6.96
Q99KC8	Vwa5a	4	3	3	5	793	87.1	6.58
Q8BFP9	Pdk1	6	3	3	4	434	49	8.19
Q3U7R1	Esyt1	4	4	4	5	1092	121.5	5.95
Q8BLV3	Slc9a7	4	3	3	3	726	80.2	6.4
Q9CQH7	Btf3l4	28	2	3	4	158	17.3	6.35
P63213	Gng2	42	2	2	5	71	7.8	7.99
Q80U87	Usp8	3	4	4	4	1080	122.5	8.47
P51125	Cast	6	4	4	5	788	84.9	5.52
O35887	Calu	17	4	4	4	315	37	4.67
Q9WU01	Khdrbs2	7	1	3	6	349	38.8	6.48
Q8K274	Fn3krp	13	4	4	6	309	34.4	7.87
Q9ET77	Jph3	6	3	3	3	744	81.2	9.22
Q3V038	Ttc9	10	2	2	3	219	24.3	8.85
Q9Z2A0	Pdpk1	8	4	4	4	559	63.7	7.37
Q9D0L4	Adck1	8	3	3	3	525	59.7	8.05
Q8R1I1	Uqcr10	38	2	2	6	64	7.4	9.19
Q8BYA0	Tbcd	2	2	2	4	1196	133.2	6.51
Q8BZJ7	Dcun1d2	10	1	2	3	259	30	5.59
Q5DU31	Ipcef1	10	3	3	4	406	45.8	6.73
Q8BK67	Rcc2	7	3	3	4	520	55.9	8.72
P28659	Celf1	6	3	4	6	486	52.1	8.46
Q80ZW2	Them6	20	4	4	4	207	23.8	9.11
Q9DCP2	Slc38a3	7	3	3	5	505	55.6	7.09
Q9CPS6	Hint3	11	2	2	6	165	18.8	6.33
Q8BTV2	Cpsf7	9	4	4	4	471	52	8
Q8JZV7	Amdhd2	10	3	3	3	409	43.5	6.23
P53657	Pklr	3	1	2	29	574	62.3	7.09
Q61327	Slc6a3	5	3	3	4	619	68.8	7.21

Q9R226	Khdrbs3	9	2	4	6	346	38.8	8.1
Q3TMH2	Scrn3	11	4	4	4	418	47.6	5.66
Q61074	Ppm1g	8	4	4	4	542	58.7	4.39
G3X9K3	Arfgef1	2	3	5	5	1846	208.4	5.86
Q9R1V4	Adam11	6	4	4	5	773	84.1	7.5
Q9QZB9	Dctn5	10	2	2	6	182	20.1	8.02
Q9D8S3	Arfgap3	9	4	4	5	523	57.4	8.47
Q80YR5	Safb2	5	1	4	7	991	111.8	6.38
Q8BUR4	Dock1	3	6	6	6	1865	214.9	7.62
Q62383	Supt6h	3	3	3	3	1726	199	4.93
Q3TDQ1	Stt3b	3	3	3	6	823	93.2	8.95
Q7TT45	Rragd	6	1	2	4	449	51.2	5.25
Q8BH79	Ano10	4	2	2	3	659	76.1	6.7
Q9EQG9	Cert	7	4	4	5	624	71.1	5.44
Q61334	Bcap29	13	3	3	4	240	27.9	9.72
Q60790	Rasa3	5	3	4	4	834	95.9	7.34
Q7M729	Scn4b	7	2	2	4	228	25.2	8.82
Q8K4Q0	Rptor	3	3	3	4	1335	149.4	6.87
A6H611	Mipep	5	3	3	4	711	80.8	7.15
Q9CZX0	Elp3	6	3	3	3	547	62.3	8.9
Q8BU14	Sec62	12	5	5	5	398	45.6	7.31
P62331	Arf6	21	2	2	4	175	20.1	8.95
P98084	Apba2	8	4	5	5	750	82.7	4.88
Q9DBC0	Selenoo	6	3	3	4	667	74.2	5.83
O55100	Syngr1	10	2	2	9	234	25.6	4.65
Q9CYG7	Tomm34	12	3	3	4	309	34.3	9.14
B2RXC1	Trappc11	5	4	4	4	1133	128.3	7.58
Q8BT60	Cpne3	7	2	4	5	533	59.5	5.78
P59808	Sash1	3	3	3	3	1230	135.5	6.2
Q8VEH8	Erlec1	8	3	3	5	483	54.9	6.25
Q8K1X1	Wdr11	4	4	4	4	1223	135.9	7.11
P60761	Nrgn	32	3	3	6	78	7.5	7.05
Q9D3A9	Ttyh1	5	2	2	5	450	49	5.03
Q8BY89	Slc44a2	5	3	3	4	706	80.1	8.79
Q9JIZ9	Plscr3	7	1	1	2	296	31.8	6.38
Q9CYN9	Atp6ap2	9	3	3	5	350	39.1	5.54
Q3TLS3	Gdpgp1	15	4	4	5	386	42.5	6.19
P35283	Rab12	14	4	4	5	243	27.3	8.41
P39038	Cdh4	3	1	3	7	913	100	4.81
O54828	Rgs9	5	4	4	6	675	76.9	9.33
Q91YL2	Rnf126	8	2	2	5	313	34.1	5.17
O08582	Gtpbp1	5	3	3	6	668	72.3	8.29
Q9D6K5	Synj2bp	31	4	4	4	145	15.8	6.3
Q6P9Q6	Fkbp15	2	3	3	4	1216	132.9	5.07

Q61425	Hadh	10	3	3	4	314	34.4	8.65
Q8VEJ9	Vps4a	9	3	4	7	437	48.9	7.8
Q8BYL4	Yars2	7	4	4	4	472	52.6	9.16
Q80Y24	Prickle2	4	4	4	5	845	95.7	7.27
Q91Z38	Ttc1	8	2	2	4	292	33.2	5.01
Q99LD9	Eif2b2	7	2	2	5	351	38.9	6.24
Q922R1		8	3	3	5	422	47.4	7.74
Q8K4Z3	Naxe	11	3	3	5	282	31	7.69
Q9JMK2	Csnk1e	12	3	4	5	416	47.3	9.66
Q8K221	Arfip2	8	2	2	4	341	37.7	5.87
Q8VCC9	Spon1	4	3	3	3	807	90.8	6.02
Q9ES00	Ube4b	3	4	4	5	1173	133.2	6.07
P51437	Camp	16	2	2	4	172	19.4	8.68
Q99KF1	Tmed9	11	2	3	8	235	27.1	8.41
P28063	Psmb8	12	3	3	4	276	30.2	6.68
Q91VW5	Golga4	2	5	5	5	2238	257.4	5.36
Q99J08	Sec14l2	11	3	3	3	403	46.3	7.11
P01899	H2-D1	10	2	3	4	362	40.8	6.73
Q9CYZ2	Tpd52l2	16	3	3	3	220	24	6.15
P41105	Rpl28	17	3	3	5	137	15.7	12.02
Q9QZI8	Serinc1	8	3	3	4	453	50.5	6.28
Q80UK0	Sestd1	4	3	3	4	696	79.3	5.1
Q9D1H7	Get4	9	3	3	5	327	36.5	5.41
O88602	Cacng2	12	3	3	3	323	35.9	8.98
Q91WT9	Cbs	8	3	3	6	561	61.5	6.48
P52479	Usp10	5	4	4	4	792	87	5.17
Q8CH18	Ccar1	2	3	3	6	1146	132	5.76
P59759	Mrtfb	2	3	3	7	1080	117.5	6.16
Q8BX94	Osbpl2	8	2	2	4	484	55.3	6.2
P59672	Anks1a	2	1	2	5	1150	125.2	6.05
Q9Z0G0	Gipc1	13	3	3	4	333	36.1	5.91
Q3TLI0	Trappc10	3	4	4	4	1259	141.5	5.96
Q6NVE9	Pptc7	16	4	4	6	310	33	5.27
Q921S7	Mrpl37	10	3	3	3	423	48.3	8.84
Q8BFY9	Tnpo1	3	2	2	3	898	102.3	4.98
Q99JT1	Gatb	7	3	3	3	557	62.1	8.54
Q3UGX3	Nat8l	9	2	2	3	299	32.8	8.53
Q6PD26	Pigs	3	1	1	2	555	61.7	6.93
P48428	Tbca	25	3	3	4	108	12.8	5.27
P61620	Sec61a1	8	4	4	4	476	52.2	8.06
Q91YP0	L2hgdh	7	3	3	4	464	50.9	8.29
Q6PD19	Armh3	4	3	3	3	689	78.6	6.6
Q8BL97	Srsf7	13	4	4	4	267	30.8	11.9

O70503	Hsd17b12	7	3	3	5	312	34.7	9.52
Q8BR63	Fam177a1	15	2	2	3	207	23.6	4.59
B1AY13	Usp24	2	4	4	5	2617	293.8	6.19
Q8C1Y8	Ccz1	7	3	3	4	480	55.5	6.02
Q8BIG7	Comtd1	7	2	2	4	262	28.9	8.32
Q9R1P3	Psmb2	12	2	2	3	201	22.9	7.02
O54901	Cd200	16	4	4	7	278	31.2	8.79
B1AVZ0	Uprt	11	3	3	4	310	34.3	6.23
Q9DBR3	Armc8	7	4	4	4	673	75.3	6.73
Q91V76		13	5	5	7	315	35	6.29
Q8C729	Fam126b	7	4	4	4	530	58.6	7.72
Q8R191	Syngr3	12	2	2	7	229	24.5	8.18
Q8C9H6	Strip2	5	2	3	4	844	96.2	5.77
Q91Y97	Aldob	5	1	2	4	364	39.5	8.27
P63089	Ptn	10	2	2	4	168	18.9	9.6
Q6PDI6	Mindy2	7	3	3	3	601	65.6	4.63
Q9CRA9	Fgfr1op2	12	3	3	5	253	29.4	5.83
O35682	Myadm	13	3	3	5	320	35.3	8.31
Q8VBT9	Aspscr1	8	5	5	5	550	59.8	6.96
Q9CY18	Snx7	9	3	3	3	387	45	5.07
Q91W39	Ncoa5	7	4	4	4	579	65.3	9.82
Q91VZ6	Smap1	10	3	3	3	440	47.6	8.51
Q62523	Zyx	7	3	3	3	564	60.5	6.4
Q9D6K8	Fundc2	18	3	3	4	151	16.6	9.7
Q8VBV7	Cops8	11	2	2	4	209	23.2	5.2
Q9R1L5	Mast1	2	3	4	4	1570	170.9	8.44
O88746	Tom1	7	3	3	4	492	54.3	4.94
Q99JB8	Pacsin3	4	2	2	3	424	48.6	6.1
Q9QYI4	Dnajb12	5	2	2	4	376	42	8.51
Q7TSV4	Pgm2	7	3	4	5	620	68.7	6.14
Q8K3C3	Lzic	16	2	2	3	190	21.5	4.96
Q99K46	Usp11	3	2	2	5	921	105.3	4.96
Q811I0	Atpaf1	14	4	4	4	324	36.3	8.19
P56375	Acyp2	33	4	4	5	106	11.9	9.26
Q8K268	Abcf3	6	4	4	4	709	79.8	6.16
Q4VA53	Pds5b	3	4	5	5	1446	164.3	8.5
Q68ED7	Crtc1	6	3	3	5	630	66.9	6.05
Q69ZS7	Hbs1l	5	2	2	4	682	75.1	6.46
P54729	Nub1	6	4	4	4	614	70.3	5.88
O88986	Gcat	10	3	3	4	416	44.9	7.33
Q9QYA2	Tomm40	11	3	3	3	361	37.9	7.74
P49710	Hcls1	7	4	4	5	486	54.2	4.84
Q8K224	Nat10	4	3	3	3	1024	115.3	8.32

Q9DCN2	Cyb5r3	7	2	2	6	301	34.1	8.38
Q9JKD3	Scamp5	9	2	2	6	235	26.1	8.54
Q9CQY6	Uqcc2	38	3	3	4	136	16.3	9.19
P70429	Evl	11	4	4	4	414	44.3	8.85
P62500	Tsc22d1	3	2	3	6	1077	109.7	5.83
Q9CZM2	Rpl15	15	3	3	4	204	24.1	11.6<br>2
P53702	Hccs	13	3	3	3	272	31	7.12
Q8R1F6	Hid1	6	3	3	6	788	88.7	5.94
Q60770	Stxbp3	7	4	4	4	592	67.9	8.02
Q9DBC3	Cmtr1	3	3	3	5	837	95.6	7.27
P62267	Rps23	8	2	2	3	143	15.8	10.4<br>9
Q0GA42	Cnnm1	3	3	3	4	951	103.9	6.51
Q61609	Slc20a1	4	4	4	5	681	74.1	6.89
P97461	Rps5	13	4	4	9	204	22.9	9.72
Q60673	Ptprn	4	2	3	3	979	106	7.09
Q3UHK6	Tenm4	1	2	3	4	2771	308.2	6.57
Q91X78	Erlin1	8	2	3	5	348	39.2	7.21
Q3TLH4	Prrc2c	1	3	4	5	2846	310.7	9.1
Q91YR1	Twf1	10	3	4	6	350	40.1	6.67
Q9D1J3	Sarnp	18	3	3	4	210	23.5	6.65
Q8C4G9	Adgra1	7	4	4	6	578	63.5	7.43
Q9WVG6	Carm1	5	3	3	5	608	65.8	6.77
P01631		12	1	1	2	113	12.3	8.88
Q8VEA4	Chchd4	27	2	2	4	139	15.5	4.32
Q3TW96	Uap1l1	6	3	3	4	507	56.6	5.43
Q9QXG4	Acss2	5	3	3	5	701	78.8	6.64
Q08093	Cnn2	7	1	2	4	305	33.1	7.62
Q8BWM0	Ptges2	7	3	3	5	384	43.3	9
Q9QYS9	Qki	10	3	3	3	341	37.6	8.56
Q9CZL5	Pcbd2	18	2	3	4	136	14.8	9.16
Q9WV85	Nme3	24	4	4	5	169	19.1	6.68
Q9ER73	Elp4	8	3	3	4	422	46.3	8.78
Q99NF2	Nsmf	7	3	3	6	532	60.3	8.98
Q924S7	Spred2	13	3	4	4	410	46.8	6.81
Q8BH86	Dglucy	6	3	3	3	617	66.3	7.09
Q3KNM2	March5	12	3	3	3	278	31.2	8.7
Q6P5E8	Dgkq	3	2	2	5	934	102.2	7.52
Q9D772	Fam219a	13	2	2	3	157	17.5	4.68
Q9D6Y9	Gbe1	6	3	3	3	702	80.3	6.43
P70408	Cdh10	6	5	5	6	788	88.3	4.94
Q8BWQ6	Vps35l	4	4	4	4	963	109	7.31
O55128	Sap18	24	4	4	6	153	17.6	9.35

Q920M7	Syt17	8	4	4	4	470	53.3	7.15
O55013	Trappc3	21	4	4	5	180	20.3	4.96
Q80TN5	Zdhhc17	4	3	3	4	632	72.6	7.39
O08715	Akap1	6	3	3	3	857	92.1	5.02
Q8K2I1	Fntb	6	2	2	3	437	48.8	5.8
Q9CQX2	Cyb5b	17	2	2	3	146	16.3	4.89
O88531	Ppt1	12	3	3	3	306	34.5	8
P56371	Rab4a	19	2	3	4	218	24.4	6.07
P83882	Rpl36a	18	3	3	6	106	12.4	10.5<br>8
Q6P9S0	Mtss2	6	3	3	3	715	76.8	6.99
Q99LG2	Tnpo2	3	2	2	3	887	100.4	4.98
Q9D8B4	Ndufa11	24	2	2	2	141	15	8.35
Q8BTZ5	Ankrd46	12	2	2	3	228	25.2	5.73
Q6DIC0	Smarca2	3	3	4	4	1577	180.1	7.2
Q99MR0	Actl6b	7	1	3	5	426	46.9	5.71
E9Q5C9	Nolc1	5	3	3	4	702	73.7	9.36
Q8C0L9	Gpcpd1	5	3	3	3	675	76.5	5.58
P11087	Col1a1	3	4	4	6	1453	137.9	5.85
Q03059	Chat	5	3	3	3	641	71.8	7.74
Q9JJR8	Tmem9b	20	4	4	5	199	22.6	8.18
P62911	Rpl32	17	2	2	4	135	15.9	11.3<br>3
P53612	Rabggtb	6	2	2	5	339	37.8	5.16
Q8BSZ2	Ap3s2	11	2	2	4	193	22	5.22
Q3UJB9	Edc4	3	3	3	3	1406	152.4	5.78
P59648	Fxyd7	25	2	2	7	80	8.5	7.84
Q7TQK5	Ccdc93	10	5	5	6	629	72.6	8.29
P98195	Atp9b	3	2	3	4	1146	128.9	7.64
P60840	Ensa	29	2	3	4	121	13.3	7.24
O88745	Scrg1	16	2	2	6	98	11.2	7.91
Q9WUB0	Rbck1	5	3	3	4	508	57.5	6.19
Q8CC21	Ttc19	7	3	3	5	365	41.2	6.21
Q9DCB1	Hmgn3	18	3	3	4	99	10.8	9.7
P23591	Tsta3	9	2	2	3	321	35.9	6.74
Q91ZM2	Sh2b1	6	4	4	5	756	79.6	5.25
Q7TNF0	Doc2a	6	1	2	4	405	44.6	7.12
Q9Z2D0	Mtmr9	5	3	3	3	545	62.9	6.62
P21661	Pcsk2	4	2	2	3	637	70.7	6.42
P42225	Stat1	5	3	3	3	749	87.1	5.58
Q7TPV4	Mybbp1a	3	3	3	3	1344	151.9	8.95
Q8R2U4	Ntmt1	15	2	2	3	223	25.4	6.9
Q8VBX6	Mpdz	2	3	3	3	2055	218.6	5.02
P51791	Clcn3	4	3	3	4	818	90.8	6.28

P63143	Kcnab1	9	1	2	2	401	44.7	9.32
Q80WM4	Hapln4	9	3	3	3	400	42.8	8.85
Q8CGF5	Tlcd4	9	2	2	2	276	31.2	8.57
Q91Z69	Srgap1	4	2	4	4	1062	121.4	6.74
Q3TJZ6	Fam98a	6	2	2	3	515	55	8.95
Q9Z2Q5	Mrpl40	18	3	3	3	206	24.3	9.47
Q50H33	Kctd8	6	2	3	3	476	52.7	8.38
Q7M6Z0	Rtn4rl2	6	2	2	3	420	46	7.65
Q8BIF0	Cd99l2	8	1	1	4	237	25.4	4.86
Q9D358	Acp1	18	2	2	4	158	18.2	6.74
Q9QXJ1	Apbb1	6	4	4	4	710	77.3	5
P55144	Tyro3	4	2	2	3	880	96.1	5.92
Q9EQC5	Scyl1	5	4	4	6	806	89.1	6.44
Q9DCF9	Ssr3	8	1	1	3	185	21.1	9.61
O08579	Emd	13	3	3	3	259	29.4	5.01
Q9CQV1	Pam16	37	3	3	3	125	13.8	9.64
P46737	Brcc3	7	2	2	4	291	33.3	5.83
Q9Z2N8	Actl6a	7	1	3	5	429	47.4	5.6
P62849	Rps24	25	3	3	3	133	15.4	10.78
Q9D7N3	Mrps9	9	3	3	3	390	44.9	8.81
Q8CJG1	Ago1	3	1	2	3	857	97.2	9.16
Q8CCJ4	Amer2	6	3	3	3	672	69.9	6.64
P54869	Hmgcs2	4	1	2	4	508	56.8	8.41
P02463	Col4a1	2	3	3	4	1669	160.6	8.24
P70318	Tial1	7	3	3	3	392	43.4	7.99
Q8R050	Gspt1	5	4	4	5	636	68.6	5.21
P60755	Mdga2	4	3	3	3	949	106.6	7.02
P43406	Itgav	3	3	3	5	1044	115.3	5.63
P35123	Usp4	5	5	5	6	962	108.3	5.64
Q9JL56	Gde1	12	4	4	4	331	37.6	6.9
O09114	Ptgds	9	1	1	5	189	21.1	8.25
Q8C031	Lrrc4c	4	2	3	4	640	71.9	7.15
Q922Q9	Chid1	6	2	2	3	393	44.9	7.97
Q9D328	Tmem35a	13	2	2	5	167	18.5	9.99
Q9QZ23	Nfu1	14	3	3	4	255	28.5	5.03
P63248	Pkia	42	2	2	3	76	8	4.54
Q80TL7	Mon2	1	2	2	3	1715	189	6.13
A2AQ19	Rtf1	5	3	3	3	715	80.7	8.16
P54116	Stom	12	3	3	3	284	31.4	6.93
O08807	Prdx4	9	2	3	6	274	31	7.15
Q8CES0	Naa30	10	3	3	3	364	39.4	5.64
P03987		5	2	2	4	398	43.9	7.14
Q9D8N2	Dennd10	10	2	2	3	357	40.4	6.47

P57784	Snrpa1	15	3	3	3	255	28.3	8.62
Q8VD63	Tspyl4	8	3	3	3	406	44.8	6.99
Q8CH77	Nav1	2	3	3	3	1875	202.2	8.06
Q78JW9	Ubfd1	10	3	3	7	368	40.1	8.85
Q8CAK3	Shfl	6	2	2	6	290	33	7.28
Q9QZH6	Ecsit	6	2	2	3	435	49.8	6.6
E9QAT4	Sec16a	2	3	3	3	2357	254	5.81
Q3V3Q7	Pacs2	5	3	3	4	862	94.9	6.51
P04186	Cfb	3	2	2	3	761	85	7.37
P61759	Vbp1	12	3	3	5	196	22.4	6.28
Q9CXI5	Manf	12	2	2	3	179	20.4	8.07
Q9JIG7	Ccdc22	4	2	2	4	627	70.8	6.01
Q6PNC0	Dmxl1	1	3	4	4	3013	335.8	6.42
Q8K245	Uvrag	7	5	5	5	698	77.5	7.97
Q6ZWY8	Tmsb10	34	1	3	10	44	5	5.36
P18826	Phka1	3	3	3	3	1241	138.7	5.86
Q922U1	Prpf3	5	4	4	4	683	77.4	9.5
P35288	Rab23	15	3	3	3	237	26.7	6.79
Q9CQZ6	Ndufb3	17	2	2	9	104	11.7	9.04
Q80U56	Avl9	5	3	3	3	649	72.1	6.14
P56382	Atp5f1e	42	3	3	8	52	5.8	10.0<br>1
P22005	Penk	13	3	3	4	268	31	5.68
Q8BM13	Olfm2	8	3	3	3	448	50.7	8.51
Q921C1	Gjc3	5	2	2	2	269	30.3	8.37
Q9WTK7	Stk11	6	2	2	2	436	49.2	6.84
Q8BZA9	Tigar	15	2	2	2	269	29.2	8.18
Q9QYE9	Plekhb1	12	3	3	5	243	27.3	6.98
Q99NH0	Ankrd17	1	4	4	4	2603	274	6.52
B1AR13	Cisd3	16	3	3	5	137	15.7	9.79
Q9QZ73	Dcun1d1	10	1	2	3	259	30.1	5.34
Q9QX60	Dguok	14	2	2	2	277	32.3	7.66
P97927	Lama4	3	4	4	4	1816	201.7	6.21
Q01279	Egfr	2	2	2	3	1210	134.8	6.86
Q8VEL9	Rem2	12	3	3	4	341	37.3	7.39
P97820	Map4k4	3	2	4	4	1233	140.5	7.47
Q8BVG4	Dpp9	4	3	3	3	862	97.9	6.65
Q3U214	Mast3	2	2	3	3	1321	144.1	8.44
P62960	Ybx1	11	2	2	3	322	35.7	9.88
Q9JJ59	Abcb9	5	2	2	3	762	83.9	7.62
Q9Z2Z6	Slc25a20	10	3	3	3	301	33	9.11
Q8K070	Samd14	9	3	3	3	417	45.1	9.47
Q9CXW2	Mrps22	6	2	2	5	359	41.2	8.56
P63030	Mpc1	21	3	3	5	109	12.4	9.61

Q62407	Speg	1	2	2	3	3262	354.1	8.21
O55222	Ilk	7	3	3	3	452	51.3	8.07
P48759	Ptx3	6	2	2	4	381	41.8	5.38
Q9QXY7	Xk	4	1	1	2	446	51.1	8.19
P61924	Copz1	12	2	2	3	177	20.2	4.81
Q3UE37	Ube2z	10	4	4	4	356	38.3	5.62
Q62426	Cstb	28	4	4	5	98	11	7.39
O88384	Vti1b	9	2	2	3	232	26.7	8.79
Q3TZZ7	Esyt2	6	4	4	5	845	94.1	7.75
P49586	Pcyt1a	5	2	2	4	367	41.6	7.03
Q3U7U3	Fbxo7	7	2	2	3	523	57.6	5.83
Q9CRC8	Lrrc40	5	3	3	3	602	68	6.89
Q9JHL1	Slc9a3r2	9	3	3	4	337	37.4	7.59
O08967	Cyth3	6	1	2	3	399	46.3	5.54
Q8BTG3	Tcp11l1	7	3	3	3	509	56.3	5.5
Q9JKF6	Nectin1	6	2	2	3	515	57	6.35
Q9JIK5	Ddx21	3	3	3	3	851	93.5	9.11
Q8BPB0	Mob1b	11	2	2	3	216	25.1	6.73
Q9DB70	Fundc1	19	2	2	5	155	17.1	8.63
O35326	Srsf5	12	3	3	6	269	30.9	11.56
Q99KR7	Ppif	11	2	3	5	206	21.7	9.16
Q9DCZ1	Gmpr	5	1	1	2	345	37.5	7.09
Q64455	Ptprj	2	2	2	3	1238	136.7	5.57
O55201	Supt5h	3	3	3	3	1082	120.6	5.05
Q8BGB7	Enoph1	7	2	2	3	257	28.6	4.92
P59108	Cpne2	4	1	2	3	548	61	5.96
Q9WTP6	Ak2	13	3	3	4	239	26.5	7.39
Q80V42	Cpm	7	3	3	4	443	50.5	7.78
Q52KR3	Prune2	1	3	3	4	3084	339.3	4.46
P53395	Dbt	7	3	3	4	482	53.2	8.6
Q8C547	Heatr5b	1	4	4	4	2070	224.2	7.14
Q99JT2	Stk26	9	1	3	3	416	46.6	5.24
Q9R0H0	Acox1	6	3	3	3	661	74.6	8.48
P52623	Uck1	12	3	3	3	277	31	7.88
Q9WTU6	Mapk9	6	2	3	5	423	48.2	5.97
Q9CXW4	Rpl11	17	3	3	3	178	20.2	9.6
Q61703	Itih2	4	3	3	3	946	105.9	7.27
Q8R1S0	Coq6	6	3	3	3	476	51.4	7.17
P01644		31	2	3	4	108	11.9	7.97
Q9Z0Y1	Dctn3	13	3	3	6	186	21	6.06
Q8C6B2	Rtkn	7	3	3	3	564	63	6.67
Q3TC93	Hs1bp3	11	2	2	2	395	43.7	4.98
Q8BH61	F13a1	6	3	3	3	732	83.2	5.92

Q3TL44	Nlrx1	3	3	3	3	975	107.8	7.37
Q9CQ48	Nudcd2	18	2	2	2	157	17.6	5.07
Q8BFZ2	Plppr1	5	2	2	4	325	35.9	6.83
A8Y5H7	Sec14l1	3	2	2	2	715	81.2	6.34
P01898	H2-Q10	6	1	2	3	325	37.2	5.25
Q0VGB7	Ppp4r2	7	2	2	3	417	46.5	4.56
Q9JIA7	Sphk2	6	2	2	2	617	65.6	6.57
E9Q4N7	Arid1b	2	4	4	4	2244	237.6	6.73
Q505D7	Opa3	11	2	2	4	179	20.1	8.34
Q99PU8	Dhx30	3	4	4	4	1217	136.6	8.75
Q6P069	Sri	11	2	2	4	198	21.6	5.59
O88597	Becn1	6	3	3	3	448	51.6	4.93
Q3UEZ8	Slc10a4	3	1	1	3	437	46.6	5.01
Q9CPQ1	Cox6c	29	3	3	5	76	8.5	10.14
Q8VDN4	Ccdc92	8	3	3	3	314	35.2	9.38
Q8CDA1	Inpp5f	3	3	3	3	1132	127.5	7.11
O55057	Pde6d	16	2	2	4	150	17.3	5.67
Q8BUY5	Timmdc1	6	2	2	3	285	31.8	9.29
Q60823	Akt2	7	1	2	2	481	55.7	6.37
P35285	Rab22a	11	2	2	2	194	21.8	8.15
P58854	Tubgcp3	4	3	3	3	905	103.4	8.32
O55033	Nck2	6	2	2	3	380	42.9	6.95
P26369	U2af2	6	2	2	3	475	53.5	9.09
Q04519	Smpd1	4	3	3	4	627	69.9	7.2
Q9DAU1	Cnpy3	13	3	3	4	276	30.5	5.62
Q3TCJ1	Abraxas2	8	3	3	4	415	46.9	6.18
P97823	Lypla1	7	2	2	6	230	24.7	6.62
Q6QWF9	Camk2n1	35	2	2	2	78	8.5	5.45
Q9QY81	Nup210	2	2	2	2	1886	204	6.65
P19258	Mpv17	24	3	3	3	176	19.7	9.44
Q8BGC4	Zadh2	11	4	4	4	377	40.5	7.42
Q9Z315	Sart1	4	3	3	4	806	90.8	5.82
Q8BYH7	Tbc1d17	4	3	3	3	645	72.8	5.25
Q9D773	Mrpl2	10	3	3	5	306	33.3	11.06
Q63850	Nup62	6	2	2	3	526	53.2	5.31
P52189	Kcnj4	5	2	2	3	445	49.9	6.21
Q64378	Fkbp5	9	4	4	4	456	50.9	7.8
D3Z7H4	Gsg1l	11	3	3	4	322	35.9	7.01
P41317	Mbl2	11	2	2	3	244	25.9	5.06
Q80TE0	Rpap1	2	2	2	2	1409	155.2	6.65
Q8VCL2	Sco2	10	2	2	2	255	28.9	8.29
Q8R104	Sirt3	7	2	2	3	334	36.6	7.44

Q99J36	Thumpd1	9	4	4	6	350	38.9	6.07
Q9EPC1	Parva	8	2	2	3	372	42.3	5.95
Q60960	Kpna1	7	3	3	3	538	60.1	5.01
Q9QYY0	Gab1	4	2	2	3	695	76.8	5.67
Q9JLB2	Mpp5	3	2	2	3	675	77.2	6.09
P09813	Apoa2	19	2	2	3	102	11.3	7.18
Q6PFD9	Nup98	2	3	3	3	1816	197.1	6.18
Q8BND5	Qsox1	5	4	4	4	748	82.7	7.17
Q8BK03	Miga2	6	3	3	3	593	65.5	5.41
Q80UM7	Mogs	3	3	3	3	834	91.8	9
Q8BK08	Tmem11	17	4	4	6	190	21.3	7.36
Q9DC63	Fbxo3	8	2	2	2	480	55.2	5.02
Q66L44	Cbarp	1	1	1	6	698	74.1	6.33
Q9D662	Sec23b	4	1	2	2	767	86.4	6.96
Q8C0P5	Coro2a	6	4	4	5	524	59.5	7.71
O35239	Ptpn9	7	4	4	5	593	67.9	8.1
Q8BHF7	Pgs1	5	2	2	2	553	62.5	8.9
O70152	Dpm1	10	2	2	3	260	29.2	9.51
Q3U186	Rars2	5	3	3	3	578	65.3	8.02
Q9CPQ3	Tomm22	20	2	2	3	142	15.5	4.34
Q61097	Ksr1	3	3	3	3	873	96.7	8.54
Q9CQ86	Mien1	32	4	4	5	115	12.3	4.51
Q8K4R4	Pitpnc1	12	3	3	3	332	38.4	6.32
Q9WTI7	Myo1c	3	3	3	3	1063	121.9	9.35
Q91YJ3	Thyn1	16	4	4	5	226	26.2	9.11
Q61129	Cfi	5	4	4	5	603	67.2	7.46
Q61249	Igbp1	9	2	2	3	340	38.9	6.18
Q60610	Tiam1	2	2	2	3	1591	177.4	6.67
Q4PJX1	Odr4	5	2	2	3	447	50	6.14
Q9D0A3	Arpin	15	4	4	5	226	25.2	5.19
Q91YW3	Dnajc3	9	4	4	4	504	57.4	5.85
S4R1M9	Osbpl10	3	2	2	2	766	83.8	8.28
Q8VDG5	Ppcs	8	2	2	3	311	33.8	6.55
Q8K2Y9	Ccm2	9	2	2	2	453	49.9	5.62
Q6P4T0	Atg2a	2	4	4	4	1914	210.8	6.06
Q8R502	Lrrc8c	5	3	3	3	803	92.3	7.81
Q60854	Serpinb6	9	3	3	3	378	42.6	5.74
Q9WU40	Lemd3	4	2	2	2	921	100.2	7.55
Q6P8I4	Pcnp	17	2	2	2	178	19	7.49
Q923Z0	Gprc5b	3	1	1	2	410	45.9	8.38
Q924S8	Spred1	7	2	3	3	444	50.6	6.47
Q8K2L8	Trappc12	5	3	3	3	797	87.6	4.81
Q8R2U0	Seh1l	8	3	3	3	360	39.7	8.05
Q07813	Bax	7	1	1	3	192	21.4	4.98

O89114	Dnajb5	12	3	3	3	348	39.1	9.04
O35474	Edil3	6	3	3	4	480	53.7	7.58
P56376	Acyp1	21	2	2	5	99	11.2	9.04
P46467	Vps4b	4	1	2	5	444	49.4	7.11
Q9R190	Mta2	5	4	4	4	668	75	9.67
Q8BGT5	Gpt2	5	3	3	4	522	57.9	8
O88587	Comt	6	2	2	4	265	29.5	5.83
Q8VEG4	Exd2	4	2	2	3	650	74.3	7.78
Q9D8X1	Cutc	13	2	2	2	272	29	7.34
Q9R1C7	Prpf40a	3	2	2	2	953	108.4	7.69
Q8VHE0	Sec63	1	1	1	4	760	87.8	5.38
Q60648	Gm2a	11	1	1	2	193	20.8	5.9
O35075	Vps26c	10	2	2	3	297	32.9	7.68
O70310	Nmt1	4	2	2	4	496	56.9	8
Q62083	Pick1	8	3	3	3	416	46.6	5.52
P61255	Rpl26	12	3	3	5	145	17.2	10.55
Q924A2	Cic	1	3	3	3	2510	258	8.02
Q5HZI9	Slc25a51	8	3	3	3	298	33.7	9.66
Q3UFY7	Nt5c3b	5	1	1	2	300	34.4	6.24
E9PZ19	Igsf9b	3	3	3	3	1328	144.9	6.7
Q9D0W5	Ppil1	23	3	3	3	166	18.2	7.99
Q91YM4	Tbrg4	3	2	2	4	630	71.5	8.34
Q91WK0	Lrrfip2	7	2	2	3	415	47.1	5.68
Q8VE62	Paip1	8	2	2	2	400	45.7	4.55
Q921W0	Chmp1a	12	3	3	5	196	21.6	8.06
Q9D8B3	Chmp4b	11	2	2	3	224	24.9	4.82
Q99JX7	Nxf1	6	3	3	3	618	70.3	8.73
Q8BWU1	Sprn	17	1	1	1	147	14.6	10.35
Q52KF3	Spire1	6	4	4	5	598	68.4	9.01
Q6GQT1	A2m	2	2	3	4	1474	164.2	6.61
O88848	Arl6	18	3	3	3	186	20.9	8.25
Q8K1J6	Trnt1	5	3	3	4	434	49.9	8.56
Q8BXZ1	Tmx3	9	2	2	2	456	51.8	5.16
Q923G2	Polr2h	15	2	2	2	150	17.1	4.68
Q9CQX8	Mrps36	21	1	1	4	102	11.1	9.99
P58044	Idi1	13	2	2	3	227	26.3	6.16
Q6ZWZ2	Ube2r2	8	2	2	4	238	27.1	4.42
Q9D0B6	Pbdc1	17	3	3	3	198	22.2	4.55
Q9WV32	Arpc1b	9	2	2	2	372	41	8.35
P55096	Abcd3	5	2	2	2	659	75.4	9.26
P70205	Adcyap1r1	5	3	3	4	496	56.6	6.52

O55003	Bnip3	10	2	2	3	187	21	7.14
Q9Z0H3	Smarcb1	8	2	2	3	385	44.1	6.23
P0C0A3	Chmp6	14	2	2	3	200	23.4	5.44
Q9D6E4	Ttc9b	13	2	2	2	239	25.9	9.48
P12961	Scg5	17	2	2	2	212	23.9	5.81
Q9CX80	Cygb	10	2	2	3	190	21.5	6.8
Q8BXT1	Rgs8	16	3	3	3	180	21	9.26
Q8VE22	Mrps23	14	2	2	3	177	20.3	8.59
Q3UHD2	Gfod1	8	3	3	3	390	43.3	5.92
P01670		23	1	2	2	111	12	8
P70698	Ctps1	6	2	2	3	591	66.6	6.58
Q8BTY2	Slc4a7	3	1	3	3	1034	116.4	6.24
G5E870	Trip12	2	3	3	4	2025	224	8.35
Q9R0I7	Ylpm1	1	1	2	8	1386	155	6.65
Q8K019	Bclaf1	3	3	3	3	919	105.9	9.99
Q9DCS2	Mettl26	12	2	2	3	204	22.7	6.52
Q69Z99	Znf512	4	1	1	2	562	63.9	9.51
Q8K382	Dennd1a	4	3	3	3	1016	111.5	6.79
Q8BGC0	Htatsf1	4	4	4	5	757	86.2	4.4
Q64337	Sqstm1	9	2	2	3	442	48.1	5.21
Q9CQI7	Snrpb2	8	1	2	3	225	25.3	9.72
Q6W8Q3	Pcp4l1	49	2	2	3	68	7.5	5.52
Q99P31	Hspbp1	10	2	2	2	357	39.1	5.36
Q8VCR7	Abhd14b	14	3	3	3	210	22.4	6.27
Q5M8N4	Sdr39u1	9	3	3	3	293	31.4	9.31
O09126	Sema4d	4	3	3	3	861	95.6	7.81
Q8CE50	Snx30	2	1	1	3	437	49.5	5.35
Q9D1Q4	Dpm3	24	2	2	2	92	10.1	7.08
Q8BXQ2	Pigt	5	3	3	3	582	65.7	8.4
P70345	Bcl2l2	14	3	3	3	193	20.8	5.58
P63056	Olfm3	6	2	2	2	478	54.9	8.15
P35821	Ptpn1	4	2	2	3	432	49.6	6.16
Q8BGB5	Limd2	13	2	2	3	128	14.2	9.04
Q9Z351	Kcnq2	5	3	3	4	759	84.4	9.69
Q8BTF8	Ralyl	9	2	3	3	293	32.4	7.93
Q99JP0	Map4k3	2	2	2	4	894	101.1	7.64
Q3ULF4	Spg7	3	3	3	3	781	85.9	9.04
Q8VHK1	Caskin2	3	1	3	4	1201	126.7	7.39
Q9R020	Zranb2	9	2	2	2	330	37.3	9.89
Q60936	Coq8a	3	2	2	3	645	71.7	6.54
Q9Z2D1	Mtmr2	4	3	3	4	643	73.2	7.25
P63280	Ube2i	22	3	3	3	158	18	8.66
Q80Y98	Ddhd2	6	3	3	3	699	79.5	5.31
Q68ED2	Grm7	5	3	3	3	915	102.2	7.88

Q9JLT4	Txnrd2	4	2	2	3	524	56.6	8.46
P70265	Pfkfb2	6	3	3	3	519	59.9	8.12
Q91WV0	Dr1	12	2	2	3	176	19.4	4.75
Q61466	Smarcd1	5	2	2	3	515	58.2	9.25
Q9WUN2	Tbk1	5	3	3	3	729	83.4	6.87
Q80ZS3	Mrps26	14	3	3	4	200	23.4	9.96
Q8BH64	Ehd2	4	1	2	3	543	61.1	6.51
P70349	Hint1	23	3	3	3	126	13.8	6.87
Q812E0	Cpeb2	7	2	2	2	521	58.4	7.5
Q9Z1K5	Arih1	6	3	3	5	555	64	5.08
Q9R1C6	Dgke	5	3	3	4	564	63.6	7.44
Q9CPQ8	Atp5mg	25	2	2	8	103	11.4	9.74
Q9R257	Hebp1	15	2	2	3	190	21.1	5.26
Q61749	Eif2b4	4	2	2	4	524	57.6	9.25
Q91V57	Chn1	7	2	2	2	459	53.2	6.76
O35083	Agpat1	7	1	1	1	285	31.7	9.14
Q01149	Col1a2	2	4	4	6	1372	129.5	9.19
P97298	Serpinf1	8	2	2	2	417	46.2	6.98
Q8BGU5	Ccny	9	2	2	3	341	39.4	7.2
P52825	Cpt2	4	2	2	2	658	73.9	8.37
O70433	Fhl2	10	2	2	2	279	32.1	7.3
Q8BQP9	Rgs7bp	14	2	2	2	257	29	8.46
Q9D273	Mmab	10	2	2	2	237	26.3	9.2
Q80X73	Pelo	5	3	3	4	385	43.3	5.99
Q91WK1	Spryd4	13	3	3	3	207	23.3	9.45
O70493	Snx12	16	2	3	3	165	19.1	7.34
Q3U0B3	Dhrs11	11	2	2	2	260	28.3	6.34
Q9DBS9	Osbpl3	3	2	2	3	855	96.9	6.51
Q9D7V2	Lysmd2	16	3	3	4	215	23.7	5.71
Q64674	Srm	10	2	2	2	302	34	5.5
Q8R3T5	Stxbp6	13	3	3	3	210	23.7	9.19
Q5U430	Ubr3	2	3	3	3	1889	212.6	5.9
Q9EQN3	Tsc22d4	4	1	2	4	387	40	8.13
Q8C4X2	Csnk1g3	6	3	3	3	424	48.9	9.11
O55026	Entpd2	5	2	2	4	495	54.3	8.37
Q149L6	Dnajb14	6	2	2	2	379	42.3	8.59
Q3UHG7	Dennd11	3	2	2	3	455	51.4	5.25
P14131	Rps16	15	3	3	6	146	16.4	10.2<br>1
Q9WV91	Ptgfrn	3	3	3	3	879	98.7	6.61
Q02105	C1qc	10	2	2	2	246	26	8.54
Q8R317	Ubqln1	6	1	2	10	582	61.9	4.94
Q9D1H6	Ndufaf4	15	3	3	3	173	20.1	9.39
Q14B80	Kcnc2	3	1	2	2	642	70.5	7.69

Q7TNV0	Dek	4	1	1	2	380	43.1	6.86
Q91VA6	Poldip2	7	2	2	2	368	41.8	8.63
O70492	Snx3	21	2	3	3	162	18.7	8.66
Q8BJW6	Eif2a	5	3	3	3	581	64.4	8.91
Q8CIV8	Tbce	5	2	2	3	524	59	6.29
Q6PHQ8	Naa35	4	2	2	2	725	83.3	7.3
Q8BFV2	Pcid2	9	4	4	4	399	46.1	8.53
Q99LB6	Mat2b	7	1	1	2	334	37.4	6.95
P51163	Uros	11	2	2	2	265	28.5	6.54
P97326	Cdh6	3	2	2	2	790	88.3	5
P52800	Efnb2	6	2	2	2	336	37.2	8.97
B7ZMP1	Xpnpep3	8	2	2	2	506	56.6	7.68
Q920Q6	Msi2	9	3	3	3	346	36.9	8.47
Q9QZB1	Rgs20	14	2	3	4	239	27	5.16
P26516	Psmd7	3	1	1	2	321	36.5	6.77
Q9DBD0	Ica	4	2	3	3	700	76.7	7.25
Q80WS3	Fbll1	10	2	2	2	314	33.3	10.15
P83917	Cbx1	15	1	2	3	185	21.4	4.93
Q3TIV5	Zc3h15	5	2	2	3	426	48.3	5.3
P55194	Sh3bp1	4	2	2	2	680	74.1	6.6
Q3UVG3	Fam91a1	3	2	2	2	837	93.4	6.35
Q8VE09	Ttc39c	6	3	3	3	580	65.4	6.87
Q9WV54	Asah1	6	3	3	3	394	44.6	8.46
Q8CHK3	Mboat7	8	2	2	2	473	53.4	8.69
Q4QQM4	Trp53i11	13	2	2	4	189	20.9	9.41
P70182	Pip5k1a	3	1	2	4	546	60.4	8.59
Q9D061	Acbd6	8	2	2	2	282	30.9	5.11
Q5NCX5	Neurl4	1	2	2	2	1563	167.5	5.95
Q62086	Pon2	9	2	2	2	354	39.6	5.83
Q9ERT9	Ppp1r1a	10	2	2	3	171	18.7	5.25
Q02566	Myh6	1	1	2	4	1938	223.4	5.73
Q6ZQ58	Larp1	1	2	2	3	1072	121.1	8.79
Q6P9R2	Oxsr1	6	1	2	2	527	58.2	6.43
P62311	Lsm3	12	1	1	2	102	11.8	4.7
Q80WW9	Ddrgk1	11	3	3	3	315	36	5.35
Q9Z2L7	Crlf3	5	2	2	3	442	49.5	5.03
P56380	Nudt2	15	2	2	4	147	17	6.28
P39749	Fen1	7	3	3	4	378	42.3	8.34
Q05CL8	Larp7	6	3	3	3	570	64.8	9.54
P29788	Vtn	4	2	2	5	478	54.8	5.88
Q9CY50	Ssr1	9	2	2	4	286	32	4.45
Q569Z5	Ddx46	3	3	3	4	1032	117.4	9.26
Q9QXE7	Tbl1x	4	1	2	2	527	56.8	5.72

Q8BRV5	Kiaa1671	9	3	3	5	308	34.6	6.09
Q8BH55	Thnsl1	4	3	3	4	747	83	7.2
O89106	Fhit	11	2	2	3	150	17.2	6.73
P08103	Hck	2	1	1	6	524	59.1	7.24
O35744	Chil3	8	2	2	3	398	44.4	5.69
P62320	Snrpd3	8	1	1	3	126	13.9	10.32
Q8BGA9	Oxa1l	2	1	1	4	433	48.2	9.61
Q99LJ0	Cttnbp2nl	4	3	3	3	638	69.8	7.71
P20352	F3	9	2	2	4	294	32.9	9.32
Q9DCC8	Tomm20	17	2	2	6	145	16.3	8.6
Q9QZC2	Plxnc1	2	3	4	4	1574	176.4	7.75
Q6ZPF3	Tiam2	2	2	2	2	1715	192.4	7.44
P34884	Mif	30	3	3	7	115	12.5	7.34
Q9Z1Q5	Clic1	10	2	2	3	241	27	5.17
Q8R4E6	Purg	7	2	2	2	350	39.9	9.51
Q3UMB5	Smcr8	4	2	2	2	935	104.9	5.4
Q9DBY1	Syvn1	3	2	2	2	612	67.3	6.95
D3YZV8	Ccdc8	11	2	2	3	685	73.9	9.09
Q8R2H9	Phospho1	10	3	3	3	267	29.9	7.94
Q14C51	Ptcd3	3	2	2	2	685	77.7	5.88
Q61599	Arhgdib	11	2	2	3	200	22.8	5.11
O35454	Clcn6	3	2	2	2	870	96.9	7.02
O88735	Map7	4	2	3	3	730	82	9.36
Q8VHX6	Flnc	2	3	4	4	2726	290.9	5.95
Q9Z2G6	Sel1l	4	3	3	4	790	88.3	5.57
Q3TIR1	Trappc13	4	2	2	3	417	46.4	5.49
Q9CQ49	Ncbp2	12	2	2	3	156	18	7.81
P56212	Arpp19	33	1	2	3	112	12.3	9.09
Q9QYE3	Bcl11a	3	2	2	2	773	83.8	6.28
Q9CQK7	Rwdd1	14	2	2	2	243	27.8	4.26
P63042	Stmn4	8	1	2	5	189	22.1	5.99
P70169	Doc2b	4	1	2	3	412	45.8	8.07
Q7TSH2	Phkb	2	3	3	3	1085	123.8	6.83
Q9JI75	Nqo2	8	2	2	3	231	26.2	7.01
Q9QWR8	Naga	2	1	1	2	415	47.2	6.44
B1AXH1	Nhsl2	2	3	3	4	1219	132.3	8.48
Q9DB27	Mcts1	13	2	2	4	181	20.5	8.82
P97470	Ppp4c	4	1	1	2	307	35.1	5.06
Q8CH09	Sugp2	3	2	2	2	1067	118	8.31
Q922H4	Gmppa	7	3	3	3	420	46.2	7.62
Q99K30	Eps8l2	4	3	3	3	729	82.2	7.18
P56565	S100a1	21	2	2	5	94	10.5	4.5
P85094	Isoc2a	12	2	2	3	206	22.4	8.02

Q9DCS3	Mecr	8	2	2	2	373	40.3	9.07
Q3UUQ7	Pgap1	1	1	1	2	922	104.5	8.91
Q91WG4	Elp2	2	2	2	3	831	93	5.82
Q9CZN7	Shmt2	6	3	3	3	504	55.7	8.47
Q8R555	Crtac1	4	2	2	2	646	70.3	5.14
Q8BTX9	Hsdl1	5	2	2	5	330	36.8	8.48
G3XA57	Rab11fip2	5	2	2	2	512	58.2	9.45
Q9DCH4	Eif3f	4	2	2	3	361	38	5.58
Q8VDH1	Fbxo21	5	2	2	2	627	72.1	6.06
P12657	Chrm1	5	2	2	4	460	51.3	9.32
Q08024	Cbfb	12	2	2	3	187	22	5.77
Q9R0A0	Pex14	5	2	2	3	376	41.2	5.11
Q6PDH0	Phldb1	2	3	3	3	1371	150	8.87
Q811U4	Mfn1	3	2	2	2	741	83.7	6.51
Q9Z0R9	Fads2	5	2	2	2	444	52.4	8.82
Q9CR67	Tmem33	4	1	1	2	247	28	9.66
Q9CYK1	Wars2	7	2	2	2	360	40.1	8.82
Q8BTV1	Tusc3	5	2	2	2	347	39.5	10.11
P49769	Psen1	7	2	2	2	467	52.6	5.38
Q8BWZ3	Naa25	2	2	2	3	972	111.6	6.52
Q9DCT1	Akr1e2	8	2	2	3	301	34.4	7.33
Q9CPX6	Atg3	6	2	2	3	314	35.8	4.72
P60603	Romo1	32	2	2	3	79	8.2	9.33
Q66GT5	Ptpmt1	15	3	3	3	193	21.9	9.72
Q61120	Shc3	5	3	3	3	474	52.1	7.3
Q9CQ91	Ndufa3	21	2	2	3	84	9.3	8.47
Q9CQH3	Ndufb5	8	2	2	4	189	21.7	9.41
Q08642	Padi2	4	2	2	3	673	76.2	5.53
Q9JLC4	Sorcs1	2	2	2	2	1167	129.6	7.55
Q9DC29	Abcb6	3	2	2	3	842	93.7	8.05
Q8VDC1	Fyco1	2	2	2	2	1437	162.2	4.98
P54731	Faf1	5	3	3	3	649	73.8	4.86
Q9CZU3	Mtrex	2	2	2	3	1040	117.6	6.4
Q8R086	Suox	3	2	2	3	546	60.7	6.54
Q8K2C6	Sirt5	10	2	2	2	310	34.1	8.53
B2RY56	Rbm25	3	3	3	3	838	99.5	6.32
Q9D1L0	Chchd2	7	1	1	2	153	15.7	9.66
Q91XF0	Pnpo	4	1	1	2	261	30.1	8.22
Q9QUG9	Rasgrp2	2	1	1	3	608	69.4	7.66
Q63959	Kcnc3	2	1	2	2	769	82.1	7.09
B5X0G2	Mup17	6	1	1	3	180	20.6	5.02
F6W8I0	Yjefn3	14	3	3	4	251	27	7.4

Q63943	Mef2d	4	2	2	3	514	55	7.88
Q8CCX5	Krt222	9	3	3	4	294	34.2	5.78
Q3UA37	Qrich1	2	1	1	2	777	86.5	5.96
Q8VE97	Srsf4	6	3	3	3	489	55.9	11.4
Q8R3R8	Gabarapl1	13	1	2	3	117	14	8.73
Q8QZV4	Stk32c	5	2	2	2	488	55.2	6.16
Q5SVR0	Tbc1d9b	2	2	2	2	1263	141.7	5.29
Q9D8T7	Slirp	17	2	2	2	112	12.6	9.82
Q60996	Ppp2r5c	6	3	3	3	524	60.8	6.64
Q9Z1T1	Ap3b1	3	3	3	3	1105	122.7	5.66
Q80U04	Pja2	6	3	3	3	707	77.9	4.44
Q9JHS4	Clpx	4	2	2	3	634	69.2	7.71
O08739	Ampd3	4	3	3	3	766	88.6	7.33
Q8BLJ3	Plcxd3	7	2	2	2	321	36.3	6.62
Q9DCA2	Mrps11	10	2	2	2	191	20.2	10.77
P35290	Rab24	11	2	2	3	203	23.1	6.23
Q02956	Prkcz	3	1	1	2	592	67.6	5.76
Q6PD28	Ppp2r5b	4	1	2	2	497	57.3	6.84
Q9DAI2	Ift22	12	1	1	1	185	20.8	5.25
Q8K4B0	Mta1	2	2	2	3	715	80.7	9.29
Q8BG73	Sh3bgrl2	11	1	1	3	107	12.2	5.55
Q8BTS4	Nup54	4	2	2	3	510	55.7	7.02
Q3UDK1	Trafd1	5	2	2	2	580	64.2	5.48
Q80TN4	Dnajc16	4	2	2	2	772	89.1	7.55
Q9D706	Rpap3	5	3	3	3	660	74.1	7.99
P56135	Atp5mf	14	1	1	2	88	10.3	9.95
Q8K1B8	Fermt3	5	2	2	3	665	75.6	7.05
Q8BTG7	Ndrg4	4	1	1	2	352	38.5	6.32
O35409	Folh1	3	2	2	3	752	84.5	8.1
Q7TSY6	Celf4	5	3	3	3	486	51.9	8
Q9JKC8	Ap3m1	3	1	2	3	418	46.9	6.93
Q3UHD3	Mtus2	2	2	2	2	1353	147.3	8.24
O35143	ATP5IF1	23	3	3	3	106	12.2	9.64
Q80U72	Scrib	2	3	3	3	1612	174	5.12
Q04735	Cdk16	5	2	3	3	496	55.9	7.87
P62996	Tra2b	6	2	2	3	288	33.6	11.25
Q7TSQ8	Pdpr	3	2	2	2	878	99.2	6.35
Q7TNE3	Spag7	11	2	2	4	227	25.9	7.43
Q07235	Serpine2	4	2	2	3	397	44.2	9.85
Q80ZD8	Amigo1	2	1	1	2	492	55.3	6.86
Q99LC8	Eif2b1	3	1	1	3	305	33.8	8.32

Q8BGZ4	Cdc23	3	2	2	3	597	68.5	7.18
Q922P9	Glyr1	6	3	3	3	546	59.7	9.22
Q8BTU1	Cfap20	11	2	2	2	193	22.7	9.76
Q61733	Mrps31	6	3	3	4	384	43.9	8.51
Q8BGV0	Nars2	6	3	3	3	477	53.9	7.36
Q99J23	Ghdc	5	2	2	3	532	58.5	7.56
Q9JKL4	Ndufaf3	6	1	1	2	185	20.7	8.05
D3YZI9	Pgbd5	5	2	2	4	523	58.3	8.85
Q9CSU0	Rprd1b	5	1	1	2	326	36.9	5.97
Q9D6F4	Gabra4	6	2	2	2	552	60.8	9.32
Q80U30	Clec16a	4	3	3	4	1036	116.2	6.05
Q9CYL5	Glipr2	8	1	1	2	154	17.1	9.51
Q8CFI5	Pars2	4	2	2	3	475	53.5	7.84
Q8K1R3	Pnpt1	3	2	2	2	783	85.6	8.03
Q9JJG0	Tacc2	1	1	2	3	1149	124.1	5.06
Q8CHP5	Pym1	15	3	3	3	203	22.7	9.5
P62855	Rps26	14	2	2	5	115	13	11
Q91YR7	Prpf6	3	3	3	4	941	106.7	8.09
Q9D666	Sun1	3	3	3	3	913	101.9	6.81
Q9CXF4	Tbc1d15	4	2	2	2	671	76.5	5.3
Q3TIX9	Usp39	3	2	2	2	564	65.1	8.9
Q8BGT7	Smndc1	4	1	1	3	238	26.7	7.24
Q9QYK7	Rnf11	8	1	1	3	154	17.4	4.78
Q7TSE6	Stk38l	5	3	3	5	464	53.7	6.96
Q61418	Clcn4	4	2	2	2	747	83.7	6.86
O08586	Pten	5	2	2	2	403	47.1	6.37
P98086	C1qa	11	2	2	4	245	26	9.11
P61458	Pcbd1	16	1	2	2	104	12	6.8
O88444	Adcy1	2	2	2	2	1118	123.3	8.47
Q99KI3	Emc3	12	2	2	2	261	30	6.81
Q7TMW6	Ciao3	5	2	2	2	476	53.1	6.48
Q80ZJ7	Snx32	8	3	3	3	404	46.6	6.96
E9Q735	Ube4a	2	3	3	3	1028	118.1	5.33
Q920P3	Brinp1	3	2	2	2	760	88.6	8.97
Q8R349	Cdc16	3	2	2	4	620	71.4	5.76
P38585	Ttl	7	2	2	2	377	43.1	6.46
Q99K23	Ufsp2	7	3	3	3	461	52.5	6.76
Q9DBR1	Xrn2	2	1	1	3	951	108.6	7.59
Q8R066	C1qtnf4	9	2	2	3	326	35	8.98
Q9ES46	Parvb	8	2	2	2	365	41.6	6.33
Q9CQC9	Sar1b	14	2	3	3	198	22.4	6.11
Q8CHW4	Eif2b5	3	2	2	2	717	80	5.07
Q99J45	Nrbp1	4	2	2	3	535	59.8	5.08
Q9JMC3	Dnaja4	6	2	2	4	397	44.9	7.58

P97813	Pld2	3	2	2	2	933	106.1	7.39
Q8VED5	Krt79	4	1	2	3	531	57.5	7.69
Q9D882	Fam241b	18	2	2	2	120	13.1	11.41
Q9ER58	Spock2	5	2	2	2	423	46.8	4.87
Q810J8	Zfyve1	2	2	2	3	777	86.9	7.34
Q8BHW2	Oscp1	5	2	2	3	379	43.2	7.2
O70293	Grk6	4	2	2	2	576	65.9	8.1
Q60612	Adora1	3	1	1	2	326	36.6	8.48
O35099	Map3k5	2	3	3	3	1380	154.4	5.78
Q9CR21	Ndufab1	10	1	1	2	156	17.4	5.21
O54950	Prkag1	6	2	2	2	330	37.5	7.14
Q9JI99	Sgpp1	5	1	1	1	430	47.7	8
O89079	Cope	7	1	1	3	308	34.5	5.06
P35550	Fbl	9	3	3	4	327	34.3	10.24
A2ARP1	Ppip5k1	2	2	2	2	1436	159.8	5.39
Q8K207		14	2	2	3	121	13.9	5.22
P04104	Krt1	4	2	2	3	637	65.6	8.15
Q3UMB9	Washc4	2	3	3	3	1173	136.3	7.37
Q8R0X2	Cacul1	10	2	2	2	377	42.1	5.27
Q0P5W1	Vps8	2	3	3	3	1427	161	5.58
Q8VCH5	Rabepk	2	1	1	3	380	41.1	5.95
Q8VE19	Mios	3	3	3	3	875	98.3	6.71
Q8BIF2	Rbfox3	7	1	2	3	374	40.6	7.9
Q3TRM8	Hk3	2	1	2	2	922	100	5.94
Q80UY2	Kcmf1	5	2	2	2	381	41.8	5.76
Q8VDS4	Rprd1a	6	1	1	1	312	35.7	7.58
Q6PE15	Abhd10	7	1	1	1	297	33	8.79
P06537	Nr3c1	2	2	2	2	783	86	6.35
P58069	Rasa2	3	2	3	3	847	96.3	7.59
Q3UGS4	Mcrip1	26	2	2	2	97	11.1	9.14
Q7TQG1	Plekha6	1	2	2	3	1173	131.3	8.97
Q3UMY5	Eml4	2	2	2	3	988	110	6.54
Q64310	Surf4	10	2	2	2	269	30.4	7.78
Q5RKR3	Islr2	4	2	2	2	745	79.7	5.49
Q9CQN3	Tomm6	19	1	1	2	74	7.9	4.89
P56379	Atp5mpl	16	1	1	5	58	6.7	9.99
P08905	Lyz2	15	2	2	2	148	16.7	8.81
Q9DB15	Mrpl12	13	2	2	3	201	21.7	9.29
P70399	Tp53bp1	1	2	2	3	1969	212.6	4.63
O35393	Efnb3	4	1	1	3	340	35.9	8.25
Q8BLN5	Lss	3	2	2	2	733	83.1	6.4
Q9WUU7	Ctsz	6	2	2	2	306	34	6.6

Q8BI72	Cdkn2aip	4	2	2	3	563	59.7	9.16
Q80YA7	Dpp8	4	3	3	4	892	102.1	5.81
Q99M01	Fars2	3	1	1	2	451	52.3	7.17
Q06180	Ptpn2	7	2	2	2	406	47.3	8.37
Q00560	Il6st	3	2	2	2	917	102.4	5.52
Q99MS8	Tpgs1	6	1	1	1	303	32.6	8.94
O89017	Lgmn	6	2	2	2	435	49.3	6.39
Q8BHJ5	Tbl1xr1	4	1	2	2	514	55.6	5.63
Q99LM2	Cdk5rap3	6	2	2	2	503	57	4.83
Q9R0L7	Akap8l	4	2	2	2	642	71.4	5.05
Q9WTN0	Ggps1	9	3	3	5	300	34.7	6.46
Q9CQW0	Emc6	9	1	1	2	110	12	10.07
A2AAE1	Kiaa1109	0	2	2	3	5005	555	6.61
O89116	Vti1a	4	1	1	3	217	25	6.4
Q9DBS1	Tmem43	6	2	2	3	400	44.8	7.36
Q91W43	Gldc	2	2	2	2	1025	113.2	7.56
Q9CQQ8	Lsm7	22	2	3	4	103	11.6	5.27
Q9DBX6	Cyp2s1	3	1	1	1	501	55.6	8.78
Q9D1G3	Hhatl	2	1	1	2	503	56.4	6.65
Q8N7N5	Dcaf8	5	2	2	2	591	66	5.87
Q9JJV5	Cacng3	5	2	2	3	315	35.5	9.48
Q3U2A8	Vars2	3	2	2	2	1060	118.4	7.14
Q3U487	Hectd3	3	2	2	2	861	97.3	5.47
Q9D5R2	Wdr20	4	2	2	3	567	62.7	8.48
Q6PCN3	Ttbk1	2	2	2	2	1308	141.5	5.62
Q9QY36	Naa10	10	2	2	2	235	26.5	5.64
Q3V3V9	Carmil2	2	2	2	2	1296	141.3	7.18
Q8VCN9	Tbcc	5	2	2	2	341	38.1	5.3
Q99P58	Rab27b	10	2	2	2	218	24.5	5.54
Q99MD9	Nasp	3	2	2	2	773	83.9	4.37
Q6NTA4	Rragb	2	1	1	3	374	43.2	6.38
Q8BYY4	Ttc39b	4	2	2	2	617	70.2	6.64
Q9DBR0	Akap8	4	2	2	2	687	76.2	5.14
Q62413	Epha6	2	2	2	3	1035	116.1	6.95
Q3UMT1	Ppp1r12c	3	2	2	2	782	84.6	6
Q9Z2C9	Mtmr7	4	2	2	2	660	75.6	6.43
Q6P5D8	Smchd1	1	2	2	2	2007	225.5	7.24
P41242	Matk	3	2	2	4	505	56	8.81
Q80TE4	Sipa1l2	1	1	2	2	1722	189.3	6.79
Q9R0P6	Sec11a	9	2	2	4	179	20.6	9.33
Q9CZG9	Pdzd11	17	1	1	2	140	16.2	7.14
Q9CZN4	Shisa9	7	2	2	3	424	46.8	8.62
P23298	Prkch	4	1	2	2	683	77.9	7.68

P97290	Serping1	5	2	2	2	504	55.5	6.29
Q8JZM7	Cdc73	2	1	1	2	531	60.5	9.61
Q80U35	Arhgef17	1	2	2	2	2057	221.5	6.3
Q9JJZ4	Ube2j1	7	2	2	3	318	35	6.99
Q924L1	Letmd1	5	2	2	2	360	41.7	10.54
Q9D0Q7	Mrpl45	5	2	2	3	306	35.4	9.23
Q01815	Cacna1c	1	2	2	2	2139	240	6.87
Q8BG67	Efr3a	3	2	2	2	819	92.6	6.83
Q02257	Jup	4	3	3	3	745	81.7	6.14
P24457	Cyp2d11	3	1	1	4	504	57	6.25
Q8BGW1	Fto	7	4	4	4	502	58	5.12
Q3U319	Rnf40	2	2	2	2	1001	113.9	6.48
Q9WTX8	Mad1l1	2	2	2	3	717	83.5	5.66
Q9CQ80	Vps25	6	1	1	3	176	20.7	6.34
Q2TA57	Asphd1	6	2	2	2	360	38.2	8.25
Q6NVG1	Lpcat4	5	3	3	3	524	57.1	8.75
Q61161	Map4k2	3	2	2	2	821	91.2	6.46
Q62422	Ostf1	9	2	2	2	215	23.8	5.68
Q8R310	Tmcc3	5	2	2	2	477	53.7	8.6
Q3UQ84	Tars2	4	3	3	3	723	81.6	7.87
Q80SY4	Mib1	2	2	2	2	1006	110	6.92
Q2WF71	Lrfn1	3	1	1	1	766	81.9	7.59
Q8C181	Mbnl2	5	2	2	2	373	40.1	9
O88983	Stx8	8	1	1	2	236	26.9	5.01
O89001	Cpd	2	2	2	2	1377	152.3	6.18
Q570Y9	Deptor	5	2	2	3	409	46.1	7.91
Q3U1N2	Srebf2	2	1	1	1	1130	122.8	8.44
Q9CR86	Carhsp1	13	2	2	3	148	16.1	8.21
O88502	Pde8a	2	1	2	2	823	93.1	5.85
Q3U2I3	Fam160a2	1	1	1	1	975	106.5	6.76
P17439	Gba	6	2	2	2	515	57.6	7.75
Q8K2H2	Otud6b	3	1	1	2	294	33.7	5.53
Q5SWP3	Nacad	1	2	2	3	1504	156.7	4.56
Q9JHJ0	Tmod3	6	2	2	2	352	39.5	5.14
P17047	Lamp2	5	2	2	2	415	45.7	7.39
Q99JX4	Eif3m	7	2	2	2	374	42.5	5.74
Q8BHE8	Maip1	3	1	1	2	291	33	9.14
Q60751	Igf1r	1	1	1	2	1373	155.7	5.74
Q8C996	Tmem163	8	2	2	2	288	31.2	7.93
Q6EDY6	Carmil1	2	2	2	2	1374	151.8	7.8
L0N7N1	Kif14	1	1	1	3	1674	186.3	7.84
A2A7S8	Kiaa1522	2	2	2	2	1013	104.8	9.69

Q63739	Ptp4a1	12	2	2	3	173	19.8	8.97
Q3UQ44	Iqgap2	2	2	2	2	1575	180.4	5.64
Q91WA3	Hdac11	6	2	2	4	347	39.1	7.14
Q924T2	Mrps2	7	1	1	1	291	32.3	9.14
Q9WV96	Timm10b	19	1	1	2	100	11.3	7.08
Q8C170	Myo9a	1	3	3	3	2542	291.9	8.97
Q80YD1	Supv3l1	3	2	2	2	779	87	7.84
Q9CZR3	Tomm40l	8	2	2	2	308	34	7.27
Q3U0M1	Trappc9	3	3	3	3	1148	128.2	6.47
Q91W34		4	2	2	2	466	50.4	7.15
Q91WG5	Prkag2	3	2	2	2	566	62.9	9.36
Q8K0C4	Cyp51a1	5	2	2	3	503	56.7	8.41
Q9DCV4	Rmdn1	6	2	2	3	305	35	8.7
Q9WUB4	Dctn6	14	3	3	3	190	20.7	6.67
P50096	Impdh1	4	1	2	3	514	55.2	6.8
Q8K2Q7	Brox	7	3	3	3	411	46.2	7.69
Q8K2Q9	Shtn1	4	3	3	3	631	71.3	5.44
Q6PHS6	Snx13	3	2	2	2	957	110.7	6.62
Q9DBN4	P33monox	6	2	2	3	303	32.7	9.42
Q80X85	Mrps7	7	1	1	1	242	28	9.94
P70268	Pkn1	3	3	3	3	946	104.3	6.27
Q8K097	Faim2	7	1	1	1	317	35.2	6.92
O35166	Gosr2	8	2	2	2	212	24.7	8.21
Q99L27	Gmpr2	7	3	3	3	348	38	7.44
Q99KK1	Reep3	6	1	1	1	254	29.2	9.58
Q9DBH5	Lman2	6	2	2	3	358	40.4	6.95
Q9JJG6	Tmem47	6	1	1	3	181	20	6.73
O89020	Afm	4	2	2	2	608	69.3	5.78
Q8C0I1	Agps	3	2	2	3	645	71.6	7.5
O55060	Tpmt	9	2	2	2	240	27.6	6.44
Q922H1	Prmt3	5	2	2	2	532	59.9	5.3
Q3U1V6	Uevld	2	1	1	2	471	51.6	7.28
Q9CQT1	Mri1	5	2	2	2	369	39.4	5.91
Q6A0D4	Rftn1	4	2	2	2	554	61.5	7.68
Q02013	Aqp1	7	1	1	1	269	28.8	7.43
P41234	Abca2	1	1	1	1	2434	270.3	6.76
P98203	Arvcf	4	2	2	3	962	105	6.64
Q8BP48	Metap1	5	1	1	1	386	43.2	7.17
Q2TPA8	Hsdl2	4	2	2	2	490	54.2	6.74
Q9R0X5	Rpgr	2	2	2	3	1001	111.7	4.61
Q80ZJ6	Zer1	3	2	2	2	779	89	5.94
Q6DG52	Churc1	12	1	1	2	112	12.8	4.84
Q9ESN4	C1ql3	7	2	2	2	255	26.7	6.79

P70677	Casp3	6	2	2	2	277	31.5	6.92
P61600	Naa20	11	2	2	2	178	20.4	5.03
Q8R409	Hexim1	6	2	2	2	356	40.2	5.38
O35598	Adam10	4	2	2	2	749	83.9	7.94
Q9D883	U2af1	4	1	1	3	239	27.8	8.81
Q64314	Cd34	8	2	2	2	382	41	5.3
P61804	Dad1	19	2	2	2	113	12.5	7.08
Q8CJ61	Cmtm4	9	2	2	2	208	22.9	5.59
Q8BH51	Cox14	26	2	2	4	57	6.4	9.79
Q9D3B1	Hacd2	7	2	2	4	254	28.4	9.58
Q9WTU3	Scn8a	1	1	2	2	1978	225	6.25
Q6NZN0	Rbm26	2	2	2	2	1012	114.1	9.16
Q9QZB0	Rgs17	10	1	2	3	210	24.3	5.71
Q99M31	Hspa14	4	2	2	2	509	54.6	5.92
Q6P5G6	Ubxn7	5	2	2	3	467	52.1	5.03
Q8BH24	Tm9sf4	3	2	2	2	643	74.6	7.23
Q8CGZ0	Cherp	1	1	1	2	936	106.1	9.14
Q99NF3	Cep41	5	1	1	3	373	41.4	8.12
Q8HW98	Iglon5	5	1	1	1	336	36.7	7.69
Q8BPB5	Efemp1	5	2	2	2	493	54.9	5.14
O08576	Rundc3a	6	2	2	2	446	50	5.5
Q8K3E5	Ahi1	2	2	2	2	1047	119.6	7.18
Q9CQM5	Txndc17	27	2	2	2	123	14	4.77
Q09200	B4galnt1	4	2	2	2	533	59.2	8.59
Q8K2Q0	Commd9	10	2	2	2	198	21.8	5.82
P46414	Cdkn1b	7	1	1	1	197	22.2	7.02
Q8JZS6	N4bp2l2	3	1	2	7	575	66.2	5.22
O88851	Rbbp9	11	2	2	3	186	20.9	5.97
Q9D8V7	Sec11c	8	2	2	3	192	21.6	9.23
Q91V36	Nrbp2	4	2	2	3	499	57.3	6.35
Q8BQM8	Eml5	1	3	3	3	1977	220	7.8
								10.4
Q9JIK9	Mrps34	10	2	2	2	218	25.8	3
Q9DBX2	Pdcl	7	2	2	2	301	34.4	4.87
Q791V5	Mtch2	5	1	1	1	303	33.5	8.25
Q7TN79	Akap7	6	2	2	3	314	35.5	7.87
Q9QXT0	Cnpy2	14	2	2	3	182	20.8	5.07
O54916	Reps1	3	2	3	3	795	86.5	5.58
								11.2
Q6PFR5	Tra2a	7	2	2	2	281	32.3	8
Q9D1M4	Eef1e1	10	2	2	2	174	19.8	8.59
Q9CWD8	Nubpl	6	1	1	1	319	34.1	9.07
Q3TCN2	Plbd2	4	2	2	3	594	66.2	6.13
Q91WG7	Dgkg	3	2	2	2	788	88.5	6.76

Q99LB7	Sardh	2	2	2	2	919	101.6	6.74
P35441	Thbs1	1	2	2	2	1170	129.6	4.96
Q99J10	Ctu1	4	2	2	2	420	43.8	8.1
Q9CZN8	Qrsl1	4	2	2	2	525	56.7	5.95
Q8BJ05	Zc3h14	2	2	2	2	735	82.4	7.37
O88939	Zbtb7a	4	1	1	2	569	60.2	5.12
Q9JKW0	Arl6ip1	5	1	1	2	203	23.4	9.32
Q8BXR1	Slc7a14	3	2	2	2	771	83.9	5.35
Q3THK3	Gtf2f1	4	1	1	1	508	57.2	7.01
Q8BG89	Znf365	4	2	2	2	408	46.8	9.14
Q8R4F1	Ntng2	3	2	2	3	589	66.1	6.13
Q6PGB6	Naa50	13	2	2	3	169	19.4	8.81
Q3UHE1	Pitpnm3	3	2	2	2	974	106.4	7.12
Q99JW4	Lims1	6	2	2	5	325	37.2	8.05
Q9CQF3	Nudt21	11	2	2	3	227	26.2	8.82
Q6A028	Swap70	3	2	2	2	585	69	6.05
Q9JI19	Fibp	2	1	1	4	357	41.2	6.76
Q9CQN7	Mrpl41	19	2	2	2	135	15.3	9.82
Q5SUF2	Luc7l3	6	2	2	2	432	51.4	9.77
Q9R0U0	Srsf10	7	2	2	3	262	31.3	11.27
Q8R092		5	1	1	3	253	28.7	9.36
Q99J31	Ophn1	2	2	2	2	802	91.9	7.96
Q8C460	Eri3	5	2	2	2	337	37.2	8.07
P56716	Rp1	0	1	1	2	2095	234.2	7.53
Q9CQY1	Atg12	6	1	1	2	141	15.2	4.97
Q6P1D5	Sez6l	2	1	1	1	963	104.8	4.69
Q8VCS3	Fam20b	7	2	2	2	409	46.6	6.95
Q3TRR0	Map9	3	2	2	2	646	73.5	7.62
Q6NXJ0	Wwc2	2	1	1	1	1187	132.5	5.71
Q80Z10	Astn2	1	1	1	1	1352	149.3	6.07
Q6NSW3	Sphkap	2	2	2	2	1687	185	5.06
P18531	Ighv3-6	14	1	1	1	116	13.1	8.78
Q8K1R7	Nek9	2	2	2	2	984	107.1	5.63
P16390	Kcna3	4	2	2	2	528	58.5	5.33
Q68FH4	Galk2	3	2	2	2	458	50.5	6.9
Q8BTY1	Kyat1	4	1	1	1	424	47.5	6.95
Q6DFV7	Ncoa7	3	2	2	2	943	106.3	5.43
Q91VF2	Hnmt	11	2	2	2	295	33.6	5.06
Q61136	Prpf4b	1	1	1	2	1007	116.9	10.23
P09602	Hmgn2	9	1	1	3	90	9.4	9.99
Q9Z239	Fxyd1	13	1	1	1	92	10.3	8.54
Q3UN02	Lclat1	5	2	2	2	376	44.4	8.53

Q9ET22	Dpp7	3	1	1	2	506	56.2	5.39
Q8BM85	Tbck	2	2	2	2	762	86.3	6.2
Q99JT9	Adi1	14	2	2	2	179	21.5	5.5
Q9D2U5	Naa38	14	2	2	3	125	13.4	5.5
P70288	Hdac2	3	2	2	3	488	55.3	5.91
Q99N95	Mrpl3	6	2	2	4	348	39.1	9.55
Q9CPT3	Nanp	9	2	2	2	248	27.8	6.07
O54962	Banf1	17	2	2	3	89	10.1	6.09
Q60710	Samhd1	3	2	2	2	658	75.8	7.93
Q9CRB8	Mtfp1	7	1	1	3	166	18.3	8.68
Q67BT3	Slc13a5	1	1	1	2	572	63.8	7.64
Q8VCH8	Ubxn4	4	2	2	2	506	56.4	6.61
P53995	Anapc1	1	2	2	2	1944	215.9	6.35
Q8R3L2	Tcf25	4	3	3	3	676	76.6	6.51
P28184	Mt3	34	2	2	2	68	7	7.47
Q8C050	Rps6ka5	2	2	2	2	863	96.5	7.34
O35114	Scarb2	2	1	1	2	478	54	5.1
O88736	Hsd17b7	5	1	1	1	334	37.3	6.73
Q8CB44	Gramd4	3	1	1	2	633	72.2	9.07
Q9R112	Sqor	3	1	1	2	450	50.3	9.09
Q6ZQ88	Kdm1a	2	2	2	2	853	92.8	6.52
Q62507	Coch	4	2	2	2	552	59.9	8.44
Q9WUR9	Ak4	4	1	1	2	223	25	7.53
P53986	Slc16a1	4	2	2	3	493	53.2	7.47
Q8K1C0	Angel2	3	2	2	2	544	62.4	8.21
P24472	Gsta4	9	2	2	2	222	25.5	7.39
Q8BPM2	Map4k5	3	2	2	2	847	95	7.83
Q9CR84	Atp5mc1	5	1	1	18	136	14.2	10.01
Q9WVQ5	Apip	9	2	2	2	241	26.9	6.9
Q80YF9	Arhgap33	2	1	2	3	1305	139.7	9.19
P83870	Phf5a	13	2	2	2	110	12.4	8.41
P58466	Ctdsp1	8	2	2	2	261	29.2	6.13
P83887	Tubg1	4	2	2	2	451	51.1	6.02
Q921H9	Coa7	10	3	3	3	231	25.6	6.29
O35900	Lsm2	7	1	1	2	95	10.8	6.52
Q8BGY9	Slc5a7	1	1	1	2	580	63.3	5.33
Q5SUC9	Sco1	8	2	2	3	284	31.6	8.47
Q8BVG8	Nat14	15	2	2	2	206	21.8	10.74
Q9CZ83	Mrpl55	12	1	1	1	127	15.1	10.51
P01843		16	1	1	1	105	11.6	6.27
Q8BH27	Megf9	4	2	2	3	600	62.8	5.47

Q9CXX9	Cuedc2	6	1	1	1	284	31.8	4.98
Q9QX66	Dpf1	7	2	2	2	387	44.2	7.09
P61460	Depdc5	1	2	2	2	1591	180.3	6.74
Q9D1I5	Mcee	11	2	2	2	178	19	9.09
P57080	Usp25	2	1	1	1	1055	121.3	5.31
P62080	Tspan5	6	1	1	2	268	30.3	4.78
Q9D0V7	Ebag9	9	1	1	1	213	24.3	6.29
Q8BIE6	Frmd4a	2	1	1	1	1020	113.8	8.92
Q9JM63	Kcnj10	6	2	2	2	379	42.4	8.29
Q9D7I5	Lhpp	8	2	2	2	270	29.1	5.1
Q61578	Fdxr	5	2	2	2	494	54.2	8.66
Q8R4I7	Neto1	3	2	2	2	533	60.2	6.99
Q9Z2C5	Mtm1	3	1	1	1	603	69.5	7.75
P84104	Srsf3	5	1	1	3	164	19.3	11.6<br>5
Q9CXI0	Coq5	5	1	1	1	327	37.3	7.49
P12265	Gusb	3	2	2	2	648	74.1	6.7
Q61503	Nt5e	4	2	2	2	576	63.8	6.64
Q922H9	Znf330	5	1	1	1	316	35.6	6.16
Q3UUI3	Them4	11	2	2	2	230	26	9.64
O35316	Slc6a6	1	1	1	2	621	69.8	7.23
Q8R059	Gale	5	1	1	1	347	38.2	6.74
P70295	Aup1	5	2	2	2	410	46.1	8.4
Q8BHJ6	Serinc5	4	2	2	2	461	51.8	8.07
Q8BIQ5	Cstf2	5	3	3	3	580	61.3	6.83
Q7M757	C6.1al	3	1	1	2	291	33.1	6.23
Q9DC23	Dnajc10	2	2	2	2	793	90.5	6.96
Q3U3E2	Fam117b	4	2	2	2	584	61.3	9.95
Q76LS9	Mindy1	4	1	1	2	468	51.2	4.73
P19324	Serpinh1	3	1	1	2	417	46.5	8.82
Q9D0T1	Snu13	9	1	1	1	128	14.2	8.46
Q9CQU3	Rer1	9	1	1	2	196	23	9.51
Q8R5A3	Apbb1ip	3	2	2	2	670	74.3	5.35
Q9CYA0	Creld2	5	1	1	1	350	38.2	4.58
Q8C0L6	Paox	3	1	1	2	504	55.4	5.11
Q9D517	Agpat3	5	2	2	2	376	43.3	8.51
A2AT37	Upf2	2	2	2	2	1269	147.5	5.64
Q91VC9	Ghitm	3	1	1	4	346	37.3	9.8
Q6ZPR4	Kcnt1	1	1	1	1	1224	138	7.52
P06797	Ctsl	5	1	1	2	334	37.5	6.83
Q8R2Y8	Ptrh2	10	1	1	1	181	19.5	7.42
Q9WTX2	Prkra	5	1	1	1	313	34.3	8.43
P03899	Mtnd3	13	1	1	2	115	13.2	4.64
Q6IMP4	Panx2	1	1	1	3	677	74.6	7.52

Q78IK4	Apool	8	2	2	2	265	29.2	9.31
Q9EQJ9	Magi3	1	2	2	2	1476	161.6	8.05
O70481	Ubr1	1	2	2	2	1757	200.1	6.05
Q9DCL8	Ppp1r2	5	1	1	2	206	23.1	4.83
Q9CZ28	Snf8	9	2	2	2	258	28.9	6.65
Q80X82	Sympk	1	1	1	1	1284	142.2	6.05
P51655	Gpc4	3	1	1	2	557	62.5	6.33
Q80U58	Pum2	2	2	2	3	1066	114.2	7.08
Q64008	Rab34	5	2	2	2	259	29.1	8.27
Q8VBT0	Tmx1	7	2	2	2	278	31.4	5.29
Q6P1I6	Psd2	1	1	1	1	770	84.2	5.21
O08784	Tcof1	1	1	1	1	1320	134.9	9.35
Q8C1D8	Iws1	2	1	1	1	766	85.2	4.67
Q8BGE6	Atg4b	7	2	2	2	393	44.3	5.07
P01592	Jchain	18	2	2	3	159	18	4.89
Q7TSG2	Ctdp1	2	1	1	1	960	104.5	5.39
Q80YA3	Ddhd1	3	2	2	2	547	61.8	6.61
Q9WVI9	Mapk8ip1	1	1	1	2	707	77.2	4.96
Q61625	Grid2	2	1	1	1	1007	113	6.1
P70444	Bid	9	1	1	2	195	21.9	4.81
Q0KK55	Kndc1	1	2	2	2	1742	191.2	6.3
Q9DB75	Cdip1	8	2	2	2	208	21.8	5.66
Q6QD59	Bnip1	8	2	2	2	228	26.2	8.75
P03911	Mtnd4	3	2	2	4	459	51.8	9.38
Q80YV2	Zc3hc1	3	1	1	2	501	55.2	5.38
Q9D287	Bcas2	12	2	2	2	225	26.1	5.66
Q61189	Clns1a	16	2	2	2	236	26	4.12
Q8R3Q0	Saraf	5	2	2	2	334	35.8	8.19
Q6PGE7	Slc6a7	4	2	2	2	637	71	6.61
Q69Z23	Dnah17	0	1	1	2	4481	511.3	5.64
P05532	Kit	2	2	2	2	979	109.3	7.21
Q8R313	Exoc6	2	2	2	2	802	93	6.15
Q9D1G2	Pmvk	9	2	2	2	192	21.9	5.99
Q3TKY6	Cwc27	4	2	2	2	469	53.5	5.53
Q8BXN7	Ppm1k	5	2	2	2	372	40.9	6.39
Q6P3D0	Nudt16	10	2	2	2	195	21.8	7.12
								10.2
Q9D4F2	Plpp6	6	2	2	2	292	31.7	9
Q8VD62	Bles03	6	2	2	3	298	31.8	6.1
Q504M8	Rab26	3	1	1	2	260	28.6	9.31
Q3TYD6	Lmtk2	2	2	2	2	1471	160.4	4.51
Q9CVD2	Atxn3	5	1	1	1	355	40.5	4.83
Q9ERG0	Lima1	2	1	1	1	753	84	6.6
Q7TPW1	Nexn	4	3	3	3	607	72.1	5.01

Q6NSR8	Npepl1	2	1	1	1	524	55.9	6.84
Q5NCI0	Urgcp	1	1	1	1	926	104.6	6.32
Q6PDC0	Rundc3b	2	1	1	2	408	45.1	5.17
Q91XD6	Vps36	4	1	1	2	386	43.7	7.15
Q3UNA4	Nxt2	15	3	3	3	142	16.2	5.81
Q5NCF2	Trappc1	11	1	1	1	145	16.9	9.16
Q80XU8	Lrfn4	2	1	1	2	636	67.2	6.81
Q9DBE0	Csad	4	2	2	2	493	55.1	6.61
P69566	Ranbp9	2	1	1	2	653	71	6.84
Q8BZZ3	Wwp1	2	2	2	3	918	104.6	6.38
Q8VE92	Rbm4b	7	2	2	2	357	40	6.74
Q8CEI1	Bola3	6	1	1	4	110	12.2	8.97
Q8BP40	Acp6	6	2	2	2	418	47.6	7.72
P14220	Gypa	18	1	1	1	168	17.7	5.1
Q60766	Irgm1	4	1	1	2	409	46.5	8.28
Q9D6T0	Nosip	6	2	2	2	301	33.2	8.62
Q9CQ79	Txndc9	3	1	1	3	226	26.2	5.95
Q8C2E7	Washc5	2	2	2	2	1159	134	7.12
Q61127	Nab2	3	2	2	2	525	56.5	6.86
Q8BRN9	Cc2d1b	2	2	2	2	848	93	5.41
Q91YN9	Bag2	9	2	2	2	210	23.5	6.42
Q00519	Xdh	1	2	2	2	1335	146.5	7.56
P18608	Hmgn1	8	1	1	3	96	10.1	9.76
Q8R2Z5	Vwa1	6	2	2	2	415	44.7	6.54
Q9CPR7	Sike1	8	2	2	2	207	23.5	5.38
Q8BX17	Gemin5	1	2	2	2	1502	166.5	6.71
Q07797	Lgals3bp	3	1	1	1	577	64.5	5.14
Q8C4Q6	Aida	6	1	1	1	305	34.9	6.74
Q3UFS0	Zyg11b	2	1	1	2	744	83.9	6.87
Q3UKC1	Tax1bp1	3	2	2	2	814	93.6	5.33
Q9DCR2	Ap3s1	6	1	1	1	193	21.7	5.39
Q8BIV3	Ranbp6	3	2	2	2	1105	124.5	5.03
Q32NY4	Cnnm3	4	2	2	2	713	76.2	5.58
Q9CY73	Mrpl44	6	1	1	1	333	37.5	8.51
Q99M80	Ptprt	2	2	2	2	1454	162.9	6.84
P29416	Hexa	3	2	2	2	528	60.6	6.54
P22682	Cbl	3	2	2	2	913	100.5	6.67
Q8C436	Vcpkmt	7	1	1	1	228	25.5	4.73
Q7TMC8	Fcsk	1	1	1	1	1090	119.2	6.6
Q6NS46	Pdcd11	1	1	1	1	1862	207.6	8.75
Q9QXT8	Kcnip3	5	1	1	1	256	29.4	5.69
P31809	Ceacam1	3	1	1	1	521	57	5.58
Q9CPP0	Npm3	13	1	2	2	175	19	4.82
Q7TQF2	Fbxo10	3	2	2	2	950	104.4	7.85

Q61672	Slc29a2	5	2	2	2	456	50.2	6.51
Q8C0M0	Wdr59	2	1	1	1	992	111.7	7.85
Q9CQ85	Timm22	8	1	1	1	194	20.1	8.22
O70362	Gpld1	2	2	2	2	837	93.2	7.12
Q9D8L5	Ccdc91	5	2	2	2	442	50	5.07
Q9CQE5	Rgs10	7	1	1	1	181	21.1	6.81
O35188	Cx3cl1	5	1	1	1	395	42.1	5.54
Q9DCD2	Xab2	2	2	2	2	855	99.9	6.23
P51906	Slc1a1	3	1	1	2	523	56.7	6.11
Q61823	Pdcd4	4	2	2	2	469	51.7	5.16
Q64669	Nqo1	6	2	2	2	274	30.9	8.72
Q8BKC8	Pi4kb	2	1	1	1	816	91.5	6.32
Q5DTM8	Rnf20	2	2	2	2	973	113.5	5.96
Q641K1	Agtpbp1	1	1	1	1	1218	137.1	6.52
Q99M28	Rnps1	5	1	1	1	305	34.2	11.84
Q64520	Guk1	9	2	2	2	198	21.9	6.55
Q8C561	Lmbrd2	2	1	1	1	694	81	7.34
Q571K4	Tab3	2	1	1	1	716	79	8.5
Q5SXA9	Wwc1	1	2	2	2	1104	124	5.97
Q61271	Acvr1b	3	1	1	1	505	56.7	7.17
P0DN90	Tmem254b	9	1	1	1	123	14.2	9.74
Q61107	Gbp4	2	1	1	2	620	70.8	6.64
Q9CQV7	Dnajc19	7	1	1	2	116	12.4	10.1
Q6GQT5	Tmem151a	3	1	1	1	468	51.3	7.99
Q9R099	Tbl2	4	2	2	2	442	49.6	9.04
Q8VC42	Rmc1	2	1	1	1	657	74.9	7.83
Q9D8Y1	Tmem126a	11	2	2	3	196	21.5	9.41
Q62074	Prkci	3	1	1	1	595	68.2	5.85
Q8C8T8	Tsr2	13	2	2	4	191	20.9	4.23
Q8CCP0	Nemf	2	2	2	2	1064	121.1	6.8
Q3TMX7	Qsox2	3	1	1	1	692	77.7	8.59
Q80W54	Zmpste24	3	1	1	2	475	54.7	6.95
Q91ZH7	Abhd3	3	1	1	1	411	46.2	7.49
Q9D735	Trir	11	2	2	2	173	18.4	9.67
Q91ZV0	Mia2	1	1	1	1	1396	156.4	4.55
Q8BGA3	Lrrtm2	4	2	2	2	515	58.8	8
Q9Z0J0	Npc2	11	1	1	1	149	16.4	7.68
Q9QXY9	Pex3	3	1	1	1	372	42.2	7.77
Q9Z2X2	Psmd10	12	1	1	1	231	25.1	6.06
Q3TAS6	Emc10	6	2	2	2	258	27	5.91

Q9CQL1	Magohb	14	1	1	1	146	17.1	6.39
Q8R4G0	Ntng1	3	1	1	1	539	60.5	6.24
P08030	Aprt	10	2	2	2	180	19.7	6.79
Q8R0X7	Sgpl1	3	1	1	2	568	63.6	9.1
Q80ZM7	Gtf2a2	6	1	1	2	109	12.5	6.62
Q9QZD8	Slc25a10	7	2	2	2	287	31.7	9.32
P12246	Apcs	9	1	1	2	224	26.2	6.35
Q9EP72	Emc7	8	2	2	2	241	26.3	9.23
Q920M5	Coro6	4	1	2	2	471	52.6	5.96
Q9JMD0	Znf207	3	1	1	2	495	52.8	9.1
P21300	Akr1b7	2	1	1	2	316	36	7.25
Q6IFX2	Krt42	4	1	2	2	452	50.1	5.16
Q8K296	Mtmr3	1	1	1	1	1196	133.8	5.83
Q9D2N9	Vps33a	3	2	2	2	598	67.5	7.08
Q8BH82	Napepld	5	2	2	2	396	45.8	5.94
Q6P1H6	Ankle2	1	1	1	1	964	106.1	7.37
E9PZJ8	Ascc3	1	2	2	2	2198	250.4	7.02
C0HKD8	Mfap1a	5	2	2	2	439	51.9	4.98
B9EKI3	Tmf1	1	2	2	2	1091	121.7	4.87
Q3U2S4	Otud5	3	1	1	1	566	60.3	6.54
Q9ERA6	Tfip11	1	1	1	1	838	96.2	5.9
Q6V4S5	Sdk2	1	2	2	2	2176	239.8	7.36
Q14CH0	Fam171b	3	2	2	2	825	92	8.37
Q9CZP5	Bcs1l	6	3	3	3	418	47.4	7.93
Q99M04	Lias	5	2	2	2	373	41.9	8.88
Q8CAF4	Nhsl1	1	1	1	1	1587	169.3	7.49
P56393	Cox7b	9	1	1	2	80	9	10.27
E9PXF8	Sbf2	1	2	2	3	1872	210.3	7.28
Q9CR59	Gadd45gip1	8	2	2	2	222	25.8	10.27
Q8BH70	Fbxl4	3	1	1	1	621	70.2	6.37
P97952	Scn1b	7	2	2	2	218	24.6	4.83
Q6ZWQ0	Syne2	0	2	2	2	6874	782.2	5.33
Q3USH1	Insyn2a	4	1	1	1	422	46.2	7.14
Q8VI63	Mob2	5	1	1	1	235	26.8	6.52
Q61923	Kcna6	2	1	1	2	529	58.6	5.03
Q9QZ08	Nagk	5	2	2	2	343	37.2	5.73
Q99KW9	Itfg1	2	1	1	1	610	67.4	5.73
O35704	Sptlc1	3	1	1	1	473	52.5	6.4
P05555	Itgam	2	2	2	2	1153	127.4	7.27
Q99MX7	Tmem121b	4	1	1	1	572	58.1	9.01
Q9DAM5	Slc25a19	5	1	1	1	318	35.6	9.2

Q8K0X8	Fez1	5	2	2	2	392	45.2	4.36
Q61387	Cox7a2l	14	2	2	3	111	12.4	9.6
P61148	Fgf1	7	1	1	2	155	17.4	7.02
								11.8
Q91Z49	Fyttd1	8	2	2	2	317	35.9	4
Q91WE2	Fam192a	7	2	2	3	254	28.7	5.14
P06683	C9	4	2	2	2	548	62	5.78
Q3UJD6	Usp19	2	2	2	2	1360	150.5	6.38
Q9Z2A5	Ate1	2	1	1	1	516	59.1	8.19
Q9EPU4	Cpsf1	1	2	2	2	1441	160.7	6.39
Q9JIG4	Ppp1r3f	1	1	1	1	799	84.1	4.7
Q9CWX2	Ndufaf1	6	2	2	2	328	37.8	8.6
Q9QXE0	Hacl1	2	1	1	1	581	63.6	6.27
P19973	Lsp1	3	1	1	1	330	36.7	4.82
A2AFR3	Frmpd4	2	1	1	1	1320	144.9	5.25
A2A6T1	Cdr2l	3	1	1	1	465	53.2	5.76
Q9Z1M8	Ik	3	1	1	1	557	65.6	6.64
Q8BR70	Yipf6	6	1	1	1	236	26.1	6.55
Q9ER88	Dap3	6	2	2	2	391	44.7	8.94
Q8R0P4	Aamdc	15	2	2	2	122	13.2	7.99
O54786	Dffa	7	2	2	2	331	36.6	4.91
Q03717	Kcnb1	2	2	2	2	857	95.5	8.16
Q8BU88	Mrpl22	9	2	2	2	206	23.8	9.86
P15388	Kcnc1	3	1	2	2	511	57.9	6.58
Q6RUT7	Ccsmst1	18	1	1	1	136	15.3	4.93
Q8C5L6	Inpp5k	4	2	2	2	468	54.1	5.88
Q8BKG3	Ptk7	2	1	1	1	1062	117.5	6.84
Q7TNG8	Ldhd	3	1	1	1	484	51.8	6.62
Q7TSK3	Pcdh8	2	2	2	2	1070	113.2	5.39
Q78J03	Msrb2	6	1	1	2	175	19.1	9.06
P19137	Lama1	1	2	2	2	3083	337.9	6.71
P11103	Parp1	1	1	1	2	1013	113	8.95
P97799	Nrsn1	7	1	1	1	196	21.6	7.99
Q6P9J5	Kank4	2	1	1	1	1016	110.3	4.88
P21995	Emb	4	1	1	1	330	37	6.02
Q8K1S3	Unc5b	1	1	2	2	945	103.7	6.16
Q8BYZ1	Abi3	4	1	1	1	367	39.1	5.54
Q9R1J0	Nsdhl	6	2	2	2	362	40.7	7.85
Q8VHK9	Dhx36	1	1	1	1	1001	113.8	8.29
Q6P5F6	Slc39a10	1	1	1	2	833	94.3	6.71
Q3UN16	Gpr162	2	1	1	1	588	64.1	8.79
Q8VCG4	C8g	7	1	1	1	202	22.5	9.25
Q5DTN8	Jakmip3	2	1	1	1	844	98.7	5.74
Q8BM65	Nyap2	2	1	1	1	682	73.9	8.72

P52332	Jak1	1	1	1	1	1153	133.3	7.59
P62309	Snrpg	26	2	2	3	76	8.5	8.88
Q9D7M1	Gid8	7	1	1	1	228	26.8	4.97
A2AJT4	Pnisr	3	1	1	1	805	92.1	10.01
Q99JH7	Clstn3	2	2	2	2	956	105.8	5.34
B0F2B4	Nlgn4l	2	2	2	2	945	97.3	6.32
Q6IEE6	Tmem132e	2	1	1	1	1074	116.2	6.14
Q91WG8	Gne	1	1	1	1	722	79.1	6.81
Q9QYF9	Ndrg3	4	1	1	1	375	41.5	5.25
Q60967	Papss1	3	2	2	2	624	70.7	6.77
P97822	Anp32e	5	1	1	1	260	29.6	3.88
P43346	Dck	6	1	1	1	260	30.3	5.43
O88967	Yme1l1	2	1	1	1	715	80	8.97
Q9D823	Rpl37	14	2	2	2	97	11.1	11.74
Q6P5D3	Dhx57	1	1	1	1	1388	155.7	7.87
Q68FE6	Ripor1	1	1	1	1	1223	132.3	5.88
Q3ULB5	Pak6	2	1	1	1	682	74.8	9.44
Q8C0Y0	Ppp4r4	1	1	1	2	875	99.4	7.71
Q8BM72	Hspa13	3	1	1	1	471	51.7	5.63
Q3UQS2	Lsmem1	5	1	1	4	128	14.2	5.48
P13439	Umps	2	1	1	1	481	52.3	6.61
Q8BW22	Ss18l1	3	1	1	1	402	43.7	6.58
Q9D920	Borcs5	10	2	2	2	195	22.1	6.55
Q9D853	Eef1akmt2	7	1	1	1	244	26.8	5.15
Q9QXW2	Fbxw5	2	1	1	1	573	64.6	6.13
Q9Z2D3	Gsdme	2	1	1	1	512	56.6	5.39
P48024	Eif1	12	1	1	1	113	12.7	7.44
Q91VU6	Dcaf11	3	2	2	2	549	62	6.44
P07934	Phkg1	5	2	2	2	388	44.9	6.55
Q8K2T8	Paf1	2	1	1	1	535	60.5	4.65
Q9D6W8	Borcs6	4	1	1	1	360	38	5.34
P0DP60	Lynx1	15	2	2	3	116	12.8	8.19
Q9R0D8	Wdr54	4	1	1	1	334	35.6	6.21
P62342	Selenot	7	1	1	1	195	22.3	8.6
O08908	Pik3r2	2	1	2	3	722	81.2	6.07
Q9JKF1	Iqgap1	1	2	2	2	1657	188.6	6.48
Q8VDU5	Snrk	1	1	1	1	748	81.9	7.49
O70139	Pkig	21	1	1	1	76	7.9	4.21
Q3UN04	Usp30	3	2	2	2	517	58.2	8.46
P60041	Sst	7	1	1	2	116	12.7	5.52

Q8R1F1	Niban2	2	2	2	2	749	84.8	5.94
Q9D554	Sf3a3	3	2	2	2	501	58.8	5.34
Q8C5Q4	Grsf1	4	1	1	1	479	53	6.67
Q8BXA5	Clptm1l	2	1	1	1	539	62.1	8.84
P97855	G3bp1	4	1	1	1	465	51.8	5.59
P25911	Lyn	2	1	1	2	512	58.8	7.15
Q8BJ03	Cox15	3	1	1	1	413	45.8	9.79
Q9ET26	Rnf114	5	1	1	1	229	25.7	7.03
Q9WVH9	Fbln5	4	2	2	2	448	50.2	4.7
Q62203	Sf3a2	3	1	1	1	475	49.9	9.54
E1U8D0	Soga1	1	1	1	1	1418	159.1	6.46
O88874	Ccnk	3	1	1	3	554	61.3	8.41
Q5SZV5	Kiaa0319	2	1	1	1	1081	117.9	5.17
Q91W96	Anapc4	1	1	1	1	807	91.6	5.4
O08581	Kcnk1	6	1	1	1	336	38.2	6.07
S4R2P9	Slc8a3	2	2	2	2	928	102.9	5.24
Q8CBG9	Rnf170	5	1	1	1	286	33.4	5.66
Q99ME9	Gtpbp4	3	2	2	2	634	74.1	9.52
Q8VE11	Mtmr6	2	1	1	1	617	70.9	7.83
Q9JHW4	Eefsec	2	1	1	2	583	63.5	8.29
Q5FWH2	Unkl	3	1	1	1	727	79.6	7.99
Q8BZH4	Pogz	1	1	1	1	1409	154.8	7.52
Q9QZN1	Fbxl17	2	1	1	1	701	75.6	8.1
P36895	Bmpr1a	3	1	1	1	532	60	7.3
Q8R0G7	Spns1	3	1	1	2	528	56.7	7.15
Q8VDP3	Mical1	2	2	2	2	1048	116.7	6.05
P49935	Ctsh	8	1	1	1	333	37.1	8.4
P35951	Ldlr	1	1	1	1	862	94.9	5.02
Q4U2R1	Herc2	0	1	1	1	4836	527.1	6.27
Q8CAK1	Iba57	4	1	1	1	358	38.4	9.01
O08644	Ephb6	2	2	2	2	1014	110	6.83
Q60805	Mertk	2	2	2	2	994	110.1	5.5
Q62384	Zpr1	3	1	1	1	459	50.7	4.78
Q8C5P7	Tdrp	8	1	1	1	182	20.2	5.73
P52624	Upp1	3	1	1	3	311	34.1	6.58
P41731	Cd63	6	2	2	2	238	25.7	6.98
O08989	Mras	7	2	2	2	208	23.9	8.78
Q8BP00	Iqcb1	3	2	2	2	598	68.7	9.31
Q9CXJ1	Ears2	3	1	1	2	523	58.3	8.75
P29351	Ptpn6	2	1	1	1	595	67.5	7.81
Q9CQ71	Rpa3	14	1	1	1	121	13.6	4.84
Q8K377	Lrrtm1	3	2	2	2	522	58.7	7.11
Q3UIA2	Arhgap17	2	1	1	1	846	92.1	7.56
Q8CB27	Yod1	3	1	1	1	343	37.5	5.76

Q9CQV4	Retreg3	2	1	1	2	466	51.6	4.97
Q8C079	Strip1	2	1	2	2	837	95.5	6.25
Q78T81	Fam102a	4	1	1	1	392	42.8	8.56
O54784	Dapk3	3	1	1	1	448	51.4	8.7
Q8BTR5	Dusp28	11	2	2	2	163	17.5	7.75
Q9R062	Gyg1	4	1	1	1	333	37.4	5.29
Q8VE88	Fam114a2	3	2	2	2	497	54	4.93
P49138	Mapkapk2	2	1	1	1	386	44	8.57
Q9DCU6	Mrpl4	7	2	2	2	294	33.1	9.82
Q9QYF1	Rdh11	4	1	1	1	316	35.1	8.91
Q8BW41	Pomgnt2	2	1	1	1	605	69.3	8.78
Q91WE1	Snx15	3	1	1	2	337	37.7	5.24
Q8K479	C1qtnf5	6	1	1	1	243	25.4	6.68
Q9JJF0	Nap1l5	8	1	1	1	156	17	4.32
Q8VEB4	Pla2g15	3	1	1	2	412	47.3	6.47
Q9JK81	Myg1	3	1	1	1	380	42.7	7.02
Q91WF7	Fig4	2	2	2	2	907	103.4	6.98
Q8BZ05	Arap2	1	2	2	2	1703	193.3	7.18
Q80Y55	Bsdc1	4	2	2	2	427	46.9	4.4
Q99J95	Cdk9	4	1	1	1	372	42.7	8.79
Q99N87	Mrps5	3	1	1	1	432	48.2	10.14
A2RSQ0	Dennd5b	1	2	2	2	1274	144.5	6.68
Q8CB77	Eloa	2	1	1	1	773	87.1	9.61
Q9JMA2	Qtrt1	3	1	1	1	403	44.1	7.68
P97314	Csrp2	7	1	1	1	193	20.9	8.62
Q8BVU5	Nudt9	4	2	2	3	350	38.6	6.76
Q9CPV9	P2ry12	3	1	1	2	347	39.4	9.58
Q61103	Dpf2	4	1	1	1	391	44.2	6.47
Q9JMG3	Tmub1	4	1	1	1	245	26.3	5.03
P06327	Gm5629	6	1	1	3	117	13	9.1
Q9CQ06	Mrpl24	6	1	1	1	216	24.9	9.5
Q9D1M0	Sec13	5	1	1	1	322	35.5	5.38
Q78PG9	Ccdc25	4	1	1	2	208	24.5	6.95
Q0V8T9	Cntnap5a	1	1	1	1	1304	145.6	6.28
Q9JI11	Stk4	4	2	2	2	487	55.5	5.19
Q9CXI3	Moxd1	3	1	1	1	613	69.6	6.67
Q9JII5	Dazap1	4	1	1	1	406	43.2	8.56
Q9CW46	Raver1	2	1	1	2	748	79.3	8.72
Q923D5	Wbp11	2	1	1	1	641	69.8	8.4
P21952	Pou3f1	3	1	1	1	449	45.3	7.74
P09925	Surf1	5	1	1	1	306	34.8	9.72

Q9DBH0	Wwp2	2	2	2	2	870	98.7	7.11
P55772	Entpd1	4	1	1	1	510	57.2	5.86
Q8CD92	Ttc27	2	1	1	1	847	96.4	5.77
Q8CI78	Rmnd1	3	1	1	1	450	51.8	7.99
P56818	Bace1	2	1	1	1	501	55.7	5.6
Q8CGQ8	Slc24a4	3	1	1	1	622	68.9	7.14
Q9Z2G9	Htatip2	5	1	1	2	242	26.9	8.59
Q9Z0Z4	Heph	1	1	1	1	1157	129.6	6.13
P60762	Morf4l1	4	1	1	1	362	41.5	9.32
Q9WTS5	Tenm2	1	1	2	2	2764	306.3	6.68
Q8CIM7	Cyp2d26	2	1	1	1	500	56.9	6.64
Q8BHS6	Armcx3	4	1	1	1	379	42.6	8.68
Q9JHK5	Plek	4	1	1	1	350	39.9	8.34
Q8R3D1	Tbc1d13	3	1	1	1	400	46.4	5.36
Q9D9E0	Slc22a17	2	1	1	1	401	43.2	8.73
Q9CQB5	Cisd2	10	1	1	1	135	15.2	9.51
Q9D1F4	Akt1s1	6	1	1	1	257	27.5	4.72
Q8CDM8	Fam160b1	2	1	1	1	764	86	5.25
Q62186	Ssr4	6	1	1	3	172	18.9	5.78
Q60928	Ggt1	1	1	1	1	568	61.5	7.15
Q922B9	Itprid2	1	1	1	1	1252	136.9	5.26
Q9Z1S3	Rasgrp1	2	1	1	2	795	90.2	8.1
Q9EPK2	Rp2	3	1	1	1	347	39.4	5.24
Q8BGX3	Lrtm2	4	1	1	1	370	41	6.4
Q6PD24	Ankrd13d	3	2	2	2	605	68	5.47
Q8BFQ4	Wdr82	2	1	1	2	313	35.1	7.69
Q80UP8	Slc20a2	2	1	1	1	656	70.8	6.48
Q8BKR5	Ppp1r37	2	1	1	1	712	77.5	5.06
Q07113	Igf2r	0	1	1	1	2483	273.6	5.71
Q6WKZ8	Ubr2	1	1	1	1	1755	199	6.33
Q9D074	Mgrn1	4	1	1	2	532	58.4	4.88
Q9DBY0	Foxp4	2	1	1	1	795	85.9	7.08
Q8R1V4	Tmed4	7	1	2	3	227	26	8.18
P62313	Lsm6	21	2	2	3	80	9.1	9.58
Q6A026	Pds5a	1	1	2	2	1332	150.2	7.85
Q3UQN2	Fcho2	1	1	1	1	809	88.7	6.89
Q9D2H5	Trim42	1	1	1	4	723	82.9	8.09
Q810B7	Slitrk5	1	1	1	1	957	107.1	6.93
Q61112	Sdf4	3	1	1	1	361	42	4.96
Q8K3G5	Vrk3	4	1	1	1	453	50.8	8.57
Q8BHH2	Rab9b	5	1	1	1	201	22.7	4.93
Q9D8B1	Aig1	3	1	1	2	262	29.7	8.07
Q3V384	Afg1l	1	1	1	2	480	54.3	8.03

Q91WM3	Rrp9	2	1	1	1	475	52.1	7.88
Q9WV95	Phlda3	8	1	1	1	125	13.7	9.67
P50153	Gng4	23	1	1	1	75	8.4	7.08
Q66JZ4	TCAIM	2	1	1	1	499	57.4	9.25
Q8VCA8	Scrn2	2	1	1	1	425	46.6	5.71
Q9JI78	Ngly1	2	1	1	2	651	74.2	6.89
Q3UUF8	Ankrd34b	3	1	1	1	508	55.4	8.02
Q9WTK3	Gpaa1	3	2	2	2	621	67.9	8.4
O35972	Mrpl23	9	1	1	1	146	17.1	9.76
O70480	Vamp4	9	1	1	1	141	16.3	7.36
Q9Z108	Stau1	3	1	1	1	487	53.9	9.52
Q6NS65	Gpr17	3	1	1	1	339	37.8	8.72
Q8R1K1	Ubac2	4	1	1	1	345	39	9.44
Q8BGK5	Slc35f1	2	1	1	1	408	45.3	7.43
Q9Z0W3	Nup160	1	1	1	1	1402	158.1	5.52
Q9CY57	Chtop	8	1	1	1	249	26.6	12.23
Q8VEK2	Rhbdd2	2	1	1	1	361	39.1	9.48
Q64327	Mea1	7	1	1	1	174	18.6	4.08
Q8BXK9	Clic5	4	1	1	1	251	28.3	5.94
P46662	Nf2	2	1	1	1	596	69.7	6.35
Q924Z4	Cers2	2	1	1	1	380	45	8.75
Q91WN1	Dnajc9	5	1	1	2	259	30	5.94
Q8K4F5	Abhd11	5	2	2	2	307	33.5	9.61
Q9QZN4	Fbxo6	4	1	1	1	295	34.5	8.18
P50429	Arsb	2	1	1	2	534	59.6	7.24
O35668	Hap1	2	1	1	1	628	70.1	4.74
Q8K284	Gtf3c1	0	1	1	1	2101	237.3	7.25
Q91VJ5	Pqbp1	5	1	1	1	263	30.6	6.23
Q8BGR2	Lrrc8d	2	1	1	1	859	98.1	7.44
P97447	Fhl1	4	1	1	2	280	31.9	8.37
Q62415	Ppp1r13b	1	1	1	1	1087	119.1	6.68
A6H6A9	Rabgap1l	1	1	1	1	815	92.3	5.31
Q64471	Gstt1	3	1	1	2	240	27.4	7.27
P97789	Xrn1	1	1	1	1	1719	194.2	7.5
Q8BHL8	Psmf1	5	1	1	1	271	29.6	5.25
Q8BG09	Tmem184b	4	2	2	2	407	45.6	7.37
Q8QZY9	Sf3b4	3	1	1	1	424	44.3	8.56
Q9D4F8	Tubgcp4	2	1	1	1	667	76.1	6.65
Q8K2F0	Brd3	2	1	1	1	726	79.7	9.36
Q8K2V6	Ipo11	1	1	1	1	975	112.3	5.26
Q7TSS2	Ube2q1	4	1	1	1	422	46.1	5.1
P56387	Dynlt3	9	1	1	1	116	12.9	5.12

Q9CY97	Ssu72	6	1	1	1	194	22.5	5.21
Q8R0F6	Ilkap	4	2	2	2	392	42.7	7.36
Q61554	Fbn1	0	1	1	1	2873	312.1	4.92
Q9ESU6	Brd4	1	1	1	1	1400	155.8	9.19
Q920R0	Als2	1	1	1	1	1651	182.5	6.3
O88307	Sorl1	1	1	1	1	2215	246.9	5.54
Q8K093	Trhde	1	1	1	1	1025	117.4	7.06
Q8R1E7	Tmem234	6	1	1	1	140	14.9	7.12
Q8BGR9	Ublcp1	5	2	2	2	318	36.8	6.46
Q99LG4	Ttc5	3	1	1	1	440	48.8	6.29
Q9Z2D8	Mbd3	5	1	1	1	285	32.1	5.82
Q6PIP5	Nudcd1	3	1	1	1	582	66.7	5.3
Q8VD26	Tmem143	3	1	1	1	458	51.6	9.26
P62315	Snrpd1	17	1	1	1	119	13.3	11.56
Q8K215	Lyrm4	15	1	1	1	91	10.8	10.13
Q61818	Rai1	1	1	1	1	1889	201.4	8.75
Q6PHZ5	Rbm15b	2	2	2	2	887	97	9.91
Q9CRT8	Xpot	2	1	1	1	963	109.7	5.25
Q8CGA3	Slc43a2	2	1	1	1	568	62.4	7.93
Q8R1G2	Cmbl	7	2	2	2	245	27.9	7.18
Q9D4H2	Gcc1	2	1	1	1	778	87.6	5.54
Q8K0V2	Dcun1d3	8	2	2	2	304	34.4	5.12
Q8BUV8	Gpr107	2	1	1	1	551	62	7.59
Q9DBE9	Ftsj3	2	1	1	1	838	95.5	8.38
P97785	Gfra1	3	2	2	3	468	51.7	7.84
P22437	Ptgs1	2	1	1	1	602	69	6.83
O70572	Smpd2	4	1	1	1	419	47.4	6.44
Q9EPL9	Acox3	2	1	1	1	700	78.4	7.12
P02802	Mt1	20	1	1	1	61	6	7.96
B9EHT4	Clip3	3	1	1	1	547	59.5	7.94
Q61550	Rad21	2	1	1	1	635	72	4.64
P50285	Fmo1	2	1	1	1	532	59.9	8.47
Q08943	Ssrp1	2	1	1	1	708	80.8	6.76
Q9CR39	Wdr45b	5	2	2	2	344	38	7.56
Q60759	Gcdh	4	1	1	1	438	48.6	8.73
Q9DCJ9	Npl	3	1	1	1	320	35.1	8.03
Q9R0C8	Vav3	1	1	1	1	847	97.9	7.06
Q80V91	Dtx3	3	1	1	1	347	38	8.73
Q9QXQ1	Pde7b	3	2	2	2	446	51.3	7.4
Q922M7		4	1	1	1	232	26	9.55
Q9DC37	Mfsd1	2	1	1	1	464	51.4	8.21
Q4VBD2	Tapt1	2	2	2	2	564	63.9	8.22

Q6ZWM4	Lsm8	10	1	1	1	96	10.4	4.48
Q8BYJ6	Tbc1d4	1	1	1	1	1307	147.4	7.33
P16254	Srp14	12	1	1	1	110	12.5	10.17
Q8BH10	Orai2	3	1	1	2	250	28.2	7.94
P36552	Cpox	2	1	1	1	443	49.7	8.53
P47867	Scg3	2	1	1	1	471	53.3	4.93
Q3UZP4	Svip	14	1	1	1	77	8.4	8.91
Q9R1Q7	Plp2	8	1	1	1	152	16.6	7.14
Q8BYM8	Cars2	3	1	1	1	551	61.2	8.18
O88829	St3gal5	3	1	1	2	414	47.3	8.51
O35127	Grcc10	10	1	1	1	126	13.2	5.14
Q6P5H6	Frmd5	3	1	1	1	517	58.5	8.69
Q9D4C9	Clvs1	5	1	1	1	354	40.6	6.73
Q07456	Ambp	4	1	1	1	349	39	6.32
Q8C650	Septin10	3	1	1	1	452	52.4	6.6
F7BWT7	Tspan15	3	1	1	1	294	33	5.97
P0C913		22	1	1	1	63	6.4	6.57
P62274	Rps29	14	1	1	2	56	6.7	10.13
Q8BGG7	Ubash3b	2	2	2	2	638	71.4	6.81
Q8R1Y2	Bmerb1	5	1	1	2	203	23.5	5.63
Q91W89	Man2c1	1	1	1	2	1039	115.6	6.51
Q8BGT1	Flrt3	2	1	1	1	649	72.8	8.06
Q8K2F8	Lsm14a	2	1	1	1	462	50.5	9.52
P48542	Kcnj6	5	1	1	1	425	48.6	5.38
Q811S7	Ubp1	2	1	1	2	540	60.2	6.27
Q80TS7	Ddn	2	1	1	1	710	76.4	9.98
Q9D0D3	Mtpap	2	1	1	1	585	65.2	8.88
Q8BWU5	Osgep	6	2	2	2	335	36.3	6.24
Q6DFY8	Brinp2	2	1	1	1	783	89.2	7.88
Q9Z0S9	Rabac1	8	1	1	1	185	20.6	7.9
Q05A62	Dnal1	13	2	2	2	190	21.5	5.76
B9EJ80	Pdzd8	1	1	1	2	1147	127.7	6.04
Q3THJ3	Eif1ad	9	1	1	1	170	19.5	4.87
Q8VHQ4	Rab40c	4	1	1	1	281	31.3	9.19
Q8R1C3	Vopp1	5	1	1	1	172	19.4	8.32
O88492	Plin4	4	1	1	1	1403	139.3	8.59
Q9CQF0	Mrpl11	6	1	1	1	192	20.7	9.73
O08545	Efna3	7	1	1	1	230	25.6	8.44
Q91YQ3	Csdc2	10	2	2	2	154	16.8	7.55
Q8BH69	Sephs1	3	1	1	2	392	42.9	5.97
Q9D428	GOLGA7B	5	1	1	1	167	18.3	6.01

Q8CFJ9	Wdr24	1	1	1	1	790	88.1	6.46
Q3TY86	Aifm3	4	2	2	2	605	66.8	9.04
A2BH40	Arid1a	0	1	1	1	2283	241.9	6.68
Q7TSG1	Cep120	1	1	1	1	988	112.5	5.94
Q8R4V2	Dusp15	6	1	1	1	235	26.2	9.26
Q5XKN4	Jagn1	7	1	1	1	183	21.1	9.8
Q2YDW2	Msto1	2	1	1	1	556	61.2	6.49
Q9CPZ8	Cmc1	9	1	1	1	106	12.5	8.24
Q99J09	Wdr77	4	1	1	1	342	36.9	5.27
Q9R1Z7	Pts	9	1	1	1	144	16.2	6.52
Q8BUY9	Pggt1b	3	1	1	1	377	42.3	6.83
P56213	Gfer	10	2	2	2	198	22.9	7.93
B1AS29	Grik3	2	1	1	1	919	104	7.52
P35294	Rab19	5	1	1	1	217	24.4	6.42
Q61285	Abcd2	1	1	1	1	741	83.4	9.09
Q5PR69	Crad	1	2	2	2	1207	132.2	5.4
Q5SVQ0	Kat7	2	1	1	1	613	70.6	8.72
Q8BFY6	Pef1	5	1	1	1	275	29.2	6.3
Q91YR5	EEF1AKNMT	2	1	1	1	698	78.7	6.83
P00416	mt-Co3	5	1	1	1	261	29.9	7.3
Q8R1A4	Dock7	1	1	1	1	2130	241.3	6.71
Q9DCG9	Trmt112	17	2	2	2	125	14.1	5.27
Q8R2Q4	Gfm2	1	1	1	1	779	86.1	6.43
Q9ERR7	Selenof	7	1	1	1	162	17.8	5.35
Q99LN9	Dohh	4	1	1	1	302	32.9	4.84
Q9D7A6	Srp19	17	1	1	1	144	16.2	9.96
Q149F1	Rpusd2	5	2	2	2	553	61.5	6.77
Q8BHN0	Ppm1l	2	1	1	1	360	41	5.99
Q63829	Commd3	6	1	1	1	195	22	5.59
Q8BUY8	Gprasp2	1	1	1	1	826	92.7	5.11
Q64282	Ifit1	3	1	1	1	463	53.7	7.52
Q505B7	Zbtb8os	5	1	1	1	168	19.7	4.56
Q8BHK1	Nipa1	4	1	1	1	323	34.1	8.37
Q9ERY9	Erg28	6	1	1	1	140	15.8	9.91
Q6ZPK7	Zfyve28	1	1	1	1	905	99.7	5.1
Q8BGT6	Micall1	1	1	1	1	870	94	6.68
Q8CA71	Shisa4	5	1	1	1	197	21.5	7.42
P17095	Hmga1	8	1	1	1	107	11.6	10.32
Q7TPD3	Robo2	1	1	1	1	1470	161.1	6.33
Q8K4L3	Svil	1	1	1	1	2170	243	6.87
Q8CIB6	Tmem230	10	1	1	3	120	13.2	9.31
Q8CBX0	Tmem63c	2	1	1	1	802	93	7.77

P54797	Tango2	5	1	1	1	276	30.9	5.1
O70145	Ncf2	2	1	1	1	525	59.4	6.61
O35604	Npc1	1	1	1	1	1277	142.8	5.71
Q3TYD4	Arsg	3	1	1	1	525	57.4	6.39
Q3UVU3	Slc30a10	2	1	1	1	470	50.9	7.02
Q8C190	Vps9d1	1	1	1	1	649	71.2	6.87
Q922V4	Plrg1	2	1	1	1	513	56.9	9.17
								11.3
P47964	Rpl36	10	1	1	1	105	12.2	4
Q7TMS5	Abcg2	2	1	1	1	657	72.9	8.54
P50586	Tub	2	1	1	1	505	55.3	8.68
Q9D6U8	Fam162a	11	1	1	1	155	17.7	9.88
Q9JI10	Stk3	2	1	1	1	497	56.8	4.98
P05366	Saa1	7	1	1	1	122	13.8	7.03
Q8BKX6	Smg1	0	1	1	1	3658	409.5	6.4
Q8VHL0	Slc14a1	2	1	1	3	384	42.1	7.9
Q91VR8	Brk1	12	1	1	1	75	8.8	5.45
Q9ES56	Trappc4	8	2	2	3	219	24.4	6.21
Q9D3P8	Plgrkt	7	1	1	2	147	17.3	9.5
Q8BZW8	Nhlrc2	2	1	1	1	725	78.4	5.54
Q8CGV2	Tph2	2	1	1	1	488	55.8	6.24
Q925J9	Med1	1	1	1	1	1575	167	8.73
Q8K341	Atat1	3	1	1	1	421	47.1	9.98
B2RR83	Ythdc2	1	1	1	1	1445	161	8.51
O89103	Cd93	1	1	1	1	644	69.3	5.07
Q8CBY0	Gatc	7	1	1	1	155	16.7	5.16
Q8BYB9	Poglut1	5	2	2	3	392	46.4	8.85
Q8BRU6	Slc18a2	2	1	1	1	517	55.7	6.23
P98156	Vldlr	1	1	1	1	873	96.3	4.78
Q9JKF7	Mrpl39	2	1	1	1	336	38.5	7.94
P11859	Agt	3	1	1	2	477	52	5.44
Q7TSH8	Tmem94	1	1	1	1	1360	151.7	6.46
Q8BHT6	B3glct	2	1	1	1	489	55.3	6.92
P97770	Thumpd3	2	1	1	1	505	56.4	7.43
P54818	Galc	1	1	1	2	684	77.2	6.74
O88653	Lamtor3	9	1	1	1	124	13.5	7.34
Q63918	Cavin2	4	1	1	1	418	46.7	5.21
Q8R4R6	Nup35	4	1	1	1	325	34.8	9.25
Q3UHH1	Zswim8	1	1	1	1	1832	196.9	6.84
Q9DAF3	Ddi1	2	1	1	4	408	45.7	6.18
Q9WVL2	Stat2	1	1	1	1	923	105.4	5.27
Q8CBH5	Mfsd6	3	2	2	2	775	86	6.06
Q60738	Slc30a1	2	1	1	1	503	54.7	6.62
E9Q555	Rnf213	0	1	1	1	5152	584.4	6.8

Q8VI51	Sorcs3	1	1	1	1	1219	135.9	6.49
O88662	Emp2	5	1	1	1	172	19.7	7.72
Q9D7S7	Rpl22l1	10	1	1	1	122	14.5	9.45
Q61743	Kcnj11	3	1	1	1	390	43.5	8.19
Q8R4V4	Cpz	2	1	1	1	654	73.7	8.05
Q8R0Y8	Slc25a42	3	1	1	1	318	35.2	10.05
Q8CHY3	Dym	1	1	1	1	669	75.8	5.77
Q8BX02	Kank2	2	2	2	2	843	90.2	5.55
Q9D1K7		8	1	1	1	174	19.5	6.68
Q689Z5	Sbno1	1	1	1	1	1390	153.6	8.07
Q9D8S9	Bola1	12	1	1	1	137	14.4	8.76
Q9ES74	Nek7	3	1	1	1	302	34.5	8.25
Q924D0	Rtn4ip1	4	1	1	1	396	43.3	9.2
P70671	Irf3	2	1	1	1	419	46.8	5.43
Q64213	Sf1	6	2	2	2	653	70.4	8.98
Q62193	Rpa2	3	1	1	1	270	29.7	6.15
Q3UH99	Shisa6	3	2	2	2	525	58.4	9.48
Q9CXE2	Bcl7a	4	1	1	1	210	22.8	5.06
Q3U1Y4	Dennd4b	1	1	1	1	1499	164.6	7.71
Q0PHV7	Dact3	2	1	1	1	610	63.2	10.42
Q9JLI6	Scly	3	1	1	1	432	47.1	6.8
P70362	Ufd1	3	1	1	2	307	34.5	6.7
P58802	Tbc1d10a	3	1	1	1	500	56.2	7.85
Q8R3N6	Thoc1	3	1	1	1	657	75.4	4.97
Q9QYK9	Pnck	3	1	1	1	343	38.5	6.47
Q9QZR0	Rnf25	2	1	1	1	456	51.2	6.29
A2AR02	Ppig	1	1	1	1	752	88.3	10.27
Q9CYW4	Hdhd3	4	1	1	1	251	28	6.8
Q80U93	Nup214	1	1	1	1	2085	212.8	7.08
Q6NXI6	Rprd2	1	1	1	1	1469	156.5	7.59
Q9D708	S100a16	10	1	1	1	124	14.3	5.86
Q9CWT3	Snx10	4	1	1	1	201	23.5	5.78
Q925N1	Sfxn4	3	1	1	1	313	35.6	9.45
Q9DBG7	Srpra	1	1	1	1	636	69.6	8.95
O55229	Chkb	3	1	1	1	394	45.1	5.41
Q52KI8	Srrm1	2	1	1	1	946	106.8	11.87
E9PV86	Mctp1	1	1	1	1	951	106.7	8.37
Q3ZK22	Vezt	1	1	1	1	780	87.9	5.2
Q921L3	Tmco1	5	1	1	1	188	21.2	9.74
O88507	Cntfr	3	1	1	1	372	40.8	6.83

Q80ZJ8	Cracr2b	2	1	1	1	394	44.4	5.2
Q8BYN3	Itpk1	3	1	1	1	419	46.1	6.29
Q8VD57	Sft2d2	6	1	1	1	159	17.5	9.09
Q91V24	Abca7	0	1	1	1	2159	236.7	7.28
P23475	Xrcc6	1	1	1	2	608	69.4	6.79
Q8K1E0	Stx5	2	1	1	1	355	39.7	8.92
Q9CZB0	Sdhc	17	1	1	1	169	18.4	9.94
O35709	Enc1	1	1	1	2	589	66.1	6.81
Q8CBC4	Cnst	2	1	1	1	711	76.8	4.59
P47758	Srprb	5	1	1	2	269	29.6	9.28
Q8BWW4	Larp4	2	1	1	1	719	79.7	6.39
Q99KD5	Unc45a	1	1	1	1	944	103.4	6.34
Q8BTJ4	Enpp4	2	1	1	1	456	51.6	6.73
Q60739	Bag1	2	1	1	1	355	39.7	8.46
Q5F2E7	Nufip2	2	1	1	1	692	75.6	8.7
O88455	Dhcr7	3	1	1	1	471	53.9	8.35
Q91W82	Ube2e2	7	1	1	1	201	22.2	7.71
Q8C3X4	Guf1	1	1	1	1	651	72.4	8.76
Q91X58	Zfand2b	3	1	1	1	257	27.9	6.93
P13808	Slc4a2	1	1	1	1	1237	136.7	6.14
Q9D2R6	Coa3	9	1	1	1	108	12	9.86
Q3U5F4	Yrdc	5	1	1	1	280	29.4	6.07
O88522	Ikbkg	2	1	1	1	412	47.9	5.85
Q3U3Q1	Ulk3	2	1	1	1	472	53.5	7.03
Q61687	Atrx	0	1	1	1	2476	278.4	6.68
Q9WU63	Hebp2	7	1	1	1	205	23	4.48
Q8BVF2	Pdcl3	5	1	1	1	240	27.6	4.72
Q9D387	Lamp5	4	1	1	1	280	31.7	6.14
Q8BND3	Wdr35	1	1	1	1	1181	133.9	6.34
Q9Z1T6	Pikfyve	1	2	2	2	2097	236.7	6.68
P97454	Smad5	2	1	1	1	465	52.1	7.71
Q8C739	Fam110b	2	1	1	1	366	40.3	9.23
Q99PL7	Scd3	2	1	1	2	359	41.4	8.94
Q8C5W3	Tbcel	4	1	1	1	424	48	5.53
Q8BXN9	Tmem87a	2	1	1	1	555	63.3	6.67
Q99N84	Mrps18b	4	1	1	1	254	28.7	8.35
Q8CE33	Klhl11	2	1	1	1	709	80.4	6.01
P58064	Mrps6	8	1	1	1	125	14.3	9.5
Q9QYK4	Hs6st3	2	1	1	1	470	55	7.01
Q3UD01	Atxn7l3b	10	1	1	1	97	10.7	4.32
Q8CF89	Tab1	2	1	1	1	502	54.6	5.52
Q8BJD1	Itih5	1	1	1	1	952	106.7	8.16
Q91VJ2	Cavin3	4	1	1	1	260	27.8	5.57
Q9QZD4	Ercc4	1	1	1	1	917	103.6	7.71

Q8C6I2	Sdhaf2	5	1	1	1	164	19.4	6.35
Q6P2L7	Casc4	3	2	2	2	435	49.4	5.45
Q9Z131	Sh3bp5	2	1	1	1	463	51.8	5.11
O88559	Men1	2	1	1	2	611	67.5	6.39
P30875	Sstr2	2	1	1	1	369	41.2	8.97
Q3TIU4	Pde12	3	2	2	2	608	67.5	6.92
Q91WM6	Eva1a	7	1	1	1	156	17.8	6.55
A2A8Z1	Osbpl9	1	1	1	1	736	83.1	6.07
P39087	Grik2	1	1	1	1	908	102.4	7.77
E9Q6B2	Ccdc85c	5	2	2	2	420	45.3	6.96
Q3UTZ3	MAP11	2	1	1	1	580	62.7	8.68
P01723		8	1	1	1	117	12.2	5.21
Q9D0I4	Stx17	5	1	1	1	301	33.2	6.74
P97412	Lyst	0	1	1	1	3788	425	6.6
Q91W53	Golga7	11	1	1	1	137	15.8	7.05
Q80W00	Ppp1r10	1	1	1	1	888	94.3	9.16
Q8K358	Pigu	2	1	1	1	434	49.8	7.72
Q9D924	Isca1	12	1	1	2	129	14.2	9.07
Q8K4M5	Commd1	5	1	1	1	188	21	7.59
Q99P47	Cntnap4	1	1	1	1	1310	144.6	7.06
Q8QZV7	IntS13	2	1	1	1	732	82.7	6.92
Q9D4J7	Phf6	2	1	1	1	364	41.1	8.73
P0C7M9	Clec2l	7	2	2	3	211	23.6	7.2
Q9D338	Mrpl19	4	1	1	1	292	33.6	9.44
P70697	Urod	3	1	1	1	367	40.7	6.65
Q8BGY3	Luzp2	3	1	1	1	345	39	8.5
Q6P3A8	Bckdhb	3	1	1	1	390	42.9	6.7
P97401	Frzb	3	1	1	1	323	36	8.27
P58158	B3gat3	6	2	2	2	335	37	8.78
Q91XE8	Tmem205	9	1	1	1	189	21.2	9.42
P59644	Inpp5j	1	1	1	2	1003	107.5	9.36
P05977	Myl1	4	1	1	1	188	20.6	5.03
Q8BP71	Rbfox2	4	1	2	2	449	47.3	6.55
Q3TWI9	Tmem63b	1	1	1	1	832	94.7	7.49
Q9QWH1	Phc2	1	1	1	1	850	89.7	8.75
Q3TRM4	Pnpla6	1	1	1	1	1355	149.4	7.83
Q9ERB5	Slco1c1	1	1	1	1	715	78.3	8.81
Q9Z0H1	Wdr46	3	1	1	1	622	69	9.77
B9EJI9	Tmem229a	4	1	1	1	371	41.5	9.35
Q9JKV5	Scamp4	5	1	1	1	230	25.3	8.66
Q6P4T1	Snx19	1	1	1	1	997	109.7	5.12
P62965	Crabp1	7	1	1	1	137	15.6	5.38
Q8CD91	Smoc2	3	1	1	1	447	49.9	8.51

Q8BMB3	Eif4e2	4	1	1	1	245	28.2	8.87
P26262	Klkb1	1	1	1	1	638	71.3	8.02
P58137	Acot8	3	1	1	1	320	35.8	7.64
Q9WUZ9	Entpd5	2	1	1	1	427	47.1	5.29
Q8VHV1	Baalc	5	1	1	2	145	15.5	7.06
Q9D799	Mtfmt	3	1	1	1	386	43.1	9.42
Q9D1L9	Lamtor5	11	1	1	1	91	9.6	4.87
Q9Z160	Cog1	1	1	1	1	980	109	7.18
Q80YE4	Aatk	1	1	1	1	1365	144.5	4.55
P20491	Fcer1g	10	1	1	1	86	9.6	7.97
Q99N94	Mrpl9	3	1	1	1	265	30.2	10.08
Q9ERF3	Wdr61	2	1	1	1	305	33.8	5.36
O88291	Znf326	2	1	1	1	580	65.2	5.19
Q8BKY8	Mterf2	3	1	1	1	385	43.4	8.94
Q6RKD8	Flrt1	2	1	1	1	646	71.4	6.55
P04940		7	1	1	1	107	11.6	9.36
P34928	Apoc1	8	1	1	2	88	9.7	9.09
Q80Y56	Rbsn	1	1	1	1	783	88.4	5.3
P49070	Camlg	2	1	1	1	294	32.5	7.74
P51175	Ppox	2	1	1	1	477	50.8	8.84
Q80XS6	Samd4b	2	1	1	1	687	75	6.74
Q6PGH1	Bud31	8	1	1	1	144	17	8.82
Q99MX0	Tktl1	2	1	1	1	595	65.2	6.02
Q64096	Mcf2l	1	1	1	1	1149	129	5.9
O35739	Klf9	6	1	1	1	244	27.2	8.57
Q8BKI2	Tnrc6b	1	1	1	1	1810	191.8	6.3
Q9D1B9	Mrpl28	5	1	1	1	257	30.2	9.29
Q8C0J6	Sowahc	3	1	1	1	512	54.9	6
Q8CE90	Map2k7	2	1	1	1	535	59.3	9.11
Q8BI08	Mal2	6	1	1	1	175	19.1	6.49
Q31125	Slc39a7	2	1	1	1	476	50.6	6.87
P97291	Cdh8	1	1	1	1	799	88.2	4.74
Q9JHI7	Exosc9	2	1	1	1	438	48.9	5.11
Q68FF0	Kiaa1841	2	1	1	1	718	81.9	6.62
Q80W37	Snupn	3	1	1	1	358	41	6.29
Q64704	Stx3	3	1	1	1	289	33.2	5.45
Q69Z66	Mysm1	1	1	1	3	819	93.4	5.29
Q8BGF6	Elmod2	3	1	1	1	293	34.7	8.57
P03893	Mtnd2	2	1	1	1	345	38.7	9.92
Q5U4F6	Wdr34	2	1	1	2	535	57.9	6.52
Q8K0G8	Esrp2	1	1	1	3	717	77.3	6.38
Q6ZPS6	Ankib1	1	1	1	1	1085	121.8	5.16
Q69Z38	Peak1	1	1	1	1	1735	191	6.87

P22907	Hmbs	3	1	1	1	361	39.3	6.87
Q6ZQH8	Nup188	1	1	1	1	1759	196.6	7.01
Q5U4D9	Thoc6	7	2	2	2	341	37.3	7.12
Q8BG94	Commd7	6	1	1	1	200	22.6	5.94
Q8CFV9	Rfk	6	1	1	1	155	17.4	7.55
Q3UFY8	Trmt10c	3	1	1	1	414	48.4	9.38
O54949	Nlk	2	1	1	1	527	58.3	8.13
Q8C9B9	Dido1	1	2	2	2	2256	247	7.91
Q3V1M1	Igsf10	0	1	1	1	2594	285.4	9.29
Q8C9S4	Ccdc186	1	1	1	1	917	104.8	5.53
P41241	Csk	2	1	1	1	450	50.7	7.06
Q9CQS8	Sec61b	10	1	1	2	96	10	11.56
Q8K1N1	Pnpla8	1	1	1	1	776	87.3	9.23
Q3UGP8	Alg10b	1	1	1	1	474	55.4	9.54
Q5Y5T1	Zdhhc20	3	1	1	1	380	43.9	8.25
P70261	Pald1	1	1	1	1	859	96.7	6.58
Q09014	Ncf1	3	1	1	1	390	44.6	9.01
Q99JN2	Klhl22	2	1	1	1	634	71.6	5.71
O35387	Hax1	3	1	1	2	280	31.6	4.91
P00520	Abl1	1	2	2	2	1123	122.6	8.65
Q9CQL5	Mrpl18	7	1	1	1	180	20.7	9.28
Q9WTX7	Fhl5	9	1	1	1	284	32.9	7.65
Q9DCU2	Pllp	9	1	1	1	182	19.8	9.41
Q8C2E4	Ptcd1	1	1	1	1	695	77.5	8.79
Q9CQ10	Chmp3	3	1	1	1	224	25.2	5.06
Q91WM1	Strbp	1	1	1	1	672	73.7	8.72
Q9DB32	Haghl	4	1	1	1	283	31.5	5.55
Q8BR07	Bicd1	1	1	1	1	835	95.8	5.71
B1AUE5	Pex10	5	1	1	1	324	37.1	9.94
Q9CRB0	Snx24	5	1	1	1	169	19.6	7.01
Q8BG16	Slc6a15	2	1	1	1	729	81.7	5.19
B2RWJ3	Tmem240	5	1	1	1	173	20	7.8
Q3UBX0	Tmem109	4	1	1	2	243	26.3	9.89
Q8BVU0	Lrch3	1	1	1	1	778	86.3	7.03
Q9ES89	Extl2	2	1	1	1	330	37.4	9.04
Q9CZV5	Supt7l	2	1	1	1	412	45.9	5.07
Q8CGA0	Ppm1f	2	1	1	1	452	49.6	5.3
Q99K95	Rtf2	4	1	1	1	307	33.9	8.81
Q64689	St8sia3	3	1	1	1	380	43.9	9.52
Q9D0B0	Srsf9	5	1	1	1	222	25.6	8.65
Q9D5J6	Shpk	3	1	1	1	476	51.3	6.24
Q8R3P6	Ints14	2	1	1	1	515	57.2	5.17
Q8R121	Serpina10	3	1	1	1	448	51.8	5.67

Q60603	Kcnh1	1	1	1	1	989	111.2	7.47	
Q9D7B1	Dus2	3	1	1	1	493	55.3	6.34	
Q8R1B5	Cplx3	4	1	1	1	158	17.6	4.89	
Q8R4X3	Rbm12	1	1	1	1	992	102.7	8.32	
Q8VE96	Slc35f6	3	1	1	1	372	41	7.14	
Q4VBE8	Wdr18	3	1	1	1	431	47.2	6.89	
P70193	Lrig1	1	1	1	1	1091	119.1	6.86	
Q9D8X2	Ccdc124	5	2	2	2	217	25.3	9.64	
Q5HZI2	C2cd4cC2CD4 family		3	1	1	1	419	44.6	9.73
Q80WV3	Chst2	2	1	1	1	530	57.8	9.99	
Q3TGF2	Fam107b	6	1	1	1	131	15.6	8.31	
Q99MR3	Slc12a9	1	1	1	3	914	96.3	7.02	
A2AB59	Arhgap27	1	1	1	1	869	97	5.94	
Q8BH15	Cnot10	1	1	1	2	744	81.8	7.68	
Q499E0	Brinp3	2	1	1	1	766	88.4	7.9	
P03958	Ada	2	1	1	1	352	40	5.72	
P09242	Alpl	2	1	1	1	524	57.5	7.01	
P32211	Chrm4	1	1	1	1	479	52.9	9.88	
P58929	Gmeb2	2	1	1	1	530	56.6	5.31	
Q66L42	Map3k10	1	1	1	1	940	103.1	6.87	
Q64345	Ifit3	2	1	1	1	403	47.2	5.64	
Q8VE95		4	1	1	1	218	24.3	8.7	
P42227	Stat3	2	1	1	1	770	88	6.3	
Q9JJ69	Kcnip2	3	1	1	1	270	30.9	5.05	
Q8BFR4	Gns	2	1	1	1	544	61.1	8.24	
P42128	Foxk1	1	1	1	1	719	74.9	9.17	
Q9CQ02	Commd4	6	1	1	1	199	21.8	6.67	
B8ZXI1	Qtrt2	2	1	1	1	415	46.3	6.16	
Q8CAL5	Gpc5	2	1	1	1	572	63.8	7.3	
P14847	Crp	6	1	1	1	225	25.3	6.2	
Q99LH2	Ptdss1	2	1	1	1	473	55.6	8.28	
P58242	Smpdl3b	2	1	1	1	456	51.6	6.43	
Q3V2J0	Fam92b	2	1	1	5	292	33.3	6.11	
Q8CD54	Piezo2	0	1	1	3	2822	325.4	5.66	
Q9QXW9	Slc7a8	1	1	1	1	531	57.8	6.67	
P03975	Iap	1	1	1	1	557	62.7	9.31	
Q3U0D9	Hace1	1	1	1	1	909	102	5.76	
P03921	Mtnd5	1	1	1	2	607	68.4	9.03	
Q3U213	Serac1	1	1	1	1	654	73.9	8.18	
Q8BH43	Wasf2	2	1	1	1	497	54	5.53	
O70200	Aif1	5	1	1	1	147	16.9	8.76	

P97490	Adcy8	1	1	1	1	1249	140	6.93
Q7TSK2	Sez6	1	1	1	1	991	107.4	5.36
Q3TC33	Ccdc127	4	1	1	1	260	30.5	9.13
Q8R3K3	Ptcd2	3	1	1	1	381	43.8	9.04
Q9Z224	Mocs2	9	1	1	2	88	9.7	4.61
Q8CDD9	Lrif1	1	1	1	1	755	82.9	9.5
Q80XD1	Chn2	4	1	1	1	332	38.2	8.09
Q8JZX4	Rbm17	2	1	1	2	405	45.3	5.82
Q9EST4	Psmg2	3	1	1	2	264	29.5	6.05
Q6NZL6	Tonsl	1	1	1	1	1363	151	5.97
Q9CR30	Josd2	6	1	1	1	188	20.8	6.54
P54254	Atxn1	1	1	1	1	791	83.7	8.16
P24452	Capg	2	1	1	1	352	39.2	7.2
P52432	Polr1c	3	1	1	1	346	39.1	5.21
Q8R3C0	Mcmbp	2	1	1	1	642	72.8	5.66
Q8R0K9	E2f4	2	1	1	1	410	43.8	4.75
P61211	Arl1	9	1	1	2	181	20.4	5.72
O35684	Serpini1	3	1	1	1	410	46.3	4.72
O70400	Pdlim1	3	1	1	1	327	35.8	6.84
Q9CQ89	Cuta	8	1	1	1	177	18.9	6.77
Q99M51	Nck1	3	1	1	1	377	42.9	6.47
P47879	Igfbp4	4	1	1	2	254	27.8	7.17
Q5FW52	Mlip	4	1	1	1	269	29.4	6.25
Q9R1E6	Enpp2	1	1	1	1	862	98.8	7.27
O35963	Rab33b	3	1	1	1	229	25.8	7.69
Q91X52	Dcxr	7	1	1	1	244	25.7	7.31
Q5DU41	Lrrc8b	1	1	1	1	803	92.1	6.7
Q8BK63	Csnk1a1	2	1	1	1	337	38.9	9.57
Q8K2M0	Mrpl38	2	1	1	1	380	45	8.1
Q8K2C8	Gpat4	2	1	1	1	456	52.1	9.35
Q6P8M1	Tatdn1	3	1	1	1	295	33.3	6.18
Q8R143	Pttg1ip	4	1	1	1	174	20	8.63
Q3TPE9	Ankmy2	2	1	1	1	440	48.7	6.67
Q8BQU6	Gjc2	2	1	1	1	440	47	7.12
A3KGB4	Tbc1d8b	1	1	1	2	1114	127.8	5.49
Q9Z1R3	ApoM	4	1	1	1	190	21.3	6.52
P01674		11	1	1	1	108	11.7	6.27
Q8C0S1	Dis3l	1	1	1	1	1053	120.2	6.81
Q6GQW0	Btbd11	1	1	1	1	1109	121.5	6.81
Q149G0		4	1	1	3	166	18.6	7.01
Q99JG2	Gpr37l1	2	1	1	1	481	52.7	7.36
Q61474	Msi1	3	1	1	1	362	39.1	7.87
Q8C6G8	Wdr26	2	1	1	1	641	70.5	6.16
Q9WUH1	Tmem115	5	1	1	1	350	38.1	7.5

Q9ESP1	Sdf2l1	4	1	1	1	221	23.6	7.42
Q8BGN5	Nipal3	2	1	1	1	410	44.8	7.75
Q8K448	Abca5	1	1	1	1	1642	185.8	7.2
P98191	Cds1	2	1	1	1	461	52.8	7.72
Q3UP75	Ugt3a1	1	1	1	2	523	59.7	7.34
P09470	Ace	1	1	1	1	1312	150.8	6.55
Q8CG03	Pde5a	1	1	1	1	865	98.3	6.05
O09117	Sypl1	3	1	1	1	261	28.9	8.79
Q9D071	Mms19	1	1	1	1	1031	113	6.21
Q6PDN3	Mylk	1	1	1	1	1941	212.8	6.25
P70170	Abcc9	0	1	1	1	1546	174.1	7.47
Q924Z6	Xpo6	1	1	1	1	1125	128.6	6.37
P28662	Bri3	6	1	1	1	125	13.6	8.41
O35657	Neu1	2	1	1	1	409	44.6	6.02
Q99MU3	Adar	1	1	1	1	1178	130.4	8.7
Q9CRC0	Vkorc1	8	1	1	1	161	17.8	9.14
Q8CI59	Steap3	2	1	1	1	488	54.7	9.22
Q3V4B5	Commd6	10	1	1	2	87	9.8	5.38
O70209	Pdlim3	3	1	1	2	316	34.3	7.91
Q60953	Pml	1	1	1	1	885	98.2	5.63
Q3UZ39	Lrrfip1	2	1	1	1	729	79.2	4.82
P70158	Smpdl3a	2	1	1	1	445	49.8	6.46
Q8BYK5	Phactr3	2	1	1	1	558	62.6	8.31
Q8VHI3	Pofut2	2	1	1	1	429	49.4	6.48
Q8R554	Otud7a	2	1	1	1	926	100.7	8.22
A6H5Y3	Mtr	1	1	1	1	1253	139	5.57
Q6ZQA6	Igsf3	1	1	1	1	1194	134.6	6.05
Q920N2	Hlcs	1	1	1	1	722	78.5	5.34
Q8BWU8	Etnppl	3	1	1	1	499	55.5	6.93
P62838	Ube2d2	5	1	1	1	147	16.7	7.83
Q9CWU9	Nup37	3	1	1	1	326	36.7	6.09
Q8BJ64	Chdh	1	1	1	1	596	66.4	8.51
Q61409	Pde3b	1	1	1	1	1100	122.1	5.71
P61971	Nutf2	11	1	1	1	127	14.5	5.38
Q8K353	Cystm1	10	1	1	1	104	11.4	4.32
Q61660	Foxj1	2	1	1	1	421	45.4	5.12
Q8BH50		4	1	1	1	245	26.4	5.19
Q6P2K6	Ppp4r3a	1	1	1	1	820	93.8	4.89
Q6P5B0	Rrp12	1	1	1	1	1295	143	8.91
P70274	Selenop	2	1	1	1	380	42.7	7.09
P27048	Snrpb	3	1	1	1	231	23.6	10.9
Q5PR68	Cep112	1	1	1	1	954	112.6	6.8
Q8C0Q2	Zhx3	1	1	1	1	951	104.3	6.57
Q9DBU3	Riok3	3	1	1	1	519	58.7	5.69

Q8BR65	Suds3	5	1	1	1	328	38.1	5.66
O35955	Psmb10	4	1	1	2	273	29	6.87
A2AJL3	Fggy	2	1	1	1	552	60.3	6.42
Q8CGE9	Rgs12	1	1	1	1	1381	149.5	7.59
P62843	Rps15	8	1	1	2	145	17	10.39
Q571F8	Gls2	1	1	1	1	602	66.3	7.66
O54990	Prom1	1	1	1	1	867	97.1	6.68
Q925T6	Grip1	1	1	1	1	1127	122	6.55
Q61739	Itga6	1	1	1	1	1091	122.1	7.03
P41778	Pbx1	3	1	1	1	430	46.6	7.03
Q9CZ69	Cmtm6	5	1	1	1	183	19.8	6.29
Q60857	Slc6a4	3	1	1	1	630	70	6.18
Q8VI59	Pcnx3	0	1	1	1	2028	221.4	6.62
Q9CWW6	Pin4	10	1	1	1	131	13.8	9.77
P42337	Pik3ca	1	1	1	1	1068	124.3	7.15
Q6NS82	Retreg2	2	1	1	2	541	57.5	4.46
Q3U1V8	Map3k9	1	1	1	1	1077	118.7	5.86
Q3U308	Ctu2	4	1	1	1	514	56.1	7.08
Q80WV7	Srrm3	1	1	1	1	648	71.1	11.55
Q9WV68	Decr2	5	1	1	1	292	31.3	8.81
Q9D2R0	Aacs	1	1	1	1	672	75.2	6.71
Q3UGR5	Hdhd2	3	1	1	1	259	28.7	6.05
Q62179	Sema4b	2	1	1	1	823	91.3	8.15
P62257	Ube2h	8	1	1	1	183	20.6	4.67
Q6P6M7	Sepsecs	1	1	1	1	504	55.3	8.06
Q8BG21	Cacfd1	5	1	1	1	171	18.3	5.44
Q8BTW3	Exosc6	9	1	1	1	273	28.4	6.11
Q78RX3	Smim12	10	1	1	1	92	10.8	9.04
Q9QY33	Tspan3	3	1	1	1	253	28	5.86
Q80YE7	Dapk1	1	1	1	1	1442	161.3	6.89
Q99KN2	Ciao1	3	1	1	1	339	37.6	4.88
Q6PDJ6	Fbxo42	1	1	1	1	717	77.7	7.58
P63166	Sumo1	12	1	1	1	101	11.6	5.52
P39429	Traf2	2	1	1	1	501	56	6.96
Q80W47	Wipi2	2	1	1	1	445	48.4	5.86
Q6PFX7	Nyap1	1	1	1	1	833	87.6	9.48
Q9Z1X2	Ptdss2	1	1	1	1	473	55	7.09
A2AHL1	Ano3	1	1	1	1	981	114.5	8.87
Q8K2G4	Bbs7	1	1	1	1	715	80.3	5.81
P50428	Arsa	2	1	1	1	506	53.7	5.87
E9Q9D5	Rabl2	4	1	1	1	223	25.6	5.66
Q9CU65	Zmym2	1	1	1	1	1376	154.5	6.37

Q923D4	Sf3b5	17	1	1	1	86	10.1	6.35
P49817	Cav1	6	1	1	1	178	20.5	6.02
P62305	Snrpe	13	1	1	1	92	10.8	9.44
Q3V140	Acrbp	1	1	1	1	540	61.1	4.98
Q3TZX3	Slc25a33	5	1	1	1	320	35	9.67
O88427	Cacna1h	0	1	1	1	2365	261.9	6.92
P97930	Dtymk	3	1	1	1	212	23.9	7.9
O70404	Vamp8	8	1	1	1	101	11.4	8.19
Q3URS9	Ccdc51	2	1	1	1	406	45.1	8.09
Q8K4L6	Mepe	7	1	1	1	441	46.8	9.14
Q9DC04	Rgs3	1	1	1	1	966	106.2	5.86
Q5ND34	Wdr81	1	1	1	1	1934	211.8	5.77
Q8K3A9	Mepce	2	1	1	1	666	72	9.25
Q08879	Fbln1	1	1	1	1	705	78	5.16
Q7TMY7	Ipo8	1	1	1	1	1010	117	5.16
Q8BGN2	D3Ertd751e	4	1	1	1	192	22.4	5.58
Q8BIL5	Hook1	1	1	1	1	728	84.4	5.22
Q9CWG8	Ndufaf7	3	1	1	1	436	48.4	6.95
P42866	Oprm1	3	1	1	1	398	44.4	8.25
Q8VDZ4	Zdhhc5	2	1	1	1	715	77.5	9.01
Q8CB67	Lrp11	3	1	1	1	483	51.8	7.78
Q8K2P7	Slc38a1	2	1	1	1	485	53.8	7.08
Q9JLK7	Cabp1	3	1	1	1	227	25.9	4.93
Q61214	Dyrk1a	1	1	1	1	763	85.4	8.75
Q6PE01	Snrnp40	2	1	1	1	358	39.3	8.1
O54940	Bnip2	2	1	1	1	326	37.7	4.97
P97372	Psme2	3	1	1	1	239	27	5.76
Q61205	Pafah1b3	5	1	1	1	232	25.8	6.93
Q922J9	Far1	2	1	1	1	515	59.4	9.19
P56671	Maz	1	1	1	1	477	48.7	8.95
Q61210	Arhgef1	2	1	1	1	920	102.7	5.6
Q8CDV6	Ccdc63	4	1	1	1	558	65.2	9.04
Q61239	Fnta	2	1	1	2	377	44	4.93
P61963	Dcaf7	2	1	1	2	342	38.9	5.52
Q99NH2	Pard3	1	1	1	1	1333	149	7.94
Q8CDG1	Piwil2	2	2	2	4	971	109.4	8.97
P60853	Lzts1	2	1	1	1	599	67.2	7.56
Q78T54	Vma21	12	1	1	1	101	11.4	7.24
Q8VDB8	Lrrc2	2	1	1	1	371	42.9	6.93
Q91X51	Gorasp1	3	1	1	1	446	46.9	4.65
Q5SSH7	Zzef1	1	2	2	2	2924	328.1	6.11
Q9CQC8	Spg21	3	1	1	1	308	34.9	6.28
Q9R0S3	Mmp17	3	1	1	1	578	64.3	6.38

O70496	Clcn7	1	1	1	1	803	88.7	7.27
Q03958	Pfdn6	6	1	1	2	127	14.4	8.88
Q9CWQ0	Dph5	3	1	1	1	281	31.2	5.29
Q8BHY8	Snx14	1	1	1	1	964	111.8	6.83
Q9QXM1	Jmy	1	1	1	1	983	110.5	6.13
O55028	Bckdk	2	1	1	1	412	46.6	8.91
Q00899	Yy1	3	1	1	1	414	44.7	6.29
Q8VEB1	Grk5	2	1	1	1	590	67.7	8.19
Q640M6	Gdpd5	1	1	1	1	607	68.8	8
Q80V26	Impad1	2	1	1	1	356	38.6	6.47
P48543	Kcnj9	2	1	1	1	393	43.9	5
Q9D1E8	Agpat5	2	1	1	1	365	42.2	9.38
Q80W22	Thnsl2	1	1	1	1	483	54.2	6.39
Q61247	Serpinf2	3	2	2	2	491	54.9	6.3
E9Q942	Smim13	9	1	1	2	88	9.9	6.27
Q9JJI8	Rpl38	14	1	1	1	70	8.2	10.1
Q9JK23	Psmg1	3	1	1	1	289	33.1	6.37
Q8BTN6	Leng9	1	1	1	1	485	52.6	5.83
Q99PV8	Bcl11b	1	1	1	1	884	94.5	6.62
P14069	S100a6	9	1	1	1	89	10	5.48
Q80W03	Tox3	1	1	1	1	575	63.1	7.59
Q9QYM9	Tmeff2	2	1	1	1	374	41.4	5.15
Q6QI06	Rictor	1	1	1	1	1708	191.4	7.23
Q62010	Ovgp1	1	1	1	1	721	78.8	9.19
Q69ZP3	Pnkd	3	1	1	1	385	43	8.94
Q80ZK0	Mrps10	8	1	1	1	160	18.7	8.84
Q9QWK4	Cd5l	2	1	1	1	352	38.8	5.16
Q9CR27	Washc3	4	1	1	1	194	21.1	4.46
Q8K182	C8a	1	1	1	1	587	66	6.54
O35304	Slc18a3	2	1	1	1	530	56.6	6.01
Q9D7H3	RtcA	3	1	1	1	366	39.2	7.9
Q9QUM7	Msh5	1	1	1	1	833	92.5	5.95
Q9Z1M0	P2rx7	2	1	1	1	595	68.3	8.22
Q8CAE9	Podxl2	2	1	1	1	603	64.9	4.36
P51569	Gla	3	1	1	1	419	47.6	5.72
O35386	Phyh	4	1	1	1	338	38.6	7.53
Q99JP6	Homer3	2	1	1	1	356	39.7	5.36
O08992	Sdcbp	4	1	1	1	299	32.4	7.15
Q5S006	Lrrk2	0	1	1	1	2527	284.5	6.83
Q03249	Galt	3	1	1	1	379	43.2	6.77
Q8BUE4	Aifm2	2	1	1	1	373	40.6	8.98
Q9CWL8	Ctnnbl1	1	1	1	1	563	64.9	5.07
Q8R516	Mib2	1	1	1	1	973	105.9	8.09
Q9CYU6	Dph7	3	1	1	1	477	53.2	6.33

Q80TT8	Cul9	0	1	1	1	1865	209	5.63
Q76M72	Slc22a27	1	1	1	1	551	62	8.73
Q8K157	Galm	4	1	1	1	342	37.8	6.74
O89050	Mkln1	1	1	1	1	735	84.8	6.34
Q8BGV7	Kctd13	4	1	1	1	329	36.4	6.99
Q91ZV7	Plxdc1	2	1	1	1	500	55.6	6.62
Q8BXV2	Bri3bp	4	1	1	1	253	28.2	9.52
Q91VU7	Pus7	2	1	1	1	660	74.7	5.87
Q3U0S6	Rasip1	1	1	1	1	961	103.5	7.83
Q9CQA1	Trappc5	5	1	1	1	188	20.8	9.66
Q9Z0S6	Cldn10	4	1	1	1	231	24.7	7.8
P19182	Ifrd1	2	1	1	1	449	49.9	7.18
Q9WVD4	Clcn5	1	1	1	1	746	83	7.01
E9Q414	Apob	0	1	1	1	4505	509.1	6.81
P06802	Enpp1	1	1	1	1	906	103.1	6.6
Q91YN0	D6Wsu163e	2	1	1	1	552	63.6	6.18
Q9D7V9	Naaa	2	1	1	1	362	40.1	6.46
Q8CEC5	Nkiras1	4	1	1	1	192	21.6	7.33
Q3ZT31	Snx25	1	1	1	1	840	97.1	6.29
O88630	Gosr1	4	1	1	1	250	28.5	9.29
A3KGF9	Ccdc9b	1	1	1	1	545	58.7	9.55
Q8R332	Nup58	1	1	1	1	587	59.4	9.29
Q5XG73	Acbd5	2	1	1	1	508	56.6	5.41
Q923M0	Ppp1r16a	1	1	1	1	524	57.5	5.81
Q91YT2	Rnf185	5	1	1	1	192	20.5	6.52
P52795	Efnb1	2	1	1	1	345	37.8	9.03
Q8BQ48	Cep295	0	1	1	1	2412	272.6	6.06
Q9CVI2	Fam133b	3	1	1	1	245	27.9	10.07
Q5U464	Asap3	1	1	1	1	904	99.2	6.83
Q8K135	Kiaa0319l	1	1	1	1	1048	115.2	6.16
Q920L1	Fads1	2	1	1	1	447	52.3	9.29
Q91ZU1	Asb6	2	1	1	1	418	46.2	5.78
C3VPR6	Nlrc5	0	1	1	1	1915	211.6	6.38
Q9D009	Lipt2	4	1	1	1	231	24.9	8.6
Q9CW07	Ppp1r3g	2	1	1	1	347	37.8	5
Q91ZW2	Pofut1	2	1	1	1	393	44.7	8.41
Q8R1Q9	Rbks	2	1	1	1	323	34.1	5.47
Q8K3I4	Myrip	1	1	1	1	856	94.9	6.07
Q5GH67	Xkr4	1	1	1	2	647	71.5	8.03
Q6PAV2	Herc4	1	1	1	1	1057	118.3	6.29
Q8VDP6	Cdipt	3	1	1	2	213	23.6	8.27

Q6PE87	Cfap206	1	1	1	2	622	70.9	6.77
O35710	Noct	2	1	1	1	429	48.3	7.27
Q69ZT1	Fan1	1	1	1	1	1020	112.9	7.44
P06328	Ighv1-72	8	1	1	1	117	12.9	9.29
P59823	Il1rapl1	1	1	1	1	695	79.6	6.32
Q61324	Arnt2	1	1	1	1	712	77.9	6.71
Q99PW4	Tp53rk	3	1	1	1	244	27.4	9.23
Q9JHZ2	Ankh	2	1	1	1	492	54.3	7.88
Q8R480	Nup85	1	1	1	1	656	74.7	5.57
Q8K2W3	Txndc11	1	1	1	1	948	105.9	7.88
E9Q1P8	Irf2bp2	2	1	1	1	570	59.3	8.69
Q9JJA9	Grasp	3	1	1	1	392	42.3	9.1
Q9ERN0	Scamp2	4	1	1	1	329	36.4	6.32
Q60611	Satb1	1	1	1	1	764	85.8	6.54
P61329	Fgf12	4	1	1	1	243	27.4	9.96
Q80V03	Adck5	2	1	1	1	582	66.6	8.76
Q9DBL2	Gdap2	2	1	1	1	498	56.2	5.39
P47759	Nsg2	5	1	1	1	171	19	9.39
Q9D9H8		2	1	1	1	365	41.7	7.87
Q99J27	Slc33a1	2	1	1	1	550	61	8.16
Q9DB98	Leng1	3	1	1	1	261	30.5	9.95
O88904	Hipk1	1	1	1	1	1210	130.6	8.22
Q9Z2V6	Hdac5	1	1	1	1	1113	120.9	6.2
P56528	Cd38	4	1	1	1	304	34.4	8.29
Q3UHF7	Hivep2	0	1	1	1	2430	266.5	6.96
Q99KR3	Lactb2	3	1	1	1	288	32.7	6.33
Q61235	Sntb2	2	1	1	1	520	56.3	8.69
Q91YI1	Atg13	2	1	1	2	516	56.4	5.26
Q61206	Pafah1b2	4	1	1	1	229	25.6	5.92
P70271	Pdlim4	2	1	1	1	330	35.5	8.05
Q8VCE6	Nt5m	4	1	1	1	220	25.6	8.35
Q8CFI2	Cdc34	5	1	1	1	235	26.6	4.56
P52187	Kcnj12	2	1	1	1	427	48.4	5.94
Q8C570	Rae1	2	1	1	1	368	40.9	7.83
Q60722	Tcf4	1	1	1	1	670	71.6	7.17
Q811C2	Atg4c	2	1	1	1	458	52	6.43
Q9Z1R4	D17h6s53e	3	1	1	1	293	32	6.89
Q9CRA8	Exosc5	7	1	1	1	235	25.2	7.56
Q91XB0	Trex1	3	1	1	1	314	33.7	7.99
Q9JHR9	Nrip2	3	1	1	1	270	29.3	7.68
Q8CGS6	Polq	0	1	1	1	2544	280.5	7.34
B2RRE7	Otud4	1	1	1	1	1107	123	6.76
Q8R3H7	Hs2st1	2	1	1	1	356	41.8	8.57

Q99LI7	Cstf3	1	1	1	1	717	82.8	8.12
Q8CHE4	Phlpp1	1	1	1	1	1687	182.3	6.19
Q8K0V4	Cnot3	1	1	1	1	751	81.9	6.2
Q91VM3	Wdr45	3	1	1	1	360	39.8	6.99
Q6PFE7	Tmeff1	2	1	1	1	372	40.1	6.81
Q91ZI0	Celsr3	0	1	1	1	3301	358.3	6.6
P97499	Tep1	0	1	1	1	2629	291.3	7.24
Q8JZN7	Rhot2	2	1	1	1	620	69	6
Q5BL07	Pex1	1	1	1	1	1284	141.3	6.35
Q3URU2	Peg3	0	1	1	1	1571	178.8	5.45
Q3TQB2	Foxred1	1	1	1	1	487	54.1	7.53
Q9CR25	Dph2	2	1	1	1	489	52.3	5.53
Q9QXA5	Lsm4	5	1	1	1	137	15.1	10.05
P07361	Orm2	4	1	1	1	207	23.8	5.45
A6PWD2	Fhad1	1	1	1	1	1420	163.6	6.64
Q9Z0U0	Xpr1	1	1	1	1	695	81.7	8.24
Q9D8M3	Slc48a1	8	1	1	1	146	16.5	9.45
P0C605	Prkg1	2	1	1	1	671	76.3	6.04
Q8VBW1	Slc6a8	1	1	1	1	640	71	6.61
Q9D4V0	Etnk1	3	1	1	1	363	42	5.44
Q9CQ40	Mrpl49	4	1	1	1	166	19.1	9.5
Q9CY45	Eef1akmt1	3	1	1	1	214	24.5	4.67
P70694	Akr1c6	2	1	1	1	323	37	8.28
Q64373	Bcl2l1	4	1	1	1	233	26.1	4.94
Q8BFR6	Zfand1	3	1	1	2	268	30.2	8.1
P63032	Rasd2	3	1	1	1	266	30.2	9.01
Q9ES34	Ube3b	1	1	1	1	1070	122.7	8.32
Q91V64	Isoc1	4	1	1	1	297	32	7.39
Q8VBX0	Asb13	3	1	1	1	278	29.9	6.67
Q9QZI9	Serinc3	1	1	1	1	472	52.6	7.23
Q62132	Ptprr	1	1	1	1	656	74	7.81
Q9EPQ2	Rpgrip1	1	1	1	1	1331	151.9	4.94
Q9DCT5	Sdf2	4	1	1	1	211	23.1	7.33
Q52KG4	Zbtb45	2	1	1	1	520	55	7.01
Q91ZR2	Snx18	1	1	1	1	614	67.9	6.67
Q80XQ2	Tbc1d5	1	1	1	1	815	91.8	6.79
Q9WUD8	Faim	4	1	1	2	179	20.2	5.64
Q6TDU8	Casc1	1	1	1	1	730	84.9	5.38
Q8CHK4	Kat5	2	1	1	1	513	58.6	8.48
Q7TSH7	Kcnf1	2	1	1	1	493	55.6	6.06
Q03391	Grin2d	1	1	1	1	1323	142.9	8.37
O09106	Hdac1	2	1	1	1	482	55	5.48

P97364	Sephs2	2	1	1	1	452	47.8	6.06
Q9JI59	Jam2	2	1	1	1	298	33	8.4
Q9R1S8	Capn7	1	1	1	1	813	92.5	7.97
O70451	Slc16a7	1	1	1	1	484	52.6	9.31
Q9CR89	Ergic2	2	1	1	1	377	42.5	6.89
Q3V1L4	Nt5c2	1	1	1	1	560	64.8	6.21
Q9DA08	Sgf29	4	1	1	1	293	33.3	8.1
Q5XPI3	Rnf123	1	1	1	1	1314	148.6	6.7
Q9D6G9	Cmtm5	8	1	1	1	156	17.4	4.84
Q8K1C9	Lrrc41	2	1	1	1	807	88.1	8.31
Q3TUF7	Yeats2	1	1	1	2	1407	148.9	9.04
Q80Y19	Arhgap11a	1	1	1	1	987	108.4	8.81
Q8C0D4	Arhgap12	1	1	1	1	838	95.3	7.59
O35671	Itgb1bp1	4	1	1	1	200	21.6	6.54
P63147	Ube2b	8	1	1	1	152	17.3	5.01
Q8BYI6	Lpcat2	2	1	1	1	544	60.2	5.83
Q8BS40	Cptp	3	1	1	1	216	24.6	6.74
Q9D7E3	Ovca2	3	1	1	1	225	24.2	5.88
Q8JZS9	Mrpl48	5	1	1	1	211	23.9	9.44
Q99LJ7	Rcbtb2	2	1	1	1	551	60.1	5.49
O08747	Unc5c	1	1	1	1	931	103	6.01
Q7TPQ3	Shprh	0	1	1	1	1674	191.4	7.46
Q9Z2B2	Slc25a14	3	1	1	1	325	36.3	9.69
Q9Z1B5	Mad2l1	3	1	1	1	205	23.6	5.2
Q8BJE2	Btnl9	3	1	1	1	536	60.5	6.42
O89023	Tpp1	1	1	1	1	562	61.3	6.57
E9Q7D5	Arhgef5	1	1	1	1	1581	176.6	5.83
Q6Y5D8	Arhgap10	1	1	1	1	786	89.3	7.18
Q80U57	Rims3	3	1	1	1	307	32.6	9.29
Q5FW53	Mybphl	2	1	1	1	355	38.7	8.32
P70303	Ctps2	1	1	1	1	586	65.5	6.49
Q9D023	Mpc2	6	1	1	1	127	14.3	10.61
Q8VDS8	Stx18	2	1	1	1	334	38.4	5.77
Q8BWB6	Steap2	5	1	1	1	489	55.7	9.2
P46412	Gpx3	6	1	1	1	226	25.4	8.22
P62046	Lrch1	2	1	1	1	709	79	6.13
P50608	Fmod	2	1	1	2	376	43	6.04
P19785	Esr1	2	1	1	2	599	66.9	8.06
O70361	Per3	1	1	1	2	1113	120.8	6.43
Q80WP8	Gadl1	2	1	1	1	550	62.5	6.96
Q5U4C9	Dyrk2	1	1	1	3	599	66.5	9.67
P61961	Ufm1	8	1	1	1	85	9.1	9.31

Q6DFW0		2	1	1	1	481	54.2	6.09
Q9Z0L0	Tpbg	2	1	1	1	426	46.4	6.83
Q3UM29	Cog7	1	1	1	1	770	86	5.38
O08648	Map3k4	1	1	1	1	1597	179.7	6.43
C0HK79	Arxes1	8	1	1	2	180	20.1	9.6
P70403	Cux1	1	1	1	1	678	77.2	5.35
Q9QXA7	Trim44	2	1	1	1	346	38.2	4.18
Q9JKQ4	Irx5	1	1	1	1	484	50.7	6.42
Q9D552	Spata17	2	1	1	1	379	45.5	9.67
Q8BFX3	Kctd3	1	1	1	1	815	88.8	7.15
Q9JMG1	Edf1	7	1	1	1	148	16.4	9.99
Q9D9Z5	Dda1	12	1	1	1	102	11.7	8.68
Q80XI6	Map3k11	3	1	1	1	850	93.2	8.43
Q91WP0	Masp2	1	1	1	1	685	75.5	6.07
Q61043	Nin	1	1	1	1	2113	243.6	5
P97479	Myo7a	0	1	1	1	2215	254.8	8.54
Q3V0M2	Lrrc36	1	1	1	1	755	83.7	7.31
Q61084	Map3k3	1	1	1	1	626	70.7	8.87
Q8CIH5	Plcg2	1	1	1	1	1265	147.5	6.77
Q61333	Tnfaip2	1	1	1	2	691	78.1	6.07
Q9JJI6	Pigo	1	1	1	1	1093	119.1	8.82
Q8R5K4	Nol6	1	1	1	1	1152	129.1	6.79
Q91VH6	Memo1	2	1	1	1	297	33.7	7.14
B1AZA5	Tmem245	1	1	1	1	876	97.3	8.75
Q7TS63	Zfat	1	1	1	1	1237	137.8	7.2
Q8VCM3	Zfyve21	3	1	1	1	234	26	8.34
Q91Z22	Tmem123	5	1	1	1	195	20.2	9.51
Q9QY01	Ulk2	1	1	1	1	1037	112.8	8.53
P49446	Ptpre	1	1	1	1	699	80.6	7.12
Q9D8C2	Tspan13	3	1	1	1	204	22.2	8.37
Q3TZA2	Cdkl4	2	1	1	1	342	39.4	9.16
P70445	Eif4ebp2	11	1	1	1	120	12.9	6.52
P58059	Mrps21	8	1	1	1	87	10.6	10.32
Q80YQ8	Rmnd5a	2	1	1	1	391	44	6.06
Q9DBB8	Dhdh	2	1	1	1	333	36.3	6.44
Q810C1	Slitrk1	1	1	1	1	696	77.8	6.49
Q8K2A1	Gulp1	2	1	1	1	304	34.4	7.9
Q66T02	Plekhg5	1	1	1	1	1073	118.9	6.87
Q80VM8	Pnma8a	4	1	1	1	430	48	9.45
Q9D486	Cmip	1	1	1	1	773	86.2	6.81
Q8K4K6	Pank1	1	1	1	1	548	60.1	8.03
Q9R1Z8	Sorbs3	1	1	1	2	733	82.3	9.2
Q99PP7	Trim33	1	1	1	1	1142	123.8	6.74

Q8K2C7	Os9	1	1	1	1	672	76.1	4.84
Q3TBW2	Mrpl10	3	1	1	1	262	29.4	9.69
Q810C0	Slitrk2	1	1	1	1	846	95.4	7.65
Q8BR90		2	1	1	1	294	33.5	5.19
Q5DU56	Nlrc3	1	1	1	1	1064	115.9	7.94
Q9JLV1	Bag3	1	1	1	1	577	61.8	7.27
Q04207	Rela	2	1	1	1	549	60.2	5.76
O70422	Gtf2h4	3	1	1	1	463	52.2	9.04
Q8K004	Spata2	2	1	1	1	515	57.8	8.63
A7XV04	Skint7	2	1	1	1	395	45.2	7.59
Q9JI39	Abcb10	2	1	1	1	715	77.1	9.6
Q9ET54	Palld	1	1	1	1	1408	152	6.25
A2A6Q5	Cdc27	1	1	1	1	825	91.8	7.02
Q8BIP0	Dars2	1	1	1	1	653	74.1	6.98
Q6ZQF0	Topbp1	1	1	1	1	1515	168.8	6.67
Q9D9Q6	Calr3	2	1	1	1	380	44.2	6.43
Q9WU60	Atrn	0	1	1	1	1428	158	7.27
P52430	Pon1	3	1	1	1	355	39.5	5.22
Q8BTI9	Pik3cb	1	1	1	1	1064	121.6	7.09
Q9Z2R6	Unc119	3	1	1	1	240	27	6.15
Q8CIR4	Trpm6	1	1	1	1	2028	232.7	8.27
Q8BGC9	Creg2	3	1	1	1	288	31.7	7.77
Q9JJ26	Mefv	2	1	1	1	767	86.4	7.2
P0C7Q1	Ccdc153	5	1	1	1	202	23.4	9.44
Q6PFX9	Tnks	1	1	1	1	1320	140.9	7.05
Q62441	Tle4	1	1	1	1	773	83.7	7.5
Q8C2B3	Hdac7	1	1	1	1	938	101.2	7.47
Q3UZZ6	Sult1d1	9	1	1	1	295	35.1	5.76
Q9D5Y1	Ccdc39	1	1	1	1	937	110	6.6
Q14AT5	Ano7	2	1	1	1	859	97.1	6.13
Q91YK0	Lrrc49	2	1	1	1	686	78.8	8.13
Q99LB2	Dhrs4	3	1	1	1	279	29.9	9.38
Q8K327	Champ1	2	1	1	1	802	87.5	7.85
Q9DB00	Gon4l	0	1	1	1	2260	248.6	4.87
Q03146	Ddr1	1	1	1	1	911	101.1	6.58
P51954	Nek1	1	1	1	1	1203	136.6	5.45
O54943	Per2	2	1	1	1	1257	135.8	6.28
Q99KL7	Rab28	3	1	1	1	221	24.7	5.67
Q9CPX7	Mrps16	5	1	1	1	135	15.2	9.67
Q05860	Fmn1	1	1	1	1	1466	163.5	8.59
Q9CWV1	Mcm8	1	1	1	1	833	92.3	7.62
Q9CQ82	Itgb3bp	4	1	1	1	176	20	9.82
P08121	Col3a1	1	1	1	2	1464	138.9	6.52
O08665	Sema3a	1	1	1	1	772	88.8	7.42

Q8R5L3	Vps39	2	1	1	1	886	101.6	6.99
O35855	Bcat2	2	1	1	1	393	44.1	8.29
Q9WUU9	Mcm3ap	1	1	1	1	1971	217.2	6.61
Q6PDM1	Msl1	1	1	1	1	616	67.3	9
Q91VX2	Ubap2	1	1	1	1	1132	117.9	7.72
A8E0Y8	Cd101	1	1	1	1	1033	114.1	8.02
Q8BLY3	Lrfn3	1	1	1	1	626	66	7.08
Q9CXB8	Alpk1	1	1	1	1	1231	136	6.38
Q5SS80	Dhrs13	2	1	1	1	376	40.7	7.93
Q8CG64	Fkrp	2	1	1	1	494	54.8	7.17
Q5D525	Syce1l	3	1	1	1	247	28.4	4.94
Q99MY0	Spz1	2	1	1	1	378	43.1	8.81
Q9D2Y4	Mlkl	1	1	1	1	472	54.3	8.25
Q60665	Skil	1	1	1	1	675	76.3	6.58
Q9D5H4	Ftmt	8	1	1	1	237	27.1	7.56
E9PVX6	Mki67	0	1	1	1	3177	350.7	9.72
Q8R4X1	Acer1	3	1	1	1	273	32.1	8.22
Q9WUS4	Gja10	4	1	1	1	505	57.1	8.27
Q8CGC6	Rbm28	1	1	1	2	750	84.2	9.54
P21183	Il5ra	2	1	1	1	415	47	7.84
E9PVD1	Ccdc62	1	1	1	1	701	79.3	6.23
Q9CVW4	Spata45	8	1	1	1	97	11.4	9.51
Q8BXX9	Ccdc169	3	1	1	1	214	24.7	9.33
Q920F6	Smc1b	1	1	1	1	1248	144.4	7.12
A2AJX4	Malrd1	0	1	1	1	2123	236	5.3
Q9Z2R9	Eif2ak1	1	1	1	1	619	69.7	6.01
Q3UEI1	Pde4c	1	1	1	1	686	76	5.02
Q80XE1	Ric8b	2	1	1	1	520	58.6	5.72
O70370	Ctss	4	1	1	1	340	38.4	6.96
Q9D4H9	Phf14	1	1	1	1	881	99	5.34
Q8R1B0	Stac2	3	1	1	1	408	44.8	7.75
O70340	Nptx2	2	1	1	1	429	47.1	5.81
P49891	Sult1e1	3	1	1	1	295	35.6	7.01
P43883	Plin2	2	1	1	1	425	46.6	6.87
Q8K0D2	Habp2	2	1	1	2	558	62.3	6.37
Q9D0I6	Wdsub1	2	1	1	1	474	51.7	6.34
Q8K007	Sulf1	1	1	1	1	870	100.9	9.01
Q64104	Nr2e1	2	1	1	1	385	42.6	8.94
Q3U962	Col5a2	0	1	1	1	1497	144.9	6.7
P81117	Nucb2	3	1	1	1	420	50.3	5.15
Q8BL06	Usp54	0	1	1	1	1588	176.6	7.62
Q7TSH4	Ccp110	1	1	1	1	1004	111.1	8.85
Q9D3A8	Nos1ap	2	1	1	1	503	55.8	6.09
O54879	Hmgb3	7	1	1	1	200	23	8.37

Q9DAW6	Prpf4	1	1	1	1	521	58.3	7.28
Q8BGX0	Trim23	2	1	1	1	574	63.9	6.46
Q9R269	Ppl	1	1	1	1	1755	203.9	5.54
Q8CIM1	Lrrc45	1	1	1	1	670	76.3	6.33
O54931	Akap2	1	1	1	1	893	98.5	5.21
Q6PB90	Pcdhb14	1	1	1	1	796	87	4.89
P11416	Rara	2	1	1	1	462	50.7	7.9
Q99NG0	Rad54l2	0	1	1	1	1466	162.4	6.13
P15307	Rel	2	1	1	1	587	64.9	6.54
Q8R2M2	Dnttip2	1	1	1	1	758	84.2	6.44
Q76HP3	Tmem132d	1	1	1	1	1097	121.3	5.2
Q3UV71	Tmtc1	1	1	1	2	942	105.7	9
Q8BZH1	Tgm4	3	1	1	1	670	75.5	8.47
O88428	Papss2	1	1	1	1	621	70.3	7.58
Q64676	Ugt8	1	1	1	1	541	61.2	9.42
Q4QRL3	Ccdc88b	0	1	1	1	1481	166.5	5.33
O08850	Rgs5	4	1	1	1	181	21.1	8.68
Q4VK74	Ifnl2	4	1	1	1	193	21.7	9.25
Q3UPF5	Zc3hav1	1	1	1	1	946	106.6	8.27
Q80VW7	Akna	1	1	1	1	1404	153	6.19

Supplementary Table 2 | The expression levels of all the proteins quantified in young and aged mice.

Accession	Aged-ICH-1	Aged-ICH-2	Aged-ICH-3	Aged-sham-1	Aged-sham-2	Aged-sham-3	Young-ICH-1	Young-ICH-2	Young-ICH-3	Young-sham-1	Young-sham-2	Young-sham-3
P16546	0.868594476	0.959150244	0.969859319	1.020486258	0.993936346	1.016340133	1.01533628	1.000126699	1.009531146	0.981818545	1.072202348	1.082340336
Q9QXS1	0.996785448	0.973100839	0.95438379	0.987388422	0.958501793	1.02506554	1.033248126	0.962688314	1.011627513	1.031044046	1.043119797	1.051753074
Q62261	0.877832157	0.98647702	0.986637887	1.020431763	1.030078887	1.028212336	1.004528378	0.996346372	1.004524519	1.001008094	1.033388916	1.038354996
Q9JHU4	0.999396598	0.993657646	0.982587633	0.997195036	0.98350036	1.008056563	1.022849472	0.981111176	1.015841027	0.99614647	1.015551631	1.021397695
Q6PIC6	1.047464665	0.962173496	1.012529162	1.054140868	0.980630711	1.001152667	0.923061269	0.973208245	1.011749568	0.922691528	1.039801688	1.0751804
Q9QYR6	0.940152572	0.946739554	0.955005571	1.018671834	1.013378284	1.013805283	1.016672455	0.980642242	0.992199568	1.114987951	1.032213955	1.041100416
P20357	0.876114636	0.921380036	0.985551188	0.990942467	0.974584919	0.988069986	1.049060716	1.031719506	0.99134975	1.12258848	1.046188406	1.066058094
O88737	0.924732889	0.966852213	0.966166405	1.002287254	0.984314381	0.99837364	1.041076844	0.978652289	1.008511269	1.100176761	1.032399036	1.045276195

Q68FD5	0.897229579	0.930200316	0.971083767	1.049101194	0.954624731	0.975366293	0.979628674	0.984806767	1.030377339	0.936220019	1.12173433	1.137410577
Q8C8R3	0.948636692	0.962116255	1.00213751	1.011872196	1.008805744	1.003530669	0.996084236	0.988650144	1.012387864	1.048446971	1.013290014	1.040485121
Q61879	0.96701623	0.96198457	0.958930355	1.005365355	0.982248596	1.017928295	1.018776747	0.984410291	0.996821525	1.005675682	1.054697226	1.062864406
Q8VDN2	0.92908558	0.904895176	0.908320969	0.98866071	0.993003195	1.007279697	1.066081887	0.994687138	1.027325847	1.085786495	1.056778951	1.07477457
Q6PIE5	1.064371979	0.949354728	0.99066873	1.012305672	0.998433597	1.028569378	0.965403266	0.953317992	1.002171956	0.972213643	1.033996276	1.054249711
P14873	0.973817253	0.967574445	1.002916727	1.033087314	1.033399735	0.993464688	0.973395674	0.965709474	0.983846772	1.072019258	1.015175982	1.0461187
Q9QXZ0	0.961617379	0.974854253	0.981012973	1.014759714	0.999169592	1.01384981	1.001423437	0.982159075	0.9895141	0.996485322	1.052415865	1.047813085
P07724	1.477585956	1.494875798	1.632586391	0.556956641	0.443171951	0.364654996	1.366901634	1.431604361	1.564215817	0.464387	0.460335252	0.399438557
Q9R0K7	0.930272935	0.919786049	0.916669606	0.972422635	0.95194418	0.927022949	1.065786546	1.004234215	1.073658385	1.039894509	1.096879489	1.098646364
Q8VDD5	1.117397877	1.008640892	1.007982207	0.940389127	0.933671756	0.981178132	1.055758725	1.007792798	1.044419521	0.96771717	0.973405158	0.968515063
P60710	0.963365827	0.93685774	0.959203125	1.001371002	0.932740062	1.062780416	0.993610128	1.063512965	1.003941503	1.008217077	1.009793139	1.084623436
P52480	1.01165305	1.003964815	1.00892199	1.081033959	1.038365446	1.065610605	0.933612024	0.957718719	0.949357586	0.934784622	1.003807694	1.038395107
G5E829	0.913256906	0.940819878	0.92330246	1.024550173	0.963088394	0.955148954	1.032440094	0.970434303	1.025739321	1.032263845	1.089181685	1.143839364
Q99104	1.020383043	0.979401534	0.981530601	1.012230834	1.015433466	1.071064199	0.936451216	0.967280621	0.922940823	1.032953348	1.046197183	1.074340742
Q9QYX7	0.958650298	0.979512892	0.963356053	1.001404115	0.963536855	0.994813243	1.034901206	0.98030411	1.015286852	1.111355536	1.015887897	1.040376502
Q7TPR4	0.899831157	0.920456808	0.923163449	0.984211376	0.960186312	0.956360084	1.064616257	1.003775129	1.050158449	1.030816995	1.08461321	1.120010653
P56480	0.985251877	0.945064908	1.002249183	1.01983052	1.060886604	1.069370975	1.010285639	0.996451464	0.982397791	0.965890338	0.985300864	0.995222988
Q03265	1.012739481	0.934668299	0.988969885	1.018685702	0.988705888	1.079910103	1.004897611	0.973806323	1.000094965	0.976612719	0.987828689	1.058285693
Q8CAQ8	1.000478796	0.951916955	0.981746863	1.021197171	0.99127281	1.067735872	1.001078439	0.985035094	0.997457603	0.978114762	1.003201317	1.042260908
P46460	0.943985999	0.964516724	0.990119937	1.049374638	1.038478638	1.030398624	0.986314508	1.004548103	0.955701763	1.031051746	1.017955261	1.029909313
O08553	0.973122104	1.01019806	1.060703626	1.108625349	1.161862034	1.129982837	0.884995145	0.960895649	0.87216438	0.907853455	0.991162084	0.972314142
O08599	0.952830272	0.978075257	1.00469426	1.031445332	1.030261833	1.036366332	1.007125012	0.914187001	0.937403948	1.069930351	1.058359469	1.036347189

Q7TMM9	0.969365819	0.926469858	1.013165592	1.055425467	1.006449709	1.024044965	0.986213448	1.004399148	0.993279456	0.938536342	1.019078718	1.062637751
P17182	0.974530301	0.993184223	0.998862057	1.068856494	0.981527311	1.099110182	0.954941496	0.978238805	0.970873523	0.953583948	0.987058092	1.064206686
P63017	0.964256662	0.95409669	0.966948181	0.963672163	0.96265713	1.012782702	1.032687116	1.014828392	1.027745485	0.973387912	1.03498948	1.085619057
O55143	0.955965915	0.953501142	0.941324023	1.038169537	0.998494583	0.953188169	0.973440123	0.947147755	1.008903553	0.952471218	1.11902502	1.151818729
Q9CWF2	0.683386078	0.628808307	0.761338181	0.689786847	0.619364683	0.754069415	1.352819431	1.336377238	1.448852585	1.103058666	1.164000503	1.294093452
Q99KI0	1.002428594	0.971517277	0.98864137	1.025995197	1.010734951	1.067382569	0.983395494	0.963884864	0.973849216	0.971167908	1.033274083	1.028599711
P99024	1.096534129	1.042548779	1.057508193	1.044174779	1.038617207	1.010126352	0.943854173	0.96816483	0.916866144	0.970126242	0.983759267	0.976796748
P07901	0.973827283	0.913145286	0.959023717	0.997433376	0.928145832	1.01020738	1.03043714	1.016191361	1.033258228	1.0516665	1.027677448	1.0879424
P08553	1.261726301	1.073420412	0.967742518	1.032352561	1.133015674	1.090052594	0.890933184	0.936106064	1.006831287	0.88957522	0.899986947	0.875546997
P39053	0.937481116	0.975816841	0.969153094	1.01643598	1.002970136	1.007303222	1.037923166	0.972119324	1.002387183	1.058248041	1.017667232	1.042437207
Q7TSJ2	0.986926838	1.017068761	1.014250331	1.071464901	0.998392983	1.020336128	0.982280511	0.938045779	0.948175657	1.159862155	0.954909524	1.026054534
P68369	0.923859067	0.952033444	1.029727981	1.039565756	1.236583033	1.058546088	0.880745205	1.005968809	0.794348781	1.122311799	1.096447488	0.961790601
P68372	0.979353732	0.970323285	1.023545803	1.066812718	1.046352554	1.019169726	0.934892163	0.962091195	1.001115334	0.933807463	1.015157347	1.056309532
Q71LX4	1.03849417	0.994620163	1.005458715	0.987282608	0.96220608	0.990457646	1.018630108	1.017505174	1.030877715	0.969936107	0.990267163	1.000774464
P68368	0.921563324	0.906380857	0.957259924	1.177091659	1.06906037	1.03360102	0.845524654	1.025693778	0.900640688	0.816128858	1.190615628	1.113650679
P46660	1.196643791	1.097353154	1.020215741	1.055782108	1.10261885	1.150381225	0.85013454	0.915428506	0.968941773	0.869780736	0.909430606	0.913542904
P17710	0.995526103	0.965053255	1.007009699	1.019358455	1.004887622	1.034832961	0.999068027	0.971620525	0.988831917	0.975400123	1.018936262	1.036270234
P11499	0.921688381	0.897097837	0.948790484	0.97486609	0.958639428	0.982703548	1.032983038	1.028124943	1.068940136	1.028517087	1.055412168	1.103066479
Q9D6F9	1.055976367	1.00721508	1.000743588	1.039682325	1.138415425	1.104617559	0.971612132	0.96511181	0.94861243	1.020839352	0.916091514	0.910123783
P05063	1.011088521	0.987167247	0.985998462	1.024999726	0.989274642	1.070376623	0.994743946	0.965470817	0.984296158	0.932936489	1.00683487	1.055576759
P63038	0.960424529	1.034543602	0.983993723	1.00421054	1.015630281	1.0209704	1.010593593	1.011740987	0.987116863	0.941047008	1.018735251	1.005764405
P05064	0.945128728	0.968452188	0.978162378	1.036755584	1.016711148	1.032781444	0.986552261	0.98329994	0.967423145	0.997858021	1.047253266	1.062972184

P17426	0.957698109	0.947425485	0.966590757	1.013239343	1.005796168	0.986574192	0.984968343	0.985772248	1.00558535	1.007138581	1.066654546	1.089581947
Q9ERD7	0.994809825	0.991005021	1.029627526	1.062977824	1.021784594	1.02290191	0.934009856	0.985501504	0.991609789	0.928389963	0.9955584	1.052134098
Q01853	0.979399794	0.947890284	0.987469919	1.011223441	0.990349131	1.014200851	1.004961085	1.013488858	1.006169208	1.01695301	1.005777851	1.049764483
P60879	0.973362283	1.03554159	0.99582416	1.0183529	1.032779426	0.981875465	1.017547886	1.015071219	1.005020165	0.803592998	1.045698545	1.006367397
P19096	1.011497004	0.992644985	1.000728829	0.991150343	0.968848487	0.984929312	1.03138036	1.004009777	1.039678036	0.967138327	0.992415917	1.016929758
Q9Z1B3	0.909411788	0.965013667	0.94555838	1.042124378	1.035654958	0.996506562	0.963573764	0.96534605	0.957539527	1.075354498	1.09960864	1.094580283
P08551	1.280059353	1.072794212	0.955710844	1.026414816	1.174213864	1.143501701	0.848882909	0.920004108	1.007316812	0.921811764	0.878648668	0.856489447
P16330	1.219086369	1.074224768	1.017392957	1.145636214	1.18759455	1.144337715	0.797291746	0.877649906	0.959083247	0.796263155	0.909075533	0.916982863
Q61316	0.973680628	0.936874774	0.967583482	1.015529738	0.933919428	1.04273203	1.041912076	1.004543579	1.07382921	0.959559038	0.986903232	1.056361232
P57780	0.951544956	0.921194216	0.91446363	0.924374457	0.969623266	0.984691121	1.080447124	1.009534546	1.027697862	1.109431173	1.076295372	1.065723793
P05214	1.080542638	1.068803369	0.986318175	1.089831262	1.149749859	0.952937457	0.977761647	0.919862556	0.855517022	1.152263116	1.05823246	0.834437028
P63101	0.921087086	0.986161704	0.992431777	1.062307956	0.974941092	1.052540054	0.962946912	1.045779992	0.982840129	0.907779373	1.012843438	1.083732479
Q8CHC4	0.98177023	0.979773494	0.981291689	1.015698799	1.040619869	1.019520521	0.990611006	0.956615292	0.960721103	1.162202047	1.001255411	1.014094316
Q6Q477	0.928085249	1.013830738	1.008115462	0.982555284	0.968609849	0.966798616	1.028605895	0.98524152	0.999623269	1.05562411	1.0311703	1.059412728
Q02053	0.972746438	0.987502935	1.009445958	1.030859466	1.049311938	1.010665335	0.957429064	0.98751271	0.947326746	0.989399195	1.048648634	1.035205734
Q99P72	0.969001502	1.016586994	1.012741522	1.030781197	1.011184754	0.984115776	0.9758537	1.015021435	0.981267763	1.024576698	0.997202598	1.019038606
Q8BPN8	0.943718061	0.953450376	0.980029991	0.998752508	0.991097025	0.986636276	1.014919172	0.983389259	1.018794259	1.041188408	1.038541701	1.075574721
P12960	1.018836863	0.999666689	1.003802248	1.037030138	1.010283206	1.036173561	0.979479923	0.973841303	0.979501183	0.997734939	0.989410866	1.013827135
O08788	0.999140553	0.988017694	0.985651443	1.01845103	1.021156212	1.035993548	1.007439237	0.984080349	0.97915823	0.98777082	1.012013127	1.006184054
P48722	0.99978638	0.995554614	1.014980646	1.02760903	1	0.967180266	0.954856844	0.985705313	1.014083077	0.91226022	1.066274122	1.042227846
Q9WV92	1.004853972	1.007035395	0.990047114	1.056783683	1.077984998	1.170968942	0.914969792	0.957836601	0.969923331	0.977509797	0.955967831	0.969817988
P19246	1.277168727	1.089050904	1.011908751	1.095723918	1.166579197	1.109227868	0.823171567	0.905837171	0.953524813	0.906479556	0.880989168	0.871736142

Q04447	0.990657215	0.988023398	0.988492322	1.083471115	1.151025577	1.075595675	0.95984087	0.948486946	0.949125557	0.976322716	0.970571848	0.969340885
P38647	0.999678256	0.95183882	0.992030875	1.007230309	0.989600163	1.033288314	1.009266147	0.994808796	1.00879462	0.985723604	0.997705744	1.049413898
P17183	0.953043137	0.997776331	1.028647699	1.065824935	1.051576151	1.022622029	0.972015438	0.983907873	0.981325496	0.942476085	0.998938311	1.010024561
P62259	0.944984425	0.969721422	0.991723321	1.011275157	0.992566178	1.028287047	1.000926441	1.028577938	0.9748426	0.958778684	1.040044705	1.055843001
P13595	0.936998186	0.908197861	0.976280397	0.968608217	0.982499243	0.984018094	1.044363561	1.010788041	1.02931946	1.140830234	1.017309287	1.060231227
Q3UHJ0	0.925582143	0.960896796	0.987404967	1.000496155	1.042076882	1.018450283	1.026829714	0.962778383	0.987350202	1.102983089	1.013733744	1.031969876
P50516	0.97859393	0.994151454	1.003860013	1.058843609	0.995583986	1.023920543	0.954518284	0.989283297	0.964230027	1.009059211	1.033737203	1.029898434
Q91XV3	1.063621203	1.240375182	1.157221506	1.066510223	0.710750109	0.895140915	0.919460309	0.88482608	1.020590128	0.928512038	1.033954699	1.070620418
P16858	1.084370731	1.018540035	1.049868465	1.093311957	1.04920706	1.12736683	0.920265522	0.920362658	0.957337124	0.871063202	0.933158355	1.000666555
P17427	0.941776083	0.960677618	0.979870859	1.047481573	0.994004207	1.020204935	1.012793225	0.996141933	0.983075894	1.031369947	1.012037373	1.056730041
Q921I1	1.270236394	1.419721623	1.346731369	0.699797081	0.661914602	0.720524954	1.275591056	1.210401216	1.279734636	0.664291051	0.64664002	0.616156432
P62737	0.987342143	0.918831841	0.991233105	1.018668888	0.967068122	1.049916184	1.060510517	0.994813058	0.993096258	1.059675592	0.983591575	1.022101393
P15508	1.041492196	1.023528684	1.019158912	1.007511976	0.999082289	0.981779098	0.999114078	1.034093729	1.015762798	0.941672309	0.970978485	0.969192213
P09411	0.978001228	0.981043138	0.989846341	1.038810486	1.041617147	1.036579095	0.971471064	0.968409991	0.978364705	0.978664186	1.018290539	1.045177735
Q9JMH9	0.985560547	0.984963033	0.9601332	0.977826573	0.983655476	0.989274837	1.035109711	1.006789426	1.015899129	1.037757949	1.019637702	1.031950886
P11798	0.865881875	0.921777969	0.92207941	1.012826099	0.936840245	0.961037818	1.031896504	1.026265449	1.024766325	1.003381357	1.111533576	1.168013254
Q64727	1.158856916	1.024275742	1.038071255	0.992742837	0.990083762	1.039984041	0.973744377	0.981960921	1.018738961	0.952170779	0.920265683	0.945200235
P26443	1.090690186	0.990876442	1.051333141	1.027744652	1.010363903	1.041595572	0.961966931	0.938205486	1.005049796	0.928036831	0.97349071	0.999197652
Q9QWI6	0.932028494	0.965638518	0.973669068	0.953238244	0.938190617	0.944991166	1.10357519	1.035935063	1.076998677	1.025464749	1.009963889	1.032998131
P03995	1.538394482	1.219687613	1.227118358	0.974626099	1.3222852	1.224001716	0.802344869	0.850667446	0.811770348	0.704056263	0.727151972	0.674328156
D3YVF0	0.853770389	0.929099407	0.943561593	1.044865945	1.034531523	1.01443018	1.014155129	1.0547179	1.004440607	1.06734558	1.019016413	1.054525199
Q91VD9	1.025868169	0.969919691	0.995354309	1.040825172	1.012468637	1.073518643	0.982001936	0.966994114	0.989077208	0.963827962	0.982372939	1.026699904

Q61644	0.889429896	0.958285079	0.995487042	1.054513576	1.005716931	1.028970849	0.983285231	0.980345052	0.999845543	0.9873182	1.032930508	1.09447082
Q4KMM3	0.924504747	0.947292873	0.934551236	0.985916226	1.04115174	1.017354559	1.032767252	0.9694932	0.977124745	1.134785566	1.0391848	1.069306166
O88935	0.926817092	0.977762938	0.967646058	0.972145074	0.995322566	0.975172265	1.038836585	0.982876412	1.006907525	1.219350952	1.013673936	1.021790335
Q3UNH4	0.929487519	0.995966953	0.952087888	0.962842567	0.984050548	0.995840261	1.021242022	0.981289405	1.004578271	1.259607547	1.007238368	1.025439367
Q9JI91	0.901033916	0.979623904	0.934343253	1.061477449	1.053653775	1.069629459	0.9524846	0.943301961	1	1.062665865	1.01482083	1.088908369
Q8BL66	0.995793158	0.991639633	1.003003847	1.005612849	0.941466528	1.009527453	1.02679104	0.991920689	1.004124725	0.996553321	1.005374504	1.04549076
P58281	0.994277438	0.993000184	0.998655279	1.035507599	1.048416967	1.019174026	0.961818127	0.974984776	0.959569285	0.987842896	1.035839876	1.021199914
P42932	1.002044018	0.97237326	1.017901484	1.013820601	1.009444102	1.0104944	0.986701124	1.008847508	0.971864232	0.985748626	1.017030601	1.024790425
Q9DBG3	0.95440792	0.982672816	1.009473242	1.059387819	1.024597261	0.989713432	0.925319076	0.962831281	0.947131003	1.004746914	1.081529223	1.089833636
P26039	1.150953292	1.016304881	1.060338773	0.947883917	0.950749686	0.996072911	1.043767659	0.996085475	1.032835391	0.920219716	0.941011334	0.94214959
P01027	1.29530331	1.475990455	1.192785238	0.731381775	0.686139517	0.674279312	1.237956279	1.210656774	1.265508837	0.733015983	0.699621605	0.654318955
Q9ES97	0.934223401	0.988867486	0.977441395	0.992685906	1.031296894	0.996512891	1.025835976	0.98150273	0.966916042	1.084950889	1.043716973	1.025564894
P70336	0.952561772	0.982610942	0.96723495	1.016992882	1.023956365	1.00907119	1.023601559	0.977282133	0.974366161	1.014387033	1.042304904	1.041565736
Q80TJ1	1.010150911	1.000130317	0.991474912	1.016985063	1.009996291	1.002598747	0.978480387	0.967247577	0.998378439	0.99472455	1.022267909	1.035483667
P20029	1.017037694	0.943604505	0.97956897	0.947169027	0.934256507	0.997947693	1.078215833	1.036069893	1.064484278	0.968808464	0.98994901	1.031447335
Q60932	0.95099808	0.973500635	1.025011649	1.061265856	1.003288625	0.985705555	0.917716712	0.985585923	1.008272176	0.875001203	1.074241904	1.106393252
P61982	0.942298272	0.964902055	0.981401209	0.985591924	0.924936771	1.010119285	1.041326938	1.013315061	1.037024596	0.966072178	1.018302891	1.103002682
Q8CI94	1.049270192	1.025716466	1.063506876	1.070313223	1.09409239	1.077263498	0.931659951	0.917833475	0.946734179	0.916705269	0.976531163	0.958848617
Q8K1M6	0.961290226	0.964706691	0.974961322	0.982403964	0.98009187	1.009465111	1.027370454	1.016986091	1.018740359	1.00221757	1.020079305	1.052000062
Q8BYI9	0.95570323	0.960992617	0.950370935	1.035642833	1.016121681	1.063025847	0.973004842	0.937083	0.984689031	1.023603106	1.04799272	1.093397767
P28652	0.930274828	0.932319108	0.939916939	1.040956232	0.988560666	1.006133929	1.000235483	0.980100381	0.986155553	1.038393388	1.070861771	1.118650824
P16125	1.037014015	0.987873904	0.985960486	1.075829958	1.041646415	1.070625487	0.939052841	0.960267534	0.947815919	0.924638309	1.022688414	1.030019503

P08249	0.993747842	1.005125299	1.043128428	1.112840515	1.00894665	1.033620923	0.919573887	0.958221721	0.985967526	0.815813449	1.013793641	1.078060189
Q8BH59	0.990353612	0.976204546	1.023330279	1.053322479	1.043320283	1.038973446	0.97294593	0.963084887	0.976834657	0.967836726	1.007767019	1.009063483
Q6ZWR6	1.033013917	1.109661355	0.997671341	0.997862303	1.004490505	1.021891511	0.982488703	0.97265132	0.942395074	1.0044199	1.000210327	0.977590558
P62814	0.955097735	0.987477068	1.009401045	1.05393607	1.009802375	1.000434871	0.975137721	0.96199598	1.012129888	0.877644719	1.044618447	1.082571823
P68510	0.953667197	0.954137667	0.944662565	0.966121893	0.922854079	1.077319381	0.990927281	1.182398832	0.94919608	0.962361299	1.012070752	1.08144723
O55131	1.015675692	0.997541637	0.995163945	1.027784045	1.07080019	1.026290977	0.960682058	0.944628148	0.950585087	1.020276987	1.039392086	1.001023147
P48678	1.092719463	1.023308036	1.037907662	1.000246588	1.033199055	1.0351778	0.991541481	0.94941229	0.975200051	0.95069478	0.978483272	0.958646958
Q8BZ98	1.073462755	1.014281619	1.034605902	1.052742295	1.019395191	1.059929832	0.951103547	0.976084058	0.959511354	0.976582158	0.940153806	0.994598838
P63328	0.934283252	0.980455516	0.921186214	1.039464823	1.023783483	1.02979167	1.004422264	0.982812868	1.011874936	1.013785199	1.028322217	1.058525944
Q9Z2I9	1.035535251	0.995297205	1.014857834	1.024930501	1.007167898	1.037454448	1.01733709	0.995762926	0.983360349	0.930232676	0.978676567	0.985966533
Q9CQV8	0.967479199	0.963241801	0.973601837	0.971573831	1.060599275	1.013923587	1.047656287	0.99090176	1.008312406	0.964053385	1.020975922	1.016462433
P50396	1.037067741	1.00674913	1.01096668	1.027859816	1.063237317	1.037682896	0.937760414	0.980323569	0.948184281	1.018760563	0.993555708	0.997076182
O08532	0.940201956	0.946830837	0.977524458	0.985603879	0.982086757	0.997799237	1.047515069	1.033038612	1.03522246	0.993645399	1.010281907	1.049784431
P14824	1.097938754	0.9796631	1.015058892	0.975913921	1.057147239	1.042205281	0.98028123	1.012351266	0.967788726	0.971360052	0.977766443	0.956277767
O70318	1.036071928	1.011336736	1.019049194	1.058898445	1.076306363	1.084452603	0.946107435	0.951293693	0.936907249	1.022396841	0.959323081	0.971090977
Q9DBJ1	1.016026083	0.994025362	1.023016142	1.045437401	1.000454191	1.036431339	0.989139268	0.997150962	1.021697996	0.879159179	0.966033182	1.01559394
Q9Z1G4	0.958377208	0.943349109	0.981097876	1.018983581	0.944084113	1.012011443	1.015479934	1.027681902	1.014857092	1.033437438	1.025130666	1.048723682
P28738	1.010909166	1.037202088	1.023825961	0.975007868	0.989567873	0.953031543	1.018880864	1.015622452	1.019738368	0.971878466	1.010149884	0.97333924
Q810U3	1.160200104	1.073288353	1.041291855	1.075414324	1.068686634	1.100524758	0.880175918	0.877905417	0.942926573	0.911754329	0.960391664	0.962744177
Q91ZU6	1.003903342	0.99254877	0.973926328	0.997190011	0.984172541	1.014571097	1.021929234	0.991230698	1.002583769	1.017742369	1.007462443	1.023372261
Q60864	0.976746598	0.965869454	0.994180933	1.022056095	0.933415655	1.003927124	1.020864164	1.025654334	1.036732004	0.922322203	1.01561465	1.059668234
P18872	0.964595328	0.955725065	0.993132706	1.051128656	1.040536965	1.021330201	0.981091512	0.953823075	0.999975089	0.944639491	1.029660839	1.07045072

Q9CZ13	1.044577788	1.000262192	1.008922873	1.039906917	1.016202238	1.044858197	0.983313403	0.971786059	0.986056477	0.898708053	0.986945074	1.020462091
P28663	0.951777365	1.026487672	1.042121021	1.057191648	0.977827154	1.009077414	0.963118473	1.015228495	0.941734869	0.892518171	1.065286487	1.032406486
P20152	1.348424	1.020051188	1.052717815	0.950017165	1.130482057	1.179751295	0.928366299	0.954888795	0.909198084	0.827860857	0.913007869	0.820996854
P60469	0.935484577	0.959488818	0.976163289	1.008077238	0.990787726	0.991381782	1.015279426	1.001868251	0.985510224	1.033845665	1.061913806	1.062229853
Q8QZT1	1.02378496	0.954387541	0.987281078	0.996792544	0.993944174	1.006375329	1.017156873	0.994975763	1.011378142	0.955286654	1.018241144	1.04403027
Q9QXS6	0.928364537	0.940047841	0.94186417	0.968382233	0.985925177	0.97500763	1.024150987	1.017860472	1.016638259	1.170392116	1.043066564	1.060657881
Q7TQF7	0.958550677	0.993177027	0.99118425	1.03095452	1.073619636	1.040785907	1.004761213	0.99992658	0.981277764	1.081695445	0.94889808	0.96624989
Q05920	1.061580109	0.998545717	1.022752307	1.019653505	1.006235293	1.050851708	0.991474763	0.98586233	0.98741335	0.94560331	0.965892553	0.984870277
Q6ZQ38	1.010543134	1.00175504	1.002362945	1.004503704	0.980138981	0.999542642	1.001729223	0.983604184	1.008921594	0.978058036	1.008750761	1.034907068
O08638	1.309771059	0.950069237	0.968367012	0.967133754	0.884825463	1.048610862	0.983719116	0.977610478	0.998112998	1.009629608	0.987811853	0.982053845
Q8BGQ7	1.02530776	0.99164615	1.01822852	1.007327969	1.012188801	1.011838456	1.017035032	1.0000935	0.99720414	0.985558314	0.968902353	0.989965228
Q6URW6	1.090131266	1.046950431	1.008265085	1.045514991	1.034003312	1.131796546	0.941757472	0.932772467	0.970643092	0.926932232	0.954212512	0.954481098
P68254	1.014405531	1.031780347	1.039087952	1.01518636	1.022732915	0.997225508	0.958619337	0.992513376	1.006117696	0.872762331	1.008201159	1.017350088
P47857	1.030328645	0.97682589	0.980373687	1.020377623	1.057959138	1.046217037	0.985893478	0.953844759	1.00142842	0.961903626	1.003551639	1.005026197
Q61301	0.929537832	0.964916576	0.964113563	0.986443246	0.976024405	0.957864666	1.021511892	1.01597567	1.03370294	1.049355802	1.05455212	1.063490201
P63318	0.984267145	1.018810763	1.008516179	1.048993068	0.990504167	0.986955869	0.935681099	0.995154518	0.970906354	0.933196646	1.052823237	1.075823902
Q62108	0.9190658	0.936054542	0.958014857	0.979024032	0.99241716	0.971306501	1.045883278	0.998806543	0.985282322	1.095710134	1.095731939	1.056789782
P11983	0.987360316	0.990497233	1.00677111	1.015781077	0.995959331	0.997540488	0.982272548	0.997482301	1.010604493	0.978636854	1.01110584	1.039626229
P28660	0.972619612	0.997519017	0.963531927	1.011720294	0.974303332	0.993055297	1.001959984	1.000946865	1.014947513	1.032384352	1.019564737	1.049240877
P43006	0.878015011	0.959289757	0.988003505	1.053059836	0.994134977	0.896430523	0.937644273	0.97249918	1.12861653	0.836294299	1.144045836	1.12058349
Q922F4	1.002316487	0.998782562	1.009824646	1.063730532	1.008344403	1.017522659	0.955843992	1.007803127	0.990663971	0.905417546	1.009805173	1.025182027
P11881	0.902953453	0.95549496	0.964153819	1.029130194	1.019426944	0.979294398	1.010651272	0.974375602	0.991905225	1.020173753	1.071513356	1.098169831

Q91V92	1.027427739	0.999367525	1.01445432	1.066828623	1.032309975	1.030527001	0.954456203	0.953526304	0.965104096	0.948810843	1.003445034	1.031277533
Q9JJZ2	1.014471502	1.045996309	0.967441172	1.021081451	0.974406226	1.04132505	1.09563787	1.007940863	0.884391059	1.118123989	0.930479815	0.998505944
P15105	1.023198012	1.025357471	1.039854057	1.127545739	1.035294793	0.929337286	0.836898508	0.911363193	1.083968944	0.701563768	1.072747781	1.126476422
Q8C419	0.945524854	0.978989079	0.973117696	1.020751009	0.951782253	0.980817259	1.003479014	1.036455607	1.024201438	1.010592734	1.039524935	1.043976708
O35643	1.001059537	1.017900574	0.969489106	0.989821281	0.978541898	0.98717376	1.017574787	1.007727899	1.011786651	1.013431831	1.004387558	1.027128623
Q9CZW5	0.969157265	0.975663959	0.999787968	1.080183665	0.952026146	1.005272159	0.980294452	1.030737125	1.035169797	0.844097796	1.005753477	1.077919425
Q91V14	0.957176201	0.945771238	0.983768645	0.981239225	0.979687399	0.964295236	1.020639292	0.99804685	1.036604223	1.022144321	1.061604291	1.056308367
Q9R111	0.897837357	0.978055049	0.992028785	1.033362769	1.018326386	0.99878759	0.986710592	1.033125274	0.972755134	1.002593478	1.058435889	1.037177758
P40142	1.080473207	1.030651859	1.067535322	1.048965756	1.089497459	1.060586456	0.93493505	0.943349182	0.939575295	0.934869935	0.956022866	0.953160605
P97427	0.898684546	0.907856394	0.941906419	1.02352257	0.917283535	0.973757883	1.061282505	1.004305939	1.062902912	1.032660782	1.038817125	1.143664033
P0DP27	0.936086991	0.918356741	0.940930834	1.016410887	1.141970274	1.025766733	1.070129602	0.954017257	1.006995771	0.858012001	1.096691336	0.981095843
G5E8K5	0.974537804	1.002337647	0.989863631	1.01642203	1.026707134	1.007107207	0.979697246	0.969062217	0.969669478	1.052172675	1.026063972	1.037949381
Q3UHL1	0.849983322	0.967491326	0.983568772	1.080120804	1.009732903	1.000710566	0.994322811	0.953372641	1.004849465	1.049681408	0.97351746	1.18130258
P80315	1.106103673	1.048886146	1.000894599	1.043643787	1.015891487	1.033434206	0.930414622	0.986826972	0.989604813	0.895771788	0.981959763	0.979770855
P68404	0.912617448	0.974631751	0.939549427	1.083054889	1.007844599	0.957767008	0.946581684	0.995417907	1.033531436	0.869381457	1.105851478	1.129166914
P48962	0.964689581	0.896816848	0.970327208	0.969567392	1.001374203	0.950759	1.010571611	0.980925227	1.030394689	1.003112764	1.109321621	1.108966317
Q9DB77	1.00852481	0.976791687	1.009816225	1.019725841	1.010818214	1.075302684	1.010737381	0.998050202	0.988979863	0.945996158	0.972501159	0.994453178
Q91XM9	0.971464407	0.962998952	0.979616187	0.993630925	0.973158582	1.008622243	1.03219833	0.996094088	1.019190256	1.047998625	1.008063257	1.040483308
Q76MZ3	0.983047463	0.992754494	1.012341124	1.003946213	0.959061895	0.978393048	1.013097586	1.007525716	1.03499435	0.959878837	1.015376074	1.032299226
Q8K2B3	1.037742036	0.969114221	0.999894271	1.017702299	1.023144235	1.02182302	0.961792139	0.957808655	1.006484613	0.997547277	1.006289499	1.037724773
P17751	0.973110092	0.97131684	0.997805719	1.052233597	1.007755966	1.065906215	0.945348323	0.932511191	0.991556757	0.974051957	1.027806812	1.087079343
Q9EPN1	0.956761774	0.974050789	0.973927263	1.002783958	1.004082647	0.979645392	1.000636391	1.00185252	0.98510681	1.084469576	1.047909321	1.037960563

P05202	1.02021792	0.983811459	1.013135703	1.0534845	1.116604184	1.059050597	0.938174758	0.935663983	0.941294084	0.956832532	1.017013307	1.001468993
P10126	1.020983998	0.951042544	1.010143123	0.978977419	1.045574533	1.013699792	1.020212419	1.039684884	0.989136245	0.988153643	1.007715764	0.948785175
P97807	1.039502892	0.996401944	1.035816301	1.03889971	1.035489038	1.057912571	0.953508955	0.98930739	0.937400627	0.985748722	0.983548156	0.991309165
P51881	0.981309737	0.93523536	0.982211798	1.027346915	0.969520885	0.951935548	0.940063139	0.976901234	1.032007165	1.001042197	1.084231711	1.132212394
P58252	0.968213628	0.949883521	0.974054451	0.980195553	1.012802876	0.96578986	1.017396073	1.008103109	1.024503698	0.982480319	1.067812278	1.043383426
P80314	0.973757391	0.957714618	0.996275219	1.003211251	1.048324885	1.026473089	1.020665922	1.001055435	0.980074703	1.033422847	0.992060269	1.006993198
Q91ZX7	0.995583668	0.986455838	0.999944565	1.005357288	0.983918361	0.987743958	0.991595159	0.994859156	1.031316635	0.952300201	1.027554983	1.038881083
P21619	1.091222832	1.041226937	1.086371896	1.041383113	1.074994382	1.090848675	0.925814836	0.956735126	0.927484691	0.958623298	0.938671609	0.919224418
Q8K0U4	0.990294195	0.996081374	0.974407925	1.016904914	1.014760892	1.021622405	0.973507201	0.997625297	0.996237864	1.012889173	1.014869647	1.025259092
P10637	0.912451074	0.950519533	1.031378702	1.084610237	1.014129972	1.030677915	1.018632502	0.973481433	1.004622819	1.050776272	0.952923887	1.023982332
Q8BRT1	1.01601454	0.972516972	0.993689496	1.019480994	1.023591412	1.01345992	0.99173304	0.993597303	1.007651046	1.010415952	0.993867378	0.998848069
P23819	0.894947031	0.926358232	0.956174193	0.986048574	1.017807321	0.993450093	1.049734399	1.005443912	0.972302991	1.123768467	1.077323242	1.045836044
P61264	1.046252534	1.010741829	1.019580597	1.022521713	1.068582461	1.088711421	0.945799204	0.970074999	0.966235425	0.94026875	0.971096114	0.979642156
Q9QYC0	0.877356591	0.940255188	0.962915714	1.017401564	1.007567767	1.025126964	1.002980242	0.972708646	0.99901572	1.114250737	1.070106776	1.061253745
Q8R0Y6	1.024721657	0.969494806	1.010270091	0.978746943	1.005007923	1.01135342	1.024772582	0.990909125	1.020016023	0.972632329	1.003244088	0.996426437
E9Q3L2	1.005763609	0.997703044	0.972205861	0.979613797	1.005135933	0.981411993	0.992325868	0.963892605	1.021386229	1.052715211	1.013458217	1.061106295
P05201	1.031813945	0.994414794	0.995004437	1.043734631	1.014122599	1.096014293	0.977680862	0.936053799	0.966814311	0.932703974	0.990738537	1.044539126
Q64332	0.917134097	0.980149041	1.046186847	1.08357864	1.004794194	1.078323609	0.952557301	0.990498351	0.951944031	1.00458078	0.990721959	1.036229472
Q62420	0.964139851	1.024384794	0.985753017	1.022966361	1.052835199	1.007636828	0.971849866	0.983916346	0.942571384	0.938932053	1.058448019	1.050962495
Q9WUA3	1.023762999	0.975154067	0.986088081	1.007716335	1.058329501	1.061679989	1.018338402	0.960975995	0.98031732	0.940772654	1.008991748	0.988629973
P56399	0.967907613	0.979179811	1.025479421	1.01411799	0.996346664	1.018686037	1.013207142	1.020675378	0.978587834	0.962878433	1.003660651	1.023384247
P46096	0.917602014	0.951209884	0.965877426	0.993300304	1.003421011	1.031705262	1.050745996	0.999735382	0.986083646	1.076889707	1.031206727	1.032011388

Q9WV27	1.038312064	1.004391773	0.993497801	0.988494996	0.983092561	0.9720899	1.002308329	1.019133533	1.036699109	1.045942505	0.989677705	0.965311859
Q61768	1.046729992	1.0404924	1.042638678	0.992193299	0.975337303	1.013826368	1.000275818	1.006732511	0.99460546	0.940645458	0.967914772	0.985414264
Q8BWF0	1.088379164	1.025368331	1.065863366	1.102793165	1.038602852	1.067037505	0.896477553	0.956117186	0.942270262	0.862940651	0.971645427	0.997062754
Q8CGC7	1.027984084	0.969342197	0.968361009	0.986197965	1.017453875	1.034178517	1.005274048	0.982190505	1.015818974	1.001042806	0.996298523	1.026781682
Q8BFR5	1.054545664	1.04135248	1.043980616	0.99555069	0.948699053	1.016322178	0.969074818	0.979391164	1.028740516	0.96620179	0.949647381	1.028973273
Q61699	0.89985128	0.943357267	0.955200464	1.005419334	0.982543878	0.944334601	0.991991006	1.018778841	1.060726247	0.974383116	1.104401633	1.09421578
Q91VR2	1.042287213	0.956310889	0.99346822	1.000609609	0.989943002	1.058054485	1.042048135	0.989718376	1.052907825	0.935887183	0.932633446	1.011705655
Q9CWZ7	0.988677259	0.976420101	0.999004355	1.032937788	0.953687668	1.034597483	1.029911298	1.003787437	1.013116536	0.948430115	0.977166266	1.045301776
Q6PH08	0.918405645	0.969649074	1.018253306	0.984337873	0.981022713	0.959608745	1.022534764	1.024714606	0.994380513	1.060980423	1.065022332	1.019191154
Q61292	1.159666199	1.031594977	1.038651208	1.063440564	1.004146379	1.023179569	0.962126651	0.962504276	0.992163836	1.048224162	0.88967094	0.91818842
D3Z7P3	0.954492006	0.950726578	0.937355913	0.968079103	0.980648073	1.005593228	1.061254917	0.994302729	1.011328138	1.08681523	1.03072516	1.062346841
A2AGT5	1.018009259	1.006982286	1.006332096	1.020620415	0.981253762	1.005828754	0.98597151	0.979701368	0.974472096	1.011144671	1.013746017	1.032880504
Q99KJ8	0.987994618	1.004125913	0.999755985	1.011277163	1.031160612	1.067264373	1.015713843	0.968681445	0.998801558	0.942798402	0.989279256	0.989100111
Q61553	0.883559935	0.938993988	0.939147943	1.021433183	0.994885793	0.895856626	0.971245228	1.008857485	1.063720982	0.917392813	1.143934714	1.170381304
F6SEU4	0.918374565	0.968506318	0.964007352	1.006608173	0.961804868	0.988100684	1.02332879	0.98460157	1.016918429	0.995582875	1.066574999	1.105610915
P17156	1.017156309	1.020243818	1.066500712	1.050428955	1.084575043	1.074996236	0.966387827	0.989067558	0.944700651	0.910830322	0.953172709	0.936024125
Q6P9K8	0.958184685	0.989401521	1.022458638	1.055579384	0.959124571	0.938093436	0.948245737	1.006575227	1.011978259	0.853325556	1.094494464	1.108797041
Q9Z2Q6	0.963358052	1.007572606	1.01562528	1.072554016	0.961823068	0.950425242	0.928866629	0.958367175	1.080882969	0.832307923	1.040803102	1.134792751
Q8K596	0.921202957	0.986708505	0.973951497	1.043110406	1.010565562	1.000493591	1.022867254	0.98386107	0.960742167	1.077351844	1.028687399	1.043363804
P14733	0.990640595	0.922060219	0.952932986	0.931736529	0.939263303	0.985768635	1.085370706	1.038275843	1.08104438	1.033788001	1.009208871	1.035354284
P14094	0.985075628	0.950176931	1.01164311	1.03319441	1.017868073	0.982489747	0.954259191	1.013632329	1.025446192	0.846605004	1.054977935	1.077275092
P62874	0.96595497	0.988390423	1.034878828	1.097845869	1.056495283	0.948201465	0.932992354	0.977014388	0.961132773	0.88296694	1.080452651	1.053080409

Q64521	0.975471368	0.996448146	0.985372627	0.96761043	0.942831845	0.963050006	1.049758351	1.039382467	1.04117123	1.014698942	1.006773057	1.023779932
P08113	1.002724469	0.951737603	0.997573721	0.956083653	0.936205701	0.987975274	1.047525198	1.02204039	1.028705918	1.028509346	1.025797742	1.02845481
Q9Z0H8	0.963733299	0.983289315	1.000821127	1.029615489	1.030275849	0.987462599	0.986348225	0.979121956	0.977442165	1.02245997	1.043950487	1.026498645
Q60598	0.924304084	0.955263713	0.958067816	0.9660373	1.003883284	0.994422129	1.048671113	1.008233453	1.007748479	1.133943838	1.028799965	1.028662731
Q62188	0.867636107	0.817649815	0.87212164	0.868763506	0.839747587	0.852542117	1.175593291	1.144724536	1.18210778	1.116036289	1.091120485	1.141847135
Q61595	0.96294331	0.95864345	0.969895127	0.967049557	0.972617205	0.997192927	1.009837525	1.101033506	1.007957477	1.042544068	1.017272475	1.014439017
Q9DB05	0.980934339	1.014815775	0.995499732	1.027450211	0.983479531	1.012221085	0.9969109	0.982333623	0.998047755	0.892197812	1.024983921	1.070604867
P06151	0.84671438	0.914479188	0.947961191	1.002189222	0.97770054	0.949079194	0.999814383	1.028115542	0.983772377	1.028596616	1.157231203	1.157527055
Q8BKX1	0.942186099	0.978253011	0.998465208	1.003700564	0.977379859	1.005645707	1.048853778	0.997680348	0.99432124	1.01758394	1.016134367	1.036986437
P62631	0.989868208	0.974100646	0.996345663	0.999407674	0.968852723	0.988070028	1.008276354	1.019864987	1.008967621	0.963484827	1.020696646	1.062259574
P80313	1.02360481	0.975341305	1.024431954	1.040781081	0.988907075	1.037969893	0.97253636	0.98429075	0.983573966	0.948582414	0.988370256	1.0496411
Q9Z0E0	0.929326281	0.990846722	1.022958032	1.075347023	1.032720367	0.986151934	0.882339085	0.999852017	0.949124804	0.942024355	1.10447651	1.084804083
Q6PHZ2	1.015234162	1.022812917	1.102294417	1.053092168	0.969716742	1.065606497	0.96105523	1.046314084	0.94510948	0.935123549	0.915527707	0.988318043
Q9Z2H5	0.99286655	0.980415491	1.008159184	1.026564604	0.990479747	0.968204709	0.992896951	1.023677088	1.038233498	0.970426313	0.987671471	1.027487308
Q61753	1.068366338	1.00283337	1.04731517	1.073781795	1.064881985	1.090035815	0.947864587	0.953319759	1.005540816	0.863460928	0.926553257	0.961339319
O08539	1.02135804	1.036510901	1.020297448	1.089291288	1.09055911	1.113915254	0.937497476	0.926673874	0.936573683	0.899740248	0.978483467	0.973707201
P62880	0.993158069	1.008539135	1.029915585	1.119865295	1.010126196	0.933906385	0.881071594	0.973055041	1.000807725	0.812203205	1.075984288	1.117886382
P14152	0.963781839	0.979498587	1.013907191	1.080106035	1.00464515	0.999195345	0.951956861	0.989313244	1.019493475	0.791599422	1.045101614	1.098431118
P40124	0.976742718	1.051457132	1.00679149	0.980068126	0.973979315	0.938260894	1.010197144	1.013220228	1.024944106	1.027081625	1.015964552	0.999815497
Q8BWT1	1.105397915	0.980615812	1.051515347	1.063748943	1.003201699	1.109100242	0.954473178	0.970578056	0.939154026	0.96572658	0.949723454	0.958480515
E9Q401	0.953870738	0.974286621	0.956018315	1.012989663	0.972856473	0.977604891	1.020229061	0.980733902	1.015252337	1.041736425	1.050485291	1.071686566
Q8BTM8	1.06032092	0.991026157	1.079681705	0.985713899	1.071046238	1.006160999	0.964296395	0.996998223	0.936155751	0.992976667	1.010656189	0.938878467

Q9QYB8	0.9315971	0.984850679	0.994005622	1.00726922	0.995480301	0.993365195	1.048750194	1.025616523	1.002913049	1.06866587	0.990965294	0.994345536
Q11011	1.005505556	0.972399076	0.999577524	1.031529371	1.015401859	1.047256306	0.992165894	0.990085193	0.976482802	0.973206681	0.981876053	1.042772368
P27773	1.024272755	0.952912007	1.003768241	0.963842543	0.97027764	0.983563167	1.027258757	1.016957681	1.057710959	0.966462143	1.009041657	1.017025273
Q80SW1	1.037720064	0.99404266	0.985399477	0.993951003	1.028060883	0.980234954	0.889695742	0.985876872	1.027079531	1.05917503	1.051514498	1.021193912
P33173	0.999898311	1.022434738	0.989579573	1.000025307	0.996176085	0.991501911	1.025068509	0.990410096	1.003326641	0.99901186	0.997556718	1.007646577
Q8K310	0.982693308	0.971408517	1.020226077	1.003572894	1.013353722	0.990216132	1.022446742	0.992704344	1.009734349	0.969219084	1.009828511	1.017748838
P18760	0.916561934	0.929759626	1.006628013	1.060147362	1.058665401	0.946523754	0.953054112	1.000929902	1.058727391	0.932281465	1.048295491	1.067841039
Q9Z0P4	0.927906254	1.028250587	0.991705742	1.033767565	0.979487451	1.043265416	1.033553465	1.008751633	0.99357629	0.933167972	0.99961947	1.015744134
P84091	0.943535407	0.982558453	0.970574072	1.009067087	0.996181468	1.018712047	1.007455248	0.974929587	0.992963982	1.04731591	1.040564371	1.052118787
Q7TQD2	0.967075482	1.069841299	1.057237456	1.111351791	1.10099363	1.095169876	0.92547402	0.923476557	0.92702904	0.921618628	0.942662301	0.994516449
Q9WV34	0.920466459	0.977603738	0.965353737	1.006731322	0.995395784	0.961949767	1.019659396	1.00703601	1.038750796	0.972043296	1.067056578	1.052689511
P70296	0.962790866	0.950307885	1.014224708	1.103153687	1.075684486	1.068788793	0.91997045	0.968883536	0.963501092	0.947541111	1.002578813	1.050428737
Q01065	0.857134738	0.964880983	0.982473073	1.215535829	1.112602543	1.013980493	0.813450154	0.962222588	0.94352536	0.812030724	1.099796525	1.195270783
Q6R891	0.990577224	0.985692035	1.000472092	1.016151912	1.026736749	1.03743101	1.003044971	1.006609152	0.981922238	0.996556689	0.98757502	0.99617643
Q9D6R2	1.013800971	0.955658458	0.995023923	1.059373423	0.998009188	1.063562221	0.971009701	0.967216863	1.031472764	0.92795929	0.981168051	1.044234379
Q9D051	1.024634154	0.980395316	0.985780873	1.031300327	1.015867644	1.067837578	0.99586914	0.975642299	0.990782775	0.972808908	0.980581	1.008389879
O35526	0.888047668	0.961218754	0.93177941	0.975438918	0.989839738	1.007690861	1.102434607	0.997693744	1.01558607	1.050336369	1.038874442	1.055977247
P48453	0.912070053	0.956218176	0.98837275	1.060583557	0.985943769	0.9546723	0.982608309	1.000075324	1.028256667	0.857382056	1.082848876	1.13523526
Q8R0S2	0.968409937	0.960395367	0.971666565	1.001022904	1.00767967	0.992141334	1.04125028	0.992509183	0.980891301	1.074713074	1.037526661	1.017190397
Q91WC3	1.066911757	1.005838191	1.007524181	1.025710059	0.969227409	0.996381748	0.995557805	0.973219211	1.012145729	0.932750119	1.004194185	1.015830813
Q4ACU6	0.940809239	0.954578001	0.92751122	1.016203076	1.025540244	0.996747872	1.017626204	0.987082016	1.005217569	1.105953423	1.026531653	1.059644872
Q3TXS7	1.00853642	0.971809258	1.000190591	0.987861559	0.971640086	0.991176329	1.009891298	0.984352503	1.063366248	0.942890672	1.000457626	1.058791052

P61922	1.099675465	1.012800568	1.067128075	1.082508702	1.027299203	1.075227209	0.871182588	0.96117482	0.967511964	0.861000608	0.96745521	1.01991214
Q9JLM8	0.906666092	0.940049563	0.968456662	1.004120535	0.940004836	0.957059024	1.024968198	1.031595542	1.029002421	1.035560911	1.064125877	1.105112359
Q61548	0.97807086	1.022941643	1.017949752	1.023467772	1.006925503	1.026142426	0.999639503	0.999589158	0.958178798	1.043920095	1.008328885	0.959270633
P80316	1.036030621	0.977427087	1.039021546	1.009782732	1.033977195	1.014495654	0.993572488	0.990870734	0.985319092	0.982050582	0.976902398	0.988625772
Q923T9	1.028255563	0.987821137	0.972864567	0.967443814	1.034595657	0.996685556	1.015172233	0.900234811	1.018363364	0.996175896	1.05852492	1.044812828
Q3UM45	0.999783547	1.003573664	1.017176015	1.074645535	1.009377033	1.094020526	0.972157427	0.957588996	1.002425828	0.843956752	0.967676809	1.038787345
O88342	1.025948265	1.00019727	1.009895317	1.010171565	1.003645474	0.994471185	0.992025141	0.990110132	1.010645608	0.923782133	1.015332484	1.01742367
Q9CZC8	0.961947117	0.982972944	0.977754896	1.076012418	1.053677447	1.046351592	0.962262559	0.96481989	1.007376678	0.921102104	1.004543094	1.046190112
Q91YT0	1.036006421	0.973712812	1.015013509	1.04497617	1.007318202	1.049277363	0.986093007	0.991879307	0.981418524	0.961391617	0.96787035	1.013128746
P30275	1.028821572	1.003700242	1.033914856	1.038591852	1.020418227	0.969620416	0.973245685	0.987074853	1.011999934	0.899839343	0.992506904	1.031869184
Q5SQX6	0.978825283	1.057654948	1.010110712	0.978696866	0.989342746	0.974672027	0.991192398	0.9805872	0.952838519	1.060902455	1.045173048	1.025264469
Q8VEK3	1.008495533	0.955302405	0.986933852	1.025987097	1.013917097	1.050952068	1.005740599	0.971303502	1.020052465	0.917304541	0.990990666	1.050975434
Q8BMJ2	1.003229013	0.997866424	0.982962394	0.988871769	0.969184726	0.995959085	1.031598622	1.0036085	1.034484065	0.994663035	0.987217538	1.025797375
P09405	1.025873899	0.952454911	1.019615901	0.997083091	1.007093256	1.02853571	1.00584727	0.987710408	1.008585289	1.134791903	0.930711616	0.99492448
Q9CPY7	1.099611457	1.05261189	1.072671469	1.095172	1.132123897	1.060098236	0.868323952	0.926390654	0.945613978	0.852437676	0.947235773	0.971609517
Q80Z38	0.92429353	0.928658322	0.933806801	0.962665321	0.960115406	0.987687562	1.073555765	1.003946158	1.038025685	1.079099487	1.058472175	1.073280482
P02088	1.36895856	1.262727602	1.329234297	0.353694149	0.319912369	0.249523412	1.46293296	2.157690639	2.044727631	0.408079502	0.32221423	0.213573693
Q8C1B7	0.985306688	1.000600587	1.010105562	1.058498707	1.014024031	0.970527854	0.951516997	0.94632748	0.990462213	0.90201831	1.079110085	1.075743286
P06837	0.999971794	1.014291639	1.116876779	0.984152221	0.899922386	0.877784395	1.061909186	1.108972118	1.021753108	0.995270579	0.878420609	1.049897386
Q60597	1.018137811	1.027041167	1.017518651	1.043377939	0.988474673	0.979400284	0.962770986	0.972373205	0.994432726	0.926154481	1.029050254	1.041487572
Q9D6M3	0.885362947	0.873963493	0.92020149	0.97069444	0.91687905	0.981657734	1.073287998	1.014501905	1.082802677	1.06152978	1.065104111	1.158354454
P47738	1.104054546	0.983042607	1.045001427	1.048252062	1.025781108	1.089587017	0.949172122	0.934555499	0.959435128	0.917456242	0.973746877	0.998677458

P17742	0.977389272	0.934989983	0.976369732	1.021508139	1.012453103	1.065871822	0.992424675	0.980759894	0.982658487	1.044563928	1.007322598	1.053966811
Q9R1T4	0.976223335	0.981576742	0.981368902	1.04765766	1.008522546	0.960818761	0.951424332	0.955640561	1.026833907	0.948421596	1.067947043	1.092831936
Q80TL4	0.899041235	0.978689429	1.000548455	1.040276208	1.026801694	0.972793263	0.969766267	1.010399207	1.012579562	0.927217488	1.058107561	1.080821469
Q6PB66	1.035220246	0.990001801	0.98309212	0.991473743	1.005760072	1.010485168	0.997558915	0.992636919	0.990792995	0.987605657	1.014111083	1.02628153
P06745	0.982559695	1.00855464	1.021707423	1.064565915	1.066281398	1.106182583	0.946001805	0.970290788	0.928808713	0.955939288	0.97655308	1.009889849
Q9R1Q8	0.960170965	0.957139963	1.001041534	1.011116681	0.984853419	1.004712724	1.032634775	0.982956585	0.999125565	1.042983272	1.015919271	1.039991566
Q9JME5	0.941833997	0.952470446	0.973763501	0.987535921	0.984873548	1.008410318	1.048750382	1.030544648	1.036370067	1.019028222	1.007302795	1.019608659
Q00PI9	1.003140626	0.995035845	1.001323164	0.987982088	1.017624297	1.006696914	1.000043745	0.970868777	0.993274055	1.019398343	1.005965801	1.034284915
P47708	1.012027374	0.979596873	0.971818511	0.981441391	0.977370323	0.970530761	1.00573658	0.9905084	1.01728365	0.995631606	1.054506822	1.054944709
P62821	0.972175048	0.963256534	1.006348006	0.970776494	0.972908812	0.996388315	1.03823855	1.001792553	1.017980547	0.988003889	1.020314552	1.05351826
O35098	0.963164993	0.956846137	0.975279776	0.995166907	1.002610723	0.99436885	1.010404061	1.023758281	1.024684061	1.035912837	1.0113496	1.033779854
O54774	0.986252148	1.168169352	0.978281674	1.022987265	1.003883443	0.9865047	0.957226853	0.957617347	0.930850957	0.986933809	1.043710282	1.010883081
Q8VD37	0.960313984	0.965635474	1.007804201	0.997807384	0.994955888	1.018309206	1.020520065	1.009212269	1.015335055	1.023735192	0.995579196	1.01445745
O88569	0.9362591	0.947498032	0.998955435	1.003762974	1.092883549	0.989930692	0.980847332	0.996763864	0.956085527	1.236852027	1.011778621	0.971202109
P27546	1.000402547	1.023919905	0.995371309	1.026470749	1.055588671	1.045355135	0.964936365	0.968015793	0.963291615	1.05311481	0.973018168	1.000273433
Q8VD04	0.977954764	1.005365726	0.974271991	1.007630356	1.040566874	1.033654879	0.983927675	0.99585456	0.973577526	1.029204183	1.025752662	0.992047075
Q9R0P9	0.989386476	0.960913557	0.990321735	1.05101466	0.921781452	1.07624184	1.015599801	1.006750048	1.056796077	0.896973701	0.938107059	1.083049903
Q9CYT6	0.936377182	0.994532548	0.972069161	1.013193994	1.06340359	1.000178715	0.988799587	0.976855853	0.973110586	1.032028387	1.059414565	1.022177473
Q9Z2Y3	0.82531502	0.896809113	0.885742745	0.995803123	0.907472537	1.004851892	1.097204801	0.975045814	1.064153577	1.037838625	1.10878965	1.189769241
P09103	1.060120306	0.968218467	0.994262805	1.003375087	1.021130753	1.07502321	1.017676656	0.977631818	0.995148735	0.986423487	0.948279033	0.992412917
Q7TMB8	1.00066409	0.979336515	0.977460627	0.991557291	0.97060833	1.023053874	1.022439704	0.987688527	1.036593304	1.007118179	0.990989713	1.035506189
P26040	0.963462056	0.94438983	0.973339239	0.952836632	1.034536312	1.094617683	1.069743852	0.963878247	1.02897465	0.990155603	1.01963183	0.967621919

Q9CZU6	1.022154072	0.990467381	1.030064691	1.056690175	1.002999309	1.016315336	0.973591787	1.008839988	1.004248433	0.830279756	1.000889424	1.025311549
Q8K0T0	0.851212298	0.989696502	1.012964706	1.107616514	1.071298294	1.045482343	0.934885191	0.985812611	0.920350271	0.980308123	1.053657675	1.06867918
Q9D0F9	1.052191527	1.029676634	1.056707398	1.036137139	1.049698564	1.058511089	0.946107661	0.972530872	0.943611904	0.948293836	0.969305168	0.970571251
Q8BMF4	1.071073311	0.993030175	1.027874233	1.055031782	1.040560859	1.034630885	0.941645714	0.955112235	0.955047424	0.952972691	0.994894518	1.015054129
Q9WU78	1.030059573	0.985535734	1.013004206	1.022051621	0.982033794	1.038120994	1.015677734	0.978448018	1.01257368	0.97405049	0.968241564	1.002257397
P49025	1.049213007	1.020536838	0.988962503	0.98887105	0.982423591	0.991311772	0.999501567	0.981693495	0.971947698	1.002321781	1.025509868	1.030140229
P26638	0.969584598	0.945215262	0.957586597	1.006825094	0.954705117	0.998150574	1.012411588	1.002542418	1.033739015	1.025087664	1.038085023	1.076366459
P60202	1.548589629	1.276935955	1.118693035	1.302100574	1.406565768	1.280385388	0.635699223	0.696242778	0.747682647	0.640883682	0.757146593	0.719442529
Q61598	0.970371817	0.968466121	1.015531512	1.038628757	1.031386403	0.976801148	0.961685174	0.997503092	0.967932477	0.969841624	1.055912219	1.05669591
P08752	1.009081414	1.005169475	1.058519249	1.03909921	1.108976947	1.043656306	0.944996071	0.960750444	0.917202188	1.025287774	0.993710904	0.955215027
P47754	0.942603659	0.976329703	0.986429699	1.00330843	0.986793457	0.981577698	1.03399285	1.022134336	1.022212361	1.002449997	1.019923752	1.028276761
Q9CS84	1.15635799	1.08235386	0.877709847	0.852698977	0.851696279	0.932521475	1.170353907	1.155649806	0.901940256	1.316192934	0.928047217	0.934560161
Q99PL5	1.095832626	1.008894449	1.00946007	0.985495989	0.920822851	0.975876674	1.037019217	0.985426272	1.048806533	1.038577569	0.959197782	0.97675316
Q60930	1.090622992	0.956119908	1.026122307	1.043044994	1.04086756	1.053620362	0.942718002	0.954492184	0.966069276	0.943747763	0.98963722	1.027438786
P07759	1.06099758	1.078068771	1.098047859	0.730529073	0.670544729	0.641694894	1.476691013	1.332377646	1.447048555	0.831056124	0.728426795	0.706329327
Q8R081	0.95932199	0.936325138	0.993754304	1.018497526	1.002342002	1.019661178	1.014038619	0.990171909	1.001850087	1.017349496	1.016031896	1.056424815
Q6ZPE2	0.944953013	0.984375879	0.98803708	0.999158499	0.979924109	0.948947624	1.002931275	1.015505981	1.024652857	0.990843118	1.064071114	1.052428772
P39447	1.0589275	1.029348071	0.997904661	0.986878361	0.994005571	1.02195813	0.992325355	0.983616018	1.028426555	1.002621761	0.970392438	0.968050252
O55042	0.844780105	0.934317265	0.955645381	1.043490803	0.979943739	1.037428432	1.023060755	1.02875868	1.015931895	0.923405329	1.041145943	1.141505603
Q9JIS5	1.008749958	0.960546503	1.012615487	1.007249429	0.976202041	1.026953096	1.011505908	0.997956934	1.000712033	0.99562787	0.990272558	1.035426158
Q8K0E8	1.304083046	1.383034044	1.212783554	0.661992062	0.611208379	0.587931278	1.253244468	1.330555828	1.447492339	0.779743626	0.625052696	0.639676858
P99029	1.078315661	0.995535393	1.026969908	1.033373526	1.001229039	1.106550153	1.000327398	0.923496128	1.004817155	0.836097422	0.97100676	1.003226075

P61979	0.955898228	0.961509394	1.019150715	1.013433008	0.972282188	1.032368224	1.010950789	0.971924495	1.011203982	1.013557622	1.017314283	1.040297072
P62761	0.972807171	0.966955922	1.018184551	0.981518452	0.995558213	0.964791341	1.045042017	1.004212131	1.027821763	0.916770378	1.052414764	1.016717152
Q9WUB3	1.132503483	1.045070261	1.050817199	1.002046498	1.054074506	1.026878995	0.967095646	0.939174891	0.940698933	1.001469077	0.973090787	0.928330809
Q8CC35	0.872267227	0.962878139	0.930222325	1.01647352	0.948480023	0.987094188	1.069578152	0.985013321	1.051557573	1.052565413	1.042767203	1.095489975
P70398	0.949772045	0.959467613	0.985998944	1.016204494	0.978309945	0.97536788	0.982580808	0.981329132	1.028397413	0.986570045	1.075884205	1.079726515
P48036	1.195279006	1.052344879	1.057138551	0.992400766	1.193221038	1.080916851	0.919293552	0.938976743	0.924198541	0.907569871	0.919130831	0.873145421
P28665	1.214831135	1.314326582	1.205610541	0.712778662	0.666163855	0.634109934	1.395899319	1.256123015	1.408431627	0.700607805	0.647254147	0.637365194
P10630	0.95766932	0.958305679	0.976269385	0.982014487	1.002836857	0.986622237	1.045789872	1.014876907	1.027376422	1.04515199	1.028025589	0.996120171
P23818	0.923629608	0.99585898	0.982447924	0.998312201	0.977617432	0.966057394	1.032215899	1.033411205	0.989743818	1.029689331	1.047233166	1.037346115
Q8BVE3	0.962621159	0.972272113	1.015507337	1.035360874	1.073282214	1.03584744	1	0.968726954	0.970641911	0.975958721	0.994074714	1.019734563
P39054	1.013640834	1.013721107	1.01345512	1.017909972	1.083916094	1.036595421	0.968165791	0.971910443	0.965998036	0.946465246	0.992125274	0.997698859
P50518	1.028085222	1.061749911	1.064823414	0.971912827	0.930954531	0.978141997	1.086912808	1.043481057	1.036422006	0.893380977	0.937430006	0.930583014
Q60902	0.948012956	0.97008071	0.998229928	1.006909787	1.027256434	1.032359183	1.01202679	0.985665639	0.97575667	1.041454569	1.03440356	1.001845828
P31324	0.895673018	0.914607998	0.951552572	1.046742434	0.92759614	0.969927481	0.998365052	1.00409919	1.02982569	1.002881491	1.066189113	1.196663742
P67778	0.97890518	0.949625077	0.989165091	1.00740819	0.966658962	1.041055009	1.042230338	1.00681284	1.027503748	0.924954895	1.005906894	1.041693751
Q8BMS1	1.062555696	0.99117257	1.017575417	1.010921707	1.036904263	1.060286848	0.95009765	0.97836809	0.960252829	0.961078483	1.007078414	0.993917895
Q7TMY8	0.979386409	0.955350008	0.994029017	1.021156598	0.975315968	0.972963242	1.007980052	1.027924187	0.999861831	1.028771324	1.030914874	1.03138826
Q91V61	0.990589019	0.966019513	0.981188165	1.025385064	1.02400938	1.037554293	0.997429205	0.968628912	1.00237143	0.985643017	1.002988086	1.043922334
Q8CA95	0.969984381	1.000744219	0.969501421	1.180891135	1.078776016	1.117130067	0.894305984	0.906944239	0.978468965	0.853162682	0.98361202	1.078880692
P42669	1.011952281	0.959508324	1.02113648	0.974757204	1.032781402	1.018151275	1.033031423	0.999609541	1.000147609	1.050210758	0.959826265	0.98632585
P26043	1.07402548	1.002473404	1.012795718	1.021714938	1.031484816	1.072212024	0.990643819	0.97257831	1.000508817	0.961142011	0.933280895	0.963275574
O89053	0.945542178	0.949428756	0.964877151	1.017876632	0.94848508	0.999258116	1.034363434	0.981464267	1.05141678	0.972480741	1.031320672	1.097921795

O08749	1.02702794	0.966293859	0.984862997	0.9956204	1.029161328	1.071675316	0.990802984	0.966442963	1.002475583	1.020176582	0.983764407	1.007833396
Q9D0K2	1.005201531	0.9848037	0.987179549	0.982186231	0.973269146	0.994645275	1.053103385	1.014670635	1.053961888	0.986778226	0.967525247	1.005039368
Q922J3	0.93784903	0.946280432	0.966881666	0.998154074	0.995879394	1.018128889	1.026938626	0.982439042	1.00096673	1.03618352	1.048218475	1.06684247
Q61696	1.010800778	1.052960541	1.072308886	1.045516947	1.062996377	1.044869053	0.949415805	0.966312642	0.928322995	0.981395036	0.971841008	0.956495678
P12382	0.989320721	0.987956102	0.992356939	1.02548571	1.038178778	1.021660683	1.007816877	0.99102835	0.970916704	1.002394761	1.014753789	0.987891704
Q61361	1.005736292	1.002386401	1.012526909	1.076079231	1.04489359	1.032019267	0.93767518	0.960517365	0.974197316	0.950960269	0.998796661	1.032214101
Q9CZ44	0.978820813	0.987357033	1.00466159	1.00934514	1.0131148	1.046124718	1.016622172	0.983595048	0.991844872	1.010849953	0.985253237	1.003994295
Q810U4	0.9294886	0.958044572	0.954007272	0.990456017	0.99544554	0.992877861	1.033687064	1.007597111	1.009775549	1.023281593	1.060157539	1.056748856
Q61792	0.850822677	0.950064549	0.921478291	0.998592129	0.91792072	0.917230452	1.007450629	1.022881937	1.144925609	1.093344447	1.091417402	1.087350795
P97300	0.863280806	0.920461323	0.916115156	1.016318543	0.963359383	0.957049912	1.062124257	1.00105156	1.059778392	1.004380656	1.090160288	1.130361246
Q3THK7	0.986893982	0.991023122	1.031989586	1.032877087	0.963731626	0.970022836	0.983830484	0.975935127	1.046429787	0.927121714	1.000877377	1.078009959
P63330	0.914317481	0.97613793	1.020754897	1.095492833	1.01047298	0.913417252	0.912067647	0.98481945	1.069352208	0.783319678	1.104977867	1.12722477
Q7TQI3	0.978194739	0.966625922	1.023988057	1.0163755	0.964774158	1.015776543	1.006876686	1.002991739	1.005949422	0.922218631	1.015167794	1.065478202
P14211	0.974876925	0.920845656	0.954562816	0.964474681	0.957851942	0.96969033	1.024686237	0.997347249	1.017852114	1.246426862	1.022380919	1.060665364
O08709	1.012760421	1.01649913	1.042328827	1.069571654	1.020680161	1.102375705	0.996308295	0.951999283	1.007844987	0.848090102	0.931460921	0.985003172
Q8VDQ8	1.100884415	0.974190803	0.919737567	1.039770031	1.099092953	1.078930161	0.909290389	0.955432124	1.042459323	0.923215578	0.98829854	0.999403431
P02468	1.162823053	1.015164129	1.043687627	1.14649891	1.036334353	1.078591905	0.864178061	0.894419626	0.924434043	1.080320689	0.925021012	0.966259456
P70333	0.98647546	0.967886857	0.979400397	0.989206707	1.049189213	1.023471705	1.013527392	0.953143483	1.009223062	1.079355932	0.999787504	1.008830822
Q91WQ3	1.048491621	0.985404548	0.96139675	1.005784549	0.949222763	0.999154832	0.994912964	0.968590212	1.047143262	0.95562983	1.000058214	1.096388993
Q61838	1.408863929	1.537305365	1.194893659	0.639831445	0.638125935	0.636520798	1.318557835	1.287044042	1.32008774	0.70551757	0.595221968	0.561296477
P28740	1.017998352	0.976908125	0.987691032	0.999489166	0.960601093	0.998458366	1.017778733	0.990682278	1.025869518	0.998832744	0.995679515	1.052064316
Q8CGK3	1.004839187	0.985088866	0.993206769	1.008295469	1.031101868	1.019428232	1.0005012	1.046298116	0.997742216	0.951839259	0.98445723	0.984078374

P10852	1.021660775	0.958245197	0.985454536	0.971878571	0.944897762	0.992858312	1.039942846	0.992013403	1.053137234	1.003992933	0.990682393	1.061215955
Q8CGY8	0.94501513	0.959592018	0.971087449	1.001527454	1.000004527	1.010207697	1.036823147	1.009708557	1.012112397	1.010270517	1.022367321	1.035242215
P61205	0.921578228	0.97332913	1.004576224	1.006222773	1.089179428	1.046055677	1.013385201	0.978299961	0.91414219	1.107502212	1.030299591	0.986273491
Q922S4	0.917969634	0.97709716	1.007802764	1.036426968	0.948767728	0.990468894	0.995509677	1.038839315	1.026692026	0.915888354	1.046246652	1.063242926
P08228	1.005415423	0.93144623	1.041663639	1.049680361	1.02987245	1.067598447	0.98420091	0.993406223	1.003828389	1.001472066	0.938487135	0.994258231
P62715	1.04227437	0.964051	0.950071309	1.018702871	1.06753432	1.046431222	0.949276822	0.96869192	1.020645524	0.966257376	1.001919603	1.035012322
Q63810	0.940600281	0.947100519	1.001567515	1.04509621	1.184102071	1.041746547	0.942103036	0.924401989	0.901895696	1.110348722	1.088479293	0.956114295
P80318	0.977250966	0.95727773	1.0069872	1.00988828	1.041748278	1.009643168	0.980770058	0.997107678	1.002104182	0.937099301	1.027877662	1.047763693
P70704	0.959987359	1.003271799	1.047568809	1.055131961	0.99317639	1.058170359	0.970009731	1.006736231	0.956709836	0.971919805	0.99380724	1.003290687
O35737	0.948627581	0.978939639	1.038006486	1.05713119	0.996897327	0.936323458	0.936899099	1.044351234	1.038687914	0.950058366	1.019195521	1.046234636
Q9Z1G3	0.978105618	0.986082356	1.007182462	1.014131561	1.01167493	1.045625962	1.012560998	0.98482847	0.993551594	0.889275017	1.008229909	1.04768959
Q3U1J4	1.000706439	0.985354937	0.996358915	0.987726014	1.043649841	1.005365152	0.995800089	0.993485617	0.960240185	1.093026328	1.014552539	0.99135868
P33175	1.022752774	1.029653112	1.046419589	0.954751614	0.962870998	0.964606729	1.051891557	0.994118391	1.009830176	1.019179683	0.98169042	0.983210742
P56564	0.924530339	0.935066765	1.048053659	1.053006555	1.074307176	1.07282711	0.984048578	1.026261838	0.952299995	1.08048089	0.963961518	0.949625032
D3YZU1	0.93448336	0.947441708	0.937623709	0.925444516	0.944445798	0.956420557	1.082415625	1.021196991	1.048991232	1.156952487	1.054734006	1.037312783
Q2PFD7	0.911818201	0.989022844	0.974704611	0.980634533	1.005363038	0.983965679	1.063079939	0.972282454	1.002323247	1.129459454	1.038276869	1.003161638
O08917	1.014771582	1.054468574	1.036121934	1.02511299	1.020536836	1.042653701	0.980132202	0.977056687	0.939538662	1.011707118	0.976735921	0.970355407
Q5DTL9	1.020040098	0.974981971	0.958587698	0.938541417	1.011150016	0.982458612	1.011606012	0.956864134	1.010485753	1.186753558	1.042299819	1.000023634
Q3U0V1	1.010706207	0.963664101	0.99666236	1.034302712	0.918966408	1.054828056	1.043875659	0.971889421	1.060171785	0.914613259	0.949196454	1.072167611
O35551	0.960121102	0.966060487	0.971595235	1.065092317	0.977973093	1.025941027	0.999150413	0.957368618	1.007746934	1.004329812	1.025919093	1.066085042
Q61490	0.875876371	0.916488741	0.944855996	1.029554306	0.949838019	0.990507186	1.024562899	1.069620614	1.027889942	1.024721884	1.048006845	1.101768869
Q9JII6	1.080532469	1.05633044	1.050739306	1.046693278	1.104147107	1.0482835	0.930600733	0.970986508	0.918835768	0.920285472	0.972099615	0.933761799

P80317	0.990376003	0.970185418	1.012693247	1.013875523	1.011787581	1.006345934	0.981450017	0.986416104	0.982329553	0.984432402	1.02657858	1.052907174
Q68FL4	1.096002804	1.028787675	0.996748244	1.013942147	1.033360606	0.960929255	0.934188456	0.994906689	1.008670939	0.975028351	0.999370776	0.992148143
Q6NZL0	1.052697596	1.023331044	1.016608034	0.993848198	0.944514474	0.987059668	1.036510624	1.009313772	1.030197915	0.95721665	0.951625518	1.005645243
P63005	1.023180293	1.024190498	1.014514858	0.988227861	0.99348129	1.013026709	1.036885215	0.985279711	1.008563007	0.987607383	0.977127595	0.966969034
P47757	0.96204084	0.979377009	1.001212812	1.022263149	1.014257101	0.988824907	0.985746493	1.024950386	1.012389142	0.95216992	1.02711785	1.024262732
Q91V41	1.002822149	0.974583196	1.014871923	1.016776809	0.974800729	1.015403498	0.980648787	0.99708002	1.021164047	0.896512054	1.023084576	1.059780859
P46097	1.195619799	1.085962123	1.05090794	0.98843605	1.110286556	0.984232508	0.977705298	0.951677541	0.971464059	0.910072269	0.937120647	0.867003743
P35486	1.048046333	0.988867629	1.003455276	1.045481451	1.038661511	1.049325899	0.96197999	0.960711994	0.966818173	0.970201282	0.988348405	1.017637996
Q8BVI4	1.151282427	1.06260859	1.054228782	1.068507434	1.153004732	1.152374707	0.913242255	0.895447642	0.934359702	0.918101245	0.858527339	0.912916592
P48758	0.971984161	0.970718341	1.027561226	1.055374584	1.010539377	1.057419228	0.972330546	0.974717467	0.978826918	0.98889608	0.986024789	1.037622582
Q8R071	0.904728936	0.989031046	0.959532136	0.959997616	1.007152235	0.991212249	1.032114078	1.034087077	0.99286686	1.068993069	1.073232396	1.008584546
Q99PU5	1.064118681	0.998110228	1.002152634	1.00379932	0.993040276	1.050939034	0.979141701	1.005561628	1.00729372	0.959807071	0.972163258	0.986021024
Q8BJ42	0.905574377	0.948605343	0.951626323	1.014129739	1.054111438	1.02292338	0.996877526	0.987441763	1.046013467	1.008621509	1.030990527	1.046356934
Q60875	0.940299072	0.95959218	0.948156721	0.984154759	0.937312148	0.959605006	1.05472946	1.033889716	1.054090206	1.037650969	1.025151954	1.076687004
Q9JKY5	1.017623039	1.009742295	1.015186953	0.968207436	0.945443633	0.954577264	1.023173249	1.018671636	1.062706968	0.991212524	0.989936997	1.006871332
Q9QZQ1	0.965689747	1.010009583	0.999643092	1.01948758	1.065654975	1.008847281	0.966846239	0.987397173	0.969584291	1.022182201	1.030239921	0.99144063
P46471	0.965759421	0.979168972	0.983730418	0.997146398	0.987720369	1.020775344	1.026421255	0.997157557	0.998970931	0.993813006	1.016224843	1.047212089
Q9EQ20	1.125906016	1.047648547	1.07287849	1.109739137	1.058215027	1.078349098	0.893156278	0.912631718	0.930380348	0.862731356	0.958022487	0.97872997
Q9Z1S5	1.018016143	1.02280194	1.02923423	1.045087066	1.040945149	0.98673971	0.941566692	0.97529746	0.992929019	0.934103713	1.010525565	1.012794829
Q91ZA3	1.092919508	0.992063583	1.01078825	1.011696444	1.027251635	1.03468807	1.037254894	0.996413408	0.955183067	0.98081207	0.954802382	0.946225486
Q8R570	0.954631992	0.958780383	1.011612157	0.986668034	0.987626926	0.999366601	1.014011182	1.018405348	1.005128878	1.032345742	1.032493848	1.017793491
P16054	0.928659022	0.961289826	0.966377571	1.042751293	0.974097415	1.005596439	1.018741011	1.014755172	0.999939307	1.003417265	1.025536902	1.074592392

O70133	0.960185332	0.960533239	0.997696083	1.010339507	1.020897063	0.973133647	0.959460402	1.003980541	1.053662642	0.883054709	1.057557984	1.078650533
Q9CVB6	0.946668329	0.972893616	0.993502895	0.976030288	1.06188078	0.941818695	0.998851876	1.020714615	0.96513352	1.070661912	1.075791003	1.011678431
Q64487	0.978504121	0.967358143	0.996579488	1.014304585	1.013828688	0.9797272	1.00384456	0.99862337	1.001674438	0.983721662	1.040207947	1.030169249
Q05793	1.127142173	0.965930244	0.970538371	1.101151264	1.002622646	1.027995657	0.884364285	0.915754769	0.929705732	1.352915274	0.943014987	1.016509595
P97315	1.07746776	1.050115897	1.06986936	1.125717472	1.14013101	0.983768424	0.867049451	0.921632841	1.000239035	0.80825417	0.976566713	0.969385404
Q91V12	0.976910473	0.940783466	0.970673288	0.993487841	1.008716723	1.025628853	1.01546721	1.000668075	0.995838398	1.048283951	1.017978055	1.04587356
Q922D8	1.011440789	0.98477409	1.010100838	1.052862648	1.042852577	1.050878282	0.938888263	0.958578649	0.918956253	1.066453408	1.026808116	1.014615265
Q920I9	0.925621331	0.971444306	0.965982595	1.016939476	0.996735074	0.987192529	1.010653523	0.988010204	1.019228694	0.983493816	1.070902541	1.060398999
P70175	0.951833757	0.948283866	0.963116866	0.983443437	0.963674924	0.985964869	1.019804846	1.004931495	1.011155626	1.025771796	1.068036072	1.087257433
A2AN08	0.995266375	1.004452192	0.985232079	0.978289034	0.980123395	0.984043881	1.021554942	1.013002708	1.011837933	1.031661544	1.010663023	1.01153911
Q78PY7	1.003559448	1.022261732	1.005104337	0.980367864	0.968616338	0.99337968	1.015867759	1.006731499	0.974225544	0.987043427	1.023934635	1.032916817
Q811D0	0.989821213	0.979571519	0.959503705	0.983321121	1.006211502	1.026454545	1.016476462	0.97729363	1.01820834	1.038174567	1.015227051	1.026137625
Q04690	0.965622496	0.995227643	0.997405475	1.018248579	1.02140976	0.997914259	0.974363841	0.987293107	0.979562716	0.993926317	1.047254476	1.040842938
Q9D1A2	1.026362089	0.985499078	1.004277089	1.025077037	1.022808148	1.059420979	0.996076968	1.000616756	0.988420032	0.962154729	0.966698943	0.986622396
P42859	0.956342645	0.998543871	1.018123666	1.057982718	1.017354666	1.022612638	0.996878893	0.972514704	1.001439198	0.968546228	0.996546089	1.00648782
O70161	0.905982815	0.932479091	0.935885379	0.992402936	1.002867605	1	1.045510095	1.003707785	1.024912418	1.140022256	1.015926103	1.064174433
P26041	1.137547283	1.069617832	1.046684427	0.962486358	1.028107503	1.008380621	0.988978868	0.981698275	1.004172083	0.932608583	0.945730118	0.912308478
Q80TE7	0.948030957	0.963167139	0.957199125	0.994586587	0.997978996	0.999713882	1.018198077	1.01007355	1.005711036	1.046809142	1.030489276	1.059931936
Q9Z218	0.930756637	0.964744921	1.002735362	1.000524538	1.018486879	0.982699136	0.964509808	1.046046455	0.954442663	1.029522823	1.087169189	1.037211982
Q80TV8	1.039518501	0.976771991	1.00824511	0.975236168	0.973228966	0.960000949	1.044715355	1.048157982	1.041614688	0.961104479	0.970694947	0.99878012
Q64514	1.00895268	0.972303883	0.993244625	0.884611808	0.862394737	0.828820283	1.126026626	1.138621062	1.28307387	0.881831119	0.959419229	0.954243538
A2AQ25	1.032812842	1.029659656	0.966789719	0.958309361	0.958330614	0.992360894	1.059577419	0.968354693	0.99612056	1.108476594	0.986506238	1.012757535

Q9QXY6	0.993992104	1.005333627	1.004263477	0.993695623	1.047001907	1.048758066	1.001302229	0.994224718	0.993260528	1.048683316	0.960545198	0.9624266
P63011	0.907616666	0.938264683	0.986853811	1.044819509	0.970036901	0.947086278	0.974185834	1.014474384	1.051364581	0.948656787	1.076227254	1.115878724
P10649	1.129238268	1.084873829	1.124397125	1.080819249	1.163140712	1.184149278	0.923826607	0.918048252	0.887122119	0.883832498	0.851322119	0.82629182
P61164	1.06219176	1.030212003	1.025541803	1.020806219	1.017863937	1.025133884	0.98558284	0.99939972	0.990126553	0.926623556	0.957648039	0.972275575
Q9CWS0	0.961409319	0.983267506	1.027948199	1.07832383	1.120072956	1.059194478	0.893566034	0.965828542	0.888417853	1.021300043	1.04429412	1.01901985
Q6ZPJ3	1.004485851	0.990090951	0.994615852	1.033896603	1.014074499	1.015502172	0.987998911	0.986073299	0.990903058	0.984246912	1.008566721	1.014505695
Q8K212	0.965421791	0.976959903	0.987443495	1.014109632	1.036031328	1.006484659	0.990706207	0.981570473	0.979639665	1.059321517	1.028178709	1.022951877
P04370	1.40381474	1.438486216	1.167544655	1.36974652	1.140177835	1.350719853	0.635371106	0.770055958	0.775557289	0.581902432	0.733120748	0.707694775
Q8BGD9	0.939544772	0.947167735	0.962393355	1.009464924	0.99910957	1.034713403	1.022448745	1.000777395	1.019549565	1.074370258	0.997449294	1.040467107
P70168	0.978115494	0.96352459	1.002440875	1.001871582	1.001506291	1.012793861	0.991896295	0.967958058	1.016570621	0.994649305	1.031513758	1.052725371
Q3UTJ2	0.969148572	0.928981001	0.920769181	0.986513925	0.932172915	0.977673705	1.0592909	1.025150027	0.995087495	1.113959559	1.059743838	1.083198314
Q99JY9	0.937534344	0.993501124	0.995348308	1.036940557	0.993443631	0.959394682	0.962841986	1.009320368	1.023691629	0.917400393	1.065812464	1.078574
Q9CR68	1.019353041	0.989043642	1.021287294	1.019255603	1.063123312	1.028618152	0.955563911	0.983048368	0.914635854	1.147872875	1.000106168	0.966751137
P08003	1.000221793	0.94889855	0.984926433	0.955811662	0.989401997	0.985733657	1.047959249	1.035570435	1.017726931	1.042076805	1.017126672	0.997803761
P00920	1.198735198	1.097266469	1.116550989	1.015405655	1.070413398	1.023830506	0.943763034	0.983347498	1.062943077	0.866030849	0.824747313	0.811984302
O35136	0.912457628	0.904354009	0.947335208	0.983128762	0.963693924	0.967665711	1.033971492	1.013978049	1.040262063	1.071043431	1.0745573	1.107396824
Q921M7	0.993886129	0.970504029	0.991938145	1.005776701	0.965267885	1.042898	1.008528905	1.047935656	1.043717098	0.914627195	0.963470901	1.036593171
Q60625	0.811836111	0.939124781	0.959513629	1.081972276	0.98373633	0.960845139	0.973966369	1.012991117	1.026239686	0.906105399	1.125145838	1.167388416
Q3URD3	0.998434126	0.981842097	0.975981585	1.003498281	0.996757202	1.026056355	0.997943089	1.001876984	1.00978509	1.05129965	0.985798184	1.020152209
Q9JLB0	1.022378466	0.97498487	0.998596388	1.006148612	0.977564895	1.000346214	1.000671326	0.994070811	1.005098924	0.99424015	1.011200572	1.037146264
P20444	0.933189666	0.953327218	0.996966308	1.063121113	1.086068077	0.973452819	0.927367957	0.996801858	0.965001948	0.985867711	1.096391965	1.036686013
Q922B2	1.030932014	0.99395047	0.993036989	0.973839763	1.025668327	1.032729611	0.994408565	1.009859424	1.020847323	1.036170338	0.971981556	0.960216794

P13707	1.029453159	1.018298874	0.959886231	0.993058253	1.003639434	0.99904277	1.028240106	0.964808125	1.060984879	0.936737788	0.994081832	1.008494126
Q80TB8	0.987447384	0.999134976	1.18486049	1.00243193	0.970176169	0.998898097	0.914484773	1.050868512	0.972690535	0.873885838	1.008734815	0.997328752
Q91YQ5	1.011026832	0.980000876	0.984339488	0.982875772	1.005357732	0.991665849	0.998928919	1.00441435	0.974212514	1.027252411	1.044593449	1.027788257
Q9D394	1.00797405	0.98542381	0.979570297	0.998135222	1.017780729	1.050071345	0.986669873	0.997854446	0.991726391	1.020824321	0.979626988	1.029275742
Q9D8Y0	1.100320075	1.070224258	0.954766401	0.976250228	1.092003757	1.083152031	1.006197595	0.919317922	0.967370239	1.040525874	0.935822738	0.93777913
P50247	1.039608511	0.99191579	1.013374601	1.028277059	1.005467565	1.022806628	0.984672517	1.023654476	1.004452182	0.959497374	0.95668206	0.990899038
Q812A2	0.914085965	0.950248436	0.982386565	1.006298391	1.002962847	0.999342491	1.02294409	1.00043466	0.989944489	1.041467852	1.05696878	1.05465079
P10605	1.031934303	0.987938116	1.022914702	1.007705816	1.062037784	0.99967052	0.982223783	0.95425009	1.000731034	0.952924589	1.007875668	1.004059055
O88643	1.017894781	0.999824098	0.985452145	0.974079562	0.974130505	1.012113052	1.017263248	1.000890844	1.01367614	1.100325833	0.966443944	1.006065027
P60843	0.979196822	0.95944552	1.001190925	0.933909188	0.981253984	0.971040589	1.095825849	1.042807499	1.05461063	1.046573027	0.978257331	0.969508577
Q02248	0.952322403	0.979508055	0.978033947	0.985648983	1.000164999	0.975200355	1.022017477	1.016740922	0.987148002	1.057141538	1.053830143	1.022167511
Q91ZZ3	0.921354775	0.935911475	0.994663925	1.003002432	1.056411847	1.083492967	1.04811922	0.892823983	1.003878034	0.95188806	1.034980967	1.07133046
Q99JR1	1.010347126	1.002119557	1.007449153	0.98188452	0.982586596	1.027901385	1.017170266	0.990194118	0.987465306	1.005529463	0.992490267	1.023387973
P11276	1.203940386	1.217107237	1.134111625	0.812724034	0.737975288	0.759319313	1.197514524	1.130024436	1.255142523	0.962790325	0.761012998	0.786562822
O55106	0.913510214	1.008052594	1.004090071	1.122864873	1.020453547	1.041107633	0.988502405	1.028916143	0.966193442	0.895046905	0.987388888	1.012837211
Q9EQH3	0.965483144	0.971986288	0.997555101	1.009158929	1.046890327	0.993282211	0.970441196	0.946273565	0.957662007	1.140122312	1.076060911	1.004443046
Q8BKZ9	1.001659556	0.971151659	0.986051327	1.013114032	0.976121287	1.014464118	1.016012573	1.010308848	1.0264748	0.959153075	0.999610047	1.02887189
P28661	1.129882725	1.059484765	0.987516793	1.028178419	1.104196338	1.055164392	0.928064319	0.941986229	0.979369861	0.984938836	0.946331221	0.922758255
Q9Z0X1	1.044846062	0.964614445	0.952585282	0.995977201	0.983812072	1.052007101	0.998704348	0.98360312	0.990313144	1.047803601	1.000884892	1.042279953
P51863	0.902039482	0.958316501	0.988615175	1.028331686	0.929978861	0.989215398	0.9994	1.009281714	0.975275117	0.939448882	1.128496783	1.120263388
Q8C0T5	0.985419628	1.002219514	0.990126146	1.032649399	1.026119551	1.004670699	0.993872826	1.005796686	0.989617129	0.992810347	1.01034587	0.989903036
Q99LX0	1.051176544	1.042808878	1.06943531	1.065463393	1.095569283	1.08606621	0.936955362	1.010470232	0.943815661	0.947307475	0.898103316	0.89905216

P12367	1.001526324	0.967207971	1.036521167	1.010522506	0.97666755	1.028181167	1.01594369	0.998146306	0.985066419	1.017075065	0.983990262	1.011514137
P84309	0.897375965	0.965629049	0.966912324	1.097883121	1.126972483	1.058512132	0.924433568	0.925387174	0.924038608	1.012505923	1.080112226	1.067392568
Q0KL02	0.942978342	0.959337847	0.956396369	0.954978505	0.975887824	0.932661803	1.027034728	1.001251701	0.993481217	1.118546147	1.097720788	1.083952303
Q9QWY8	0.964857482	0.976144414	0.948361875	0.918118254	0.898136727	0.947287267	1.121852028	1.031691214	1.11011103	1.075804538	0.976218927	1.046649496
Q61885	1.408087454	1.285875771	1.173214668	1.293473752	1.429491876	1.283536408	0.675550234	0.717122335	0.730959927	0.701351454	0.725808261	0.711262524
Q3UHD9	0.889050061	0.953421275	0.974379515	1.049429407	1.008407031	0.98208119	0.98515306	0.999692592	1.012522386	0.956144134	1.089361551	1.083365127
Q62059	1.177958145	1.142060934	1.07069943	1.146888304	1.179358868	1.173859659	0.838048054	0.853768034	0.862481288	0.835405714	0.894586043	0.891702527
Q5SVL6	0.898775569	1.004145651	0.973268216	1.039114063	0.986842761	0.951719332	0.990848005	1.005951486	1.020282107	0.976354115	1.061644668	1.084676783
P54227	0.999138511	1.014440016	1.015775355	1.033887958	0.950056967	0.997949267	1.057080482	1.007276207	1.047877015	0.886060643	0.949484206	1.013305074
Q8BG39	0.961266957	1.051209283	0.993324371	1.003545674	0.936699121	0.978580316	1.013798762	1.018663186	1.017028082	1.01100878	1.003970963	1.026929513
Q9JLV5	0.988262564	0.999545517	1.010455241	1.010990195	1.020269335	1.00471568	0.998021368	1.010226893	0.970418637	1.011246924	0.991815186	1.018456914
Q9Z0R4	0.953740649	0.918419181	0.987065936	1.00602648	0.972065494	1.018439923	1.021105804	0.996899459	1.001198685	1.069426011	1.029985138	1.066362777
P16627	0.986477234	0.963771926	0.973360535	0.943211726	1.020746421	1.046930912	1.016664327	0.941241292	0.958759609	1.108099058	1.047022491	1.062291071
Q9QUR6	0.979905462	0.994406534	0.966975543	1.009864968	0.995275731	0.998432162	1.010506661	0.995426479	0.992640067	1.010165831	1.034239801	1.036167174
P62823	0.85535555	0.957701769	1.042740482	1.115558876	0.97700033	0.905433296	0.902490831	1.057427535	1.003343243	0.752935564	1.163063581	1.152321928
P97351	1.033000512	1.0013196	0.986767559	0.978769739	0.930082991	1.00228063	1.065087645	1.015849135	1.068364019	0.956156082	0.946846348	1.013543024
O88485	0.971905559	0.971365715	0.992703379	1.004165372	1.004115162	1.017662256	1.010457692	0.988702896	0.984504835	1.075158514	1.014054247	1.017855125
Q5SYD0	1.29030578	1.133625609	1.059043489	1.116988545	1.253678807	1.199281397	0.800481048	0.852561718	0.85835729	0.847857411	0.85932814	0.826461002
F6ZDS4	0.951830591	0.979437832	0.985650971	0.997885566	1.004928814	1.029901161	1.024966362	1.014686739	0.992822252	1.020550923	1.019066026	1
Q60634	1.019040469	1.086136607	1.011630805	1.025329358	1.016490306	1.020224624	0.970671464	0.972461693	0.962807517	0.96959285	1.009222108	0.961764511
Q91VR5	0.978528062	0.963272899	0.991935604	1.016660248	1.014524638	1.002742103	1.000367669	0.984231452	0.993600898	1.002873602	1.035599194	1.037174247
B2RSH2	0.979994399	1.017702899	1.020364598	1.041465656	1.10327542	1.010297898	0.92957359	0.956450149	0.9532123	0.923362768	1.063274488	1.004437527

Q9ESJ4	0.97258509	1.049891419	1.007860915	0.961361277	0.982784792	0.967895538	1.027488004	1.00786924	1.036288807	1.057251899	0.988196723	0.971590966
Q9WVK8	1.021533695	0.98313493	0.986554025	0.992379196	0.927855037	0.969599498	1.028843978	1.033680586	1.054362889	0.980063222	0.97358672	1.055745607
Q9JKK7	0.985944549	1.037053587	1.05169485	1.043156293	1.052298251	1.033637545	0.976197491	0.992637256	0.963592536	0.916132434	1.007496005	0.935980893
Q9JHK4	1.015306543	1.040567096	1.058066751	0.997105458	1.002772333	0.924764731	0.973216348	1.007355563	0.996571081	0.98937003	1.020056586	0.988311494
Q8BUV3	0.952239046	0.943071998	0.968964798	1.011081588	1.008994503	1.029085304	1.004912043	0.996031978	0.994383316	0.996786555	1.047209081	1.061710483
Q9Z1Z0	1.021235571	1.006110133	1.001297179	1.002236627	1.002732528	0.993376354	0.980515102	0.999021099	0.98207034	1.020812862	1.014864456	1.013579416
Q91WD5	1.012429433	0.983204133	0.980832966	1.054466036	1.008985365	1.036016983	0.979765813	0.973850775	1.005152584	0.98191766	0.983174824	1.033493767
Q8C0M9	1.052079564	1.048653581	1.03671884	1.024373036	1.022766514	1.012057834	0.950412873	0.983708852	0.96611846	0.991262714	0.972112388	0.984224427
Q9DC69	1.063261254	0.969094352	0.994647402	1.008022774	1.004537315	1.063623428	1.02208627	1.003699544	1.020239989	0.963849447	0.926693182	0.987840508
Q80U63	0.943555978	0.988835156	0.967045552	1.003900851	0.969874253	0.99127239	1.005533152	0.98369931	1.025553588	0.993422959	1.038154244	1.097493933
Q8R5H6	0.87304062	0.952867919	0.914481089	1.024562014	1.084557859	1.063396226	1.016617053	0.961452035	0.933577068	1.237529291	1.00972314	1.060033248
Q91ZJ5	0.962310384	0.987365723	0.963249624	0.993258611	0.962529091	0.998243565	1.035229005	1.006998606	1.047528866	0.940196537	1.023457556	1.060820235
Q99PT1	1.017009577	1.015340163	1.034178567	1.006248495	1.041808762	1.012120052	1.01218769	1.026033451	0.999462819	0.988714581	0.93655034	0.938596992
Q99PV0	0.987340393	1.000331226	0.990418748	0.996439754	0.980968992	0.994398086	0.999174782	0.984428999	1.031198622	1.016590578	1.011106114	1.031684598
Q99L47	1.024493467	0.993037957	1.043211582	1.063043997	1.039037525	1.058631206	0.966492924	0.994332061	0.980311942	0.874263205	0.957324688	1.001462555
P51150	0.984029111	0.994442395	0.998430841	1.019947413	0.978011443	1.063050506	1.029034862	1.031798134	1.000101806	0.896575867	0.973663881	1.012589826
O88844	1.045241688	0.996853493	1.006522772	1.00241073	0.991655398	1.025302375	1.008860137	0.974158227	0.976396627	0.948786262	1.007151454	1.02972109
E9PV24	1.1980087	1.3078969	1.154459641	0.773734775	0.726470599	0.663656864	1.167095345	1.239188333	1.312783775	0.915201031	0.723770774	0.739675759
Q8CHH9	1.027484854	0.981371312	0.989332438	0.999117329	1.023230423	1.037217905	1.01459136	0.96343886	0.943696674	1.058126748	1.020341766	1.002401228
B9EKR1	0.962015177	0.971465791	0.98426931	1.01178855	1.002575735	1.022171308	0.991415395	0.983476329	0.968933789	1.02924665	1.04454053	1.062701758
Q9WTX5	0.904490068	0.92103255	0.977708368	0.983523701	1.175235261	1.112314687	0.941163895	0.892857875	0.951452788	0.980207191	1.126623791	1.04794901
Q8CHG7	0.994124806	1.110307807	0.988060214	0.971279616	0.961389625	0.946528854	1.023000577	0.987715492	1.009786109	1.020586217	1.041509496	0.961792105

P49443	0.948249891	0.965782445	1.000902249	0.995276558	1.016495467	1.021479397	1.003838198	0.999152903	1.001850016	0.988148867	1.022457034	1.046081108
P35235	0.980842491	0.968597379	1.000307973	1.026139299	1.060459259	1.040451823	0.97193502	0.977103533	0.97894277	1.001351191	1.01698505	1.009872712
Q8BLQ9	0.935235242	0.989034499	0.932684101	1.070284557	1.047673053	1.000714804	0.946533362	0.998421211	1.028980028	0.925112399	1.062935884	1.052221695
Q8BIJ6	1.029315669	0.997357136	1.020076696	1.056291472	1.017154533	0.963289326	0.946954702	0.959538464	0.992219204	0.921385437	1.040272125	1.058777728
Q9CWJ9	1.032022451	1.015545271	1.003630134	1.011710225	0.991540233	1.055276561	0.978536585	0.981218234	1.022898805	0.979744625	0.967591009	0.987864961
P55012	1.107662916	1.018203455	1.008973649	1.069774554	1.089064233	1.09932349	0.924011585	0.945171605	0.958332249	0.943286341	0.94157919	0.949434452
Q5DU25	0.93825126	0.935926492	0.944261932	0.961285411	0.949741547	0.980601281	1.087211236	1.014367796	1.0060395	1.177007738	1.051217072	1.020204689
Q62419	0.922950013	0.966139464	0.942169211	0.974458365	0.961041221	0.983986727	1.0615557	1.01963388	1.012185398	1.038733632	1.057460524	1.0714172
P19536	0.970190487	1.031758622	0.96509071	1.130335334	0.929122978	0.981619952	1.000030147	1.005944504	1.039893526	0.82158923	1.030379074	1.040173139
Q80TK0	0.902899955	0.983720654	0.922866251	0.989471945	0.99141937	0.97536747	1.028397153	1.010334265	1.01329151	1.118022897	1.041306339	1.076337325
Q8BKC5	0.939299061	0.956160723	1.008105739	1.005349583	1.006218249	0.965971134	0.993503903	0.999230568	1.022648186	0.984583857	1.062649663	1.051851567
Q9QYB5	0.95597119	0.995035516	1.015917128	1.01745154	1.065405193	1.059089018	0.98056303	0.961791715	0.955094547	1.066213572	1.003545399	0.985108456
Q8CJ19	0.935680347	1.004543495	0.997523477	0.994542874	0.982681925	0.97314585	1.009495487	1.027601739	1.035633562	0.970180156	1.035801462	1.020058323
O54983	0.914840722	0.950491065	1.031326066	1.076561779	1.055919967	1.081491986	0.944985779	1.026371828	1.001305787	0.849213358	0.992762608	1.040237431
Q99KK7	0.96540482	0.962969858	0.96576392	0.992811321	0.989578302	0.995517609	1.022256689	1.020709279	1.010565521	1.021600775	1.021361742	1.053279185
Q9D8N0	0.943486951	0.949886019	0.976257516	0.962410292	0.954589858	0.964788278	1.040419562	1.000790538	1.039027272	1.045748449	1.045355913	1.09213892
Q5SWU9	1.073121386	1.047894823	1.006645981	1.012516359	1.007881578	1.03439767	0.978271265	0.961767847	0.966179702	0.999126403	0.974606117	0.987892887
Q07076	1.042542999	1.048241655	1.04938769	1.055690077	0.88787827	1.016676226	1.062635674	0.993551051	1.022392235	0.984948307	0.905861776	0.956232708
Q9JJK2	0.924500013	0.945392214	0.968855117	1.019641913	1.000622186	0.993870702	1.011421039	0.995457748	0.98520893	1.095491415	1.026440444	1.089745897
Q9QXL2	1.070104005	1.004928531	0.988340457	1.001973992	1.048621793	1.077023747	0.98618008	0.966321575	0.952477036	1.043156157	0.972274668	0.960212453
Q8BGT8	0.960444586	0.955362884	1.009468523	1.02421172	0.995302066	1.008261788	0.987513267	0.999735033	0.988642406	1.020107253	1.021519676	1.058312812
P63044	0.973585413	1.20022447	0.965599851	1.048190838	0.779149825	1.055222505	0.98066992	1.091912913	1.021040198	0.715820588	0.98429509	1.086058578

Q9JLJ2	1.119750335	1.030135291	1.117735325	1.061728442	1.051376899	1.069840937	0.908613043	0.969744746	0.946870096	0.86294518	0.924766315	0.952225797
Q6P9K9	1.009397983	0.980837956	0.99089027	1.000184762	1.000388461	0.990234441	1.019219262	1.013369958	0.989187837	1.050141226	1.006179128	0.993123295
P61161	0.91167031	0.937988727	0.953646506	0.989390589	1.050800034	0.97448187	1.032051945	0.999544751	0.981566705	1.056054072	1.095639649	1.039590562
Q9DCT2	0.986501131	0.958409311	0.98573415	0.998023201	0.953327389	1.067630893	1.025779461	1.010085376	1.027337824	0.916789277	0.998424322	1.054585472
O88487	1.028046107	1.011327501	0.980871203	1.00198936	1.065653263	1.025489514	0.980816519	0.963483262	0.970262819	1.071267662	1.000031471	0.971870256
Q8R5C5	0.986564998	0.978959612	1.007654496	1.003481261	0.996563983	0.98046002	1.034616389	1.043529644	1.011746693	1.003127916	0.982226409	0.987221939
Q6NZJ6	1.020995905	1.076142272	0.937425346	0.932831996	1.035334551	0.979180343	0.988924114	0.961308603	1.002179369	1.202604696	1.002848563	0.974335407
Q6NXK7	0.996770996	0.962544569	0.982424944	1.007237965	1.001964034	1.005562267	1.01895722	0.997375161	0.996496538	1.035834354	1.017988244	1.01270028
P01942	1.418191514	1.422940214	1.450829493	0.304312336	0.270501858	0.21192078	1.442489711	1.915916986	2.259962282	0.325472843	0.259357844	0.166211158
Q80YN3	1.069341546	1.100586435	1.01715967	1.098694343	0.97776852	0.972858825	0.956767134	0.967327249	1.059961054	0.935876213	0.897683042	0.975137563
A2ALS5	0.969092753	0.975438179	0.964435984	1.050495418	1.006524584	1.017066302	0.982077835	0.981147231	1.019020347	0.964206219	1.017818078	1.065062724
Q93092	1.017803142	1.034996719	1.006677348	1.080546475	1.145297991	1.067815127	0.948325596	1.000849867	0.971246474	0.826292672	0.959001971	0.926536093
P32921	0.986006222	0.994106414	0.996866304	1.007070992	0.993596701	0.976788127	0.998492889	1.019984932	1.013701714	0.971025502	1.020777555	1.025044672
Q7TT50	0.976794444	0.979015135	0.996690899	1.018201296	0.971363497	0.980786235	0.996899051	1.004009304	1.027805245	0.974471185	1.020797181	1.057052177
O54991	1.080518988	1.029912326	1.054954875	1.071379386	1.044973789	1.046111169	0.93534122	0.955083514	1.010461209	0.871147628	0.929643332	0.978257245
Q3UVX5	0.902124867	0.977754875	1.0112916	1.058449952	1.014099485	0.996122921	0.975143287	1.031021797	1.000209114	0.939960982	1.038709552	1.040305987
Q99MN9	1.041020349	1.051240829	1.070755398	1.01835741	0.911251692	0.947967783	0.985960886	1.045380986	1.084934561	0.868257483	0.962683526	0.968301457
Q8JZQ2	1.008616235	0.982480541	1.004149065	0.976414019	0.977261629	0.997438386	1.038420147	1.004296287	1.025966558	1.007287159	0.988110133	1.007991548
P23953	1.275186743	1.341534859	1.270313201	0.721169895	0.691468685	0.671524807	1.324513804	1.180256715	1.340537012	0.706873017	0.666576161	0.63181999
Q62418	0.945764679	0.954927262	0.995297082	1.01502785	1.000535297	0.996435167	0.997210083	0.999378571	1.018518572	0.983639246	1.025427526	1.074462205
Q8BGZ1	0.884432504	0.966459785	0.994180333	1.029877419	1.061029965	0.945870976	0.973870252	1.030706231	0.951435781	1.012093616	1.103716243	1.054586982
Q921G7	1.049046208	0.997880606	1.045850398	1.007758318	0.975201164	0.967056368	0.994431219	1.00903404	1.02335822	0.973568515	0.972842262	0.999298399

Q9CRB9	1.036006375	1.097834687	0.987960208	0.985329095	1.043104367	1.022135007	0.974575874	0.97360167	1.024582291	0.92674084	0.972745846	0.962410787
P62137	0.978683967	0.979123257	1.02389275	1.060849561	1.037830468	0.969478886	0.903670722	1.000838292	0.98725164	0.939099973	1.054717675	1.068644582
Q9CR62	1.038331689	0.984773417	1.025634338	1.018679972	1.055936338	1.048200234	0.987389126	0.971803356	0.96443611	0.998978154	0.961310203	0.992346111
Q99LC5	1.107360048	0.988364979	1.005300613	1.059926803	1.014732301	1.090504939	0.937129226	0.9782272	0.985201369	0.952410311	0.939592883	0.988620663
Q99K85	1.045553852	1.023817171	1.040404713	1.046421381	1.126600923	1.043804238	0.921154709	0.952848679	0.980024544	0.912572339	0.96520374	0.964028968
Q91W50	0.978031483	0.977694661	0.978765876	0.99267555	0.95166755	0.988565369	1.016991552	1.020793947	1.024597182	1.028466082	1.004230598	1.062790385
O55023	1.051299004	0.984721375	0.989085098	1.031013706	1.005319802	1.065511629	1.001449662	0.976047119	1.018374138	0.965508327	0.945375113	0.997164853
P60335	1.100034305	1.020756172	0.975849855	1.099601741	0.788793441	1.352916804	0.776980691	1.074127753	0.788059406	1.04837759	1.002975071	1.091126561
P26645	0.895626519	0.895215652	1.072421456	0.989513115	0.909379855	0.919784501	1.078556668	1.126141217	1.151485551	0.982290225	0.953053921	0.981289004
Q9D0M3	1.013971452	1.027019258	1.020567681	1.075008911	1.016363548	1.039984255	0.917838809	1.003718812	0.93458831	0.815748239	1.029546108	1.078166275
P55066	0.955562675	1.001921758	1.018346272	1.097831151	1.092651762	1.034202654	0.920462245	0.939132092	0.936307753	0.934209137	1.035270167	1.055849689
A2CG49	0.935289256	1.019531275	0.929958355	0.931438358	0.956965501	0.925691722	1.058535514	1.046942067	1.015994452	1.106313054	1.076809105	1.026152966
Q99LF4	1.041522433	0.983998525	1.002523634	0.972404635	0.989568106	1.014418384	1.019550542	0.986007898	1.047571922	0.967456703	0.99209807	0.988637581
P12023	1.088561731	1.078903676	1.026320506	0.941894764	0.971968893	0.939271652	1.066680431	1.02091922	1.065857558	0.945464109	0.932700974	0.916091754
Q9JKR6	0.971000732	0.961841658	0.996727924	0.987747521	0.976815401	1.010164231	1.043613878	1.023487156	1.011281251	1.040284493	0.987922404	1.018559964
Q9EQF6	0.924471066	0.897299603	0.952050453	0.920360554	0.923096213	0.942159329	1.072195978	1.083855079	1.063231867	1.038479728	1.087137793	1.078648487
Q8CJ40	0.973935359	1.105787794	1.011671642	1.060704686	0.991214802	1.00527516	0.963347417	1.016970086	0.952988928	0.899213647	1.019278967	0.989761996
Q8BP47	1.022134153	1.024999968	1.042791671	1.026920622	1.056394014	0.974149705	0.953431563	0.998873583	0.947006537	0.977153364	1.015137553	0.989297773
P53810	0.996756719	0.981996395	0.995231822	1.026353209	1.004017185	0.99320101	1.028814085	1.013348165	0.979345843	1.046630027	0.99445181	0.984488315
P26231	1.057271298	1.028274125	1.02585881	1.003023832	1.023928809	0.990280525	0.999981115	0.992737845	0.98666782	0.997239639	0.972649032	0.957836793
P28271	1.01738576	0.968672766	0.998485942	1.014504967	0.987522756	1.004547069	0.986459245	1.008076721	1.024550203	0.960649956	1.004271242	1.032626504
Q99JF8	0.977013284	0.942546034	1.026310736	1.047379781	1.065531536	1.084554097	0.975640936	0.999980593	0.958887922	1.098730355	0.937161029	0.974258375

P50544	1.059723982	0.986486208	1.029769145	0.990905554	1.061411358	1.053264686	0.980940144	0.992061699	0.948780455	1.013557869	0.984740186	0.948036755
Q9QVP9	1.009931148	1.034382643	0.977219242	1.05012467	1.026535955	1.058026511	0.973472841	1.000573193	0.955529581	0.991753197	0.973862431	0.994267883
Q8BYR5	1.013703325	1.011089787	0.983220852	0.997740436	0.959087145	0.989250785	1.016268123	0.997009964	1.027845179	0.984875083	1.012083766	1.018959102
Q8K010	1.037310662	1.014500103	1.000006694	0.99496726	1.04929395	1.055131135	0.97938804	0.948377327	0.978535779	0.998978247	0.997550765	0.987520516
Q3UH60	1.056983607	1.033482887	1.003760489	1.019499101	1.004958361	1.000055867	0.990015076	0.992233603	0.998976229	1.00731821	0.956675246	0.981650395
P58871	0.939606842	0.977234645	0.982160329	0.979658633	0.985420148	0.982963723	1.045723223	1.024738455	0.995907786	1.126085236	1.014127809	1.004029688
Q61656	0.973397724	0.987163372	1.034489737	0.975299188	1.023390522	0.960307024	1.016767411	1.033732001	0.980663074	1.038932562	1.025102209	0.975027215
Q3UPL0	0.99448001	0.966663745	0.956901295	0.965035932	0.947935915	0.997078739	1.05049813	1.013685861	1.03709286	1.065533021	0.998848244	1.043718958
Q99LC3	1.01729709	0.995310539	1.012115109	1.07431726	1.070345282	1.041287879	0.984027781	0.997681518	0.978254614	1.001729015	0.95289384	0.922630246
E9PUL5	0.988053347	0.966584924	0.919299813	0.930389209	1.135455568	0.977273865	0.98476886	0.86439753	1.012348551	1.566406089	0.921917378	1.020891754
O35129	1.000486023	0.962730247	0.974005911	0.979678858	0.987138663	1.037838842	1.04986509	1.01954961	1.053981348	0.910839933	0.980369594	1.02063388
Q60676	0.980946625	0.963303862	0.975853032	0.995723668	1.049291642	1.021232315	0.989635487	0.999795911	0.971812983	1.023318675	1.046739247	1.014705483
Q9Z2W9	0.907963286	0.972091537	0.955544658	0.964236964	1.032767148	0.977034414	1.029398941	0.991965925	0.962722369	1.080743927	1.100841311	1.057651689
Q9WTQ5	1.054556647	1.008191007	1.030880234	1.006070336	0.994778881	1.000637895	0.979034496	1.004932055	1.003934458	1.031835589	0.963604515	0.970294776
P62908	1.011329117	0.99226388	0.976985977	0.948361714	0.924500954	0.990662581	1.06972423	1.019716096	1.032935548	1.055574944	0.973198315	1.039082445
O88712	0.98927035	0.994621013	0.97679789	1.009746952	0.941767242	0.99069068	1.018300318	0.980412003	1.002686468	1.015566714	1.035923595	1.067611684
Q8R3V5	0.928233236	0.979912341	0.981078016	1.018591596	0.945846591	1.00080845	1.019144442	1.021141689	1.024982971	0.926241466	1.037050827	1.090392199
Q8R464	1.077265642	1.002776218	0.97858164	0.999326665	1.026272592	1.040062599	0.973924694	0.951153994	1.03332466	0.97100996	0.982191037	0.994801264
Q60829	0.780611999	0.952745059	0.963114869	1.358509152	1.155513581	1.12736201	0.882233575	0.943658545	0.926585248	1.046376984	0.994486165	0.954908273
P46664	0.991253392	0.995367505	0.998296599	1.009897037	1.005522252	0.963266374	0.996681435	1.005905251	1.011710021	0.964658885	1.026302359	1.033894772
Q5SRX1	0.985949652	1.011630383	0.978337626	0.987806087	1.007456751	1.029318622	1.020708815	0.982023263	1.024437622	1.015727172	0.981546593	1.004960158
Q9EPJ9	1.027939187	1.031931616	1.01403649	1.048684654	1.048224962	1.018431771	0.94174666	0.938587754	0.987306361	0.975832175	1.011300966	0.990140878

Q99MK8	0.964127266	0.98382057	1.03351764	0.959533629	0.97878946	0.924058649	1.032103657	1.0517734	1.076320784	0.95441555	1.014406679	0.99597791
Q80U49	1.039510468	0.965252785	0.977963214	1.029637964	0.957832534	1.073260423	0.996432038	0.959519285	1.006617599	1.029617472	0.965618352	1.054807269
P62141	0.95418008	0.982755085	0.980539985	1.018263793	1.036966347	1.058181014	0.965538765	1.015687926	0.958024682	1.050291762	1.032460863	0.994670912
Q68FF6	0.992461273	0.985473114	1.000599513	1.041680574	0.972751855	0.999776834	0.98290137	0.976784898	1.029948707	0.897410793	1.030449047	1.068162421
Q6P1F6	0.964058777	0.960382571	0.970868581	1.046380884	1.006533079	1.001971901	0.965177685	0.992454495	1.015765082	0.956348599	1.039522739	1.085539414
O70443	0.988612992	0.979055381	0.983495913	1.014872485	1.031684511	1.000354426	0.999848661	0.996686162	0.987750108	1.029174563	1.018474963	1.007282881
Q9JIF7	0.979012408	0.948352193	0.979974495	0.975431059	0.974856493	0.99504444	1.042100337	1.000548734	1.006435062	1.02126974	1.040390533	1.052929936
Q80TZ3	0.960797934	1.005653453	1.006200718	1.019855931	1.005959468	0.990946801	0.970789327	0.985015599	0.977687189	0.971524863	1.066189407	1.044584538
Q9D819	0.94314053	0.951537874	0.986527089	1.010321435	1.021800571	0.978854544	0.962541041	0.995053926	0.978666569	0.968857075	1.096849588	1.104577422
P97797	0.948519445	0.97870614	0.978765152	1.046448035	1.020737539	1.045024853	0.998433342	0.978048681	0.977338222	1.064099706	1.01380927	1.004334111
P19783	1.052780992	1.011686992	1.010526913	1.024035984	1.007993665	1.029755317	0.988655256	0.997289258	0.965063015	0.983240019	0.984040303	0.980315923
Q8VDJ3	1.022847336	0.995606673	0.978402425	0.96431628	0.973126395	0.995624835	1.031019684	0.99967307	1.012996845	1.045686484	0.998225895	1.021305968
O55234	0.986023971	0.989113361	0.986867365	0.988663521	1.016305944	1.00426488	1.019065134	1.021703889	0.9788837	1.009995344	1.030392318	0.989252892
Q8VEM8	1.037963835	0.980200574	1.0057063	1.057559292	1.002501673	1.07955967	0.964485104	0.958629138	0.969460014	0.970219435	0.993539331	1.016643527
Q8CIE6	0.9656121	0.98203424	1.000458709	1.013315404	0.987873013	0.972799189	0.995954242	1.03362132	0.973191917	0.998687045	1.044689606	1.045303317
Q3V3R1	1.027863592	0.993460056	0.978778561	0.991588947	1.002886209	1.013177928	1.039382034	1.015360309	0.982799094	1.003851853	0.971562736	1.013087528
Q9Z2I0	1.023164839	1.048109031	1.008181338	1.017755003	0.979115866	1.0768545	1.007678788	1.010977192	1.009929385	0.888809331	0.944999388	0.974479636
Q9R0P5	0.979605515	1.029859255	0.983529776	1.011137804	1.057362319	0.967852859	0.944419019	0.949191139	0.946134342	0.995582638	1.08384715	1.080862989
O08529	1.015832589	1.007267511	0.994016287	1.027062092	1.030926679	1.021955292	0.956640586	0.987190923	0.968365308	0.982956033	1.016395564	1.023522031
Q61171	1.029417422	1.015141757	1.002873548	0.920597417	0.888692509	0.956589947	1.082063093	1.086642053	1.152059268	0.91217967	0.940119482	0.962870942
Q99PJ0	0.858057182	0.922625225	0.926255716	0.960000304	0.989140766	0.941068146	1.057863189	0.95992202	1.07099151	1.075422848	1.122706504	1.119565267
Q61081	1.026082111	1.029477271	1.009365547	0.966760989	1.009089326	0.975549723	1.027216259	1.004867174	0.98174826	1.064916369	0.999215009	0.954279916

Q9DBF1	1.052377575	0.985850577	1.019981785	1.030929473	1.057510262	1.104405859	0.953059555	0.932704817	0.97407271	0.970030718	0.976488944	0.985491035
P21550	0.98452106	1.015859766	1.028969499	1.05971666	1.033705548	0.994370111	0.958341084	0.971730881	1.025999989	0.871898729	0.992463269	1.041555266
Q61035	0.985148458	0.975763368	1.024726666	0.999454935	1.011579412	0.99532883	1.00600422	1.004336748	1.020927349	0.992010417	0.997017616	1.000793949
P63087	0.906257474	0.970135001	0.942325187	1.00031606	1.026220239	1.120463069	0.965752755	0.920884407	0.979924732	1.044927245	1.008039606	1.172097001
Q9EPK7	0.945992444	1.001441784	1.042025507	1.021039155	0.969052585	0.948995559	0.993742763	0.999258814	1.038488757	0.953081253	1.021975992	1.050375663
O88685	0.995422634	0.970599775	0.988129344	0.983810423	0.998291379	1.030948427	1.03136206	1.019208092	1.010874082	0.988145455	0.984305438	1.014416357
Q8BH95	1.087422129	1.040996365	1.048946637	1.06979314	1.022400784	1.036188207	0.932247434	0.955236967	0.977036674	0.877157508	0.96643056	0.995537189
P08032	1.091570917	1.086947953	1.068930205	0.819951391	0.837313086	0.796356932	1.143259702	1.222717512	1.284505358	0.908009427	0.851312797	0.797716231
Q9EQZ6	0.98349582	0.998208606	0.98745481	1.014253349	0.992153488	1.010168239	0.997314785	0.979434577	0.997855628	0.994440195	1.037387287	1.025536387
B1AWN6	0.959199262	0.956131615	1.005938222	1.021608016	0.964808353	1.026971363	1.010066135	1.007186671	0.997526274	1.027860953	1.013378013	1.036757368
Q9CWK8	0.923854542	0.929043785	1.016099003	1.011557952	1.046132332	1.034376349	1.019227167	0.969773799	0.996386424	1.085232137	1.011802583	1.006512218
Q9Z1Z2	0.948004584	0.977897449	1.016591738	1.020986697	1.00636386	0.92236274	0.957391364	1.018042281	1.000840608	0.974790368	1.083570705	1.067782387
P27612	0.966502962	0.966207924	0.975462025	0.991537362	0.973658609	0.985963288	1.006918237	1.002363521	1.031241841	0.982588284	1.050755205	1.065503671
Q99K51	1.034880853	1.048900513	1.00551547	0.999182745	1.042159983	1.004298877	1.008067788	0.987747726	0.973766147	1.001571433	0.96725349	0.967223754
A2RT62	0.854172422	0.905379398	0.914191818	1.030947065	0.978106754	0.990539374	1.020994519	1.030585263	0.983370469	1.099127154	1.08671869	1.145937151
P62962	0.936456008	0.948825795	0.97143779	0.961213124	1.170223386	0.987953212	0.967691997	0.997204925	0.941963466	1.125460141	1.074736494	0.988640973
Q9D0R2	1.004172088	0.975805009	0.991169597	0.978182443	0.971004033	0.982706207	1.043209981	1.022429531	1.034766236	0.977781797	0.993450443	1.02792594
Q99N28	0.909889778	0.925259257	0.982996643	1.051508058	0.972696134	1.024624105	1.007028502	0.990894587	0.96266587	1.051766541	1.074384509	1.078514424
Q8BSS9	0.889370388	0.919456546	0.980363338	0.967430107	0.932702997	0.956112689	1.022465762	1.032884489	1.031361442	0.994940212	1.1038446	1.14588831
Q99KY4	1.01198195	1.036011319	1.046361405	0.9762784	0.977261416	0.953392111	1.008215338	1.026034954	1.030822484	0.951180212	0.993939708	0.978761099
Q64525	1.093857971	1.054205907	1.114361499	1.121373799	1.076971734	1.125453795	0.97849066	0.969828447	0.961279269	0.694023188	0.853985811	0.909973878
Q03137	0.975633483	0.986906961	0.961956157	0.997720239	1.010690296	0.981616532	1.039325038	0.992792886	1.048996862	0.976630107	1.020628514	1.006336892

P57746	1.055400366	1.011814513	1.03618289	1.008994277	1.031765347	1.073922499	1.008233415	0.986213635	0.986546054	0.930777958	0.935491882	0.95147503
Q2M3X8	0.912916216	0.967542748	0.936283598	1.033559093	1.064030982	1.035362276	0.971952773	0.996987272	0.979149337	1.055062095	1.043838054	1.050535962
O35295	0.952867122	0.913863804	0.934352443	0.937159564	0.927712396	0.938521302	1.095663211	1.078962845	1.110698595	1.05464823	1.013659069	1.04161181
Q00897	1.375660165	1.343880579	1.116822943	0.736274898	0.797331109	0.796329807	1.160981491	1.073433105	1.205341462	0.986169644	0.71187043	0.708926437
Q99NE5	0.921033433	0.958002377	0.961869704	0.995233112	0.918024315	0.96750827	1.030710917	1.026504673	1.02174768	1.059772522	1.071459702	1.084665554
P35700	1.109045135	1.065897567	1.050925935	1.057271556	1.077455146	1.063454569	0.930153741	0.955165454	0.929183745	0.935639058	0.941724753	0.932882088
Q06890	1.087535305	1.14433016	1.086171239	0.919816681	0.98182048	0.89484186	1.081073693	1.050506813	1.049621386	0.887673933	0.910664761	0.871166057
Q9WUM4	1.004632379	1.008180988	1.04183777	1.035487702	0.999843537	0.992525712	0.953052322	0.995060 8	1.001841539	0.887167274	1.01895771	1.041848101
P54071	1.142382848	1.058924855	1.127342158	1.036812887	1.054533193	1.020200042	0.931351141	0.939947917	0.952439979	0.901502354	0.954817161	0.902018429
Q9R0Y5	1.006902751	0.989084053	1.005744051	0.987125015	1.001028394	1.057592136	1.03741224	0.97900812	1.031189751	0.954242216	0.957649474	0.999932547
Q02357	1.039532419	1.00588304	1.033616107	0.923321565	0.932681748	0.91947432	1.050342914	1.077890466	1.123688175	1.012303447	0.9439512	0.934251241
Q9Z204	0.973100691	0.948054036	0.976376317	0.96231572	1.029023073	0.991089091	1.025668596	0.996528433	1.01295284	1.270775313	0.955840641	0.993063644
P62702	1.006859591	0.950551755	0.978915966	0.948106709	0.951639794	1.035373123	1.075726263	0.999504337	0.996360616	1.133143729	0.985374729	1.00909129
Q62433	1.115717225	1.095849717	1.011243419	1.104787793	1.205965514	1.163574503	0.850573211	0.883696351	0.913059165	1.034472707	0.889851332	0.862042192
P48318	1.049073077	0.960466615	1.011337537	1.025001358	1.004603123	1.045733963	0.976876926	1.020565879	0.98289329	1.029210926	0.977422558	0.967601352
Q9CQD1	0.996387845	1.001673304	0.988907748	0.938158628	0.949360 3	0.995426158	1.066413265	1.046902324	1.050350126	1.056802242	0.962298697	0.975067962
P68181	0.981679884	0.97629484	1.008199748	0.991458505	0.991645129	1.011978813	1.057940735	1.046253603	0.996487686	1.034742257	0.96420711	0.96965934
Q8K183	0.957994739	0.931782371	1.046966799	1.074518498	1.008895624	1.086692055	0.973562279	1.02845623	0.971439589	0.919203567	0.975497567	1.025032922
Q8BK64	0.987753288	0.965573155	0.982111824	0.96345473	1.009412106	0.997829204	1.025916718	1.021133924	0.994471015	1.034266966	1.028416245	1.016129412
Q91X72	1.180617359	1.437280485	1.193174733	0.771841086	0.729426161	0.817332474	1.260536945	1.120909179	1.231702382	0.673369177	0.713133222	0.706749223
Q9DC07	0.974167811	0.954078699	0.997153254	1.000718216	1.018558642	1.030527276	1.01440629	0.973559277	0.999373093	1.028610172	1.011585851	1.030673945
P62897	0.890200565	1.28583836	0.800593592	0.782989896	0.894226111	1.184734721	1.005391877	1.217790662	0.860820854	0.497205721	1.158025122	1.209441305

O88543	1.011371064	0.998767284	1.015292402	1.000397334	0.93083583	0.988113237	1.04388385	1.023761354	1.106411249	0.880326928	0.946766411	1.012921051
Q6ZPQ6	0.956579658	0.947446968	0.968041119	1.02024733	0.965220388	1.023830596	1.049919663	1.008710644	0.985591397	1.019399912	1.027888669	1.048895244
Q05816	1.035690124	1.010763701	1.069516844	1.037671551	0.899163608	0.892233015	0.930186727	1.013907882	1.15452279	0.731222703	1.000362996	1.118885565
Q8VDM4	1.003573396	0.958581436	0.993687003	0.985294051	1.050230453	1.041295866	1.027005491	0.996779285	0.973259457	1.040301607	0.988976188	0.987506058
Q80XI3	0.958184315	0.967170032	0.959090438	0.972729772	0.994452309	0.974351253	1.041833832	1.000564995	1.001637405	1.078142317	1.038489224	1.053767219
Q4KUS2	0.991378932	0.978296652	0.966696457	1.016588015	0.961497831	0.97653839	1.011661466	0.982403833	1.016662091	1.007318006	1.042096255	1.068135408
Q64010	0.9819399	0.978788316	0.988868429	0.989032764	1.017971647	1.053592743	1.017057299	0.997393835	1.001123292	1.028295511	0.98381043	0.998741522
Q00898	1.142473037	1.198079009	1.254810238	0.62601092	0.513339976	0.481444106	1.65563925	1.444596067	1.598316361	0.655238166	0.583785384	0.52347145
Q9WTX6	0.988695435	1.049726101	0.987438321	0.989849069	0.97839499	0.991610309	1.005641632	0.9961142	0.971906233	0.994406298	1.025018808	1.041828789
Q7TMK9	0.920790632	0.911819236	0.921808948	0.930432541	0.97326158	0.972623673	1.078325827	0.995064559	1.021842482	1.185912403	1.065525505	1.089946133
Q8R0S4	0.929498975	0.961929831	0.958092044	1.023048126	0.98069301	0.964756609	0.993893655	1.02884593	1.015707198	0.980129703	1.064536122	1.095816717
P62983	1.032156554	0.999510484	1.033094594	1.060716186	1.025356861	1.069111862	1.006153502	0.905308643	0.979795456	0.906964108	0.993679042	0.994359947
Q6IRU5	0.927494594	0.935791541	0.992840299	0.979354705	0.956815263	1.001880026	1.063234601	1.009729662	1.055595045	1.0533555	1.000548677	1.041284479
Q61543	1.010841685	0.955820937	1.010782232	0.993035769	0.905958521	0.964348281	1.027777951	1.014294988	1.087737455	0.922392776	0.994336589	1.084965383
P62881	0.951255166	0.972613182	0.971503551	1.029877481	1.013777995	0.990079796	1.025013448	0.987796338	0.995777533	1.033383053	1.026368049	1.034143808
Q9EPW0	0.981855171	1.006992533	1.004245009	1.020982722	0.968765215	0.963788983	1.00015848	1.00566408	0.981869912	0.971091159	1.041787988	1.058005155
Q8BLK3	0.87775037	0.891205628	0.995133159	0.999398252	0.984165832	1.044629718	1.01239613	0.993991622	0.988931479	0.99836564	1.094682414	1.113353917
P53994	0.984747829	0.958199972	1.00298175	0.971459864	0.986606697	1.09103842	0.959153749	0.9676979	1.013196294	1.040215895	1.00055375	1.068496077
Q8VHJ5	0.910425756	0.966239621	0.99819173	1.035918648	1.043819862	0.977981087	0.983777095	0.993001816	0.995259321	0.956635355	1.072957595	1.055855575
Q9DCD0	1.073078792	0.998344157	1.05697628	1.002857969	1.026323222	0.987163234	0.984523737	0.99888469	1.009378334	0.910516449	0.979797368	0.967891609
Q60668	0.984591616	0.962367936	1.025694863	1.017021474	1.014890371	0.966350073	0.998768857	0.990577424	1.003024181	1.173864934	0.959479902	1.003552058
P13020	1.114884902	1.060629027	1.046468434	0.942230562	0.982107892	0.961468436	1.036580355	1.034519925	1.062678165	0.942412954	0.920499719	0.895571196

Q91VM5	0.964646005	0.964627777	0.989620797	1.00666499	1.006778506	1.017520366	1.029725033	1.005338674	1.00678896	1.044271696	1.001498466	0.996884759
Q9CPV4	1.013731729	0.999721685	1.044906558	1.039289637	1.04964241	1.050351883	0.962911775	0.986414837	0.979269513	0.955051759	0.969448313	0.972761356
P29341	1.039546318	0.981337619	1.009108375	0.971904538	0.933523049	0.981957482	1.063609782	1.041616753	1.087378245	0.910636912	0.952683886	0.997216935
P84096	1.052445552	1.043394998	1.030389524	1.086864843	1.096126366	1.008344432	0.879183137	0.945161469	0.996041597	0.849439033	1.008229367	0.999398401
P07758	1.144530693	1.219668872	1.24114759	0.757873367	0.678849083	0.689844496	1.335437506	1.260710188	1.322458018	0.767937566	0.700209942	0.706918286
O09061	0.956224399	0.990447765	1.003466962	0.977757033	0.987658559	0.997910599	1.010444665	1.01395276	1.051617104	1.002668607	0.990848971	1.025988311
P37040	1.026391992	0.974488138	1.021968842	1.010049845	1.036855808	1.051951949	0.997844144	0.973346426	0.984539883	0.960694188	0.982620417	1.000323941
Q78ZA7	1.010301456	0.976521519	1.002412791	0.996375148	1.047152315	1.020654803	0.972845546	1.005918987	1.022659106	0.959572433	0.996795056	0.998904976
Q64436	1.158115481	0.909455906	0.984519424	0.996353817	0.766912849	1.110465239	1.074633076	1.010957608	1.060995111	0.879060055	0.977777418	1.044198993
O54988	1.025872834	0.983894879	0.986153508	1.02150028	1.028773768	1.069665097	0.976799845	0.954025005	0.967053022	1.094302194	0.979262505	0.999862199
Q9WVE8	0.994242855	0.95838063	0.998810357	1.022497262	0.942533334	0.980321921	0.966279972	0.986127735	1.055760508	1.045792144	1.013507187	1.071844515
P61226	0.954103192	0.992374804	1.016972586	1.011554088	1.014691523	1.003837215	1.001818837	1.046677016	0.970952394	1.046441352	0.994916673	0.984039426
E9PVA8	1.012521128	0.9856476	0.986166759	0.961345413	1.001843169	1.006247357	1.011512373	1.022682032	1.016998174	1.100641843	0.980146818	0.976378399
Q91VI7	1.005678728	1.01383697	1.025647651	1.060276246	1.016101929	1.022457785	0.941848285	0.975371904	0.981398105	0.939398661	1.001034559	1.03311453
P70441	0.960686407	0.920711363	0.951125133	0.983350273	0.971310617	0.98597468	1.085677543	1.030720681	1.045613458	1.036091464	0.996440973	1.049938335
Q01097	0.854772203	0.927773618	0.913885763	0.975976977	0.9333923	0.923139173	1.063430354	1.039171677	1.003229911	1.095697014	1.136265062	1.146038758
Q99LD4	0.997114463	1.016982974	1.021763027	1.046985219	0.970184855	0.95777652	0.924106092	0.947406007	1.015991605	0.88585759	1.092120287	1.09566687
P23506	1.001485446	1.045653528	1.037344134	1.067272039	1.003659183	1.020851561	1.007704124	0.997441294	0.978593719	0.879068287	0.977776773	0.967149228
P21107	1.003987107	1.13792708	1.021723607	1.055929421	0.808914926	0.994964584	0.956576913	1.029000732	1.012157838	0.74142911	1.04172836	1.105334999
P22892	0.963236313	0.960814982	0.997910951	1.018436378	0.984040083	0.967354061	0.990085383	1.000336674	0.987336405	1.029790191	1.056218706	1.070255961
Q9R1P0	1.011713337	0.974709492	0.994062971	0.986733422	0.949283046	1.005600265	1.023586129	0.993068864	1.046717496	0.949442079	1.007893694	1.049706675
Q8JZW4	0.945450519	0.96998993	0.983759849	1.091053	1.031122011	1.032643171	0.958584599	0.986700803	0.967813078	0.978952972	1.018670346	1.063753246

Q5H8C4	0.996473987	0.952670558	0.971981339	0.989544549	0.979633729	1.012159492	1.001610855	1.009054375	1.004097473	1.028003063	1.040100792	1.042208878
P43277	1.151738997	1.022151509	1.074991726	1.107010059	0.950090813	1.04012446	1.019336498	0.996467006	1.026305507	0.812715896	0.86236888	0.922115137
Q99JB2	1.086595926	1.045528079	0.992347	0.885656298	0.991609088	0.980608546	1.022052788	0.997425972	1.09135866	1.108127633	0.9307901	0.93247985
F8VPU2	1.002416876	0.997565506	0.974953996	0.977107672	0.985627599	1.018726971	0.99526863	0.964410988	1.009009514	1.112916147	1.008518734	1.02529791
Q8K0S0	0.96311342	0.974621642	0.98879818	1.035989753	1.010832291	0.974532385	0.995144049	0.961133021	1.014218929	0.940330653	1.053012691	1.080680524
Q9CXS4	0.948799476	0.92809206	0.983851947	0.997596001	1.029851361	0.986684247	1.014060845	0.951158802	1.008515509	1.180814995	1.006874115	1.058594915
O54829	0.936653636	0.941367656	0.960950998	0.947272266	0.934150642	0.944327615	1.094564949	1.040944627	1.065325341	1.053336527	1.05972631	1.018457194
P56695	0.950006935	0.995483718	1.019059262	1.110357047	0.939037583	1.090546341	0.881235159	1.239186861	0.967610117	0.939448324	0.940802726	0.934232805
P07356	1.205477598	1.031150441	1.040071464	0.927384476	0.953174821	1.010265947	1.020930276	1.009319028	1.020085328	0.96115574	0.937652656	0.90891368
Q00623	1.104462825	1.386153843	1.12156798	0.729994751	0.672660352	0.606003233	1.456887808	1.39621922	1.294309617	0.722334577	0.679476158	0.620795416
Q64331	1.025152392	1.008156502	0.998915038	1.032069032	1.106479674	1.077215941	0.926801073	0.938780002	0.934863389	1.020822611	1.009454662	0.987570276
Q9D8W5	0.987412514	0.981848834	0.988538923	0.964650485	1.115981103	1.014712414	1.00244927	1.009337519	0.974739314	1.00066409	1.038276203	0.941299043
Q91YM2	0.969123127	0.966024328	0.982864465	1.02263879	0.93784244	1.02776655	1.003245548	0.961493863	1.004050979	1.068426277	1.017411615	1.089870131
P22599	1.259277138	1.332217497	1.291961254	0.681314314	0.668861772	0.640732007	1.348992876	1.276640786	1.33209576	0.729483344	0.647329207	0.606721572
P05132	0.957890638	0.933581869	0.972876412	0.962524635	1.062874881	1.045839077	1.007180524	1.011548322	0.969192975	1.031502945	1.054688445	1.017730396
P48774	1.055246166	1.041578007	1.072993109	1.103112048	1.10718291	1.09276754	0.93426066	0.932056581	0.933967402	0.845619457	0.948456049	0.941306457
Q9JJV2	0.990634502	0.992057024	1.022742482	1.019481299	1.055931799	1.017249215	0.99416286	1.012096198	0.970892358	0.981466134	0.989133179	0.977487489
O88447	1.013477831	1.016193308	1.012173551	0.996019246	1.000316111	0.983230657	1.012963348	1.026726254	1.003630513	1.006301138	0.974607429	0.981100545
Q80UG5	0.986527414	1.020805781	1.004494977	1.051293588	1.016732607	1.061101782	0.988343299	0.96711452	0.960595934	0.960583406	0.994976361	1.01144749
P28474	1.022756345	1.000702287	1.014543953	1.08368129	1.06217474	0.992021057	0.907759191	0.948973202	0.969016467	0.935776491	1.032283793	1.053702365
E9Q6P5	1.030743824	1.017813944	0.969619863	0.983837934	0.984444956	0.979424053	1.003371167	0.979642794	1.039564476	1.04355009	0.997626132	1.012253239
Q80UG2	0.95398896	0.983114416	0.976186263	1.000168873	0.976332283	0.983411357	1.01693707	1.005628136	1.018420811	1.040543799	1.040837667	1.027455915

P70206	0.98807399	0.956365677	0.972458195	1.024763553	1.020569453	1.013720843	0.990172155	0.972270962	0.978962579	1.024479313	1.044981813	1.050608882
P20918	1.218871681	1.264590336	1.241735805	0.814903584	0.775957497	0.76031154	1.164249814	1.124954792	1.208085429	0.790433513	0.758420977	0.771562546
O35685	0.951368121	0.964765109	1.002369609	0.987548903	1.045670532	0.995136955	1.037395656	1.011045336	0.981903409	1.040408062	1.016994726	0.994675577
Q62421	1.013488534	1.061839853	1.122397504	1.041083893	1.090229516	1.090445399	0.933031456	0.969417787	0.899290621	0.97979665	0.927079742	0.926320177
Q9CQN1	0.952399295	0.939850702	0.930570623	0.956372847	0.914708687	0.982095342	1.079022501	1.059540545	1.063888169	1.051697246	1.021348831	1.059528462
P62806	0.941541155	1.249389688	1.106235129	1.263884731	0.960504711	1.20410753	0.926075067	1.033382065	0.872429636	0.466784924	0.920930326	0.908844646
Q99KK2	1.016729219	0.989581766	1.043786447	1.029326748	1.026493127	0.956712067	0.947872169	0.995059119	0.962089812	0.952764596	1.040798022	1.049950756
O08547	1.052761813	1.01647524	0.997435831	1.000186205	0.980805156	1.045605861	1.032512389	1.003496852	0.989978133	0.952902102	0.957570511	0.987849498
Q9CY58	1.007116983	0.965164996	0.992972095	0.99245237	1.022600735	1.028612078	1.02842416	0.989773024	1.013420382	1.018177859	0.985941913	0.986973106
Q99LI8	0.983067476	0.989354443	0.996508565	1.029543383	0.957990731	1.025186017	1.015385974	0.965474406	1.055685857	0.953157641	0.961963054	1.072890202
Q61102	1.093408324	1.16532988	1.018614834	1.020720061	1.036615609	1.008427778	0.939078557	0.95713452	0.915931762	0.988846908	0.977145483	0.939373638
O54865	0.96310718	0.946278505	1.003532539	1.017471798	1.022584641	1.034860376	1.00635564	1.00997692	0.971937198	1.05494406	1.012068499	1.001814784
Q6PCP5	0.998771822	0.983096095	0.9826127	0.981695744	0.980203188	0.969298023	1.041049836	1.029666139	1.00660871	1.021296174	1.040806113	0.979052868
O70589	0.934694701	0.950375075	0.965602918	1.011599498	0.974569497	1.00073032	1.014954592	0.996796519	0.982946145	1.09534249	1.060653525	1.060453594
O70172	0.9995754	1.003451832	0.998546552	1.052979045	1.092394621	1.047378558	0.974082846	0.978942858	0.942917591	0.987677524	0.976044809	0.992796824
Q6PGN3	1.037989276	1.037203803	1.030938357	1.015615571	0.995328689	0.97786133	0.955076388	0.987369759	0.988278443	0.906324206	1.017272116	1.045289128
Q9WTT4	0.904820363	1.160705887	1.041253557	1.164449894	0.907440119	0.995532062	0.881758883	0.980366299	1.005887584	0.76071005	1.044973522	1.080771131
Q9CZD3	0.99448715	0.970461894	1.001031132	1.013303961	1.032634939	0.997850984	0.980020031	1.016108419	0.98591021	1.042107318	1.015782435	0.992693466
Q9DCW4	1.094537474	1.008817733	1.018816312	1.000566785	1.021267252	1.031320926	0.946847969	0.991222243	0.971343944	0.993538815	0.983464197	0.985374913
Q8CI71	0.957358249	0.951729908	0.979005882	0.997780613	0.96823096	1.028422291	1.037477971	0.984801854	0.986869615	1.033462649	1.053518606	1.044463661
Q9D6J6	1.024652786	1.025616523	1.03027582	1.031807757	0.965365389	1.035011916	0.991935707	0.952505147	1.022912091	0.882658811	0.995084335	1.023602577
Q9Z0S1	1.015385	0.954905391	1.010666676	1.046992915	0.996950789	1.07363273	0.995078429	0.948997257	0.9847903	0.983331213	0.989438024	1.03330749

Q9ER72	0.966539037	0.969341608	0.991045568	0.990875378	0.990448094	1.002052921	1.034250079	0.992973491	1.013743246	1.026084615	1.01529174	1.029911235
Q62417	1.003113648	0.988734458	0.995587908	1.014115706	1.013155969	1.018805524	1.003802567	0.981456048	0.982259312	1.024417857	1.004190833	1.010686141
Q811P8	0.933355919	0.969017754	0.945515535	1.00952823	0.983324998	0.987951597	1.054687696	1.000260418	0.999448289	1.073142624	1.042125636	1.038666959
Q8CAA7	0.931429314	0.976905452	0.94294106	0.987470922	0.970795507	0.946803182	1.025549917	0.981636026	1.025507667	1.030828902	1.080640775	1.111468616
Q3ULJ0	0.96891388	0.991390306	0.993077841	1.023854591	0.987967956	1.003390629	0.98958262	1.011524365	1.029784122	0.972041572	1.00358794	1.031343767
Q9JIF0	1.012844133	0.992783344	1.017289965	0.994935794	0.966563006	0.911997335	0.980700625	1.022941171	0.984008269	1.051005254	1.039841918	1.062962787
Q9D1G1	0.986495201	0.991183603	1.012820947	1.015438497	0.990585437	1.035542241	1.014082852	1.016618328	0.99892912	0.916697645	0.982780057	1.029135911
P46638	1.007725278	0.988075259	0.994029339	0.978130806	1.016763755	1.031147581	1.013224048	1.001210283	1.014497234	0.989688524	0.991642613	0.992306728
P55937	1.018190464	1.032991172	0.996929153	0.956486909	1.006863955	0.98134116	1.007901243	1.020612652	0.991592082	1.081536945	0.973399905	0.992424113
Q3USB7	1.082499831	1.051222319	1.030620819	1.074712633	1.084147963	1.055517647	0.925520611	0.944012388	0.967940305	0.871711743	0.953034716	0.976104202
P23116	1.008375015	0.97073245	0.991651901	1.005161211	1.005345473	1.03036476	1.015483639	0.986151584	0.993145995	1.025934275	0.992298733	1.01540703
Q80U28	0.960497804	0.981417275	0.967031167	0.997117218	0.954444439	0.986119569	1.025632586	0.987265667	0.996264275	1.074204282	1.030565197	1.084685256
Q8VCM7	1.249297946	1.385398707	1.178147957	0.718555585	0.663273556	0.63712977	1.150086651	1.273582197	1.385994498	0.897341109	0.677302647	0.690375174
Q8BW75	1.068004397	0.995097066	1.032877472	1.077216992	1.059363069	1.100345819	0.955577257	0.934001524	0.92276935	0.981229815	0.955300051	0.982102605
Q91VM9	1.041971935	0.993178802	1.012195806	1.036441165	1.023068936	1.043592038	0.971538183	0.962983793	1.028263425	0.942127306	0.966944216	0.995098234
E9Q7G0	1.027700543	1.010528801	0.979810295	0.99791248	1.002808451	1.130192651	0.961297889	0.948815538	1.030728268	0.996417199	0.97629287	0.975829107
Q9EPU0	0.99867037	0.978962694	0.989410454	1.0296808	1.004245383	0.985076119	0.989858554	1.005066298	0.993303086	1.015535804	1.013890474	1.029453905
Q9JIA1	0.971454332	0.992272307	0.994727259	1.062152135	1.003809546	0.946281453	0.956380928	0.979255201	1.044794043	0.869038666	1.059915729	1.081497622
Q8R0A7	0.930657126	0.954250232	0.999270944	0.994257967	1.01249418	0.983099087	1.035157213	1.04177263	0.998472463	1.03194545	1.018822563	1.017397945
P35564	1.063068793	0.986949215	1.008764085	0.966314078	1.032001057	1.015560308	1.014014849	0.984942539	0.98224071	1.04889407	0.976396172	0.977313965
P30416	0.994473813	1.136565525	0.971750179	0.979845701	1.008930141	0.982412703	0.983281884	0.977302463	0.979769568	0.983622153	1.029561713	0.992260267
Q9D2U9	0.936455167	0.907711347	0.956158147	0.918274619	0.830241875	0.915047414	1.206096817	1.108067765	1.156061056	0.841389497	1.023312117	1.080971472

Q9WVJ2	0.949026291	0.955400995	1.007132528	0.99247014	1.001332099	0.961920211	1.005354527	1.006052817	0.984599597	1.053742917	1.050581903	1.063092958
Q9CR95	0.956122541	0.952885803	0.994174488	1.014385673	1.068630701	1.032390801	0.983587644	0.992825203	0.938659644	1.202991062	1.004024403	0.976504455
Q58A65	1.004914397	0.985263641	0.98039961	0.99538138	1.015990312	1.019458348	1.006949827	0.983054483	1.013171004	1.044646672	0.992878428	1.003218775
Q8BHN3	0.993703663	1.02616173	0.983764394	0.978550096	1.000671527	0.99795177	0.989987776	0.996506403	1.001348366	1.030921973	1.031221905	1.000102571
Q9DCX2	1.060473032	1.043137941	1.035689949	1.044392806	1.006144063	1.003144396	0.996155551	0.97021492	1.003193344	0.849957032	0.991431153	0.970537062
P23927	1.190552655	1.167455637	1.129102545	1.087303449	1.169733678	1.053332548	0.856565331	0.89589303	0.904696645	0.920479624	0.870750288	0.834760911
Q8C0C7	1.061854094	1.038567954	1.00591309	0.975989544	0.978634525	1.017722801	1.018401605	0.994184388	1.027395758	0.943360148	0.960891477	0.984242192
Q8K3J1	1.056814667	1.026907028	1.020958356	1.029580135	1.039577348	1.031594732	0.96890718	1.007876583	0.980829371	1.028146838	0.925476072	0.947823394
Q60575	0.979004213	0.966922429	1.059491249	0.981946118	1.116373705	0.993932991	1.001069101	0.964807131	0.976633858	1.020862388	1.039595312	0.923658579
O88990	0.878865156	0.963594528	0.930675583	1.109593358	1.025394269	1.092985995	0.952809191	0.905301545	0.939663824	1.012117846	1.044700404	1.19664609
P70663	1.115122102	1.06978218	1.081716189	0.912757982	0.971438331	0.920356758	1.028608225	1.03691217	1.099087076	1.006532018	0.882149323	0.897370106
P43274	1.290832623	1.12756965	1.138798592	1.138337799	1.001101589	1.179625905	0.910546287	0.842755028	0.94413912	0.842219934	0.784597105	0.868948454
Q9WV02	0.951080785	0.97859878	0.987643459	1.073457574	1.001704089	0.969007119	0.952192139	1.07400685	0.986361139	1.024384033	1.021430639	1.011528188
Q5SSL4	0.936241057	0.988378903	0.994750443	1.033424914	1.152432739	0.995843618	0.914752	0.989672181	0.908760869	1.000054045	1.118363201	0.994667566
P24529	0.903589576	0.98421139	0.987610335	1.169171704	1.167081169	1.026291986	0.888794894	0.975576669	0.925187272	0.942902504	1.029639524	1.032763408
P06801	1.036197598	1.024909928	1.016257712	0.994571865	1.037003534	0.992093435	0.979113381	1.005285021	0.960734235	1.041250106	1.008138601	0.954659462
B9EJA2	0.924335674	1.007408482	0.993701184	1.049778166	0.974640086	0.971168214	0.967972832	1.000715885	1.004563649	0.991283409	1.04549612	1.079215917
Q5U3K5	0.924572277	1.001859047	1.022650569	0.999766987	0.987639777	0.969284464	1.005454995	1.023610042	1.001243811	1.05659232	1.006909184	1.031820898
Q60931	0.936965057	0.932832797	0.944859907	1.035638092	1.03837022	1.07925981	1.026548475	0.929208122	0.983625574	0.939008581	1.052145517	1.10237641
Q8VIJ6	0.999938146	0.943992558	1.00526278	1.026688242	0.957372594	0.993160894	1.065963918	1.003070298	1.045746356	1.040338368	0.924686457	1.034390092
P63168	0.964663442	1.04350927	1.004534195	1.02611727	1.001212692	0.98197464	0.979616445	0.98219431	0.976795267	0.909291397	1.049593793	1.066041768
P62334	1.015525562	0.988266823	0.982849885	0.986235068	0.931523909	1.002452946	1.025818404	0.999739051	1.059867126	0.970490549	0.971122725	1.072967767

Q8VDP4	1.008138152	0.98021008	1.000710612	1.022581317	0.984058191	1.019926447	0.983273805	0.996646555	0.997758551	0.953765924	1.018385991	1.042244596
Q9DCL9	1.015099488	1.012562307	1.031668739	1.046363596	1.018484846	1.023128017	0.957980042	0.984335191	0.973952524	0.97938438	0.982011712	1.008748932
O35927	0.928872215	0.951618186	1.020362824	1.056566659	0.972618571	0.990964706	1.019108859	0.988913403	1.023019166	0.934393452	1.031198282	1.062679948
P15864	1.150229483	1.096959727	1.097842305	1.204997122	0.93821137	1.089521821	0.963222332	1.017364544	0.949404764	0.698426677	0.887010567	0.862344251
Q148V7	0.928004789	0.924851781	0.960445869	0.979902097	0.982766191	1.004937852	1.037935044	1.008930685	1.010950222	1.08968081	1.051602649	1.05739447
Q8VCT3	0.947299257	0.96922489	1.000958133	1.065147737	0.988682552	0.969781605	0.969466763	0.99280315	1.016551994	0.899721502	1.061936352	1.090727006
Q5EBJ4	1.1433914	1.121673773	1.072558961	1.087768501	1.246310003	1.167008447	0.844005717	0.877944949	0.89508214	0.966082837	0.85968031	0.829056658
P19157	1.099635547	1.062004735	1.06683363	1.091565441	1.046205066	1.162783866	0.934265484	0.914936108	0.963836344	0.861876101	0.87217997	0.955315704
Q9R1V6	0.995912304	1.044264196	0.990571774	1.083095489	0.939940688	0.988229982	0.990514139	0.967381171	0.962211207	0.915411583	1.027721296	1.09617409
P12658	0.922387864	0.988543706	1.022707377	1.022349727	0.939816967	0.948596037	0.995484751	1.045798941	1.061170376	0.877400704	1.034346848	1.084393217
P24527	1.006044183	0.981660781	1.018130085	1.007204392	1.019816922	1.032778671	1.017620244	0.987472909	0.984973153	1.014503074	0.969150988	0.999322744
Q99JY0	1.105225662	1.031150403	1.017968062	0.986318049	1.08314256	1.046910309	0.956011446	0.966261724	0.96727528	1.054728192	0.952069794	0.914222121
Q9CQW1	0.96237229	0.97771723	0.95297385	0.961314465	0.999627574	1.029207	1.039746848	0.998054712	1.029018808	1.079445782	0.987913337	1.030516947
Q8K3H0	0.950622509	0.957046682	0.980879258	1.00422197	1.028306779	1.026926355	1.006468226	0.984546988	0.994321535	1.044528152	1.018500691	1.04253437
Q6PAJ1	0.956106229	0.961271403	0.996382628	1.065916421	1.022789735	1.057877706	0.966522716	0.988499496	0.959382101	0.935448417	1.017070109	1.083067986
P14685	0.97824302	0.980438678	1.039794133	1.001073649	1.042112564	1.001530474	0.986995103	1.015876218	0.95969194	1.109643582	0.996804895	0.957947598
Q9QYJ0	0.96646614	0.985914939	0.998379126	1.012383816	0.999684379	0.978319289	1.003699198	1.038802638	0.978570293	1.005294748	1.043608783	1.002691885
Q9R1T2	0.978054504	0.97721319	1.016852046	0.99662679	1.01502394	0.986563816	0.995865042	1.012287146	0.987755881	1.033097168	1.020099307	1.011576137
Q61481	0.939318497	1.008838481	0.990566329	1.019080715	1.041662563	0.977099339	1.000548856	0.982704427	0.953806674	1.066505232	1.049528886	1.017447357
P20917	1.065347058	0.99319182	0.975087723	1.069110018	1.119910698	1.02228616	0.90327982	0.965076471	0.94670467	1.019369505	1.03218367	0.956363356
P63040	0.961693689	1.092851814	0.997396902	1.048532382	0.946770868	0.985314213	0.982097505	0.991349224	1.055842101	0.82527361	1.026231489	1.026285987
O88343	1.180077624	0.99490855	1.013970213	0.965597884	0.96279292	1.00262926	0.98541701	0.97793148	1.008607863	0.96078989	0.974737805	1.004355097

Q64105	1.05940459	1.027827783	1.040634108	1.016829458	1.043617871	1.072673566	0.983068728	0.971224669	0.978243943	0.937352654	0.941712744	0.952445816
Q08331	1.08989154	0.889623318	1.324355782	0.990819225	0.919800871	1.195876726	0.861685787	1.098740993	0.93216503	0.865964632	0.880847074	0.93991684
Q61990	0.990905737	0.989017849	0.989103232	1.026300656	1.046735442	0.97513706	1.020204353	1.03980184	0.935356712	1.123852386	0.9854206	0.962527286
P09528	1.293410241	1.162674723	1.215902265	1.229019237	1.193733689	1.256783211	0.749228182	0.825613785	0.767222652	0.775897254	0.844978412	0.771787372
Q64133	1.051859726	0.976869933	1.026879487	1.022147097	1.014688429	1.042695436	0.982503327	0.998782593	0.952407537	1.003067837	0.983127025	0.990074913
Q3TDD9	1.001880543	0.981178478	0.989779891	1.023641855	0.998785794	1.0342274	1.005192633	1.007605771	0.992731151	0.977590719	0.99570194	1.011313303
Q9Z2D6	1.063068685	1.027731548	1.083044943	1.103008243	1.075220561	1.081480666	0.931467178	0.96804307	0.952793448	0.919312569	0.896560292	0.939503307
Q9JHQ5	0.927560724	1.027289644	0.987137808	1.012012256	1.03512894	1.043293608	0.9925345	1.034398575	0.957406885	0.964006518	1.029191346	0.994183973
Q9D0M5	1.025794006	0.976297019	0.983507636	1.029365098	0.859318351	1.061892956	1.059839773	0.929240946	1.230944457	0.721616097	0.904513652	1.114824636
Q921F2	0.988819532	0.943989793	0.993214688	0.982733777	1.076675627	0.967741812	1.00307952	1.00030248	1.014967748	1.07916107	1.016222894	0.982962207
Q9Z140	1.003230923	0.977748473	1.071272667	1.005269344	0.949806421	1.03669911	1.026032897	1.056445135	0.938169881	1.051750481	0.947938717	0.98564492
Q8BMK4	1.062528034	1.053565769	0.976986997	0.913749463	0.884881041	0.976483546	1.074303449	1.039347655	1.060915152	0.948639173	0.995733437	0.993023943
Q3TXX4	1.00311063	0.980358202	0.995817107	1.072281882	1.03699946	1.058528856	1.013447054	1.018756981	0.958563619	0.952664043	0.983417241	0.945127316
Q3B7Z2	1.011907245	1.009674651	1.013471587	1.037746566	1.002892709	1.009229558	1.001044211	0.983188865	0.995814573	0.938110949	0.968305661	1.040594891
Q8C522	1.083642362	1.011930785	1.021497582	1.001748603	1.050617183	1.006863591	0.98393396	0.971931498	1.009946715	0.936558934	0.979868461	0.95572084
Q9CR16	0.985510606	1.020504221	0.993034512	0.980944347	0.971348016	0.981384528	1.039125812	1.02719123	1.019605728	1.019280624	0.99154467	0.989808842
Q9CPW0	1.062963633	1.045971235	1.018875255	1.066904882	1.051141769	1.059265485	0.9326012	0.925132974	0.917268531	1.014485791	0.992653842	0.98610835
P21279	1.000076916	0.999522465	1.024001772	1.037838243	1.032010533	0.999207447	0.984988695	1.001779377	0.991289825	0.932791981	1.004126628	0.993098439
P99026	1.034173005	1.016255353	1.041199699	0.981838267	0.997802931	1.044796744	1.055756624	1.033647422	1.029336048	1.019983005	0.876369808	0.908813082
P14869	0.975787892	0.959555365	1.013181925	0.979220412	1.083452894	0.987027486	1.03064917	1.004422759	0.970043124	1.088260209	0.996197	0.970570109
O88544	1.016401382	1.002987021	1.005242901	1.016676506	0.975504879	1.019172649	0.995674435	0.986221356	1.012551977	0.916846966	1.00933208	1.03465019
Q9Z2U0	0.969481217	0.964308741	1.00521085	0.981075604	1.013704129	1.041848969	1.023635235	1.013767643	0.987633401	1.027363971	1.001168277	0.999488061

Q64516	0.995446096	0.967188496	0.980554944	0.982077491	1.036847037	0.96048791	0.984846876	0.963872452	0.978883075	1.089446264	1.0570505	1.062988853
Q99JI4	0.960015391	0.997245362	1.019535779	1.030850627	1.024110009	0.964620007	0.936469429	0.990051538	0.971163496	0.921342926	1.095884471	1.072630178
Q61137	0.914590384	0.942376302	0.959938047	0.980951785	0.979322861	0.982025329	1.042878224	1.045791377	1.009107976	1.050113742	1.05569207	1.052088614
Q8CGU1	0.940109733	0.956646594	0.950591653	1.001706401	0.971325083	1.026259213	1.030594332	1.005098935	1.01348316	0.95946488	1.063999863	1.06799463
P61089	0.970929567	0.971162648	1.038419882	1.016018117	0.964623633	1.035670613	1.06033189	1.007387095	1.031663369	0.843759127	0.999901681	1.00134179
Q8BJY1	1.031026652	0.952028393	1.00892112	0.971682777	0.976969213	1.037331891	1.039334458	1.009847634	1.003602465	1.086468031	0.955978963	0.988840716
Q8VCW8	1.075726383	1.008838966	1.071818433	1.062064654	1.056553465	1.097925826	0.952982011	0.945652732	0.950502952	0.899709654	0.950345067	0.949465974
Q8BG05	0.988791013	0.944854859	0.988052547	0.986448088	1.043020995	0.986917235	1.039952736	0.988283288	1.047834891	1.10305233	0.951697395	0.995165177
Q8C1A5	0.905284948	0.975945724	0.959825572	0.997356433	1.063952358	1.032070592	0.991196636	0.993954667	0.959707644	1.032867174	1.077217611	1.037131215
P09671	1.041636313	0.965121596	1.004248487	1.027542672	1.162158472	1.033316756	0.967736014	0.947619983	0.905284232	1.106961588	0.998804922	0.943454894
Q9Z2H2	0.931155022	0.955545844	0.931996786	0.970449625	0.997970467	0.969186743	1.072511894	0.997690184	1.014976569	1.051848465	1.071215373	1.052238436
P35278	1.06419583	1.026845538	1.031360036	0.940095156	0.986273094	0.848713056	0.984238207	0.987303865	0.952835847	1.258424913	1.068036118	0.97383812
Q91WJ8	1.01858233	0.96856755	1.000470276	1.00895358	0.968723565	1.015600527	1.021679128	0.972534185	1.00850086	1.010382474	0.99747834	1.038752408
Q641P0	0.926613626	0.961424175	0.952675916	0.982532451	0.989506873	0.954466345	1.066950697	1.020167632	1.01741379	1.075274835	1.046630178	1.033091881
Q91WS0	1.013007418	1.027900495	1.047905412	1.096136633	0.992593849	1.051037533	0.970736031	0.963695163	0.972054293	0.940128228	0.96278902	0.985214359
Q9DBR7	0.937560734	0.975223032	0.977496673	1.004207459	1.053812248	1.027218231	1.011603633	0.9981934	0.942278055	1.058790813	1.02501888	1.038552739
Q60605	1.056475906	0.992920169	1.030639635	1.013556763	0.971257495	1.059386345	1.022367152	0.943789843	1.028112946	0.854598015	0.97142646	1.029834186
Q8BIZ1	0.87328877	0.979280276	0.983330745	1.03656728	1.003470719	0.934728409	0.986236218	0.992755477	1.028207324	0.923380048	1.095525967	1.126166674
P45591	1.04326972	0.990402586	1.011041651	0.981788466	1.041776857	1.064295402	0.995337535	0.989364206	0.986600595	1.018728845	0.950042233	0.977836813
Q91WP6	1.409159602	1.557958633	1.31400881	0.731292725	0.736891891	0.683354542	1.12881954	1.155644619	1.177158364	0.705007115	0.686790978	0.597320271
Q8R1Q8	1.015114676	0.975238552	0.983292003	0.984071012	1.003971698	1.003016803	1.0216869	0.984623593	0.993485566	1.022749553	1.018492	1.026899456
A2A5R2	0.999822265	0.979043009	0.995216116	1.008297662	1.00415536	1.022697535	1.000637658	0.983856548	0.991941785	1.052758162	0.99927906	1.012564423

Q922R8	1.026521953	0.966759859	1.050818359	0.89547024	0.891868919	0.933689387	1.126578617	1.048249359	1.074659446	0.949119705	0.993599649	1.001033214
P18572	0.960654214	0.964304968	0.937342417	0.943988491	0.958115218	0.934041809	1.069106291	1.033432291	1.065030339	1.042038279	1.050326472	1.046185062
P52503	1.045097616	1.023562982	1.058846892	1.085224323	1.042680596	1.004725493	0.914502324	0.931116822	0.981324994	0.890738373	1.016151644	1.010109568
Q9D2G2	1.020337807	0.966742819	0.974939361	0.986915984	1.009217351	1.074302809	0.996218152	0.935578721	1.025339584	0.985334244	0.985044115	1.070634383
P84075	0.934208336	1.092687845	1.053565712	1.059752167	1.126526617	0.881051651	0.931275742	1.039947286	1.024260011	0.77373025	1.046786442	0.953075996
P62192	1.013767994	0.995592319	1.021289334	0.99414188	0.987817492	1.001430287	1.044719635	1.021497162	1.007237804	0.949369914	0.98341382	0.976465202
P62827	1.003251779	1.019616431	1.031972239	1.063812898	0.915662379	0.912048573	0.948782496	0.997874374	1.052299565	0.800017918	1.069330349	1.112187535
Q80XI4	0.921636083	0.943753431	0.962157245	0.9869883	1.034277685	1.020986064	1.007782514	1.012686486	0.985194575	1.073392224	1.067008964	1.020253826
Q8QZS1	1.055590563	1.003683712	1.043581824	1.048444766	1.022687081	1.081539246	0.955104794	0.955309031	0.962804259	0.91506747	0.965949616	1.01103957
O88448	1.031254054	1.031371715	1.003476498	1.000516555	1.002400632	0.979708002	0.987412839	1.002721977	1.009708674	0.932882786	1.011808663	1.004310943
P35762	1.146720143	1.083948651	1.057286758	1.15867947	1.121663646	1.143436805	0.825660293	0.866643075	0.951020675	0.87081224	0.903628264	0.931827838
Q505F5	1.006947116	0.989815733	1.013660396	0.997294675	1.043750827	0.991605504	0.965148841	0.98230843	1.0110641	0.991701789	0.997611308	1.036019184
Q8BU30	1.02326877	1.006752109	0.979497849	0.986724159	0.973897606	0.97216018	1.009409766	0.97466465	0.994610171	1.008694496	1.025216846	1.074025005
O88910	0.957429413	0.977116194	0.958090545	1.016632203	1.026102866	0.990434985	0.975135637	1.022339122	0.999820014	1.071149579	1.042976028	1.009124888
P70404	0.962484298	0.973686016	1.022379113	1.028187087	0.980389602	1.022798492	0.943135131	0.999065662	1.039362545	0.901028015	1.049815684	1.048100733
A2AWA9	0.974213522	0.98033957	1.013862739	1.029078283	0.974443247	0.984246228	0.993466485	1.027087889	1.01609815	0.956653817	1.023264918	1.021764296
Q6GQT9	0.995650249	0.979505487	0.96180033	0.990860821	1.014597988	0.996320519	0.986024647	0.979474174	1.025762872	1.035263634	1.025098004	1.046320354
P01872	1.003779122	1.815633529	1.325019268	0.814371555	0.811833141	0.817088434	1.019124189	0.979999924	1.056122479	0.866192911	0.730275857	0.715654844
Q9CZ42	0.948851443	0.952688239	0.979510547	1.00989596	1.064990606	1.033285704	0.979339988	1.003193152	0.978425439	1.035303514	1.028095847	1.025170098
Q8VE47	0.960025783	0.998310939	1.025125413	0.997633549	1.044173884	0.926336846	0.950653387	1.024587198	1.028222006	0.901272158	1.061246686	1.047394312
P63037	0.99723825	0.966789688	0.999569166	0.974201859	1.03836735	1.034443407	1.0156218	0.965385885	0.981775987	1.033730647	1.018963661	1.012373807
Q7TMF3	1.084498879	1.014037335	1.041546713	0.96630715	0.929740442	1.002439344	1.065420715	1.009484869	1.073391563	0.97260925	0.915962289	0.933034932

P61082	0.987904628	1.029731055	0.994412485	1.033187425	1.005575795	0.98315863	0.933112331	0.959749621	1.006237161	1.011206728	1.028397151	1.063949091
Q8BHZ0	0.969091869	0.934740531	0.929154804	0.966690037	0.991853377	1.001749468	1.014043347	0.993362621	1.012122443	1.126808065	1.039912616	1.087301682
Q9DB20	1.041082486	1.059186815	1.068873133	1.109905982	1.054851954	1.015093589	0.914361342	0.984180194	0.992818282	0.726650852	0.992525795	0.976502504
Q80YA9	0.909231774	0.967644321	0.991261661	0.991129313	0.944311737	0.978455425	1.069929403	1.03268144	1.066077014	0.985608057	1.025266406	1.016684068
Q9D2V7	1.004876342	1.004950736	1.015195786	0.946387125	0.936632159	0.915335016	1.049659333	1.080613791	1.084463305	0.934548521	0.983759835	1.008820795
Q8BGH2	0.962538036	0.962475338	1.026243643	1.044997082	1.087330013	0.955349192	0.954352876	0.982738657	0.951913753	1.008066564	1.07679247	1.013197454
Q6PB44	1.05108875	1.02888499	1.012774087	1.022690189	0.957704441	1.033273765	1.007269304	0.98617004	0.97537533	0.992613857	0.972121026	0.996824618
O35593	0.99482905	0.953370281	1.010304422	1.028566725	1.017084103	1.050218129	1.045305519	1.015990824	0.979282035	0.98551661	0.952697412	0.993212698
O88532	0.960210716	0.968885291	0.967914602	1.006359938	0.985502182	0.969720268	0.991483283	0.992467683	1.041364719	1.038295193	1.045600346	1.056510281
Q9R1V7	1.05669129	1.024617104	0.991712361	1.004230401	1.035225831	1.014809981	0.969668876	0.951155979	1.001404569	0.942878485	1.009209335	1.015253451
Q8JZS0	1.057006109	1.049783672	0.99303784	1.022522034	0.987219695	1.00983278	1.001418785	1.019606701	1.006164631	0.912019114	0.967726064	0.974031669
P11627	0.954862879	0.979546657	0.996215379	0.989986298	0.994524048	1.001764023	1.023379811	1.044493301	0.999286243	1.004112125	1.000072436	1.025658487
P28656	0.918577911	0.942463465	0.977841583	1.007199426	1.012679798	1.005416903	1.000768056	0.962710118	1.040545116	1.013269462	1.03655516	1.09661856
Q9CXW3	0.969293994	0.921117299	0.993482141	0.968674295	0.921516037	0.990400149	1.076651579	1.066928393	1.051473883	0.967122377	0.999113727	1.054311821
Q9D0L7	0.924638542	0.977635381	1.036819504	1.065301678	1.038855948	0.948542425	0.94054274	1.036595976	1.016534383	0.890478982	1.061132223	1.024779899
Q62167	1.027797519	0.965921103	0.979991558	0.959664256	1.081713044	0.95941414	1.041459742	1.020970943	0.932722848	1.169326003	1.007983585	0.951948084
Q6IR34	1.023306368	0.999688524	1.052519029	1.029780814	0.990773056	1.019736962	0.998997201	1.032013503	0.962760275	0.962531852	0.962857928	0.984508736
P12970	1.063755839	1.046668276	1.015379038	0.963567168	0.973066424	0.998573083	1.072225235	1.041206373	1.030906233	0.908499103	0.93535927	0.934461391
Q9D0L8	0.944139461	0.979200075	1.023844876	1.045618023	1.015397832	0.977541147	0.976777484	0.988665587	0.964587023	0.986502387	1.052881383	1.058649193
Q9Z1R2	1.017340107	1.024128367	1.024335491	1.035110947	1.006223408	0.979250658	0.985659079	1.030489973	0.993049451	0.944124753	0.989035012	0.976755588
Q9Z1L5	0.926897529	0.953878182	0.960998751	1.037208822	1.033998694	0.996801344	1.000347391	1.009159858	0.989102356	1.011507894	1.05320876	1.046055163
O35857	0.988131893	0.983121741	0.973005093	0.996475178	1.012223886	1.0304479	1.007219185	1.002466148	1.003027173	1.003745566	1.009744716	1.014796264

Q0GNC1	0.944184355	0.965688757	0.960311155	1.047999245	1.098157621	1.017567191	0.970226758	0.984579646	0.976829416	1.003284029	1.030402902	1.034545893
Q61771	1.004282647	0.992323785	1.006609016	0.992253795	0.983654902	1.016902056	1.020325094	1.013379835	1.019289652	1.004699375	0.976389741	0.992680434
P01864	2.199086127	1.968985304	1.414961315	0.886059144	0.670712425	0.713542582	0.705611911	0.913922768	0.740353999	0.68186172	0.605057263	0.63011051
Q8R366	1.053855158	0.980014612	1.048210359	1.114303499	1.011057242	1.101060223	0.895248817	0.919369565	0.931318059	0.974225614	0.987023897	1.045184967
Q8K021	0.942516138	0.965218969	0.987616479	1.056171113	1.010026031	1.037344323	0.986005636	0.989140198	0.975100219	1.031998428	1.020673572	1.03836301
A2ALU4	0.890499715	0.918908216	0.968079224	1.014574677	0.955200275	1.012947204	1.038456238	1.019093985	1.009185875	1.029257447	1.059954411	1.091764708
Q9DBC7	1.008031452	0.96887095	1.002263096	1.011671923	0.989333197	1.027990207	1.014699675	0.980321612	0.988444429	1.005735403	1.001378018	1.031890357
P97445	0.961355359	0.965856907	0.978403944	0.976110206	0.943798132	0.98027488	1.050328478	1.005504053	1.039109934	1.016637717	1.031579529	1.057243229
Q9QUP5	1.115266271	1.081132504	1.091227485	1.187368174	1.158485544	1.098143724	0.824804425	0.883389751	0.876368804	0.885099186	0.920489162	0.949237366
Q8R5M8	0.951248362	0.994833383	1.029473064	1.024008392	0.998723577	0.96242171	0.987957325	1.003220661	1.004247473	0.866562621	1.059301716	1.070895274
Q8CGK7	0.883023085	0.955491889	0.970377745	1.13881493	1.104038012	1.045941729	0.905298581	0.942068214	0.951699456	0.944279074	1.043554216	1.140296011
Q80Z24	0.842832477	0.905314296	0.95394766	0.984559344	0.974821011	0.928835606	1.033689844	1.066288497	1.017947215	1.055779315	1.110615497	1.122125723
P84086	0.862569708	1.178187965	0.977778722	1.115146519	0.784699794	0.891466467	0.935967124	1.045186903	1.10413467	0.653838813	1.103552198	1.186118443
Q8CC88	1.098731922	1.010944271	1.022902067	1.027708566	1.020149686	1.067826133	0.9478632	0.954749336	0.979186895	1.042630006	0.940447152	0.966660127
Q8JZN5	1.031345212	0.971813363	1.002845311	1.007584869	1.031299878	1.026115306	0.977642093	0.968350338	0.989916933	0.994713183	1.009311012	1.022545758
Q9Z2W0	0.971401449	0.978556671	0.999309723	1.015753613	1.018653062	0.992606026	0.991460391	1.026595832	0.998830679	0.989942172	1.010166799	1.02251705
Q61330	1.089210715	1.020904203	1.030490769	1.05591334	1.11731354	1.098805301	0.949847515	0.935257426	0.933707809	0.970671662	0.945182972	0.914772628
P51830	0.998755687	1.009708278	0.993629906	1.004864843	0.974127601	0.97499227	1.001701978	0.993539672	1.028127378	0.934680072	1.032135627	1.041144763
Q9WV60	0.965461896	0.961703434	0.989859428	1.011086632	0.978308271	0.994869589	1.026531472	1.011685142	1.036527468	0.955521916	1.005604435	1.055760676
Q9WVK4	1.040243861	1.019090398	1.046278068	0.985875042	0.950542047	1.018607058	1.017291753	1.038226851	1.008770088	0.947543946	0.959604411	0.969018815
Q99KE1	0.970316195	1.0025616	0.977476412	1.043029377	1.017207108	0.999534648	1.024040969	0.970465117	0.986443266	0.951319525	1.016630071	1.04801676
P62196	1.001579244	0.96301572	0.976071654	0.980454136	0.972532546	1.083569536	1.04156395	1.047342458	1.024338326	1.035678378	0.939116489	0.973069008

Q80TS3	0.928217109	0.950175008	0.981519495	0.983840899	0.965560953	1.001680623	1.013772739	1.014715635	1.021583504	1.019328725	1.060406097	1.064696556
Q61584	0.994058116	0.959470922	0.994737419	0.962580889	0.970181006	0.987949935	1.01235079	1.01774989	1.009389915	1.050378328	1.025119792	1.047668958
Q8BW96	0.986623043	1.017423655	0.980747257	1.064588279	1.059728254	1.045399942	0.941366219	0.989974525	0.886310485	1.083751716	1.023525154	1.009034167
Q9JHI5	1.109919001	1.013232302	1.043541702	1.03210334	1.014476947	1.092741018	0.958117557	0.969380234	0.974006511	0.911808025	0.943361117	0.961684368
Q9ES28	0.965874938	0.961035709	0.977198387	0.968051868	0.920484149	0.968577085	1.055982951	1.034088107	1.079520976	0.995770777	1.012072647	1.05183634
Q9QUM9	0.950633339	0.972481156	1.035280176	1.032557432	0.990398079	0.941844634	0.951428611	1.002778479	0.996640113	1.009374652	1.044622104	1.090966464
P41216	0.985898734	0.984347156	1.011256637	1.028465193	1.007007373	0.98873299	0.988066112	0.992260717	0.982575699	0.980620078	1.030289774	1.036003803
Q05512	0.964418567	0.978600241	1.007681936	1.006818737	0.97877029	1.002060342	1.016143683	0.970915671	1.010261302	1.043546775	1.004458424	1.052344478
Q62095	0.993587252	0.956737885	1.02296014	0.956029307	1.031038647	0.966616167	1.013578012	1.013762895	0.989245647	1.06379888	1.03991196	0.987520371
P35436	0.983068899	1.006812322	0.971934007	0.968228638	1.00456044	0.955223703	1.021640877	0.992577665	0.984512076	1.093057282	1.060396848	1.006210396
Q9D1E6	0.998360458	1.018982405	1.019524326	1.015589332	1.037094684	1.004442033	0.973545993	0.987389028	0.94978109	1.057383196	1.006367	0.99067448
P63085	1.07906055	1.116178933	1.217974734	0.741922199	0.723158674	0.715052168	1.115264464	1.747588123	1.319655081	0.712018101	0.667909375	0.628672022
Q80UW2	0.968508726	0.998591171	1.057740101	1.083044017	1.132255007	1.022086052	0.878778225	0.89678811	1.024969089	0.862661177	1.025284062	1.035428639
P23242	1.19825591	1.087157892	1.127652782	1.093443291	1.101957256	1.125348898	0.888504325	0.900100987	0.897657541	0.895621307	0.866048369	0.888112665
Q9CPP6	1.071919203	1.044983262	1.020200698	1.002302756	0.869368739	1.080388188	1.06402465	1.030523706	1.118272125	0.778278469	0.873790875	0.978643798
Q8VHL1	0.970310186	0.953966261	0.94717381	0.980704944	0.960825663	0.971828229	1.040340927	1.027987221	1.017220784	1.081483187	1.032739618	1.055231814
P08556	0.978508257	0.949551674	0.967332056	0.968962366	0.985325123	1.014751481	1.03198873	1.028807917	1.049799928	0.998466941	1.015057907	1.015497977
Q9QZB7	0.972292759	1.008045219	0.998759982	1.027829737	1.015333394	1.002030658	0.994582386	0.993585074	0.981604119	1.026775655	1.00473308	1.012653204
P27601	1.019164874	0.990583269	1.033692903	1.036899362	1.065907921	1.046483926	0.976394271	0.949142708	0.9691704	0.957204844	0.994246532	0.986395068
Q8BJI1	0.919357375	0.944759234	0.989648663	1.012158479	0.988949958	1.007922721	1.017709399	1.03027639	1.019640526	0.984911309	1.039197775	1.040424641
Q61937	0.931945934	0.977382844	1.069461134	1.065864131	1.004845339	1.023526592	0.979168631	0.99128589	0.956476494	0.994787228	0.993100182	1.037787891
Q6A065	0.985849314	1.023784122	0.993424779	0.995836991	0.972383632	0.980361626	1.000537593	1.004066285	1.026008829	1.013381712	1.006734484	1.018873635

Q64433	0.979668286	0.945688149	1.042954238	1.036866465	0.944830771	1.032884525	1.010720189	0.975744339	1.04062998	0.860580679	1.004727972	1.082293487
P31650	1.419497756	1.07698297	1.144189047	0.921135366	0.866959268	1.038526757	0.877603034	0.951444359	1.055520132	0.951415673	0.847230629	0.917880008
Q8CBE3	1.014000039	1.006711421	1.010932423	1.010318869	1.015396607	1.04539779	0.989131082	0.965814295	0.990940916	0.934905153	1.006984112	1.01493821
Q8K394	0.970982294	0.943103634	0.952035585	0.989339123	0.983391541	1.018639298	1.039462761	0.979814845	0.994825075	1.097837638	1.026442619	1.062145963
A2ASQ1	1.164984789	1.069553168	1.018686496	1.048996764	1.037335489	0.976808901	0.920123522	0.941842697	0.988496241	0.892258141	0.972827841	0.993143295
Q8BR92	1.039235509	1.040650204	1.03443213	0.998864204	1.05735103	1.014030518	0.982843705	1.025093824	0.948700918	0.988157595	0.969527519	0.937170553
Q5RJI5	0.99149925	0.984927355	1.008682046	1.024744298	1.054380731	0.985950949	0.970625619	0.999318005	0.970883736	1.085324596	1.00701494	0.983646893
Q9CQQ7	1.030737323	0.970914844	1.033721057	1.019106381	1.039034934	1.068841498	1.023465358	1.011154594	0.995456179	0.878368186	0.947598548	0.967797462
Q925I1	0.98552088	0.931816058	0.97790668	1.013033766	0.987938784	1.031736893	1.028540021	1.001540139	1.02131457	0.985188097	1.008737783	1.038351254
Q80WJ7	1.010550354	1.013735034	0.960929948	0.975831334	0.979823692	1.004398322	1.016677897	0.970060224	1.03143202	1.095691829	0.984297734	1.021282911
Q99MR8	1.051940839	1.016583456	1.038500963	1.05384608	1.060030405	1.018173582	0.936020071	0.981150816	0.954836622	0.96304484	0.997955505	0.962389016
Q9D1K2	0.949512201	0.973632338	0.987402331	0.978853218	1.045369718	1.045404611	1.0034378	1.001003119	1.034181844	1.051125657	1.015634861	0.944554293
P14231	1.231951005	1.040065694	1.016271158	0.864470151	1.152012016	1.09871928	0.864391072	0.878435444	1.009251991	1.141731604	0.954518903	0.884763371
Q6PGF7	1.025900943	1.003830448	0.983337976	0.969800969	1.001772688	0.980570005	1.017899933	0.992727359	0.962761457	1.044840177	1.028680126	1.032213928
Q9QZE5	1.008061318	0.98513539	0.989125631	0.989999731	0.986458141	1.009274607	0.990476554	1.002297272	1.009843521	0.993371929	1.010028776	1.046725253
Q6PDM2	0.971263808	0.968843313	1.002173536	1.009624375	1.020043982	0.984121704	1.013872309	1.012271328	1.030140543	0.982246189	1.006348421	1.00461215
Q9CZW4	1.049803391	1.067495419	1.022687042	1.027538626	0.997797296	0.985378919	0.962443482	0.976248346	1.017270035	0.956051112	0.969235689	0.989682589
Q5PR73	0.899353588	0.941962228	0.953474011	1.026322713	1.002098469	0.937845411	0.95941818	0.984882655	1.047568335	0.973608564	1.111992482	1.147629541
Q9ERK4	0.997002564	1.003065773	1.011269313	1.003403291	0.972388129	0.969986238	0.989922535	0.996136777	1.011308398	0.96965944	1.030535067	1.049186888
Q3UHB1	1.041941364	0.987918197	0.975480338	0.957796088	0.936725824	1.061677249	1.067219562	1.0102544	0.99085944	1.04803801	0.952323184	1.019096837
P35802	0.908771763	1.009609955	0.998878032	1.022825458	0.99902422	0.830124171	0.975233951	1.009894864	1.02363582	1.004410623	1.123714711	1.083007156
Q63844	1.037850694	1.004871098	1.050573205	1.065175498	1.096636287	1.11617568	0.939686098	0.961953222	0.951320795	0.939964104	0.93236595	0.944412555

Q9CQF9	1.052321817	0.96003359	1.034074285	1.019825654	1.003968385	1.066546902	0.989344573	0.941540545	0.993274831	0.915077828	0.990234722	1.039848389
Q6P4T2	0.98772514	0.934524308	1.0166955	1.002524058	0.957138611	1.008772689	1.034417903	1.015550548	1.016103573	0.932230434	1.044467339	1.025517397
P30999	0.944279166	0.934600784	0.954281689	0.993860868	1.01225054	1.005652849	1.03276552	0.985491888	0.998571999	1.117432737	1.050553643	1.02785346
P60487	0.939581235	0.980666305	1.049277069	1.062820399	1.022752023	0.99784395	0.966964843	0.998442386	0.992912195	0.873413869	1.0376512	1.042845039
Q80U23	0.998901414	0.975774311	0.990638547	1.008580505	1.026589169	0.978793744	0.996856838	0.980644077	0.940527176	1.098451221	1.045630217	1.028162113
Q8BMA6	0.98866229	1.041858682	0.987117859	1.031357218	1.000927191	1.005656415	1.000955185	0.988821463	0.98303526	1.006971416	1.005321887	0.991204088
Q9WUK2	0.94352874	0.952020052	0.972440806	1.011231077	1.003315101	1.003093635	1.013502327	1.011326181	0.967157887	1.154092457	1.011555923	1.041732242
P62754	1.036358987	0.980916967	1.007216023	0.979192271	0.994451169	0.965416302	1.048724583	1.013656171	1.018686418	0.946560596	1.000515565	1.001967995
O35465	0.967498605	0.970287602	1.015906122	1.020819375	1.023499207	0.980222108	0.992173102	0.987696769	0.994889588	1.018244139	1.029397477	1.025143029
Q99020	1.004832305	1.003545757	0.993070246	0.987392448	1.005989244	0.99284864	1.010795825	1.00623738	0.992327236	1.170070506	0.961272141	0.971591035
Q91Z53	1.079616675	1.039938805	1.017391703	1.007614219	1.087677152	1.110956978	1.005758192	0.953946721	0.931809338	0.994629907	0.917842103	0.918020547
Q3UHD6	1.021200869	0.961731357	1.006514595	1.009348071	1.074238981	1.072489072	1.01661725	0.989753896	0.979214387	0.933903784	0.982336306	0.961720216
Q9D172	1.02470011	0.989216916	1.01650183	1.041221776	1.069918549	1.12752906	0.972683716	0.914830161	0.926946281	0.97469592	0.996018307	0.992686916
Q61001	0.994668374	0.935508551	1.024705699	1.08254087	1.147357949	1.025063889	0.966706739	0.953559988	0.865495104	1.146648768	1.037020467	0.934605813
Q80ZJ1	0.999907995	0.964581446	0.988185129	0.968362422	1.011725207	1.001130684	1.043250729	1.031580443	1.018209939	1.011318936	1.004861107	0.972099352
P14148	1.029627862	1.009030141	1.009615937	1.004002242	0.955263837	1.016731924	1.029929368	1.032821339	0.993427814	0.910888437	0.979683689	1.017783649
Q9EPL8	1.06087858	1.036861852	1.017539823	1.008716606	0.987279536	1.015797342	0.985185478	0.983427023	1.032900326	0.999339267	0.947867724	0.961447337
P40630	1.022145924	1.074100755	1.069961832	1.064074767	1.059965232	1.106759709	0.9484332	1.010486793	0.912452082	0.876686679	0.940605566	0.92337338
O09111	1.08907952	1.095173772	1.033453264	0.963099999	0.955452016	0.993243901	1.015842099	1.042827304	1.037204593	0.936300196	0.91429549	0.929040018
Q9WTP7	0.997488133	0.993778747	1.014864742	1.018434285	1.044416145	1.045303063	0.993680126	0.99121814	0.962520298	0.952222039	1.017285354	0.979496692
Q9ESW4	0.96533304	0.934868069	0.971385214	1.006065502	1.001880824	1.04864392	1.038361113	1.053312163	0.943212219	1.060347916	1.005227662	1.019392352
P57722	0.936716476	0.985926144	0.943171454	0.971473061	1.006586808	0.987862574	1.047711368	1.008660585	1.000914908	1.03218982	1.026832836	1.0750921

O55029	0.882174866	0.993363662	1.044133583	0.99242646	1.181672039	0.958462178	0.904489939	0.95606717	0.95709245	1.140906318	1.110581851	0.945777422
B0V2N1	0.938282578	0.971965664	0.979798951	0.989234796	1.004787705	0.967442784	1.016197844	1.070538217	0.977097651	1.113553863	1.024484019	1.000685454
P02089	1.2941588	1.220664118	1.290157838	0.519605156	0.595822039	0.419848733	1.367044612	1.64486413	1.800984192	0.619539048	0.516342721	0.373859529
Q80VP0	1.138342958	1.077563601	1.044267404	0.939789654	0.967888185	1.02217082	0.992122166	1.012907948	1.014911299	0.988055333	0.910845131	0.931042691
Q8BX70	0.948575234	0.956700043	0.980986061	0.996088585	0.987765883	0.985116614	1.015603831	1.010084191	1.000334597	1.010732434	1.043561264	1.077992599
Q9CQ60	1.048631383	1.012294262	1.037614571	1.018525574	0.984621789	1.011285343	1.01009051	0.992664266	1.013120417	0.926823357	0.966421198	0.979174475
Q9Z2X1	0.971569466	0.913212078	0.962014398	0.983741068	0.986036624	1.022181891	1.063308839	0.992692944	1.042679104	1.023736416	0.961504918	1.106590463
P32037	1.016359765	0.981472792	1.003622617	1.047696771	0.987005592	1.013984403	0.995271081	0.970030641	0.954089524	0.994876554	1.001657092	1.073399225
Q7TME0	0.906590768	0.977124234	0.949728971	0.963729069	0.970835009	0.940031822	0.993190133	1.087972529	1.057815892	1.185324123	1.027999607	1.005228006
O55091	1.033794774	0.999952999	1.081349076	1.04242873	1.000975001	1.058117216	0.922280461	0.950372889	1.016552163	0.919855924	0.976281042	1.005894393
Q03734	1.119822472	1.307373896	1.151756259	0.82364995	0.917309776	0.900416289	1.121875062	1.031161204	1.002345966	1.051073612	0.840350523	0.769178099
Q9WV55	1.018505851	0.982173397	1.001230691	1.013689226	0.955727617	1.043297586	1.049174403	1	1.037232205	0.986023432	0.931213412	1.004434963
Q9Z0P5	1.014499688	0.959199023	0.924028037	0.947790227	1.020617234	1.010044171	1.144670857	0.970500996	0.934347551	1.112293611	1.009491389	1.0236614
P38060	1.03567287	1.077689322	1.031956279	1.04439609	0.985219544	0.96817461	0.960438877	1.014517033	1.059060793	0.840108735	0.954124634	0.994260741
Q9CQC6	0.969856058	0.932986161	0.979879361	0.933660771	1.071267382	0.989985175	1.035534694	1.039048054	0.984358513	1.066420409	1.032378664	0.999890171
Q9D5V5	0.982885224	0.993069859	1.007859431	0.985071176	1.028107209	1.015568685	1.029184278	1.023127527	1.002467338	0.969573344	0.997512979	0.968650174
P62264	0.9975273	0.9698814	0.963242668	0.983528855	0.983138881	1.023408236	1.021537615	0.983857772	1.01600486	1.122916109	0.983271767	1.027671746
O35639	1.103367995	1.033536438	0.987462454	0.971978079	1.028354691	1.029726569	1.034075333	0.986389228	0.998304291	0.956070072	0.948919026	0.947850853
P34152	0.966326194	1.067889866	0.945184999	0.943251145	0.940430022	0.967879705	1.075163791	1.005729984	1.040050097	1.034374974	0.998211908	1.033303052
Q99L13	1.788328052	1.348647772	1.155475588	0.958648669	0.940967609	0.922887604	0.808395325	0.854920832	0.845399618	0.751369168	0.867247585	0.838049218
Q9CZ30	1.022173614	1.045010279	1.019061254	0.961348472	0.990353158	0.967299474	1.046881239	1.062492003	1.021655032	1.006718472	0.946805649	0.928373879
Q8CBW3	0.936585802	1.005494551	0.986840532	1.048577704	0.992444426	1.043002818	0.986031434	0.955973364	0.994279318	1.0153909	1.021695794	1.045265966

Q9JLN9	0.982274318	0.982104136	0.973319032	0.988503134	1.02124525	0.998568146	0.993399676	0.98316718	0.963419609	1.057032765	1.058327502	1.043984488
Q61029	1.037729557	1.020281859	1.053996475	0.989641208	1.072722691	1.031493198	0.98517449	0.986412171	0.969838395	0.988037121	0.956012549	0.94544298
O35874	0.975535515	1.042590582	1.054472656	1.034701572	1.070648624	0.941648735	0.905999903	0.971381534	0.972802839	0.920247961	1.078590879	1.02232701
P49722	0.92049911	0.91061198	0.989893018	1.065269703	0.926676433	1.052097968	1.027192695	1.026844543	1.041123508	0.974063017	0.976436557	1.091859894
Q9Z1Q9	1.070071941	1.055074269	1.078194078	0.960108955	0.955617896	0.954097204	1.012040668	0.999953574	1.119432775	0.923580543	0.923950639	0.928784885
Q80X50	0.942718571	0.955688531	0.971029033	0.966252582	1.023685974	1.029550921	1.038881482	0.958941812	0.996050946	1.197321016	0.991417039	1.029671426
Q9CXZ1	1.025358574	1.012972678	1.037968898	1.036677948	1.084927467	1.050145469	0.944469502	0.949119775	0.954320112	1.031987841	0.968479884	0.971335443
P68040	0.984695664	0.928991145	0.97142452	0.962921447	1.003837525	1.008587945	1.019570646	0.992222816	0.992079934	1.07543962	1.060027976	1.04182189
Q80UJ7	1.002818522	1.01522352	1.023785371	1.007820101	1.007170473	1.023735227	0.99242331	0.98055465	0.957244911	1.007658476	1.008538803	1.00793229
Q8BRF7	1.066329532	1.011138818	0.997351567	0.982436035	0.971435594	0.993742933	1.009650004	0.995736345	0.992528224	0.961433675	1.000814609	1.032816639
Q8BH66	0.950925509	0.96389643	0.989531995	1.018504911	1.010110559	1.024371549	0.99221163	0.970649569	0.990475354	1.035227002	1.042901032	1.044510699
Q922Q1	1.019826499	0.983424124	1.036784351	1.036843284	1.011742242	1.03151552	0.962314691	1.014800129	0.986201185	0.936265898	1.00029589	0.985753194
Q9CZT8	0.946112278	0.931533127	0.995007347	1.006484722	1.051733385	1.027463666	0.992861111	0.978136769	0.903477459	1.095293719	1.076173079	1.060399774
Q3THE2	0.945311246	1.016929084	1.01048082	1.006838456	1.20981243	1.01865093	0.968560434	1.028954686	0.891244961	0.960435349	1.031718297	0.93179869
P59999	0.946651511	0.970633878	0.985625485	0.999214685	0.930438208	0.99247074	1.060433258	1.034492326	1.024743142	0.930365984	1.025007919	1.070487505
Q9EPL2	1.021679119	1.059859811	1.02593536	0.91070469	0.924535769	0.913263582	1.122359517	1.053611919	1.072760907	0.969546296	0.963568543	0.937706904
Q80TQ2	0.962315587	0.973389217	1.008713801	1.058755772	1.01847788	1.010941376	0.958917999	1.029920079	0.954986394	0.99131937	1.025315716	1.032798259
Q9Z2I8	1.062462841	1.013727595	1.025384762	1.005190705	1.088285712	1.051025968	0.972749327	0.978580188	0.926470517	1.027920703	0.979245896	0.933997617
Q9WV69	0.839264758	1.020359548	0.924463454	1.048586181	0.953561425	0.994774727	1.009061623	1.003212838	1.038422005	0.956644249	1.076306839	1.111661288
Q5DQR4	1.033652681	1.117290699	1.014127359	0.941039761	0.973269646	0.938344442	1.033167435	1.003837505	1.031430058	0.959189459	0.979751387	0.973033079
O70435	0.998924598	1.01291793	0.991046975	0.976227522	1.026880039	1.030842549	1.007738499	0.986006772	0.988591886	1.056192707	0.985989551	0.991031992
Q60771	1.721819087	1.400379312	1.336480791	1.442145509	1.415283642	1.473452491	0.566012964	0.61061672	0.576256264	0.59777591	0.528721163	0.544251034

P62746	1.041968978	1.036570733	1.023775084	1.086783033	1.025308398	1.02807319	0.95051785	1.017084917	0.961931216	0.856234389	0.984818763	0.976655296
P29699	1.178167137	1.270089231	1.252043686	0.795058618	0.75446202	0.741514796	1.112419155	1.202969634	1.295477881	0.771909505	0.74658126	0.743448228
Q9QY76	0.960261886	0.975735107	1.011232047	1.012370302	0.982390411	1.060019265	1.028497673	0.988012882	1.021317285	0.952920834	0.992805138	1.010912363
Q9QUI0	1.027830263	1.002887468	1.023605661	1.040825854	1.025043469	1.003799797	0.968055257	0.993860303	0.993337311	0.932932536	0.991633742	1.005559986
P70670	1.008642617	0.974787149	1.015186743	0.928680003	0.986393156	0.969321323	1.065076894	1.031067626	1.028355858	1.141495735	0.944228007	0.976919543
Q9CYH2	1.114099309	1.033847394	1.110268042	1.028452505	1.046887866	1.140128001	0.935426693	0.955927015	0.945029462	0.913546684	0.907033098	0.904965366
P28028	0.962177771	0.975435418	0.962489634	0.997006612	0.974938688	1.016818402	1.007249122	0.992196852	0.993078951	1.056020825	1.031779095	1.072278081
Q61151	0.944758236	0.931093974	0.982686348	1.019101815	0.965316666	1.010212638	1.042426212	0.993069402	1.057002904	0.921014844	1.003908875	1.104939133
P04925	0.980036318	0.995014039	1.014833663	1.041764906	1.127361019	1.050602555	0.988084031	0.956500996	0.979216955	0.966764559	0.949350338	0.985051546
Q02819	1.04256448	1.072341185	1.038265503	1.016567912	0.959762157	1.025799256	1.023488524	0.989119485	0.981336462	0.857882567	0.989138925	0.975001628
P49312	0.995642924	1.003542963	0.986865707	1.010882508	1.024732245	1.001972817	0.99339388	0.996172771	0.925516138	1.192696499	1.005569029	0.979593991
Q9CRB6	1.111789963	1.015009841	1.105124923	1.12491906	1.271258594	1.312711545	0.812575071	0.888376136	0.854504981	0.814332943	0.874018143	0.871277381
Q5DTY9	0.897372523	0.965042647	0.921026316	1.002523762	0.927121782	0.929131629	1.017299699	1.042810356	1.033829383	1.047371658	1.105060817	1.116388841
Q8R326	0.978314619	0.994102384	1.020019122	0.991876309	0.972948 5	0.98667366	1.022921104	1.00220409	0.998845187	1.024424095	0.9978168	1.036252115
Q921M3	1.089502764	1.008268554	0.978655253	0.983059347	1.065206744	1.006878931	0.966578516	0.957119194	0.981471206	1.058499714	1.008716371	0.966166647
Q8CDG3	1.008308925	0.981421397	0.944875562	0.965898936	0.97557966	1.02528001	1.05063424	0.990016174	1.049220799	1.045849955	0.991122338	1.006443496
Q6PDG5	0.965698715	0.970111028	0.990819427	1.0046062	1.017762909	0.987623551	1.012494357	0.998544621	1.019349523	1.068173056	1.001641775	1.007788966
P46061	0.95685556	1.008295486	1.0045255	1.001862169	0.97960461	0.990868986	1.032442011	1.030011025	0.973531727	1.010279797	1.017603955	1.010751418
O35633	1.014236536	1.002591839	1.060963758	1.040312171	0.950617649	1.002333964	0.958016689	1.012536945	1.010921946	0.940736555	1.011924491	0.991166303
P50580	0.984357107	0.971156871	0.999811355	0.968191766	1.018754384	0.991616867	1.005191143	1.002728099	0.971304788	1.114760418	1.034331048	1.001719254
Q9Z268	1.112183751	1.096246519	1.15501175	0.92164223	0.882418379	0.890481501	1.105174703	1.023535505	1.052107241	0.877320955	0.91638721	0.920214761
Q8BVQ5	0.961750403	0.94215922	0.950591688	0.988265338	0.97397086	0.984402002	1.030238014	0.960958351	1.030272662	1.074015762	1.048380653	1.094653599

Q8VHI6	0.982675329	0.994420075	1.020334093	1.008979305	1.009051734	1.025138169	1.024559117	1.018458991	0.966164527	1.099479341	0.942924315	0.981904444
P63325	1.036840594	1.037390715	1.012287393	1.025871936	1.00623969	0.99736783	1.006356072	1.009033302	1.005980567	0.838725755	1.001432929	0.983511412
Q64523	1.403959556	1.24148319	1.087053983	1.138904697	0.604908042	1.331395974	0.950909791	0.858911674	1.112019378	0.499361359	0.668008196	1.033578099
O88741	1.002725314	0.991492175	1.013259315	1.011135722	0.983139956	1.006619434	1.012778429	1.006619077	1.003631085	0.966343159	0.996868021	1.013541389
Q6A0A9	0.981697384	0.981180365	1.016087333	0.944708343	0.917890644	0.915040853	1.062483767	1.131489044	1.065100302	1.03249166	0.965559098	0.987626413
Q8CDN6	1.02198095	1.00776769	1.031822051	1.015238758	1.036463394	1.040525688	0.968500365	0.97836248	0.967868761	0.958890284	0.988392775	1.008128434
Q9Z0U1	1.092940343	1.020844647	1.036547789	1.017966418	1.09838017	1.05035567	0.928167865	0.959495919	0.976083231	0.985092604	0.953805755	0.935760836
P84084	0.968658958	0.959039984	0.980492071	1.006600876	1.027672198	0.993409735	1.024633096	1.00083757	1.015285293	0.972685129	1.027340666	1.025583374
Q6NS52	0.852480094	0.953827352	0.962193898	1.11817711	1.093531791	1.032856928	0.937674653	1.025222865	0.958160791	0.893480546	1.060951522	1.093587941
P61027	1.029558906	1.074339487	1.000267024	0.986981686	1.037793488	0.998017551	0.914496322	0.988559855	0.9097254	1.091133428	1.03662977	1.016827691
Q8R3S6	0.953063885	0.950164587	0.963352979	0.998273496	0.980651438	0.994228844	1.046269913	1.021409244	1.035691223	1.032938523	1.017490418	1.024583166
P58771	1.097192753	1.150596967	1.011313588	1.107998838	0.807274515	0.992253726	0.944302211	1.032451624	0.976042399	0.69967086	1.015713083	1.085692624
Q6P8X1	1.173176545	1.016509985	0.90651912	0.92256668	1.100185952	1.113292166	0.908688009	0.899223815	1.075657335	1.170051951	0.92005938	0.938429078
P51174	1.087617662	1.014654696	1.043997283	1.052102062	1.049094052	1.063397236	0.939893986	0.951335572	0.943750405	0.968597356	0.968904281	0.967434399
Q9D7G0	0.984914871	0.985815498	1.014744806	1.046119939	0.986354047	0.974477609	0.959290761	1.024467318	1.023458248	0.919004036	1.037659553	1.025904367
A2RSJ4	1.041946019	1.014478008	0.990651479	0.987736125	1.014887193	1.010549675	0.99541279	1.004702893	0.981088098	0.992218677	1.006816252	0.987857893
Q14BI2	0.933924277	0.948950174	0.996846032	1.055376086	1.004958022	0.950435892	1.017477984	1.07448509	0.99749139	1.006326218	1.030807554	0.990499442
Q8CGF6	0.992551689	1.005468343	1.019222448	1.034108523	0.991747026	0.993004177	0.989504729	1.012873081	1.012785278	0.925516951	1.001331212	1.013285874
Q62442	1.288354724	1.167317075	0.980541222	0.956276226	0.907534115	0.99394386	0.914372765	1.000947673	0.995045828	0.854174813	0.984441524	0.964716298
P99027	0.916087344	1.116933233	1.033695186	1.005383654	1.042816435	1.020852295	1.100847567	0.976494864	1.03169589	0.820251714	0.946243307	0.923777809
D3YXK2	1.007899663	0.95994196	1.008667073	1.015876811	1.032753749	0.974381541	1.025838527	1.03060494	1.020261534	0.945389855	0.996309266	0.977099264
O08759	1.050295491	1.039994412	0.968793737	0.955871742	0.975579311	0.992846802	1.032599928	1.023870545	1.00353639	1.034927268	0.973350169	0.989972814

Q9JM52	0.948687364	0.989433349	0.975070872	1.026098771	1.005815071	1.014279551	1.001732754	1.009187934	0.973599754	1.043902067	1.026455185	1.025027535
P48320	1.149809582	1.007701442	1.145335853	1.014790303	1.008280089	1.075689777	0.868200811	1.046796818	0.917098339	0.886320421	0.932150936	0.970470177
O70325	1.202185633	1.08146947	1.160068265	1.014933032	1.093583531	0.995943504	0.989325259	0.927011217	0.888178665	0.926688363	0.884401916	0.891401999
Q61411	0.998598097	1.049804067	0.99247644	0.924157092	0.899410997	0.978433396	1.084842087	1.059841291	1.072358454	0.983712119	0.944947335	1.003690515
A2AQ07	1.047872028	1.091584249	0.953507638	1.014557421	0.89634845	1.005842491	1.154020769	0.9767364	1.013208297	0.895628829	0.909653419	1.031737474
Q9D832	0.983063629	1.02448661	1.058179844	1.026118971	1.055638456	1.015811613	0.964807059	0.984331842	0.966780856	1.026904357	0.992292908	0.944653297
Q8C854	1.024835516	0.955640176	1.000608649	0.998626982	1.02646342	1.007926819	1.001908878	1.004678312	0.981929325	1.094008876	0.982738391	0.992322828
O35864	0.99573697	0.982070681	1.028848347	1.034569728	0.977796067	1.010254636	0.964292829	0.991455949	1.005715715	0.945667807	1.015252789	1.051300797
Q920E5	1.064923408	1.05376518	1.057759109	1.023287884	0.976772233	0.995806269	1.010401605	0.98523548	1.008696932	0.920562794	0.935500857	0.973442094
Q8VD75	0.983285806	0.981821527	0.987407653	1.001282129	0.998019017	0.988695312	0.994381727	0.991054056	1.028260559	0.965666107	1.030523458	1.050773599
P16332	1.00207242	0.981541781	1.006360733	1.0509047	1.036190006	1.094425257	0.954581223	1.020084861	0.939787932	1.019042082	0.981249409	0.968499689
Q9D7N9	1.025637531	0.985994415	1.002864244	1.002018988	1.035543727	1.014172069	1.002348522	0.992098727	0.953744885	1.027529169	1.00458149	1.000536014
Q8BMF3	0.945083356	0.945162911	0.967557409	1.073119489	1.058785754	1.059361407	0.994799021	0.979796118	0.951225216	0.965784709	1.024209189	1.057837544
Q9WUA2	1.015146596	0.98637235	0.994386131	0.981530541	1.004636202	0.987226827	0.998704456	0.987251959	0.999796748	1.004501369	1.020271808	1.045495867
P62071	0.797114248	0.827511992	0.807872721	0.808586581	0.800672322	0.802779119	1.212018581	1.184568637	1.226438702	1.195371751	1.146186363	1.157854313
Q9JMA1	1.106336676	1.077218794	1.082262975	1.055842982	0.981551528	1.005360597	0.939642281	0.96298831	0.956047058	0.888594435	0.937644783	1.025126131
Q9R1P4	1.013705869	1.002288952	1.013112815	0.997809358	0.996986423	1.038018784	1.024116932	1.014756509	1.038011852	0.946464056	0.936435226	0.983525459
Q3UTQ8	0.936750446	0.941630456	0.945960228	0.949190506	0.98173084	0.964882392	1.074064684	1.034458273	1.075585197	1.050746507	1.040914419	1.008752888
P58404	0.952458805	0.971842727	0.985904182	0.979256883	0.969031731	0.917028083	1.028537657	1.048260269	1.05776037	1.008527442	1.034929203	1.042216514
P63137	1.033874087	0.991152816	0.949128928	0.974496484	1.042268829	0.993604155	0.989715746	0.952507599	1.01474361	1.082122636	1.040608296	0.996381998
Q9CXJ4	1.296672083	1.132749072	1.060301873	1.157368936	1.260680422	1.251368908	0.801502739	0.805993771	0.822146566	0.823752962	0.850183701	0.842740811
P35279	1.009421227	0.995097189	1.01018804	0.983695281	1.024060707	1.034477451	1.043951935	0.979565878	1.024790378	0.98710583	0.954341832	0.973884914

P60122	0.995908412	0.982210875	1.013608435	0.993806664	0.994877109	0.97387456	1.02077581	1.047252224	0.996393476	1.053156749	0.98441346	0.983049169
Q9R0Q6	0.962368022	0.968983756	0.971375796	1.011486432	0.937397995	0.981139284	0.995953157	0.988297306	1.012246052	1.036978478	1.060216087	1.098847185
Q8BMG7	1.008942669	1.000072052	1.002114509	1.023969549	0.997357987	1.025822018	0.975891411	0.999152392	0.973700522	1.007296556	1.00623888	1.015966697
Q9DCJ5	1.056859153	1.02502096	1.052463077	1.092003544	0.99188299	1.03586423	0.944926953	0.95980458	1.037992137	0.742965035	0.967183461	1.033348178
Q3THS6	0.988443604	1.023309186	1.031263225	1.055197945	1.033292577	1.025530178	0.965308503	0.992923761	0.950323561	0.958656975	0.991758497	1.007835086
P18242	1.081672327	1.032334609	1.074606936	1.03240307	1.166662313	1.06958965	0.920310244	0.939730223	0.925999149	0.938208463	0.934632299	0.934401371
Q9D0E1	0.999687709	0.988308747	1.029407089	0.986037869	1.02554685	0.994555859	1.010723953	0.977273767	0.992029431	1.03958216	0.998632424	0.997739054
P62281	1.020977708	1.019667833	1.053733773	1.041564538	0.957182399	0.870216961	0.954008198	1.054197949	1.0881877	0.762034714	1.016934852	1.069855023
Q8R550	0.995749693	0.969962911	1.025053032	0.938378721	1.194213693	1.221919108	0.802359807	0.971470712	0.853450667	1.335402564	0.927178271	0.993324121
Q80X90	1.176788749	1.025393226	1.006508163	0.993734837	0.992149457	1.050832934	0.992532912	0.942784256	1.008171231	0.96174345	0.935084592	0.960272335
P51859	0.979358728	0.993879544	1.000127465	1.03107358	1.056863595	1.02485185	0.96626437	0.973188584	1.013851326	1.115640554	0.948442354	0.983980588
Q80XN0	0.909480561	0.917214015	0.916536966	0.94256	0.915359143	0.853614128	1.070785455	1.057179752	1.131524922	0.939801314	1.122524708	1.1539982
P32883	1.079276581	0.983973924	0.999093913	1.005989639	0.934280312	1.086534216	1.065643155	0.988649015	1.060194845	1.01478767	0.847960994	0.986409715
Q9QYS2	0.889690459	0.941567693	0.94063948	0.987274591	0.979209698	0.898372706	1.010298139	1.012327735	1.083899375	0.968689967	1.12301948	1.127534176
Q8C5H8	1.063462332	1.015601817	1.045465218	1.04491912	1.106777404	1.056528654	0.927410401	0.949063889	0.904583328	1.003085312	1.016891392	0.926919532
O08795	1.022726087	0.962524953	0.994816154	0.970971199	0.96859387	1.002139466	1.063414848	1.026881877	1.024551913	0.976492283	0.977675423	1.01460579
Q9CTY5	0.958844553	0.967109079	0.958466443	0.958750987	1.005274609	1.004195665	1.045382361	1.000652083	1.016061621	1.030971225	1.036973694	1.035676952
P28650	1.075817674	1.01121609	1.000090256	1.024657101	1.073474449	1.059930345	0.974800696	0.948555329	0.976289268	0.982284713	0.963281916	0.959272349
Q8K4G5	0.948245115	0.96453481	0.984292756	1.009112608	1.017718062	1.028110121	1.008320522	0.999903439	1.002445402	1.060632399	1.005093585	1.01541729
Q01405	1.009271928	0.99629627	1.015225086	1.015277968	0.999677637	0.971468903	1.022692948	1.022933354	1.005982214	0.920632102	1.005519788	1.000497407
Q6ZQ08	0.959367239	0.980475354	0.955838605	0.968511221	0.983708621	1.00674901	1.042041657	1.005424385	0.988091984	1.126168997	1.027384415	1.019534788
Q60780	0.946724246	0.961252574	0.966216344	1.020823432	0.987172059	1.053379866	1.035747516	0.985273985	0.998195592	0.975396575	1.023914706	1.052922702

Q99L45	1.04315232	1.102788834	1.019718052	0.941077283	0.939957809	0.966874081	1.032080364	1.040976258	1.028747847	1.070664225	0.942974693	0.91711858
P23198	0.972724123	0.965406296	1.013818936	1.011521177	1.020065395	1.00024722	1.023730777	0.977271706	0.977166777	1.080660257	0.999767749	1.014965057
Q99MN1	0.994895349	1.007997383	1.001442473	1.025560128	1.001339772	1.001356167	0.985114212	1.002385894	0.973142177	1.000960902	1.013426734	1.022052631
Q62277	0.928133811	0.953531132	0.988065003	1.025972867	0.968420741	0.953943674	1.029843679	1.027271068	1.039084474	1.003395357	1.037909425	1.043318086
P45376	0.97695432	0.99807132	1.030590035	1.058412568	1.056474044	1.037066765	0.961070416	0.962153211	0.991746603	0.885882327	1.031542574	0.992247558
Q9WU79	0.990016535	1.001231529	1.029616753	1.04764415	1.01726605	1.004911233	0.97889886	0.989087544	1.005656931	0.980990992	0.97436828	1.004888572
O89112	0.931008953	1.003535049	1.011527102	1.04165491	1.015619292	0.942477388	0.940932963	1.008813665	1.040845791	0.880890606	1.069798986	1.069225443
P56959	0.961099516	0.946313744	1.034422825	1.03800048	1.1717927	0.983719281	0.957613992	0.969137622	0.988221104	1.284793629	0.911309539	0.919718956
Q9ESN6	1.0039902	1.002833533	0.990806918	1.000421146	0.969821538	1.032156759	1.001371728	1.011236109	1.028257738	0.966415468	1.009494134	0.986329365
Q8VCD6	0.948611186	0.999235239	0.962714696	1.020820094	0.946385661	1.021059912	1.041558719	1.028883606	1.007801613	0.95989152	0.998789562	1.062655705
P42567	1.020597362	1.021608109	1.015322888	1.007989194	1.031991534	1.01842954	0.987664978	1.024267068	0.957828346	1.031454514	0.986723923	0.943681203
Q62318	1.008297168	1.022190352	1.024230225	1.031635399	1.017413593	1.030742188	0.968083609	0.996834988	0.979041205	0.967716452	0.994753874	0.980487147
Q9Z1P6	1.029725985	1.03862867	1.017447098	1.080528633	1.013780049	1.014000885	0.970951196	0.966214276	0.996216067	0.871609454	0.991656062	0.998906792
Q8C437	1.058002743	1.030964887	0.967619792	1.00569583	1.03252774	0.990666968	0.98202357	1.014128746	0.976236806	1.005849605	1.023620444	0.949424072
Q91Z67	0.980988624	0.966775104	0.984327047	1.017014789	1.035965489	1.016575122	0.975163421	0.965541757	0.96320161	1.074827134	1.041450277	1.039514836
Q3ULD5	1.040059178	0.962398834	0.984467823	1.031307417	1.067212192	1.044182022	0.970414427	0.97027811	0.996595849	0.985695408	1.01767883	0.961993538
Q3UJU9	0.934387489	1.000875055	1.005664142	0.955581885	1.013441549	0.988762265	1.013752852	1.022670872	0.971335626	1.061743581	1.053909599	1.006108763
Q80UM3	0.992423112	0.996755961	1.015164468	1.060320313	1.004383777	0.97870031	0.958358588	0.983729986	0.998333688	0.927580413	1.037153282	1.044092023
P31648	1.007246919	0.962085883	0.999257113	0.991786087	0.975541603	1.006231583	1.009554048	1.008444024	0.976465406	1.036263727	1.025372153	1.037853921
Q9Z1W9	1.047616516	1.075661934	1.039670066	1.058623019	1.034967148	0.983101989	0.921938087	0.958702207	0.942123769	0.917116521	1.01495114	1.023756986
Q05BC3	1.056835589	1.025112475	0.941196293	0.966598032	0.978506427	0.981201015	1.001765972	0.994768057	1.047267247	1.023282464	1.008628696	1.008072074
P21460	0.998823159	0.97007854	0.974620902	1.051137333	1.059954043	1.092727971	0.998207142	0.955671779	1.021379171	0.991653766	0.954329705	0.971296568

Q3UUG6	0.936065466	0.948346353	0.970712573	1.038944453	1.047464958	1.015429524	0.994235191	0.975999358	0.94260782	1.096698119	1.053921569	1.045656396
Q9R0P3	0.99418745	0.991910633	1.057482206	1.07632178	1.009433479	0.919449595	0.907344072	1.009801329	1.011628442	0.829530542	1.069817121	1.072377572
Q00612	1.075551356	1.013385901	1.039179747	1.064509374	1.062402706	1.065779314	0.956548291	0.971762698	0.9786902	0.891328792	0.930118719	0.969070371
Q9R0N7	0.97719118	0.982332048	0.984150057	1.001590146	0.968806698	0.994818905	1.039191885	1.021317321	1.004145015	1.063988588	0.996644414	1.007134014
P56391	1.042116724	1.013262901	1.060229386	1.055557735	1.009401742	1.001267345	0.968988407	0.955987825	1.02777661	0.76432106	1.018623053	1.014583135
P31938	0.951400685	0.974199665	0.996256781	1.039550029	1.038167285	1.049167426	0.970489164	1.027718848	0.977819031	0.986236636	0.998305904	1.014435336
O08585	0.974155413	1.078632393	1.039360282	1.053451252	1.051144559	1.046442979	0.99948024	0.931098548	0.981075152	0.897019083	0.997880149	0.941259015
Q8CCB4	0.971096983	1.001248904	0.973616407	0.996216783	0.967715996	0.989183105	0.989929962	0.987050062	1.014737725	1.034389672	1.042759608	1.06033047
P28741	1.031039326	1.017631113	1.013229574	0.980888428	1.010848553	0.991573842	1.019110278	1.004448434	0.992426932	1.012255176	0.991398567	0.965052107
P47934	1.019979543	0.982492797	1.012223624	1.014398213	1.033561481	1.030315488	0.994399879	1.004133484	0.972886045	0.970845324	1.001186335	0.984126499
Q9Z2U1	0.959427282	0.984378605	0.971513586	1.008337068	1.102915627	1.024307287	1.029915364	1.010525538	0.998387648	1.013312105	0.994892779	0.929570245
Q8CHT1	0.916897151	0.984946812	0.978308352	1.062372385	1.072766831	0.983961507	0.994205854	1.025460596	0.942579164	1.04317128	1.033946285	1.003563147
Q99MI1	1.004254094	1.035934894	1.001911171	0.983298695	0.903648569	0.911182512	1.050649296	1.149403077	1.034341535	0.934951978	1.013987042	0.938484649
Q9JKB1	0.910911824	0.948879831	0.968048136	1.012255132	0.927618443	0.955772294	0.990531151	0.999327497	1.127653986	0.891570404	1.067315	1.139971014
P31230	0.930448833	1.094163881	0.958604401	1.001161734	0.999333523	1.008645966	1.007310328	1.004356235	0.950024288	1.003617833	1.03915429	1.024893578
P70195	0.985844135	0.989081256	1.002062696	0.996706684	1.025455464	1.018956079	0.993994567	0.997526746	0.990060591	1.071693738	0.990500266	0.994840095
P32067	1.022156397	0.999085243	1.007426978	0.997931716	1.011654641	1.034159434	1.022009184	0.981772891	1.00063472	0.986724002	0.967705661	0.995774529
Q9JMH6	0.99524551	0.993456075	0.998258602	1.003977504	1.010962804	0.979196986	0.942016861	0.963443305	1.002479431	0.933330627	1.083069371	1.086801977
P28867	1.292010615	1.199547628	0.914689628	0.998669422	1.029540017	1.022358337	0.917591185	0.99372588	0.95674434	0.822907674	0.977710504	0.890858268
Q99JI6	1.022551618	1.015936778	0.975226062	0.988754743	1.031498002	0.994362479	1.005385187	1.002643416	1.001844361	1.039079755	0.999869548	0.966174193
Q9Z1J3	1.033357791	1.005295198	0.993534894	1.017185646	1.018167865	1.059279433	0.960777142	0.971663329	1.019684337	1.012030755	0.96696913	0.988933858
Q8BG32	0.943932608	0.919736496	0.96157101	0.997616308	0.910879498	0.984473019	1.063993806	1.001226832	1.008420979	1.024284196	1.061914513	1.130763772

P59281	0.969098198	0.970508851	0.971185567	0.969883739	0.946839161	1.006900015	1.05224407	1.019341886	1.033461926	1.07223598	1.00788196	1.013973475
Q8BNU0	0.945465017	0.946758245	0.96098213	0.989831536	1.002582524	0.957388265	1.02534274	1.023051679	1.016604877	1.03896434	1.06913878	1.03872319
Q99KN9	0.968354281	0.982214264	1.016092617	1.014881508	0.978134974	1.002247019	0.979712664	0.99371345	0.995150012	1.022954272	1.019898925	1.056240134
Q3TES0	1.078729565	1.016371214	1.067525768	0.979748635	0.900377579	0.96414162	1.062146327	1.049289302	1.053452061	0.924638871	0.936402615	0.948622328
P08226	1.043014213	1.178293596	0.978948545	1.107597396	0.912170233	1.09617373	0.849844651	0.991979451	1.029282882	0.725955161	0.971715019	1.062910873
Q9Z130	0.968618551	0.989766113	1.100785379	1.028698091	1.066440782	0.865502558	0.948857585	0.964221414	1.026492851	0.922789152	1.032605048	1.067088608
P29758	1.024173107	1.01454571	1.054368895	1.041306088	1.089330618	1.04374212	0.954006235	0.950758739	0.917994297	1.006591854	0.989000816	0.971001411
P27659	1.060692158	1.021208598	0.986498462	0.988097381	1.001722354	1.03955537	1.023800873	0.975392848	0.975071788	0.970392398	1.008973893	0.970204175
Q9ESN9	1.017724844	1.01099132	0.98727944	0.965409509	0.946491245	1.003257645	1.038609084	1.0019359	1.019948555	0.958136431	1.021342512	1.023139839
O08756	0.975624562	0.951030876	1.030019137	1.004068715	1.058246854	0.993569349	0.968712556	1.01093263	0.972491156	0.97302734	1.053281522	1.015633078
Q8VHH5	0.986779601	0.971274415	1.007003808	0.998528697	0.965780874	1.029063517	0.99724117	0.980273151	1.028646267	1.01546469	0.999468956	1.046288331
Q9D0S9	1.053458036	1.005919284	1.041902112	1.010301726	0.971403868	1.036608838	1.020667851	0.948047396	0.981633791	0.920406385	0.996318405	1.014405807
Q6ZQ18	0.957168853	0.959454424	0.978382965	1.033151253	0.990753738	1.02173308	1.019696404	1.002791737	1.004744792	1.040161771	0.996553581	1.033417831
P21614	1.341169529	1.345652242	1.332183508	0.726438778	0.700644813	0.722140266	1.248860449	1.116909095	1.294004356	0.738397288	0.654335161	0.64239811
P35803	0.845512244	0.910551555	0.912618117	1.006259097	0.964436029	0.996156178	1.086149331	1.027441137	1.041171963	1.060617799	1.051740684	1.107840236
Q9QZD9	1.02606795	0.992946672	0.995223758	1.008767957	0.986117489	1.000984094	1.020255225	1.005295492	0.986444609	0.980707887	1.002192571	1.014370713
Q91WK5	1.158729031	1.050038991	1.102671406	1.019947257	0.987556285	1.032306075	0.884387334	0.892254649	0.906753525	0.913433902	1.016790669	1.07226161
P24549	1.000917234	0.828146898	0.895671368	1.038678107	1.030982898	0.962027706	1.039625057	1.005502619	0.94398915	1.084560691	1.068698459	1.167369245
Q6PHN9	0.990884074	1.074332647	0.985139866	0.979249307	0.986495836	1.013735932	1.016536379	0.985427485	0.998776531	0.983569477	0.998945973	1.003093355
Q8CCT4	1.058300736	0.992821644	1.028833052	1.010586759	0.88189987	0.938989956	1.089271239	0.931943758	1.064592554	1.215708889	0.865999348	1.045147908
Q71M36	0.914419208	0.975877159	0.968556614	0.987267544	0.996885516	0.970464996	1.028446324	1.034218627	1.06836233	1.050976247	1.01806195	1.000999342
P21836	0.97238634	0.988243374	0.984160791	1.146611904	1.042522914	1.048567967	0.918059244	0.965139799	0.956301542	0.922889951	1.004359343	1.076450683

Q640R3	1.041421348	0.992733281	0.993590893	1.0456358	1.065248644	1.077341844	0.929926819	0.946662608	0.942152917	0.982340927	1.022147618	1.008977606
O35382	1.040163408	1.060571928	1.018351416	0.938547403	0.965385379	0.90323309	1.007740263	1.086806179	1.080815099	1.021649281	0.974972625	0.910704539
Q62443	0.981391487	1.036078404	0.982967065	0.955092636	0.926049743	0.943601844	1.101610114	1.04430467	1.008736116	0.98009393	1.023014022	1.003654946
Q8C878	0.971780136	0.971062428	0.971104146	1.011514209	1.061248737	0.993770883	0.980833613	0.985489521	0.962876642	1.024151583	1.059994923	1.04126778
Q7M6Y3	0.977097484	0.998354904	1.000122954	1.002796487	1.035737087	1.010722296	1.001841251	1.006402043	0.950965479	1.075429479	1.016549899	0.98126508
P15532	0.960882653	0.961519071	0.990399183	0.993310489	1.023991597	1.024224711	1.027804824	1.014041039	1.028471761	0.940355791	1.008741981	1.012616353
Q99M71	0.987822114	0.973661818	0.961002729	1.003228765	0.991245136	1.009482115	1.000931783	0.999365102	1.022653725	1.002771156	1.023440979	1.044551583
Q6DFV3	0.964579894	0.981755429	1.020628024	1.026843848	1.037403027	1.024751637	0.993212998	0.999283967	0.957950321	1.010055943	1.013062693	1.001625405
P63094	1.075138126	0.995493153	1.040245966	0.979341365	1.075449146	1.050202096	0.988596727	0.96006116	0.949873963	1.012193762	0.975796115	0.950955791
Q8VBY2	1.020905731	1.037572225	1.062125324	1.010852907	1.034018091	1.008872654	0.999891566	1.000105877	1.000081506	0.926896545	0.962842386	0.935143809
Q61335	1.083189184	1.060256508	1.039579519	1.005965503	0.983638872	1.051917731	0.990891958	0.974881261	0.993054092	0.93462391	0.960795153	0.936617934
P12787	1.018356279	0.938308519	1.04186268	1.063988922	0.931916828	1.007840382	1.053480794	1.017271945	1.027171206	0.918567698	0.953698769	1.016418643
Q3TVA9	1.251185661	1.177636832	0.994929599	0.9713466	0.983655557	0.973233636	0.955111198	0.995329846	0.917700206	0.949427268	0.985794406	0.895415023
Q6Y685	0.966274374	1.014450734	0.977282918	1.058454428	1.002141326	0.962516206	0.94788411	1.013679251	0.994686783	0.941674423	1.045515879	1.076521285
Q9ERI6	0.998201562	0.948470415	0.955799525	1.044404537	0.942000326	1.006949001	1.022463385	1.051209781	1.038164511	0.956748381	0.999639197	1.034973394
Q3UMR5	0.986440024	0.997879811	0.992195782	1.02089049	1.040738928	0.952087874	0.976991251	1.024467497	0.96912505	0.948703826	1.06391421	1.024726223
O35343	0.959652036	1.020802726	1.039147211	1.003818829	1.038850855	0.937474972	0.933360743	0.985676105	0.985513237	0.985477564	1.084852117	1.030985024
Q00493	0.980629109	0.98259626	1.05192679	0.958920432	0.990371486	1.000939164	1.021680211	1.016188601	1.028194183	0.971522022	0.9916826	1.00025488
Q91VN4	0.994396576	1.011585135	1.03247116	1.046514314	0.948380437	0.983499358	0.970347053	0.993659761	1.015014533	0.899709793	1.019249936	1.065509698
Q9CZY3	1.022363311	1.028113328	1.043064867	0.97116192	0.98760496	0.961293093	1.048645092	1.020054161	1.015408678	0.969769969	0.983794941	0.947816936
Q9D154	1.223397588	1.073457498	1.039173567	1.006358646	1.133538192	1.162545031	0.898010635	0.899677996	0.88461474	1.070678955	0.887150491	0.865160611
P01869	2.833383951	2.426904986	0.954599549	0.889685078	0.690447825	0.69011059	0.737810965	0.681500645	0.70162483	0.604380748	0.519424176	0.52898417

Q80TR1	0.932779034	0.969318516	0.926859004	0.991066596	0.952423959	1.035146987	1.025782592	0.982873899	0.996977309	1.104303948	1.065742477	1.067647262
P60904	0.955098673	1.018047149	1.046080275	1.016943766	1.129235737	0.974281639	0.957201387	0.990478207	0.885063198	1.081413592	1.065945242	0.943654972
Q921J2	0.969251468	0.923235559	1.005990555	0.980315623	1.009813366	1.023448727	1.027724344	1.020155053	0.990275328	1.035251915	1.009077685	1.034413134
P47199	1.011417367	1.005864513	1.009000217	1.049160995	1.034081062	1.019459932	0.960866567	1.007574559	0.97619443	0.958511647	1.000350065	0.988164538
Q9D1G5	0.984778412	0.984444182	0.974980425	1.005607543	0.960495566	0.987889618	1.049237992	1.025691015	1.031551355	0.942908851	1.007943445	1.030620877
Q99K48	0.987395695	0.957748669	1.013695905	0.988129596	1.039298813	1.032873672	1.057612286	1.012890835	1.026319366	0.984716442	0.949026749	0.963675292
Q921Q7	0.928212436	0.994630892	0.973657409	1.003792502	0.973891669	0.974535321	1.017434351	1.059780588	1.0501283	1.054494101	0.994759122	0.998928407
Q99LR1	1.004432449	1.011331202	0.985387517	1.010378461	0.934431124	1.047625683	1.064377696	1.002199276	1.045059514	0.95110076	0.934482579	1.013002467
Q8BP92	1.060270468	1.021619943	1.02987655	0.988144793	1.038707146	1.05386942	0.979849111	1.005912978	0.961207571	0.960398035	0.968221177	0.959643709
Q07417	1.01857386	0.993595452	1.02486476	1.055360719	1.050304381	0.947355049	0.912282521	0.971397881	0.980041812	0.848229408	1.034269401	1.144508603
Q9Z1F9	1.020837192	1.005619491	1.035839663	1.014655217	1.027680859	1.029713373	1.002621091	0.998049927	0.966788434	0.973810142	0.979640838	0.968762141
Q6GQS1	1.021648164	0.94536388	0.984173383	0.932454883	1.064608365	0.992112849	0.997678539	0.979351989	1.019992896	1.266320451	0.982443613	0.948045888
Q924M7	0.985492754	0.987496309	1.007686118	1.050781237	0.994115302	0.984681567	0.986686164	1.000704558	1.005568864	0.975594559	1.014958831	1.019316811
P97450	0.883009903	1.400181016	1.045773178	1.264007194	0.798609694	1.009633341	0.847542093	0.918631069	0.931618163	0.636973166	1.130102443	1.016571558
P01837	2.63799827	2.378528631	1.65851061	0.808571317	0.636659396	0.599140496	0.656295504	0.738609843	0.671061544	0.466072687	0.44138287	0.432194173
Q6PFD5	0.996986118	1.100327357	0.892960338	0.995893693	0.998827713	0.992786658	1.025008123	0.970750033	0.920417241	1.155940198	1.012759256	1.046084713
P61294	0.985526117	0.986097306	0.998010175	0.960666422	1.004907302	1.016188463	1.0283688	0.965766265	1.073416239	1.025725633	0.968103873	1.012137545
Q3UHB8	1.065126678	1.105494609	1.028727775	1.102204715	1.136356455	1.085948219	0.885634458	0.942477406	0.90901306	0.928311809	0.950256338	0.916901045
Q8CBY8	1.02170725	1.031995316	0.964789922	0.998532605	1.026571467	1.030713375	0.979276493	0.982840262	1.003206695	1.128296362	0.958741293	0.967872591
Q8BUK6	0.96718469	0.967385039	0.983080159	0.984722466	0.954500851	0.972686886	1.037391395	0.98933472	1.022155353	1.054075362	1.050473992	1.041056624
Q9CQA3	1.01500452	0.972112782	1.040460842	1.030340931	1.004236192	1.030999484	0.985752283	0.993182352	1.020777313	0.89516661	0.986017814	1.010364372
Q9WV80	1.013713993	0.998159597	1.047748811	1.040568552	1.054222531	1.008070937	0.979786236	0.976690555	0.987699901	0.903725998	0.994821937	0.989599746

P60766	0.961780412	1.019401935	1.003700145	1.031400315	1.142811881	0.965218846	0.904466633	0.973057437	0.870173258	1.081921896	1.139349052	0.972738138
P20060	0.992871848	1.012564838	1.014995362	1.02261769	1.079123377	1.035755339	0.98001712	0.968168283	0.95366038	0.969901127	1.00907805	0.987157043
O08677	1.360584913	1.413049991	1.395367411	0.710231233	0.66077214	0.688927344	1.249827954	1.200979521	1.256471392	0.686054447	0.626306372	0.589308668
Q501J6	1.051966569	0.987656798	0.978945304	0.954081183	0.959148463	1.013862362	1.019960966	0.989565069	1.00379816	1.148744726	0.985097827	0.995797155
Q9CX86	0.96417603	0.942668332	0.99296351	0.976161633	1.018628517	1.064041892	1.005302126	0.977043212	0.983624179	1.106732863	1.010492677	1.024565101
Q9Z110	1.003021985	0.994935847	0.990214192	0.987654558	1.029676105	0.976029912	1.002937488	1.035732307	0.98722172	0.979424	1.024498468	0.999401829
Q9JI46	0.96324062	0.942965454	0.977713872	1.011150649	1.037120484	1.014468074	0.979551785	1.013569908	1.033159537	0.989762651	1.030525485	1.016772713
Q3TEA8	1.068278062	0.999789055	1.039451891	1.067075401	1.025593701	1.018881162	0.953888808	1.003080301	0.972557411	0.964208489	0.938051027	0.991447311
P47963	1.046306357	1.031063154	0.997377478	0.974082824	1.007971363	1.002686193	1.026608308	1.006487773	1.037167649	0.935440657	0.966387571	0.966788459
Q2NL51	0.952420347	0.947563081	0.955749225	0.98858046	0.971687545	1.029297573	1.010699736	0.978098544	1.012420207	1.066964335	1.026561087	1.104288235
O35344	1.00352272	0.988030387	1.032132714	1.014495703	1.007176434	1.006953034	0.984109502	1.006535728	1.008970777	0.87777751	1.026486253	1.011983222
Q9JL04	0.953166401	0.991245894	1.019895337	1.089780929	1.024127997	1.009854419	0.996544982	0.972994641	0.995037478	0.978268934	0.995449185	0.993708815
O55126	1.040616387	1.014495442	1.048168867	1.104530646	1.056525851	1.093228742	0.939025866	0.954826806	0.968069893	0.862799239	0.924047121	1.006241136
Q6ZPU9	0.943501582	1.005322188	1.026526542	1.035410851	0.956687125	0.891793999	0.975405178	1.045305729	1.041755076	0.862087289	1.084143174	1.066723965
P01878	3.448901508	2.176079412	1.398329529	0.732661061	0.62105933	0.674729722	0.570319837	0.567860151	0.562968928	0.521336056	0.508032029	0.512671061
Q9Z0N1	0.978474888	0.971461377	1.002043078	0.996473509	0.967409817	0.959602311	0.998492044	1.006652334	1.025979354	0.95195545	1.057756793	1.06869001
Q9DBP5	0.951386108	0.91917908	0.95998628	0.969823233	0.938991929	1.008383056	1.04808489	1.065607337	1.046635743	1.013289892	1.005981825	1.077054154
A2APV2	1.064758878	1.004098983	1.000564521	1.010786394	1.023181391	1.034381171	0.982293773	0.962752858	0.981197631	0.958775686	0.992481389	1.012590455
P35282	1.007331526	1.081612768	1.006568937	1.018148505	1.054838387	1.04056824	0.94292383	0.976081392	0.959708095	0.948035835	0.99347913	0.995291182
P48193	1.043237907	1.021200348	1.063721668	0.962473002	0.946290307	0.9092166	1.060178076	1.12108731	1.135698059	0.909864833	0.904851257	0.877879248
Q6ZQK5	0.98071221	0.985894789	0.962687847	0.992641891	1.030079551	1.01611971	0.993068141	1.002175292	0.999968185	1.022486244	1.034406109	1.00905376
Q91W86	1.018268576	1.008755895	0.985389921	0.998014733	0.98876356	0.994646869	1.003932032	1.026053586	1.029266912	0.980755476	0.990374535	0.98901688

Q5U458	1.026490896	1.024118943	1.018040056	0.997058888	0.989710271	0.946961251	0.997224788	0.999944525	1.023444822	0.956335786	1.028850911	0.988497403
P27661	1.035669322	1.232391329	1.065245339	1.099039889	0.939799456	1.075142562	0.928943704	0.991192413	0.890337363	0.718567386	1.002473139	0.962351569
Q5F2E8	0.998933547	1.015912757	0.984092244	0.987607289	1.019962262	0.979273582	1.015258983	1.004936201	1.004058725	1.002393294	1.023557153	0.981935256
Q9CX34	0.960681827	0.968632088	0.993753648	0.965796693	1.028316179	0.973570478	1.014036082	1.005662712	1.007927974	0.986700904	1.073279062	1.016473511
P61750	1.016425596	1.018442171	1.03716088	1.000047599	1.121083854	1.016705554	0.984020635	0.992610201	0.930719424	1.101062306	0.957889332	0.912694175
P63054	0.676806682	1.280677695	0.92450426	1.371766808	1.306589097	0.981745225	0.843014197	1.018391563	0.895042091	0.8232997	0.854277965	1.050826115
Q99PL6	0.962053792	0.959405561	0.987260132	1.026642691	0.991015768	1.035315886	1.032704635	0.999445694	1.020010424	0.948242001	0.984279366	1.053203979
P84099	1.006196953	0.992996177	1.009001109	1.020131394	0.944460586	1.038091088	1.013356226	1.012111534	1.023729987	0.950538143	0.969791684	1.023717699
Q8BLR2	1.014287806	1.005255918	1.011902748	1.08506707	0.984184105	1.028622701	0.953973218	0.992554849	0.965927624	0.904072958	1.010664222	1.046371319
P97384	1.062394611	1.040552626	0.974473323	0.974281363	0.986259658	1.034284873	1.069131544	0.960862497	1.013536941	0.952022195	0.958865477	0.987485779
Q9D6Z1	1.067971018	1.005724444	1.02270308	0.98435629	0.95329475	0.979448762	1.04336256	1.004074541	1.020951415	0.972336336	0.974960135	0.982021119
Q68FH0	1.02894928	1.018389479	1.04049928	1.024766114	1.045631509	0.984550621	0.95571684	1.005271052	1.000689006	0.892487905	0.983271631	1.011676776
Q8R127	1.024196496	0.978093204	1.027691923	1.01127995	1.016295883	1.056263576	0.9822731	0.994204171	0.949070109	0.981679397	0.978136163	1.036687089
P26883	0.970098009	1.017759547	0.993205687	1.024193151	0.994264182	0.982866474	0.995054644	1.052424129	1.014922619	1.014145322	0.987835403	0.97672616
P26350	0.91540557	0.99648675	1.019147349	1.107388787	1.490078604	1.124764136	0.925711048	0.966367025	0.889205416	1.051554419	0.771380947	0.878441475
Q01768	1.003983933	0.982086561	0.998350996	0.984409457	1.025049497	1.070306579	1.021711279	1.034115616	0.997646428	0.948844552	0.974449767	0.963101185
P54775	1.010029488	1.011188242	1.000670183	1.010729179	0.982711819	1.02026416	1.008023103	0.995695135	1.006758966	0.980740866	0.982816444	1.010472034
Q9WUM3	0.967035718	0.956652576	0.986938926	1.050060228	1.000851489	1.044150233	0.990472732	0.983316557	1.001223571	0.927570067	1.027246676	1.05952546
Q9CZX8	1.016179986	1.027496348	1.001170178	1.012562946	0.957510055	1.0097197	1.043864397	1.040459529	1.019980137	0.935789729	0.949031514	0.98355028
P36916	1.016244076	0.979841681	0.974284663	0.999879672	0.978635729	1.009798319	0.995747885	0.981796064	0.983012629	0.983647054	1.04613027	1.069665031
P63158	1.017740906	0.977700575	1.020188915	1.004019691	1.04531098	1.056932315	1.021327987	0.986119812	0.990436146	1.023566224	0.946298419	0.957349808
Q925N0	1.117768024	1.009214263	1.022477177	1.002094365	0.9713602	1.117598566	0.976448715	0.955665245	1.003693528	0.923027666	0.943190443	0.980518508

Q6A4J8	0.986361237	0.993439311	1.000949867	1.052829381	1.063755929	1.018644104	0.968852562	0.994992559	0.941890363	1.004152574	1.017557513	0.997580288
P62812	0.97549369	0.965698662	0.971287669	0.975004307	0.98664285	1.034651146	1.039247803	0.997533527	1.001616842	1.081098028	0.991303743	1.033426452
Q69ZW3	1.000730339	0.984606826	0.985628465	1.006955573	0.997874499	1.011513919	1.024593846	1.013940306	0.989931848	1.017366306	0.992378234	1.007014279
Q9CU62	1.036745126	1.057573695	1.00769377	0.983967684	0.977783415	1.008837035	1.018924212	1.001697104	1.010142524	1.006615193	0.967825671	0.952131268
P54830	0.931679627	1.009675978	0.98830008	1.10664558	1.035045807	1.060002703	0.947383426	1.008619639	0.965407915	0.840763271	1.025722937	1.051120962
P35293	1.021312685	0.999072081	1.000942605	1.01511552	0.993977402	0.99630003	0.990378342	0.961734231	1.025980807	0.956151315	1.00019832	1.050064743
Q7TPM6	1.025305929	1.009295015	1.011422091	0.987420945	1.038576369	0.997635489	1.012751582	1.022545921	0.979563914	0.986061652	0.998255905	0.951091668
P62830	0.991258639	0.971514533	1.010475371	1.004585065	0.947523482	0.97573854	1.027292815	1.036471987	1.013125063	0.946980926	1.018299877	1.043962571
Q3UEB3	0.951953097	0.958971232	0.996865193	1.010203117	0.965049508	1.043896612	1.032698034	0.990981243	0.989827844	1.082365626	0.991991681	1.038688734
Q9QXK3	0.985832334	0.967985912	0.970417624	0.996273255	0.964343144	0.994432981	1.016104691	1.018926156	0.996114437	1.009678413	1.040624759	1.056970853
Q8C1B1	0.947043065	0.964110692	1.015206416	1.023949718	1.024673583	0.991116806	0.988964873	0.982794529	0.988365949	0.997149645	1.042376574	1.049508915
Q3UU96	1.08426868	1.054667287	1.002835166	0.993603969	1.036798626	1.031180101	0.959972485	0.932295525	0.962743646	1.017346929	1.009767569	0.971364649
Q9R0X4	0.963097143	0.943526329	0.978798017	1.003959675	0.992979241	1.038652052	1.016042651	0.990259331	0.986253871	1.058106997	1.026928462	1.044269091
Q8BNY6	1.008739868	1.035000146	1.042108517	1.040382598	0.985123621	1.018070187	0.964477111	1.037119887	0.98214558	0.882874371	0.9937319	0.991619302
Q8BH04	1.014825554	0.96077464	1.032010606	1.026180578	0.992247611	1.085713719	0.999147501	0.975846157	1.016912275	0.964590862	0.968527337	0.980038899
Q91VE0	1.033766587	0.978975033	0.937031782	0.963592459	0.956614077	1.019607376	1.063335899	0.975196771	0.99983555	1.131651062	0.995993157	1.024124186
Q8R3B1	0.982457127	0.984697057	0.99160049	1.039593427	0.998879207	0.99396833	1.009244863	0.984935587	1.012988561	0.93261057	1.027453324	1.033366058
Q6NVE8	0.929230488	0.954811164	1.004651935	1.012549921	1.033365175	0.992757049	1.013801079	0.992906336	0.967682112	1.116697773	1.027126391	1.018363344
Q9JKC6	1.165448602	1.391080202	1.077030389	1.118412464	0.658804161	0.890302092	0.953928738	0.873335191	1.036260837	0.648775103	1.089883728	0.97763137
P52760	1.131876479	1.079102636	1.102601643	0.999674826	1.026167446	1.061136521	0.996792771	0.984058052	1.018645775	0.884353594	0.862298114	0.861714249
P70232	0.934613516	0.972941221	0.993433391	0.942085432	0.916257238	0.941061063	1.057396391	1.026422672	1.071381004	1.039170589	1.040560776	1.061307406
Q8BLE7	1.023203888	0.958980934	1.032250432	0.955917153	1.050730958	0.980516512	0.974169213	0.998570477	0.937774792	1.15157839	1.068346773	0.949649638

Q8K2K6	0.91716334	0.98449965	0.981904418	1.015395681	1.010895477	0.961235232	1.065482308	1.024869797	0.998287301	1.075247946	1.006741281	0.994564444
Q9Z2W8	0.933124535	0.919992859	0.953881139	1.042022053	0.993551832	1.020743761	1.026298928	0.96906021	0.965070465	0.977602819	1.028567868	1.189545672
Q62425	1.011884294	0.941478073	0.984203964	1.037536518	0.924021908	1.06709814	1.041819475	0.947049474	1.062150792	0.926715935	0.988964155	1.058636027
Q9DCS9	1.028958952	1.117537212	1.040892604	1.140701204	0.956455168	1.029023451	0.883687239	0.990392673	0.981990945	0.74176085	0.986834453	1.052013551
Q08460	0.929680786	1.016909994	0.954030433	0.994529864	0.965581346	0.921790298	0.981714592	0.999613889	1.005814403	1.067690559	1.127416741	1.056629539
Q91VC3	1.031019664	1.027433255	1.066934233	0.961687709	0.97007842	0.929722719	1.029291297	1.067251424	1.014177538	0.992039618	0.96771285	0.949030335
Q9CRC9	1.112353443	1.074841706	1.0520742	1.084152737	1.07281633	1.103936279	0.935051843	0.964765736	0.918199139	0.924549804	0.890253643	0.924772287
A6X935	1.218934675	1.408640841	1.139833294	0.785984712	0.73582879	0.768799398	1.162335595	1.123167781	1.169369379	0.819972406	0.795490235	0.784030899
Q99KQ4	0.964726551	0.95736693	0.97923914	0.974341288	1.000429622	0.99753781	1.04456189	1.026505637	1.045482139	1.017787382	1.004822837	0.998092066
Q9CZR8	0.967397581	0.974560294	1.009377018	1.017710628	1.007954973	1.050571321	0.98465173	1.018937389	0.984610995	0.965517777	0.993135354	1.038514346
Q61655	0.995263127	0.996028678	0.986114997	0.995548033	0.976362067	1.006019837	1.048757151	1.006877919	1.009039104	0.975955008	0.989508265	1.023533043
Q9D8E6	0.996716301	0.955633685	1.008056345	1.017346783	0.970851527	0.984585792	0.998304401	0.993876936	1.026901629	0.931521075	1.03502568	1.067146648
P47911	1.014337676	0.970133031	0.99221116	0.972811771	0.961019362	0.993465142	1.034460187	0.991292678	1.019330455	0.990495163	1.021574835	1.048351525
Q6WVG3	0.966314787	0.979717152	0.980140158	0.996052955	0.994498921	1.008817462	1.011612923	1.014779314	1.003992994	1.000044475	1.03279242	1.023524423
Q8BWS5	0.953419034	0.954793014	0.958189856	1.074847096	0.977799875	1.004769738	0.983894298	0.9762115	1.025378677	0.902722665	1.03978821	1.131326732
P62717	1.002847349	0.997811631	0.988533959	0.991384549	0.958946364	0.990236093	1.001754829	0.995927293	1.055222692	0.912527434	1.024134976	1.055996542
Q03141	0.898860247	0.945069113	1.012291488	1.040727827	1.025214811	1.024466948	0.993572826	1.003815503	1.002830472	1.02979917	1.006413004	1.044060043
Q8JZQ9	1.012762489	0.987542256	0.999190177	0.962184999	1.069911616	0.948300406	1.011722705	1.024298638	0.964092595	1.132402512	0.991369257	0.978173591
P28352	0.932800852	1.025225658	1.041555701	1.038455748	1.043185046	0.953642017	0.949212917	0.976949648	0.984158053	0.950709644	1.083498629	1.010662954
Q8C8N2	1.010241	1.022894458	1.003226656	0.980567709	0.98580172	0.943980213	1.055529092	1.010040866	1.008755859	1.03217271	0.996452932	0.97680313
Q9CXY6	1.003733401	0.991194863	1.002667228	0.97170077	1.009496933	1.00856747	1.030688084	0.968187906	0.998388872	1.061668482	0.997767957	1.003783745
P35438	1.034689858	1.023611898	1.003038086	0.927062534	0.973413339	0.931743293	1.078592736	1.015367058	1.038416583	1.047840433	0.990398207	0.959536056

Q61233	1.07076815	1.039272016	1.046499198	0.924398113	1.10961006	1.003889656	1.046815068	1.009989385	0.986422774	0.937421744	0.948774108	0.880945361
Q62376	0.973435597	0.95990469	0.995542473	1.0055584	1.058309037	1.065379477	1.002832703	1.02406335	0.989001828	0.961286434	0.98763668	0.988049002
Q9CQ19	1.279145051	0.971630906	1.006885609	0.94584972	0.874124092	1.053678453	1.062125733	1.04367905	0.984526408	1.086794592	0.889700581	0.896437745
Q9D0I9	0.983590958	0.960073062	0.972862129	0.994020434	1.027957315	1.010163973	1.02440979	1.003296432	1.011767259	0.996864651	1.010312378	1.022343613
P34914	0.888048322	0.929529284	0.975177177	1.011670143	0.97795439	1.06341541	1.002315792	1.002952734	0.997226459	1.045934133	1.030629161	1.104399384
Q91WK2	0.991773723	0.993146969	0.984780348	0.983686424	1.001464101	1.001854058	1.015982908	0.995788866	0.999034487	1.01723261	1.009476469	1.033941669
Q9JJY3	0.886889855	0.900447533	0.900359549	1.071573662	1.02270221	1.035233791	0.955252579	0.977234711	0.979498551	1.071134463	1.096404134	1.154102203
Q03517	0.991541563	1.011658489	1.201540967	0.983267498	0.95571359	1.066517441	0.948251761	1.027829377	0.975264141	0.914227709	0.943009747	0.966819089
Q91YJ2	0.951496798	0.98986824	1.067532074	1.025233036	1.021224517	0.937135081	0.965485734	1.035008357	1.016539923	0.858110864	1.034289674	1.046551494
Q6P5E4	0.962189942	0.93837617	0.955812909	0.966756979	1.057660818	0.973649046	1.023879504	1.022147875	1.013340641	1.050114877	1.055344399	1.005979282
Q9CQM9	0.981379733	0.975298143	0.970074894	0.993574647	0.988860927	1.006238472	1.001903456	1.02314817	1.015800854	1.024612034	1.012796782	1.033558052
P62242	1.023351575	0.964560083	0.988737062	0.971901345	0.947294942	0.972753619	1.059055309	1.045640343	1.026374164	1.020722853	0.973775371	1.028172857
Q922E4	1.002589753	0.931481204	0.965045237	1.022088802	0.995221875	0.998613963	0.990153788	0.971023573	0.983485306	1.0860339	1.036691948	1.082707661
Q80UP3	0.908793148	0.959035057	0.936290503	1.007164509	1.024925174	0.993925054	1.014467749	0.986598027	1.002589299	1.030488999	1.069564702	1.086540372
P10833	1.036246916	0.993271346	1.013967579	0.939217112	1.024118004	0.986388998	1.010901511	1.018292645	0.985415839	1.084361345	1.00684935	0.955330772
O35250	1.021125267	1.010664729	0.952312369	0.971496155	1.039201553	1.019516208	0.974616694	0.986631687	1.036417545	1.10931209	0.98304655	0.971691942
Q61735	1.033016197	1.045865127	1.017950481	1.009985928	1.047239733	1.031045449	1.027060137	0.970625555	0.975483326	1.020677462	0.93387597	0.940632246
Q8C0E2	0.957149482	0.975477809	0.996538271	0.958324793	1.030087564	1.006275065	1.030557786	0.966526549	1.0136928	1.126586319	0.994208861	1.01204228
Q8VEH3	1.039395419	1.070866805	1.025301113	1.034229107	0.956943912	0.922706795	0.96225109	1.06387119	1.060860052	0.899090717	0.97017926	0.973381762
Q921F4	0.996249119	1.005203703	0.995225604	0.959185152	0.960953207	0.93825707	1.039550375	1.035004399	1.136678053	0.992737213	0.972982429	0.957329128
Q9DB72	1.199791922	1.148525452	1.045423431	0.912028669	0.864309682	0.87959772	1.048354359	1.042163311	1.102807145	0.826575566	0.932531285	0.943321289
P47791	1.054498392	1.009897769	1.026312583	1.040515444	1.050334485	1.053039063	0.970393447	0.96559215	0.944515762	0.973458742	0.994538947	0.955934098

Q6P542	0.98619841	0.960454842	0.982012791	1.013799843	0.985321023	1.021299407	1.00549935	0.964117496	0.974939924	1.034547274	1.04596985	1.063938947
P15327	1.122187411	1.099580253	1.091094455	0.815828504	0.865746016	0.827319427	1.068694771	1.18364798	1.347443147	0.859899779	0.824650082	0.792899011
Q9ERS2	1.049427087	0.999412176	1.056406446	1.066052155	1.054248188	1.072261879	0.951165558	1.008458356	0.955363458	0.92001441	0.936796534	0.954896441
P63028	0.928359099	0.999694119	1.041432169	0.994721819	1.139346614	0.938640894	0.937892603	1.030486385	1.00248327	0.891980585	1.066204291	0.991134749
Q61166	0.872459215	0.97648552	0.947431011	0.978296765	0.975040703	1.023144617	1.064107975	1.033928711	1.05357537	0.976780266	1.046629022	1.026314152
Q9ERU9	0.967925397	0.963208922	1.019976572	1.04608363	1.002404923	0.981423316	0.962603865	0.987921331	1.005280991	0.917462736	1.041357337	1.091077794
Q9DBG6	1.039079542	0.959210667	1.008425443	0.95478565	0.995904274	0.983443127	1.026034387	1.01062925	0.970561931	1.077507159	1.016479107	1.011245155
Q8BNW9	0.960549209	1.041490695	1.028614641	1.043117278	1.05634173	0.990186545	0.98205309	0.989591985	1.014756736	0.907509827	0.989477051	0.983258667
P59279	0.899284664	0.95094011	0.988952101	1.050442631	1.090700527	0.942461803	0.93107508	0.94721277	0.90885782	1.185145789	1.117593872	1.088197424
Q6P5F9	1.022224744	1.015000102	0.99387796	0.991328521	0.922898257	0.980376092	1.018226374	1.023200626	1.094741166	0.914546702	0.968912407	1.030599584
Q61036	0.986783169	0.954771846	1.045668292	1.004967507	0.963966368	1.008467957	0.990900195	1.026792914	1.003753449	1.031701893	0.984519455	1.029644736
P42125	1.064718015	1.039769094	1.031411014	1.003348992	0.943136517	0.979016124	1.015897747	1.010106617	1.032887472	0.908600431	0.973520703	0.983886324
P29387	1.004821678	1.019835524	1.030191507	1.01396962	1.211630437	1.022355188	0.904117651	0.999794467	0.865560937	0.992444254	1.047452829	0.938756748
Q80TL0	1.059084343	0.998894084	0.999735174	0.973976213	0.921298558	0.925240375	1.059755887	1.027896106	1.035065094	0.969549791	1.010323552	1.014696168
Q8C7R4	0.995770127	0.967416711	0.951823407	0.978499355	1.012805014	1.00906323	1.01567841	0.994656795	0.97019927	1.122275141	1.036444536	1.01883498
Q9QZ06	0.987015288	0.995286399	0.993087545	1.034161262	0.969753733	1.005168757	0.99400168	0.991772112	1.036049927	0.942366594	0.996803965	1.053423102
P15116	0.979362861	0.970579312	1.007990039	0.967224122	1.00160666	0.99095051	1.025143557	1.038399827	1.004830973	1.031035769	0.994860343	1.013102818
Q8BPM0	0.99031697	0.989732757	1.000241521	1.021287448	1.024603265	1.034881654	0.99796272	0.963884641	1.019318685	0.995206682	0.989454454	0.999688883
Q3UHX2	1.011710431	1.041786103	1.017649292	1.011034798	1.060442642	0.986001359	1.021462494	1.001676342	0.964120173	1.028633043	0.933801855	0.976390178
Q9CQR4	1.091594086	1.053489006	1.056720799	1.068748096	1.147736846	1.117562605	0.936549288	0.907488275	0.881637363	0.97807362	0.92891714	0.912925672
P54728	1.013906157	0.977047752	0.995041185	1.002457194	1.040667883	1.072119534	1.027336018	0.952026696	1.048934987	0.942053294	0.954361447	0.981608729
P24270	1.018486528	1.02452088	1.025986587	0.966950849	0.959017361	0.911406468	1.003481781	1.051488794	1.09086944	0.955642832	1.000196507	0.971281903

Q6ZWX6	1.014286202	0.996081059	1.003439334	0.992713057	0.989371486	0.964590323	1.014000638	1.022872936	0.996910521	0.987499135	1.010051966	1.022451647
Q62077	0.959210603	0.958816771	0.980710971	0.988323205	0.967003852	0.992741779	1.006588235	1.034400795	1.01239858	1.044026386	1.037744423	1.042117296
Q80ZI6	1.009508439	1.057099592	1.054562078	1.016471645	0.954241728	0.98211358	0.972768577	0.996891443	1.05381219	0.871568248	0.991625238	1.002959763
Q64737	1.102476203	0.999859853	1.018475418	1.031528171	0.950213802	1.066840432	1.021027356	0.990227648	1.009741894	0.844269488	0.980163586	0.957921619
P47962	0.990258329	0.97769993	0.998915247	1.030111841	0.947747241	1.01256702	1.03335454	1.013514343	0.999059889	0.95556927	1.001898967	1.040517961
Q8R4N0	1.072372774	1.0364263	1.036461681	0.994294405	0.998071268	0.98510041	0.982863366	0.993089569	1.029577954	0.931850159	0.963055768	0.982578867
Q8BLF1	1.055360682	1.067820755	0.962466604	0.983086677	0.964094623	0.989256605	1.038936589	1.014891934	1.046191227	0.969661452	0.944949288	0.979755869
P47955	1.003666698	1.023700622	1.020486103	1.070043433	1.037658611	0.871850218	1.031229357	0.961462512	1.07067295	0.732090939	1.043146221	1.03292378
Q9JL62	1.153261401	1.199602391	1.019479631	1.092604453	1.059203381	1.156835796	0.879877655	0.944088592	0.94799275	0.807692285	0.881468725	0.881695998
P60229	1.047140675	1.001414853	0.984662693	0.955035137	0.947157245	1.01066777	1.049862457	1.008240847	1.031740215	1.015751098	0.974384244	0.999670348
Q6P1B1	0.951184455	0.978758265	0.946515523	1.007521341	0.979661976	0.992867076	1.022315335	1.01343096	1.003696465	1.087241779	1.02755501	1.038642213
Q8R1B4	1.027545755	1.026224058	1.009998311	1.015465144	1.027310459	1.003881189	0.98739044	1.002338244	0.974352254	0.981550578	1.001968378	0.967614935
Q60737	0.966238288	0.999682546	0.994896133	0.998176685	1.066748735	0.976998812	1.00909249	1.049041482	0.953370139	1.072787936	1.001856106	0.964261442
Q80VD1	1.040034575	1.051261867	1.034976492	1.019334652	0.993211471	1.0039859	0.981700048	0.996793468	0.965310252	0.938309276	0.998221779	0.987080683
Q9R0N5	1.224666271	0.954865435	0.883453768	0.828397375	0.954533577	1.16137245	1.242189055	0.987671928	0.932248456	1.199444767	0.850002944	0.924718453
P62774	0.95172566	1.002563702	0.97105734	1.046931174	1.040381259	0.998685481	1.049491508	0.954183665	1.01380066	0.846834163	1.067589893	1.003499406
Q6ZQ82	0.992449803	1.014045762	0.990765769	1.004203261	1.013078182	1.010271109	0.971918675	0.948068847	0.984313471	1.071959033	1.015117375	1.046921023
Q62465	1.128795647	1.034841899	1.152003337	1.050504444	0.990482368	1.158457482	0.921006595	0.983724621	0.961402638	0.887497947	0.847866666	0.914662361
Q68FL6	0.980525381	0.990982571	0.951821855	0.995378488	0.953441226	0.994869073	1.03967562	0.995369569	1.011990101	1.041603713	1.014532596	1.06339737
Q91Z61	1.010884872	0.993933636	1.019322227	1.017633445	1.061366572	1.048960953	0.969689118	0.979831167	0.92893896	1.05970423	0.993810866	0.986342989
P46735	1.017904942	1.001823302	0.97866815	0.953523718	0.883820477	0.888355895	1.075472287	1.126582068	1.102480617	0.933351649	0.986195098	1.008929193
O70439	0.963653404	1.020120149	0.994694694	1.003437932	1.040481961	1.005000195	0.981218117	0.995566585	1.00151155	0.959700538	1.021808935	1.01723665

O88952	0.958955799	0.965147729	1.079573789	1.068783475	1.048539147	1.005254907	0.92350628	0.984523666	1.043070516	0.780845347	1.037329584	1.030769716
Q9JIX8	0.936615601	0.959968536	1.018085545	0.985497777	1.022638423	0.999548935	1.009474438	0.992219196	1.021658252	1.036144282	1.02622395	1.012129562
Q91X97	0.986364797	1.024688312	1.017199709	1.003166505	0.970858644	0.968969805	0.995403087	0.977756458	1.028506207	0.918010479	1.029176912	1.059730331
Q99L04	1.112032233	1.030942236	1.066878378	1.005023491	1.020779915	0.961757726	0.978741642	0.984686156	1.038843156	0.87841228	0.950571148	0.960118149
Q569Z6	1.013343359	1.009391436	1.034923312	0.95783621	1.015110075	0.978157195	1.012370508	1.039384206	1.00536426	1.024204309	0.980625024	0.955438769
Q9CQI3	0.914232417	0.930330186	0.946786108	0.934363782	0.926623068	0.95992594	0.999664366	1.090618192	1.164137601	1.453753326	0.915635539	0.937819611
Q61646	1.459866725	1.627544659	1.103847535	0.735858468	0.620663085	0.809829428	1.317848237	1.103530969	1.251913231	0.65505922	0.580957454	0.612819037
Q8VCI5	1.028782735	1.055195195	1.012619561	1.016359086	0.944532112	0.961736824	1.013373989	1.030355235	1.056878293	0.867210742	0.967904767	1.009196015
Q8C754	1.053128899	1.043761454	1.025259165	0.999883278	1.12925399	1.016237028	0.912101213	0.993142118	0.88628075	1.104281506	1.010036905	0.928232021
O35381	0.865218609	0.954965974	1.030426028	1.066377928	1.143344649	1.005523619	0.953413482	1.007258802	1.039496201	0.818948444	1.014282606	1.039586486
Q9DC70	1.050517801	0.991826073	0.992214615	1.000082252	1.020022971	1.044812161	1.024153862	1.009244476	0.982720223	1.018708452	0.946968786	0.967887019
Q9Z329	0.702741436	0.898559697	0.994558693	0.940547177	0.997846576	0.94931209	1.241535712	1.090909103	1.070076932	1.326873437	0.960121798	0.900344067
P35922	1.122429734	1.086500205	1.134681953	0.826759415	0.825269679	0.821677274	1.171416685	1.113211076	1.161271205	0.928212447	0.886558108	0.858320932
Q9D4H8	0.992699851	0.995315777	0.997019521	1.006513105	1.000108579	0.977808021	0.988712452	1.010963619	0.995576866	1.004171127	1.021205684	1.03290369
Q9DC51	0.958451924	1.049862032	1.030550344	1.035927021	0.966076706	1.02862557	0.934979274	0.960765924	1.020556964	0.88715223	1.031344045	1.071585112
O88958	0.984455602	0.930912062	1.004187804	0.931464519	0.949773888	1.004281424	1.030501462	1.028885805	1.055382935	1.016246605	0.99978775	1.071985375
Q3UH66	0.939762004	0.994860278	0.972540597	1.014550863	1.025199879	0.978256749	1.004326194	0.982576196	1.001263364	1.050240223	1.039788611	1.031944813
Q8R001	0.997493574	1.013814072	1.020852699	1.007266685	1.051880088	1.056468284	0.970925189	0.960713623	0.987823183	0.96543624	0.991704009	0.997704573
Q56A07	0.952593533	1.029524062	1.026246804	1.028543357	1.070014241	0.993395555	0.96693822	0.977884251	0.94030312	1.007920575	1.052207289	0.983277818
Q8R151	1.061896872	1.007202404	1.008367919	1.019184087	0.93927751	0.959555854	1.035311487	1.053372866	0.977929601	1.010285899	0.985193836	0.972645932
O88704	0.985523315	0.970794135	0.983808918	0.992022963	0.959448717	0.986871092	1.026646987	1.014976787	1.019918451	1.060332823	1.020768045	1.013140765
Q99L43	0.95392224	1.040182699	1.095557627	0.9968011	1.14521423	1.024205072	0.889412423	1.002018891	0.836816018	1.047561378	1.078235262	0.947514119

P54823	0.951259654	0.936656464	0.996165695	0.986968823	0.990104339	1.008896173	1.04481106	1.009393522	1.001678332	1.055012462	1.012187296	1.03870335
Q9JKK1	1.049044334	1.0453917	0.997953369	0.98210732	0.960293377	1.01943766	1.063385947	1.018688761	1.010490688	1.032429495	0.911203282	0.956527292
Q8BL65	0.912232732	0.936094881	0.993921088	0.992464254	0.979097324	0.887507958	0.972493189	1.076309741	1.101369236	0.958146617	1.067019673	1.085243821
Q9QZF2	0.950121138	1.002393233	0.971646745	0.980044124	0.988695867	1.001421933	1.025345059	1.053468285	0.994714331	1.014719471	1.014975039	1.018037356
Q61147	1.183132082	1.237917144	1.126715656	0.871009639	0.866925156	0.86435321	1.137638316	1.035555228	1.178999064	0.782883377	0.810820021	0.817972908
P04627	0.917642028	0.992035132	1.002234088	0.979330041	1.043932878	0.988125227	0.937106747	1.030730263	0.993858394	1.092068691	1.056485456	1.012269674
Q80X80	0.907357986	0.972359346	0.990713128	1.056527144	1.033593754	0.981627351	0.949310809	1.015349021	1.011086996	0.951232386	1.048254145	1.07695072
Q9WVA4	1.036393969	1.042817133	1.033184318	0.980975588	0.960169285	0.995961856	1.014831605	0.966111108	0.980964015	0.929315289	1.029680956	1.022491973
O08810	0.97467123	1.026650532	0.959750988	1.011939217	0.988423707	0.985190312	1.028738633	1.01285559	1.000491154	1.064065948	0.993078832	1.002233349
P67871	0.966771285	0.981879468	1.005518377	1.030293172	1.021463562	0.994732527	0.980912564	1.001620008	1.046964829	0.916943187	1.014419989	1.019318193
P54822	1.073153357	1.000210076	1.05977671	1.001970533	1.042251408	0.982420246	0.989771537	1.038135304	0.972351638	0.92059813	0.992740697	0.924888034
Q9QYG0	1.05376662	1.005449626	1.050604257	1.032791028	1.019529459	1.073816033	0.945999511	0.97347235	0.992131624	0.901297549	0.955098696	1.005541358
Q6PER3	1.009364613	1.00337885	1.011296527	1.005121569	1.100552735	1.01668149	0.968899962	0.999618594	0.926710036	0.989578475	1.033333316	0.968341182
P97494	0.99604042	1.004533958	1.005417617	1.031616138	0.949055141	0.988764499	0.98299653	1.023542292	1.061809165	0.911137571	0.982405211	1.044341116
Q78JE5	1.014342517	1.03026806	1.035555485	0.993902614	0.980060941	0.94300377	1.03883629	1.01770827	1.044784565	0.93438759	0.987650937	0.961138254
Q8BYM5	0.977912432	0.985049442	1.012184925	0.867594834	1.005023425	0.914209534	1.09718653	1.113229397	1.093057971	1.148569777	0.938928384	0.88871451
Q9CQI6	1.009371119	1.017701232	1.055659971	1.047779596	1.036145861	1.057137896	0.959458526	0.994019836	0.953051401	0.926875469	0.976252174	0.980289513
P32848	1.663472282	1.20397352	1.034027783	0.909722946	1.117366177	1.043735254	0.960623081	0.837687229	1.011144427	0.780909225	0.775372924	0.727902826
Q9D415	0.995940824	0.962381681	0.993563424	0.964901755	0.967252995	1.002821169	1.059066543	0.979181854	0.978633258	1.040699835	1.069311895	1.007766456
P27671	1.022862537	0.993329499	1.020629379	0.969172948	1.012007955	1.059868643	0.963319175	1.012568441	0.995925894	0.935883317	1.005572736	1.009956368
Q9D2M8	0.987750825	0.946144666	0.846585329	0.934341017	0.928878732	0.968798562	0.987806567	0.947321636	1.04136601	2.261443314	0.835081394	0.883583457
P21981	1.243815666	1.038107064	1.051130693	0.94140357	0.944136214	1.00230716	0.963690234	0.991436182	1.033782479	0.922589081	0.957246753	0.926862962

Q8R164	0.991737272	1.003772238	1.045270521	1.072090006	1.033411375	0.997582617	0.948942584	0.968886703	1.011691349	0.882328961	1.011088361	1.016021933
A2AJA9	0.977951282	0.941835339	0.946677796	0.974862863	0.973801597	0.989548424	1.051918771	0.982260997	1.007448934	1.070941682	1.052804013	1.067477724
P40237	1.192703287	1.152422854	1.079990894	1.162538518	1.27674373	1.119608985	0.791825644	0.859392064	0.825121326	0.847474222	0.916989202	0.857460017
Q6ZQI3	1.078214659	0.990439087	1.016488303	0.971281759	1.014261713	0.978932532	0.988587861	1.010061246	0.955088558	1.120269223	0.980013438	0.986533652
P10922	1.296401249	1.094396669	1.094028535	1.072306027	1.117564213	1.208523279	0.906462826	0.869051949	0.913852547	0.861225141	0.80654403	0.83975526
Q99KV1	1.0049319	1.059141558	1.056821734	0.993340772	0.924390178	0.854200383	1.012222887	1.058721855	1.088286206	0.846069902	1.028312345	0.997462758
Q8CHT0	1.075028364	0.986685509	1.008569707	1.006913626	0.988659438	1.080389608	0.986872795	0.999643679	1.003782917	0.926453469	0.957700756	0.992497522
Q9D6J5	0.966234603	1.048352856	1.024387243	1.082483862	0.953802407	1.117554652	0.998752856	1.019852579	0.985167881	0.906976406	0.939213613	0.955862668
Q99MR6	0.957184012	0.984524515	0.993124874	0.98643536	0.981109026	0.984152534	1.048954163	1.024605522	1.020997932	1.00586349	0.999385698	1.023036159
A2AG50	1.025489887	1.031250838	0.979333733	1.038606676	0.987726413	1.055532182	1.008151112	0.955210487	1.023113178	0.985403862	0.938389006	1.010544886
Q6PDY2	1.022397818	0.977444377	0.984509499	0.997237379	0.991132375	1.039060079	1.004366113	0.986938317	1.008518687	0.976301057	0.993859817	1.038537796
O88696	1.026623877	1.01101297	1.062572563	1.05466218	1.015999355	1.097914211	0.979440094	0.976186686	0.976931536	0.912080254	0.937873589	0.96081144
P17563	1.124132502	1.051918306	1.045146001	1.052169705	1.041920852	1.059718604	0.962350673	0.964716564	0.953041915	0.955414309	0.922084337	0.919678737
P01831	0.899627821	0.94287903	0.950200062	0.992649601	0.981339834	1.020737682	1.072257163	0.990422084	1.029052034	0.987387811	1.019086025	1.111968083
Q9R1R2	1.02559186	1.018948165	0.994435844	0.984904159	0.935692255	1.008158891	1.029876169	1.044910225	1.048818929	0.96720254	0.953088322	0.99303225
P29391	1.065781176	1.022600931	1.111966834	1.062443009	1.093635491	1.10470863	0.928832758	0.925487442	0.903010902	0.971035917	0.952171492	0.916707748
O88441	1.075368084	0.967848869	0.972206983	1.023115338	0.983430962	1.065826806	1.010408266	0.96934115	1.044702338	0.96755801	0.956036001	0.991678104
E9Q7X7	0.973580331	0.983671618	1.016881387	0.997875691	0.989652262	1.015236364	1.014168193	1.022195779	1.002669402	1.026666237	1.00017478	0.982461156
Q9CRD2	0.998784185	1.004967215	0.971574985	0.957388238	0.989035346	1.003113861	1.050101244	1.020963873	1.020317011	1.018150715	0.996810355	0.988594701
P61087	0.955552985	0.992434286	0.985025075	1.021838984	1.154965921	1.005535264	0.972152856	0.98408491	0.913336328	1.033401645	1.049633413	0.982273164
Q8BHL5	1.003302762	0.990503557	1.000094063	0.985469137	1.014983745	1.027126436	0.998044701	1.020112004	1.019726894	1.06333694	0.94359903	0.990131087
Q80VP1	0.994072737	1.0073388	1.024849947	1.033780725	1.006149893	1.079928001	1.011709322	0.994253748	0.993366929	0.967685156	0.922435309	0.992406606

O35954	1.001244499	1.044408225	1.019085909	1.035571291	1.023407382	1.003784923	0.935267184	1.008545064	0.988282025	0.942506821	1.0178127	0.987823575
P61021	1.008671481	0.963950769	1.017507866	0.983423704	0.966919308	0.942759328	0.988324969	1.012237426	0.974910638	1.214164107	1.008370491	1.02880853
P51855	1.070652656	0.982191221	1.012940911	0.986955359	0.988618819	1.037978893	1.007987161	1.004929852	1.040155839	0.980745043	0.938191221	0.975138563
O35678	0.97087239	1.011641155	0.980496569	0.973135195	0.986359958	0.973741155	1.054181074	1.037238225	1.009700577	0.987673422	1.018123043	0.996425276
P62082	0.921019507	0.937987841	0.970557184	0.975859446	0.99004565	0.969478649	1.070788382	1.092262045	1.009735862	0.992233527	1.028911784	1.029404589
Q60973	0.912118511	0.952048168	0.997079045	1.026365684	1.029715222	1.003502738	0.962123292	1.01559188	0.988785206	1.034881643	1.073935453	1.025545295
P51880	0.95418886	0.853973501	1.015838323	0.837018515	0.785106381	0.844846233	1.105309704	1.205705817	1.182539304	1.154013647	1.000481381	1.056881059
Q5DTT2	0.870804386	0.968281233	0.95260179	1.06321061	1.004352283	0.9434762	1.013274025	1.029681248	1.013529593	1.001272167	1.086993571	1.047311195
Q80VC9	0.966259493	1.003560309	0.983899683	0.994082054	0.967154791	1.002447026	0.9810991	1.004969157	0.982283787	1.014511852	1.052690499	1.068811193
Q8QZY1	0.982751476	1.030758101	0.996160881	0.971972084	0.960229115	0.961186666	1.059543428	1.018113637	1.025673038	1.032148874	0.955683727	1.03415499
P70414	1.09124811	0.910651226	0.951715819	0.962456259	0.983256496	0.969781612	1.054365458	0.965769321	1.037253567	1.014275481	1.047935312	1.034150785
A2ASZ8	1.022788444	0.974054326	0.997110145	0.991597607	0.965606698	1.017929115	0.987383873	0.971586011	0.979213118	1.305809759	0.957067992	0.995084736
Q91YP2	0.987793287	0.997027208	1.00418098	1.006035446	1.044994762	1.00001002	1.000342432	0.992372801	0.975440386	1.012589441	1.00795053	1.004135217
Q9D7A8	0.99311025	0.984873768	1.006845194	1.024126644	0.994016152	1.035582303	0.964032653	0.988957818	0.990892785	0.901099778	1.047143251	1.052650643
Q9EQK5	1.156894089	1.193462863	1.059776716	1.007322461	1.042491583	1.012734665	0.963712349	1.005667927	0.970048734	0.851375116	0.898068976	0.84360119
Q6PF93	0.970482298	0.967960495	0.992827246	0.982075259	0.976722628	0.99615145	1.031940658	1.002773309	1.040712691	1.022733429	1.006746992	1.025564044
Q6PDL0	0.943385866	1.046558723	0.965963145	1.040529977	1.018009047	1.040518524	1.021495487	0.919412519	0.996574725	1.022125808	0.986841037	1.04201539
Q9D164	0.912194151	1.011034685	1.087050193	0.979801403	0.925479725	0.937980564	1.011200122	1.097719265	0.991084046	0.946772435	1.025386436	1.03996854
Q9CR26	0.95722704	1.012470265	1.028509628	1.083373025	0.982252165	1.037431015	0.945675964	1.008148783	1.003750348	0.862903357	0.980073142	1.07629359
Q5FWK3	0.987074439	0.969202261	0.989901591	0.977944895	1.048733546	1.053502768	1.006477872	0.998050094	1.003356206	0.980024354	0.998164478	1.003527436
Q8BWY3	1.019712686	0.9789082	1.016853592	1.007683626	1.076503779	0.91844283	0.941507784	1.007025183	0.997518242	0.875672185	1.087303361	1.039444141
Q8BH57	0.97906814	1.022013353	1.014328379	1.01445056	0.973557787	0.984155267	0.988341235	1.018189987	1.003879606	0.938263647	1.026163288	1.027224085

Q9ESK9	0.950760953	1.002968773	0.99726869	0.954163599	1.001613796	0.99831669	1.019531726	1.004489973	1.009393919	1.038771498	1.019383471	1.026815384
Q8K411	0.99090381	0.950452414	0.977263271	1.014590959	1.015600642	1.01725595	1.006235436	1.004862709	0.991630491	1.037076165	1.015892436	1.017814332
P97390	1.006548889	0.979740748	1.014451497	1.012009208	0.997824261	1.024303939	0.996941745	1.000347936	0.962783113	1.045084824	1.001276908	1.008141588
O89084	0.946546382	0.941556619	0.964325518	1.013851008	1.026806512	0.991405392	1.021207424	0.987347068	1.002620303	1.010930252	1.033106229	1.081430328
Q8BWR2	1.017923985	0.982353148	1.021618139	1.011461666	1.022531023	1.05774807	0.979611393	0.959405602	0.962501822	1.053079064	0.982425389	1.013969824
O70305	0.961847493	0.95516481	0.957807743	0.993125686	1.036411167	1.002567372	1.018569958	0.987271367	0.972126857	1.200332059	1.008602406	1.014029211
P07309	1.330010223	1.281464086	1.344462301	0.798003857	0.769977388	0.752776316	1.151511767	1.155979961	1.157196247	0.734610045	0.726091145	0.682491172
P62482	1.080113333	0.99929846	1.004760196	1.000610473	1.012488336	0.98614913	1.011075578	0.966428681	1.011852752	0.970418406	0.975095066	1.008293239
Q9Z2C4	0.961249218	0.940712802	0.992102682	1.008227064	0.999565618	1.015771911	1.022520101	0.96649634	1.00369116	0.976503481	1.036821766	1.082375299
Q4V9Z5	1.003677869	0.991143681	1.01751915	1.003591449	1.03546402	0.938435046	0.953687588	0.970045594	1.012957839	0.963833337	1.055545613	1.059196585
Q6PE13	0.961292512	0.962853641	0.985426163	1.012503843	1.006528624	0.984589116	1.023072582	1.000611455	0.985753397	1.048980806	1.026637284	1.038613178
Q6PDQ2	1.007518592	0.964963187	0.981314024	0.97019817	0.991088079	0.994902918	1.070241218	1.002009615	1.038661486	0.989748485	0.976562684	1.023319983
P59325	0.982752687	1.063636032	1.024214012	1.033162894	0.968764909	0.95653747	0.97777711	1.031645046	1.009192497	0.869528714	1.026711665	1.016978235
O35286	0.964604546	1.016627599	0.989563371	1.015389983	0.980765858	0.972635548	1.000340078	1.005483229	1.001199206	0.998813279	1.030102455	1.039136082
Q9QXB9	1.086801291	1.019772193	1.008893679	1.006251513	0.986023384	1.051609769	0.972031309	0.978815772	0.990970347	0.992348932	0.944335466	1.011826678
Q62448	0.937616564	0.939226071	0.962284927	0.976405239	1.010287652	1.016037625	1.075836829	0.939125999	1.040675723	0.998049283	1.018723884	1.092677622
P22315	0.996561569	0.994860549	1.005020812	1.031443785	1.043824029	1.059003281	0.995150636	0.969200241	0.970696705	0.973675913	0.99587049	0.991222011
Q9DAK9	0.998284242	0.981253352	1.042816845	1.064360748	1.074580227	1.090618243	0.968165627	0.974653061	0.951433396	0.99369004	0.962927627	0.943840835
P14576	1.01485713	0.963226813	0.987343886	0.989200991	1.022889444	1.003882047	1.040198612	0.992964048	0.94074751	1.035896215	1.024679166	1.027420499
P61028	1.017977866	1.004174618	1.056659647	0.974160433	1.04000903	1.009245313	1.006321315	1.001937543	0.955419729	1.001511944	0.975816865	0.989241978
O70166	1.000452742	1.018501532	1.021265764	0.973419307	0.940119657	0.99965707	1.079750265	0.990417169	1.026484881	0.966011555	0.974704384	1.00584939
Q8BFU3	0.965215603	1.004014582	0.995796695	1.017903441	0.991322656	0.968460634	0.968083375	1.004917347	0.99185921	0.965151831	1.070619841	1.055217432

B9EJ86	0.977351956	0.926478328	0.952576763	0.997168343	1.024542814	1.016959547	1.050283842	1.006367535	0.923374102	1.173981874	1.031115018	1.017568995
Q8K012	0.930288264	0.990407743	0.962258934	0.997038741	1.004227522	0.97647518	1.015080318	1.04789493	1.014988175	1.027793338	1.04260595	1.004074844
Q99LD8	1.017248055	0.984561276	1.016289837	1.0050363	0.987645515	0.985124778	1.029163198	1.013164631	1.01037657	0.966167758	0.983937627	1.009009041
Q9Z1Q2	1.010727336	0.973489277	0.990274706	0.988137064	0.982349913	1.034342098	1.024262487	0.999044402	1.015707224	1.012031508	1.001873889	0.991969763
Q69ZK9	0.929912035	0.976512086	1.006018788	0.957270179	0.942458285	0.933936086	1.035883177	1.063359939	1.046587892	1.088069582	1.028210268	1.012974558
Q9QZE7	1.10170297	1.065203659	1.057718528	1.021633825	1.156502713	1.056714033	0.898937861	0.93466577	0.931580206	1.00404211	0.952150452	0.89644061
P29533	0.938043043	0.97818175	0.936176727	0.966164813	0.942478053	0.939256819	1.053750778	1.023546021	1.035241515	1.038473958	1.063207062	1.093521741
Q80T41	1.027646657	1.00839734	1.000859836	0.965288789	0.926724406	0.971836021	1.041618547	0.976908674	1.036087137	1.029264893	1.009784308	1.028888941
P51660	1.047338741	1.014103537	1.026812532	0.99094083	1.028625213	1.008462942	0.981986246	0.988175839	0.963116387	1.008557263	1.004920314	0.975951364
P35979	0.974416231	0.957049239	1.002537349	0.980203617	0.991470151	1.014671157	1.017995314	1.006320887	0.991067352	1.016361985	1.013205278	1.058244859
Q7M750	0.958150673	1.000781759	0.99139307	0.989450205	1.111628064	1.042158769	0.950016004	0.9593557	1.061783092	0.935870441	0.991399968	1.007561319
Q3UHC7	0.862809508	0.95386812	0.985242959	1.12484601	1.109446054	0.906587797	0.914697969	0.935193631	1.017325501	0.871066747	1.208986677	1.050638593
Q9D7P6	0.997801645	0.968169118	1.02287157	1.02207631	0.968357692	1.025116136	0.996050156	1.040312804	0.974080383	1.00680119	0.986811298	1.021116175
O54946	1.001433237	1.047722102	1.051684687	1.104375602	1.0373981	0.976869441	0.973658463	0.906643951	0.917209733	1.07597691	1.002101212	0.988618605
P24369	1.014899503	0.965540997	0.991720355	0.982814881	1.053338583	1.036209569	0.964808299	0.990232518	0.930636692	1.162787958	1.036854068	0.97241582
Q9EP89	1.006939068	1.006467511	0.976885657	0.99099196	1.04227415	1.027915006	1.021444166	0.983334261	1.004432636	1.022977992	0.963882852	0.995968746
Q9DB41	1.142211304	1.054839495	1.09183369	1.048893241	1.032984907	1.006925587	0.895992307	0.960601249	0.990851799	0.823057834	0.968318169	0.972183568
Q9WUM5	1.05761684	0.958028182	1.020992832	1.060085152	1.031377902	1.09256914	0.970624237	0.976751189	0.970863958	0.915885815	0.959864349	1.006129021
Q9CQ65	1.084451959	1.067581118	1.025645419	0.979260915	1.018510327	0.964423115	0.998717574	1.000940459	0.991297859	0.982315317	0.96843074	0.948322022
Q91Z31	1.052642013	1.014617916	1.073723609	1.006710395	0.948735885	0.997348127	1.026171819	1.080245571	0.991702864	0.898567648	0.947128924	0.943459743
D3YZP9	1.074230545	1.089402692	1.079912308	1.061858702	1.009830632	0.952609634	0.99113706	0.992104201	0.98092461	0.87576329	0.940163252	0.947194718
Q8VBW6	1.020994947	0.996023644	0.982603056	0.994562764	1.021668332	1.032550145	0.987744998	0.996946496	0.979452685	1.015289385	1.018636904	0.990111598

Q80VL1	0.982165263	1.276360533	0.949371594	1.011370429	0.938377846	0.939938533	0.99995794	1.01077239	0.977488458	0.965251816	0.990175853	0.974014996
Q8R5J9	0.961852246	0.944448313	0.96789834	0.979732192	0.974901601	1.029054263	1.046470174	1.015138023	1.011784766	1.02064374	1.012189704	1.055064808
Q11136	1.004752542	1.004123511	0.987445554	1.008590369	0.952397549	0.970841223	0.967736054	0.980040164	1.049617657	0.9663292	1.031343041	1.080432965
P16045	1.023112222	1.078619573	1.00092545	1.001508811	1.022240914	0.975361705	1.015976974	0.955624665	0.941960089	1.006456078	1.029617102	0.98427023
Q922D4	1.012900547	1.055108751	0.991014006	0.994350445	0.928463857	0.984996904	0.970365991	0.960551281	0.968374836	0.999520688	1.095399255	1.056447929
Q80WC7	0.952247265	1.034837072	1.025691648	1.085982947	1.011176061	0.935646907	0.909872725	1.016659001	0.988356432	0.954831546	1.053166659	1.036183687
P58389	1.083761402	1.0502649	1.05557815	1.047114745	1.039487379	1.032131218	0.955304452	0.981164463	0.967401666	0.918387433	0.939003795	0.955435138
Q99K70	0.99205055	0.97597356	0.975162316	0.983810764	0.948473325	1.049155108	1.105588932	1.029290379	0.949622083	1.165139026	0.95761188	0.959092525
A2A432	1.023987272	0.98041218	0.991335205	0.993432583	0.978628651	1.015273183	1.004265373	1.001463444	0.996098804	0.990184298	1.002613068	1.045774209
P34022	0.997324498	0.986266304	1.014551242	1.050515578	0.913371223	1.070494334	0.991972554	1.024659049	1.016508477	0.842427756	0.974742564	1.080610627
Q9D4H1	1.003637546	0.961652711	0.969914652	0.99874464	0.953546884	1.03152257	1.035088336	0.986291735	0.994755007	1.095459424	0.996149809	1.03767987
Q791T5	1.04097321	1.013004311	1.017838745	1.052352239	0.993021957	0.994793124	0.951247008	1.015610436	0.964793351	0.946387545	1.006804577	1.021675043
Q9D8U8	0.990977707	0.983273971	1.003446567	1.029258938	0.962383941	1.025022464	0.989089887	1.032966739	0.98995579	0.963958508	1.012297121	1.0247362
O70228	1.012102812	0.974453087	1.01837792	1.026397038	0.971393795	1.004225427	0.97949052	0.98397591	0.985258121	1.008838019	1.006524009	1.063889607
O88322	1.248389151	1.05957838	1.0087771	1.044167921	1.037945735	1.12058372	0.931604753	0.94816324	0.91815211	1.052062963	0.856770268	0.907591989
Q9DD18	1.046627447	1.035475931	1.069285828	1.002745579	1.098619746	1.067409541	0.92254166	0.975786934	0.906585224	1.068943451	0.950765531	0.946899835
O35658	1.016972021	0.996010242	1.011780719	0.998230502	1.042062362	1.089526569	0.98952104	0.974616331	0.990916558	1.052907489	0.945450375	0.956460215
P63001	0.94740576	0.979255506	0.995380208	1.012994868	1.02338443	1.041474193	0.970454874	0.987585689	1.00483318	0.84906477	1.054004611	1.089185632
O35495	0.985948637	0.986781561	0.96557118	0.975105067	0.998295839	1.024736311	1.02981547	0.967558609	1.000958611	1.126038479	1.004838071	1.008180722
P56546	1.013753898	0.875903257	1.001594798	0.998406713	0.94511294	1.039032351	0.991591656	1.02295437	1.228954539	0.876447998	1.001549288	0.93691813
Q64336	1.06579848	1.067161939	1.049783535	0.845673845	0.797012541	0.858453584	1.16331666	1.206761494	1.275668652	0.840259785	0.84377352	0.871749856
Q01339	1.335440114	1.247567183	1.374732369	0.768057625	0.729278207	0.708615763	1.185573658	1.146105252	1.208357147	0.738626777	0.711799748	0.717325463

Q1RLL3	0.975861225	1.069652055	0.962947166	1.06439349	1.079979609	1.018727256	0.987465932	0.970544924	0.955695723	1.075533494	1.012817757	0.896389719
O88738	1.034897127	0.989163173	0.974491147	0.960055189	0.992327904	0.952146354	1.043175738	1.002998663	1.0366154	1.098372203	0.975115502	1.00074074
Q6X893	1.139343692	1.102937373	1.063252282	1.132715453	1.15669866	1.058936595	0.862237749	0.888826527	0.942437778	0.819792763	0.944363973	0.912121744
P51410	0.966314356	0.95137426	0.995950208	0.947710411	0.964044104	0.986469477	1.051989517	1.031297218	1.030307292	1.147577318	0.990186516	0.99941046
Q9EP69	1.065606188	0.996332819	0.994166344	1.014415516	1.005450596	1.028880815	1.000420698	0.975455619	0.940495629	1.055048319	1.016906636	0.971261387
Q8C163	0.948999994	0.917460648	0.967732246	0.985841471	0.953688344	1.024926689	1.072239778	1.034603779	1.048839888	1.03176328	0.989960356	1.038689933
P08071	1.293989159	1.175789766	1.237734844	0.742930339	0.691280774	0.718243217	1.279252436	1.20978074	1.250426812	0.798432309	0.752559702	0.718106955
Q9DBE8	0.977956728	0.993546782	1.018635651	0.999688163	1.053488035	1.011860912	0.993770222	1.011138624	0.979704792	1.019157811	1.015069046	0.954579752
Q8CIN4	1.015863999	1.006158346	1.004099712	0.985115722	1.011777785	1.023275451	1.018943771	1.014927229	0.991488244	0.993052379	0.990965029	0.965734095
Q8BG18	0.992955058	1.001835248	0.979206197	1.032825455	1.052714484	1.002416204	0.993867417	1.034056201	0.922404527	1.045951018	0.998909145	1.002205962
Q925E7	0.961198896	0.966598436	0.976936757	1.014597159	0.975064152	1.021759244	0.98726529	1.012947269	1.013077379	1.018199121	1.000951645	1.081031712
P97393	1.010160816	0.995488053	0.984697708	0.997105288	1.005224722	1.073974717	1.01920781	0.968155328	1.005649904	1.032144057	0.962595007	0.993837797
O35215	1.126610561	1.060981341	1.077384864	1.095991744	1.03716417	1.098568921	0.935083921	0.943364373	0.947443303	0.829335455	0.922481498	0.934173627
Q6KAU4	0.949365114	0.947789303	0.952491113	0.950825722	0.959757983	0.990212414	1.045245637	1.02817716	1.01844532	1.166470379	1.021982332	1.039460109
Q8R1N4	0.96900038	0.989801233	0.986857053	1.01684789	1.033546539	1.042544527	1.006495908	0.997119353	0.994432552	1.000361119	0.972229918	1.022624825
Q8BMI3	0.944796316	0.97905695	0.965183797	1.032337617	1.022046831	1.016163016	1.025816162	0.992390836	0.992199901	1.018758161	1.02559129	1.011792417
Q8BU31	1.149082397	0.953997724	1.060871602	0.83387784	1.088272073	1.065177045	1.025288399	0.950767845	0.850996158	1.140186603	0.940443994	1.066244966
Q8BHL3	0.953372439	0.97613728	0.974196679	1.038855641	1.043387185	1.008139639	0.978076178	0.991536274	0.953764974	1.080493294	1.021532046	1.046768916
Q8BLY2	0.930675208	0.9503066	0.995961903	0.952782394	0.907060276	0.933955929	1.092737504	1.083628316	1.110732432	1.039608541	0.98204629	1.011508563
Q61282	1.17536548	1.084875614	1.053340322	1.076873795	1.056195812	1.065448782	0.951288387	0.93989234	0.878704213	1.105507498	0.871753139	0.88696465
Q6P4S6	1.030968953	1.007015727	1.009695509	0.983074475	1.030656915	1.042295514	0.977892808	0.993618552	0.984890831	1.015995981	0.976733273	0.991415937
O08848	1.041660248	1.020556611	1.007727824	1.021718214	1.001809995	1.018203928	0.980446914	0.998337088	0.994818706	0.971009153	0.966144256	1.00660413

P36993	0.961868746	0.980716848	0.985705043	0.98648179	1.011801465	0.977388454	1.007329169	1.016286689	0.983690635	1.118359398	1.022672767	1.011726217
Q8BJU0	1.041938591	1.025399723	1.013136124	1.000560696	0.941961128	1.014853814	1.037334647	1.006492871	1.054426835	0.852488126	0.956375488	1.016607993
Q8C052	0.997586588	1.056692885	1.031604777	1.066993964	1.01726625	1.061757649	0.93173976	0.965888859	0.956906562	0.931663866	0.985889945	1.017668793
Q8BFZ9	0.983571414	0.978255746	1.02159309	1.013926836	0.994136492	1.001343333	0.988069776	0.977586777	0.995265521	0.983603905	1.052299248	1.018692355
Q99JR5	1.185256889	1.022537173	1.015846458	1.068298186	1.108177763	1.072793871	0.937606885	0.928150396	0.938916073	0.879894376	0.965927354	0.911731113
Q9WUL7	1.032559272	0.997991326	0.989176835	1.013388777	0.983888525	1.073951666	0.997124311	0.995909177	1.010569136	1.011252155	0.958115831	0.9781696
Q99K28	0.977981349	0.973818447	0.965878608	1.025568159	1.007222496	1.043853517	0.986424554	0.967101411	0.979423699	1.04712315	1.010977154	1.069003823
Q8CG76	1.027152145	1.019191324	1.035738516	1.070946244	1.023531303	0.963368258	0.936163844	0.993631299	1.036140566	0.761237393	1.004246585	1.064435942
A2A690	1.005243907	1.063546655	1.018128618	1.13356668	1.03863008	1.108462602	1.128276376	0.896327984	0.915835356	0.9033361	0.907658219	0.903107006
P63073	0.986538575	0.980731279	1.006061221	1.003588608	1.039549941	0.985334351	0.9764508	1.001558105	0.975950721	1.05042508	1.024607523	1.015122583
Q8R3P0	1.186321127	1.079683956	1.085520203	1.104513016	1.311291693	1.129467938	0.851128883	0.862884731	0.910227856	0.802183427	0.860457197	0.866271325
Q61033	1.077336639	1.043246019	0.978187509	0.942576917	1.04582042	1.007571415	0.994720393	0.996005861	1.006845689	1.082060109	0.970861788	0.924944065
O35841	0.927688037	0.957447918	0.983888113	1.02493594	1.017891185	1.026516971	0.998171068	0.98315791	0.955972654	1.085960883	1.027882126	1.070620093
P59017	0.920005375	1.04377683	0.98048458	1.015709354	0.962537414	0.964304483	1.028612704	1.072323417	0.972236782	1.021593351	1.017102865	1.017591542
Q9DBT5	1.021528732	1.006485262	1.017117814	0.989132128	1.032078993	0.979932091	0.994880281	0.99251976	0.92051532	1.095207649	1.035685191	0.985561688
Q99JP7	1.029022615	0.972677751	0.993665779	0.954744121	1.026520719	0.970934854	1.040238443	0.999601072	0.978387843	1.097176354	1.022995419	0.972214646
P58774	1.297404595	1.009333091	0.961987666	0.907236276	0.885233432	1.032085389	1.062155692	0.991111297	0.905788466	1.560147614	0.847971169	0.854733765
O70566	0.989485184	0.965456255	0.98789484	0.998066873	0.974174148	1.034651969	1.036477736	1.004191446	1.007099381	1.075871353	0.971826729	1.009071667
O08914	0.987726218	1.023589372	0.989411148	0.996935056	0.926990926	0.91361714	1.021550215	1.023190089	1.042573856	0.974536811	1.043565759	1.045888032
Q99J85	0.86762338	1.020241883	1.00902849	0.969029112	0.928741551	0.940306254	1.025455885	1.101558918	1.045854334	0.999186939	1.047814311	1.017314166
Q91XU3	0.942533942	0.958952954	0.954445462	0.986822241	1.044144658	1.071726705	1.058288935	0.986221498	0.9826409	1.033600639	0.993957021	1.023084287
Q9D2P8	1.335652535	1.338994894	1.249126172	1.325491558	1.338470576	1.080791877	0.642102404	0.711535019	0.871399846	0.523382414	0.797544062	0.797504131

P54285	0.997364213	1.044407749	0.983887353	1.020274199	1.035295714	1.036157621	1.014161495	1.000637664	0.939507795	1.016233526	0.996805027	0.958839793
B2RXS4	1.009287658	0.986770517	1.009013487	1.011884912	0.936448478	1.022199094	1.000202604	1.088262302	1.037466528	0.922978156	0.964744154	0.995448689
Q8JZW5	0.923105192	0.968288957	1.040027016	1.124467037	1.010866871	0.938868816	0.926573325	1.039659689	0.992367227	0.852020949	1.079516065	1.055602069
Q0VBD0	1.002461776	1.008922545	1.033084649	1.031582569	1.038972026	0.924025787	0.933063964	0.990942704	0.971041464	0.903756648	1.120126278	1.016412412
P32261	1.286878254	1.18164867	1.304041175	0.73258825	0.801697064	0.677984859	1.258594475	1.147194416	1.201064223	0.78178034	0.741739629	0.761998138
Q6PAK3	0.921474835	0.990546685	0.967891599	1.020373396	0.971504978	0.955176958	0.976964088	1.029029188	1.01929275	0.955630585	1.08683952	1.086352772
Q8CHP8	1.004122803	1.018415406	1.003926408	1.032620048	1.091821249	0.995099251	0.960881182	1.008362917	0.945955508	1.02936052	1.028221272	0.928522094
Q6PHU5	0.937307677	0.999592728	1.015447678	1.055586715	1.091768717	0.962922245	0.894010168	0.969014441	0.937845678	0.884837194	1.134851605	1.093183776
Q6I6G8	0.966931683	0.983697446	0.965456977	1.032941583	0.973803716	0.97927562	1.005453078	0.979433774	1.045758486	0.947867221	1.046729823	1.061866098
P61202	0.935084134	0.972771512	1.039945042	1.030494238	1.157272464	0.9983757	0.936525594	0.977825573	0.883385457	1.071402331	1.067373741	0.99443962
Q8VIM9	0.94878812	1.012999603	1.009551313	0.956849951	0.961925871	0.973772997	1.040671224	1.03769219	1.076447571	0.955888991	0.996282018	1.003290343
Q9D855	1.019801771	1.089906537	1.041257384	1.091990607	0.977904889	1.0554964	0.987146145	1.000206005	0.994127413	0.811908911	0.971294012	0.918685273
Q8R4U7	1.013337711	0.932659112	0.986410755	0.940961756	1.014117962	0.966745217	1.041433782	0.969133667	1.026074085	1.084048598	1.05831721	1.005113194
Q8VBZ3	1.024645999	1.003458923	0.987727409	0.988691817	0.97536254	1.012708251	1.028764593	0.969815023	1.017606211	1.047474863	0.991915956	0.99569937
Q8K354	1.030487217	1.036745095	0.915770786	0.956452047	1.027249156	1.037002251	0.933996556	0.9538526	1.033086461	1.119155477	1.016372657	1.026217809
Q7TQH0	0.991982897	0.963642723	0.977590124	0.954775279	1.03165666	1.01078179	1.030341121	0.999090577	1.028789552	1.165579157	0.964698346	0.969493047
Q922H2	1.028859027	0.996149192	1.007314669	1.030480748	0.982628058	1.012995812	0.995892434	0.988632492	1.015518023	0.941431601	0.989963595	1.016497338
Q9JM96	1.001758187	1.13755727	1.045720109	1.088228677	1.005557009	1.082112384	0.931856169	0.964611624	0.91176605	0.947641134	0.96967976	0.951664013
P70372	0.931283564	0.931979768	1.013384751	0.988357963	0.975600319	1.047665216	1.069183217	0.994276567	1.01909878	0.967139676	1.005072114	1.046696247
Q61941	0.760002115	0.740734863	0.750356353	0.716703186	0.717671826	0.716758498	1.200352008	1.280903006	1.217461771	1.169004109	1.417026527	1.198794206
Q8K386	0.949728075	0.978859797	1.002498916	0.974217103	0.997871522	0.971261116	1.024407529	0.998622395	0.94499613	1.105546446	1.065552937	1.038765803
P10711	0.936671853	0.949726009	0.989621487	1.012086263	0.984857867	1.008221156	1.02269884	1.028544359	1.042787401	1.004702006	0.993034289	1.036918259

Q9ERG2	0.971218523	1.057803753	0.944233665	0.990778633	0.998807588	1.024220964	0.975466845	0.983621217	1.000102938	1.089226652	1.008545276	1.021222775
Q80VQ0	1.092422176	1.077483568	1.066198182	1.050718564	1.130659956	1.068002896	0.933984311	0.928662221	0.91551531	0.912824954	0.941449805	0.923997847
Q6PDS3	1.028666798	1.013973791	0.944829031	0.951927876	1.00286982	0.992521981	1.017866831	0.999275469	1.037050382	1.17653809	0.970192531	0.96186956
Q61024	0.976023798	1.0613083	0.976863904	1.032918776	0.960027044	1.001208755	0.98695531	0.990319352	0.994360561	0.974861425	1.009099716	1.053446989
P21447	1.06456013	1.016781817	1.003504966	1.008006318	1.043535948	0.993985117	0.967780917	0.991094476	0.974631919	1.054121532	0.986503363	0.961620582
O55022	0.898151789	1.052372645	1.215459948	1.128004945	1.144109165	0.902930684	0.805024615	1.059715519	0.884917655	0.644627717	1.118850149	1.019979605
P62751	1.072102437	1.02822521	1.06240561	0.959893862	0.879251807	0.99378871	1.120379903	1.045223427	1.120101838	0.802317161	0.888169797	0.95036349
Q91ZP9	1.009391873	1.005992038	1.037894054	1.071483293	0.938455882	1.019857688	0.966754246	1.012985645	1.034276556	0.863520906	0.962442759	1.052603254
P31001	1.12200076	1.057257629	1.010578904	1.029796794	1.029308227	1.020995316	0.984338252	0.961480287	0.956414841	0.925963933	0.976783248	0.952670619
Q6ZWV3	1.007858066	0.991446615	0.967937898	0.966421136	1.035662946	0.988555057	1.007443697	0.995400222	0.998804938	1.159438164	1.016085661	0.951490742
Q8R146	1.114538423	1.01363261	1.049555518	0.978835715	0.990934373	0.975201593	1.028730123	1.026829153	1.005694695	0.958021057	0.948503999	0.924921317
Q8C2Q3	0.937369301	1.030652341	0.996931747	1.019070606	1.08381554	0.95696758	0.949864627	0.970613586	0.963935323	1.031077942	1.055658273	1.042073877
Q3THG9	1.080052795	1.06130837	1.070944612	1.043994173	1.041607736	0.967178021	0.921998281	1.003447577	1.014910665	0.865322824	0.94984602	0.971960989
P52196	1.112190336	1.073765896	1.015058568	1.036732274	1.034721371	1.005330631	0.935003559	0.947218016	0.994132828	0.961496599	0.945735176	0.987474915
Q99K10	0.952441814	0.926328031	0.931192314	0.95577936	0.991448044	1.043468642	0.994334527	1.050252272	0.975807756	1.129791016	1.092203479	1.013260933
P57759	0.969434433	1.118166805	1.008373224	0.960022164	0.917712661	0.966682225	1.037003865	0.984685419	1.013634639	1.015219118	1.013239666	1.007678898
O54941	1.039113216	1.070985216	0.997214978	1.023196172	0.96967551	1.015532184	1.025806504	1.025812904	0.978349642	0.944001521	0.951702254	0.973423168
Q60870	1.024005993	1.037130136	1.042464581	1.057965984	0.963325105	1.065724364	0.969543507	0.951293393	1.000519601	0.846977261	0.968579347	1.053286127
P63141	1.006582239	1.029851553	1.023065991	1.029210818	1.075860549	0.977838684	0.960209113	0.962829486	0.989697027	0.953306855	1.053852158	0.943638475
Q3U1F9	0.963676484	0.968066071	0.970419073	0.94048588	0.996876593	0.966743488	1.037849997	1.020742349	1.030021607	1.130177394	1.029151927	0.998699094
O54781	0.92210573	0.958148989	0.973453182	1.032753072	0.952684997	0.98653087	1.01228519	1.030753023	0.981670744	1.031904444	1.051866622	1.087086712
O55125	1.023919403	0.999384791	1.036934445	1.054700184	1.036130978	1.060635117	0.975594886	0.989760789	0.963977933	0.898729365	0.981634749	0.980578294

Q60631	0.940786944	0.996161412	0.979803876	0.998611882	1.027350228	1.043540509	1.012209469	0.980535983	0.996565497	0.983712838	1.018003013	1.035142222
Q8C0L0	0.988788322	0.939503006	0.981812877	0.978439542	0.988985949	1.107002913	1.058360504	1.021817492	0.936836427	1.236517828	0.948278157	0.944358379
Q8JZK9	1.002567892	1.034611793	1.012595056	0.997291854	0.983966187	0.999199199	1.012158547	1.042127021	1.039105601	0.886393268	0.964753913	0.997112883
Q8CHX7	1.024244774	1.045115314	1.045752178	0.99702007	1.008439895	1.008808784	0.992767582	0.957422431	0.968650281	1.059516582	0.977254139	0.976067534
A2AJI0	1.05743052	1.047564209	1.027295997	1.031958996	0.959833144	0.991172686	1.008923477	0.990346077	1.003591013	0.943533525	0.951469303	1.002336133
Q9D1P4	0.911917973	0.914304553	0.961828923	0.991104307	0.897119991	0.968344732	1.047843616	1.00434025	1.083540903	0.985141819	1.078274401	1.129761037
Q61290	0.910321556	1.010391593	0.987235986	1.08924357	0.97373997	1.018955807	1.011920832	1.011833344	0.985083848	0.951284979	1.01813067	1.030001459
Q3UGY8	0.971191864	0.997002044	0.995341356	0.948304498	1.003245504	0.919437426	1.039667564	1.035515067	1.045601691	1.008157911	1.034817496	0.998555692
Q9QYI3	0.987844052	0.984809043	0.994239681	0.981210504	0.985283141	1.052512574	1.044177938	1.003822274	1.007270451	1.029526998	0.973751	0.988813922
Q9WVA3	1.008975754	1.003990465	0.986243137	0.990601946	1.023172098	1.004232811	0.953192016	0.971955665	1.001054328	1.076251642	1.034375149	1.004600133
Q4VAA2	0.997961217	1.06546965	1.028479732	1.070367016	0.931665587	1.061157704	1.014487829	0.969885648	1.035036158	0.86042367	0.931631504	1.011329984
Q6RHR9	0.999667398	0.980891325	0.980109549	0.988959433	1.005617041	0.998625247	1.027774536	1.011717398	0.987886395	1.078748672	1.000299989	0.994439785
Q60675	1.08308601	1.014735704	1.015679627	1.042989504	1.100209583	1.055527648	0.950509151	0.946822615	0.907645974	1.042360923	0.967173783	0.962703375
A2A699	0.950765753	1.050597062	0.984580292	0.995613333	0.957954042	0.984586093	1.026389875	1.026862619	1.011131099	0.970974093	1.018372272	1.017545235
Q8VDC0	1.049262832	1.029276214	0.988788248	0.997799766	1.005633626	0.982574378	0.9699685	0.978022635	0.983632006	1.03452296	1.016742992	1.013865937
P12849	0.930741738	0.932114268	0.982382984	0.958315593	0.992882772	0.95147332	1.040531654	1.02653973	0.931513404	1.18988995	1.117294109	1.017335889
Q5SXY1	0.964413009	0.973353664	0.985492318	1.067079372	0.992169103	1.046600 3	0.991421697	1.030200178	0.973366489	0.931006463	1.018731851	1.024523075
Q6ZWN5	1.046181572	0.985324459	0.993333785	0.964051946	0.954375618	0.984575722	1.074679734	1.023183943	1.019332056	0.992033321	0.978291339	0.997244305
Q8K406	1.144779548	1.071933168	1.036026858	1.076703074	1.081626259	1.056098381	0.900650922	0.945727443	0.950972517	0.857154375	0.947823737	0.954694168
Q8C7X2	1.017308777	1.036050375	0.992455963	0.979346276	1.008632423	0.969606547	1.010366612	1.005966467	0.960193093	1.083219299	1.025758524	0.96894756
Q9QZX7	0.948509615	0.988964508	0.981428244	1.062512625	0.931992344	0.910502897	0.915026261	0.998346872	1.085971885	0.878338903	1.114348422	1.130994742
Q6VNB8	1.018709524	1.115342474	1.071489869	0.983605529	0.95446085	0.974745022	0.992927221	1.007198774	0.970047796	1.007342161	0.974654341	0.958111538

Q9JLZ3	1.105657766	1.042584364	1.061364267	1.061383696	1.050710248	1.000606046	0.925278006	0.955816367	0.970250237	0.832942363	0.996128713	0.985209341
Q8K0D5	1.029298764	1.018435922	0.96827681	1.003686299	0.991164591	1.007640924	1.006887158	0.991780857	0.992204513	0.991414541	1.002395874	1.025590424
O35621	1.413114579	1.377685865	1.292999562	0.972667379	1.080917301	0.934865549	0.888656192	0.943647483	0.92585437	0.687609495	0.750187057	0.725483933
Q8R016	1.034465475	1.006173565	1.074423783	0.885690835	0.982007882	0.888163929	1.027771264	1.050996373	1.158357582	0.906911907	0.980362823	0.945235527
Q8CCK0	1.034312243	0.972520159	1.02216817	0.968741879	0.932721063	0.963848526	1.045197782	1.01480134	1.061807673	1.008824533	0.992358808	0.991078012
Q9DAW9	1.077187328	1.0262657	1.051804854	0.982408685	0.983591461	0.981134251	1.040093417	1.028210843	1.039174461	1.018190106	0.891297358	0.920328157
P40336	1.046920334	0.989889289	0.99959179	0.963439407	0.969722754	1.001786031	1.063864081	1.016512178	1.031268688	1.046513331	0.937252046	0.9758797
Q9CQC7	1.032527074	1.118998119	1.050081737	1.136120398	1.118410652	1.10783715	0.894164383	0.945330545	0.919496376	0.871432769	0.920057146	0.918475192
Q923D2	1.072808697	1.063992861	1.101588333	0.894281957	0.902182754	0.862968866	1.09863273	1.168267431	1.176699953	0.908000672	0.858449468	0.832103262
Q9JKV1	0.915593596	0.968418738	1.010759085	1.037683763	1.04598088	0.989803188	0.946224436	0.961557022	1.004678337	0.985486046	1.091275852	1.045750273
P47809	0.97379497	0.966949935	1.007941338	1.055016604	0.980968624	0.945901279	0.97000572	1.009197202	1.04803072	0.915696861	1.037847476	1.065820845
Q91WC0	1.081514447	1.038469858	0.949164743	0.934841034	1.122975849	1.060279064	0.962716387	0.938721374	0.979337043	1.14830664	0.967073021	0.934767095
Q8CAY6	1.031870789	0.960410949	1.006297659	1.006774359	0.949351278	1.003883528	1.045868198	1.01784851	1.06809406	0.942838496	0.937182084	1.027406907
Q68EF6	0.908819341	1.021515304	0.926707567	0.974291886	0.965519527	0.95587767	1.061462861	1.019464069	0.971025406	1.152837385	1.065341647	1.039142997
Q9R1P1	1.077731785	1.05605039	1.077722062	0.901262017	0.948982447	0.885906023	1.100827298	1.04746794	1.040906216	1.024476019	0.93705349	0.913952966
Q4VAE3	1.093367269	0.99306334	1.023589137	0.990177293	1.023872453	1.038346826	1.014617317	1.011785318	0.97581176	1.024488621	0.927744017	0.941229648
O09044	1.100944454	1.01838797	1.009376708	1.018045875	1.027085926	1.078984462	0.973678452	0.987296054	1.002191779	0.939030715	0.914110206	0.965615911
Q60972	0.958473581	0.943275369	0.983333453	1.012378298	1.074545985	1.024384431	1.015251301	0.99338808	0.973120921	0.993543891	1.03974538	1.006282852
Q5XJY5	1.112873435	1.058314912	1.041652578	1.04589491	0.941456325	0.944582661	0.986771151	1.108478548	1.024174089	0.880471871	0.93574319	0.903190111
A2APX8	1.076578528	0.964715168	0.979267089	0.927558883	0.93850232	0.971970476	1.09486201	0.978937886	1.046672234	1.05720951	0.996470281	1.001077191
Q9DCM2	1.069385629	1.04244275	1.040242274	1.034647645	1.058975419	1.128211872	0.946663353	0.970830354	0.927993381	0.866863025	0.971217877	0.949787221
Q8BYK6	0.973120642	0.984118792	0.977582163	0.988347302	0.930367805	0.96159841	1.055537133	1.026730981	1.069501664	1.050538849	0.987750192	1.016508614

P62835	1.237449743	1.073043191	1.052428102	1.04345844	1.118753709	1.01149677	0.953197949	0.921234253	0.953396102	0.94847408	0.880352001	0.882977681
P63276	0.995934474	1.029132075	0.956474228	0.983239432	0.931607075	0.994819896	1.069615048	1.009914162	1.092895921	0.971451735	0.941738556	1.023525079
Q8C7M3	0.971967697	0.994309265	0.967779369	1.049150965	1.028797977	0.996646581	0.970415696	0.981581433	0.983250981	0.978196407	1.04545464	1.052336818
P32233	0.914685257	0.939132865	0.936090343	1.029806546	0.965889281	1.006912024	1.022924198	1.009216319	0.961223394	1.092333306	1.074066597	1.096587565
Q9JL26	0.977573949	1.014215976	0.977880099	0.964685104	1.003981291	0.977510727	1.035330689	0.983967604	0.985294217	1.074620552	1.041436203	1.006080452
Q9EQU5	0.993338451	0.925249283	0.982434404	0.928701512	1.037432543	1.001144066	1.089981603	0.999708314	1.00466895	1.249950535	0.951631153	0.955337335
Q62093	1.096952855	1.017087385	0.992896916	0.986700729	1.047198926	1.104571802	0.925411841	0.908520374	1.023275702	1.057216968	0.951662477	0.972879574
Q9D8W7	0.919794588	1.054161989	0.981685925	1.045888731	0.970855225	0.934638095	0.94215545	1.005282863	1.049334023	0.905530488	1.071993096	1.083807879
P35980	1.053101508	0.979348679	1.013487152	0.929342635	0.911292927	0.930129722	1.088739946	1.080998949	1.163621526	0.900993616	0.934527829	0.960764338
P83510	0.849543133	1.775243613	0.817294999	1.055083253	0.897496499	0.920368401	0.941662635	0.886226473	0.863409741	0.959666302	1.098278987	0.963517493
Q66JS6	0.972796304	0.981470694	1.001215805	0.953382853	1.048856115	0.977059984	1.016953692	1.032421668	1.007174018	1.019607955	1.026147818	0.977664288
Q8CHG3	0.94479469	1.028532974	0.959489225	0.963474048	1.000761945	0.971424258	1.025597938	1.00604045	0.956985548	1.047607213	1.057325914	1.069575723
Q8K400	1.030760169	1.002553585	1.005643281	0.974238129	0.941892108	0.985719976	1.025334195	1.018939046	1.03531521	0.98907524	0.987478353	1.013821792
P49615	1.064151749	1.072644962	0.9970032	0.951461943	1.048353103	0.969104802	0.944754221	0.957917822	1.087014508	1.034526769	0.959105514	0.960952332
Q8VDK1	1.062694156	0.967666754	1.027105898	1.022800791	0.913043862	0.986174923	0.987975137	0.998026782	1.087434823	0.930405908	0.962404455	1.048936952
Q9QZQ8	1.064078374	1.00971731	1.062530151	1.014256079	0.979644602	0.999838484	1.006452825	1.02341148	1.00081299	0.938422621	0.946478204	0.962329852
P45952	1.106977212	1.003738715	1.023183293	1.011287103	1.024061573	1.070878785	0.934841655	0.969263575	0.943342758	1.000304444	0.968260842	1.008111467
Q9CQ75	1.01481387	1.03197965	1.077052412	1.048314428	0.99984774	1.00867293	0.960758643	0.955217203	1.073441514	0.747947303	0.981436122	1.021582442
Q8R3Q2	0.944321656	0.990602974	0.969893865	0.983458757	1.037955188	0.990543227	1.030811605	0.979393193	0.989599985	1.127078196	1.050893965	0.966163266
P70207	0.922335761	0.978119495	0.929871854	0.929327892	1.021418871	1.063922126	1.04337342	1.014765226	0.950450146	1.335702167	0.989026787	0.979438653
Q62048	1.036226716	1.102608622	1.176032471	1.151496646	0.964244944	0.958544744	0.931231818	0.921445331	1.018762302	0.654239695	0.983883786	0.99755286
Q5XG69	1.111030959	1.144292124	1.181253696	0.96787481	0.93654158	0.923217216	0.974108497	1.000783051	0.992745018	1.020686379	0.866315888	0.933252999

Q9WUT3	0.996560446	0.967463705	1.049815941	1.060600066	1.003423543	1.033531071	0.939464894	1.008659577	0.96777371	0.933767746	0.998968153	1.050446097
P50171	1.033110105	0.994280855	1.037903311	1.034781043	1.035349284	1.03125911	0.946908953	1.012988338	0.960755111	0.975815555	0.968788024	1.00524707
Q8BZN6	1.078675024	1.019365316	1.002799783	0.986842128	1.066048499	1.048110983	0.938049563	0.951638657	0.98536869	1.073605462	0.970389465	0.963689244
Q9QYB1	1.073976471	0.995774403	0.971439806	0.992973553	1.062225426	1.087703007	0.979653256	0.916182701	0.904738071	1.067088273	1.011149581	1.027706887
Q8BG40	0.977738205	1.022978847	1.003248978	0.984811456	0.957998496	0.940760103	1.016649152	1.03419103	1.012240217	0.999166951	1.030692033	1.02401383
Q9WV18	1.004171117	1.058390675	1.031771154	0.944635761	0.897935013	0.876170172	1.038892808	1.047344155	1.155596774	0.886794125	0.986018155	1.006223211
Q920N7	0.984244587	0.987468821	0.934854658	0.96095053	0.98444508	0.989300199	1.039270072	1.018952813	1.034643462	1.094609356	1.021969578	0.995739462
Q9JHR7	0.960263113	1.02770679	1.054827315	1.103455721	1.017479662	1.081789755	1.02659741	0.973243557	0.979368677	0.974906865	0.917009549	0.914574887
O35459	1.124944471	1.080626655	1.042823929	1.035665688	1.091272159	0.975201658	0.876451125	0.950029953	1.00944057	0.903686094	0.984966214	0.945742897
Q8R361	1.027607538	1.007833114	1.016854596	0.967317693	1.011073285	1.008227662	0.985549376	0.959437637	0.98462082	1.102766563	1.001602851	1.000304412
O35435	1.053875081	1.026183959	1.047665012	1.005133737	1.043608439	1.064226423	0.98947062	0.959798317	0.976392072	0.999284554	0.948274235	0.932565058
P62301	1.085493177	1.055511427	1.057006977	0.931034083	0.860868518	0.940551047	1.110768148	1.123548621	1.139371367	0.858622106	0.88380574	0.886805953
Q9JJC6	0.995666302	1.024300768	1.044660225	1.01110563	0.999212869	0.909586124	1.014648854	1.053495059	0.968929116	1.072697965	1.020559346	0.925680417
Q9ESE1	0.982393403	0.999480667	1.000058521	1.017496152	0.971536693	0.95429777	1.007756191	1.051621631	0.97972927	0.99216765	1.058893162	0.988376391
Q9QUR8	1.003463046	0.969851043	0.936463374	0.973495588	1.005065701	0.942415511	1.014287849	0.986364244	1.042999194	1.091205839	1.064567139	1.014185186
O08915	1.073200595	0.993233	0.963795252	0.969492918	1.009404226	1.0617808	1.08764934	1.011086814	0.949078921	1.093352384	0.91564969	0.958869565
Q8QZT2	0.908379808	1.094142594	1.013266058	1.11828276	0.976768162	0.958060822	0.981960812	1.015919611	0.963212958	0.993344903	0.98260836	1.021750257
Q80Y17	1.040578792	0.982056473	0.984076446	1.02727629	1.032064042	1.052230351	0.933651076	0.928272231	0.965362546	0.999224272	1.040721439	1.062197646
Q8CCN5	0.923399104	1.005353626	0.990465452	1.03194487	0.981894618	1.004055767	0.961814144	0.988826354	0.976914774	1.009955116	1.075351306	1.067619126
Q80UU9	0.976159511	0.97121135	0.99277161	1.019069358	1.038074176	0.98562274	1.004905562	1.017406904	0.986729461	1.097513483	1.003130253	0.970082083
Q920Q4	1.020145913	1.057883941	1.030308525	1.00804066	0.958917801	0.949753566	0.992177302	0.996441691	0.984146634	0.964308296	1.036703319	1.005240578
P36536	0.973859888	0.975265735	0.960995959	0.995222564	1.034891154	1.065960272	1.016814051	0.918460989	0.990253363	1.030270925	1.016780674	1.065795643

P00405	0.96350997	0.921169569	0.987052886	1.009042111	0.996641548	1.110472305	1.031284208	0.970001936	0.983494709	0.966683178	1.002725153	1.070913003
Q8C078	0.900527152	0.903032739	0.899003947	0.976779376	0.967275081	0.968244454	1.074515134	1.033331131	1.043591037	1.152976447	1.046946153	1.087732525
Q8BGN3	1.049144966	0.967532477	0.866852478	0.970207113	1.078806395	1.048192488	0.955063618	0.978368943	1.068322225	1.049379757	1.010746888	1.015746964
Q99LP6	0.94682085	1.062996975	1.001337063	1.011639215	0.982720659	0.979210234	0.99592196	1.026975977	0.995947226	0.919801784	1.031395102	1.023516831
Q99J99	1.037248945	1.040905176	1.022126418	1.022830437	0.993050922	1.020084926	0.966577996	1.035206789	1.028308815	0.952033583	0.933638081	0.965445937
P42208	1.030562386	0.990751636	1.008200047	1.030183371	1.036629952	0.984002667	0.968859849	1.026828148	0.998660637	0.939195862	1.014096284	0.975676191
P62743	1.01818265	1.013994363	1.067831986	0.999478825	0.93931938	0.917794804	1.022412327	1.010713707	1.038443818	0.9115651	1.0239641	1.001699256
Q80TY0	1.013485235	1.03571255	1.008605885	1.028608171	1.051903697	1.014408492	0.958209334	0.970055472	0.936298921	0.992824642	1.025757212	1.003776096
Q9CY64	1.020645645	0.980839761	1.011011506	0.977273886	1.028838355	1.013445315	1.010679195	0.990442502	1.003375326	0.97697344	1.006007411	0.994004361
P70297	0.852750146	0.949469827	0.977008351	1.014762916	1.041325508	0.992035195	0.995682146	0.979949499	0.955572384	1.226006717	1.0741997	1.039763728
P47753	1.039731901	0.969942493	1.059481311	1.006767898	0.970745759	0.940812276	1.016439801	1.066531827	1.010129868	0.949335483	0.969880414	0.997511566
Q7TPH6	0.9780499	0.972888346	0.993893091	0.985604336	1.04121094	0.997761039	0.979254919	0.984962218	0.980383968	1.133817014	1.01046442	1.024009486
Q9QZ88	0.985043539	1.008350376	0.986426052	0.957835739	1.035065491	0.98865829	1.039143587	1.000542665	0.998213909	1.00260872	1.034796825	0.974087614
Q9DBS5	1.010696057	1.008904392	0.970961835	1.003841336	0.97383746	1.035548041	1.017908588	0.99331413	1.001033417	1.025051943	0.986374491	1.012232029
Q8R2Y0	0.967851297	1.005293432	0.956980693	0.993244398	0.989898762	1.002439688	1.036997811	0.993398561	1.00455187	1.054614159	1.020501717	1.011658232
Q61702	1.404794288	1.367500991	1.23136552	0.708381788	0.674462344	0.690641851	1.219619779	1.201176302	1.27947682	0.753262103	0.671495533	0.676981264
Q6ZWR4	0.977559238	1.044116208	0.970189183	0.938888012	0.959093356	0.926977434	1.033104926	1.056023663	1.055257834	0.973402227	1.031079183	1.016645851
Q9D1X0	1.026217643	1.255033836	1.141792396	1.004601176	0.945195743	1.003987476	0.915778635	1.035902793	0.83667208	0.719791405	1.090863078	0.937231004
Q99KX1	1.014282338	1.021656971	1.013684784	1.104717172	1.122613362	1.078250116	0.944533201	0.939722394	0.899988153	1.035252518	0.956032235	0.95850628
Q9DCZ4	0.975437251	0.947893958	1.033464389	0.988207201	1.014606442	1.065798698	1.020313209	1.005270988	1.037063618	0.990330274	0.945792935	0.992395431
Q9EQQ9	0.978009227	1.004741426	0.989716526	1.024682994	0.974675993	0.947116595	0.961065156	1.01032502	1.040509045	0.87781752	1.0579718	1.093986966
Q8BY87	1.012971463	1.157988887	0.981099473	0.986384947	0.980996917	0.992421008	1.03565268	1.003713188	0.95137062	0.98324921	0.982625912	0.956925358

Q9QZS3	0.956097191	0.968002449	0.991279607	1.053969754	0.989430033	0.948068054	0.986327026	0.975948144	1.049835871	0.944236866	1.038250216	1.088418897
P28658	0.984608207	0.93788489	1.019131907	0.999267434	1.000991474	1.037189792	1.012354014	1.027151249	0.961795013	1.066005252	1.001293281	1.002245208
Q9D1T0	0.945302097	0.945868	0.938751218	0.955659025	1.007023869	0.999297673	1.069099138	1.032289913	1.015932189	1.052185881	1.01819478	1.04580118
P05480	1.022805067	0.98985498	1.021825299	1.010682309	0.994909169	0.978941754	0.970374216	1.022066647	1.00207672	0.931897739	1.022381607	1.026865435
Q9EQP2	1.06942906	1.08272616	1.050367106	1.016690554	1.001964772	1.027525052	0.965425461	0.991449668	0.992601033	0.943577838	0.923241426	0.962407826
P13634	1.325521802	1.288832604	1.391852289	0.663012871	0.629883901	0.578959317	1.168070765	1.364969145	1.506060529	0.688129109	0.61815589	0.558158676
Q924N4	0.937645887	0.94106599	0.948712675	0.926441256	0.991150593	1.011778466	1.042807173	1.011963269	1.031540775	1.102745513	1.076215561	1.008838389
Q9WUQ2	0.999702773	0.908939543	0.98487058	1.005944863	0.990508161	0.998241701	1.010175668	0.979974601	0.975353372	1.092135298	1.035072119	1.081499978
Q69Z98	0.943020214	0.983239975	0.958513161	0.979476925	1.000850521	1.020694429	1.042402304	1.005864581	1.006841518	1.061072442	1.02379481	1.007928226
Q9CZ04	1.162407125	1.207442628	1.126638965	0.928506266	0.861049932	0.868931724	1.026498114	1.099886009	1.186862034	0.794013606	0.812600454	0.851450319
P63321	0.987044807	1.015120128	0.984228316	1.032919799	1.074273056	1.050834134	0.965169027	0.989851963	0.952217541	1.03916658	0.986484894	0.984242132
Q6A068	0.943217704	0.959003971	0.969968818	0.976189739	1.02714645	1.018343708	1.021577301	1.007397659	0.992001773	1.026362443	1.036473122	1.044749244
P97765	0.996092816	1.03429223	1.086192258	0.987833381	1.008453758	1.01760429	0.984726895	0.933956962	1.007769618	1.122264734	0.936757813	0.967240872
Q8BIW1	0.938698781	0.938740436	0.983427682	1.009568813	1.00402355	1.047886478	1.01274442	0.978814556	0.970306312	1.032624851	1.049003069	1.064530576
Q5SNZ0	1.013127548	1.04265414	0.966815064	0.971683534	1.065375056	0.960915913	0.99168939	0.99705064	0.963824495	1.085139434	1.041208873	0.961683885
Q9D7X8	0.995658637	0.977195603	0.993229361	1.009337832	1.029940556	1.047930093	0.983112355	0.984828805	0.965042215	1.067330242	1.000271429	1.009672878
Q3UV70	1.014583775	0.977301395	0.949082076	1.005916739	0.978334761	1.053655918	1.016925236	0.983205894	0.990653923	1.079728239	0.986997005	1.031703963
Q3UHK1	0.9723344	0.940953308	0.989666274	0.988421364	1.000844682	0.982139264	0.946218144	1.05468278	1.057389012	0.985525315	1.028710098	1.055165628
Q8BX10	0.97385355	0.968146808	1.002057355	0.95709011	0.961478948	0.977626121	1.04990415	1.018954601	1.029719583	1.003689222	1.012902515	1.047949065
P62073	1.026552875	1.099598774	1.063277472	1.01761348	1.091004068	1.011600996	0.848645577	0.911837616	1.053741615	1.005004225	0.94291299	0.984679379
Q9WVR4	1.03042596	1.011578551	0.897868632	0.924218617	0.987615978	1.019701684	1.025096842	0.966657334	1.068561394	1.174473987	1.003950739	0.979588919
Q9D8B7	1.059415972	1.017546511	0.998430527	1.057003794	1.143113396	1.104175585	0.917109211	0.903100202	0.931643687	0.930691749	0.990713286	0.993627672

O88456	1.015190533	0.959328584	0.935026416	0.993212544	1.02101123	0.993357263	1.006318927	0.988362508	1.009058245	1.081306011	1.013885187	1.046350002
Q8BVI5	0.978036638	1.03484267	1.038453424	1.01075863	1.012284233	0.986318579	0.998039881	0.991796157	0.996886821	0.889918241	1.026697903	1.006706702
Q78IK2	0.942391155	1.006247074	0.976201647	1.045045299	0.971601585	0.973734003	1.001867436	1.005161709	0.996061644	0.913170116	1.078011283	1.062588304
Q99N96	0.933802074	0.951060221	1.023601687	1.058695048	1.01367145	0.973870129	0.970175002	1.016502077	1.071585742	0.918821023	1.008080174	1.034915097
Q8JZU2	1.008699622	0.996027063	1.012159982	1.006116315	1.050528534	1.029095366	0.960086376	0.960515409	0.991904032	0.92105941	1.009948729	1.057996577
Q8VD65	1.017239477	0.965116196	0.994674202	0.999733761	0.977992189	0.98777361	1.03911494	1.0106515	1.022428605	1.036079584	0.998014298	0.981935288
Q8VCX5	1.192921316	1.438767745	0.851237161	0.985230495	0.984789492	0.957930205	0.911637757	0.969997499	0.911046529	0.942713623	0.95918342	0.966977945
Q6NVF9	1.047352166	0.978347233	0.985424774	0.969772136	0.957437613	1.022268939	1.064833238	1.009725558	1.058299145	1.069994285	0.92448811	0.964166961
Q9CQE1	1.145754719	1.021687991	0.884735844	0.859171287	1.045166063	1.122583537	0.949438448	0.941570737	1.079610444	1.274688128	0.898353782	0.951640817
P11404	0.973004231	0.98095475	0.963710928	0.985300239	0.980033296	1.036873018	1.056112042	0.98096481	1.0385515	1.027895128	0.974056422	1.031875785
Q62283	0.95141324	0.953176839	0.955935359	0.9997549	0.955371584	0.988262747	1.022721682	1.003290224	1.019736006	1.148753466	1.019594726	1.053917474
P80560	0.962313455	0.89804796	0.889389065	0.875230423	1.072026268	1.111413086	0.938403294	0.829552868	1.015985801	1.526372491	0.995388968	1.14927585
Q69Z26	0.953791857	0.946782422	0.992520354	0.998072611	0.966642711	1.006743958	1.046380642	0.987438114	1.010796374	1.03223701	1.020975937	1.059852828
P06728	1.19882555	1.361447475	1.228759544	0.778044061	0.743797501	0.73423994	1.162317316	1.184827104	1.269320539	0.723656849	0.747939188	0.713767209
P55264	1.00632925	1.017323211	1.011152547	1.01811209	1.248341346	1.018438717	0.910846293	0.941644909	0.89398862	1.109280371	1.010283911	0.923009003
O54724	1.106295192	1.04358203	1.010926541	0.973922512	1.031476443	1.052052117	0.996275555	0.99448446	0.961068194	1.052174185	0.91628839	0.941501049
B2RUJ5	0.959993883	0.98178662	0.962646572	0.986590395	1.017267353	1.002068566	1.030387981	0.95975372	1.004922394	1.078669614	1.018089412	1.049278688
Q6DFW4	0.988322554	0.997823901	0.959405063	0.98991586	1.030648937	0.992425224	0.996961758	0.984750895	0.996706442	1.098183	1.022719623	1.006711314
Q69ZH9	0.976821207	1.005066707	1.014391149	1.012619065	1.051913711	0.989358505	0.997498194	1.000341221	0.996637914	1.06986187	0.974527333	0.96719015
Q9EP53	0.940933301	0.990294146	0.967618963	1.049315446	0.977158334	0.988937852	1.009489828	0.983207132	1.040768419	0.935915853	1.058084743	1.037967696
P62869	1.015175452	1.023718989	1.01212721	1.024451045	1.042471615	1.019820384	0.993037445	0.989807941	0.965484972	1.015332521	0.981301462	0.962461346
Q8K3G9	0.994681141	1.008273847	0.992608857	1.064947424	1.041993909	1.020581617	0.884236174	1.004626712	0.988709587	0.98603609	1.029639509	1.018491233

Q8VD33	0.985629493	1.05710723	0.969192991	0.94728945	0.92575815	0.94099153	1.164500725	1.070608434	1.092858518	0.866388501	0.971144943	0.935316633
P10518	1.0173052	0.989703247	1.019321521	1.060847133	1.009907951	1.093389051	0.984480763	0.969540218	0.961552683	0.926399989	0.971913667	1.013756649
Q99KB8	0.995624923	1.004618491	0.965402904	0.918364233	0.98613727	0.974731848	1.006943903	1.03582145	1.081518522	0.993650793	1.006485701	1.029907286
P50446	0.766269706	0.755335321	0.815545865	1.13258979	0.781578284	0.957885985	0.746064277	0.693067999	1.522122406	2.686614948	0.86058286	0.976329119
Q8CIQ7	0.969035681	0.969869166	1.015463409	1.085914843	0.948092598	0.908555679	1.03234653	1.173501228	0.97243583	0.985081311	0.990128312	0.950980999
Q03157	0.950929215	1.059000102	1.027155149	0.994497968	1.088117688	0.943908015	0.998179245	1.009820033	0.998682286	0.913629285	1.039531111	0.949790687
O08688	0.957913746	1.000172435	1.030209414	0.949232192	0.92606513	0.91825357	1.002660309	1.074584703	1.102234934	0.956586169	1.018683918	1.026813645
Q9Z2M7	1.002024928	0.992677206	0.998083403	1.013895555	1.064927396	0.975999456	0.958595627	1.05867291	0.934028068	1.031529659	1.037376525	0.974924542
Q91YS8	0.984652339	0.935095085	1.013057231	0.991166155	0.978921634	0.99453773	1.049796913	1.033316567	1.011191902	1.015301273	0.994917265	1.01427725
Q7TNC4	1.008006697	0.977404125	0.975413878	1.006435841	1.00474036	1.007847792	1.01753646	1.028612588	0.998407616	1.033730221	0.99483292	0.985155089
P97379	1.004253878	0.955123461	0.972616483	0.949283691	1.01653403	1.00147166	1.052660871	1.001227504	1.022281681	1.112481374	0.987482591	0.986386245
P06684	1.203684272	1.235496178	1.134126471	0.840450891	0.781389706	0.794467555	1.207717751	1.146262301	1.182392454	0.802995457	0.775531091	0.800380149
P24668	0.99107949	1.052173013	1.040547646	1.033999916	0.996502198	0.963327148	0.947622194	0.984261236	1.016232992	0.891177154	1.028283068	1.031810229
Q63912	0.997835241	1.013895447	1.060361834	1.082490234	1.178694203	1.069933201	0.920651216	0.947539031	0.908785404	0.906705974	0.985664341	0.953066782
Q8R574	0.975713478	0.972129205	0.996847702	1.014785587	0.971135082	1.048615411	1.033628565	0.994444955	1.004421595	1.031377006	0.979777156	1.012414683
Q8K4P8	0.915656417	0.922210034	0.932110807	0.971275	1.009201381	0.895983701	1.079795875	1.060490121	1.023723918	1.160987417	1.066766554	1.011897493
Q99LY9	0.919915949	0.932919865	0.98713968	1.058132575	1.038705813	1.063415547	1.024735513	0.991096787	0.984568176	0.98024795	1.011022227	1.021565561
P47740	1.018240035	1.018609724	1.007014766	1.023644309	1.039162061	1.042754201	0.953614072	0.947174273	0.976371213	0.99821868	1.021831229	0.99185563
Q9CYI4	0.924739672	0.914680311	0.938073415	1.037022403	0.948947238	0.993087299	1.008033366	1.057174215	1.013192322	1.071675865	1.044767444	1.083971407
P16283	1.020725276	1.05358502	1.020790832	1.0721223	0.981790876	1.042046651	0.954054408	0.974175901	0.938566294	1.015861638	0.973746498	1.012727502
Q8R010	1.009633753	0.975582566	0.998437698	0.992122809	0.927936007	0.994945532	1.02185985	1.012843118	1.047553546	0.902795991	1.010341289	1.07680793
P49813	0.877076008	0.972053463	0.976650994	1.004597881	0.994572265	1.021673075	1.042299354	1.060815423	0.986001565	1.004883304	1.037711656	1.020700229

Q9D024	1.038787831	1.014739825	1.012452622	0.98424181	1.044415184	0.954014985	0.987859899	1.001092223	0.964982044	1.029756909	1.017943829	0.992471167
Q9WTR5	0.870525428	0.928901341	0.976851769	0.938406333	0.995478329	0.950146059	1.076183305	1.063967793	1.044586492	1.069102384	1.058685856	1.027043612
Q99M87	1.015372743	0.99884526	0.978301165	1.001322521	0.985941026	1.010702505	1.051471034	0.998157499	1.001728273	0.986357362	0.977534982	1.015418733
Q9Z275	0.981364274	1.029551246	1.004550987	1.061968199	0.999778648	1.087080132	0.983306227	0.945010272	0.948029669	1.030837423	0.958331479	1.035404683
P25444	0.978119979	0.921743044	0.964023586	0.986000375	0.93765018	0.967813771	1.043980903	0.96148582	1.070069937	0.929737006	1.10726748	1.091738206
Q99JX3	0.875345815	0.859611713	0.956677317	0.976073089	1.211881413	1.171771322	1.045375805	1.05581064	0.956148836	0.994454969	0.976150429	0.94133714
Q8JZR6	0.991396239	1.014684213	0.959569169	0.949362432	0.942307418	0.951996035	1.071521047	1.080195359	1.037429529	1.044314742	1.035799387	0.928534828
Q8R4H2	0.937306067	1.055926627	0.92637452	0.957730666	1.024300424	0.96260569	1.007208676	1.008982739	0.880559344	1.046528113	1.144594051	1.078949815
Q9Z1X4	0.962151341	0.971320855	0.963752018	0.981548638	1.011500295	0.998412402	0.963782679	0.967050817	1.052182213	1.173323917	0.986465661	1.063711542
Q8CC27	0.964519043	1.002755708	1.019388698	1.051804351	0.993777574	0.982596572	1.028631067	1.01158077	1.015152167	0.914902169	0.997515572	0.997876694
P83093	1.019611599	1.039168397	0.978223199	0.944803844	0.970363616	0.956620104	1.032538582	1.013768329	1.039534516	1.071946329	1.006175201	0.965234517
Q05D44	1.000487399	0.997233627	0.987162219	0.986418713	1.010929839	0.993248505	1.008772129	0.996044214	1.0305012	1.025440212	0.990725668	1.004326806
Q5SUR0	1.021207545	1.010761878	1.039027789	1.003252511	1.01543587	1.020197118	0.989284223	0.994876094	0.977136425	1.032666666	0.973878583	0.969677699
O09131	0.984433853	0.969168775	0.996820336	1.050346688	0.940004509	1.064893649	0.979752836	0.992720259	1.112531342	0.867234033	0.966687449	1.038707398
Q9D906	1.01129814	0.984959369	0.994647482	1.004752726	0.991464996	1.01074153	1.020479191	1.016642189	1.011828502	0.990368 3	0.978787552	1.00487721
Q8BIJ7	1.026450507	1.071099953	1.01086614	0.99618273	0.96527197	1.024473855	1.023897596	0.976715752	1.022911643	0.961598606	0.952325266	0.982660171
Q80XR2	1.011425941	1.000551835	0.996794237	0.969282652	1.002703127	0.914247378	0.983127642	1.016904091	1.009085186	1.089520395	1.032231551	1.025581137
Q9JMG7	0.998668691	0.986253061	1.055272834	1.028621478	1.071628313	0.972058606	0.966090417	0.961514269	0.919006953	1.309828046	0.958603134	0.948925902
P97429	1.191556062	1.072628725	1.05875492	1.014338019	1.087274377	1.085643819	0.94154104	0.941052714	0.934388206	0.908764038	0.917975571	0.893493913
P08414	0.905980125	0.942907014	0.928022853	1.040263207	1.016416571	1.004178986	1.015494529	1.037569525	1.021466187	0.994955336	1.032354746	1.068264027
Q8CCJ3	0.983547208	0.911849694	0.986477238	0.995055025	1.045989366	1.034290864	1.00199445	1.070416526	0.939219387	1.055077485	1.002477875	1.024860083
Q8BK72	0.992841027	1.00613992	0.989702432	1.031377825	1.005451195	0.99692155	0.975198893	1.020624891	0.978942735	0.936221966	1.024052701	1.044370714

Q8C008	0.985452956	1.012945844	1.011962549	0.980293409	0.999650934	0.981738049	1.035973456	1.042487188	0.981561313	1.101324589	0.975268908	0.950631576
Q5DTX6	0.962237314	0.946528928	0.984404606	1.018456413	1.013457181	1.013967721	1.03883278	1.000930198	0.944336393	1.055628211	1.036834882	1.028015537
Q9Z0F7	1.019431739	1.013153076	1.005980251	1.036288694	1.057993257	1.062555271	0.960727513	0.954437314	0.915618706	1.006134781	1.013325851	1.009155987
Q8K4Z0	1.112926268	1.039818343	1.01034918	1.00193678	1.042058541	1.034642665	0.955830594	0.977071553	0.974698478	0.996881988	0.93674248	0.978578493
Q99KH8	1.000869177	0.976281796	0.983310913	1.008310225	0.99347158	1.000812826	1.032421718	1.014076594	1.032937645	0.967393828	0.985550941	1.010192828
P97370	1.185140968	1.059343013	1.087103325	1.140939099	1.099839258	1.029401356	0.859528057	0.936967334	0.97483496	0.745649732	0.913713724	0.960118985
Q5M8N0	0.95213151	0.95105934	0.999776021	1.008226944	0.971209766	0.977446999	1.034455158	1.017500592	1.03604433	1.005444153	1.013994333	1.038944363
Q8BJ71	1.080136328	1.020890541	0.968584698	0.935228244	0.96610648	0.993144034	1.079297912	1.069295506	1.018793899	1.095740136	0.910113559	0.929635191
Q9D1Q6	0.959301107	0.988501405	0.948787343	0.961790542	1.021051856	0.951497056	1.021918637	1.016194218	0.968937103	1.054071279	1.106150364	1.025349911
Q91VT4	0.974208208	1.0262072	1.001919576	1.091493166	0.952418586	1.087216342	0.976342796	0.965859828	0.912881994	0.963100126	1.017570397	1.061767599
Q8R1T1	1.016513549	0.964166497	0.970675495	0.974044343	1.022304953	1.052877945	0.997874289	0.956842344	1.011003224	1.060539518	0.999884571	1.029597694
Q9CQ62	1.112852258	0.988052267	1.011735521	0.997305363	1.075425325	1.077519938	0.955937799	0.939049846	0.955847004	1.063538053	0.958939576	0.955134507
Q9ESM3	1.477048588	1.316204971	1.218092372	1.201312699	1.412897089	1.29352331	0.721836754	0.769057236	0.709429921	0.678624541	0.667972555	0.661123662
Q8BTI8	1.053054931	1.063731127	1.072090766	0.965644068	0.984203205	0.988941861	1.005832528	1.042103722	1.022418432	0.926475675	0.931854087	0.938233429
P63323	0.98300693	0.97369583	1.003653382	1.009801813	0.947152555	0.983758572	1.01087031	1.023950014	1.014909722	0.944345185	1.034853502	1.056066188
Q9CZS1	0.917868953	0.899610748	0.944011636	0.938805112	0.948968715	0.973719792	1.090045017	1.050421996	1.133028008	0.977762296	1.008833733	1.083607257
Q8BP67	1.030334991	1.041053291	1.033683631	1.076080509	0.982077498	0.982163076	0.951330829	0.995678737	1.000444757	0.789423741	1.034382377	1.027136058
O55017	1.009589655	1.066340442	0.878284 6	0.960707777	1.042609165	1.022568064	0.974849578	0.974599321	1.091012629	1.119005127	0.977063924	0.961514923
P99028	0.930190708	1.21901664	1.148459231	1.260289519	1.040377234	0.948405959	0.787607513	0.934945118	1.079411193	0.552582728	0.937422233	1.033521184
P83940	1.018615485	1.025082334	1.028409048	1.031219518	1.01621663	0.989186088	0.974003231	0.982451977	0.992612121	0.923955459	1.007221994	1.011432849
Q9ER00	0.973077865	0.942484383	1.039666665	1.040795724	1.022962769	1.015296609	1.008719371	0.99845163	1.042403856	0.899923416	0.994809172	0.995033887
Q7TNR6	0.897723771	0.971777621	0.941184502	0.915609929	1.028908782	0.874497048	1.105028585	1.037962134	1.018593534	1.071093412	1.039088669	1.114022358

Q9D7X3	0.999275806	0.998573836	0.97381385	0.987461216	1.041848099	1.012530649	1.008737187	1.007252354	1.044112388	0.950865276	0.976071589	1.002999266
O35066	0.940922851	0.954638889	0.984477156	0.983567204	0.973343277	1.014501007	1.083813352	1.069230537	0.988184051	1.04122784	1.000700582	0.983948145
Q8BI84	0.934526532	1.051860168	0.978958373	1.013471882	0.91925283	0.944103698	0.970048098	1.011235437	1.032303808	0.957753561	1.076171127	1.092766805
Q8BIK4	1.01727501	1.028331979	0.99374684	0.985729444	1.025235737	1.017015023	1.005090732	1.002191952	0.972941355	0.997923742	0.990868966	0.99541311
Q8WTY4	0.966991918	0.97609797	1.012428426	1.012596203	0.989302069	0.974928139	0.982183153	1.004772748	1.041603176	0.876797993	1.043073807	1.076431434
Q9ERR1	1.043917696	1.095124273	1.029970929	1.022290231	0.965997382	1.021157894	0.965517262	0.967048202	0.975582341	0.904885014	1.002765299	1.005403302
Q9CPU0	1.030122298	1.00706691	1.043069287	0.985331818	1.070716551	1.053629326	0.994470158	0.995391176	0.963190067	0.981763074	0.963285176	0.943995344
Q9D020	1.0027047	1.050382693	1.012166843	1.022075854	1.089755623	1.06740564	1.011684717	0.946475438	0.948974029	0.972804908	0.967992307	0.943386929
Q0VBF8	0.981094252	1.009289675	0.981346455	1.010501821	0.988872054	1.070302634	1.010112936	0.992963784	0.971093766	0.972893712	0.999068911	1.032144978
P56812	0.966605526	1.05352367	1.050604897	1.031880839	1.103763468	1.121924949	0.963826701	0.963814418	0.925959501	0.923314954	0.950441886	0.966418624
Q8BH35	1.044168434	1.234253872	1.029678853	0.775071398	0.72206771	0.705783429	1.407801634	1.240176217	1.321226356	0.82343606	0.802037186	0.727556054
P28571	1.251831954	1.110781631	1.047197854	1.043504496	1.079111836	1.084929178	0.875979966	0.933636911	0.910767318	0.92545366	0.906211532	0.910035126
Q62351	0.943604863	0.966965208	0.901083432	0.902717806	0.886627272	0.937349993	1.098994822	1.060587587	1.060892202	1.127547617	1.040629868	1.106178123
Q9Z0H4	0.996909259	0.994741963	0.973711432	1.016984285	1.011218322	1.010577853	1.012188155	0.99658042	0.997285826	0.995045728	1.002483781	1.017774493
Q06335	1.02886057	1.055991681	0.927212295	0.897704481	1.085146317	1.026832264	0.949654031	0.936162935	1.040332266	1.305390353	0.94061789	0.977013128
Q9Z2V5	1.037142601	1.008511711	1.028250855	1.069570751	0.986558182	1.069363807	1.077355688	0.926587912	0.943888911	0.958753524	0.948077428	0.97794263
Q8BGX2	1.211724807	1.113725904	1.131680733	1.122436868	1.26703888	1.142651996	0.862049103	0.88584429	0.833944754	0.828187999	0.861675521	0.805525791
P43276	1.035492032	0.956082355	1.005275602	0.881929265	0.871929509	0.89053756	1.161280766	1.087396847	1.153050982	1.039112038	0.913536715	0.997517025
Q8BG51	1.01811864	0.977923984	0.975496908	0.958385284	1.03890157	0.992137823	0.997020016	0.959062469	0.991545102	1.142120017	1.045737253	0.983691726
P35585	1.045868637	1.010431376	0.958965305	1.001653475	0.994396115	0.995432838	0.95025142	0.962067873	0.974987776	1.120297522	1.017978628	1.061136411
P01867	1.909642206	2.805052185	1.394548278	0.872446985	0.713322767	0.592217439	0.571090456	0.877175373	0.702887413	0.549277105	0.540640821	0.553650993
Q9CWE0	0.91200345	0.969422909	0.934884229	0.919535269	0.893341125	0.854195687	0.865221462	0.942484196	0.988265536	0.84970025	1.688074233	1.012579621

Q9D3D9	1.006604623	1.003607856	1.031491676	1.040146818	1.006663011	0.999184784	1.007182656	0.962137493	1.027200912	0.897004965	0.989091508	1.013804185
Q9CRA5	1.03295233	0.99836372	0.980466909	0.964409583	1.021665544	1.026364915	1.031432097	0.988548243	0.976601171	0.954097112	1.024579224	1.007051009
P55288	0.947895939	0.970444006	0.976105269	0.939318088	1.058877395	0.962489956	1.023787063	0.993066632	1.005876328	1.086549821	1.069648284	1.001085736
Q9WVT6	1.04783019	1.087084157	0.99482457	1.018138299	1.043452488	1.082443951	0.984918626	0.989715208	0.937113658	0.984384711	0.957954996	0.920505964
Q9D0M1	1.028941398	0.977885369	1.013032314	0.973803139	0.96366839	1.011675463	1.032466236	1.076623341	1.028558252	0.994413922	0.965419585	0.944384971
P14206	1.02454135	0.957174895	1.005873918	0.95145448	0.984850671	1.015675379	1.054400875	0.99418042	1.019539713	1.073803211	0.980774203	0.984467951
Q9Z2Y8	0.97925702	0.970812991	1.003323808	0.998285615	1.060183	1.08238698	0.974652692	0.970218555	0.975110222	0.971121402	1.013347853	1.022339628
Q60692	0.976440616	0.993276544	0.988305842	0.979037535	1.01520848	1.049310988	1.010489604	0.963300733	0.993511386	1.088013523	0.984310184	1.023806474
P11531	1.026293733	1.020890806	0.960376616	0.968907905	1.041436172	1.066941366	0.985381372	0.963895047	1.035469687	1.075412487	0.957971385	0.965835105
P55302	1.014346895	1.02985689	1.078165464	1.05558816	0.934997698	1.014966261	0.981593518	1.036769768	1.003210826	0.905982271	0.945637105	0.990170416
B1AZP2	0.966987333	0.987070705	1.001618075	0.964596957	0.958774991	0.986944285	1.021460983	1.054860747	1.005941558	1.012846533	1.043613467	0.998712159
Q9CT10	0.948510694	0.960380278	1.040179013	0.98817052	0.978654474	0.993509767	1.061174794	1.055302907	1.006964135	1.010467018	0.982978401	0.979822095
Q69ZU6	1.044413535	1.004359733	0.986532069	0.999715133	1.008576395	1.054365518	0.977317816	1.005331745	0.988656376	1.042226857	0.979521653	0.965326871
Q6PAR5	1.07726621	1.045656834	0.989451392	1.007326951	0.979990129	1.033890038	0.967718488	0.980725446	0.94651301	0.999688108	0.99932059	1.022329335
Q64442	1.067839054	1.264271558	0.997632921	1.024269607	0.993245948	0.960972733	0.963378068	0.947356521	0.967699223	0.90866025	0.968369357	0.953106467
Q69ZK0	0.943149872	0.909422136	0.943641127	0.997291368	0.929242521	0.966734382	1.034813655	1.001565005	1.036127131	1.096368185	1.078336504	1.09776867
P62317	1.037601846	1.039409588	1.04858984	1.063107998	1.089720795	1.066799355	0.969521817	1.002652347	0.919498347	0.902580459	0.940767644	0.940972866
P60867	1.005357926	0.972211349	1.008115966	0.968672432	1.103489781	1.058835443	0.972130474	0.962270444	0.985500039	1.175919877	0.955116013	0.947032854
Q61315	0.941998252	0.985548758	0.929796297	0.943486127	0.985129367	0.916789329	0.999453284	1.021900731	1.147108034	1.240721832	0.997535858	0.979135454
Q01063	0.991535638	0.981179964	0.978775171	0.94879921	0.969984406	1.004474439	1.040535142	1.062154115	1.106771881	1.039851887	0.946729039	0.946773028
Q8BXK8	1.034314594	0.998028269	1.069398896	1.012206948	0.995504737	0.992458962	0.933645997	1.02454969	0.978453116	0.987009401	0.970281709	1.037186835
P21278	0.95223004	0.947920323	1.010765267	0.984810304	1.077651214	1.029811366	1.002439728	0.994183024	0.979314888	1.030700496	1.030934492	0.98851026

Q9QUJ7	0.992312632	1.005320602	1.010429725	1.039440968	0.94480711	0.932441713	1.02382837	1.023147159	1.009577359	0.974154199	1.012526502	1.035345916
P47802	0.955954474	0.965440114	0.962899434	0.987172236	1.027627329	0.96245193	1.050437413	1.039823398	0.961026559	1.108593976	1.043733328	0.988504003
P59016	0.995489184	1.000970209	1.021002177	0.983422162	0.995249348	0.966879579	1.012922732	1.017776759	0.962482323	1.076260928	1.018704137	0.998651565
Q9JM76	0.979384416	1.021137937	0.970756117	1.023701435	0.989187071	1.036496662	1.02905129	0.974684657	1.035074329	0.926687923	0.974848841	1.03499773
Q62172	1.024716646	1.038846711	1.03393224	0.979849912	0.996164828	0.949030684	0.981297958	1.064755672	0.998833994	1.009262349	1.019750025	0.919044644
Q9WUP7	1.008266614	1.034414004	0.997440938	1.001610449	0.99108366	0.955666561	0.980996759	1.02000256	1.02399578	0.976904176	1.002368218	1.018825844
Q9CR57	0.970456178	0.946122211	0.986041213	0.976054408	0.936668496	0.943986683	1.047166486	1.001081505	1.019156917	1.041385774	1.040456026	1.110756869
O70551	0.965158898	1.016589183	0.966220716	0.928767783	0.98268035	0.884135092	0.980710233	1.104726561	0.933251222	1.248830838	1.077486328	1.016105328
Q3URE1	1.013749029	0.983028069	1.035915172	0.959752227	0.975599845	0.963765758	1.004161538	1.047244343	1.042301901	1.046290191	0.963646935	0.996460423
Q91YE6	1.033488836	0.99520666	1.002903392	0.96191021	0.965050249	0.954045809	1.038349185	1.01708768	1.037611258	0.991954631	1.006251157	1.002233864
P62858	0.977950815	0.976209112	1.020961188	0.967817002	0.930727818	0.991166645	1.092459364	0.97750246	1.043655641	0.923124035	1.010306886	1.054597279
O88533	0.976389302	1.007803853	1.031909484	1.134112511	1.077427102	1.090620238	0.921098325	0.959898752	0.927740632	0.921344122	0.956085242	1.031260222
Q8R3Z5	0.948725367	0.888861917	1.001744397	1.034927008	0.998984425	1.020526961	0.978281303	0.920801222	0.995962295	0.980905245	1.079345852	1.16229659
Q9QUH0	0.96644737	1.013220095	0.974884356	0.974160691	0.96809572	0.960544694	1.057520446	1.019085355	1.071575961	0.937509937	0.992526714	1.040170284
Q9Z0J4	0.964147398	1.001776144	1.070770341	1.071820676	1.002843875	1.020648675	0.937776058	0.98151885	0.964774574	0.974968837	0.997802355	1.03615573
P00493	0.985481956	1.0106787	1.0863298	1.002913447	1.059466281	1.017603387	0.942994134	0.938935627	0.952360156	0.974029004	1.021484473	1.030866081
Q9DC50	1.00554722	1.208012883	1.071315731	0.963595534	0.923401354	0.92871972	1.053441472	0.984321532	1.016024908	1.019278899	0.956142396	0.888160675
Q3UPH1	0.999382595	0.99938753	1.011533737	0.973510517	1.034749499	0.995877878	1.031952646	1.115175493	0.954723399	1.054358382	0.944703516	0.931771289
P83741	0.997059911	0.997647848	1.001482825	1.046918323	1.076521388	1.023824779	0.994685378	0.995148084	0.941872939	1.024859645	0.986284447	0.966979114
Q8C080	0.961432295	1.048072384	0.925246773	0.968902736	0.993446048	0.952898929	0.987655525	1.025009992	1.015555096	1.261278465	1.022158273	0.958561592
P08122	0.985557459	0.974616064	0.983917376	1.12934546	0.960909561	0.998394559	0.889716731	0.926651966	0.92000675	1.466459948	0.968878128	1.056888602
Q9D7S9	0.953903097	1.052728887	0.987299193	1.018542154	1.054837908	1.042539836	1.006515224	1.046076684	0.958411642	0.881907095	0.996735647	0.97920606

P0C7L0	0.943113016	1.099350027	1.007400917	1.176631904	0.893960854	0.964188797	0.96638818	0.892742057	1.010421076	0.886173618	1.04144094	1.103715397
P97434	0.999704779	1.04668979	0.976723594	1.030569434	0.999400299	1.001614331	0.999154455	0.975084265	0.959222583	1.026953946	1.012590852	1.019112904
Q99J47	0.982845791	0.944686379	0.989093158	1.013106484	1.008922891	1.009837161	1.016131601	1.010040251	1.017124928	0.998342525	0.998916428	1.030153529
P01029	1.278669676	1.148833537	1.081577277	0.867809148	1.011343541	0.922517522	1.207328809	1.058579477	1.136321597	0.746590574	0.741888698	0.728103928
P97492	0.898346178	0.950067783	1.032906836	1.086981964	1.032928403	1.042593353	1.001301968	1.027893233	0.986106982	0.980806765	0.960725606	1.016920256
Q9DBG5	1.059130829	1.084847194	0.99469357	1.032108701	1.176601908	1.096187311	0.908120518	0.941211127	0.928575112	1.006538748	0.953097596	0.899233466
Q920P5	0.921356898	0.965085705	0.975990254	0.981436907	0.985952582	1.007171298	1.061397287	1.012519982	1.000862718	1.14343904	0.974890812	1.040969445
Q60865	0.953615976	0.965792546	0.94840244	1.009933016	0.956483009	1.031848493	1.064619024	1.001422193	1.012797886	1.096633906	1.003083588	1.007337002
Q9CRY7	0.97277319	0.940248814	0.993344567	0.974910624	0.997578692	1.020141383	1.029816075	1.009561941	1.005729047	1.050749775	1.005435956	1.03456059
Q9JKL5	0.997519418	1.003735739	0.951898674	0.993680508	1.048111307	1.036591624	1.013242962	1.000006568	1.000891912	1.067178352	0.965909052	0.983774185
Q91XL9	1.003050539	0.997353041	0.948245557	0.940804963	0.949163628	0.971264505	1.055604293	0.998831361	1.062170988	1.094986499	1.005479325	1.016676116
P97355	0.961949186	0.998102134	1.039025111	0.999872394	1.11649757	0.983642911	0.960972718	0.979182053	0.955858089	0.96667876	1.052241941	0.995764923
Q8R1X6	1.012523506	1.011021415	0.991452338	1.052604728	1.062581145	1.070619321	0.935554306	0.975071425	0.945348525	1.002459898	1.00271083	0.991218951
Q9DAM7	0.962390865	0.964934732	0.951031104	0.983471251	1.077097482	0.998046015	0.93819573	1.012053252	0.973131186	1.219151718	1.05896115	0.974173027
Q8VE37	1.067330576	1.004233318	1.00356193	1.056368487	1.028909989	1.075773875	0.952496597	0.954981317	0.975062547	1.020828239	0.948878778	0.982100913
Q61464	1.05189007	1.032051662	1.002104849	0.990325761	0.982925456	1.052268563	1.022682804	0.953980574	0.95943341	1.057086625	0.980511167	0.979284863
A6H630	0.992959824	0.961322291	0.977648801	1.033342188	1.004177998	1.04160816	1.019920291	0.994406098	0.983509986	1.040046769	0.984630815	1.015822943
P02535	1.185174305	0.941966934	0.851579612	0.990466232	0.778772801	1.088171803	0.704074168	0.699788918	1.155207377	2.693441419	0.893347161	0.807554128
Q8BWG8	0.969149961	0.949574934	1.007132946	1.027372916	0.991574859	0.997206419	0.969351256	1.018147147	1.080592633	0.870740194	1.010512245	1.065730669
Q8K1Z0	0.96196131	1.020389039	1.050086413	0.988813564	1.022934969	1.098323444	0.985497465	1.016057231	0.982108568	0.955644663	0.986264075	0.934918197
P35505	1.090103551	1.045493582	1.01996314	1.069545005	1.102286311	1.058640226	0.92160663	0.945375911	0.94205806	0.898856717	0.981974201	0.952551841
P97449	1.120488915	0.980640143	1.007498006	0.947769055	0.897956032	0.950986947	1.112727281	1.037432837	1.020953157	0.984874138	0.946462499	1.0060688

Q9WTM5	1.007482664	0.954789009	1.010481083	1.006813825	0.979390462	1.024659246	1.016326664	1.036483095	1.018393176	0.924492532	0.992663508	1.015104972
P56395	1.003073365	1.018490399	1.01138764	0.994449224	1.061497638	0.993651627	0.998451457	0.988032025	1.005427407	1.015186717	0.995526787	0.946178839
Q8R2R9	0.95000701	0.978965918	0.991309431	0.986137365	1.039496932	0.998748234	1.017622775	1.009740154	0.974715534	1.069342831	1.016681608	1.013063967
Q9CQ54	1.034712033	1.046643008	1.059367546	1.10247461	0.944328867	1.070521018	0.9991745	0.966728315	0.983634264	0.833818647	0.920969316	1.022239518
Q9DBS2	0.969958938	1.041479187	1.090624524	1.054688172	0.971336437	0.957304509	0.916720506	1.044279279	0.995643931	0.860389506	1.032927757	1.022700859
P46935	1.046991437	1.023024217	1.01667251	0.97829423	1.007752942	0.98230443	1.001735558	1.028454687	0.978990658	0.999931064	1.007217557	0.953202721
Q8CJH3	1.056555406	1.032772008	0.998376242	0.894359449	0.907144524	1.005698527	1.150664468	1.069088286	1.027318042	1.069404837	0.897729759	0.929271154
P11031	0.970681074	0.951576406	1.025090954	1.039816324	1.059639957	1.068368451	0.989808727	0.963302973	0.969732881	1.078983085	0.967500375	0.987897412
Q6NZB0	1.018561888	1.008341002	1.022945086	1.03777268	1.009707711	1.056987952	1.025625515	1.003797495	0.97689006	0.959111981	0.942161994	0.961145805
Q6P2B1	0.966726681	0.990173659	1.012436079	1.045770118	1.010685773	0.961991696	0.962544783	0.999410081	1.013155439	0.917251532	1.04377678	1.059665043
Q8R404	1.008464586	0.940818987	1.066822277	1.020429216	0.950421988	1.064852414	1.041380252	1.015170801	0.985938637	0.893024031	0.975342062	1.014865749
Q62393	0.990081335	1.028545293	1.009011229	0.999941668	0.941346992	1.125296095	1.060832709	0.956670379	0.996401442	0.934475763	1.003052058	0.945257876
Q3UHD1	0.955176441	1.020544573	0.950142407	0.948394867	1.004824123	0.981865412	1.049619169	1.013094358	1.026674048	1.094254063	1.006821056	0.993523416
Q922P8	1.053026611	1.007857479	0.947223883	0.912810032	1.041900742	1.004671902	0.949180492	0.98227966	0.99547874	1.411264963	0.970597493	0.933371374
Q9JLC8	0.995278368	0.993800749	0.967365865	0.970189711	1.079291728	1.008512298	0.99613093	0.977823041	0.958467163	1.091900924	1.042666961	0.983828979
P62889	1.010737617	0.987598576	1.028096808	0.989127774	1.020676743	0.938478787	0.995304587	1.001371886	1.041684343	0.88355526	1.007074632	1.064590286
Q8C167	1.137541565	1.026696785	1.090159519	0.912552461	0.998993179	1.004285511	0.962447023	0.946451363	0.999608244	1.00478413	0.99955817	0.952405816
P11438	1.104077623	1.157337142	1.052041277	1.067506263	1.07385248	1.047674217	0.904269953	0.965689926	0.936785908	0.821576379	0.953470319	0.916266934
P16460	1.180665598	1.027921032	1.143678921	1.102118871	1.177496066	1.184966774	0.846982663	0.90444352	0.858212457	0.906459502	0.871991053	0.879724277
Q9D892	0.949693441	0.988586266	0.999729575	1.046290765	1.085030092	1.037231686	0.96112651	0.988121038	0.916863162	1.059802961	1.038611169	0.990090881
Q8BL86	0.945660612	0.947862946	0.993887483	0.968787493	0.950975266	1.013903867	1.043395525	1.018301428	1.013939478	1.053568532	1.018126877	1.056212927
Q8BGH4	0.963565949	1.095436322	1.028713748	1.073672514	1.011700844	0.886819427	0.908414956	0.995416989	1.065676795	0.804649634	1.043113996	1.058032959

Q3UHH2	1.033519838	1.003559164	1.008236256	0.958139949	0.954252447	0.960054513	1.040459664	1.030357176	1.043677977	1.001871142	0.978940246	0.998958175
P60521	1.026360218	1.00986033	1.041558518	1.002435789	1.01699884	1.058666393	1.008791283	0.995398224	0.966490418	0.977924523	0.958761486	0.965460991
Q9CQJ6	0.980496957	0.964513021	0.983418277	0.988769318	1.031398965	1.025806958	0.971943769	0.974756201	0.994039898	1.110512705	1.008263358	1.040946943
P11352	1.052037432	0.957831764	0.969006471	0.906877959	0.931748097	1.011527607	1.085105705	1.023369802	1.16228218	1.017594487	0.936887579	0.948945833
P70392	0.964348505	0.952017653	0.987495889	1.0049382	0.993472008	1.02084165	0.98692366	0.965120829	0.992179715	0.977818663	1.080958416	1.078000503
Q8R123	0.997876714	1.000013241	1.009210387	1.016700113	0.998872569	1.013460372	0.985365525	0.997403491	1.009096292	0.945156944	0.992863459	1.039169286
Q91VW3	0.967342232	1.006211256	0.983198812	1.062580272	1.027428842	1.04289996	1.062317058	0.941077299	0.99645716	0.903390919	0.989719461	1.007650302
Q99JY8	0.918890022	0.947080089	1.008151784	1.084323615	1.091658759	1.033022693	0.937373288	0.972366751	0.913501683	1.041004218	1.070352695	1.033728241
P47941	0.93089625	0.941347757	0.992619901	1.015719592	0.974894503	1.059887233	1.020237709	1.006081633	1.014616971	1.084995294	0.984049463	1.025322388
P49442	0.962611378	0.960270687	0.965392096	0.994082574	0.998605893	1.047233907	1.035995253	1.016760886	0.962227578	1.088177846	0.985863403	1.046446839
Q9D0G0	1.080935036	1.041911333	0.981799117	0.984945154	1.075703629	1.067495233	0.94857571	0.961692128	1.032246423	1.077330007	0.933420802	0.898752761
Q99NB9	1.037153342	0.992615714	0.998700759	0.998692808	1.012046618	0.988584494	1.004359602	0.982651757	1.024783738	1.014577701	0.998412109	0.978836133
Q9JM14	0.972534042	1.03798716	1.065043889	0.982983353	0.979878392	0.977521214	1.011676509	1.052749289	1.086312542	0.830931947	0.969289096	0.961684686
Q8BHG1	0.938938299	0.956925137	0.962801034	0.980103285	1.015241113	0.978744989	1.004138826	1.026053871	1.005152359	1.004240783	1.0666971	1.066333065
Q9CXT8	1.152840991	1.053853618	1.008023211	1.05534951	1.184851458	1.079112628	0.941116003	0.892079684	0.864095079	0.997577162	0.954831194	0.915950977
Q8BTZ7	0.945865086	0.958893203	0.96541129	1.009306038	1.002294274	0.973297242	1.037229911	1.040102162	0.984693557	1.031475113	1.044019144	1.02780076
Q9QXV0	1.045266866	1.085467456	1.085254192	0.943707609	0.951755573	0.879824841	1.024116847	1.066989302	1.077503101	0.977207094	0.967694771	0.8810029
O08808	0.877206205	0.993669275	1.015386807	1.07101237	1.081015508	0.980997098	1.00025358	1.007800626	0.934873247	1.017457938	1.04754515	0.996470605
Q8JZP2	0.944960646	0.989243287	1.011093674	1.089092844	1.026632568	1.09838792	0.952216328	0.982486734	0.889837711	1.067508743	1.01224192	1.012906029
P10107	1.352693658	1.134296269	1.208686962	0.790812259	0.884115431	0.805360388	1.155902677	1.10934341	1.027457934	0.954717083	0.784127686	0.803080919
P70335	1.109758745	1.028934953	1.035410462	0.990533996	1.032071539	1.052786665	0.946727376	1.063764755	0.974431953	0.961221173	0.927890907	0.913105278
P47856	1.00211821	0.97630549	1.008689235	1.004205752	1.002004593	0.973259089	0.995400366	1.034248554	0.982907138	1.028046666	1.041295694	0.9775824

Q91W90	1.049018529	0.996069711	1.022586494	0.992627203	1.052608414	0.991458642	0.976038944	0.956723825	0.908837246	1.09251141	1.010741817	1.03817584
Q69ZZ6	0.934437129	1.122269674	0.985485081	1.067772625	0.999407775	1.01570993	0.931620598	0.935370942	0.972003487	0.963981441	1.056713346	1.031935695
Q9Z0R6	1.025054807	0.981712918	0.982305571	1.01516976	1.019441219	1.017408969	1.001712049	0.977393801	0.961599119	1.049508967	1.004348582	1.02334897
Q9WV98	0.979480239	1.043672704	1.09675593	1.188978979	0.909314918	0.83644578	0.885127807	0.9853366	1.09327565	0.732342817	1.073843567	1.073358465
P61022	1.00457803	1.023697077	1.045139864	1.050750142	0.967792402	1.034816368	0.955855035	0.937316093	0.917508649	1.054009232	1.028395223	1.045598138
P63242	1.010715976	1.008609931	1.03021043	1.041945328	0.996120813	1.029494143	1.001222102	1.032422173	1.010425215	0.941658274	0.942691616	0.962714672
O89051	1.080893462	1.037098814	1.048201797	0.956236527	0.976154776	0.940335371	1.04825015	1.024030496	1.091229901	0.919986387	0.911129132	0.951846486
O35988	0.948333449	1.096022678	1.033592933	0.907486701	0.938577149	0.995038921	1.163317867	1.093639414	1.133197629	0.828125858	0.882756631	0.887219169
E9Q8I9	0.981991699	0.975655202	0.991336984	0.983125536	0.968427157	0.968103329	1.026974545	1.02941018	1.009291046	1.055501943	1.024095896	1.016477411
P62245	1.092273394	1.000777133	0.996552623	1.004535495	0.921925916	1.013432293	1.02310418	0.946336338	1.03140319	1.026549035	0.910630195	1.093778361
P27005	1.454049776	1.328939127	1.55479505	0.601873796	0.637017764	0.593085822	1.350856279	1.173038782	1.243938814	0.604735619	0.639627871	0.598336557
P54763	0.971397091	1.015150399	0.992539361	1.010366615	1.026046108	0.907851534	0.963632417	1.007325158	1.035820099	0.951403185	1.077035348	1.026136745
Q9CPR4	1.003378025	0.990382336	0.996545463	1.029324331	0.941815419	1.019111935	1.066627136	1.01629366	1.052592193	0.893922797	0.952597175	1.011494607
Q8C7D2	1.020174009	1.035680985	1.058568415	1.030418071	0.96956157	0.935754367	0.948562883	1.054201408	1.105366739	0.832528166	0.963184495	0.98989334
Q9WUC3	0.953354349	1.043559708	1.116681425	1.057110333	0.928356731	0.902036458	0.912747507	1.060132127	1.022048221	0.765313521	1.079815521	1.057524549
Q8BG02	1.01741146	1.030290031	1.017400072	1.054034678	0.993788892	0.918328652	0.951239896	0.975465577	1.033899648	0.775824751	1.094254631	1.059016721
Q8R180	0.943174742	0.971157572	0.986550925	0.98550996	1.001243222	0.96406092	0.990750729	1.004722608	1.066225579	1.067218522	1.048224266	0.994979069
Q924C1	1.005406305	0.986386366	0.975577484	0.972580142	0.964441988	0.977395548	1.042516179	1.052250498	1.017525822	0.943406681	1.024237717	1.021716842
Q8QZR5	1.081075954	1.097587974	1.060901243	1.069138182	1.14368448	1.105050655	0.907593025	0.911115742	0.920206964	0.94542787	0.909289717	0.915978578
Q8VH51	1.018791026	0.971158788	0.954820792	0.962865533	0.955959688	1.023005354	1.068586665	1.067625195	0.960364182	1.149256154	0.972321064	0.979787817
Q8BJH1	0.970769645	0.998032713	0.971992682	1.003485168	0.944931188	0.990118982	1.068864151	1.00183191	1.041834107	0.956321973	0.994149134	1.048642171
Q8K1S4	0.986667627	0.979554561	1.008751428	0.976565188	0.998443599	0.999875485	1.016997053	1.003603984	0.997704247	1.046377663	1.021095625	0.996714122

P97742	1.125764959	1.045899793	1.032769454	1.035163233	1.086017356	1.07460037	0.939386383	0.963548671	0.917302708	0.9188974	0.97185703	0.92724289
P62484	0.930247594	0.998780552	0.942326905	1.013313473	0.980730415	1.013161923	1.035974456	1.043710594	0.999836436	1.000085869	1.020095262	1.032332102
P46425	1.044785872	1.111648378	1.064697497	1.026960389	1.032621634	1.011833082	0.932143694	0.971495762	0.893649818	1.053266576	0.995313467	0.937770305
Q8VE33	1.012958782	1.039551929	1.001924392	1.013849811	1.081409	0.966682115	0.910992133	1.012305501	0.971293727	0.971745572	1.051939052	0.986315883
Q60749	0.968550165	0.948805788	0.989637442	1.038839002	1.041204996	1.021807502	0.97340592	0.999183046	0.980401879	1.117991766	1.01167662	0.986055956
Q5SV85	0.97013504	0.980947528	0.990007084	1.002221608	1.045918358	1.019821142	0.980049072	0.990790736	1.008444324	1.066869	1.014333854	0.980108374
O35350	1.039714648	0.981409467	0.990731528	0.948072477	1.000166662	1.032444551	1.04920487	0.99909003	0.989686379	1.0746811	0.974526875	0.977570777
Q9EQ80	1.00483361	0.973289816	1.007922702	1.012390559	0.976109292	0.975378719	1.003789975	0.980834078	1.023357436	0.936599534	1.037317377	1.057319221
Q64511	0.991275247	0.987994565	0.999400681	1.035413599	0.996447355	0.99805711	0.982098825	0.950864965	0.979138815	1.038168644	1.031259042	1.056102678
Q8R0F8	1.009579747	1.007226365	1.035355225	1.024388119	1.019504284	1.014270324	0.991050712	0.985718343	0.988109426	0.983832159	0.969278979	1.000472708
Q6KAR6	1.045448477	1.078425211	1.05628368	0.910645966	0.902752238	0.8694307	1.095217746	1.0977067	1.081458904	0.936982316	0.948984483	0.938045283
Q8VI75	1.045437528	1.013698399	1.012585386	1.001630359	0.951607778	1.037326955	1.00737491	0.998735507	0.988472414	0.972935054	0.96736963	1.027686808
Q80XK6	1.002502046	1.076240015	0.978758622	0.977181823	0.975051163	1.0623498	1.007031717	0.957991297	0.946311124	1.238800725	0.979981507	0.933827075
Q8VED9	1.024558806	1.023759901	1.037750903	1.099717951	1.057799254	1.120222606	0.926769719	0.914723082	0.933321006	0.884464681	0.978975862	1.018604318
Q6PHS9	1.048205925	0.998640285	1.100316852	1.086497586	1.076416486	1.091748163	0.883773208	0.999007514	0.891744101	0.898746389	0.991588378	0.955781644
A2AP18	1.023260154	1.026701304	1.027456768	1.060903025	1.032402149	1.069999324	0.956581048	0.991025323	0.953818635	0.981694441	0.94470896	0.979670566
Q9QX11	1.039192631	1.00718719	0.984283389	1.058158534	1.007334332	1.097820894	0.976131578	0.985973515	0.959656628	0.940990789	0.984853409	0.984605346
Q3UH93	0.944246223	0.92238666	0.998070548	1.014648257	0.935335527	0.961902239	1.07402487	1.09993559	1.042866984	0.999882009	0.970687699	1.0333655
Q7TN29	0.875358836	0.918712076	0.976198667	1.03333258	0.979988836	1.002945243	1.02282275	1.016971782	1.021249718	1.046475667	1.041567572	1.082357174
Q9D2N4	0.94955429	1.005498156	0.97641758	1.060655786	1.033835174	1.012061219	0.979466759	0.964265153	1.009086205	0.896457531	1.039775566	1.055037204
O54984	0.958237261	0.979766746	0.999320822	1.021062572	1.091201422	1.044665886	0.955832357	0.965552679	0.93837113	0.983577205	1.061400892	1.02628777
Q5KU39	0.997678098	0.990096661	0.986533965	0.981632294	0.987582664	0.999022507	1.033264099	1.023205542	1.001564406	1.044662975	0.993230144	0.99797342

Q8BML9	0.995856064	0.98506138	0.973395754	1.002403208	1.003226938	1.004674293	0.99715716	0.975304222	0.991625685	1.017432432	1.053183382	1.028214968
O08528	0.922713393	0.908422936	0.998671566	1.015755065	0.963604995	0.964048703	0.962854237	1.032045958	1.103260615	1.025900244	1.01830916	1.091745553
O54734	0.998388816	0.935200036	0.972505785	0.966019613	0.953524002	1.002830053	1.060228203	0.968353841	1.050777734	1.02471742	0.999848224	1.08954454
Q61207	1.026639258	1.118845607	1.054124645	1.094625085	0.990785173	0.932673984	0.982820981	0.962755347	1.01550413	0.862947197	0.971569118	0.966915386
Q9DBZ5	0.966369186	0.966033393	1.028652667	1.045364288	0.961122958	0.909107457	0.946176199	0.98580761	1.110552344	0.810202053	1.06827726	1.121583893
Q9EPE9	1.005744022	0.966322835	0.930772955	0.988778337	0.993821696	0.99570521	1.029126969	0.982968612	1.019685715	1.086556939	1.020867904	1.036511421
Q9CZX9	0.986914242	1.012705359	0.993429713	0.956446092	0.905032497	1.011318959	1.044002816	1.037255885	1.048089013	1.007672663	1.006538602	0.992151535
Q9CR00	1.02009027	0.998341855	1.015446833	1.003597229	0.994503146	1.038245045	1.043450208	1.051025281	0.971334671	1.004090078	0.945951361	0.94598668
P10493	1.059136104	0.959510078	1.001588102	1.037512953	0.998510597	0.99828432	0.94805815	0.9680937	0.994680452	1.011792958	1.054832324	1.005143698
P32020	1.057877449	1.000137051	1.039028114	1.015545217	0.980586983	1.039776103	1.000549145	1.015588021	0.999807878	0.886345347	0.949501453	1.006617343
P61222	0.996126764	1.011380322	0.965012799	0.933961703	0.945560257	0.979230371	1.018475866	1.031505598	1.049452476	1.090276499	1.000797686	1.021618461
Q9CR51	0.957494548	1.00508401	1.01750169	1.099215408	0.858582765	1.025630768	0.984177101	1.011059389	1.069551751	0.770136797	0.973861868	1.153041335
Q8VDM6	0.997812807	1.000341693	1.01006509	1.028847176	1.059203465	0.943682534	0.937969465	0.918445026	0.971534399	1.217613946	1.002239993	1.046900089
A2ARS0	0.900671821	0.949717742	0.968061291	1.118912016	1.039213183	1.125472757	0.949126077	0.980688067	0.963056	0.926864129	0.973507106	1.125536159
P53026	1.020081679	1.042092347	1.010749789	0.996318781	0.954406811	1.067148026	1.033254468	1.036410452	1.05750204	0.821181347	0.949126113	0.956506459
Q9CR61	0.95847699	1.004708009	1.022276281	0.994195382	1.000031499	0.99586153	1.003060362	1.026922605	0.998318944	0.964783524	1.026141532	0.999765081
Q6NXK8	1.047210485	1.069554779	1.008431217	0.986238166	0.956548054	0.94713448	0.972898738	1.046538436	0.990751366	0.970651408	1.0111896	1.00485394
P37804	1.655782664	1.167302496	1.076927701	0.927564016	0.840670062	0.973023137	0.901057492	1.022941688	0.987622327	0.863537298	0.819105584	0.835989355
Q61037	0.986218514	0.996199112	1.011095386	1.050789673	1.045502011	1.071326649	0.966149406	0.989288904	0.947667679	1.066908482	0.969043453	0.974365288
Q9CQZ5	0.993089354	1.0766394	1.060215394	1.05082534	0.957127864	1.049757564	0.991800372	1.103496449	0.985853684	0.837240946	0.932415691	0.922000796
Q9DCB4	0.772596826	0.978693726	1.030106919	1.32925523	1.102697026	0.994314001	0.896504701	1.019522606	0.918265734	0.821200445	1.014596494	1.096008368
P70188	1.108041147	1.028484677	0.993332896	0.951106894	0.958703408	1.005170826	1.02569653	0.995638102	1.038457256	0.996026511	0.949092576	0.982028647

Q9CYR0	0.969566663	0.989112168	1.032028929	1.033337696	1.011134169	0.989656947	0.982981223	1.031025056	1.013625512	0.912437071	0.9898521	1.029744326
P26450	0.992502837	0.990383012	0.988096002	1.017032611	0.997961091	0.949181482	0.997462805	0.98476071	1.03597566	0.917721074	1.044182415	1.064565999
Q06185	0.908563039	1.105478842	1.087133745	1.112703212	0.9739748	1.093813167	0.919848408	0.965384161	0.980865288	0.761484176	0.963697391	1.069721484
Q8K0C9	1.00503846	0.976275575	1.021107904	1.069549989	1.000501484	0.998984979	0.960566634	0.941680779	1.010897907	0.915640036	1.016233245	1.084215633
Q8K1A6	0.975786544	0.965871432	1.037346348	1.028483228	1.029596678	1.003047612	1.043056283	0.996236115	1.047268233	0.939712721	0.953402495	0.972894469
Q3TDN2	1.14277586	1.060758836	1.000198996	0.978190629	1.011308633	1.062742902	0.99157644	0.91930402	0.924230932	1.091983297	0.957163977	0.966930354
Q8VHW2	0.871912985	1.093219917	0.908486942	0.980737304	1.003371628	0.920262539	1.029724037	1.055202172	0.968306635	1.110569078	1.092089241	1.004520852
O09167	1.101202317	1.01310415	1.043061287	0.978523335	0.982334974	0.988728055	1.084973067	1.060733466	1.035425101	0.926942711	0.889752607	0.893468876
Q3UJP5	1.006728994	1.031621831	1.019286968	0.991942494	0.987270035	0.967942957	0.967209149	1.004910262	0.994465945	0.954932425	1.038494833	1.035966392
Q6PHZ8	1.024954476	1.06202302	1.029281578	0.990471513	0.972970797	0.942364486	0.988176469	0.993899465	0.995192087	0.968773915	1.021373809	1.018528229
P16014	0.994000348	1.016968035	1.062462172	1.036482762	0.925445417	0.928827934	1.0136538	1.002669204	1.066688983	0.931013616	0.99339959	1.005025954
Q921H8	1.114591899	1.016233249	1.055604308	0.905333428	0.913755927	0.953170158	1.070780271	1.097713826	1.157214874	0.92570264	0.871545748	0.889296804
Q8VEH5	0.938367564	0.992954574	0.994125546	1.006527461	1.121232823	0.986982167	1.005934286	1.008740017	0.899586187	1.10489794	1.052060192	0.957028748
Q61686	0.965493104	0.958886382	1.038286607	1.031160832	1.0412543	1.031380483	0.974606154	0.977000487	0.978564375	0.997431066	1.000061287	1.03512823
Q69ZS6	1.040775302	0.982258407	1.106479511	1.164341804	1.030333066	1.091154705	0.873877319	0.977288421	0.965883569	0.893669558	0.926740337	0.974423382
Q9D0J8	0.825923765	0.932490275	0.968091719	1.093287082	1.176043695	1.137602809	1.002386559	0.931521853	1.006490678	0.860140211	0.986734529	1.05429235
Q6WQJ1	0.952612107	0.966785267	0.968447474	0.967327787	0.967930668	0.983820177	1.012590337	1.006012769	0.997722585	1.123011814	1.067925767	1.038708516
Q91V09	0.94045825	0.967767729	0.990104346	1.000523133	1.045142376	0.997394742	0.983973571	1.012986479	0.940260105	1.085387195	1.070048324	1.017123469
Q8VE70	1.019980683	1.015624814	1.028114559	1.031681376	0.936686037	1.080673885	1.058202	1.004151252	1.046388277	0.847672364	0.899574862	1.000478808
Q9QYR9	1.053975453	1.089198567	0.968572202	1.031761164	1.081568744	1.049646041	0.866179377	0.918689437	0.996018377	0.96251716	1.014828069	1.013094683
O08919	0.908347838	0.974288791	0.981740905	0.999079151	1.016383942	1.008307414	1.036150651	1.007941029	0.985002909	1.049519522	1.014351974	1.050659018
Q9JKN6	0.986223109	0.944051579	1.020738188	1.013904901	0.989861923	1.027938021	1.021131457	0.961349404	1.001840709	1.050348532	0.994205745	1.033055664

Q91XD7	0.9718498	0.971668752	0.99410138	1.016706175	0.968414694	0.967147461	0.993844838	1.028997446	1.037678598	0.994325712	1.01718985	1.046045028
A6H5Z3	0.965783338	0.984698886	1.003873511	0.990943218	0.998756142	0.960182383	0.971627139	0.992968187	1.046437988	0.924925591	1.069660253	1.061732932
P17809	1.051679135	0.967035388	0.952744598	1.022608942	0.978309037	1.121455853	0.98005927	0.960608964	1.022238555	0.970210068	0.976112065	1.030352134
P54754	0.963796182	1.040715275	0.975011034	0.98466333	0.949859352	0.952285273	1.044526352	0.98183282	1.050966994	1.05024895	1.010371726	1.020944986
Q80Y14	1.029615027	0.975371059	0.961961456	0.974048882	1.014871415	0.992167349	1.06515868	0.986124059	1.068183573	0.925772763	0.990576736	1.000091299
Q7TQ95	0.952679739	0.98025235	0.992553448	1.036813314	1.029634348	1.004726043	0.981479013	0.978086856	0.928636934	1.151364805	1.040774025	1.01562303
O35681	1.007070845	0.972253243	1.005705948	1.004570582	1.041816427	0.971129459	1.009955774	1.04155141	1.003073846	1.007295535	0.994014591	0.96519808
Q3UVL4	0.988390132	1.080658591	1.001765271	1.019689288	1.003508038	0.947347683	0.952532425	1.003647739	0.983222506	1.008766167	1.040335557	0.99696873
Q9EST5	0.887123818	0.971236694	1.003028548	1.071894438	1.147146953	1.062150029	0.968214004	0.996219671	0.959795636	0.958201845	0.990435936	1.003012794
Q8VHY0	1.016101232	1.030634307	0.990112289	1.002913334	1.025949234	1.021888911	0.993069002	1.007070912	0.989378157	1.038343338	0.961911536	0.97591067
Q8BGD5	0.996119216	0.937128322	0.989785355	0.966961883	1.029082802	0.959047782	0.979772811	1.072000666	1.027740194	1.077597435	1.046331039	0.952787936
Q9D6S7	1.033370462	1.029481756	1.040745202	0.983048936	0.98650682	1.053230113	1.018943021	1.00355321	0.983686723	0.97279287	0.944080292	0.973856922
Q3UKJ7	1.057495826	1.005634591	1.031496661	1.027901499	0.992039427	0.998296519	0.96526572	1.024151417	1.010930361	0.927750923	0.974031093	0.99050488
Q9DC16	1.011719577	0.94398288	0.970450817	0.970434841	0.947450702	1.021086339	1.034728001	1.020450819	1.024823196	1.055440322	0.998859092	1.038550146
Q9EQ06	1.090171354	0.97530948	1.029567694	0.967369842	1.095125158	1.08414186	0.98709826	0.998167006	0.990739318	1.02181783	0.917237699	0.904235454
Q9JIG8	1.032764068	1.053615431	0.998996705	0.996723189	0.919020854	0.997933103	1.050418703	1.044160469	1.034644627	0.924404147	0.960672948	0.972510458
O88545	1.040644952	1.005493156	0.985867247	0.959006229	0.938473873	1.061914635	1.077603581	1.013678934	0.980132363	1.048480026	0.96025844	0.974481679
Q0VGU4	0.990827276	1.018445579	1.000569825	0.968534636	1.022602424	1.04649863	0.967531177	0.98395722	0.988656647	1.048463822	1.001837008	1.011190649
P43275	1.118313565	1.05205151	1.006554583	0.959319083	0.991368077	1.033589988	1.032993155	1.015513254	1.078399508	0.946606855	0.894901971	0.883606083
Q6P5H2	1.113139598	1.068146197	1.033589131	1.031588117	0.952081777	0.987319694	1.011585772	1.053930199	1.036686342	0.876684152	0.903447374	0.921849368
Q9JJU8	0.969732757	0.93511189	0.986202926	1.023178998	0.980962904	1.100801431	1.047664442	0.985065836	0.971444793	1.042828182	0.999619686	0.999760859
Q8VEK0	0.967352012	0.973004078	1.024050486	1.020688019	1.003253022	1.036176619	1.012294914	0.991906402	0.947904463	1.037673491	1.016139496	1.009983973

O88951	0.966738271	0.939807188	0.963980658	0.974896847	0.961224536	1.006971974	1.051091127	1.017612419	1.017328836	1.027631617	1.037522585	1.04988284
Q9D0F3	0.97787044	0.972182179	1.009350454	0.976299495	1.003235312	0.987205385	1.024760394	1.007089976	1.007252513	0.978114022	1.045659697	1.007007535
O09172	1.043185658	1.034635059	1.040225579	1.005218982	0.918760596	0.920473584	0.969275452	1.090994796	1.14903959	0.747248498	0.960507287	1.018454702
Q8CFV4	0.923688274	0.954758011	0.990522106	1.078026126	1.038499353	0.986354947	1.042010866	0.988113803	1.006580231	0.973228308	0.986219215	1.043162986
P97352	1.143842853	1.112310462	1.109638701	0.975574514	0.96835956	1.105535306	0.97204779	0.979989412	1.01312585	0.838034618	0.864448287	0.909357258
Q9R0P4	0.781205378	0.961656606	0.999030726	1.082062787	1.224112246	0.999435099	0.962607563	1.034472451	0.884297768	1.127532556	1.062290243	0.952610791
P61967	0.99118418	1.046564346	0.968306676	0.996786126	0.922923925	1.045174075	1.076610686	1.016798181	0.949823681	1.017043092	1.021924139	0.970282515
Q8BFQ8	1.002610075	0.977169557	0.999666421	1.025237972	0.989706602	1.006047723	0.998695628	1.020777263	0.98936986	1.050426196	0.972299158	1.020624743
Q9CQ92	0.953906729	0.99539235	1.036082876	1.081162009	0.919822097	1.015527557	0.971831108	0.997568378	1.017778186	0.849634841	1.001546988	1.120721989
P97411	1.021990141	1.060067168	1.019994106	1.015329667	0.996783668	1.001354849	0.982769757	1.013617783	0.969316666	0.924566307	1.000365137	0.995096701
Q9D710	1.065206641	1.086303582	0.999273983	0.975102355	0.975325611	0.902923347	1.046624651	1.09754008	0.994856431	0.932218115	0.99159163	0.91949895
P01865	1.729005137	1.619499985	1.283402167	0.891299008	0.693198851	0.812147518	0.833178832	0.949358451	0.880606443	0.951072907	0.737684291	0.752348214
Q9CY34	0.948785589	0.957508288	0.985716086	1.023117742	1.017006748	1.026463733	1.037682348	1.007579874	0.999192045	1.00228462	1.000111104	1.013036555
Q5SW19	1.045189141	1.086892214	1.0339414	0.952128893	0.933107331	0.971653856	1.02497347	0.985088364	1.023696013	0.998094327	1.014521747	0.939736833
P63216	0.891859029	1.114210872	1.025258807	1.073341334	0.881873788	0.964401853	0.959339886	1.039161513	1.04579296	0.84443395	1.02851397	1.067904622
Q9D1C8	1.006652494	1.047707813	1.004501653	0.990445977	1.017869193	0.956796678	0.977978294	1.047723562	1.002029485	1.042326761	0.996063168	0.948257022
P54923	1.023230743	1.041424394	0.993702872	0.953302813	0.950851788	0.965373595	1.066464588	1.066209016	1.031442497	0.984310284	0.949057243	0.980813087
Q71RI9	1.053868598	1.016815048	1.036228699	1.110225866	1.083461923	1.065386722	0.899708365	0.920338817	0.96986531	0.88819086	0.978475961	1.000172746
Q3UHU5	0.966363977	1.022332835	0.999926847	1.002152246	1.039373033	0.970800082	0.985783553	1.011688581	0.985047862	1.069062224	1.018720971	0.976818448
Q62189	1.043914477	1.021924683	1.011287615	1.012771715	0.997548703	1.001184047	1.014896044	1.031195386	0.992432257	0.951926682	0.965335531	0.968249184
Q8BH44	0.956877064	1.032448718	0.985156021	1.02027392	1.003704879	0.937292389	0.932837684	0.978535381	1.003840074	0.947224043	1.107855912	1.083189191
P62918	0.978113923	0.966972464	0.96964453	0.960379711	0.898959224	0.98917789	1.05927746	1.016606953	1.050696764	0.940241824	1.054916734	1.082310089

Q9QYI5	1.031654251	1.087932298	1.007199093	1.045207479	1.105612476	1.095803897	0.931290038	0.941256744	0.951345331	0.995426321	0.952493838	0.918491623
Q8CI32	1.034613505	1.04348325	1.031375639	1.015244887	0.943496322	0.952430536	0.964667837	1.040270077	0.975075882	0.960511906	1.003139028	1.048493362
Q9CW03	1.09018011	0.987819297	0.99843537	0.977706951	0.977290173	1.008412089	1.044367473	1.005999043	1.039389958	1.018342875	0.940301186	0.950236936
P70699	0.962510657	0.981819854	1.025458717	1.020935819	1.022452503	0.983128258	0.998251862	0.992181005	0.939558938	1.07977461	1.043393429	1.004489131
Q8BH58	0.919912012	0.961086917	1.075686585	1.038761417	1.001158332	1.023266464	0.996664563	1.020878115	0.965510242	0.993114622	1.038846543	0.968434949
Q99K01	0.987287332	0.959515861	1.017562528	1.009026441	0.999618413	0.988057613	1.001163058	0.967257863	1.009391653	0.957717976	1.037211804	1.06584617
Q923S9	1.136969675	1.071588406	0.982336945	0.972052105	1.035343165	1.038801695	0.962160353	0.984197879	0.956625238	1.071323634	0.974283667	0.901122996
Q922J6	1.063733048	1.056422863	1.057459735	1.108461938	1.114875393	0.99387776	0.888244123	0.929151539	1.008619102	0.767242574	0.983704584	0.993970822
P10639	0.983648828	0.965902419	0.996962602	0.984206297	1.010895246	0.996301975	1.020106386	1.04582725	0.993680951	1.040739057	0.992401364	1.003622625
Q9CPW4	0.896909317	0.992562408	0.971373484	1.023865523	1.011829888	1.06218649	1.029767959	0.938553447	0.995088234	0.906504311	1.055201543	1.090467307
Q8JZR0	0.957068156	0.995436727	0.980912012	0.972666275	1.012446357	1.002241196	1.021616831	0.97815218	1.02563744	1.141434477	0.989200302	0.99711805
Q1HFZ0	0.995832613	0.974130302	1.006588133	0.917596073	0.926532705	0.947509723	1.087133216	1.026091697	1.052305125	1.000751913	0.999029549	1.060467096
Q8BFT9	1.032029784	0.97567486	0.98856385	0.994570853	0.986980653	1.012455808	1.000783659	0.97466865	0.957064899	1.103902547	1.041683073	1.00292307
Q8BWT5	1.043293016	1.005772483	1.000391532	1.014958077	1.026069642	1.002899456	0.94879429	0.941681812	0.992174201	0.942473922	1.050952676	1.042043824
Q9QUN9	0.954159698	1.044994546	1.013406285	0.938295779	0.991573674	0.982180766	1.0862565	0.962015047	1.029655009	1.022108469	1.01027635	0.972039654
Q8CFE4	0.953208685	0.985695971	0.949874659	1.019896792	1.026142707	0.979507213	0.986967397	0.992834467	0.980944216	1.158929969	1.034067615	1.02075129
Q61704	1.188871444	1.117744761	1.046940638	0.970891824	0.95349858	0.956344405	1.003668376	0.994541996	0.998375176	0.886912332	0.954844055	0.926838038
P53996	0.954923031	1.316177298	0.988752291	1.011328092	0.917743885	0.867160293	0.932341542	1.018935988	1.079434698	0.787616187	1.004156095	1.045017327
Q9WTR1	0.948101478	0.951958519	0.984628299	0.991754435	0.985908772	0.997889398	1.010572113	0.988277787	0.995592069	1.165324879	1.033256878	1.026279389
P70302	1.020927147	0.99832476	1.004938359	0.986622173	0.942325866	0.960405678	1.052515311	1.066070926	1.070413148	0.9519144	0.950171988	0.983967066
P61358	1.039211215	1.020666288	1.030618338	0.986933923	0.964265133	1.034867509	1.071200731	1.021496597	1.032667151	0.821907046	0.94775539	0.974775004
Q3TCH7	1.005897819	0.978980512	0.98823992	0.963146384	1.024303537	1.01467172	1.02236574	1.01639287	1.02858184	0.933522944	0.997495518	1.015989909

Q3V0K9	1.253014164	1.163621537	1.142327793	1.039022868	1.097778967	1.055863605	0.905082022	0.917436797	0.898278706	0.936456991	0.829563821	0.850688303
Q9D9V3	0.969302568	1.003843912	0.972000802	0.997343223	0.996232911	0.975624865	1.012057797	1.036722236	0.997090058	1.02943088	1.027403374	1.007713333
Q08274	0.959807195	0.987534553	1.022971832	1.024507718	0.97601202	0.965231443	1.016617204	1.033483524	1.012205606	1.037774618	0.986915106	1.005449309
Q6NS60	0.914419109	0.957289251	0.951422449	0.944656098	0.967949885	0.996824174	1.033652839	1.005982722	1.003230391	1.089343307	1.087619571	1.07615611
Q9JHW2	0.93789368	0.975591093	0.950135146	1.007253054	0.954156928	1.343287779	0.922978641	0.983238388	1.025837646	0.918736843	0.964900832	1.023187881
Q3U5Q7	1.047546357	1.015406816	0.972790647	0.966430618	1.039802898	1.014071517	0.980342454	0.942316053	1.066945483	1.069590065	0.970411883	0.975973696
Q0VE82	1.064146036	1.066498597	1.119287453	1.028863443	1.020598019	1.02945195	0.87806757	0.997692066	0.904012435	0.894040148	1.027147673	0.975559865
Q8BRK8	0.982846101	1.008954347	0.996908452	1.030867586	0.991952659	1.001139278	0.991951553	0.993741157	0.966183048	1.047035947	1.016207763	1.020092359
Q9CQJ8	1.007824001	1.074909438	1.002109226	1.008126788	0.929388584	1.005617977	1.032035089	1.014273312	0.983133578	0.996950608	0.973098825	0.997563151
Q8BG95	1.009399258	1.013053503	1.052022231	1.055699782	1.022703331	1.011655371	0.971865134	1.016400104	0.96450526	0.976921786	0.969610616	0.965322996
Q3UYG8	0.969998291	1.021785619	1.014593927	1.093300681	0.982534886	0.946525284	0.896630322	1.008387108	1.036138419	0.824655856	1.018436834	1.14605558
Q9DCT8	1.01380074	1.076515877	1.006615689	0.968525947	0.891931031	0.92315486	1.106591872	1.079716179	1.075417419	0.91789315	0.937181086	0.966594995
Q9QYJ3	0.94940025	1.008396486	1.001032893	0.997651461	0.965273767	0.967364947	1.023570594	1.002579234	1.023798883	0.979937521	1.024027449	1.054486022
Q69ZR2	1.020852352	1.068401539	0.985586431	0.978439911	1.024056341	1.014936199	1.019483506	0.971992797	0.957890263	1.106698848	0.972127468	0.962530537
P63046	0.874478386	0.993040082	0.985357241	1.057936346	1.006334731	0.834706611	0.843078963	0.976466089	1.088872897	0.76180332	1.287900457	1.157249896
P62069	1.363897987	1.133273335	0.98551935	0.864142252	0.990203728	1.027858369	1.176794053	1.059783515	0.800024704	1.114096945	0.822714295	0.805244421
Q8C6E0	0.99616861	1.012840094	0.995365361	0.999577065	0.962558797	1.021755418	1.032476956	1.011153827	1.021375441	0.977379425	0.978178241	1.001196448
Q7TSC1	0.928936092	0.990168	0.983159324	1.03477785	0.972471916	1.005041895	1.038363277	1.010346286	0.993525784	1.058705935	0.985957599	1.040850395
P0C192	0.985821703	0.975296314	0.985070038	0.983610466	1.014566066	1.001268119	1.015988547	0.985444882	0.974780616	1.067385685	1.022910825	1.038400492
Q8CIG8	0.977473641	0.992478698	1.008557664	0.99078209	1.055467295	0.994940228	0.99819558	1.052755589	0.990548177	0.953468	1.003261282	0.981144753
Q9QUR7	0.880136327	0.984350149	0.99631248	1.048854728	1.022257923	0.975569713	0.960012967	0.982532226	1.029708087	0.877728821	1.101096404	1.089437457
P15209	0.977619773	0.955418164	0.98439711	1.003338435	0.98048315	0.962666863	1.023062774	0.99798643	1.037889997	0.972666131	1.043256391	1.055733747

Q3TKT4	0.963041528	0.96365539	0.96422973	1.007407585	0.976142044	1.066485003	1.020684675	0.964794383	1.023671603	1.004618754	1.024157422	1.039829856
Q9ESX5	1.039175978	0.990779322	1.027756246	0.978490917	1.01259501	0.98251953	0.981875568	0.953371715	1.026980712	1.00494174	1.014084528	1.013465943
Q9DCJ1	0.902233828	0.960726079	0.952218089	0.98822986	1.022867027	1.009585318	1.01707827	0.991273313	1.013248725	1.016981952	1.080261628	1.051058793
Q9DAS9	0.978013124	1.26129645	1.056914964	1.142927687	0.987834718	1.028893498	0.850329347	0.942817463	0.975956826	0.764092493	0.98930302	0.982015602
Q80U40	0.996221625	1.021552513	0.981942917	1.0117754	0.998204648	0.982713185	0.953581458	0.963538799	1.026316135	1.011385998	1.034288271	1.047731099
P61290	0.980361542	1.004142461	0.999191143	0.981968823	0.96204246	0.901852445	0.989820359	1.088355826	1.080422496	0.902814435	1.041987324	1.016776322
Q8R5H1	0.96429431	1.000200133	0.99622305	1.029920405	1.039754233	0.984748201	0.976479935	0.976524033	1.006401437	0.982361404	1.051345578	1.001824513
Q6GYP7	0.982595711	0.965875637	0.991920612	0.921225603	0.93422037	0.930218637	1.067505416	1.176506985	1.072504361	1.028428763	0.962618195	0.961625771
D3Z6Q9	1.049612613	1.012528956	1.078146536	0.977064877	0.977580509	0.941991911	1.029352394	1.019203851	0.996052141	1.087103973	0.950393959	0.939162061
Q3UIU2	0.982214528	1.116774368	0.991911445	0.999027695	1.007126977	1.051265927	0.964657741	0.993706654	0.969547094	0.992900506	1.010757614	0.947723527
Q99JW2	1.026700583	0.976663148	1.001537891	1.055111598	1.005682343	1.059804041	1.002107479	0.98339935	1.023281076	0.941050571	0.949170523	0.991539633
P04919	1.14306059	1.061368134	1.144138535	0.729882572	0.719856112	0.671121495	1.208035809	1.404541503	1.523611606	0.773586609	0.718420995	0.702384082
P62270	0.985709785	1.066242864	0.994715437	1.02818637	0.902542083	1.052733335	1.042485794	0.99321665	1.05144281	0.797494283	0.977758715	1.040591928
P22723	1.07915836	1.058819992	1.027587647	0.914022008	0.967026572	0.930677389	1.0337139	1.017243057	1.024271966	1.062925109	0.959535549	0.970612584
Q5IRJ6	1.06992981	1.063354311	1.03935819	0.99187583	1.07476499	1.029675055	0.960691209	0.973931883	0.967407714	1.044943259	0.945641432	0.909323683
Q9ET01	1.05221829	1.012676729	1.093831943	1.066962298	1.064042682	1.073588489	0.974529162	0.965555879	0.951214934	0.893072809	0.932485648	0.932828794
P97772	1.191170606	1.197260971	0.952594003	0.974420449	1.082379232	1.0162634	0.867151565	0.895398207	0.93833532	1.089028464	0.978121238	0.945757321
Q3UTH8	0.976363674	0.974786464	0.972686739	1.003937276	1.049906285	1.007329009	0.999737029	0.985010155	0.994261101	1.065230941	1.030989213	0.988166354
Q9QZM0	1.201211596	1.203467454	1.079567791	1.124163075	1.076377523	1.132408346	0.91195644	0.919476157	0.832259703	1.000456461	0.830723896	0.818703524
Q9CQW2	1.01810132	0.949698489	0.973750131	0.974414392	0.923249237	1.028775975	1.085268893	0.971195543	1.017454214	1.083190243	0.979944885	1.048018189
Q9JK42	1.032011402	0.996682638	1.034533121	1.041123138	0.958073154	0.994667634	0.983827199	1.002318531	0.998312034	0.974861034	0.979583018	1.026584819
Q8R0H9	0.942102922	1.024109903	1.002039652	1.034303731	1.029373139	0.997855647	0.963511864	0.99423188	0.968496069	0.926599983	1.056855664	1.051536974

P19221	1.16089397	1.187810654	1.140708667	0.868294733	0.817353143	0.818962481	1.154541208	1.100800057	1.154240496	0.845642877	0.835574387	0.839215798
Q8BXR9	1.031079189	1.038162706	0.964170072	0.95881727	1.076447469	1.031686083	0.96911916	0.980485318	0.953615465	1.16953028	1.00043994	0.938317196
Q8BGN8	0.938621439	0.95178947	1.013377142	0.996164809	1.059992587	0.952691346	0.998471705	1.017869824	0.960382366	0.993966508	1.106150307	1.008628541
E9Q4S1	0.907513477	0.936108512	0.971018585	1.028828571	1.033292888	1.03777507	1.006288734	1.037536455	0.97951606	0.982609306	1.03195757	1.054663559
P68037	0.984342037	1.032550732	1.09114152	1.083600474	0.999806926	1.082549933	0.913684488	1.039605041	0.944649424	0.819677618	0.980741566	0.995446067
Q9D6Y7	1.088832288	1.039307258	0.993400351	0.963550973	1.053627964	0.974974334	1.001185541	0.976545216	1.016029115	1.048003905	0.981967955	0.917164551
Q3TC72	1.020194334	1.05669238	1.019968964	1.030599537	0.981151347	0.984591093	0.949630212	0.952914106	1.067601528	0.938336865	0.992622883	1.007349666
P59764	0.955021638	0.93070218	0.942028561	0.967184786	0.977603254	1.029894426	1.006996689	1.002935074	1.054763349	1.076349102	1.028243433	1.06636541
P67984	1.003229765	0.945426955	0.944481425	0.962801245	0.974566137	0.998164336	1.072523035	0.997726435	1.001192545	1.24524881	0.975604467	1.000730314
Q8BMP6	1.014885022	0.982105325	0.990449951	1.004865987	0.98065283	1.009965606	1.026007784	1.022478314	0.986785796	1.00007316	1.004208443	1.000409014
Q9JJL8	0.989002934	0.962661612	0.985140901	1.005402887	0.911820789	1.012401626	1.045114468	1.027777699	1.068415367	0.931340274	0.966074151	1.078186405
Q5SSZ5	1.024691513	1.026859324	1.043507587	1.008381595	0.978331397	0.994949687	0.983067338	0.973528904	1.003301027	0.977658648	0.997021627	1.00695451
Q8VC30	1.012764124	0.995698987	1.010325199	0.967010018	0.947409832	0.972785352	0.990922849	1.059563805	1.014403711	1.0134956	1.019646569	1.010670052
Q9ERL9	1.038813788	1.091027561	1.066875456	1.019169687	0.997815046	1.022996978	0.964855904	1.005494955	0.990758367	0.924775973	0.959460565	0.923189725
Q8VDQ1	0.990486962	1.027211551	0.986299838	1.033391866	1.088041451	1.011596105	0.933322935	0.956815419	0.957597859	1.006635984	1.030362057	1.02475114
P19253	1.090198802	0.991796129	0.937766047	0.964676322	1.034016987	1.040198662	1.014502641	1.00577978	1.018906354	1.110079572	0.93825341	0.944249205
Q62178	0.964422075	0.944682463	0.96881128	1.011545548	0.971065579	1.033820118	1.015443994	0.989913821	1.016415145	1.062004415	1.003550171	1.063850136
Q8C015	1.008202682	0.966674609	0.971806896	1.003854808	0.976404596	0.975252358	0.999015763	0.994108088	1.013513793	0.99257683	1.051427778	1.060385467
P63080	0.956081814	0.953049112	0.95716138	0.968883153	0.990055799	1.020732522	1.040288453	0.933406657	1.006665375	1.106702348	1.042283053	1.08231854
Q9WU28	0.939692091	1.069524999	1.013552323	1.013669947	0.961629419	1.067629292	1.002371789	0.988806517	1.010349976	0.820199142	1.000135505	1.056833043
Q8C5W0	1.091647149	1.072351994	1.028861579	0.974229611	0.99035428	0.964038992	0.985367826	1.03900831	0.988497872	0.963367091	0.975318868	0.945347318
P20108	0.993425216	0.972159718	1.032052706	1.089052581	1.008628516	1.067083921	0.976836488	0.979420798	1.015090721	0.869488448	0.973115449	1.006817646

Q9CQ45	1.00895798	1.030065775	1.082912052	1.000660094	0.999728889	1.058174359	1.026365496	0.946245139	1.011559086	0.9455752	0.946632091	0.948855705
Q80XA6	0.959128539	0.971484995	0.971126113	1.018442349	1.015520782	1.020248603	1.045535003	0.974494279	0.971549774	1.098894391	1.003840552	1.01548498
Q6PEB6	0.906241892	1.01062813	1.021145292	1.100169606	1.103659899	0.902753991	0.876827634	1.021114334	0.968361065	0.829651384	1.137152281	1.065834984
Q9CQU5	0.970963319	0.983556558	1.002367786	0.957391584	0.889001805	0.993517737	1.06139718	1.073133797	1.033099349	1.023854298	1.005867884	1.008039559
Q3V1U8	0.973404484	0.948133157	0.981568338	1.012812991	1.003286626	1.031133325	1.012965769	0.953423617	0.938068542	1.037151155	1.030746224	1.126812996
Q62348	1.074170683	1.096859023	1.137219221	1.130297089	1.201608482	1.056006281	0.864586463	0.945822506	0.894607326	0.752920452	0.909188452	0.930224353
Q9JK48	0.977328024	0.96052267	0.946110061	0.995553696	0.94232862	1.037984859	1.048200462	0.996470842	1.000538901	0.984990607	1.027406321	1.092630872
Q9QX47	1.032504871	1.000526636	0.947455639	0.961463481	1.033700996	1.039378497	1.00852568	0.984341666	1.055866725	1.164357198	0.928965933	0.945686548
P55088	1.085056847	1.01691973	1.02012923	1.019914999	1.00621087	0.961715423	0.97835128	1.023360236	0.936519458	1.087186805	1.00209513	0.940200833
O88703	1.17964281	1.123685505	0.992894308	1.015340242	1.025971337	1.009015714	0.952029925	0.958737498	0.922192035	1.074928631	0.949618279	0.905104477
Q9DB34	1.113131986	1.071368319	1.148551514	0.934707238	0.990585753	0.957009548	1.056932372	1.019311393	0.995859521	0.899105689	0.918571333	0.87720847
Q9ER35	1.177914903	1.040350024	0.949443423	1.031315317	1.052626873	1.084362265	0.954149415	0.958701647	1.00886302	0.935816293	0.93888141	0.915391355
Q8C0D5	1.025298081	0.978100377	1.022647513	1.012460923	0.990512223	0.983065502	0.999131177	1.019828742	0.982621802	1.070999741	0.995947641	0.97462746
O08663	1.015185654	0.96071132	0.974692215	0.947530112	0.990633581	1.028559995	1.004425969	0.98059252	1.006873799	1.13383318	1.031377055	0.997899234
Q60803	1.104714745	1.000673472	1.120900848	1.033705168	0.975298939	0.953799862	0.912088115	0.97943202	0.985298608	0.87450381	1.017088748	1.029264563
O35226	0.964906944	0.967491518	1.015971617	1.04144079	0.932688968	1.000327369	1.02154868	0.999046706	1.016663489	0.958899881	0.99178098	1.090301078
Q69ZS8	0.886130335	0.982427435	0.990579681	1.076333967	0.98501175	0.996808801	0.92754356	0.994931159	1.016949404	0.873406882	1.094418814	1.13116108
O70251	0.925069614	0.95300852	0.983291356	0.925713834	0.911788052	0.960814869	1.043832723	1.04175851	0.993023573	1.094589934	1.082961767	1.107859437
P60670	0.968310073	0.989946633	0.973753244	0.983761974	1.000663225	1.023879114	1.037811549	0.997873185	0.992598987	1.067275473	0.993969664	1.018267896
P06909	1.276119196	1.306666678	1.166450988	0.806761568	0.775807045	0.79182998	1.184849591	1.133681115	1.18127483	0.758803351	0.775845553	0.734008406
P70236	1.000967034	1.006197707	1.019051312	1.015319324	0.976256376	0.965315803	1.022098001	1.035202366	0.981588312	0.970396839	0.99193302	1.025414908
Q3TPX4	0.923107459	0.948862454	0.966049071	1.017314454	1.009509652	1.014766827	0.988969697	0.994969683	1.028616048	0.992627654	1.05936383	1.059406597

Q922Y1	1.036297326	1.092503083	1.0429632	1.017090806	0.964953386	0.997790468	1.024753703	0.992751707	1.009208802	0.888429362	0.961577151	0.953837332
Q8CH25	0.990336277	0.992478957	1.011796448	1.003847783	1.038694947	1.014630209	1.002648186	1.006762802	0.958749426	1.060928523	1.007327071	0.963882312
O88998	0.951484793	0.996688687	0.955952417	0.990507511	0.980342695	1.019729291	1.023578881	1.010826676	0.95705955	1.110331853	1.021094499	1.049040942
Q9Z0V2	1.029768528	1.043360448	1.007316749	1.017993478	0.951188808	0.960694845	0.985455207	0.989100432	1.049844769	0.904019825	1.009835559	1.031653298
P14115	0.982122926	0.959030937	1.017928285	1.002534458	0.923235302	1.027747943	1.033700738	0.997791287	0.995093855	1.17923028	0.996401239	0.97262686
Q9R0M6	0.975668774	1.018930044	0.993004416	0.981283367	1.06505267	1.031343077	0.979025945	1.006814476	0.993872442	1.05405539	0.982573089	0.969546658
Q99J77	1.057831331	1.032918795	1.01033594	1.057129336	0.976953244	0.973483664	0.964995089	1.021773175	1.032290053	0.861197461	0.983863224	1.004145993
Q8K0G5	0.97002866	0.986969303	0.98878266	1.023734648	1.046120152	0.98894341	0.983970556	0.973739811	0.94988567	1.09328819	1.028113478	1.037106461
Q9QYH6	0.930928056	0.95324286	1.005659865	1.046064524	0.985366416	1.059151919	1.030631695	0.962420527	0.974937787	1.063246601	0.996378423	1.042620533
Q61220	0.990439782	1.014060373	0.986515701	0.899057793	0.848973364	0.936029389	1.16673905	1.014901788	1.202496921	0.890621487	0.947119846	1.025512064
Q6Y7W8	0.903933335	0.962616079	0.939291065	1.020188816	0.98304619	0.961207574	1.022506841	1.020760871	1.022215255	1.044231302	1.077762908	1.056281909
Q9JHS3	0.886994579	0.994031886	1.051729905	1.008723496	0.967468658	0.909573902	1.038667312	1.066594764	1.037983976	0.91206896	1.04108589	1.028644273
Q80W21	1.150945358	1.162633559	1.146369939	1.049169622	1.043848583	0.949064311	0.945395744	0.983089524	1.015860261	0.734036587	0.893737134	0.874699685
A2BDX3	1.084489352	1.042413713	0.988924147	1.02067472	0.959400315	1.045784642	0.972395807	1.021100999	1.053994664	0.953846487	0.918420451	0.963848989
Q80W04	0.989900438	1.087108873	1.054128904	1.078465852	1.026589976	1.054496463	0.944173651	0.988459078	0.94800733	0.915201555	0.973127533	0.952280419
Q6VNS1	0.929135744	0.949214755	0.961059674	0.985298754	1.010536732	1.019276149	1.031695194	1.020859894	0.974503521	1.050349814	1.049977389	1.047271485
Q9WTL7	1.035275679	1.037454745	1.007429061	1.040794042	0.972650883	1.062932993	1.005403803	0.968911864	0.994368147	0.873918403	0.976484778	1.012387982
Q64012	0.992217583	0.975264164	0.954739167	0.97818793	0.989633706	1.048477237	0.990533679	0.930195173	1.003543167	1.187939062	1.014049495	1.042715206
P02469	1.043700386	1.001303258	0.954044793	0.947590639	0.926628293	0.919680816	1.037507843	1.04680636	1.025251129	1.127601414	1.034298134	0.992831534
Q9CPT4	1.023537176	0.935969642	0.984061331	0.985667294	0.94209933	1.02369856	1.063511039	1.030703181	1.042844233	1.013327085	0.96422279	1.011339474
P51910	1.160753787	1.190598905	1.122002078	0.948321696	1.12086352	1.010006625	0.937932345	0.951275881	0.894932503	0.951101618	0.918649888	0.848661591
Q8CG72	1.045779901	1.004611468	0.991753055	0.996665904	0.934451702	0.976181718	1.021517669	0.972805895	1.027744366	1.029075977	0.997360543	1.037858597

Q6PD03	0.968211845	0.986476763	0.989735985	1.01783162	0.994308567	0.996445817	1.006621915	1.025485571	1.005589268	0.933945096	1.009557056	1.057023396
Q6PDI5	0.947468357	0.978754365	1.018123729	1.048425441	1.012420015	0.938551714	0.953659322	0.990744782	1.010189833	0.929443968	1.09012497	1.060524128
Q6PA06	1.140490632	1.088948508	1.159753232	0.947380083	0.98658411	0.935326877	0.975975408	0.975166254	0.955588046	0.967477406	0.958486694	0.935053724
Q9JMD3	0.958475593	1.07544775	1.039236244	1.043592435	0.999259351	0.995851904	1.000664976	1.02284511	0.928089056	0.976585777	0.982047088	1.00083536
Q00915	0.914159108	0.945192916	1.064657697	1.084090926	0.980124103	0.96974194	0.917116545	1.086686169	1.050915182	0.787852921	1.037307183	1.076591242
Q9D1R9	1.012270244	1.060928974	1.075222246	1.066677557	0.943433301	0.96112846	1.017631548	1.080605529	1.034058175	0.732694959	0.96963481	0.947907936
Q9CWZ3	0.932456149	0.971061679	1.004949423	0.964176394	1.108555909	0.973350966	1.001084905	0.988334045	0.901743805	1.318514945	1.019271508	0.974895154
Q8K0T4	0.907057418	0.930580124	0.978989246	0.94877016	0.989031408	0.951656688	1.09162404	1.066768793	1.052269369	1.064843003	1.026081031	0.99764836
Q9DB60	1.046488405	0.990713948	1.019779095	0.984499746	0.986806522	1.006149347	0.979754631	0.964262305	1.00206669	0.98517549	1.018326898	1.03873054
Q922Q4	0.911737499	0.989323494	0.97716814	0.991943219	1.022628665	1.024170083	1.016241353	0.9962319	1.018630568	1.021338604	1.024825048	1.021858812
Q8K298	1.171258782	1.14235065	1.054393915	1.182585586	1.213882116	1.049782742	0.802142739	0.891197354	0.896458652	0.805609476	0.93006766	0.905932346
Q8C3W1	1.03319053	1.124784826	1.137671448	1.054668505	1.082920822	1.113179765	0.893258172	0.973842238	0.868627323	1.091635465	0.892959206	0.849320062
Q03173	1.054539043	1.029134861	1.009135231	1.017880204	0.969057627	1.019904803	1.01018629	0.950834109	1.019247301	1.087509665	0.933503967	0.977735142
Q8BHC1	0.976019695	0.957372191	1.010923329	0.977502129	0.994826192	1.002516735	1.048927622	1.039847888	1.005590228	1.072742752	0.978867529	0.976020059
Q8CFI0	0.943094383	0.924563149	0.997673347	1.012791848	1.002939426	0.978967181	1.015250207	1.031929634	1.036109478	0.958269534	1.049478781	1.030196551
Q5SSM3	1.047551394	0.979798621	0.950082065	1.023672986	0.912631348	1.017524359	1.031064825	1.003301916	1.041579552	1.000912803	0.990886158	1.026193156
Q9JI90	1.167137948	1.1433296	0.935128867	0.887816328	1.102076276	1.029712573	0.903024499	0.933533605	0.971449541	1.268995039	0.927917518	0.920182592
Q91VK4	1.021934353	1.010213783	1.047577907	0.953297898	1.000368552	0.982870325	1.039393371	1.042009936	1.032284764	1.044090276	0.92335943	0.939085432
Q9DC28	0.974444342	0.974779108	1.006403821	1.015676282	1.007834505	1.018770452	1.025478809	0.987708387	1.005081569	1.014638025	0.989091741	1.007982138
Q3U9G9	1.083116672	1.010517562	0.944980065	0.90144641	0.928884588	1.009565332	1.096953756	1.028000633	1.047381599	1.190567222	0.913869758	0.945533493
Q80YV4	1.021088436	0.993498122	0.979107486	0.976825996	1.005169347	1.012827282	0.984414257	0.97345484	1.001045498	1.118059427	1.001302688	1.012446044
Q8BYN5	0.999198354	1.072536646	1.041926134	1.04523008	1.097795822	0.972004847	0.914750826	0.972302847	0.950181567	0.944410975	1.044925404	0.959769342

Q05186	0.943382778	0.888745875	1.040453899	1.095842411	1.015159204	1.044283778	1.002345127	0.963982953	0.975922008	0.907661141	1.002036374	1.114165717
Q8R3F5	1.020599918	1.015394668	0.987533352	1.03174779	1.036217576	1.026104015	0.994055663	0.96650113	0.997963959	1.034521482	0.960327251	0.986851904
Q9ESB3	1.262156264	1.436684052	1.370307134	0.687952561	0.642383668	0.670819471	1.322555164	1.142211832	1.213686788	0.805398359	0.681347553	0.632828184
Q9R069	1.245491354	1.091108407	1.143267134	1.138341866	1.048786277	1.008489682	0.85543669	0.918554891	0.907686599	0.852249664	0.907454202	0.93202893
Q9EPR5	1.050711879	0.965812438	1.017600361	1.032101002	0.953189473	0.988105955	0.98885437	1.005421577	0.966574229	1.102766184	0.968644941	1.044458461
P24288	1.152660661	1.123625992	1.085555728	0.937546231	0.889328608	0.964211206	1.027667849	1.055372693	1.058764713	1.020714715	0.86885446	0.853575458
Q5QNQ6	0.93077958	0.919111133	0.931988141	1.004909818	0.990518984	1.010155744	1.047180765	1.022140699	1.00217042	1.065184826	1.024766093	1.089386614
O70252	1.070502837	1.128257578	1.032334428	1.011472759	1.04151485	0.959450165	0.970229728	1.00670637	0.96945831	0.904155278	0.973219952	0.937247218
Q9QY42	1.129295107	1.150991684	1.09126243	0.992821986	1.102598241	0.965903376	0.936889093	1.064178851	0.993041883	0.946596559	0.893522274	0.763357435
Q59J78	1.075553521	1.058952142	0.942391934	0.926351412	1.117816082	1.028709906	0.965323387	0.967460966	0.99131976	1.176431352	0.963740865	0.90920635
Q9WVQ1	1.041524816	0.984142768	0.952308885	0.930298571	0.987874489	1.019433639	1.038622605	0.95413552	1.038244396	1.187072728	0.984598674	0.981654317
Q9JM13	0.946637197	1.002691665	0.97614907	1.03757812	1.047532916	1.022219819	0.98424849	0.995572576	0.957394826	1.011773122	1.04458941	1.003947141
Q9CWM4	1.008986834	1.139123017	1.022473252	1.049445778	0.974732955	1.041953343	0.985733701	0.993060232	0.999865006	0.816347581	0.945667171	0.988265223
Q99LE6	1.007427211	1.012202459	0.978230066	0.990838304	1.021823345	0.990924422	0.992600904	1.002318823	1.030392551	0.992788392	0.993493054	1.008360002
Q9DC61	1.047388877	0.985674915	1.038516538	1.027982023	1.06041527	1.045932767	0.965807259	0.957292169	0.958633253	1.10015773	0.930369684	0.982055823
Q60649	0.998156097	0.986795179	0.979835249	1.005705173	0.992885503	1.016352373	1.005934085	1.028803156	0.994224971	1.065735172	0.982622474	0.996921934
Q8CHU3	0.878028242	0.94939697	0.964986044	1.086446926	1.032683962	1.063552936	1.009598244	0.98043253	0.994929931	1.10241778	0.957806813	1.05439583
Q9D898	0.913877337	0.975473147	0.980323623	0.998916442	0.944594413	1.025104048	1.029749043	1.009384261	1.03376475	0.899963733	1.088476504	1.047176922
Q810A7	1.265872045	1.256631359	0.886832989	0.74728987	1.033284281	0.974079656	1.003169184	0.948506641	1.211895583	1.353861648	0.755615474	0.767969893
Q80TH2	0.978546177	0.992259352	0.971156078	1.0082883	1.038682045	1.007875752	1.002552488	1.016475743	0.978576735	0.964433568	1.023531055	1.027625078
P60824	0.906047571	0.977628513	1.049756275	1.016843916	1.125893782	1.068140014	0.985269445	0.967257113	0.949012258	1.100354286	0.945296563	0.98868905
Q3V0G7	1.131514598	1.097685846	0.882717087	0.877048463	1.139719768	1.077909453	0.879554019	0.923359824	1.084220452	1.422554883	0.895929095	0.831287142

Q921W4	1.013432927	0.986312833	1.0404762	1.048331028	0.957968785	0.991865164	1.011508207	1.052952579	0.996480464	0.92652179	0.981675139	0.984092346
Q8K4X7	1.033465262	1.025801331	1.007575229	0.981536203	1.050807195	0.988087786	0.968279551	0.99295257	0.956354834	1.024134868	1.026721353	0.987827146
Q8VHR5	0.94640897	1.032009174	0.985207543	1.016975767	0.974039821	1.026962326	1.045138019	0.987278299	0.961960322	1.016733906	1.03975899	0.988204481
P31725	1.29858327	1.333015356	1.420316349	0.598141747	0.607561485	0.621785126	1.407738358	1.364679988	1.222902299	0.596329786	0.651668683	0.625562974
Q80WG5	1.040114292	0.994145109	1.023112211	1.012857754	0.999367409	1.005298829	1.006984896	0.999680868	1.034148259	0.960474874	0.980279511	0.951439522
P70290	1.022871624	1.010540568	1.038162572	0.980642735	1.021921874	1.000415308	0.998314041	1.041955298	1.044915541	0.930018002	0.946553453	0.956635144
Q9WUD1	1.004407259	1.045991167	1.012901059	1.065951137	1.004912444	0.984942778	0.907547096	0.98634717	1.050914646	0.874701604	0.992358638	1.05320314
O35405	0.918734455	0.942157403	0.942156677	0.940126035	0.952223387	0.914457774	1.095103325	1.040947891	1.030436291	1.111530249	1.079229807	1.055686084
Q91YD9	0.948541932	1.013886002	0.933008802	0.990762506	1.110818927	1.075039378	1.005459777	0.938644032	0.990289388	1.117741866	0.960564455	1.007164041
Q8CI51	0.955955657	0.979268548	1.021299267	1.033287618	1.052162118	1.019789974	1.038746569	0.979053435	0.962147756	1.02560744	0.996466742	0.971948204
P25976	0.98414805	1.00171816	0.993273564	0.994176807	0.966916458	1.008145642	1.009339604	1.020031829	1.000723731	1.003805032	1.017182726	1.017379241
Q80U19	0.961430009	0.995181567	0.998248955	1.050231702	1.124602052	1.148229425	0.95845179	0.950490446	0.899226232	1.016659636	0.98101501	0.983473019
P57776	0.957624332	0.937445807	1.003430899	1.007056861	0.98327945	1.037981871	1.003649199	0.998198853	1.039565441	0.969348088	1.008424977	1.053579367
Q91WU5	0.995580863	0.933653067	1.034584756	1.019299275	0.948730261	0.978076603	1.000496323	1.085827386	1.000270985	1.030842073	1.009673966	0.984297062
Q68FD9	1.013272539	1.012912289	1.005440045	1.008819981	0.976954328	1.033463399	1.003454609	1.000529511	0.983142691	0.961089715	0.984983642	1.03206341
P54726	1.010440091	0.97027657	1.023705186	0.993114838	0.9865467	0.979560159	1.044437057	1.042525411	1.024276051	0.892496087	1.004419617	0.992151836
Q8C3Q5	0.918924751	0.977271085	0.957127486	1.026471952	1.003777045	1.002574128	1.024427895	1.047266201	1.014617471	0.985963887	1.006097961	1.040850328
Q80TM9	0.988666864	0.997551671	1.001512517	1.000666358	1.014694213	0.946854861	0.996091565	1.029990023	0.942436855	1.097666258	1.06706315	0.971660286
Q9JIY5	0.961484504	0.973173456	1.009745323	0.996587106	1.011342224	1.017201307	1.007014568	1.01397819	1.028598714	1.019586015	1.012579757	0.965342271
Q9WTQ8	1.012181419	1.011890108	1.014132935	1.009468284	0.996969916	1.011233246	0.983727669	0.99639013	0.992788435	1.048892272	0.981603077	0.992531469
Q9D1D4	0.964734206	0.919612106	1.014642794	0.989125474	0.936867221	0.953232177	1.068119375	1.052117015	1.060506022	0.965259104	1.016127515	1.034722332
P45878	1.033364652	1.043839816	1.042401516	1.042973367	0.980393276	0.951775554	0.965994455	1.029860128	1.000980939	0.896629893	1.005096169	0.990418274

P0C027	0.97667971	0.981722989	1.012777758	0.93780137	1.000664858	1.017981942	0.992050113	1.016524164	1.042778443	1.052959827	1.001845721	0.99526692
Q9R1K9	0.995245153	0.997023058	1.028051161	0.954896687	0.986980052	1.072515021	1.017809549	1.029832593	1.031011359	1.010905552	0.946556523	0.951482915
Q8R307	1.105430855	1.005523745	0.983666189	1.000610491	1.105269943	0.973131526	0.894921068	0.965445135	1.024240888	1.182537173	0.94006269	0.957298031
Q61016	0.995673726	1.095106564	1.045215307	1.127459037	1.068506856	1.080795086	0.867324963	0.930463715	0.910035856	1.005394142	0.980346616	0.968920144
P58021	1.008415942	1.012601568	1.021641987	1.012300964	1.020292015	0.990486988	0.982504133	1.008442791	0.983502636	0.943197804	1.024626469	0.992491099
Q9ERB0	0.967257879	1.010222811	0.95188247	0.998889262	0.989949596	1.018339309	1.021654727	1.023993962	1.003627767	0.987022835	1.015742323	1.023069746
Q9DCM0	1.322252241	1.240828024	1.020848853	1.052220769	1.047390107	0.90734066	0.835927606	0.901068386	0.93786179	0.740641592	0.991383738	1.00281416
Q64444	0.99925719	0.958884297	0.950833723	0.963672967	0.956987253	1.018867566	1.068533595	1.00353278	1.063627496	1.012078116	0.985854403	1.032185448
P17225	1.151142676	0.953400847	1.021992901	0.924918267	0.97623982	1.02883701	1.047021979	1.004200415	1.011650615	0.993165678	0.947983109	0.972528988
O70311	0.983510625	0.98269806	0.999462292	1.04267179	0.982702422	1.035997278	0.990313286	0.976131499	1.008289152	0.983560372	0.992939054	1.044926298
Q922Q8	1.008543508	1.01934055	1.02183363	1.011967496	0.952664064	0.952131235	1.012588908	1.002170148	1.010816302	0.895902385	1.049853194	1.028582185
Q8BQZ4	1.042340793	1.012925034	0.982733683	0.95540766	1.02412631	0.97182526	0.995935953	0.958835173	1.046229394	1.043841462	1.009355814	0.996713141
Q8R0G9	0.970322931	1.043582682	1.048039928	1.010521478	1.013249116	0.923639646	0.954703755	0.986715467	0.99594335	0.91982954	1.070274302	1.040876682
Q80XU3	0.94844269	0.941618645	1.034812055	1.026639035	1.030845775	1.055120618	0.986631571	1.026545429	1.017851274	1.180790633	0.919111773	0.934220736
P70202	0.956663633	0.981534778	1.047562822	1.033457295	1.04251372	0.977802898	0.958989681	1.026400133	1.028566782	0.924879032	0.998777657	1.008708301
Q8BYW1	0.915306598	1.032723165	0.94893858	1.059536962	1.00734515	0.968183664	1.001206562	1.028369844	1.014575346	0.914784952	1.065121261	1.014724498
P97371	1.034495188	1.104563773	1.059057253	1.021577683	1.034204804	1.028793658	1.038237953	1.009823089	0.964475965	0.90697026	0.900048333	0.904831933
Q8VHK5	1.094429687	1.003956745	1.012391554	1.038219666	1.068857283	1.064005036	0.91335493	0.955871567	0.989043795	0.934804961	0.984341008	0.973523299
Q7SIG6	1.020546147	1.005452076	0.934985371	0.961667998	0.997733277	1.026598324	1.051833399	0.933581089	0.98112976	1.068932062	1.028342674	1.046117935
Q505D1	0.994467602	0.944496208	0.978842918	0.99701539	0.971842267	1.016931113	1.02450537	1.017444056	1.01003742	0.955328458	1.031793487	1.052384512
Q8K0D0	0.950811149	0.996852815	0.963727584	1.03621837	1.057657695	1.049564222	1.010106765	0.985621425	0.942654013	1.017391235	1.001870228	1.032384928
P62900	1.029585233	1.060191173	1.01850224	0.988543373	0.974678744	1.023998011	1.076898199	1.022616501	1.040839788	0.802181658	0.929636527	0.972706705

O54833	0.908293159	0.870448752	0.99304601	0.997545048	0.993450247	1.007280296	1.04795331	1.010122079	1.033406453	1.104469715	1.051685061	1.015630214
Q6NV83	1.03227474	0.995165973	0.960847585	0.957118025	1.012257041	1.076480324	1.003489775	0.967125735	1.027501374	1.114261411	0.980100143	0.950653747
Q921M4	1.000788524	0.998489968	0.987747756	0.961615976	0.96938774	0.973417956	1.034595074	1.001918223	1.029975095	1.005401414	1.025222607	1.020782665
Q06138	0.975232684	0.976158936	1.03157779	0.997825928	0.923264309	0.993039737	1.030067025	1.029194141	0.930704592	0.974418335	1.075448448	1.059247854
Q9CQR2	0.979474427	0.98833304	1.027939152	1.002801953	0.926659907	1.018645152	1.039481328	1.007393481	1.02964186	0.927663839	0.968752739	1.068596571
Q3UDE2	0.989431952	1.034948365	0.97860019	0.980533447	0.97835559	0.954716768	0.968464961	0.979486069	1.007621533	1.053059538	1.043070416	1.072653675
Q9WTS4	0.918483104	0.990225319	0.991692516	0.924764034	0.959353921	0.943647622	1.038731082	1.109010539	0.988062613	1.055262475	1.054977781	1.032612217
Q8BHE3	0.930841874	0.971329929	0.937100047	1.047902838	0.940635875	0.961998894	1.024610403	1.074894323	1.01144873	1.036498974	1.027052433	1.057406029
Q80YX1	1.088538047	1.042698439	0.968086058	0.958621757	0.891087546	0.909757299	1.041718754	1.084365978	1.001785585	1.041196326	0.985126741	1.023505702
Q8BVL9	1.005048855	1.021317039	0.968706197	1.00316038	1.038879252	0.987056931	0.990377357	0.995180351	0.978500449	1.00565518	1.043065307	0.990865667
Q99LS3	0.942710354	0.990695806	1.008467851	1.005182754	1.092197153	1.041620878	0.970916531	0.960004859	0.974473446	0.935686121	1.027351278	1.053088697
Q9CYN2	1.006072298	1.010610815	1.045005384	1.023120613	1.012101356	0.963307319	1.034960595	1.071617257	0.997039385	0.995108714	0.951394464	0.907142471
Q8BPQ7	1.014758998	1.125678435	1.008334265	1.073085837	1.06758089	1.152153969	0.912955788	0.920682731	0.943085961	0.896451059	0.965194591	0.946818362
O88271	0.955580706	0.938160161	0.986989702	0.987201451	0.987818433	1.030549346	1.058114473	1.021896721	1.020319109	1.159807979	0.956232183	0.977178911
P84244	1.0384182	1.100593843	1.043610668	1.14495852	1.042426248	1.103695686	0.951806658	0.982203143	0.922912469	0.846295671	0.93630419	0.893798182
Q8BMJ3	1.009069997	0.98302404	1.035418171	0.992451143	1.056097961	0.926856591	0.99621796	1.023232254	1.008194881	1.02823022	0.990005565	0.983340006
Q9QXG2	1.020747029	1.00133211	1.007477402	0.999879029	0.982346536	0.969136571	1.017439737	1.039964882	0.996203924	0.980777564	0.978375121	1.023225668
Q3UMU9	0.940802342	1.032069107	1.009526681	1.077923884	1.02088966	0.944301485	0.969613344	1.028758591	1.010458753	0.983977563	1.006994226	0.987587297
O08692	1.536328326	1.110875933	1.455606734	0.529539016	0.546885232	0.542627673	1.331507808	1.383541889	1.329183493	0.745120211	0.65478716	0.647007246
Q8BPU7	1.076122761	1.095433765	1.028061962	1.060955992	1.118906784	1.121458308	0.920544307	0.976158421	0.918258785	0.972720972	0.893164927	0.893151126
P14602	1.191936398	1.065674464	0.99731837	0.945655946	0.981830358	1.014142538	1.00099283	0.968260786	0.970471929	0.998482537	0.966645968	0.954095246
Q9EQZ7	1.152669761	1.121431528	1.071625944	1.170150128	1.178629516	1.129726803	0.860673208	0.856314029	0.868316465	0.947159856	0.891092222	0.857980937

Q91YH5	1.061922399	1.043060849	1.025016301	0.994955169	1.031978251	0.993613722	0.949436846	1.013722565	0.993764875	0.927269256	1.008906163	0.959717563
Q9R0Q7	0.97376295	1.028032185	1.047088677	1.094403146	0.961038143	0.899889362	0.909193268	0.964630359	1.084529908	0.711726318	1.042464645	1.182900311
P11835	1.049867381	1.002428875	1.052580231	1.008482728	1.005120852	0.959065976	0.993090611	1.027727743	0.949516506	0.994996129	1.018760782	0.963417901
A2AHC3	0.99242317	0.969726717	0.951483661	1.001489892	0.952202146	1.009278079	1.050904739	1.02937317	1.020293052	1.080646673	0.985126793	1.007136472
O55137	1.03841057	0.990090079	1.002559305	0.996534202	1.042684192	0.947296483	1.012004767	1.282226082	0.998093284	1.014547144	0.918108483	0.776209627
Q9D404	1.012993786	0.955123569	1.013675597	1.035798311	1.03827435	0.968926464	1.017948313	1.013787916	0.987575115	0.981221998	1.022779971	0.965324065
O89086	1.009452727	1.036691031	1.060710798	1.02496218	1.181171147	1.108670296	0.961019531	0.945828832	0.874969788	1.03627577	0.954560011	0.887329445
P97441	0.826416388	0.964185886	0.986411613	0.994658326	1.092698409	0.958526763	0.982442045	0.982209787	0.866625249	1.237391257	1.17235276	1.031220694
Q8K2C9	0.985949024	0.944384487	1.040299551	1.002342296	0.976054121	0.959165653	0.988786117	1.014064325	0.981535827	0.964814553	1.092414592	1.038290007
Q5XJV6	0.978716033	1.000816963	0.984351399	0.995487664	0.952948243	0.98548134	1.0515817	1.018290389	0.972915305	1.098842394	1.013582725	1.001854539
O08583	1.022257015	0.994650328	0.971621717	0.97185085	1.023293794	1.015393279	0.981840824	0.992159624	1.042149912	1.169750326	0.96077156	0.954055119
P21126	1.004293244	1.094763166	1.030963636	1.098730534	0.946791929	1.046989403	0.934375774	1.000424711	1.040898234	0.896715951	0.887382156	1.028326905
P59326	0.997728743	1.003514567	0.979875995	1.031713745	0.992515142	1.062640324	0.981037365	0.95421405	0.982563487	1.00178447	1.023133404	1.024189114
Q9JLI8	0.979113933	0.984495243	0.96818028	0.970517291	0.980418032	0.982937329	1.009636848	1.031617767	1.049135915	1.030042124	0.993945155	1.043779484
P70452	1.142610728	1.054652917	0.972001938	1.005178168	1.071706415	1.146565853	1.003339199	0.946198191	0.89504864	1.065376805	0.91687565	0.89257928
Q6IRU2	1.285228429	1.156876438	1.014333294	0.9144468	0.917945173	1.122071085	1.015477449	0.969359159	0.958785044	1.140486979	0.831984506	0.816553506
Q04750	1.049228862	1.011839626	0.998634488	1.032400325	0.938671041	1.03218762	1.014300823	0.977844835	1.021349449	1.016521217	0.944251845	1.008494748
Q8CFI7	0.958312026	1.031377548	1.009351722	0.998240613	1.055807637	1.014819328	1.012951251	1.066837873	0.921095594	1.082734136	0.995967335	0.910769885
Q60714	1.040361183	0.983059291	0.988285801	0.969788721	1.035887436	1.038775466	1.003319321	0.962040564	0.968799561	0.997125005	1.021379985	1.024462214
Q99MR1	0.928244203	0.976746798	0.973516368	0.998069064	1.002394496	1.006616139	1.009951153	0.96611514	1.004843395	0.992296213	1.078114568	1.065160159
P51432	1.024612272	0.943267952	0.96431939	0.999984279	0.839972334	1.043651894	1.09372672	0.9658256	1.174643242	0.968917809	0.892761082	1.087687518
P24547	1.056682735	1.021058313	1.004185395	0.991825875	0.991809011	1.030024856	1.005216128	0.988349027	0.995018065	0.993462132	0.968823317	0.988342793

Q8K4Z5	1.046040569	1.027221989	1.017478639	1.01864571	0.9701229	1.00975595	1.010518884	0.996935375	0.992832634	0.94796027	0.959210257	1.019702207
Q80ZF8	0.884639497	0.958572386	0.929037939	1.010256011	1.000522745	0.950790512	1.007557847	1.015774057	1.019586393	0.985782621	1.139034209	1.078061241
Q91ZR1	1.000028206	0.961619302	0.992238656	1.011480041	1.031898297	1.069409422	0.983035759	0.938610257	0.982824597	1.010304413	1.008705653	1.05214937
P47915	1.123628648	1.078917995	1.082445601	1.07408477	1.029924861	1.009420068	0.947607278	0.967704988	0.998158954	0.918232686	0.868756041	0.940401219
P62075	1.046841763	1.060564354	0.999356557	1.038130495	1.019825001	1.032036862	1.007120357	0.96866759	1.060530889	0.802453887	0.941167544	0.980838245
P17918	0.912313166	0.937952775	1.017081085	0.978230009	1.424172619	1.004509358	0.936768923	0.903107094	0.765152141	1.504542379	1.040405039	0.850130036
Q9WU84	1.016081997	1.024272586	0.992640407	0.991557978	1.049700047	1.064094765	0.946557923	0.951685421	1.010268083	1.048799438	0.953051186	1.019270233
Q61730	1.053757943	1.043263502	1.054037887	0.943188935	0.978290048	0.950860894	1.02754561	0.97426736	1.109512259	0.840670497	0.977847435	0.986852398
P62852	1.258786903	1.233445401	1.147357881	1.162063637	0.846360398	0.986866759	0.932360952	0.970996581	0.979207189	0.79933114	0.804810345	0.895187649
P70280	0.986811025	0.942094164	0.992257644	0.997951239	1.05430331	1.063599079	1.018349201	0.964227637	0.940260323	1.168374286	1.003968263	0.971687162
P61514	1.068484114	1.096367322	1.05970197	1.08273105	0.940518391	0.908078011	0.989302988	1.038641098	1.073574209	0.739853728	0.975300817	0.940084046
Q3TDK6	0.968608725	0.986874886	1.003957777	1.055307497	0.944993648	1.090401889	1.02992519	1.035110971	1.058258912	0.856819267	0.924229771	1.010367125
P97798	1.003791966	0.965519002	1.001089286	0.962049075	1.017857562	1.03310016	1.009183828	1.022466078	1.013281482	1.073054372	0.957409385	0.996868298
Q99KG3	0.986711317	0.984021435	1.00700608	0.978738214	0.966625939	0.97422482	1.004403372	1.091377297	1.042726277	1.004960029	1.000080006	0.962702649
O70591	0.949338171	1.081408595	1.003678605	1.03471309	0.93368815	1.118524028	1.021215493	0.962623407	1.060017069	0.811902743	0.947692508	1.02127568
Q8BV13	1.066913165	1.006907395	0.992154577	0.906740945	0.916483837	0.954858134	1.100069759	1.076966702	1.019997838	0.938996022	1.018686467	0.971940518
O55142	1.025506751	1.01004778	1.078034992	1.023586063	1.044330689	0.989837081	1.018675597	1.040192475	0.967496811	0.9409656	0.932882936	0.939221176
Q6ZQ29	0.985249252	1.010654135	0.986246374	0.9979045	0.963513424	0.960393391	1.094957777	1.018043851	0.946434878	0.981150164	1.067751583	0.98363975
Q9CRD0	0.9555068	0.972338386	1.009544206	1.073216062	1.063290263	1.08009648	0.977528502	0.955473244	0.929371326	0.955034439	1.015348183	1.038610068
Q8R2U6	0.876694446	1.064778849	1.00745311	0.91275408	0.937712276	1.085746776	1.106689787	0.972163513	0.930545398	1.046285118	1.093889413	0.967425109
Q5EG47	1.029499496	0.988784086	1.006253963	1.008923646	0.970741242	1.043872897	1.027105796	1.015074279	1.02315034	0.960209355	0.950521723	0.988405559
Q9D8S4	1.006906663	1.018226368	1.01872253	1.096998734	1.000455485	1.070631975	0.986235352	0.996889099	0.997157212	0.862555395	0.933462518	1.003785138

P63034	0.94434591	0.971678432	0.952450191	0.975973181	0.968053637	0.958557714	1.056931082	1.034953571	1.000473764	1.055827962	1.054965564	1.046902314
O35691	1.05968943	1.017082363	0.955301484	0.95162365	1.027335978	1.098380778	1.117139792	1.005372092	0.897334488	1.178064373	0.890885126	0.932020438
Q9QYY8	0.959424114	1.542580917	0.892879665	0.980004891	0.990994169	1.011218066	0.901936006	0.946525817	0.87504509	0.962773954	1.08423118	0.884019798
Q61627	0.945587342	0.990490917	0.993152256	0.981833108	0.981859273	0.963602362	1.003857487	1.025325478	1.009847657	1.085714766	1.042326539	1.014132351
Q9CY27	1.073723228	1.027546512	1.063138383	1.068786915	1.022235268	0.983440112	0.903740908	0.958230013	0.993912119	0.770892157	1.01326314	1.076357748
P17665	0.9906463	1.055006049	1.096299883	0.958861654	1.004775576	0.928774284	1.004945675	1.021730813	1.069632508	0.902499847	0.975618319	0.951128291
A2AHG0	0.87471445	0.920591936	0.94970955	1.032130189	0.972660755	0.988707439	1.029110205	0.955293941	1.00829651	1.005057298	1.146228212	1.106985894
Q9Z2I2	0.999715306	1.042607092	0.989080272	0.974348591	1.005525357	0.940278324	1.036261967	1.017912245	1.045559716	0.997285893	0.994280402	0.96543229
Q7TT37	1.000649031	0.957536084	0.976677598	0.966223548	0.972623828	1.001508627	1.016806611	1.013506754	1.081779855	1.036002459	0.977054629	1.025491242
P68433	1.131374876	1.074536315	1.119481821	1.111984333	0.945284881	1.108075651	1.046836238	0.991668978	1.014716684	0.673028845	0.829862209	0.881580776
Q9CQR6	1.045308368	1.023803546	0.99158067	1.065702432	0.982597269	1.037912927	0.970855322	0.990240787	0.988579515	1.009274577	0.957080159	0.9907137
Q9JKX6	0.887616178	0.953773011	0.946576351	1.105362472	0.893336696	1.022091897	0.963985286	1.015830992	0.986759114	1.062436444	1.057889167	1.146562748
Q9WUA6	0.960704416	0.998015944	0.944690309	1.010767694	1.037324488	0.984232811	0.978625287	1.001181451	0.920542458	1.165196481	1.071340322	1.022797911
P14106	1.059551859	1.075494739	1.101422622	1.078633025	1.104789726	0.981658253	0.885309047	0.938098096	0.933947132	0.848485286	0.992880938	1.00124919
Q3THW5	0.985761579	1.237678487	1.142338213	1.138273018	1.23112184	1.030415727	0.887099743	1.000659124	0.76986661	0.763091522	1.006721002	0.784926242
Q6P5F7	0.923325928	0.965288329	0.953980814	1.010084993	0.926807002	1.001634988	1.026318346	0.949100081	1.051553544	1.08073306	1.075338902	1.065157951
Q9JKS5	0.981399412	0.967582031	0.959644465	0.990543167	0.962427184	0.992464617	1.033782756	1.037905483	1.006069083	1.051689437	1.004701634	1.048091463
Q9JIS8	0.962617866	0.966555052	0.982667571	0.975571432	0.868500928	0.918226372	1.012749765	1.008456121	0.98660848	1.128324876	1.061331055	1.18576076
Q91YI0	1.024080849	0.977672554	1.061969269	1.025363399	0.972790206	1.063726076	0.999357297	1.004415289	1.004089695	0.959884068	0.92494815	1.002690541
Q91WG2	0.963954395	0.988073159	0.969038187	0.949544445	1.015544913	0.987029729	1.033104599	0.986429042	1.012179053	1.048852988	1.008278517	1.072861116
Q9CQS4	1.006496155	0.948708317	0.96731264	1.005287686	1.056368679	0.990586101	0.948107705	0.963747707	1.031000626	1.14596177	1.020974678	1.004850898
O88845	0.979825519	0.954903826	0.983505178	0.99190368	0.981037043	0.999613375	1.017106571	1.001734557	1.013497917	0.99352118	1.040603847	1.050696883

Q3TY60	0.9863	0.9989	0.9976	0.9996	1.0329	0.9656	0.9730	1.0386	0.9846	1.0506	1.0336	0.9767
	49663	50713	45654	52387	45985	5783	98191	12199	9564	85801	42209	36547
P23492	1.1112	1.0208	1.0246	0.8969	0.9136	0.9075	1.0975	1.1215	1.1292	0.9867	0.9063	0.8724
	58376	46645	78912	05193	94205	37329	48669	33946	29171	37776	07093	21687
Q6P9R4	0.9810	1.0281	0.9799	0.9607	1.0127	0.9569	1.0477	1.0535	0.9664	1.0882	1.0051	0.9690
	41263	29425	93893	27028	10281	92299	01309	68532	78299	18795	65658	74968
Q99KP6	0.9788	0.9486	1.0016	0.9911	0.9764	0.9441	1.0113	1.0293	1.0511	0.9643	1.0474	1.0388
	08801	47003	30017	83386	04213	64988	09742	51959	36275	23447	19276	55646
Q8CJG0	0.9786	0.9648	0.9982	1.0168	0.9406	0.9758	1.0173	0.9770	1.0300	1.0468	1.0229	1.0618
	30114	65187	77668	81134	04231	63967	81154	0877	24099	3665	67594	32856
Q9R118	1.1788	1.1422	1.1303	1.5803	1.3442	1.3572	0.7639	0.7891	0.7138	0.7411	0.7043	0.6915
	68234	92456	12041	66307	26333	90606	18407	80713	59442	80147	25924	21108
P31750	1.0385	1.0065	1.0639	0.9982	1.0387	0.9437	0.9631	1.0225	0.9413	1.1110	1.0062	0.9431
	35107	08739	32338	14971	0373	53155	94733	68365	99004	42342	21024	15836
Q6P5U7	0.9362	0.9246	1.0038	1.0581	0.9839	0.9766	1.0154	0.9833	1.0189	0.9311	1.0480	1.1003
	74299	73254	33345	80104	71886	97367	14666	31902	3255	42173	54927	99619
Q14BB9	0.9736	1.0606	0.9383	1.0138	0.9848	1.0106	1.0143	0.9891	1.0101	0.9947	1.0243	1.0035
	47932	30303	44669	78225	57726	78133	50767	71058	04905	90815	83244	96424
Q61187	1.0258	1.0534	1.0366	1.0547	0.9140	1.0146	0.9724	1.0381	1.0056	0.8076	0.9922	1.0327
	51305	81819	03763	24938	43378	84579	39162	10051	65292	02451	64384	51288
Q7TNM2	0.9623	0.9578	0.9361	0.9672	0.9749	0.9659	1.0442	0.9910	1.0584	0.9994	1.0611	1.0781
	66995	35973	16717	79229	39667	56352	26605	15365	84964	57872	81376	99791
P70122	1.0472	1.0034	1.0073	1.0073	1.0268	1.0574	1.0278	1.0137	1.0303	0.9334	0.9208	0.9335
	59505	94572	76356	50217	9095	07479	13696	1065	4559	03793	30731	75443
Q3UXZ6	1.0057	1.0425	0.9953	0.9869	0.9120	1.0272	1.0555	0.9541	1.0347	1.0134	0.9553	1.0460
	06623	26838	54425	23765	24954	55744	33756	60503	93878	71367	30956	49658
Q8BJZ4	1.0517	0.9900	0.9261	0.9432	0.9841	1.1499	1.1587	0.9893	0.8888	1.2445	0.8997	0.9269
	04622	80127	38097	70494	75241	48759	33725	45581	48312	79701	85839	57338
Q9JME7	0.9528	0.9520	0.9687	0.9624	0.9911	0.9685	1.0389	1.0197	1.0411	1.0192	1.0460	1.0431
	71774	18532	64521	55615	98213	59171	66492	56191	02808	93611	06024	94837
Q9D880	1.0496	0.9726	1.0191	1.0010	1.0081	1.0412	1.0507	0.9733	1.0159	0.9693	0.9446	0.9754
	20363	17634	23173	06846	39326	25554	33357	32635	67867	65263	78815	46743
P97950	1.0050	0.9761	1.0218	0.9856	1.0171	0.9954	1.0330	1.0415	0.9755	1.0391	0.9787	0.9684
	48115	52899	52102	96554	7737	79513	63723	69264	69986	16093	50455	15693
Q9CQ69	1.0706	1.1303	1.0865	1.0813	0.9400	1.0391	0.9824	1.0611	1.0180	0.6917	0.9102	0.9017
	26929	74353	05404	0856	15069	4504	65018	08533	20925	60837	52748	60031
Q9D964	1.1626	1.0665	1.1098	0.9354	0.9648	0.9278	1.0511	1.0189	1.0734	0.8696	0.9061	0.8822
	87803	96554	14984	97376	40173	94002	04154	49475	5946	89585	51445	66195
Q7TPB0	1.0327	1.0612	1.0330	0.9684	1.0279	0.9491	0.9679	1.0635	1.0152	1.0272	0.9694	0.9173
	64454	53812	91094	69258	10612	91371	15406	47431	48911	26296	28706	32134
Q8BHG2	1.0080	0.9359	0.9705	1.0400	0.9817	1.0696	0.9859	0.9978	1.0856	0.9600	0.9153	1.0707
	1855	59266	69263	64398	21957	36397	79182	70463	1408	37496	65671	90721
Q9D379	1.0764	1.0748	1.0105	1.0135	1.1036	1.0964	0.9427	0.9606	0.9116	0.9741	0.9762	0.9152
	78283	8274	982	60189	26191	25143	73675	89053	15715	129	26591	67464

Q62446	0.947604953	1.025644813	1.032707594	0.964921866	1.07907349	0.971398353	0.971869685	1.000338042	0.945878707	1.101518907	1.065404777	0.947209896
Q9CQD4	0.997615723	1.045447389	0.999473088	0.988536182	1.011036524	1.033007802	1.015045186	1.014816759	1.000226766	0.929063289	0.974775827	0.988174842
Q68FM6	0.927582288	1.021225756	0.946645896	0.992909721	0.97539688	1.005276639	1.023291412	1.051907803	0.997967301	1.063987218	1.016414757	1.012593742
Q9D9M2	1.046725782	0.996199453	1.025596672	1.084398686	0.919783879	1.015946877	0.948490929	1.005317554	1.000541764	0.948508927	0.981330252	1.047400841
Q810B6	0.959039022	0.966228838	1.03378362	1.029780968	1.013191187	0.973862598	0.952865799	0.991834529	0.996404171	0.976707364	1.065764072	1.043981417
Q3UH68	1.007944509	1.093320718	1.113345093	0.957299558	0.981290161	0.929990259	0.994434224	1.042863451	1.075279632	0.98038405	0.93015646	0.890611567
Q9JHU9	1.092339702	0.990140802	0.992071416	1.024295305	1.043612693	1.027970798	0.948976021	0.975994753	0.970762018	1.018849382	0.971337457	1.010195459
Q61701	1.123468959	1.023338083	0.973182972	0.92302095	1.026826832	1.042200187	0.99588372	0.950015298	1.045627556	1.054677593	0.940512185	0.969552006
Q8BGQ1	0.885092778	1.081560596	0.954916442	1.00306672	0.913479207	1.00788949	1.03144003	1.008624362	1.049124105	1.047135859	0.988142292	1.051880498
O35609	0.971298806	0.978090823	0.978202163	1.016916322	0.988203529	1.008928901	1.007693563	1.012608145	0.989628491	0.994696804	1.023922698	1.047507258
O35449	0.918275329	0.934012229	1.043615403	1.039559172	1.135641324	1.221357131	1.010373588	0.89364601	0.839576841	1.071127789	0.96955796	1.01607651
Q8CCS6	0.991205064	0.977844577	0.975511198	0.957973411	0.99730016	1.047581123	1.069343677	0.969641589	0.967262297	1.171615797	0.961178755	1.014435961
Q8CH72	0.956350961	0.987609833	0.994618873	0.990390753	1.010211209	0.968990143	1.029959587	1.02089654	1.010802059	1.027128903	1.024607341	0.995737378
Q8CIP4	1.069853415	1.061749244	1.004417916	1.029595085	0.968931323	1.048925213	0.993348671	0.966240503	0.934608779	1.030353763	0.947744575	1.016374026
Q80TI0	1.014516867	1.000701424	1.000380881	0.978005892	0.968725132	0.981209109	1.007457666	1.010050016	0.93377705	0.974571537	1.051053407	1.093375654
Q3TTY5	1.022700556	0.993551471	1.034369106	1.014973745	0.92048074	1.007716982	0.971171179	0.938891454	1.129319729	1.276924237	0.932567282	0.888990886
P55258	1.010048117	0.982658344	1.007267149	1.002625879	0.978966451	0.97662869	1.014234668	1.042075723	1.019078775	0.947890803	1.012839589	0.99680883
Q8R4C2	0.974403925	1.032958223	0.990535996	0.96951538	1.02373706	0.947714451	0.993699398	1.025904 2	0.967445288	1.054676302	1.06696556	0.984286396
O55135	0.98956454	1.010368427	1.016857914	1.045244048	0.952699338	0.883515999	0.93027033	1.046719462	1.085808232	0.851959495	1.059076884	1.068194549
Q8CGM1	0.935651972	0.98865376	0.958065443	0.939866779	0.970432916	0.945309065	1.023212745	1.050579025	0.988587409	1.037734311	1.083730823	1.087392726
P20065	0.795883572	1.258888022	1.120241981	1.37418562	1.103635295	0.960865443	0.824573864	0.991558327	0.984445689	0.872252542	0.708553909	1.058191667
O35345	0.987711898	0.977088948	0.986804191	0.990622261	0.950041444	1.014564121	1.054147764	1.000010819	1.006603517	1.025536656	0.979045488	1.058080224

Q91VR7	0.966364855	0.939539762	0.975464672	0.957450291	0.948449757	1.00207516	1.081798369	1.054133704	1.054582964	1.052105116	0.978768363	1.008552133
Q8CIB5	1.001333291	0.942644731	0.97308342	0.967738915	0.967119571	1.09961128	0.988099528	0.943010858	1.01109208	0.999000333	1.047523674	1.081324426
P12815	0.99636134	1.048760544	1.04761618	1.043718852	1.081617242	0.999675565	0.916906139	1.000646702	0.994658581	0.880782764	0.987965543	0.991530264
Q8K1L5	1.011601226	1.027803826	1.043176529	0.961441111	1.04607276	0.952324374	0.977777596	1.063480312	0.955226039	1.050379636	1.035528534	0.909853013
Q7TNG5	1.093711957	0.999881506	0.991868858	0.958076882	0.950592138	1.036247825	0.985118046	1.021798655	0.979693128	1.065401517	0.997500352	0.981346467
Q9DBL1	1.026507828	0.981827727	1.03272773	0.974670232	1.03661234	1.050269217	0.986833511	0.990764636	0.976089348	1.063890412	0.982526019	0.956406804
Q9DCB8	1.029083584	0.963671139	1.005233314	1.039624575	0.960088241	1.044353469	1.010772241	1.075291269	1.014422498	0.921288198	0.936556392	0.999263781
Q61140	1.004327667	1.017396087	1.051336886	1.023791098	1.022460442	0.938213184	0.929164862	1.083971759	0.976877329	0.952450473	1.038722129	0.958937647
P06330	2.203461506	2.348992294	1.537953106	0.831529617	0.670850885	0.641985529	0.606515234	0.833417726	0.694090705	0.546347089	0.585659374	0.588742784
Q80TA6	1.042869986	1.062841475	0.922167282	0.931076224	1.083736069	0.999308464	0.930610644	0.974647527	1.012098742	1.195359825	1.010874601	0.953433208
Q3TIR3	1.032361627	1.169003598	1.116652934	0.853033421	0.763986903	0.779667997	0.981367029	1.184604161	1.41371628	0.784428161	0.835843554	0.939532236
Q6ZWU9	0.989804512	0.975131487	1.047438475	1.001533475	0.965158911	0.966807391	1.012625861	1.031759671	1.048518588	0.979362063	0.962766903	1.022966789
Q8BS95	0.986889534	0.967538216	1.00373614	0.981235363	0.967326911	1.05344885	1.025162486	0.985459156	0.963589629	1.125235529	1.029918457	0.97936317
B2RQC6	1.010989864	1.030255178	0.967006769	0.866631862	0.836939971	0.88813792	1.210139423	1.04133098	1.327530095	0.889043385	0.899360957	0.934014067
P16388	0.983261084	0.980467923	0.976761833	1.110030111	0.981155735	0.961599968	0.910076439	0.946986015	1.097859623	0.939993552	1.055523879	1.052094571
P40240	1.289396332	1.231239492	1.187245136	0.867286569	1.060529348	1.215972669	0.845723716	0.89548984	0.804350246	0.917587822	0.930799653	0.828109596
Q8C3F2	0.98810617	0.962227513	1.011779287	0.983336444	1.009640137	0.987190693	1.003086533	1.019953064	1.012932293	0.966214977	1.020809691	1.0338358
P62627	0.967998047	1.13752056	0.991930712	1.008728966	0.941821618	1.017310192	1.062803755	0.991336306	1.001581987	0.92581241	0.972979342	0.968280525
Q64152	1.017590728	0.961919757	0.925984591	0.979342674	0.963656468	1.039588367	1.055109903	1.000111073	1.062904484	0.978499179	1.040943609	0.970542503
Q9JHG6	0.889832472	0.986657432	0.944700794	1.028587109	0.998527768	1.039170746	1.050619621	0.973763457	0.995317225	1.087100541	1.017738592	1.037009207
Q3UL36	0.985621238	1.06823118	0.987580857	1.011434785	0.980424804	0.909377603	1.042915591	1.069178989	1.004733955	0.981634194	1.009201948	0.94933573
Q8C166	1.094432276	1.071074123	1.1271839	0.953011821	0.926293991	0.91622501	1.03122235	1.061548017	1.114834698	0.857527122	0.894441323	0.900482282

Q80U95	0.925450854	0.954353338	1.026166386	1.031811693	0.986986865	0.951393218	0.970319266	1.028880118	0.963391718	1.064518769	1.06697427	1.064616335
Q3TYX3	1.023613775	0.984717326	1.012536625	1.053403159	1.011009355	1.004140969	0.975116557	0.962620484	0.983095268	0.962449658	1.017607217	1.031020888
Q99KP3	0.928006756	1.001510129	1.012586581	1.082044429	0.982699028	1.029027616	1.003112295	1.00039001	0.992387884	0.96701592	1.004771048	1.003418925
Q9D0J4	1.064266263	1.059908888	1.003630613	0.984049052	1.013865163	0.966917521	0.988618347	1.012492137	0.997072984	1.008838383	0.983564531	0.953539997
Q2L4X1	0.993486376	1.019561639	1.036196241	0.934082712	1.003619241	0.968801811	1.044114035	1.049889709	1.066491232	0.952488676	0.945497515	0.970790741
Q9WVL0	1.110267351	1.053910884	1.053053666	1.076450603	0.930979525	1.090977845	0.974795071	0.933827221	1.046765329	0.792805503	0.887666127	1.026137119
P28667	0.920538645	0.911159008	0.954369588	0.950916491	0.905451587	0.879867385	1.079933712	1.106342877	1.112225501	1.020607225	1.048126858	1.082982887
Q9R0Q3	1.123400968	1.027591596	0.98289514	0.935288314	1.04999529	0.961615114	0.972087923	1.018227703	1.003390274	1.100837105	0.98176076	0.923404053
P18052	1.01367025	1.000626468	1.011958592	0.981790062	1.005970892	1.024107428	1.02367724	0.965486387	0.994446112	0.940818042	1.006233057	1.032121514
A2ADY9	0.953651235	0.975878164	0.979883444	0.99868419	0.979768854	1.010917658	1.050369542	1.015452998	1.03835234	1.000173489	0.995896806	1.00825169
Q99J39	0.908702886	0.955139192	0.922076932	0.952673031	0.942146823	0.944693776	1.035896952	1.081952609	0.987881233	1.088406263	1.135804405	1.058753475
Q9EPR4	1.0364113	0.979893833	0.993315936	0.948666213	0.950937974	0.96338476	1.028530899	0.984413947	1.026576701	1.047917885	1.031415678	1.0389379
Q3UYV9	0.938546938	0.942169144	0.987845742	1.025339692	1.033654379	0.981119562	0.984170344	0.962572947	0.977602645	1.080226636	1.096614297	1.033572257
P39688	0.868972824	1.013142832	0.991946319	1.121655172	0.953594442	0.890566916	0.863935954	1.018955909	1.11256102	0.797570419	1.157365461	1.10879789
P01887	1.236444863	1.15659643	1.117807218	0.894686081	0.861932094	0.902691019	1.176654136	0.970842049	1.162937163	0.804859082	0.799836676	0.853856714
Q8CGF7	0.935426331	0.975521366	0.984230272	0.984800118	0.973497465	0.977824914	1.049318316	1.010547491	0.997400045	1.082034279	1.014039896	1.056369886
Q0KL01	0.989752011	0.907604602	0.97658286	1.153435483	0.89644727	1.046129358	1.015042455	0.994723082	1.019150774	1.02939614	0.972973102	1.045864455
Q9D7B6	1.137687939	0.987292296	1.032906795	0.997590387	1.046165799	1.090498678	0.947498445	0.94292992	0.975499423	0.999656499	0.944003921	0.964391053
O08997	1.082172245	0.869556513	0.923435321	0.868479109	1.049131402	1.109061914	1.129090102	0.975777611	0.951695297	1.463620968	0.908819968	0.902816996
Q01730	1.135491705	1.053609113	1.034919602	0.954558315	0.962216633	0.981173826	1.018528506	1.048900253	0.997030209	0.942677922	0.941719002	0.94204116
Q91ZW3	1.001129494	0.957923816	0.934620812	0.932167344	1.049642285	1.022240202	0.98491762	0.93628447	1.02061412	1.345090839	1.001111504	0.985090674
P53811	0.937075593	1.054152151	0.991154952	1.053554506	0.992221782	0.986658912	1.005171715	1.004519416	0.884203383	1.17983837	1.028482074	0.989161

Q9QYE6	1.039989757	1.065613884	0.98855761	0.983860879	0.955189667	0.988900435	1.033184826	0.99872807	1.004315995	1.000375589	0.975802268	0.992969908
P46978	1.042917311	0.99433268	1.023759256	1.011161277	1.023323698	1.015902253	0.964479087	0.977713755	0.962123013	1.009781666	1.028353347	0.985396532
Q7TPS5	0.977524956	1.037219363	0.990568539	0.961971952	1.018624252	1.045314061	0.975814309	0.94870009	1.028366915	1.090387913	1.015452942	0.966533969
Q9CR98	0.938102135	1.025013626	1.058462911	1.100290152	0.924250307	1.016238183	0.95207668	1.023305736	1.065398183	0.690029418	1.017696082	1.070574703
Q61165	0.983252132	0.98941519	0.984171845	1.008676264	1.041113461	1.070477538	0.934145624	0.960216521	0.951345046	1.030510337	1.036515666	1.063330353
Q8BNN1	0.933689555	1.004769427	1.002768153	1.100456663	1.028375206	0.951689097	0.922856589	1.017577906	0.995420956	0.905339936	1.069061916	1.048885002
Q61545	0.961389231	0.997212542	1.031256163	1.103313164	1.160602362	0.909840306	0.898421534	0.94531541	0.88140813	1.338442826	0.980644346	0.99856159
Q08509	1.088108236	1.123501996	1.056442734	0.944875192	0.946266491	0.955320974	0.990938612	1.002183159	1.010077979	0.994222036	0.963541821	0.950051515
Q920B9	1.096439552	1.033343336	1.064077847	0.974088477	0.971644504	0.975296643	1.023637375	1.011459808	1.02259196	0.935790085	0.95084584	0.941748998
Q80TL1	1.006107781	0.997643997	1.003788745	0.999126811	0.925837184	0.956062114	1.044415428	1.091509718	0.984072128	1.013890954	0.994893741	0.999270711
Q9Z1N5	1.1190087	1.008240174	1.004166952	0.991644128	0.984443005	1.010656772	1.025419635	1.022819739	1.03321527	0.929111673	0.939165012	0.940445343
P26339	1.061921394	1.060462817	1.038353224	0.972008695	0.984215891	0.916488604	1.051614031	1.071754121	1.058038533	1.013916004	0.912612843	0.880457263
P63082	1.087551192	0.984693548	1.049806266	0.882248485	0.991483343	0.933608595	1.083506841	0.943855369	0.927675596	1.23631072	0.9850896	1.020363302
Q69ZF3	1.015134255	0.987335135	1.009841874	1.041606581	0.992505975	0.98418294	0.972524386	0.972643532	1.011761795	0.906276607	1.038404048	1.054718861
Q8K0Z7	1.021687206	0.959820562	0.970099018	0.980766559	1.022706812	1.063184663	1.059020925	1.009456861	0.954979273	1.013880649	0.965946617	1.021409783
Q62245	0.999832922	1.000899828	1.023970583	0.998174419	0.994655028	0.943972123	0.950839479	0.99744178	1.043765787	0.938945167	1.031390485	1.065978697
Q61234	1.0683636	0.982118757	1.00163712	0.992177683	1.022026151	1.097746534	0.962718013	0.933083974	0.985439241	1.003695843	0.97667488	1.028169106
Q8JZZ7	0.916049529	1.463952243	0.891561395	0.989519813	0.945431593	0.90569628	0.980474367	0.962580314	0.941310934	0.968651428	1.048238548	1.001189595
Q9CX00	0.931348684	0.983456591	1.000954638	1.030616732	0.948648208	1.002134822	1.020145304	0.982599058	1.000312219	0.994950259	1.020945357	1.095522669
Q9R0N0	0.977385382	0.957947933	0.942061811	0.936368059	1.131404381	1.053798863	0.985084113	1.035535942	0.963938129	1.171932949	1.002996492	0.941769756
P97467	0.994812585	0.992908097	1.079251166	0.920342545	0.985875443	0.988138624	1.023333025	1.014315671	0.979426172	1.034715696	1.015683933	0.990503096
Q8CI75	0.981463825	0.979813335	1.012846301	1.033222987	0.968994382	1.026482876	0.984863237	0.969694951	1.02155279	0.938146246	1.005044878	1.077174842

Q9Z172	0.982429105	0.944190511	1.031725263	1.096069757	0.981274742	1.111045179	0.982132349	0.986333912	0.974554173	0.927521859	0.937020852	1.06565722
Q99MB7	0.994888685	1.06850071	0.980449134	1.015074639	0.981551045	0.999522773	1.006377908	0.981495011	1.012289927	1.023093914	0.984013308	0.989992365
P29595	0.999698216	1.030068043	1.038728834	1.011108428	1.007294181	0.990770221	1.000710832	0.959438811	0.956575268	0.968567443	1.056681294	0.988111923
P84089	0.99763253	0.994995319	1.033356423	1.051195718	1.031640377	1.047885161	1.027802632	0.929934756	1.020895134	0.91409023	0.946589037	1.006402468
Q8VHQ9	1.056577488	0.963591182	0.974618796	1.00517391	0.940104108	1.038762815	1.055068337	1.011972707	1.048765503	0.935809624	0.940654591	1.032095154
Q9JJ28	1.067030083	1.030223756	1.026983544	0.979960419	0.996188858	0.993221311	0.989144633	1.013149204	0.967471132	1.004853378	0.984400637	0.985490635
O70378	1.106001661	0.996797952	1.017836468	1.052335806	0.986694485	1.0698472	0.983891739	0.955073293	1.006767939	0.899692674	0.940878324	0.99997212
Q6NVF0	0.941347289	0.992563131	0.977245007	1.008783645	1.051164508	0.995041615	0.991162393	0.992960394	0.940871003	1.124773045	1.073386344	0.97920858
Q6NZC7	0.97753081	1.085162486	0.948977813	0.999654009	1.036000745	0.990859431	0.988702154	1.012301742	0.95635312	1.129201831	1.012508258	0.946445415
Q9JIW9	1.102182549	1.003335384	1.01368393	1.01053648	0.98479615	1.072569978	0.996720076	0.992033369	1.002252967	0.955348259	0.913291256	0.989652545
Q9DB29	1.085617355	1.018857057	1.01228228	1.032611248	1.012567424	1.016795639	0.999934521	0.994141906	0.995969094	0.907362892	0.967758665	0.961733675
Q3UYC0	1.011765039	1.006775876	1.001445592	1.010572312	0.989981753	0.972928127	1.022992389	0.984118425	1.030373331	0.961192783	0.99116807	1.022439054
Q9EQI8	0.937711848	0.974325478	0.970579161	1.016035274	0.926121195	1.00282542	1.002586449	1.071612387	1.053173374	1.022778255	1.008177276	1.024457689
Q9DAR7	1.012281996	1.006091926	1.022901668	1.020302755	1.041778672	1.020002175	0.981943515	1.007937606	0.953203904	0.986337042	1.012473699	0.961780527
Q9DCC4	1.025656293	0.991920578	0.983379355	1.01334309	0.956642562	0.987954043	1.007318663	1.013630412	1.01233559	0.923788826	1.055285515	1.010651224
Q922B1	1.102641374	1.09215726	1.142991986	1.108947023	1.019161192	0.947915895	0.917494131	0.963508403	1.045332592	0.773944808	0.920703832	0.923931741
O09164	0.954609586	0.971642216	0.977484934	0.950840885	0.946925468	0.929657704	1.047745168	1.069639639	1.068485518	1.036477864	1.038073684	1.006211807
P31786	1.301856102	1.134881021	1.176724103	1.030274905	0.990006769	1.170104166	1.002879854	0.853034367	1.055046835	0.650014447	0.768974463	0.818620176
Q61191	1.011979332	1.007374732	1.013635949	1.047394853	1.029059118	1.011731909	0.979814051	0.982645737	1.00477324	0.912309714	0.999704974	0.996325717
Q9DB73	1.01944844	1.026755497	1.014458381	1.008928186	1.074363671	1.020688966	0.944807989	0.969876057	0.982405438	1.007452856	0.989286515	0.987586432
P48771	1.046886666	1.237023663	1.051026833	1.081669278	0.935868211	0.835165508	1.026838295	1.016280523	1.03419768	0.785028501	0.974977143	0.906589606
O08842	0.999002365	0.967533152	0.984270284	1.009531621	1.075562003	0.936968619	0.94854778	0.971305117	1.048833006	1.048584919	1.033294408	1.018408976

O70194	0.977617969	0.979009629	0.987581636	0.980503773	0.957668694	0.947969901	1.035393237	1.030210729	0.989786631	1.075235119	1.038163339	1.038058273
B1AXV0	0.964073972	0.944201319	0.928553387	0.972352452	1.003695418	1.037393404	1.017950585	1.037303017	0.987357753	1.041305917	1.052478327	1.042500504
P09055	1.032787954	0.943177961	0.992761156	1.027203261	1.000921674	0.986491668	1.003124152	1.002221277	0.989165602	1.001182925	1.02130246	1.028181911
Q9ESZ8	0.995261906	0.975589229	0.99445978	1.047728887	0.981977834	1.054179728	1.026326279	1.019729746	1.000252512	0.970710382	0.976397968	0.972502536
Q9CX56	1.063916615	1.0336873	0.991359851	0.93454403	0.955149449	1.020125276	1.004663706	0.939820342	1.044719641	1.020972605	0.999772321	1.024316209
Q9Z127	0.987347336	0.973023731	0.985216368	0.948140406	0.95076327	0.976588801	1.030581227	1.000960189	1.037709718	1.082698367	1.032092548	1.030513953
Q8BFS6	0.963876881	0.994828193	1.024205171	1.031174067	1.09333257	0.986971397	0.985550244	1.007792637	0.935466176	1.023078722	1.040557453	0.947910426
Q9WUR2	1.06383478	0.97003977	1.015816859	1.024045794	1.023987781	1.087232234	0.991528912	0.998976834	0.934241679	1.008757398	0.964283678	0.973750071
Q921T2	1.019559415	0.995399713	1.004973739	1.013415887	1.015865794	0.994388245	0.979754698	0.992224756	0.9824914	0.9772216	1.027968373	1.015752805
Q8C129	1.061517176	1.02853527	0.977268306	0.920443655	1.031339772	0.983922318	0.980799793	0.991289546	0.983796376	1.207018266	0.993398069	0.959204275
Q6ZWY3	1.022830253	1.018264735	0.977140726	0.999163086	0.952174595	0.929230163	0.979082696	1.03856948	1.068167959	0.903126103	1.0798453	0.990206382
Q61772	0.934074904	1.25103297	0.92489526	1.043687105	0.963872633	0.961270489	0.975501005	0.989872072	0.935625959	1.004944442	1.035435959	1.015066881
Q91XU0	0.947852065	0.963957608	1.013738692	0.966878578	1.08202805	1.01323815	1.006582991	1.002194518	0.965964505	1.009931376	1.024273896	1.025417774
Q3UI43	0.970474658	0.951581183	0.991707509	0.999658511	0.952961344	0.976271715	1.062737974	1.008069642	1.0439251	1.013914304	1.003282577	1.034972846
Q80WQ2	0.863244418	0.98380596	0.89549587	1.026454646	1.001131435	0.981410946	1.049597778	1.053931705	0.990557611	1.060402212	1.0423751	1.080134376
Q70FJ1	1.034228914	0.993157154	1.016808083	1.037764366	0.977537217	0.991751417	0.980005326	0.976068297	1.002584577	1.024847173	1.02050461	0.982561759
Q8K2T1	1.180106528	1.140484903	1.108716437	1.178303577	1.210869572	1.13430072	0.78211695	0.884300592	0.865367659	0.805249122	0.89210308	0.875493083
A2A8L5	0.948446351	1.096006933	0.954394721	0.956893503	1.033850457	0.913235116	0.994665066	1.030957501	0.998624833	1.113180072	1.052570217	0.958613549
P26049	0.914539821	0.928234222	1.01534073	1.031374062	0.951963979	0.958975052	1.063518174	1.054604215	0.981280587	1.07087627	1.025915299	1.030459195
Q6PGL7	0.968867676	1.033139164	1.025715248	0.997305369	1.040232108	0.99383901	1.033218401	1.02201978	0.920913092	1.103437133	1.00304239	0.92421727
Q9Z0E6	1.171638482	1.197224425	1.052938403	0.865152113	0.925268769	0.854907672	1.182201821	0.902848402	1.029733861	0.90756308	1.010617202	0.863988761
Q5NCE8	1.141184096	1.108851837	1.037997952	0.990142079	0.905403441	0.973837896	1.013302696	1.042845058	0.938969763	0.958618258	0.969934168	0.944246171

Q9CQE8	1.008376904	1.153608225	1.125475164	1.1190277	1.178749102	1.077789467	0.888147225	0.928855894	0.840829606	0.813364183	0.943293206	0.933566871
Q4VC33	0.97828214	0.987871403	0.951193719	1.025226059	0.974957636	0.99868299	0.95926245	0.970152908	0.959289589	1.097966067	1.07244574	1.093446066
Q9D967	1.018788649	0.958790223	1.047583482	1.008265922	1.056056793	1.066996469	1.022291204	1.008632291	1.018423597	0.987516819	0.905262758	0.93299105
P47746	0.954487803	1.01408048	1.011977559	1.081696645	1.019142679	0.981955639	0.981054893	1.005896283	0.997894266	0.925671456	1.01027105	1.010155858
P50136	1.030047035	0.967320016	1.003633831	0.963928656	0.989090751	0.995373029	1.026168204	1.058154572	1.011790089	1.026786857	0.989571281	0.963922269
Q9CYR6	0.992288535	1.37732704	0.95508608	1.007833017	0.947070291	0.826915691	0.942476495	0.978939307	1.050505195	0.693262313	1.067589344	1.048667869
Q9Z1D1	0.986179094	1.032012349	1.037303233	1.035729407	0.995250299	0.964561957	0.960367049	1.005743501	0.956746812	0.973865116	1.043082587	1.02265421
Q62165	1.019775365	1.005679537	0.970545209	1.009369879	1.040902756	0.948421219	0.952704362	1.036375866	0.979325989	0.932877791	1.066381641	1.034337681
Q3TUH1	1.005835228	0.943097094	0.995865816	1.082094146	1.146196752	1.008244335	0.955794055	1.032387738	0.952710725	0.945956324	1.002033418	0.953374454
Q9CQV6	1.038981589	0.976957888	1.034008517	0.958369487	0.928048416	0.957346436	1.054386562	0.996084124	1.006106329	1.121420786	0.978597182	1.01539736
Q8R3Q6	1.004948113	1.055741998	1.021632684	1.057393467	0.987419193	0.985601767	0.984656913	1.001862509	0.987198663	0.875593649	1.017174099	0.996982308
Q99P88	1.013046068	0.982314479	0.98966857	0.977253052	0.998119001	1.018803754	1.025436918	0.956336967	1.00278794	0.948594663	1.052780379	1.029884289
Q8JZL3	0.998510532	1.005860753	1.017385795	1.006962995	1.01406438	1.008456407	1.074226686	0.980533133	0.985553864	0.960276555	0.986492237	0.966551502
Q8BK30	1.098635925	1.057998252	1.064776189	1.098551948	1.023734003	1.033415095	1.017248994	0.948667611	1.01514167	0.875499974	0.832761541	0.954703178
Q14CH7	0.993607142	1.015742055	0.98568992	0.968313441	0.943217992	1.043236524	1.02965774	1.02825976	1.010282033	1.016664417	0.981308624	1.008055876
Q61831	0.913917913	0.888907192	0.942128492	0.999610491	1.018331946	1.045015862	1.012655027	0.893960047	1.01145807	1.041935794	1.113602769	1.143067147
Q99KC8	1.112729023	0.982387473	1.023865371	1.009971658	1.083325099	1.064732424	0.991764463	0.961750663	0.960517227	1.0023029	0.941191685	0.927715583
Q8BFP9	0.929470046	0.922303877	0.969239864	0.93675998	1.148815638	0.991314196	1.087574211	1.039730171	0.986877311	1.032191711	1.015244867	0.957197994
Q3U7R1	1.130282823	1.046676954	0.983220231	0.943072909	1.054390495	1.008526536	0.980655384	0.987402038	0.99055815	1.008832556	0.981502178	0.934860464
Q8BLV3	1.021770278	0.9573554	1.034116363	0.958897896	0.982548804	0.992777226	0.965303842	0.998265536	1.011837798	1.049724769	1.052232036	1.006533854
Q9CQH7	0.924983752	0.908730548	0.962202686	0.976544746	0.962087697	1.002322771	1.036815536	0.999388652	1.063337784	1.086782736	1.00372991	1.111037223
P63213	0.958885573	1.318078596	1.096420641	1.06896919	0.786870286	0.817789631	0.859249163	1.09304194	0.95539285	0.814760819	1.167423835	0.973471629

Q80U87	0.95707032	1.003469467	1.024885639	1.128337109	1.193265852	1.221551943	0.930873272	0.932678949	0.900612308	0.929275589	0.942610133	0.883639392
P51125	1.156397295	1.078759823	1.0444147	1.047750983	0.991954102	1.015154575	0.948525524	1.003857217	1.015047053	1.001133066	0.879921172	0.884094363
O35887	1.019545709	1.020623283	1.017235446	0.964634638	1.054454985	1.03990816	0.996160629	0.986576653	0.961903019	1.111182028	0.990721217	0.9147628
Q9WU01	0.978096113	1.029440579	1.119873865	0.98607664	1.017801404	0.928411838	0.982008775	1.076297213	0.944211671	0.995311809	0.980846096	0.976080798
Q8K274	1.074763874	1.066827274	1.0435627	1.032330996	1.00711029	0.990905328	0.966986425	1.019661408	1.033035308	0.797735208	0.954656413	0.967019045
Q9ET77	0.901871831	0.905254345	0.971104522	1.023984219	0.966325955	0.99261325	1.026429172	1.022815227	1.051796349	1.006693899	1.02976361	1.1022928
Q3V038	0.951002272	1.056183556	1.023239266	1.015361845	0.954812479	0.9704831	1.019210058	1.003145611	0.976894014	0.903439461	1.016727581	1.085870981
Q9Z2A0	0.957351736	0.968537285	0.970688717	0.983679648	1.095113558	1.065374852	0.98030993	0.997638259	0.936552036	1.14099305	1.005756866	0.992267955
Q9D0L4	0.980070851	0.969255633	0.969478895	1.081058368	0.936815979	1.013907332	0.954388189	0.960247972	1.110383971	0.803003941	1.039127529	1.116578461
Q8R1I1	1.02563702	1.09814265	1.069452181	1.029390728	0.968915009	1.159082119	0.968213711	1.017208813	1.007596013	0.725021481	0.915149527	0.946146694
Q8BYA0	1.028657813	1.014938445	0.966892687	0.973635394	1.046456177	0.994655681	1.004042382	0.97533167	0.998318425	1.074119755	0.992843491	0.994011476
Q8BZJ7	1.020699953	1.119207482	1.152575477	1.042642012	0.919519316	0.817208246	0.89245482	1.085414728	1.155253191	0.711530898	1.011965255	0.938726331
Q5DU31	0.940722248	1.072392171	0.963224715	0.973980189	1.011636011	0.919823412	1.036233219	1.006992089	1.059503976	0.914959863	1.060813259	0.996119121
Q8BK67	0.947994049	0.9896359	1.004746413	1.023109402	0.972554415	1.019827162	1.024544404	1.005135108	0.981286381	1.003812411	1.020401452	1.023273311
P28659	0.958236147	0.959361125	0.919837371	0.967824999	1.016890266	1.061582762	1.04435989	1.007139607	1.014568079	1.080416563	1.018238403	0.998210303
Q80ZW2	1.033472533	1.006996072	0.983206761	1.015560015	1.031277472	1.039452037	1.001545509	0.981118152	0.949359288	0.997255857	1.003142952	0.998650919
Q9DCP2	0.98167936	0.957188115	0.969376387	0.993528811	1.061741083	1.032979806	0.995258035	0.981872762	0.973501127	1.077863903	1.031133489	1.003070016
Q9CPS6	1.000536127	0.997439751	0.990737724	1.043748389	1.014300682	1.05664742	0.980912859	0.968224211	0.983155146	0.995096648	0.97650015	1.034216847
Q8BTV2	0.977609982	0.996335598	0.99442651	1.004828083	0.94907087	0.993724256	0.987566291	1.004893799	1.009157349	1.042295029	1.02511184	1.048038255
Q8JZV7	1.029173616	0.995439448	1.005863759	1.040012055	1.040983545	0.950913422	0.984430028	1.015137113	1.052590734	0.948185395	0.974993013	0.967938452
P53657	1.180037412	1.19187314	0.987674451	0.917679742	0.969810991	0.894489276	0.978385243	1.106541151	1.055506461	1.055817767	0.909198993	0.810699415
Q61327	0.923823105	0.996098397	0.971781586	1.261007259	0.991958378	1.008355684	0.870002806	0.911699396	0.993182387	0.800885473	0.998650154	1.260527192

Q9R226	0.955744676	1.00586504	0.906013409	0.902982387	0.97706065	1.028111222	1.167742462	0.972588872	0.97334845	1.22109202	0.996225045	0.991025845
Q3TMH2	1.007484235	1.009540972	1.022633363	1.017148571	0.931083279	0.994899663	1.04809685	1.071289011	1.060013314	0.88829699	0.945945755	0.967780623
Q61074	0.995830171	1.016315655	0.979730371	1.011075845	1.087176148	1.008756934	0.954862354	0.959056534	1.014149863	0.932871426	1.024396082	1.02088746
G3X9K3	0.96277291	0.982311293	0.98405213	1.024527255	1.001269699	1.030634709	1.0517536	1.009326778	0.999458702	0.969158427	0.98805863	1.003569253
Q9R1V4	1.074451337	1.056882019	1.07624263	1.094610558	1.066901745	1.049573156	0.917748432	0.96107267	1.011134399	0.755730414	0.963362522	0.926476816
Q9QZB9	0.999792262	0.998791814	0.961882138	1.019426572	0.973024363	0.991407023	0.982455577	0.941364403	1.192274142	1.16528968	0.90405228	0.958731129
Q9D8S3	0.997295669	0.957875079	0.993870773	0.964433392	1.028331388	0.986601072	1.009361112	0.974076288	1.007053231	1.027864417	1.061398657	1.01362899
Q80YR5	1.047058942	1.005367223	0.989027808	1.037371233	0.988868974	1.031916101	1.001621903	0.978846392	0.999131302	0.939506733	1.001640136	0.990122086
Q8BUR4	1.069606439	0.982577029	0.987184746	1.03322926	0.89200013	1.019081282	1.056801648	1.01359181	1.089451042	0.885749415	0.916071854	1.035782873
Q62383	1.005727151	0.986856286	1.020267519	0.97976962	0.979042082	0.9448793	1.041508045	1.033177664	1.023305709	0.999733124	0.997733444	0.996102654
Q3TDQ1	0.951177497	1.01178301	0.980080743	1.034969016	1.087664766	0.991217947	0.960109383	0.997347686	0.912883058	1.030303402	1.070411747	1.016092994
Q7TT45	1.107840595	1.021040045	1.059875494	0.884699786	0.997004099	1.125991815	1.024804512	1.011356538	1.044515501	0.945370221	0.920242462	0.861047544
Q8BH79	0.97900027	0.988558972	0.985896338	0.975169807	0.976419914	1.009993687	1.016712099	0.984793912	1.026543528	1.076754025	1.006979124	1.018721474
Q9EQG9	1.037606356	1.049329413	0.990231622	0.943122849	0.987954324	1.021119814	1.018001982	0.979044706	0.976246909	1.091408888	0.980551334	0.995382476
Q61334	1.027868343	0.997601294	1.045493339	1.024650439	0.978890603	1.024584879	1.034624142	0.931763858	0.98401114	0.911158048	1.009710832	1.021649355
Q60790	1.073408792	1.030147321	0.98585947	0.942443728	0.970583977	0.986493563	0.979466433	0.93506755	1.025346172	1.035026351	1.044138976	1.030755345
Q7M729	1.08926338	1.044365169	0.98927514	1.102855442	1.08305445	1.056948578	0.957069211	0.934048909	1.004654122	0.884900496	0.915108112	0.966184945
Q8K4Q0	1.035300165	0.925205908	0.951207883	0.991852456	1.049209971	1.067425728	1.066106421	1.015510582	0.967496319	1.196703637	0.898594475	0.966295643
A6H611	1.021986035	1.133460129	1.10699159	0.929259712	0.991962283	0.96491808	0.988727045	1.012281021	0.985102674	0.920712081	0.97451966	0.955463783
Q9CZX0	1.056305731	1.078488073	1.052448194	1.075843926	1.044043263	1.018208598	0.923540024	1.012039436	0.930194096	0.967291874	0.980936125	0.901825051
Q8BU14	1.070548562	1.031461895	0.98918012	1.026699069	1.006294522	1.029541034	0.983316153	1.005757515	0.976283369	0.993277999	0.96848868	0.963627893
P62331	0.995428919	1.004354283	1.018222378	0.97438979	0.940380001	0.960929666	1.08691485	1.064732557	1.086151491	0.940086605	0.924885255	0.980632897

P98084	0.96294435	0.988360787	0.95980795	0.898456981	0.960756194	0.976807992	1.016584	1.04391362	1.064560723	1.111617079	1.026125342	1.028119792
Q9DBC0	0.985021771	1.046043904	1.000683434	0.972804019	1.005792023	1.023035623	1.029748526	0.994172303	0.974439437	1.006836841	0.985353232	1.004371719
O55100	0.962133522	0.998192843	0.978341875	0.977248919	0.923499687	0.943906534	1.110748271	0.972650954	1.026332442	1.21204109	0.961002227	1.029725858
Q9CYG7	1.01147824	0.972102544	1.007833979	0.938390296	0.968663892	0.913050363	1.085732077	1.087206662	1.085873462	1.003045235	0.967838681	0.95037799
B2RXC1	1.080915844	1.008641951	0.969156061	1.04646833	1.000105312	0.993487716	0.993337384	0.988181152	1.011240693	0.966714269	0.96294735	1.013255364
Q8BT60	0.962259658	1.01167879	1.017660146	1.074607635	1.109390962	0.974224503	0.926391082	0.966996477	1.04532757	0.758959663	1.035396885	1.044985505
P59808	1.172660203	1.148032961	1.083290623	1.059802953	0.865709137	0.850177384	1.119320068	0.989433677	1.058160421	0.78092957	0.870078146	0.949855633
Q8VEH8	1.001638302	0.974508647	1.021758377	0.92341742	0.97492757	0.950710178	1.007912783	1.04806431	1.092399978	0.927290918	1.004634621	1.037352053
Q8K1X1	1.048938742	1.00952921	1.000878275	0.938949669	0.965449187	1.005606179	1.066782906	1.079131308	1.014725364	1.025327043	0.944754362	0.926143235
P60761	0.679640599	1.085360372	0.830889502	1.087189648	0.90094194	0.899901906	1.193779208	1.010986071	0.986078152	0.865541759	1.190261114	1.163133812
Q9D3A9	0.958310359	0.972115764	0.932194928	0.969000104	0.981127743	0.992322549	1.053285022	0.932509128	1.027706486	1.081260381	1.048104609	1.095515052
Q8BY89	1.067573322	1.075847793	1.112201922	1.183641626	1.143736356	1.046761682	0.842757204	0.918141786	0.920272894	0.738809821	0.958164593	0.974953674
Q9JIZ9	1.011532306	1.021061681	1.011874605	0.924640465	0.965348855	0.957606215	1.014725341	1.066887656	1.014043211	1.204582854	0.984461983	0.913501839
Q9CYN9	0.975256307	1.018686543	1.028788582	0.944395391	0.850359546	0.90968137	1.08757914	0.975157243	1.001758657	1.122383577	1.078460734	1.040923732
Q3TLS3	0.937188292	1.005491406	1.006279525	1.007186609	0.995550142	0.953038643	0.983633519	0.994996794	0.978189399	1.026805694	1.081582909	1.047287802
P35283	1.008104496	1.007724061	1.034671455	1.046552662	1.045304632	0.992946847	0.978264357	1.041733	0.97058488	0.957065041	0.961034521	0.976514529
P39038	0.980760786	1.14674912	1.010238157	1.038382965	1.036370626	0.992491609	1.002596869	0.887519062	0.782607836	1.245231682	1.075818285	0.955615319
O54828	0.965280673	0.988168682	0.972658935	1.103890094	1.021685725	1.015415124	0.950847035	0.983967103	0.945286586	0.936592904	1.029442697	1.105733903
Q91YL2	1.053195437	1.096459304	1.029573246	0.986844644	1.071898891	0.963920266	0.981200106	1.019448353	0.938608248	1.039768678	0.993309238	0.879843612
O08582	0.984374637	0.991726696	0.971314572	1.014384667	1.028142111	1.039527974	0.96779812	1.022520743	0.982900535	1.113442957	0.985601817	0.978973601
Q9D6K5	1.028786309	0.982582555	1.030989719	1.018338516	1.006362519	1.002352595	1.037291813	1.033233331	0.999268745	0.947215376	0.978699938	0.935055989
Q6P9Q6	0.958150446	0.988503423	0.970156302	1.008739759	1.062638885	1.049003997	0.994797685	0.972607189	1.042615879	1.069366553	1.025711554	0.899785392

Q61425	1.217421695	1.005897471	1.025204893	1.0352114	0.968725532	1.115171144	0.974040193	0.966490158	1.024798885	0.902552911	0.875007735	0.926241848
Q8VEJ9	0.999349003	0.963182688	0.992858202	0.994346569	0.988969929	1.043243744	1.00746588	0.994110767	1.027178878	0.98390464	0.97647288	1.048156582
Q8BYL4	1.045474853	1.012015142	0.981575494	0.943513595	1.009233377	0.96606078	1.048859232	1.000914126	1.032257269	1.062214768	0.990312436	0.949141376
Q80Y24	0.905427657	1.017935459	0.969835595	1.062575846	0.968292153	0.913270399	0.984180843	1.016878434	0.986561507	1.068046896	1.103851071	1.030750729
Q91Z38	1.034322652	0.969719357	0.995778925	1.003763462	0.971951227	1.04418718	0.999569289	0.968531542	1.020598968	1.047216875	0.952111225	1.05038048
Q99LD9	0.982010246	1.067263775	0.976268587	0.967039145	0.981164189	0.995954292	1.046228897	1.029635814	0.980365262	1.021716312	1.004839349	0.971047253
Q922R1	1.006114931	1.071258189	1.058195234	1.023316489	1.004993457	0.92380994	0.927746857	0.987785707	0.972909557	0.920754717	1.077947237	1.011759775
Q8K4Z3	1.032008278	1.037834843	1.064401214	1.055912491	1.026697472	1.02826463	0.936239949	0.962431522	0.964881719	0.862668954	1.008523344	1.007295057
Q9JMK2	0.970710663	0.964481639	0.989602711	0.975185147	1.013386988	1.021334285	1.010830271	0.932195718	1.056400746	1.121667185	1.008099795	0.999772057
Q8K221	0.925472258	0.982147128	1.034604648	1.005167629	1.027716829	1.013012725	1.031847259	0.999568275	0.945954322	1.016739349	1.024702554	1.014232914
Q8VCC9	1.011562897	1.017346763	1.034512245	1.010816476	0.88790573	0.907791228	1.052154358	1.042276698	1.026194872	0.930868814	1.040675624	1.004581438
Q9ES00	1.024705414	1.109362118	0.961079128	0.931363088	1.035510262	0.989858054	1.017344518	0.988638703	0.991841395	1.092338125	0.986495749	0.937601921
P51437	1.333258205	1.073705445	1.313585845	0.70370886	0.729806261	0.699684062	1.209974071	1.295488786	1.203754174	0.85950102	0.736047227	0.742142921
Q99KF1	1.002559389	1.001048053	0.951679252	0.992032826	0.975075439	1.006255679	1.040224703	0.988924002	1.023362803	1.053550429	0.994227153	1.014358151
P28063	1.114866265	1.035603278	1.064933402	0.973141331	1.038227643	1.043428833	1.028865442	0.952646504	1.007724175	0.876894775	0.922127449	0.937441129
Q91VW5	0.999026307	1.026483175	0.957671853	0.992695652	0.9729166	0.976529297	1.011068451	1.073038607	0.972439324	1.027414938	1.030531822	0.987623874
Q99J08	0.963070214	0.976826152	1.008359594	1.009088348	1.078458373	1.100379666	1.016543756	0.955651821	0.978501989	0.938388637	0.989261102	0.99261942
P01899	1.22668202	1.22748137	1.131370859	0.825503325	0.918064158	0.824740762	1.218718163	1.059018089	1.108322578	0.780032228	0.823439661	0.768396722
Q9CYZ2	1.014815433	1.044665728	1.044558541	1.02934405	1.001702665	1.064603607	0.981606116	0.972762613	0.970715868	0.837837301	0.978385137	1.03333416
P41105	1.059700018	1.026801621	0.996695792	0.974640865	0.978121172	1.005511194	1.018224741	1.009392762	0.992806405	1.00876938	0.973403291	0.991903473
Q9QZI8	0.970457325	0.99855156	0.991979114	1.030311797	1.001532125	1.011972228	0.989110172	0.989885906	0.966981984	0.979689675	1.057754241	1.02410743
Q80UK0	0.950560521	0.967624448	1.031599534	1.028070264	0.986579232	0.929690837	0.990838	1.011790422	1.010311605	0.973312333	1.048416673	1.066129086

Q9D1H7	1.011437381	1.015884185	1.041246118	1.033049622	1.067272819	0.969515929	0.923808811	0.97833287	0.981964061	0.901257115	1.034060414	1.036291063
O88602	1.013764264	0.936981858	0.917938764	0.884454019	0.885764893	1.057030825	1.13228864	1.006671546	0.995564746	1.569204545	0.910703757	0.942704688
Q91WT9	1.10173369	1.024787667	0.971927647	0.946639399	1.067357107	1.09008793	1.013869183	0.979363209	1.023891749	1.027818399	0.913352943	0.900873018
P52479	0.99990571	0.968737484	0.989932566	0.996738603	1.014097535	0.987229148	1.005732723	0.991625573	1.003157317	1.068482885	1.002391754	1.024528227
Q8CH18	1.028658994	0.97493219	0.97841963	0.992982668	1.057638226	0.971650077	0.982394336	0.98078085	0.991409812	1.131916976	1.02406007	0.968825395
P59759	0.974989247	0.967210046	0.933297516	1.017057159	0.960215921	1.091114944	1.065386176	1.000791038	1.024338018	1.022575709	0.935056316	1.048640998
Q8BX94	1.017263233	1.044364899	0.998047749	0.95464626	1.004633429	0.958343558	0.990126988	1.063097442	0.981850814	1.073033574	1.026508563	0.931509523
P59672	1.007793976	1.043218581	1.080176896	0.988263383	1.030711892	1.013727442	0.984090249	0.991328309	0.966948694	0.873030743	1.003136592	0.991615341
Q9Z0G0	1.113630553	0.974616378	0.999308811	0.915909725	0.947124349	1.290749679	1.176032015	1.029946914	0.81640813	1.246197697	0.760418388	0.917588952
Q3TLI0	0.981289645	1.047091365	0.998963321	1.020238872	1.012575269	0.986441299	0.998765691	1.012267979	0.964523257	0.997983033	1.013905233	0.990994447
Q6NVE9	0.992434789	1.089710458	0.964928569	1.002158927	1.057232494	1.024924056	0.993604284	0.965435372	0.978790042	0.988852393	0.940081959	1.048019771
Q921S7	1.061470895	0.985799115	1.03188524	0.987823888	1.05831539	1.029647215	0.983868914	0.962401623	0.936821879	1.046299821	1.013115842	0.963723972
Q8BFY9	1.11827202	1.040958308	1.03741302	1.025556559	1.005646631	0.994361118	0.950655122	0.97499803	0.965270995	1.006736704	0.963221344	0.977030264
Q99JT1	1.208219878	1.082924181	0.864556971	0.86396946	1.076817794	1.019063788	0.922280801	0.94712979	1.116295286	1.398884404	0.870002351	0.863765351
Q3UGX3	0.992065814	0.968877818	1.003751556	1.007683756	0.991880656	0.991111726	1.017353973	1.033988319	0.989284004	1.054585276	0.998042844	0.993222962
Q6PD26	0.986578272	0.952999101	1.009600625	0.971302131	0.95438475	0.945606441	1.048280332	1.013938821	1.009294922	0.969330842	1.056719339	1.068080968
P48428	0.963837897	1.06583629	1.029572454	1.090744197	0.97090418	1.05626741	0.955973842	1.018540649	0.978705838	0.893005835	0.957265457	1.015968514
P61620	1.03336414	1.014227015	1.014683106	1.003334217	1.051859515	0.986386606	0.99083484	1.043146985	0.971499214	1.041441431	0.989140486	0.907273052
Q91YP0	0.98211307	1.016239394	1.05209781	0.991384432	1.016815565	1.045163747	0.996106557	1.004950373	0.97258715	1.060262441	0.967514475	0.946804225
Q6PD19	1.097337672	1.017064353	0.989134318	0.947036886	0.97101658	0.995396393	1.031986256	1.019057719	0.985737484	1.076358302	0.960201978	0.976622119
Q8BL97	1.181483809	1.028812558	0.903676296	0.937196592	1.123649421	1.083423817	0.892484911	0.911434425	1.086907803	1.24136746	0.916948899	0.866187127
O70503	1.054163837	0.988090456	1.017441966	0.920341965	0.927229769	1.000891048	1.025640956	1.007259864	1.050018106	1.028537799	0.983458094	1.020520151

Q8BR63	0.980384529	1.022008929	1.000706645	1.038994255	1.05805734	1.033064276	0.96321552	0.968371369	0.976607346	0.973214103	1.01927597	0.989702267
B1AY13	0.960749035	0.984107834	1.024523311	0.989870751	1.006079743	0.937748174	0.952251783	0.992779162	1.008954196	1.054067725	1.040665015	1.084330059
Q8C1Y8	1.059214441	1.069920943	0.991968588	0.921028496	1.023138619	0.983394486	1.027547915	1.020752072	1.003670888	1.01970356	0.996702343	0.910450418
Q8BIG7	1.062288782	0.980088044	1.074892323	0.963759607	0.940762693	0.96964345	0.993945977	1.042798702	1.03997548	0.906084602	1.011331805	0.982547817
Q9R1P3	1.031327905	1.048759009	1.039786676	0.944697204	0.904096068	0.895285592	1.124120479	1.127988115	1.149264045	0.919369193	0.889329124	0.876066586
O54901	0.963328422	0.962531497	1.069535005	0.973279829	1.023038586	0.983166294	1.001693954	1.030427501	0.959449782	1.016748549	1.034027259	0.998070353
B1AVZ0	1.00871687	1.01806425	1.004953842	0.993135349	0.98874092	1.030781575	1.021149035	1.028821174	0.972493639	1.077871064	0.941741388	0.980237934
Q9DBR3	0.961992213	0.966315967	0.959209872	0.974364007	0.977048121	1.020765292	0.969416186	0.940716322	1.019747911	0.97374861	1.100209474	1.135800548
Q91V76	1.057944509	1.000301232	1.003366734	1.010446207	1.022235914	1.005312999	1.003344136	1.026065925	1.009427832	0.905687378	0.967965954	0.98393492
Q8C729	1.066015917	1.015456481	1.026225619	0.92125263	0.844189595	0.910740644	1.080971555	1.14362114	1.252157844	0.956771976	0.867607155	0.872396526
Q8R191	0.981410008	0.936779213	0.909954801	1.068293676	1.015813987	1.082491924	1.002337083	0.943596779	0.991934116	0.870235018	1.037941097	1.146638302
Q8C9H6	0.907866125	1.012863802	0.935889759	1.009787061	0.931024703	0.909790565	0.893464603	1.031105022	1.088800385	0.980014932	1.150310769	1.124423241
Q91Y97	2.319018691	1.424493054	0.978035139	0.95700952	0.824275735	0.748764615	0.877131948	0.819685984	0.792790078	0.859264888	0.806978823	0.804292225
P63089	0.887539831	0.972539635	1.036480093	1.093848581	0.982410898	0.865996804	0.868833174	1.072877874	1.064100206	0.796518897	1.128830118	1.134244899
Q6PDI6	0.995364823	0.956324721	0.988534785	0.980819425	1.04846736	1.028666405	1.02384228	0.952230062	0.980065081	1.189319636	0.960868167	1.010791065
Q9CRA9	0.987378466	1.021391985	1.004701059	1.049736303	0.945524571	1.012043206	0.983962167	1.000578538	1.007886674	0.989514187	1.011143817	1.004267875
O35682	0.963988421	1.023345614	1.0495952	1.121205278	1.078972151	1.023570952	0.90957344	0.944445728	0.961729838	0.84549946	1.035831067	1.022695785
Q8VBT9	1.046587986	1.01399178	0.999185441	1.024585703	0.963985319	0.990972742	0.989311022	1.006672714	1.038920389	0.885786435	0.992965703	1.02716935
Q9CY18	0.920623601	0.970435003	0.967265922	0.937686995	1.080912011	0.998418055	0.96019041	1.080042187	1.006388499	1.176090803	1.0112022	0.971608226
Q91W39	0.934137962	0.93726385	0.972438229	1.027277242	1.014714432	0.981226714	1.016997937	1.003938801	1.018439649	0.989176996	1.071188376	1.030837092
Q91VZ6	0.925136991	0.917053706	0.957670164	1.026582107	1.03874027	1.02675118	0.999787554	0.963968303	1.017936044	1.140721338	1.001803629	1.065517486
Q62523	1.020660541	0.982638323	0.942617427	0.983492628	1.033382511	1.050630994	1.002316654	0.935217702	1.017446796	1.103410612	0.973904268	1.038173724

Q9D6K8	1.077924668	0.986635016	1.067293509	1.087426441	1.084356077	1.055055411	0.924794669	0.916805457	0.9744539	0.952182131	0.948038748	0.973711764
Q8VBV7	1.016916726	0.995694389	0.978215289	1.005671254	0.97987237	1.032201345	0.972103809	0.995414747	0.990622856	1.028384732	1.006521914	1.043364779
Q9R1L5	0.919158148	0.920040456	1.008753149	1.076365095	1.008161685	0.983337291	0.94869089	0.939394072	0.979215876	1.010024418	1.140980323	1.077460721
O88746	1.024263635	1.052034525	1.048982642	1.017771269	0.990253138	0.992608776	1.01159644	0.965238341	1.06966702	0.915693435	0.953331116	0.945942724
Q99JB8	1.007620334	1.012828896	1.028176413	1.042150934	1.049072687	1.034077307	0.997765671	1.002348094	0.94586909	0.949904428	0.990819516	0.95693539
Q9QYI4	0.966520974	0.947139604	0.991225435	1.011827582	0.939277372	0.946180682	1.052763738	0.98635682	1.046701673	1.013685148	1.02778434	1.078260223
Q7TSV4	1.087090562	0.995446023	1.000741678	0.968609376	0.984106053	1.044597808	1.000464053	0.963711191	1.040466135	1.000390436	0.955874363	0.996213603
Q8K3C3	0.970633898	1.06481742	0.977081718	1.003333687	0.993929534	0.983881791	1.039594809	1.015473391	0.999454283	0.946250207	1.033703744	0.959057502
Q99K46	1.007636838	1.046502402	1.038414564	0.97232597	0.958256481	0.971663367	1.012669928	1.061099528	0.988582396	1.046350443	0.976452735	0.954617564
Q811I0	0.986742545	1.021916951	0.997777164	0.988069344	0.964747998	1.011647843	1.059792293	1.029074917	1.021764416	0.936112754	0.985416977	0.981131059
P56375	1.039475251	0.985757281	0.947320185	1.00253391	1.029131956	1.050327827	1.005956406	0.986903183	0.996227786	1.013819412	0.960908535	1.033251556
Q8K268	1.07310429	1.007827388	1.028298459	0.987856064	1.089012976	1.006627536	0.922368159	0.972735255	0.897727987	1.05402146	1.049978352	0.983128415
Q4VA53	0.971350162	1.022590528	0.986604046	0.9778903	0.98778172	1.00633935	1.044592735	1.015728487	1.050778777	1.025946917	0.969276359	0.961861786
Q68ED7	0.877969888	1.009536946	0.948310976	1.04726213	1.034188238	0.974239722	1.02052377	0.998089243	0.986446906	1.070448363	1.036133274	1.038196223
Q69ZS7	0.940236661	0.967809414	0.941143996	0.954617567	1.001995579	1.024383011	1.047539253	1.009614246	0.983514596	1.163781373	1.00583752	1.041872887
P54729	0.991324315	0.996981305	1.029893735	1.017839562	1.016207394	0.982086418	0.979461484	0.982121902	1.005798363	0.962451412	1.023559565	1.017716178
O88986	1.008804966	0.99893632	1.066919171	1.035055545	1.100651664	1.098520482	0.97287255	0.907408081	0.913151695	0.988498567	0.966029816	0.998462652
Q9QYA2	0.897911584	0.896952892	1.029890323	1.029228046	0.928910938	0.947123754	1.005371041	1.024620821	1.087070399	1.00105998	1.047590697	1.087282654
P49710	1.10055012	1.049184454	1.00353853	0.928255608	1.025695409	0.989960728	1.013983841	0.998219471	1.050365142	1.073924898	0.916987124	0.915380591
Q8K224	1.259940521	1.133035147	0.891833839	0.939841526	1.282474026	1.233914258	0.806166615	0.807570375	0.99723763	1.149383071	0.860252329	0.839778194
Q9DCN2	0.97722458	0.970807529	1.021402263	1.038432699	1.150413085	1.068872464	0.955934242	0.953402003	0.878188955	1.073527991	1.027964924	0.965947068
Q9JKD3	1.00093599	0.91977979	0.982047345	1.004735957	0.994973135	1.00182576	1.013579082	0.984515636	0.9807377	1.017979095	1.045767173	1.082279068

Q9CQY6	1.023098507	1.038023005	1.021895224	0.992966142	0.93172623	1.010070535	1.052346078	0.979634813	1.026298017	0.993855381	0.967757292	0.979089701
P70429	0.948521778	0.988997231	0.975001149	1.023774755	1.025014322	0.987583919	0.986835158	0.96947525	0.968993127	0.954037211	1.073811947	1.099484285
P62500	0.846627983	1.04350222	0.928411495	1.030738706	1.047372754	1.01807802	0.981263276	1.042519877	0.949409476	1.076537471	1.094840263	0.975981226
Q9CZM2	1.039728746	1.013918931	1.015662407	0.959599708	0.909883674	0.988124989	1.042415608	0.99510184	1.026710407	0.939725986	1.030396669	1.020441704
P53702	0.993093267	1.003299506	0.973460898	1.005985795	1.017427837	1.031935829	1.002411781	0.970022617	0.989753976	1.075637846	0.993589724	1.007216468
Q8R1F6	1.010043971	1.066325119	1.068206552	0.90001593	0.922388231	0.8896225	1.001339906	1.05873618	1.096937669	1.011847447	0.998305286	0.965431514
Q60770	1.063155803	1.021303941	1.030361	1.03069428	1.08385983	1.02063029	0.961317753	0.951505863	0.994684651	0.905873638	0.968180454	0.980804912
Q9DBC3	0.973364649	0.941026364	0.965585102	0.947549194	1.025936004	0.956997412	0.934128171	1.003970754	0.970741079	1.550888554	0.983636194	1.005243235
P62267	1.006936956	1.006899813	1.032229456	1.02334427	0.929416733	1.022523905	1.05542639	1.043307143	1.013264106	0.911662478	0.961977911	0.972932133
Q0GA42	1.127710204	0.991600812	1	0.961097596	1.036851637	1.04504331	1.017204243	0.988780227	1.009245566	1.011393202	0.937115332	0.925416776
Q61609	1.132265992	1.034203168	0.956972978	0.933273108	1.185976955	0.980428963	0.944287369	0.992622558	1.003885958	1.012338656	0.950844868	0.938848181
P97461	0.983360918	0.965874704	1.006918166	1.033494237	0.993774114	1.026741397	1.000240202	0.96663565	1.011126246	0.979413847	1.002867279	1.04716082
Q60673	0.834456066	1.077258302	1.055819832	0.889035385	1.031186522	0.945130744	0.972329307	0.949215006	1.063979651	1.065960662	1.09567095	1.016306161
Q3UHK6	0.913708718	0.942347908	0.929822952	0.921736306	0.973582508	0.904940638	0.959446283	1.178043406	0.951277783	1.128995137	1.20771712	1.007284302
Q91X78	1.107771374	1.132638713	1.17073162	0.882732602	0.883164794	0.824798889	1.103214735	1.17870121	1.173485268	0.858760002	0.815575 4	0.790607664
Q3TLH4	0.96286136	1.0014199	0.996680143	1.023827305	1.005164413	1.025762451	0.999014693	0.9834946	0.979601838	1.040525327	0.98468046	1.045222818
Q91YR1	0.988503988	1.074034798	1.120303359	0.99970839	1.055362446	0.909715931	0.933046984	1.01545153	0.979737047	0.996309594	1.03662266	0.901474183
Q9D1J3	0.954026985	0.992485743	0.997007527	0.999872896	0.989092024	1.001986884	1.014903248	1.02496718	1.021787399	1.007837015	0.98836637	1.02481465
Q8C4G9	1.068593898	0.969476109	1.006284779	0.965275105	0.935798647	0.983390947	1.053819442	1.058618019	1.07982732	0.919802455	0.951031171	0.986510955
Q9WVG6	1.030644755	1.038745628	0.979439429	0.954960985	1.002982578	0.953812051	0.98016012	0.99666406	1.002935393	1.117536955	1.026068863	0.985931381
P01631	2.781817444	2.093171278	1.704058761	0.73882772	0.630858194	0.570500052	0.660680798	0.770228658	0.664472948	0.542823025	0.508096095	0.481876703
Q8VEA4	1.038199556	1.044978422	1.069076658	1.134298322	1.085767026	0.901493938	0.936768448	0.969754036	0.921669988	0.875587523	1.028090999	0.995133605

Q3TW96	1.015366114	1.024486405	1.003658245	1.010817591	1.011073873	1.018790007	0.991971909	0.975848273	0.980299604	1.024102925	0.982777442	1.007967909
Q9QXG4	1.041080013	0.993646957	0.95775008	0.929536666	0.986597853	1.017190862	1.031068922	0.94413893	1.059283362	0.987722092	1.032273685	1.028107806
Q08093	1.142274426	1.216888584	1.128867745	0.918089788	0.934620401	0.885764251	0.966566282	1.029650066	1.002250892	0.887882889	0.955211242	0.912920427
Q8BWM0	0.958117874	0.965651783	1.005752051	0.977087353	1.088402446	1.02613	0.939611804	1.027819684	0.967328642	0.965273569	1.074286954	1.006288876
Q9QYS9	1.043040337	1.00933196	1.038050979	1.058288777	1.021335208	0.928956841	0.928664537	1.044690215	1.057879141	0.978034965	0.963679183	0.949297585
Q9CZL5	1.065482885	1.003874798	1.078642515	1.052693941	1.040158162	1.071609794	0.927797171	0.98250876	0.912586396	0.912046971	0.971250802	1.007496656
Q9WV85	1.024746062	0.962532782	1.03946658	0.971215056	1.014004118	0.976957777	0.995645896	1.024535261	0.985921068	0.995303268	1.02474587	0.999835361
Q9ER73	1.028408449	0.971994856	1.038493579	1.015949924	1.074823735	1.070090165	0.943897031	0.970388537	0.929152853	1.09091769	0.97867213	0.97785628
Q99NF2	0.936218483	1.140749493	0.873092908	0.913873838	0.992871703	0.948106019	1.043400585	0.997827914	1.032052881	1.159041357	1.04933009	0.978694568
Q924S7	1.04586677	0.965760327	0.963746129	1.007305394	1.018260356	1.09003858	0.969731698	0.946872955	1.008161965	1.079671991	0.973931891	1.01123115
Q8BH86	1.052095553	1.134766661	1.009257765	1.065685288	1.025907467	1.021564292	0.957750338	0.970523166	0.943358608	0.934072551	0.943380137	0.979105329
Q3KNM2	1.060267377	0.973022693	1.020779437	1.010646098	0.932037649	0.962552629	1.050122445	1.057803182	1.087678599	0.866008574	0.931892919	1.007995845
Q6P5E8	0.989785917	1.041870176	0.965308396	1.011617423	0.995443036	0.931467858	1.022366629	1.041730386	0.936896273	1.076804712	1.019455756	1.025145135
Q9D772	0.954427839	0.974667763	0.990935468	0.977002909	0.9264861	0.964346871	1.045581142	0.971465309	1.049587328	1.108664434	1.013950584	1.069231875
Q9D6Y9	1.033296382	1.001258527	0.954277227	0.935867671	1.069460639	1.067995798	0.970086087	0.995656977	1.024761023	1.067845949	1.016179009	0.916615304
P70408	0.924266718	1.000130909	0.971109916	0.961706749	1.01973231	0.95466993	1.017723715	0.993571051	1.032840836	1.037132652	1.061298789	1.037562058
Q8BWQ6	0.983171483	0.995792873	1.005450274	0.9810614	0.913330884	0.975230875	1.035074094	1.055300938	1.073878972	0.91627888	1.017027247	1.006188651
O55128	1.004858783	0.984994379	1.039364302	1.035646799	1.010304214	1.026423208	0.999390286	0.99293625	0.98073346	1.064431169	0.94928602	0.975692114
Q920M7	0.921584208	0.8985815	0.9766298	0.990988521	0.884342389	1.007727454	1.103488005	1.033992506	1.127740361	0.953292556	0.94983836	1.121616156
O55013	1.021551917	1.070869656	1.015013031	0.979883632	1.045061473	1.033436439	0.970107961	0.998237855	0.993411221	1.041774424	0.948193149	0.939365924
Q80TN5	1.046316355	0.998947861	0.979146115	0.98643458	0.985031688	1.013187061	1.0099697	0.989772042	0.994453869	1.048180796	0.990002672	1.011370161
O08715	0.895669483	0.948253366	0.9925276	0.997146086	1.03558639	0.926255192	1.008285601	1.038041764	0.972405246	1.045850978	1.089660042	1.064871974

Q8K2I1	1.08766071	0.9963343	0.999408584	0.969014808	1.162959208	1.06437373	1.010370481	0.92432788	0.885713667	1.140408112	0.956251528	0.931616943
Q9CQX2	0.970949977	1.015083764	0.976006257	0.967639104	1.060333403	1.04556994	0.968720027	1.011523112	0.948159338	1.039491708	1.062003464	0.972246647
O88531	1.194825974	1.136227254	1.15356555	1.073858826	1.187742798	1.071590483	0.899260252	0.882047994	0.881011036	0.862686571	0.875386356	0.836481106
P56371	1.067279869	1.030271916	0.995036108	0.968214563	1.009735539	1.044451204	1.034897368	0.963514843	1.023740826	0.998464611	0.92426746	0.981185054
P83882	1.105852447	1.119105767	1.087528821	1.119752227	1.012078448	1.031090004	0.971348199	1.048528332	0.927650197	0.737605316	0.917093776	0.87937764
Q6P9S0	0.995248319	0.983973317	0.994999818	1.059707885	0.990750984	1.046308531	0.987820375	1.020060423	0.954221225	0.925393193	1.006204888	1.041101383
Q99LG2	0.955343637	0.901047791	0.946112514	0.987429904	1.023394879	0.991650514	1.035729527	0.947311786	1.031858629	1.041257046	1.087308068	1.071288235
Q9D8B4	1.112859834	1.096638819	1.110677225	1.016261099	0.965672366	0.800375828	0.946438572	1.080126301	1.112711459	0.82995137	0.928243732	0.948185739
Q8BTZ5	0.99505155	0.997880821	1.035482472	1.033505319	0.970482727	0.984132247	0.968732921	0.993967941	1.056178117	0.812319993	1.038101891	1.047769859
Q6DIC0	1.058836873	0.981974871	0.979068139	0.929449764	0.965956219	0.950164388	1.114108261	1.035636773	1.061146733	1.097433919	0.965542237	0.903446257
Q99MR0	0.970529178	0.980618313	1.034256931	1.015781981	0.992944485	1.041590353	0.982121489	1.009134837	0.96462887	1.089326183	0.981524785	1.003630135
E9Q5C9	1.001618549	0.997964276	1.010011049	0.995437929	0.986722645	0.992113673	1.013230812	1.016328872	1.001398494	1.06885336	0.966613213	1.003591842
Q8C0L9	0.989218972	0.988675257	0.987190344	1.014854152	0.990143777	1.013760883	1.013348351	0.988692868	0.995700478	0.989579011	1.001927226	1.0488415
P11087	1.744480039	0.65573291	0.963065437	2.322901799	0.565885064	1.130200407	0.55480134	0.527284295	0.571939802	2.375785307	0.75896179	0.792774162
Q03059	0.996544203	1.001302152	0.991950169	1.086890149	0.983791772	1.027694303	0.945836124	1.002275816	0.982344027	0.890143229	0.996617558	1.093206499
Q9JJR8	1.017539095	1.043316411	1.036064273	1.009520807	0.979631889	0.980243788	0.980831755	1.022438641	0.992788094	0.887927579	1.028953433	0.993216232
P62911	1.074185229	1.043414097	1.077858849	1.07322252	0.963621796	0.954916477	0.920712749	0.99225686	1.058244206	0.73199257	1.002133766	1.02834161
P53612	1.143166664	1.089087019	1.076664307	1.07484177	1.10375383	1.162755946	0.935777556	0.935139202	0.924872413	0.847406843	0.857471577	0.885588052
Q8BSZ2	0.984524519	1.002454873	0.999675931	0.987232955	0.938207324	1.074265419	1.050435091	0.968142022	0.978724478	1.004329183	1.003579271	1.031155748
Q3UJB9	0.943484779	0.989569818	1.000653948	1.006304283	0.973388825	0.969644425	1.012273198	1.040657261	1.025889828	0.979037128	1.012271092	1.04386551
P59648	0.934882643	1.0659717	1.037138651	1.039000877	1.083407408	0.940348208	0.952187134	0.985452702	1.101842632	0.678300578	1.016806106	1.040270835
Q7TQK5	0.959941111	0.946991974	1.014879966	1.047166591	1.015390644	1.021686272	0.977496082	1.023652115	0.994680617	0.969215767	1.020610897	1.015707854

P98195	1.140661956	1.004502483	0.962217493	0.929813605	1.15775653	0.979327602	0.981580306	0.914950236	0.934957044	1.17685439	1.02779913	0.91914923
P60840	0.917772257	1.167868143	0.99313241	1.11304214	0.884961768	1.005617775	0.979396981	0.95648144	0.991538259	0.855884702	1.044029859	1.051739765
O88745	0.972285953	0.967425804	0.956569912	0.951451376	0.985717259	0.994675331	1.061056621	1.036622479	1.04672852	0.948031591	1.006981619	1.053939088
Q9WUB0	1.063793667	1.006308137	1.057884099	1.014166132	1.04430123	1.040113684	0.954149428	0.957492747	0.964985518	0.966668693	0.98828154	0.974772693
Q8CC21	1.046761582	1.019683076	0.982138148	1.028547365	0.995394087	1.038096847	0.956581101	0.954597421	0.960876439	0.922109824	1.03298585	1.075831225
Q9DCB1	1.073266383	1.043898722	1.064808126	1.065118269	0.967499095	0.92810416	1.060633107	0.937827048	1.05294929	1.008742655	0.85364171	0.995480239
P23591	1.090807041	1.101607468	1.005273584	1.04914655	0.94219631	0.987527582	0.92650463	0.942674596	0.935526552	1.018703102	1.024756389	1.039286407
Q91ZM2	0.955072641	0.974950121	1.009150576	0.985509157	1.04578733	1.001938037	0.988363398	0.973399232	0.955876335	0.990483848	1.054963798	1.081475679
Q7TNF0	1.091547604	1.015398353	1.010368834	1.026023771	1.06393639	1.121786085	0.987315872	0.92511811	0.98863985	0.908930998	0.934255594	0.952679717
Q9Z2D0	0.99208501	0.95656706	0.976124256	1.007834017	0.964799743	1.016393864	1.023538788	1.000572949	1.01487362	1.02660967	1.004267903	1.047080335
P21661	0.887522008	1.023795726	1.044356371	0.981606048	0.961299262	0.907606441	1.029818852	1.045292252	1.027230837	0.958411707	1.059716816	1.035403938
P42225	1.17703622	1.041107379	1.016609465	0.950219706	1.009449246	1.009419984	1.029219904	0.923431217	0.993062456	0.960594249	0.983096595	0.937796079
Q7TPV4	1.175034089	1.118221895	1.022773245	0.740086265	0.714418628	0.779515655	1.204505358	1.186722563	1.267204992	0.907493738	0.890282382	0.899380457
Q8R2U4	1.035934128	1.02905087	0.971762874	0.966045044	0.95611917	0.988035797	1.085065847	1.043387303	1.020951534	1.022971957	0.953853789	0.953706344
Q8VBX6	0.955794284	0.98254643	0.998430963	0.962524914	1.035212235	1.019275209	1.005109663	1.009569654	0.973420407	1.049036415	1.044120903	0.996693409
P51791	1.040158219	0.991184944	1.014189047	0.984599374	0.984726518	1.009795021	1.00805891	0.974462381	1.006616481	1.031569095	0.997387879	0.996697216
P63143	0.985026224	1.063105773	0.970641262	0.960310978	1.057491775	1.058719945	0.999968689	0.997307568	1.066694133	0.997812715	0.90745634	0.967371586
Q80WM4	1.036111562	1.008651725	1.003735316	1.00486126	1.164375609	0.982732939	0.942794573	0.959694369	0.936514902	1.13201791	1.01361733	0.918903147
Q8CGF5	1.150740883	1.035199138	0.990580469	0.974863274	0.922885399	0.921089832	1.077639459	0.928219416	1.011826912	1.158585792	0.931616765	1.007307546
Q91Z69	0.979427923	0.996912674	1.015293847	1.08297757	1.067722406	1.012679204	0.924721545	1.007541632	0.986338776	0.920170251	1.001534073	1.012009696
Q3TJZ6	1.034143358	1.0162743	0.970379021	0.910419706	0.978693815	0.984779696	1.016690556	0.994843608	1.049209271	1.119986523	0.986001766	1.003757869
Q9Z2Q5	1.003761362	1.100007354	1.109378223	0.98891554	1.033356213	1.047710511	0.954618727	1.020735272	0.931961986	0.975103248	0.916408872	0.954263427

Q50H33	0.942960477	0.969161474	1.018569096	1.016237052	1.042847025	0.953116139	1.00911244	1.027287733	0.995854542	1.06611803	1.001498052	0.998404889
Q7M6Z0	0.768624637	1.514939924	0.90216709	1.079673888	0.967598213	0.875764966	0.943313573	0.976058359	0.874548398	0.953439296	1.161670709	0.969762512
Q8BIF0	0.968129087	1.06209236	0.989137781	0.988096523	0.990443564	0.881586698	0.913722145	1.023933731	1.072601937	0.941806602	1.139115643	0.991333729
Q9D358	1.014499421	0.994790743	1.005083	1.028277031	1.035094132	1.03429285	0.955975984	0.986202774	0.933893274	1.001929129	1.01548371	1.040053782
Q9QXJ1	0.886807815	0.958430927	0.977425811	0.972069711	0.981300624	0.959349408	1.054455678	1.076197425	0.968625038	1.062037909	1.094204024	1.016794581
P55144	0.923183866	1.146888333	0.874761124	0.996638808	0.998327103	0.965571924	0.973337531	0.982846901	0.960625077	1.077317963	1.102208643	1.048005044
Q9EQC5	0.973801924	0.986833243	0.997222532	1.014190412	1.012455976	1.078102733	0.960627874	1.020228649	0.982576225	0.94071222	0.995063502	1.046278974
Q9DCF9	0.953366756	0.934682681	0.991874307	0.955527818	0.95106769	0.975642107	1.052826134	1.052005178	1.056114794	1.079711812	1.019692409	0.999044595
O08579	0.949873445	0.974301617	1.029580439	0.993562675	0.996426087	1.015047466	1.012967714	1.005642302	0.981421025	1.064938471	0.995806892	1.024250075
Q9CQV1	1.016443725	1.035098442	0.970320087	1.044440581	0.989989508	1.085939055	0.976659038	1.019841767	1.016060825	0.899312761	0.973418114	0.967951744
P46737	0.970056497	0.997922077	0.990060581	0.966445091	0.99793358	0.958574948	1.053816849	1.03409242	0.997750465	1.037462625	1.01413229	1.003452044
Q9Z2N8	0.923240677	0.965724498	0.950964739	1.026581004	1.110396874	0.955821735	0.967470814	1.012239852	0.947120296	1.081052999	1.07542027	1.039214699
P62849	1.034024496	1.010310637	1.020306386	0.906371	0.942379152	0.969470347	1.11028156	1.027271085	1.053116161	0.97540204	0.943268445	1.001802064
Q9D7N3	0.964887713	0.928764936	0.97831842	0.995461452	0.990352773	1.035355613	1.039117599	0.977261938	1.027138915	1.028343728	0.995306112	1.06869277
Q8CJG1	1.06617613	1.02911176	0.998270309	1.007674606	0.96012667	0.994078989	1.055833778	1.040814184	0.972126904	0.965566155	0.95060288	0.980841244
Q8CCJ4	0.994682313	1.006396796	1.049789933	1.014350484	0.992783287	0.996600671	0.988057732	0.988787679	1.031087314	0.918082913	1.016354398	0.98279435
P54869	1.168857468	0.973458126	0.96404111	1.001662648	0.942412663	1.228282902	0.963928189	1.022176665	1.094327613	0.92002485	0.836916223	0.918218522
P02463	0.975221283	0.921228806	0.995504859	1.086109983	1.028703625	0.986079127	0.932908273	0.93813896	0.915084194	1.16151422	1.073957709	1.094677904
P70318	0.928008635	0.936414026	0.957476331	0.993670247	1.002601496	1.029351045	1.009753722	0.970684372	1.004233538	1.129590966	1.021609875	1.087477176
Q8R050	0.934334547	0.995860246	0.99869705	1.00323852	1.088907251	0.946386	0.933674648	1.012164705	0.994155984	0.933217455	1.107818951	1.030321677
P60755	1.026971204	1.019744176	1.05672894	0.934506313	0.916972987	0.984624445	1.023670271	1.004933777	1.093488994	1.060918739	0.946573386	0.960074589
P43406	1.013437939	1.000316508	1.014951807	1.076964343	0.960570084	0.996715385	0.98638934	1.028264144	1.043650771	0.874707304	0.961394149	1.02046911

P35123	1.034740643	1.051684292	0.963515874	0.989065034	1.032693314	0.992332247	0.973722571	0.971245329	1.036295407	1.032741368	0.984393957	0.985489287
Q9JL56	0.9011548	0.919628129	0.970620531	0.990464755	1.0203677	0.88506616	0.919386854	0.959213098	1.078557833	1.021805556	1.202295598	1.115311703
O09114	1.073607662	1.017356912	1.032013973	1.004872326	0.973665932	1.017358702	1.009401988	1.01797169	1.022791746	1.02917513	0.908284686	0.945429669
Q8C031	0.860961854	0.911148899	0.963675094	0.97786118	0.982863826	1.018921531	1.0491577	1.102168171	1.014354531	1.001982102	1.061489141	1.036225688
Q922Q9	1.008395151	1.002690631	1.03110956	0.907596181	1.13326192	0.88159225	0.971527493	0.962964758	0.913183724	1.245617458	1.144910314	0.90421388
Q9D328	1.024721773	1.087222126	1.019508599	1.029505241	0.960989734	1.031502117	0.941193527	1.073892351	0.973561747	0.912230848	0.988558242	0.953938308
Q9QZ23	0.970507658	0.947434998	0.970125274	0.983219385	0.977160298	1.063378628	1.033794738	0.993100307	1.055108856	1.043503781	0.966284148	1.031490702
P63248	0.834103173	1.036021638	1.035126682	1.153748023	1.206648134	1.056083561	0.957094794	0.94656026	0.902160561	0.939886709	0.996632608	0.95911017
Q80TL7	1.023690777	1.009505037	0.962817864	0.957639488	1.048824946	1.014240301	0.993153436	0.975298006	1.037704228	1.136439666	0.954709601	0.976079133
A2AQ19	0.900665934	0.958913982	1.003030089	0.947374429	1.005874368	0.938602708	1.051601621	1.01179835	1.018529459	1.103443011	1.055493742	1.033235681
P54116	1.001662076	1.454129532	0.947954653	0.980080098	0.904536278	0.904878194	1.010454825	0.995660673	0.984818003	0.982911513	0.995349501	0.853354
O08807	1.033083708	0.954470069	0.950919337	0.966672934	0.963143104	0.995625385	1.058338101	1.000416284	1.055594933	1.011991677	0.967084399	1.068598687
Q8CES0	0.925514569	1.003392038	1.000897379	1.013315619	0.986251855	0.961102056	1.021365591	1.026264003	0.996946193	0.972448784	1.053664622	1.028232157
P03987	0.862100471	0.94835162	1.587166551	0.798840864	0.798395319	0.767010681	1.016462316	1.235752807	1.169529776	0.797020502	0.904455589	0.92299983
Q9D8N2	1.021119203	0.958074784	0.99287239	1.002860523	1.016347283	1.045543412	1.034580316	1.006409248	1.033008178	0.969110907	0.924244673	1.017948813
P57784	1.007086443	0.983494532	0.964488112	0.984614116	1.031700384	1.050324015	0.989735098	0.978228461	1.036487619	1.08726125	0.933803379	1.028719551
Q8VD63	0.978401895	0.954522373	0.988423991	0.990550848	1.01435112	0.997778123	0.958739334	1.027943802	1.012737399	0.940209731	1.066507734	1.056167844
Q8CH77	0.930203185	0.913989256	0.923782633	0.966343767	0.934298686	0.996638067	1.079571361	1.066731787	1.014275931	1.133620853	1.026357187	1.064754366
Q78JW9	0.928965801	1.025441849	0.974350795	1.006806711	0.97553769	0.985434006	0.985925808	1.084522498	0.962968868	1.002324056	1.055299712	1.020426628
Q8CAK3	0.932856945	0.950118247	1.019618021	0.965379439	1.106506098	1.001782753	0.984348873	1.004948646	0.90160382	1.131022396	1.110476722	0.954302363
Q9QZH6	1.097357294	1.04136781	1.001739203	0.927698447	1.008769123	1.011211775	0.993553373	1.014247252	0.982560522	1.101605016	0.947243279	0.95654119
E9QAT4	1.054404141	0.921741199	0.968441308	1.039567021	1.018867893	1.010656494	0.968106561	0.90228564	1.006695506	1.256185106	1.0124956	0.990064044

Q3V3Q7	0.949383649	0.993946679	1.026026746	1.010710731	1.092934915	0.969175519	0.915764903	0.974039563	0.989212514	1.086646498	1.032729394	1.02194478
P04186	1.21947101	1.263140417	1.107077122	0.773776598	0.776665326	0.784307782	1.217902856	1.158953731	1.212523034	0.978313292	0.760776503	0.715520724
P61759	0.991283588	1.070114597	1.013799791	1.051268168	0.990098791	1.04515964	0.979007221	1.022139542	0.948558583	0.871230222	0.989101629	1.013986723
Q9CXI5	0.963702788	1.035141527	1.027514146	1.045650002	0.953596747	0.889673403	0.960661234	1.03189492	1.066195298	0.81360448	1.061335969	1.072713981
Q9JIG7	1.014641173	0.9891707	1.003405488	0.968257457	0.99244608	0.964224598	1.052372276	0.998842614	1.013711376	1.049841353	0.981655507	1.00958787
Q6PNC0	0.99783767	0.945891824	0.9543686	0.948339451	0.922805852	0.969519058	1.081307571	0.992442189	1.090830892	0.998513597	1.018967013	1.073481238
Q8K245	0.997800271	0.955394458	0.983449353	0.955734732	0.997624639	0.988059259	1.061577162	1.028152729	1.009463634	1.009622011	1.031347948	0.98949019
Q6ZWY8	0.770169212	1.036771912	1.138291525	1.226862469	1.024333105	0.834297473	0.955873977	1.124069871	1.039559502	0.985995452	0.75550354	1.142095773
P18826	0.972322809	1.105681686	0.96549112	0.975842179	1.001580096	0.964201782	0.957682144	1.008470601	1.005012977	0.993296962	1.02341805	1.04630688
Q922U1	0.96053914	0.930851851	0.956272082	1.000878158	0.964849372	1.025015517	1.019069508	1.059195909	1.076754586	0.903486592	0.982686395	1.08944588
P35288	1.019297511	1.066870898	1.014589448	0.927863121	1.081076958	0.925128933	1.029870865	1.020664732	0.992419764	1.056502026	0.980298291	0.928105015
Q9CQZ6	1.083078908	1.063393488	1.022467785	1.037940275	1.077269266	1.112531736	0.94889174	0.962461345	0.987348352	0.978011495	0.888495973	0.90049089
Q80U56	0.909684083	0.890882968	1.014823455	1.015862492	0.934234383	0.984826149	1.004113162	0.972748594	1.087257558	0.994547738	1.076495332	1.096791741
P56382	1.014862407	0.960480727	0.97658096	1.029139893	0.999276059	1.10708896	0.993701396	0.886067032	1.013140015	0.839789646	1.039507359	1.111401312
P22005	0.962672637	0.965634332	1.009069282	1.00621615	1.020453099	1.051262374	0.956896664	1.079602044	1.006118206	1.008779623	0.949361583	1.015889486
Q8BM13	0.913899481	0.915196782	0.976703714	0.916532413	0.919733094	0.971929387	1.097720807	0.985799572	1.057140287	1.058654714	1.096482264	1.083057193
Q921C1	1.014795064	0.987200509	0.937005847	1.032587656	1.257142978	1.020944358	0.901502478	0.959046392	0.863056466	1.123896155	1.086963437	0.929257721
Q9WTK7	0.997865137	1.010692809	0.983424373	0.96810261	1.034591087	1.047463188	1.004840177	0.953400007	0.972545534	1.047085841	1.007426744	1.024377367
Q8BZA9	0.950274986	0.969241322	1.024928893	0.988910293	1.022282935	0.961368072	0.998804653	1.004516133	0.977841798	1.133629741	1.023829289	1.012540623
Q9QYE9	1.095441452	1.055120721	1.073471432	1.094752003	1.127815137	1.030823232	0.916790572	1.025685594	0.89825808	0.977153341	0.909081189	0.866758685
Q99NH0	1.008532891	1.007465798	1.040495858	1.022333537	0.951807218	0.946718402	0.967483193	1.045977173	1.06978542	0.936858564	0.99739653	0.987891559
B1AR13	0.989675692	0.985804948	1.050561934	1.07667589	0.953429323	0.917846342	0.932039691	0.961758825	1.086348973	0.681385716	1.106893414	1.12778086

Q9QZ73	0.971064476	1.001291311	1.073590802	1.035493405	0.988023052	0.931591163	0.930650572	0.987949289	1.055606026	0.91343185	1.04133656	1.042191838
Q9QX60	1.125639124	1.151167867	1.054739754	0.825652461	0.788802797	0.789969581	1.159537766	1.204026495	1.307398637	0.886598134	0.822297991	0.789769677
P97927	0.897391808	0.864517488	0.972282738	0.977989343	0.988031382	0.910641074	1.073866183	1.121843873	1.178116811	1.136496616	0.987205178	0.908126833
Q01279	1.02259185	1.045441092	1.036035897	0.87941843	0.837538256	0.796382182	1.170588764	1.170936218	1.129550561	0.894623164	0.992096194	0.929599339
Q8VEL9	0.993487323	1.060775858	1.019878431	1.02932776	1.070418732	1.087426211	0.933613408	0.987751498	0.99866542	0.936181703	0.957539512	0.943398337
P97820	0.991073802	0.969324454	0.974553583	1.015616326	1.047639975	0.958876263	0.988215647	1.022850602	0.950768233	1.006179814	1.063193785	1.037676535
Q8BVG4	0.954659652	1.049950104	0.935283533	1.021122804	0.906908531	0.900473838	0.990244419	1.028347985	1.04256804	0.828969639	1.10352272	1.164970591
Q3U214	0.931781087	0.988341986	1.02512259	1.118305506	1.070711174	1.130679709	0.930730828	0.938630427	0.975805504	0.954976714	0.989543184	0.973639716
P62960	1.19295312	1.056178324	1.054032703	1.026254734	1.087315116	1.055580522	0.999026542	0.993770865	0.905393616	1.064724393	0.854469904	0.826217844
Q9JJ59	1.451101899	1.193175316	1.384669788	0.824908791	0.832415395	0.85795302	1.341114512	1.015638684	0.819266438	0.889452194	0.694355611	0.714111234
Q9Z2Z6	1.014859063	0.955264919	1.008431925	1.022867264	0.920569572	0.97075209	1.010363826	1.020807909	1.082730564	0.902838178	1.019812848	1.03439304
Q8K070	1.128093214	1.066993855	1.070806893	1.06432661	0.980252244	1.108645323	0.968044852	0.969351109	0.943106337	0.946859578	0.871956499	0.939061838
Q9CXW2	1.001685686	1.007051866	0.947875633	0.973381905	1.063922111	1.057588851	0.974274567	0.9848609	1.030372039	1.118927366	0.957302122	0.96893101
P63030	1.010964722	0.965465591	0.962313461	0.985940549	0.928608073	0.977337141	1.065599126	0.981059594	1.058734138	0.962009302	1.065175969	1.017643774
Q62407	0.996172789	0.972863936	0.977908107	1.01141129	1.141193424	0.980928947	0.937270219	1.030783698	0.892416617	1.068961266	1.075662901	0.980724206
O55222	1.091369203	1.129839239	1.002769605	0.956675623	0.918355634	1.010089474	1.004521849	1.080077818	0.988168382	0.887100216	0.947310751	0.971079305
P48759	1.208228124	1.253527825	1.206619516	0.682214931	0.746117996	0.701987987	1.309437106	1.168659392	1.260061115	0.910569476	0.780976493	0.667441191
Q9QXY7	0.987034015	0.959295538	0.968283783	1.019858436	0.929547157	1.004290623	1.066754334	1.01035308	1.036881446	1.010227864	0.998971778	1.022909702
P61924	0.998590703	0.961817995	1.003417543	0.988544588	0.960676927	1.025339217	1.030315261	1.015070286	1.019494033	0.93286824	1.029130598	1.015505198
Q3UE37	1.002168414	0.972395219	0.989502507	1.020964444	0.975991915	1.000338536	0.996344647	0.994834183	1.026845556	0.875300936	1.011874484	1.105786539
Q62426	1.150933561	1.092936799	0.950513762	0.914865179	1.128634687	1.048178228	0.899412233	0.920524409	1.023559082	1.29766446	0.905941581	0.86438095
O88384	0.954535645	1.037973562	1.031828946	1.044543971	0.987268493	0.977310654	0.987460698	1.024172776	0.972141896	0.81471262	1.041751482	1.066829951

Q3TZZ7	1.124211484	1.021585884	1.113161877	0.896685492	0.875226655	0.856506872	1.038897426	1.131753901	1.246586434	0.840988149	0.869931309	0.899217753
P49586	0.983130257	0.98189385	0.993192138	0.975796756	1.006397225	0.984483841	1.04373717	1.047635431	1.022687475	1.001199212	0.986289314	0.984161819
Q3U7U3	1.191942725	0.97021823	0.962233681	0.995595596	0.746570624	1.125578466	1.120449216	0.938287145	1.232826068	0.792983888	0.784040447	1.096996097
Q9CRC8	0.96905257	1.007081022	1.001836831	1.004834692	1.024985866	0.977813928	1.001415179	1.030654362	1.00035582	0.982321039	0.99813875	1.013401384
Q9JHL1	1.061870799	1.007429372	0.999070512	1.047485826	0.970598428	1.016619887	0.997108915	1.024724946	0.987652196	0.933144751	0.967775648	1.000434388
O08967	0.923280348	0.940329141	0.98374452	0.961344223	0.989027218	0.988783413	1.073057155	1.023883258	1.044818858	1.123797831	0.997833637	0.995310598
Q8BTG3	1.013599885	1.007080335	0.952835779	1.075456659	1.018140217	0.986630371	0.922870698	0.992742903	1.042375414	0.823872113	1.082146116	1.034131222
Q9JKF6	0.937276851	0.938928568	0.964208342	0.94456447	0.993366839	1.006492739	1.029461198	1.020196187	1.024185309	0.987286041	1.060051085	1.085363704
Q9JIK5	1.05906084	1.046271546	1.058604181	0.927688812	0.964443225	0.894900139	1.044245488	1.033426985	1.021524355	1.099981074	0.965406627	0.935551827
Q8BPB0	1.001690531	0.991424389	0.999964609	0.985554589	1.026492987	1.068456433	1.019397692	1.00423827	0.967722412	1.089744167	0.962198344	0.954122463
Q9DB70	0.919443603	0.943090867	0.926604567	1.041191957	1.031071288	1.059603232	1.00658017	0.972884002	0.98500226	1.1512225	0.994683538	1.063581846
O35326	1.048034573	1.020827555	1.005581992	1.004631465	1.095457017	1.015188507	0.975847776	1.007890975	0.945697177	0.987718274	1.016043113	0.911370264
Q99KR7	1.050993789	0.950581807	0.986135236	0.996716853	1.072198749	1.105833116	0.979363432	0.946593506	0.96510996	1.015207685	0.973342894	1.01887159
Q9DCZ1	0.988848532	1.025521114	1.024856479	1.108161448	1.0312794	0.926735026	0.971820946	0.994632958	1.048539059	0.834885246	1.003164509	0.998208007
Q64455	0.922315179	0.931077251	0.90309424	0.953013534	0.909711625	0.921003088	1.069531085	1.092274249	1.069802878	1.049535994	1.087687347	1.082942528
O55201	0.983757467	0.97373821	0.97611675	1.024205282	1.001396466	1.014036662	1.016177759	0.971808491	0.977406783	1.037196741	1.02512576	1.041070388
Q8BGB7	0.95624287	0.962886273	1.000604565	0.994500542	1.084334651	1.037450683	0.960424074	0.999497222	0.923926107	1.121764204	1.035063302	1.005310781
P59108	1.046377377	1.066266722	1.039709139	1.009909328	1.042911477	0.982123718	0.943443455	1.016351332	1.001029406	0.981196422	0.944658004	0.961731811
Q9WTP6	1.204537903	1.16923533	1.109182127	0.832510449	0.839933123	0.864835778	1.162289939	1.12089816	1.150096312	0.801846218	0.831536225	0.824989579
Q80V42	1.057395199	1.060657213	1.033375745	0.991803741	1.12687052	1.072892657	0.934056211	1.000220871	1.095266251	0.824629715	0.909857747	0.861930096
Q52KR3	1.002562277	1.00307088	1.058663892	0.981487333	1.047681961	1.015670572	0.990159708	1.056084508	0.932918641	1.05637357	0.980887121	0.926035073
P53395	1.074557514	0.970385638	1.027418494	1.012001681	0.986576241	1.052770011	1.006565642	0.956454079	1.026799484	0.930818285	0.959857466	1.006372679

Q8C547	1.01469023	1.007741974	0.983987355	1.00605494	1.001152323	1.011979181	0.974377276	0.960455532	0.970285148	0.968929398	1.058630265	1.058506337
Q99JT2	1.078635811	1.048212225	1.137904977	0.901170631	0.921581615	0.932544787	1.119731866	1.047508742	0.993872719	0.985733106	0.894889392	0.943949449
Q9R0H0	0.962485186	1.43846581	0.92843013	1.043461408	1.039258623	0.990448957	0.904424123	0.885751277	0.906186145	0.948856014	1.046026708	0.944457065
P52623	0.997910695	0.978297813	1.000008954	0.966854232	0.957757225	1.012783716	1.032262564	1.012765607	1.033440736	0.98510287	0.988879838	1.040725825
Q9WTU6	0.896868827	0.953002975	0.988725333	0.99188634	1.111441956	0.989652691	0.99938555	1.023068461	0.892019323	1.12109508	1.088098875	1.008590858
Q9CXW4	1.058198038	1.005252795	0.994069399	0.992805097	1.008085337	0.972910261	0.986576662	0.991111747	1.055787625	0.932176695	1.005422332	0.992867783
Q61703	1.393274491	1.262872005	0.96616299	0.762266999	1.058489022	1.037397756	0.995814016	0.926659142	1.225818449	1.103868976	0.701532957	0.689774936
Q8R1S0	0.980082966	0.960045747	1.017872455	1.065032439	1.080506567	1.041867102	0.947288327	0.961090248	1.009309887	0.93767082	0.978676041	1.03743772
P01644	1.356301459	1.402569534	1.078951231	0.979673242	0.892210589	0.943515639	0.904643226	0.919992992	0.914057463	0.916976876	0.865458999	0.902901461
Q9Z0Y1	1.095593202	1.057304848	0.964278757	0.999287545	0.974222507	1.0408634	0.989269396	0.964302626	1.104613462	1.009883269	0.928664594	0.913612534
Q8C6B2	1.067626896	1.093125118	0.897802925	0.922588664	1.046122299	1.093364165	0.975980123	0.93019066	1.043777284	1.247058353	0.927394008	0.909026156
Q3TC93	0.973661742	1.125883863	0.98167266	0.97567146	0.972599667	0.979306706	1.073621946	1.004056666	0.978423608	1.139272274	0.925765417	0.954964894
Q8BH61	1.084094032	1.063414757	1.068182159	0.88802084	0.930951747	0.91954535	1.094193833	1.060344507	1.084481137	1.00743325	0.919942797	0.880309714
Q3TL44	1.079206227	1.002427299	0.972577141	0.924517393	1.006364544	1.014843164	1.01296598	0.986186094	1.045320307	1.173002989	0.941551138	0.944701503
Q9CQ48	0.935023467	0.948636174	0.980068861	1.040519037	0.991605439	1.008529405	1.011576108	1.026907709	1.025241012	1.053813761	0.983695639	1.033391376
Q8BFZ2	0.954856531	1.056462864	1.013505633	1.033015054	1.04063971	1.012650214	0.925823458	1.067417634	1.004232177	0.979104336	0.956899112	0.978902373
A8Y5H7	1.146713822	1.13048597	0.901114	0.850020451	1.223150454	1.068832809	0.873027068	0.804139805	1.093479024	1.507113164	0.894680517	0.795535086
P01898	0.970246097	1.163885037	1.124658214	0.842324753	1.042807168	0.787603357	1.122011557	1.075936095	1.025173184	1.081639808	0.963661329	0.805537452
Q0VGB7	0.842779521	0.93473022	0.957247177	0.93016963	1.052975401	0.934550664	1.051197785	0.983616139	0.95618552	1.231371335	1.130331999	1.071998304
Q9JIA7	0.990444426	0.988775935	1.018175031	0.988283733	1.072816889	1.052837345	0.952463707	0.9705925	0.992719489	1.107208922	1.002984238	0.937341905
E9Q4N7	1.020579669	0.963870346	0.997781332	0.938883608	0.946425473	1.007055496	1.078158755	1.029995358	1.013598117	1.115749181	0.962375743	0.985735875
Q505D7	0.971872752	0.972224957	0.974216555	1.01754746	0.94642287	1.048004883	1.022091262	0.989878448	0.989096	0.942025386	1.055499068	1.061550496

Q99PU8	1.019833069	1.071406692	1.069571078	0.962009846	0.916570462	0.880569616	1.031009255	1.095460226	1.096705783	0.85822626	0.961599177	0.970176851
Q6P069	1.041958524	0.927336851	0.973735322	1.026073795	1.021366457	1.077558727	1.028072088	0.964528781	1.0249586	1.000867828	0.95863875	0.994817812
O88597	1.058879093	0.993164327	0.955018193	0.981300484	1.029029333	0.96931811	1.04583988	1.009279529	0.959984828	1.035682982	1.010112083	0.999955659
Q3UEZ8	0.945134108	0.903960983	0.965474782	1.160693689	1.05263848	1.102606218	0.940247154	0.987436646	0.964492354	0.893400261	1.00068189	1.092871431
Q9CPQ1	0.839393303	1.068405136	1.008654508	1.101567779	0.905242269	1.095317663	0.978348895	1.009404003	1.039241824	0.701123054	1.068226109	1.05688399
Q8VDN4	1.032646781	1.011093932	0.971634629	1.012753008	1.055157436	0.928909951	1.046973049	1.013911189	1.018328616	1.008332136	0.995840098	0.929623011
Q8CDA1	0.974537102	1.015071225	0.962612892	0.976698442	1.046090708	1.091194645	0.973046483	0.979879463	0.993418152	0.929974047	1.019475325	1.039282707
O55057	0.971226844	1.047654833	0.98616125	0.99254261	1.003263865	0.989646965	0.997965517	1.021222519	1.012720684	1.013843847	0.974147424	1.018800521
Q8BUY5	0.997590795	0.975932957	0.951697169	0.910894791	1.002417291	0.947681658	1.067374197	1.007570649	1.012735696	1.070371612	1.072648425	1.007384002
Q60823	0.950126503	0.935471939	0.945510366	1.011703857	1.122056261	0.985200297	1.007103964	1.072669235	0.974013613	1.020019433	1.084738498	0.905310021
P35285	1.014986113	1.010998552	1.030886957	1.036158828	1.004960834	1.1195896	0.996775346	0.978379433	0.985115432	0.90394507	0.95905032	0.960303999
P58854	0.967387429	0.982341928	1.030506766	1.064520868	1.03765849	1.009737993	0.956440179	1.00370224	0.961627579	0.956829766	1.009648699	1.036021482
O55033	0.980122685	0.969731979	0.984494892	0.998296508	1.033435238	0.974802309	0.976867204	0.976403561	0.977352351	1.046161257	1.085390435	1.031682788
P26369	1.039622219	0.975882934	1.012531563	0.993856429	1.007972627	0.993089154	1.028202092	1.015926385	0.988034019	0.961809648	0.969136471	1.030169522
Q04519	1.120359012	1.04207605	1.046586013	0.992692087	1.072342347	1.065436093	0.964502883	0.927418507	0.949328235	0.987612334	0.938102804	0.956643262
Q9DAU1	0.943534507	1.014845891	0.983283114	1.009177607	1.047210133	1.094317959	1.004107359	0.949032541	1.018700733	0.971027917	0.944075673	1.045887976
Q3TCJ1	0.978355271	1.002536452	1.026715267	0.964389629	0.983972665	0.9785079	1.024012379	1.023862042	1.02907955	0.935998467	1.031742542	0.993912101
P97823	1.069268049	1.027644474	1.008653253	1.010774036	0.950995441	1.051806013	1.015743231	0.978552591	1.011638799	0.975907265	0.951472434	0.976586629
Q6QWF9	0.923318882	0.980544511	0.987926531	1.058983337	0.962233216	0.927966153	1.141221089	1.038894008	1.013597323	0.906017775	1.025859853	0.985352063
Q9QY81	0.995792921	1.019280534	0.940761372	0.961914627	0.973089806	1.005235875	1.028833017	0.985845835	1.008869301	1.060950216	1.014027749	1.050661088
P19258	0.810508859	0.810692252	0.862959425	0.895140064	0.8298155	0.880902666	0.910408086	1.055400003	1.078369687	1.069408326	1.432085569	1.302770637
Q8BGC4	0.913541966	0.920231186	0.989301463	0.999065499	1.138173071	1.076598036	0.988646477	0.952811414	0.926932937	1.002071295	1.087355438	1.028305081

Q9Z315	1.045917142	1.028601972	1.030591348	1.01179964	1.049384616	1.000248139	1.000150181	1.003896279	0.948578479	1.026040321	0.956615676	0.954099373
Q8BYH7	1.0276303	0.962422536	0.962667226	1.049044647	1.015964827	1.029574239	0.991323339	0.978436362	0.996723613	1.090737631	0.97750566	0.998377159
Q9D773	1.038356491	1.007003498	1.011093525	1.040130873	1.024458553	1.033094569	1.019056685	1.042215716	0.973828154	1.018188065	0.906664739	0.941148696
Q63850	0.992023938	1.033002196	1.013024806	0.970245241	1.040154528	0.925114801	1.02693847	1.019307466	0.936915922	1.298513925	0.976515904	0.917398827
P52189	0.821954218	0.926514218	0.952835448	1.093829759	0.979801465	1.008470103	0.991559608	1.054457362	0.942439064	1.056521492	1.050625036	1.157135851
Q64378	1.038087591	1.067650007	0.99188711	1.00549029	0.97647877	1.026263698	0.970112231	0.98621325	1.048208252	1.040919591	0.941097378	0.961353129
D3Z7H4	1.00964545	0.996540368	0.976390132	0.994608203	0.996735298	0.987318944	1.007585731	1.053130249	1.012046443	0.957689611	1.018157509	0.988123063
P41317	1.143271386	1.158482006	1.089374158	0.812295662	0.777728672	0.792594151	1.192582048	1.161607368	1.236439833	0.902508842	0.813334912	0.844401144
Q80TE0	0.963288983	1.004259964	0.981741894	0.96038586	1.045483379	0.974662952	1.065073243	1.033841972	0.919833161	1.123903161	1.039895109	0.951110216
Q8VCL2	1.163309266	1.093981089	0.913532853	0.881912351	1.086690473	1.053959	0.918520397	0.946422818	0.980863353	1.42171597	0.977990587	0.795936978
Q8R104	1.07071703	1.052035871	0.911337504	0.951430127	1.018644708	1.109669985	0.928537327	0.952550547	1.04454649	1.259626573	0.942665049	0.918140416
Q99J36	0.980447104	0.982365266	0.968034847	1.008473059	1.060908303	1.061645255	0.977406153	0.984047704	0.998515912	1.057546961	0.982220685	0.998510625
Q9EPC1	1.016886508	1.133672081	1.020613863	1.001705618	1.075765955	1.006104683	0.945093805	1.042490919	0.921151169	1.012597719	0.991169434	0.879951936
Q60960	1.002226868	0.972116921	0.999325572	0.991405623	0.981258028	0.974832907	1.002494582	0.980364693	1.082656277	1.023954358	0.966943237	1.050462972
Q9QYY0	0.981173918	0.970772368	0.940420845	0.940206776	1.093413933	0.998062496	1.015369461	1.006891425	0.91795354	1.222120371	1.020009442	1.017982111
Q9JLB2	1.014261027	0.976020767	1.008728851	1.00949151	1.057667693	1.056282067	0.983925095	0.991212773	0.973834842	0.995256886	0.976709592	0.995211756
P09813	0.956718614	1.667376497	1.157260838	0.808903294	0.690369657	0.669397	1.236104339	1.202396902	1.341367055	0.657931877	0.729797343	0.661051029
Q6PFD9	0.965023111	0.989295415	0.986111757	1.045472511	0.971626187	1.044084974	0.994734286	0.997066695	1.022930343	0.933435998	1.018089504	1.023522631
Q8BND5	1.135884471	1.178014887	1.093220256	0.835536938	0.855373819	0.886618719	1.175091828	1.130822131	1.146011054	0.875229377	0.822155788	0.801837007
Q8BK03	1.083873805	1.026738385	0.961357066	0.955894859	0.993908639	1.009599844	1.008051771	1.005551401	1.020532359	1.058074508	0.941832428	0.999586604
Q80UM7	1.007760955	0.988024693	0.990104084	0.997145615	0.997588227	1.027535608	1.011122635	1.020812002	0.99683537	1.027897013	0.979579129	0.994393414
Q8BK08	1.082893475	1.029204711	0.961575531	0.980861095	1.091870397	1.020271083	0.938719876	0.929798957	1.035899294	1.094151013	0.960564183	0.967215397

Q9DC63	1.032889612	1.08771521	0.925268036	0.94026525	0.987411868	0.993788827	0.964110051	0.993270074	1.035336318	1.113797612	0.984410158	1.020897685
Q66L44	0.966657333	0.96929949	1.050249297	0.995246173	1.029031015	1.00588966	1.044514105	1.044627448	0.999428878	0.973842331	0.975685326	0.945472941
Q9D662	1.038090414	0.980596537	1.023591901	1.021362434	0.970969697	0.930374524	0.975384901	1.018766438	1.064168118	0.845007141	1.045705276	1.030826742
Q8C0P5	1.050400533	1.026776704	0.954728312	0.97998878	0.908427109	0.910756066	1.082113354	1.01209893	1.08434299	0.951419059	1.000353664	1.022683853
O35239	0.947826914	0.979001169	1.035731571	1.038929093	1.015861646	0.910996244	0.910003818	1.043589617	0.96675375	0.979067669	1.125262891	1.042716261
Q8BHF7	0.993585476	0.992094889	0.980463685	1.023860108	0.991955785	0.982621427	0.995820886	0.95388274	0.974923712	1.048840417	1.043685285	1.066196276
O70152	0.990694999	1.111078724	1.068243383	0.782168329	0.862597488	0.827989491	1.101220278	1.189581872	1.297634182	0.936854665	0.925222014	0.804787048
Q3U186	1.015010909	1.006448115	1.039502833	0.97178577	0.908875297	0.987795446	1.038808308	1.050970847	1.092314614	0.918499343	0.95624723	0.980868262
Q9CPQ3	1.012946138	1.048782767	0.977558556	1.000484991	0.969288751	0.959127184	1.043770336	0.977867226	1.020939845	0.996515631	1.002042342	1.008640617
Q61097	1.025177182	0.978736331	1.014927119	1.015238086	1.019317158	1.030472689	0.994759634	0.993711479	0.974422093	1.042288048	0.994940002	0.966757888
Q9CQ86	1.002504712	0.986720852	1.018658891	1	1.104877825	1.009951178	1.022628275	1.034221962	0.954479567	1.029567693	0.959226801	0.924166391
Q8K4R4	1.116296246	1.014013358	0.923874647	0.97750602	0.971725466	0.978249589	1.04108817	1.02579293	1.050188572	0.98079247	0.963290798	0.983047652
Q9WTI7	1.02894758	0.998524332	0.986655712	0.986226789	1.085247064	0.992615441	0.9725201	0.968142212	0.880503638	1.111334491	1.075964788	1.000061499
Q91YJ3	1.067038972	1.002664869	1.02826958	1.021913374	1.034557909	1.071625213	0.991027642	0.999419262	0.95505164	1.022566113	0.91609887	0.957015131
Q61129	1.168272765	1.21709765	1.167334636	0.865097129	0.825156833	0.831214351	1.12337301	1.121959101	1.186059858	0.813329148	0.797704267	0.795977579
Q61249	0.900502483	1.082653572	0.982511729	1.000993536	1.005914597	1.014028627	1.021692417	1.037284211	0.954017507	0.969388508	1.068261423	0.951857498
Q60610	0.92614998	1.032811599	0.978651163	1.002119537	0.939342245	0.991762381	0.971258229	1.087426764	0.977043805	1.159138097	1.006869891	1.005011614
Q4PJX1	1.007111607	0.952366977	0.969394304	0.954472631	0.9611341	1.036979959	1.069824345	1.021080568	1.00586171	1.042517497	0.999101601	1.010340968
Q9D0A3	1.034737883	1.022057865	1.070926934	1.027198478	1.143374061	1.074373871	0.932249655	0.956178791	0.892929108	1.095468666	0.980483903	0.869306614
Q91YW3	1.012624147	0.934678419	0.986662413	0.986067504	0.962057661	1.026105321	0.990198107	1.003400088	0.993360821	0.957557088	1.062289144	1.084026065
S4R1M9	0.974739051	0.979774308	0.93848173	0.965936257	0.982869751	1.03498223	0.984431589	0.948172216	1.01697959	1.144172125	1.053704043	1.052686664
Q8VDG5	1.020587159	1.112632172	1.047371206	0.923455848	0.968384981	0.963429094	1.019861837	1.036590769	1.018044159	0.908130047	0.987243652	0.965006711

Q8K2Y9	1.315529223	1.149115057	0.969765537	0.974433964	0.891163115	0.909098354	1.01210289	0.947991603	0.965752322	0.902133483	0.984982706	1.007824542
Q6P4T0	1.007583602	0.981276205	0.945073465	0.982979613	0.998031049	0.990554797	1.043488714	1.074473862	1.041470903	0.946524429	0.999760777	0.976594119
Q8R502	0.991207664	1.025972503	0.977820269	1.026526148	1.016097477	0.99875556	1.022390378	1.016977299	0.99010427	0.982944455	1.005956934	0.961517495
Q60854	1.018243833	1.04618762	1.023019317	1.003361026	1.02360867	1.061503685	1.048530605	0.996042052	1.022562114	0.917461613	0.894985715	0.948509991
Q9WU40	0.903206408	1.023336442	1.010174853	1.068392893	1.009436095	0.939327229	0.872016758	1.039886371	0.978089423	0.842672607	1.163301643	1.086758619
Q6P8I4	0.89635569	0.940017945	0.966985806	1.07554889	0.954879777	0.993559908	1.010929269	0.946689551	0.981449675	1.199550772	1.03199297	1.104315362
Q923Z0	1.085680895	1.182183104	1.062821825	0.949231807	0.96299492	0.884934062	1.021704117	1.092354021	1.003685063	1.07335614	0.886098792	0.854032538
Q924S8	1.114588921	0.973236414	1.009457615	1.057769621	1.067548237	1.045760107	0.951886015	0.974781505	0.965582411	1.005335927	0.970076307	0.928364776
Q8K2L8	0.988944106	1.015122816	1.016257087	1.043935872	0.96638488	0.979388817	0.999589516	1.038136063	1.016262331	0.891374773	1.011773832	1.00336263
Q8R2U0	1.032402489	1.076163054	1.005593255	0.981225898	1.009920322	0.972899522	0.976334634	0.956896132	0.950287857	1.02527171	1.04603882	1.009309977
Q07813	1.032081686	1.060849698	0.998087354	0.884135712	1.01296309	0.896399172	0.987704085	1.100684366	1.003806514	1.106480574	1.035002948	0.925946055
O89114	0.990108994	1.000089188	0.908612526	0.853433157	0.977952462	1.057122712	0.993337968	0.962014991	1.078380763	1.405380298	0.944717304	1.016723096
O35474	1.211065417	1.385840728	1.196032673	0.959229415	0.970907781	0.986096067	0.974060467	0.961736813	0.992705451	0.803918725	0.773206168	0.780993972
P56376	0.99628641	0.994603182	1.001857149	0.994804262	1.004135864	1.041163175	1.075849156	0.995801504	1.053028306	0.875705939	0.956414992	0.974959409
P46467	0.980917088	1.018162453	1.027678833	1.009084353	1.066468343	1.009861392	1.01035775	1.034700644	0.938662472	0.953856937	0.992021087	0.96901601
Q9R190	1.020338075	1.015822618	0.965540846	0.997503568	1.004958776	1.002231135	1.005981617	1.040320611	1.008708738	1.072336043	0.988858676	0.931245386
Q8BGT5	1.027176296	0.952809302	0.966946043	0.965336377	1.019722237	1.057682143	1.022844429	0.996386345	1.014919025	1.110914412	0.956261322	0.987207999
O88587	0.996653786	0.932008493	0.991783546	0.970412976	1.126471678	1.076171554	1.033890534	0.98322241	0.935790522	1.071701819	0.997000708	0.949907585
Q8VEG4	0.942233006	0.984911243	0.992309124	0.914108318	0.972582573	0.946284121	1.067367061	1.021038235	1.012709594	1.16223252	1.043968861	0.994607613
Q9D8X1	1.027146764	1.008285977	1.053602363	1.037953749	1.029083607	1.005090911	0.997145074	0.950467286	0.995442329	0.988297227	0.972044522	0.967390002
Q9R1C7	0.975119303	0.947682752	0.92626542	0.936189305	0.912032861	1.013289011	1.098607768	1.053741548	1.017088093	1.043738461	1.01157125	1.083253139
Q8VHE0	1.08956019	1.069745561	1.03741692	0.889783048	0.943031425	0.865914034	1.014701476	1.135892605	1.13729021	0.983626009	0.925191256	0.895331533

Q60648	0.940798374	0.981082798	0.967465959	0.976005965	1.214038172	0.961294129	0.976542438	0.987785266	0.997737057	0.927678313	1.075056576	0.979858351
O35075	1.008159378	1.026371789	0.975690976	0.953961054	0.972532288	0.94514538	1.036063265	1.071013033	1.046488061	1.104423398	0.97693759	0.932034013
O70310	0.933441785	0.993713901	1.067845841	1.046625065	0.998841481	0.928597098	1.023633704	1.053480581	0.952442836	1.079661137	1.020412568	0.940037552
Q62083	0.86072415	0.923531483	0.978594096	1.078582736	0.911561456	1.0368102	1.03568181	1.005847626	1.002981671	0.96273638	1.044881562	1.145045611
P61255	1.049814578	0.978356327	1.022403058	0.952107829	0.997211165	1.008041823	1.060668113	1.06264601	1.020897725	0.944995137	0.957064252	0.938715742
Q924A2	0.960200641	0.944183687	0.950702646	0.997206603	1.041077916	0.957685278	1.044886427	0.954661446	0.977993007	1.21705787	1.039227465	1.022830423
Q5HZI9	1.049665196	1.012240415	0.995453786	0.981190117	1.086711115	1.042193946	0.96045193	0.98148832	0.933602093	1.008534283	0.999745665	1.002958406
Q3UFY7	1.013497111	1.00062101	1.018027608	1.004292874	1.002622519	0.954098247	1.025887178	1.026529378	1.063989996	0.963274331	0.964230284	0.96186119
E9PZ19	1.009018052	1.029011366	1.02283842	0.992211926	1.027277584	0.90687551	0.999543619	0.992749294	1.005904096	1.050045432	1.035577126	0.962707486
Q9D0W5	0.967924804	1.045243927	0.986120511	1.02834881	1.037340152	1.067951872	0.970338271	0.957436601	0.984588874	1.002921693	0.974605543	1.020549272
Q91YM4	0.99493698	1.013363401	0.99056719	1.022736277	0.960554194	0.982854287	0.989256212	1.002130751	1.00018796	0.968279259	1.024624192	1.05965493
Q91WK0	1.068827435	1.103489464	1.110764504	0.948973316	0.920137746	0.915784678	0.996281383	0.995705256	1.030252042	0.894929855	1.02248312	0.953459614
Q8VE62	1.005849081	0.965416867	0.976207144	0.935256391	0.803641576	0.989250525	1.124093404	1.006368437	1.189935847	0.837380606	0.973296955	1.103301978
Q921W0	0.956088066	0.978589694	0.983017807	0.987018583	1.074592523	1.003200074	1.021283123	0.988494541	0.980293194	1.013482688	1.02188016	1.017972004
Q9D8B3	1.090142541	1.227029309	1.092090949	1.05374079	0.810580454	1.009920563	0.970175725	1.041655977	1.033363868	0.639492028	0.923244935	0.995752628
Q99JX7	0.955576977	1	0.986764645	1.002660457	0.994483184	1.023651284	0.991379984	0.964403267	0.994149342	1.087070117	1.032017262	1.022952559
Q8BWU1	1.026912253	1.04078259	0.921853503	0.832126776	0.914186808	1.059271728	1.134453299	0.995908891	1.150013266	0.990750339	0.936017195	0.98651393
Q52KF3	1.040035687	1.105653162	0.9738929	0.823943024	0.76559922	0.773506716	1.295912123	1.067688507	1.526132197	0.814099057	0.825305334	0.820809791
Q6GQT1	1.129833431	1.248693943	1.069147716	0.942939087	0.908482442	0.83622791	1.043431643	1.064636054	1.012437281	0.934704216	0.944439805	0.856118389
O88848	1.024378803	1.058760418	0.980194177	0.979295185	0.96921145	0.980441023	1.05875379	1.012541864	1.001721367	0.91803163	1.001283503	0.999772346
Q8K1J6	0.978548529	1.038245604	0.955367184	0.957666444	0.954686901	0.990816534	1.056478942	1.073413969	1.056532325	1.03815981	0.993720182	0.919185825
Q8BXZ1	1.014145497	0.973550061	0.996321699	0.956871836	0.985039762	0.95720141	1.058687062	1.074661028	1.006001498	0.973080354	0.994520094	1.007339043

Q923G2	1.011263496	0.98186897	0.992508238	0.942463335	1.052825179	0.95349606	1.034156203	1.036797344	0.926859473	1.080511306	1.074974122	0.956537824
Q9CQX8	0.959804765	1.058041149	0.999085131	1.044289858	0.944324852	1.030201596	1.075091367	1.005564374	1.038488078	0.89398849	0.951050376	0.971992991
P58044	1.002681374	1.021201432	1.041879853	1.013134025	1.001380638	0.966188024	0.995759705	0.990873809	1.057512936	0.832782621	1.013878895	1.005066104
Q6ZWZ2	0.966175338	0.962117107	1.008963422	0.975864382	0.979893944	0.978884737	1.062481375	1.024445794	1.057761078	1.03115433	0.95933929	1.011191736
Q9D0B6	0.961195308	1.020137755	1.068669264	0.989134475	1.136895737	0.959569005	0.971108958	1.005627127	0.913135821	1.009348924	1.044450632	0.948382703
Q9WV32	1.05113271	0.957078725	1.003887735	0.993657891	0.984285308	1.007920413	1.061396422	1.040170613	0.985342629	1.00832068	0.952450395	0.987476315
P55096	1.145449696	1.043850316	0.970543166	0.999605581	0.986582731	0.997209093	0.871100709	0.971769923	0.981921489	0.97495542	1.014540537	1.096310109
P70205	1.000207212	1.025071114	1.112780802	1.05949032	0.9786534	0.939091351	0.939851719	1.054564948	1.012282728	0.855141903	0.979449179	1.005067772
O55003	1.052420192	1.014202712	0.995675931	0.999330012	1.055833351	1.011058836	1.0472484	0.977644688	0.987996388	1.077456716	0.935997176	0.919009746
Q9Z0H3	1.085329365	0.996382258	0.988154508	1.02187138	1.024921543	1.038831713	1.024081457	0.970675694	1.017301203	0.919245322	0.942442723	0.985012088
P0C0A3	1.004422208	1.057721283	1.035807077	1.057364031	0.898154683	0.914065107	0.963977189	1.066448668	1.103729695	0.847836858	1.008475538	0.979158026
Q9D6E4	0.963207634	0.940083885	1.035803713	1.051986939	0.993311384	0.990095011	0.958416325	0.996619387	0.999772394	0.940086903	1.045818895	1.078111628
P12961	0.926203946	1.054683129	1.016285426	1.002667221	0.982962427	0.942494999	0.960647097	1.019412571	1.010153549	1.057159998	1.028954661	1.03012959
Q9CX80	1.137643352	1.05614276	1.057780497	1.011809396	1.032568556	1.058039536	0.947317407	0.973523671	0.950579799	0.886795137	0.959485611	0.944288802
Q8BXT1	0.849290176	0.917357804	0.919580548	1.065158403	1.054059815	1.056158704	0.972430657	0.985568949	0.982196204	1.042042857	1.041928836	1.156097412
Q8VE22	1.010011689	1.053504538	1.028713831	0.961241283	0.956076202	0.959628087	1.018641124	1.055058243	1.042448957	0.909586711	0.990828953	0.982294952
Q3UHD2	0.968525497	0.982818744	0.993462175	1.00792854	0.974079757	0.980995834	1.03121566	0.97770607	0.996726449	1.035236411	1.024841013	1.056382246
P01670	1.475273576	1.652364316	1.108907606	0.900753713	0.817247108	0.813039042	0.914257492	1.015587755	0.977868369	0.854794338	0.757835214	0.762822584
P70698	0.990908315	1.071491659	0.943032942	0.946966574	0.96693573	1.03620582	1.029369072	0.956241126	0.954696817	1.163111604	1.009231479	1.029040784
Q8BTY2	0.890622375	1.074027602	1.003091587	0.867175221	1.21295645	0.812243139	1.00158842	1.036802534	0.934025128	0.999603132	0.957177209	1.239733648
G5E870	1.02069102	1.04672318	1.027039943	1.071802944	1.033824296	0.98820428	0.907412837	0.996522017	0.91360838	0.949442245	1.034969673	1.039391659
Q9R0I7	0.926352232	1.072784195	0.98751077	1.004197691	0.988063004	0.940306234	0.949978493	1.071454931	1.006229082	0.98901268	1.059966823	1.001540753

Q8K019	0.931458994	1.001788136	1.016699877	0.945501012	0.986401993	0.976939275	1.018140674	1.033722269	1.058778904	0.995174191	1.0239574	0.999088323
Q9DCS2	1.03895179	1.000336188	0.970836432	0.993782813	1.013485658	1.066141422	1.048712122	0.991204964	0.959524075	0.977970189	0.999700256	0.964224585
Q69Z99	1.098960932	0.89005584	0.821610767	0.632359254	0.712672502	1.465802233	1.526327032	1.048726593	0.809324242	2.14410853	0.694054435	0.660390596
Q8K382	0.988411872	0.967682305	0.978444194	1.025110906	1.00873762	1.021072676	0.991187282	0.951610275	0.972298067	1.025494764	1.032338476	1.080900225
Q8BGC0	1.012330847	0.987675201	0.976571145	0.928587357	0.987189998	1.009755432	1.025678617	0.987135378	1.047779377	1.09596109	0.98371638	1.012506735
Q64337	1.040494427	1.004068922	0.992703646	0.989555151	1.052718152	1.032382086	0.942965528	0.964052993	1.007117518	1.033287793	1.000214801	0.993932007
Q9CQI7	1.017347561	1.058723272	1.007201607	0.966285569	0.982667898	0.991094271	1.032925903	1.022020677	1.01291574	0.916535319	0.990820427	0.983527901
Q6W8Q3	0.827942971	1.006779729	1.004419992	1.14547076	1.061262596	0.997406594	0.902474893	1.088830665	0.98650773	0.919230158	0.949698478	1.112614723
Q99P31	0.883195463	1.048669878	1.023278654	1.033488674	0.982091657	0.904236183	0.969002148	1.034650566	0.935879861	0.984857603	1.125115045	1.065302578
Q8VCR7	1.060143539	1.058998935	1.041453656	1.052922883	1.106290959	1.024993751	0.94259105	0.950152697	0.903126394	0.898152798	1.013573145	0.966136422
Q5M8N4	1.05017606	0.967036284	1.063918101	1.059017501	1.026583049	1.120843261	0.978915847	0.993721615	0.983710797	0.887772908	0.906178084	0.972134806
O09126	0.92904571	0.963506022	0.997807685	1.025063852	0.99439667	0.983679796	0.980661887	1.031315338	1.011715875	0.957945267	1.042301729	1.073587179
Q8CE50	0.903181752	0.965960358	0.951047492	1.022206621	0.859624652	0.911953511	0.987830442	1.069937316	1.183911091	0.795964693	1.053223753	1.182943124
Q9D1Q4	1.111828208	1.182289283	1.24843282	0.923667072	0.806824156	0.781254827	0.93310816	1.111884299	1.244280558	0.730895318	0.90109411	0.886550638
Q8BXQ2	1.050183951	1.037235926	1.002748973	0.959778706	1.015704038	0.957266673	1.027499679	1.060044344	1.005353415	1.063110975	0.949813744	0.922236023
P70345	1.004977705	1.012491654	0.980087354	0.980626391	0.985410009	1.018653677	1.02740434	0.998397693	0.963224833	1.06746538	1.003922679	1.012302178
P63056	1.06993791	0.984680404	0.997320574	0.946335 3	0.986698898	0.988282139	1.062835175	1.013825759	1.001207788	1.040671875	0.964029942	0.986788713
P35821	1.016264971	1.03142807	1.015399086	1.008825812	0.967508628	0.9632144	1.007370604	0.992249716	1.028937944	0.917080607	1.010042761	1.025234649
Q8BGB5	0.792838359	1.145223382	0.974730014	1.056923978	1.02305691	0.943054589	1.043567883	1.10777726	0.956151386	1.060591473	0.963907489	0.962182647
Q9Z351	0.989590843	0.98719289	0.991011198	1.031687296	1.009470074	1.005043891	0.993917225	1.052631178	0.977691139	0.980903656	1.011303948	0.985353769
Q8BTF8	1.021778022	0.99773198	1.105330997	1.03125663	1.093829053	1.011009034	0.948047953	0.967639956	0.932312376	1.024292291	0.973690855	0.950014834
Q99JP0	1.039862633	1.098966523	1.10482604	0.927261119	0.942951006	0.877407327	1.039879423	1.084045609	1.087359339	0.988202078	0.906175858	0.897144735

Q3ULF4	0.982907343	0.985426435	0.972349174	1.016293522	1.053656729	0.975557285	1.004178725	0.95305403	0.937484162	1.164426285	1.053609718	0.998506073
Q8VHK1	1.06983109	1.051223927	1.063031438	1.030698948	1.103781363	1.033612762	0.945071223	0.972914057	0.939089529	0.91642302	0.902250886	1.010940402
Q9R020	0.896778032	0.790185458	1.063014519	1.04403993	1.161651702	0.978966329	1.053251276	1.07999449	1.023884258	1.332394057	0.887807951	0.837950769
Q60936	0.996071321	1.038554464	1.064962799	1.033635636	1.203900674	0.953003368	0.851922269	1.017798085	0.854753406	1.004172907	1.07211875	0.962289724
Q9Z2D1	1.009837267	0.989936132	1.012507127	1.073957612	1.023310701	0.98992415	0.892669	1.025047557	0.975149816	0.890663585	1.049887246	1.058989544
P63280	1.00785117	1.028011348	1.027062412	1.066839172	0.953412204	1.052262814	1.020559042	0.996201267	0.989117824	0.899686254	0.95037163	1.004624295
Q80Y98	0.966353008	1.050911216	1.003167078	1.005408859	0.957553479	0.957473209	1.048662417	1.084989544	1.029351345	0.93381786	0.983775102	0.9535764
Q68ED2	0.968906687	1.019986377	1.013104219	0.970372948	0.940480611	0.944557722	1.066628233	1.073291098	1.046563965	0.99363409	0.978636505	0.976603177
Q9JLT4	1.073677786	1.047049691	0.940365188	0.942038654	1.108198353	1.039662572	0.930834722	0.941433263	1.029919751	1.172992365	0.950512982	0.949784792
P70265	1.038203055	1.05187894	0.938581495	0.947414808	1.075160247	1.068316625	0.964589829	0.937322345	1.050636933	1.135911859	0.948892576	0.943274463
Q91WV0	0.943132351	0.962130086	0.972852201	0.973178882	1.013229715	1.015264755	1.025479948	1.007891581	0.98125861	1.027785886	1.047561039	1.051365919
Q61466	1.04480816	1.011206109	1.098482642	0.795156855	0.839103816	0.802877536	1.234009957	1.20516852	1.254992838	1.034804536	0.808675165	0.825735515
Q9WUN2	1.041620414	0.951537359	0.998681335	0.981547617	1.034746773	0.997317808	1.034679696	0.959222673	0.995811863	0.992835793	1.005082496	1.03326623
Q80ZS3	0.997339631	1.286956276	0.987840455	1.003546299	0.929678901	0.980663539	1.004163794	0.996907064	0.958316089	0.979116895	0.961971475	0.941094019
Q8BH64	1.106616217	0.980691758	1.039174977	0.990764695	0.991047995	0.983237834	0.98045213	1.01639333	1.003795797	0.940983504	0.990577547	0.985707725
P70349	1.026085831	0.972985385	1.054085248	1.026435013	1.022539957	1.082944179	1.005218538	0.972298918	0.922168924	0.955472922	0.991361316	0.993118938
Q812E0	0.953042437	1.015643606	0.94753528	0.989134877	0.898577039	1.042649357	1.093647084	0.973285986	1.077931456	0.986608843	0.931326515	1.100008486
Q9Z1K5	0.935569177	1.017669927	0.95606951	1.038664329	1.106871002	1.015618931	0.975154616	0.990766976	0.976115392	1.073228829	1.003598018	0.974747417
Q9R1C6	1.107623296	1.059737561	1.025802565	1.106303019	1.052970315	1.078462044	0.933220737	0.960553301	0.947603833	0.809300661	0.958586597	0.95592853
Q9CPQ8	0.948480353	1.005036897	0.98689883	0.978685508	1.059080666	1.033359672	1.025834309	1.025097821	0.980146316	0.96303893	0.99362557	1.005922823
Q9R257	1.032175352	1.012266211	1.001983696	1.0211928	1.093513996	1.127408705	0.948179415	0.924990534	0.917237137	1.107767607	0.951808767	0.976160271
Q61749	1.082693827	1.083557288	1.110736328	1.050154349	0.962602082	0.871520195	0.997875606	1.13933713	0.992850186	0.858456221	0.929672059	0.886954528

Q91V57	0.894422307	0.997401841	0.979695815	1.040281976	0.92372352	0.916000732	1.020545432	1.102502584	1.117154704	1.023304432	0.990288155	0.986578226
O35083	0.966317521	1.300176277	0.976310533	0.874477091	0.964913613	1.00780203	0.987232083	0.889452676	1.034226812	0.96240817	1.045784614	0.984685216
Q01149	1.487792665	0.782527271	0.972326016	1.730972601	0.720350485	1.051423408	0.756272766	0.739072503	0.763451189	1.707961496	0.907632826	0.902054074
P97298	0.997475716	1.010475836	1.013816318	1.003162366	0.961892359	0.961867862	1.025934876	1.04966674	1.030483099	0.941182101	0.967307933	1.028390498
Q8BGU5	0.914246655	0.938763908	0.958938038	0.986517549	0.967332101	1.01198014	1.016809461	1.011076655	1.046531686	1.066782432	1.052930469	1.050652502
P52825	1.182257721	0.994344829	0.919489725	0.863525856	0.958752347	1.181615818	1.164831941	0.949153223	0.945456046	1.245302728	0.879751947	0.875286923
O70433	0.925879352	0.93542844	0.937834404	1.030590152	0.901602728	0.969411547	0.984196471	1.027066131	1.076827316	0.845999928	1.105514936	1.186470276
Q8BQP9	0.939809716	1.015888497	0.964864871	0.983850188	1.024311482	0.959463427	1.035755494	0.978314105	1.030667557	1.068813956	0.990228072	1.051465131
Q9D273	0.969082848	1.02178738	0.970307955	1.028421489	0.967741712	1.030735656	0.970597306	1.020926266	0.976057828	1.021141366	1.01758215	1.042045173
Q80X73	0.985791569	0.949492424	0.990973982	0.995321748	1.062675557	0.981994087	1.007344974	1.070911243	0.967281425	1.084808312	1.018970069	0.936345902
Q91WK1	1.012263418	0.99537873	1.006687562	1.019213183	0.994645322	1.000830513	0.990494427	1.003339475	1.012202839	0.932838189	0.989240705	1.044015716
O70493	0.961583057	0.978442971	0.952979094	1.010201528	0.998060818	1.009847273	1.014747648	0.978446268	1.018480771	0.945595309	1.07589059	1.041457693
Q3U0B3	1.070397647	1.070752493	1.168971028	0.866395068	0.826670464	0.761833202	1.001895326	1.18243273	1.347999326	0.814612213	0.908579761	0.839472685
Q9DBS9	0.970187524	1.011883396	0.968844457	0.984946497	1.029060617	0.983698742	0.997166194	0.994735421	0.977809191	1.107293308	1.027257503	1.013277622
Q9D7V2	0.937474975	1.021397167	1.003244357	0.993494266	0.992312721	0.995163851	1.004246026	1.002830404	0.996552378	1.051592142	1.010915797	1.025605255
Q64674	0.919369439	0.9461286	0.935687343	0.969608525	1.078389259	1.01575755	1.039620007	1.027633477	0.963010775	1.103392577	1.060501625	0.990982511
Q8R3T5	1.147383711	1.060321914	1.048109316	1.057895184	1.109977452	1.12332346	0.936099134	0.93571365	0.91113605	0.971534062	0.901372457	0.878424526
Q5U430	0.919242565	0.950102222	0.959894704	1.021722005	1.011998061	1.023345894	1.000031746	0.979852886	0.994288516	0.995343011	1.056103342	1.101060912
Q9EQN3	0.986475907	1.016781452	0.980728073	1.001148995	0.962676475	1.051434342	1.01249291	1.002778633	1.06287795	0.994624221	0.974631482	0.967197911
Q8C4X2	1.013830007	1.033231211	0.988664822	0.942695692	0.987777015	0.949845609	1.01774803	1.001517698	0.986056059	1.145758787	1.005163745	1.007389043
O55026	0.9781318	1.014898904	1.07408115	1.119574137	1.09367166	1.101126549	0.956633946	0.969587291	0.920428458	0.941900494	0.930869834	0.937843427
Q149L6	0.991015254	0.990997656	0.986765116	0.994869818	1.01363698	1.00036477	1.025794926	1.016033006	1.019327485	0.952435594	0.983252708	1.027225865

Q3UHG7	1.052770175	1.044387447	1.055135996	1.015979717	0.922703086	0.983140439	0.952177524	1.011765762	1.119286801	0.992490953	0.937598957	0.92792569
P14131	1.064547123	0.985426908	0.965026148	0.969723194	0.983323562	1.034589391	1.011186303	0.976739071	1.06036513	1.002193738	0.971403574	1.005869742
Q9WV91	0.966049524	0.930528486	0.939488017	0.906386658	0.928389475	0.914558371	1.081893331	1.03182593	1.044521826	1.09124852	1.083590809	1.098780517
Q02105	1.004618301	1.099082715	1.129997303	1.167741519	1.158098763	0.978308709	0.799804371	0.958436846	0.934316913	0.944938603	0.930887985	0.953102084
Q8R317	1.173162005	1.219409753	1.367307345	0.789404525	0.791990065	0.749472412	1.000092998	0.972521889	1.434607098	0.686266889	0.898849662	0.714725013
Q9D1H6	0.911790271	1.041348099	1.04871339	1.100756462	1.017288354	1.021167192	0.934847192	1.016357488	0.909426519	0.863155174	1.047781297	1.062896526
Q14B80	1.191731724	1.075074884	1.003113285	0.902914394	0.980887271	0.971675905	1.016846587	0.970772621	0.989644491	1.025109837	0.94400806	0.990334031
Q7TNV0	0.982591511	0.979252903	0.975011161	1.026050879	1.113632302	1.023024223	1.055324678	1.02436933	0.929731825	1.057546819	0.921260576	0.985437626
Q91VA6	1.048778311	1.014873387	1.024008889	0.98393312	1.056636745	0.981261131	1.053731346	1.06307893	0.972965725	0.949821513	0.970064224	0.884465159
O70492	1.004850307	0.984994017	1.012693231	1.047545129	1.009777296	1.094053319	1.002003433	0.958764243	0.97519336	0.918822274	0.994524902	1.001736325
Q8BJW6	0.999841576	0.986775778	0.998432285	1.011192021	1.010289726	1.017760256	0.99532119	1.003870408	0.985744778	1.020038295	1.000210102	1.007148344
Q8CIV8	1.013711996	1.01563556	0.994072211	1.02893069	0.98621213	1.016702194	0.997755741	1.0268351	0.982531511	0.95026713	0.969484782	1.033902575
Q6PHQ8	0.979443371	0.958947797	0.981887612	0.994505758	1.012644453	0.986276543	1.037721956	1.023858617	0.970583774	1.050578264	1.018383708	1.024104451
Q8BFV2	0.959097855	0.898357526	0.954560732	0.980193351	0.906640269	1.050819546	1.096603864	1.004876382	1.09842441	0.947977811	0.95807706	1.126215502
Q99LB6	0.95509344	0.97002171	0.979756303	1.03598635	0.972185772	1.043813799	1.02400068	1.023921148	1.005228878	0.984879785	0.997292181	1.019969342
P51163	1.063895911	0.95900568	1.008217346	0.986421211	0.976694026	0.986268117	1.005163912	0.982498456	0.976593468	1.018675154	1.055418746	1.011289409
P97326	0.994397803	0.973355748	0.964783578	0.950165252	1.03412838	0.998649664	1.001466825	1.095251027	0.983442426	0.983481674	1.054386252	0.966228623
P52800	0.927923938	0.918670947	1.033479865	0.966392266	0.848572694	0.887473784	1.093000824	1.070072454	1.219770854	0.92763466	0.974217484	1.05818159
B7ZMP1	1.110466805	1.159346549	0.912705353	0.85701112	0.963656176	1.00854339	1.099642875	1.043549201	1.0085123	1.261270153	0.861836597	0.860849797
Q920Q6	0.982384622	0.938174952	0.98976889	0.973167861	1.004440412	0.97792793	0.995240579	0.938652517	0.988809397	0.989297596	1.069092012	1.167861657
Q9QZB1	0.95535893	0.975415411	1.030237667	1.069158623	1.029375182	1.040370825	0.980182213	0.993940414	1.000450437	0.878411528	1.030101858	0.986962241
P26516	1.060223199	1.028106911	1.025311388	1.016174596	1.033301576	1.083448151	0.948931026	0.899252364	0.926871925	1.012388855	0.98469962	1.050996106

Q9DBD0	1.121698256	1.122843824	1.151529536	0.825632156	0.808533568	0.757912271	1.257896118	1.135069725	1.155289165	0.851721596	0.87625872	0.827787807
Q80WS3	1.054493089	0.983352188	1.002290473	0.944929801	0.955241682	1.105159607	1.143211038	1.015546927	0.966542451	1.147667491	0.864116011	0.916423091
P83917	1.018673696	0.941488164	0.979211954	0.985769004	0.965244	0.980901199	0.987912298	0.997439358	1.021520416	1.111372462	1.044135961	1.026603973
Q3TIV5	0.972598487	1.007331808	1.045948825	1.093043514	0.980540513	1.063015897	1.004846768	1.003817044	0.986436087	0.767909818	0.990421486	1.017700899
P55194	1.021986194	1.00084171	0.93564763	0.962114888	0.986531779	1.028303581	0.977752745	0.976455696	1.080010872	1.119915344	0.97599595	1.009547372
Q3UVG3	1.036090087	1.014638104	1.043387774	0.901934022	0.882731974	0.938868597	1.047279584	1.020156962	1.091424495	1.054017122	1.009413883	0.967930129
Q8VE09	0.939345966	1.038339284	1.03399795	0.997552718	1.066858228	0.91635587	0.935370158	1.037905981	0.964362134	0.987057182	1.104615328	0.975974102
Q9WV54	1.204914939	1.124747466	0.993212773	0.97779025	1.249925108	1.191278208	0.816124658	0.87452795	0.928039752	1.04861738	0.901796414	0.829824921
Q8CHK3	1.064099888	1.021675088	1.037088628	1.015918434	0.976345061	0.980851057	0.997489581	1.004178877	1.028410253	0.976744163	0.960362255	0.957686757
Q4QQM4	0.984013571	1.013973721	1.047687047	0.956183123	0.953398473	1.004727962	1.037786787	1.004962704	1.006678476	1.073852637	0.997207715	0.955566848
P70182	0.800700447	1.099622054	0.932581202	1.018499228	1.028001601	1.049245872	1.057238089	1.029507559	0.855324928	1.204781039	1.092012287	0.920016091
Q9D061	0.969703865	0.987640649	0.979659244	1.038069782	1.065455821	1.068601597	0.961712374	0.980030625	0.991680226	0.944289865	1.010676015	1.014882663
Q5NCX5	1.046068616	1.064349879	1.02279688	1.007214679	0.947848817	0.932777355	1.017255637	1.078122983	1.017270108	0.892067859	1.00041206	0.941471644
Q62086	1.156696889	1.076595273	1.099590056	0.976919615	1.023593835	0.940663642	0.938891509	1.036366687	1.029687327	0.737116153	0.961180772	0.953429613
Q9ERT9	0.863736467	1.151788717	0.995261738	1.141929944	0.888466308	0.874245848	0.912218442	0.909627171	1.050387487	0.952246314	1.055017163	1.202955612
Q02566	1.459305855	1.411689203	1.073830477	1.096071737	0.992665454	0.941178789	0.838629318	0.869705693	0.869591581	0.785250955	0.883249192	0.842432632
Q6ZQ58	1.096721285	1.00244347	0.965517322	0.974952158	1.02064965	1.055025815	0.961039396	0.969873922	1.032967657	1.098362394	0.949906291	0.962089001
Q6P9R2	0.919941948	0.915338837	0.938878889	0.99252943	0.988894321	1.05191633	1.048616292	1.010981128	0.989506767	1.084265808	1.051387758	1.047967295
P62311	1.017456813	1.032736827	1.014215257	1.042480813	0.988745861	1.00061129	1.042895233	1.008882583	1.035716356	0.88907559	0.935317731	0.975391128
Q80WW9	1.035425102	1.105867177	0.982821017	0.950100545	1.030366844	1.003258357	0.967102209	1.008694325	0.974624575	1.13746261	0.958253233	0.943814444
Q9Z2L7	1.067385062	1.079553338	1.01261012	0.956455929	0.996845137	0.985154021	1.032839131	0.968979565	0.960036901	1.022331698	1.019989718	0.934292341
P56380	1.034362018	0.97822843	0.982022622	1.000165718	1.003089946	1.025427575	1.030965997	1.000805387	0.963131548	0.974048851	1.017876757	1.008932738

P39749	1.104683804	1.066929762	1.072956025	1.061148927	1.065652464	1.030319056	0.931071648	0.956148919	0.998489659	0.895605703	0.920968017	0.917264226
Q05CL8	1.002152836	1.018488055	0.986464254	0.966117132	1.0385854	0.997576713	0.999530923	1.056443189	0.940068383	1.051107189	1.016634305	0.97311236
P29788	1.29216529	1.314035499	1.239783906	0.641978581	0.638807274	0.594324044	1.434878421	1.281948732	1.394596791	0.745778839	0.622590038	0.609475255
Q9CY50	0.983568316	1.008527282	1.022981143	0.97966921	1.003984528	0.948278983	1.042315174	1.080662295	0.994624791	0.983925245	0.986729955	0.966493764
Q569Z5	1.034212391	1.005639674	0.989651271	0.988814638	1.001198442	0.987644797	1.019029498	1.006805336	0.959624663	1.023833412	1.020714047	1.000433016
Q9QXE7	1.038616859	1.008057167	0.989687015	1.016145717	0.960046421	1.020985273	1.016270838	1.013502645	1.014827977	0.972524444	0.969998151	0.998419471
Q8BRV5	0.907453021	1.172536978	0.913118502	0.956077261	0.954011898	0.96483151	1.03579907	0.967573168	0.972035839	1.005030868	1.095153566	1.060624974
Q8BH55	1.071373197	1.176075991	0.76329484	0.80106986	1.280557977	1.354778263	0.814604136	0.790395544	1.207836412	1.468304501	0.757990826	0.811475668
O89106	1.169992216	1.081064283	1.00858129	1.023725603	0.956832765	0.994796236	0.964047421	0.950113896	0.927717325	0.947724292	0.96047903	1.068149351
P08103	0.93352941	0.952617979	0.987058501	0.993520542	0.94852727	0.955440887	0.993028013	1.047728547	1.04073397	0.953235798	1.074274463	1.092565552
O35744	1.219633167	1.078321829	1.137526293	0.809104188	0.7522876	0.880899344	1.240164039	1.067611331	1.174162638	0.895260648	0.823111733	0.859876624
P62320	1.061691844	1.003190639	1.015210195	0.976839682	0.998972714	0.957367802	1.028171033	0.988741285	1.022691206	0.960953457	0.977952816	1.018900384
Q8BGA9	0.964685611	1.104596802	1.021248797	1.077487798	0.931870382	1.006351688	0.963679345	1.003639226	0.999874035	0.944239453	0.947549275	1.049584038
Q99LJ0	1.096118	1.049797857	0.986201759	0.966387038	0.967616817	1.001160855	1.007872742	1.081020703	1.102576882	0.898541634	0.91999558	0.903193373
P20352	0.976940081	0.956387169	0.996357643	1.001114578	1.038963195	1.09202065	0.989510044	1.002135959	0.96544438	0.998329095	0.996631002	1.018313596
Q9DCC8	0.996321118	1.081047628	1.009977128	1.075727501	0.833619677	0.96261973	1.052283402	1.079980342	0.95855583	0.825518456	0.972716277	1.104461191
Q9QZC2	0.977436774	0.971990793	1.094711685	1.063486727	0.962404022	1.017421629	0.921145989	1.040423924	0.949496981	0.940648111	1.055157327	1.000219358
Q6ZPF3	0.966760966	0.905722254	0.885687191	1.039880807	0.975789984	1.038935602	0.985035234	1.073216203	1.061216636	0.975513626	1.047469341	1.044840735
P34884	1.003278314	1.072288327	1.012490134	1.150509941	1.031133642	1.126626169	0.999181929	0.890322904	0.971676029	0.779478796	0.957843079	0.976788902
Q9Z1Q5	1.079015219	0.931311133	1.020797137	0.88934466	0.90749865	0.956269933	1.153129389	1.062723137	1.104769478	0.928086365	0.970039774	0.95038954
Q8R4E6	0.960519109	0.824813984	0.881567437	0.901886553	0.970373118	0.939113598	1.095163289	1.085155864	1.070136071	1.098732939	1.09921083	1.085719371
Q3UMB5	1.054363203	1.068169767	0.971197395	0.984909501	1.028475883	0.984935316	0.989252609	0.958941204	0.996899108	1.221854174	0.93336425	0.948381156

Q9DBY1	1.377230685	1.191377677	0.691929259	0.636229635	1.322654735	1.284570329	0.79056131	0.753126708	1.270205593	1.805786937	0.704465864	0.639520437
D3YZV8	1.16270285	1.093882784	1.132696208	1.163576211	1.167903031	1.216634019	0.845184955	0.886133136	0.831363178	0.849155424	0.862941743	0.860845927
Q8R2H9	1.173697562	1.00602844	0.950067923	0.839422014	1.052560586	0.955970564	0.976996393	0.927984986	0.948783549	0.867857421	1.338394991	0.89385155
Q14C51	0.978870041	0.963215413	0.94881597	0.939253322	1.045155773	0.951905331	1.014004192	0.955605342	0.973119171	1.105443087	1.001437434	1.200784879
Q61599	1.105554578	1.020812272	1.110894049	0.950631015	1.048313039	0.893775514	1.06082737	1.057124904	1.013666464	0.948076094	0.944837318	0.839325418
O35454	1.035401944	1.05665777	1.00243024	1.065899606	1.043219746	0.966579662	0.983936582	1.012150692	0.92431581	0.971754549	1.024772768	0.942589164
O88735	0.982803211	1.035632176	1.004013405	1.053948126	1.007827194	1.001462318	0.992328988	0.962905101	0.95107221	0.933097153	1.049769088	1.027309996
Q8VHX6	1.043717038	1.026329752	1.015186199	0.945293114	1.129886628	1.053771054	0.984633008	0.967816472	0.912093086	1.041223383	1.018866432	0.92126697
Q9Z2G6	0.943149174	1.029008679	1.002124933	1.104863161	1.047824202	1.00404939	0.961843141	0.939344473	0.99491833	0.955131935	0.975971314	1.068157971
Q3TIR1	0.964710024	0.987432719	0.986553486	0.955659876	1.067012613	1.002717793	0.98430975	1.035088807	0.903010331	1.112621605	1.064830865	1.00404982
Q9CQ49	1.023617596	0.991757744	1.010326773	0.975267045	0.987101505	1.069899451	1.055011297	1.013509056	1.019304053	1.086546424	0.915840861	0.915627165
P56212	0.881639788	1.244521564	1.055129191	1.23342603	0.708936206	0.981563862	0.931550083	0.915845917	1.022903449	0.750618621	1.044200469	1.146655886
Q9QYE3	0.881394004	0.951716612	0.975083157	0.971702777	0.967370219	0.940212618	1.056643553	1.035190214	1.026550283	1.14999906	1.053364239	1.035299567
Q9CQK7	0.945426169	0.945864904	0.994327902	0.943517713	1.282974292	1.034130749	1.003756829	0.966012649	0.957886508	1.103956061	1.016310447	0.87654185
P63042	0.955483302	0.950356867	0.95927831	0.99339647	1.064444638	0.950346839	1.033055752	0.975727708	1.014763106	1.02539637	1.007560095	1.101391843
P70169	1.12718334	1.000228172	1.021865228	0.988600749	0.988807378	0.997376198	0.990871729	1.040619339	0.964986946	0.9455845	0.978565897	0.976035346
Q7TSH2	1.065841142	1.037310297	1.021916023	1.019896435	1.051520444	0.990478093	0.942899527	0.931011431	0.990431693	0.914127547	1.037693294	1.002337432
Q9JI75	0.975886629	0.958651583	0.991416364	0.998213925	0.945898665	1.039779391	0.984568395	1.000678005	0.976716829	0.992935591	1.036702663	1.117455412
Q9QWR8	1.076528872	0.984130215	1.008254616	1.055499453	1.019514477	1.196493464	1.00618874	0.976788159	0.930917546	0.997662799	0.889977444	0.931176382
B1AXH1	0.932212821	0.968388856	0.965316897	1.020296027	1.04606692	0.993570431	1.022653178	1.083544372	0.975805926	1.0090474	0.995796644	1.008445158
Q9DB27	1.009504339	1.034245004	1.026105341	1.044386834	0.993604724	0.999145344	0.937150398	0.972163051	1.059040 3	0.914406288	0.975429429	1.031917109
P97470	0.976003038	1.029439422	1.017296896	0.959862337	1.030613687	0.963923345	1.000804688	1.035412471	0.991816727	0.94208065	1.054236452	0.978922604

Q8CH09	0.996561275	1.001960153	1.032783786	1.013154132	0.99440593	0.891825507	1.006161558	1.099989787	0.96786034	1.114832765	1.039115169	0.892033088
Q922H4	1.028743249	1.1526878	0.978951576	0.989307776	0.976620397	0.919859627	0.975864546	1.056172484	0.943052606	1.099420249	1.038392903	0.905018501
Q99K30	1.066242889	1.121296766	1.069397003	1.003250558	0.999166297	0.962882294	0.963701277	1.017203959	1.013562768	0.941576524	0.949535994	0.901863927
P56565	1.058071529	1.124069136	1.137646084	1.049760925	1.283566419	1.110365898	0.886676983	0.968224437	0.807206889	0.79648468	0.963114748	0.818326693
P85094	1.177370205	1.073904451	1.102887944	1.082204005	1.067271528	1.097326359	0.888841798	0.938420533	0.938599447	0.746886043	0.927374483	0.944475309
Q9DCS3	1.038300261	0.957505514	0.989477409	1.037829613	0.989704387	1.049315135	0.98124004	0.970121413	0.971894697	1.042958328	1.007325957	1.023243006
Q3UUQ7	0.981550593	1.004900314	1.081572833	1.092420751	0.971541394	0.930530551	0.916114207	1.067056271	0.988231781	0.923965624	1.000788897	1.035687068
Q91WG4	0.998148774	0.931670519	0.981892508	0.993905786	0.909619178	0.970836401	1.064984668	1.030107798	1.124137749	0.852074134	0.96834834	1.113845935
Q9CZN7	1.011720769	1.007638882	0.978634761	0.994151664	0.969924832	0.999348132	1.042528575	0.978777235	1.019143422	0.991626745	0.999388142	1.024292638
Q8R555	1.073565284	1.022888628	0.969566774	0.969369281	1.003623696	0.999262801	0.943290316	0.98263155	1.034872461	0.908727685	1.045295664	1.037868104
Q8BTX9	0.975026975	0.930343236	1.022238899	1.009586897	0.992075935	1.028259141	0.95858092	1.102438103	0.96168268	1.005880035	1.042547396	0.986356137
G3XA57	0.938425008	0.968953411	0.988059094	1.00588579	0.963278646	0.997614814	1.018713302	1.030451359	1.077138533	0.970211009	0.990774906	1.040442149
Q9DCH4	1.015069036	1.019471048	1.031026482	0.979379629	0.98025666	1.041292966	1.057535422	0.99383058	0.980057915	0.943436496	0.965272599	0.996095023
Q8VDH1	1.17893335	1.113639031	0.87094418	0.823972573	1.077534995	1.134517821	0.911967498	0.937666607	1.070975601	1.541990162	0.834272957	0.805810355
P12657	0.889002969	0.903205269	0.934566391	1.020045116	0.981176474	0.966364542	1.059522414	1.02278189	0.994159086	1.078916743	1.094399673	1.079888254
Q08024	1.013791693	1.079568127	1.088481546	1.006380138	1.082905853	1.034211787	0.995093188	0.95929599	0.940213706	0.984393873	0.929425559	0.928492075
Q9R0A0	1.05838597	1.045777198	1.05062698	1.007266923	0.984166709	1.058607757	1.006266477	0.964496603	0.999035133	0.873112688	0.948397858	0.992765748
Q6PDH0	1.086506558	1.05366404	0.914383894	0.928590281	1.126202216	1.098946109	0.971750808	0.941944978	1.05432632	1.216781174	0.886349533	0.871556847
Q811U4	0.999636337	0.99777015	0.951936938	1.022665973	1.230457345	1.068939929	0.925369441	1.005771102	0.914389931	1.046725033	1.012731339	0.90035043
Q9Z0R9	1.030147057	0.976524437	1.030646699	0.95267498	0.984751814	0.959541035	0.988433218	1.066518565	1.063180583	0.983567666	0.984636343	0.979981607
Q9CR67	1.005724098	0.966520502	0.991087753	0.974358979	1.009321073	0.935527748	1.027195512	0.95740817	1.033744391	1.102997261	1.02666098	1.024370195
Q9CYK1	0.985942152	1.024314032	0.988511837	0.974067369	1.075592795	0.960532682	0.967068098	1.029722522	0.964645932	1.026803042	1.013185275	1.029689994

Q8BTV1	0.931258588	0.923228943	0.942373437	1.000167654	0.929116771	0.949191601	1.022930024	0.957055963	1.041612069	1.068070645	1.105638325	1.151433803
P49769	0.91420956	1.640041943	0.836914202	0.900349849	1.045512626	0.925042805	0.937643736	0.96410581	0.756859672	1.253525434	1.066406431	0.922461537
Q8BWZ3	0.9724958	0.96452752	0.979689134	1.048085977	0.929764246	0.96830601	0.970573116	0.988688423	1.033408787	1.037725207	1.034182734	1.105564446
Q9DCT1	1.021992027	0.984319972	0.952769806	0.968944278	1.014157941	1.032336233	1.055823054	1.040795131	1.007148778	1.072971581	0.95886744	0.944957444
Q9CPX6	1.010506412	1.024064735	1.000169673	0.991699236	0.990917698	0.977720439	1.038962085	1.014950277	0.996380281	1.010343602	0.999837703	0.966601458
P60603	1.081737836	0.926734356	0.927395239	0.942520988	0.92284722	1.219930994	1.087841357	0.96759893	0.909410195	1.335052845	0.921131271	0.953072006
Q66GT5	1.063316817	1.054186999	1.109149218	0.958632066	0.918384008	0.95960327	0.984401703	1.046579801	1.025157063	0.984715769	0.957812655	0.946802098
Q61120	1.00518359	1.081484301	0.999631141	1.03673378	1.030142038	1.000012556	0.966742424	0.999617704	0.951647556	0.965843508	0.999460608	0.990506653
Q9CQ91	1.084331787	1.072524596	0.984835236	1.031457427	1.020566447	1.036528383	1.032370437	0.956326179	0.963904649	0.90102421	0.986754573	0.936139021
Q9CQH3	0.998226417	1.154880141	1.033479939	1.043320834	0.902435329	1.06462311	0.995575013	1.012918976	0.947468229	0.754969614	1.028608362	0.988077875
Q08642	1.012794353	0.991153863	1.005771126	1.038817783	1.067137554	1.050887218	0.981868286	0.970477943	0.976599942	0.978857758	0.980561935	0.980111709
Q9JLC4	0.871114732	0.952581886	1.02010335	0.976283494	1.042674839	0.898499604	1.050408294	1.025839573	1.064128121	1.038718691	1.061490897	0.98703798
Q9DC29	1.118181797	1.186110777	0.992890485	0.817806753	0.930726323	0.963199908	1.021859959	1.064964153	1.165578373	1.235000572	0.828861783	0.785413904
Q8VDC1	1.019284098	1.011726952	1.036466244	0.931312041	0.998884067	0.941405928	0.875908769	1.110358542	1.059631379	0.984088013	1.013063451	1.020642932
P54731	1.036047928	1.032216536	0.988694827	1.005939353	0.949848202	1.016446671	0.997803217	1.008092498	1.039719031	0.948040486	0.976519563	1.00579472
Q9CZU3	1.026366942	0.977859627	0.936068114	0.952909902	1.044104317	1.026331991	0.995093777	0.971880505	0.990665189	1.21122012	0.990910489	1.000653034
Q8R086	1.046628303	0.968132251	0.98854932	1.011677773	1.006504234	1.035702719	0.996953392	0.985563144	0.988640958	0.964719545	1.006716825	1.021117413
Q8K2C6	0.973412334	1.034037044	1.006559539	1.041502246	1.02212438	0.970404132	0.973140643	0.976223043	0.94245374	1.148911174	0.99966399	1.011011114
B2RY56	0.991323764	1.072554951	0.994045108	0.9879853	1.019223059	1.019768349	0.987326222	1.004769604	0.93724565	1.019236304	1.018256902	0.987698274
Q9D1L0	1.030046155	1.030031181	0.984695007	1.138662231	0.926031046	1.007768006	0.972526396	0.894670972	1.205599042	0.62191624	0.896583524	1.17950842
Q91XF0	1.038121946	1.025781693	0.998926128	1.066110228	1.008356399	1.071163688	0.937005127	0.973477206	0.941788419	1.007079281	0.995881211	0.994632532
Q9QUG9	0.938944455	0.970263076	1.018021469	1.04147409	1.104436843	1.035436121	0.999009172	0.983834728	0.953083296	0.960231021	1.001124403	1.010421087

Q63959	1.168071758	1.070378862	0.97615871	0.976954553	1.069088768	0.958690504	0.972947421	0.953269448	0.965276849	1.091377011	0.98889105	0.905652137
B5X0G2	1.105787326	0.980331471	0.968753788	0.843222957	0.816562647	0.7991259	1.23692496	1.222543365	1.252978155	1.045499827	0.870992802	0.824510876
F6W8I0	1.052431341	0.960720349	0.983311797	1.000550359	1.033344382	1.060239821	0.982733837	0.941660696	1.027217036	0.963027521	0.986307466	1.034184814
Q63943	0.931586824	1.178948069	0.960190939	0.973284726	1.042683389	0.978568614	0.993936428	0.962279058	0.961996062	1.155828517	1.00411126	0.947682218
Q8CCX5	1.080100421	1.129791471	0.911388055	0.972284771	1.036825075	1.034806286	0.976669891	0.979449744	1.001955434	1.16064459	0.953868572	0.880185651
Q3UA37	0.873352894	0.937528872	1.005618957	1.012476068	1.010660815	1.021586239	1.113314695	0.928524222	0.941690946	1.187978617	1.06396726	0.978972611
Q8VE97	0.984792426	0.966996237	1.055194164	1.056760964	0.981580557	1.023055672	1.024998571	1.001915214	1.003976063	0.923321152	0.962994196	1.00821525
Q8R3R8	0.956241134	1.053873975	1.017093223	0.986214526	1.105744339	0.998846101	0.938175895	1.093256454	0.918691746	1.029520539	1.01209191	0.930837158
Q8QZV4	0.932529122	0.962169102	0.991090328	1.043887096	0.959574132	0.976651642	0.93240403	0.984225348	1.010933756	0.941334101	1.090647033	1.162395924
Q5SVR0	0.971891354	1.044958543	0.971788031	1.008716831	0.935062939	1.000547599	0.989304123	1.024870693	1.021198571	1.007246258	0.996409589	1.050264338
Q9D8T7	0.941857652	1.046669128	1.070211614	1.048337212	0.947554736	1.048409462	0.973598648	1.013182554	1.042060084	0.830919257	0.986305604	0.992958483
Q60996	1.043354866	1.032218349	0.956290895	0.982424584	1.096211838	0.988329122	0.891977796	0.94112981	1.052464857	1.238443986	0.986274337	0.933348055
Q9Z1T1	1.060541826	1.068653748	0.964004783	0.955952117	1.021183646	1.052001301	0.954168751	1.00735157	1.052698175	1.187940194	0.909081212	0.887238295
Q80U04	0.99716442	0.996189929	1.073161712	1.026591637	0.993480861	0.966967952	0.946927154	1.017891096	0.930079963	1.104597929	1.00384974	1.019330169
Q9JHS4	0.959578101	1.014761991	1.012535419	0.982644725	1.019433988	1.012015588	1.001038754	1.006484752	0.973779734	1.008260139	1.020237369	1.010326701
O08739	1.005586049	1.029329167	1.055559454	0.988794285	1.05225303	0.994004145	1.004712963	1.035632362	0.939359759	1.006253384	1.003993602	0.910907229
Q8BLJ3	1.004070238	0.945197727	1.050522598	1.011691589	1.009189669	1.080197073	0.983040992	1.067342599	0.998048608	0.838535221	0.943279101	1.034435246
Q9DCA2	1.04712804	1.012160021	1.047033596	1.028240692	1.043162139	0.907740942	0.885278701	0.95717829	1.048867266	0.957198096	1.053500217	1.021409539
P35290	1.008437726	1.018090492	1.089566267	1.10459654	1.022399834	0.935406171	0.900750896	1.006830454	1.035175209	0.78879337	1.004735683	1.028739116
Q02956	0.9609323	1.028879665	1.031977676	1.002147837	1.143051622	0.928614101	0.939172346	0.972971261	0.94490629	1.041755906	1.062202269	0.987146463
Q6PD28	0.952725272	0.979848481	1.043370118	0.968398689	0.998861571	0.96832052	1.010907936	1.032171024	0.946257959	1.088862707	1.05268926	0.999014192
Q9DAI2	0.950711934	1.007470983	0.939562211	0.975369923	0.962346291	1.086726958	1.064067949	0.984680698	1.023696838	1.007321948	0.996268689	1.018318853

Q8K4B0	0.976361598	0.989168918	1.003638711	1.039380423	0.976706943	0.971785358	1.001704032	0.963245172	1.004871782	0.942185876	1.030307575	1.098564703
Q8BG73	0.957646688	1.060297059	1.070734122	1.146642492	1.266115919	0.90714685	0.822452422	0.914203618	0.845972656	0.807555026	1.16221211	1.015024963
Q8BTS4	0.956305585	1.010612471	1.016703029	1.024272365	1.038125404	0.982194528	0.963420595	1.021562862	1.039964624	0.906064281	1.008508336	1.008792861
Q3UDK1	1.100974128	0.997206659	1.099625478	1.002492581	0.957979803	0.922733197	0.975822552	1.105784941	1.032141666	1.027064729	0.951915618	0.854645392
Q80TN4	0.988306582	1.010017941	0.938825653	0.953119801	0.967069646	0.975110418	1.026589496	0.985909988	1.051484902	1.169469545	1.025461377	0.984684317
Q9D706	0.937570934	0.981173376	0.973475315	0.981611044	1.024612156	0.943997555	1.018785321	0.999914931	1.039586235	0.964227942	1.069311016	1.047561433
P56135	1.068570505	1.082777205	0.999884902	1.044330294	0.97796151	1.09615885	1.007092105	1.044460684	0.938849905	0.812746171	0.959794985	0.940172269
Q8K1B8	1.093039053	1.067261498	1.109285355	0.933329805	0.965083906	0.920172089	1.020754237	1.076552613	1.046931841	0.924609893	0.944206539	0.876309774
Q8BTG7	1.068455654	1.001933369	1.073701787	1.083353717	1.105824522	0.999165642	0.881744117	0.932832003	1.007161134	0.795499006	1.002369337	1.019412868
O35409	1.260366113	1.198620398	1.126014736	1.069077703	1.081980474	1.066054951	0.895957496	0.924523215	0.903492052	0.844194885	0.851139886	0.829292509
Q7TSY6	0.938653137	0.92698974	0.992458395	1.018071667	1.001550824	1.037658461	1.038269207	1.014694643	0.994648191	0.973821581	1.006005053	1.061572656
Q9JKC8	0.944585687	1.010513119	1.046714183	1.054649351	1.001407406	0.804625395	0.853032768	0.934704997	1.142701877	0.626863367	1.204996684	1.201020013
Q3UHD3	0.979253202	0.967367292	0.924283194	0.931541825	1.007542069	1.059021415	0.988017117	0.996149541	1.066323372	1.318197729	0.971508373	0.939464195
O35143	1.256065093	1.205923904	1.157141442	1.171554955	0.815379092	0.962655797	0.95648684	0.848632485	1.028265158	0.813587414	0.814595925	0.990647046
Q80U72	0.945726885	0.978612792	1.019328126	0.969352343	1.048129244	0.950852156	0.937469362	1.023716383	0.922266231	1.36074914	1.035394281	0.979315524
Q04735	1.079261225	1.004277235	0.972146654	0.975682192	0.95657522	0.930674068	1.008065253	0.948632429	1.045230136	1.06248207	1.038346315	1.024466871
P62996	0.985360959	0.938015838	1.034801018	0.95324369	1.241403734	1.0295093	1.072354132	1.023186253	0.897709648	0.893947917	1.044975695	0.861726281
Q7TSQ8	0.919173484	1.073069408	0.953304962	1.0173029	0.966720755	0.997471892	1.033572857	1.002651151	0.97043564	1.003755864	1.03978699	1.037238544
Q7TNE3	0.9625672	1.051430228	1.022965907	0.985572663	1.071563743	1.094198982	0.958506283	0.965265507	0.967365076	1.027896428	0.982735057	0.958099115
Q07235	1.119023285	1.033958102	1.039353254	0.970994296	0.960421189	1.021316522	0.993828258	1.001102823	1.025166453	0.91026934	0.941279164	0.986220127
Q80ZD8	0.859093578	0.908533285	0.960834378	1.053632863	1.016159817	1.027740296	1.026561318	1.008713414	1.025124637	1.001134841	1.035672497	1.080104008
Q99LC8	1.02262307	0.990790308	1.05993737	1.084490762	0.959339473	0.953460301	0.939347844	1.048621772	0.981741906	0.858565866	1.012577123	1.060968871

Q8BGZ4	1.126226581	1.074317876	1.021592762	1.055618157	1.132639487	1.072341157	0.886618321	0.995574976	0.92280014	0.945703367	0.952516129	0.872641509
Q922P9	1.084617833	1.023554772	1.035528015	1.046320172	1.032885621	1.006837499	0.96171895	0.993802353	0.98829567	0.881784263	0.994843776	0.944174563
Q8BTU1	1.011647176	0.978090412	0.959706164	0.947464761	1.061195416	1.07512196	0.946893073	0.997126415	1.024660668	1.118192604	0.980207792	0.98253419
Q61733	0.972807218	0.980608553	1.014271917	0.980701307	1.043499047	1.021420194	1.038459581	1.047261274	1.001664082	0.947028985	0.968698314	0.979077922
Q8BGV0	0.993546227	0.985769583	1.002009233	1.035744914	0.994858739	0.984124748	1.000769846	1.054990212	0.988288734	1.047021387	0.985366498	0.970931187
Q99J23	1.00985901	1.018880682	1.01421106	0.998581058	1.005177788	0.993165973	0.981955264	0.982241574	0.979517148	1.023530133	1.039113562	0.98610321
Q9JKL4	0.948426302	0.938269205	0.939352714	0.931755375	0.976473571	0.999872743	1.085973428	1.052297182	0.971264177	1.151458825	1.045237832	1.020014547
D3YZI9	0.93254876	0.979411791	0.970416487	1.01929886	1.005178179	1.014964096	0.990348339	0.980211629	1.012246616	1.127392699	1.022488148	1.014562478
Q9CSU0	1.009916927	1.028829571	1.039834263	0.953650921	0.941794894	0.937065314	1.007367046	1.062417247	1.01155801	0.982144828	1.017130114	1.004059633
Q9D6F4	1.029836143	0.95709175	0.976625931	0.93536807	1.032501049	0.978796791	1.040083114	0.974804985	0.994620248	1.175081355	1.004725596	0.994657591
Q80U30	0.976967335	0.946345837	0.975949411	1.006593846	0.973799828	1.033737945	0.990429348	0.975038744	0.988890775	1.152170799	0.996094862	1.077271089
Q9CYL5	1.18473595	1.093022434	1.158562931	1.018775684	0.954094127	0.922607728	0.991633423	1.118349219	0.983435208	0.921903175	0.837687311	0.83451009
Q8CFI5	0.925803652	0.975432646	0.984998627	1.016244719	1.076949473	0.958714055	1.001674388	1.032831457	0.959660367	1.074124778	1.060883236	0.97340219
Q8K1R3	0.981472917	0.996664459	0.946750238	0.987639248	0.995052531	0.998874793	0.997918738	0.974240652	1.007827158	1.001410838	1.057968231	1.072128923
Q9JJG0	0.947147899	0.990580847	0.991660782	1.043390729	0.99069785	0.98442986	0.979146584	0.985048738	0.968614322	1.107077645	1.04479773	1.032783341
Q8CHP5	1.005968634	1.067233354	1.01480582	1.067262846	0.966593962	0.959730749	1.072810581	1.020553315	1.01538347	0.935383798	0.928831063	0.94604638
P62855	0.972308081	1.049448306	1.025263501	1.04276598	0.977211395	1.037713553	1.013397115	1.03655466	0.972276943	0.841455161	1.004017434	0.983480799
Q91YR7	0.964676987	0.929675137	0.977928058	1.007649485	0.954490272	0.982941877	1.012053271	1.039010134	1.002759981	1.045030859	1.051512174	1.056492571
Q9D666	0.936504223	1.641474894	0.895998165	1.18721725	0.988303444	0.962407519	0.853552187	0.909738302	0.852045034	0.873623758	1.045606833	0.881357787
Q9CXF4	0.989640696	1.011355514	1.006181508	1.022918842	0.956428467	1.036402256	1.027941739	1.002588583	1.01268867	0.919761601	0.986972618	1.015097167
Q3TIX9	0.995071514	0.952653025	0.998391711	0.998638763	1.041850423	1.053014142	1.016524924	0.98420234	1.014442072	0.978702852	1.000023071	0.98046829
Q8BGT7	0.935042503	1.010171702	0.99448772	0.981515141	1.055036557	0.941501245	0.995861821	1.01977494	0.903902608	1.19857111	1.067868743	0.994237501

Q9QYK7	1.011423261	1.033241172	0.997162201	0.990846742	0.961436909	1.029539056	1.027764107	0.985093464	1.055237969	0.971080545	0.955812565	0.99152015
Q7TSE6	0.983965018	0.987387158	0.997311662	1.007279632	1.017948675	1.009429385	1.027427128	0.975587695	1.013257458	0.997065185	0.998240934	1.0051055
Q61418	0.901677357	0.937725107	0.938550069	1.010302315	0.918274401	1.007783019	1.050014827	1.045934679	1.035985851	1.004889052	1.034112423	1.112740175
O08586	0.950909571	0.980011867	0.995544137	1.0282462	0.974557265	0.973322158	1.027471327	1.051986404	1.056586445	0.90717267	1.017114008	0.997955696
P98086	1.163782786	1.160531605	1.04711192	0.998002123	1.182048781	1.063601895	0.847166162	0.909192808	0.879195879	1.186887556	0.869608146	0.885788907
P61458	1.078892545	0.964849374	1.12872514	0.998585391	1.027376182	1.080431835	0.94418781	1.011670357	0.951537019	0.975262657	0.936471427	0.940226439
O88444	1.029162866	0.992440218	0.966179552	1.027108776	1.023134311	0.999155423	0.995544075	0.964512457	0.984911882	1.068793536	1.007170555	1.008486883
Q99KI3	1.018682819	1.317189123	0.97386648	0.963866047	0.978572472	0.948710996	1.022201398	1.041720084	0.983335529	1.056318733	0.905806199	0.849571212
Q7TMW6	1.089500838	0.959503718	0.913758155	0.958141302	0.908028315	0.953010849	1.041661405	1.103781726	1.130009832	1.251639356	0.915746175	0.890590647
Q80ZJ7	1.025443822	1.007951346	0.977310337	1.021411655	0.966787324	1.076637501	1.002415628	1.016393471	1.052262069	1.059041375	0.883545716	0.978247678
E9Q735	1.053288038	1.001550503	0.965377783	0.969708004	0.943188117	1.001117112	0.994334312	0.947564062	1.044157817	1.006699983	1.028638169	1.070550712
Q920P3	1.062369249	0.984746485	1.11473917	1.047858612	0.973411373	0.849418747	0.923784564	0.998676467	1.048605273	0.815053896	1.053608426	1.064992426
Q8R349	1.025524681	0.975516402	0.970514979	0.98774873	0.964852926	1.017079312	1.039445033	1.006470169	1.006806262	1.104868862	0.963899716	1.008914987
P38585	0.933204601	0.915450007	0.949831666	0.986055436	1.056902247	1.014756147	1.040436735	0.978554285	1.029694264	1.086159492	1.051834113	0.995859003
Q99K23	1.004899501	1.013224408	0.970137475	0.967673188	1.048138229	0.972674095	1.00251497	1.005075606	0.965963573	1.046196114	1.037581117	1.008840285
Q9DBR1	0.947217248	0.93918931	0.986398529	1.037013586	0.998782361	0.903660782	0.968288905	1.071075392	1.018384543	1.153609193	1.006812691	1.045782691
Q8R066	0.994345396	0.947626302	0.987539486	0.975050295	0.924428848	0.973928054	1.082965195	1.086316487	1.072060644	1.008593502	0.935058666	1.019013609
Q9ES46	1.175076755	1.11477508	1.099538173	0.907369765	0.970070964	0.892293354	0.951952816	1.027171271	0.990171131	1.003000102	0.97801103	0.924834834
Q9CQC9	1.018069768	0.981154678	1.054332956	1.017606015	1.034337494	1.031983034	0.989293448	0.980502161	0.943021503	1.038505587	0.990127189	0.974738434
Q8CHW4	1.503721515	1.013716926	1.694501049	1.021435439	1.137048648	0.811371901	0.809977133	0.785116333	0.723065939	0.873832923	0.890669702	0.810398798
Q99J45	0.997068719	0.929455923	1.016306921	0.949971336	0.9786578	0.995462606	1.019009382	1.011581119	1.077732382	1.036737511	0.961885659	1.051277338
Q9JMC3	1.06707046	0.999137319	0.961229193	1.013560595	1.029397412	0.975243593	0.980827378	0.978649164	0.973705692	1.103013913	1.027083503	0.97263071

P97813	0.984106438	1.026888957	1.002397657	0.975846239	0.982808931	0.995646716	1.006303543	1.024186487	0.979124085	1.019499805	1.005775013	1.024979959
Q8VED5	0.963837961	0.859472153	1.000488485	0.995190618	0.727620902	1.089794779	0.820667717	0.757901851	1.127481876	2.265554419	1.052366708	0.868774574
Q9D882	0.945161969	1.095113344	1.021343201	0.988255987	0.907525186	0.983181187	0.989337269	1.007786261	1.052375567	0.955233285	0.998836063	1.042033767
Q9ER58	1.177264485	1.237577681	1.208122613	0.929848682	0.97399842	0.833480151	0.980481242	1.083050948	1.17572101	0.607913299	0.778049502	0.879993347
Q810J8	0.955639505	0.988230223	0.987604115	1.035778107	0.874710072	0.91232013	0.92770758	1.034565106	1.152072731	0.769144053	1.074855511	1.179719911
Q8BHW2	1.032689873	1.052223509	1.048262464	1.047275018	0.987755854	1.066298846	1.063863904	1.040867637	1.006058447	0.7707234	0.940476578	0.875050111
O70293	0.867622876	0.943060314	0.978916864	1.02870577	0.926255144	0.879121371	1.018959493	1.092199125	0.996791443	1.010923408	1.147281831	1.084352137
Q60612	1.050882229	1.01610215	1.038562517	0.959785403	0.918267376	0.952084236	1.050563542	1.016574453	1.055421084	1.003407587	0.954765506	0.99696032
O35099	1.056748811	1.029557894	0.932554489	0.949472419	1.012964231	1.000048768	0.97553788	0.98208839	0.969133934	1.143030641	1.023636795	1.022768735
Q9CR21	0.913356245	1.113390307	1.054385347	1.239047645	1.082189924	0.964758299	0.963292998	0.967265236	0.981315549	0.842133693	0.952554427	0.90934292
O54950	1.053175794	0.990929451	0.955098697	0.927362787	1.072238667	1.030727078	1.005719731	0.967088137	1.01915093	1.209240114	0.950615046	0.943691117
Q9JI99	0.987209006	1.064432412	1.029048498	0.987600386	1.034785686	0.894487618	0.990817937	1.012379828	0.954453793	1.156418302	0.979396114	1.002796052
O89079	0.979546025	0.975819	0.991970912	0.984204947	0.998507792	0.991954217	1.044664372	0.977316092	1.011972667	1.023068235	1.010238016	1.034864503
P35550	0.994216132	1.00281591	0.99030815	0.990127191	0.973956363	0.931848268	1.040219057	1.047715132	0.977820221	1.179063784	0.983529784	0.979830272
A2ARP1	0.966155898	0.994820148	0.991349433	0.983063933	1.011590783	1.025956481	1.050872788	1.000569468	0.99275642	0.929319216	1.04231706	0.98961478
Q8K207	0.938848171	0.951195038	0.986398178	1.005400353	0.962584546	1.01280433	1.016413691	0.974670437	1.032480339	1.030950276	1.041001706	1.065378872
P04104	0.905869157	0.919800133	0.930751274	1.053411953	0.903199014	0.968646269	0.91403756	0.838688119	1.18511628	1.649014507	0.987246428	1.022669715
Q3UMB9	1.050087564	0.970947312	1.001914728	1.001872666	1.000203127	1.0634105	0.999949941	0.937048102	0.954914804	1.046554331	0.988173381	1.051873312
Q8R0X2	0.990864678	1.044091272	1.013214058	0.990909097	0.959236262	0.955941012	1.038129488	1.02929055	0.986729685	1.031034721	0.966541343	1.02820003
Q0P5W1	0.941430111	0.968197271	1.039819842	0.997554341	1.014565941	0.963678354	0.998506333	1.041785587	0.997405756	1.028969427	0.998488012	1.032391816
Q8VCH5	1.066786303	1.062629457	1.008831692	0.945157771	0.972374348	1.007578351	1.001072125	1.001653846	1.053771289	1.076219759	0.924354422	0.941363221
Q8VE19	0.976654318	0.918350104	0.987748787	1.127422588	0.948065071	0.931453508	0.894253907	0.983747864	1.072767504	0.87020739	1.108342734	1.140331435

Q8BIF2	1.006499053	1.008957221	1.0118743	0.954647988	0.969182627	0.972370074	1.034533034	1.085229726	1.054976123	1.054342076	0.946682792	0.92947489
Q3TRM8	1.142943312	1.218331082	0.95269566	0.901798832	1.091143811	0.989531278	0.898903819	0.870436168	1.074623721	1.213110051	0.918321897	0.881311193
Q80UY2	0.921082387	0.904837146	0.999513602	1.062261137	1.037469091	0.983275981	0.958545721	0.981045713	1.003401564	1.039385937	1.080949146	1.055514211
Q8VDS4	0.973309302	1.17317906	0.981129968	0.894544967	0.945420239	0.893568941	1.08127171	1.065587084	0.944637504	1.078336799	1.051055417	0.943223932
Q6PE15	1.024820995	1.011172397	1.085596472	1.095122718	1.107728634	0.937548456	0.862561375	0.948879046	0.967904612	0.824644153	1.051128844	1.058218641
P06537	0.997221453	0.962908076	0.946499516	1.0201319	1.00538927	1.013855315	1.025798792	0.977951145	0.98374204	1.047026031	1.031997176	1.033407232
P58069	1.114830807	1.069010545	1.058509999	1.037949549	1.035124168	1.002846036	0.909086886	0.956622006	0.957840011	0.844150577	1.035280559	0.965754523
Q3UGS4	0.976361483	1.056040452	1.012380284	0.981028548	0.955643135	1.006509599	1.049808853	1.00968248	1.01391275	1.011967416	0.987759004	0.953309496
Q7TQG1	1.088740418	1.013093038	1.04358924	0.991845193	1.069135409	0.959708174	1.024963468	1.118630552	0.95630342	0.943403312	0.951979627	0.846936297
Q3UMY5	0.952231502	0.936312843	0.959215286	0.96294326	1.076943521	1.067574662	1.027782499	0.967360411	0.961670839	1.332588143	0.965470312	0.960217358
Q64310	1.057542827	1.077145866	1.045318277	0.916550974	0.983274029	0.959957217	1.045825115	1.025212031	1.025068954	0.983948476	0.957795047	0.930127101
Q5RKR3	1.024502825	1.120136233	0.922719274	0.85640782	1.013637306	1.053736732	0.976305799	0.977014175	1.016794579	1.304568837	0.98858956	0.896090909
Q9CQN3	0.965108399	1.042723231	0.934998702	1.002434717	0.995428866	1.003364943	1.068471142	1.021940297	1.047646626	1.066404051	0.943665238	0.953431914
P56379	1.051428576	0.962032282	0.933552158	0.890646657	1.02592186	1.068568689	1.033149008	0.80812277	1.058692836	1.335062193	0.952618188	1.061422036
P08905	1.147308224	1.078381895	1.055853428	0.889838135	0.883603103	0.830283437	1.098996078	1.058477936	1.050246261	0.922576243	0.986001454	0.964654068
Q9DB15	0.945427608	0.987842127	0.972461104	0.98145641	0.937280634	1.003268379	1.042593563	0.997181883	1.097962922	0.962184755	0.977354994	1.081956086
P70399	0.981445605	0.979570456	1.022802693	0.976014792	0.994225952	0.947609874	0.953684965	1.004948523	0.942080312	1.120183996	1.103872937	1.033398998
O35393	1.027470054	1.092215588	1.06891057	1.124883066	1.114989615	1.130192252	0.827271917	0.960824125	0.897213052	0.876451446	0.963366499	0.950249644
Q8BLN5	1.009259691	1.045520267	1.070019425	0.949367835	1.077579545	0.984481048	0.983805274	1.011976827	0.978698527	1.057124052	0.973341787	0.906936513
Q9WUU7	1.147297686	1.02337896	1.076739731	0.982102801	1.058496201	1.094060684	1.017434842	0.915240299	0.994517283	0.925734857	0.880881625	0.919605566
Q8BI72	1.057263575	0.915624913	0.980642129	0.942208026	1.095689356	1.038041141	0.998550312	0.937919328	0.986360299	1.275939115	0.963120688	0.96765758
Q80YA7	1.012903118	0.970405892	0.980710908	1.003198112	0.980937158	1.00907815	0.967862991	0.932428499	1.03976001	1.047047001	1.01054002	1.094889306

Q99M01	1.035530889	0.999622786	1.013216622	0.999129103	1.032566221	1.024818544	0.92486462	0.978106349	0.942312467	1.076607608	1.028034098	1.020142612
Q06180	0.95186193	1.024043082	0.95314517	1.011953186	0.960737791	0.98399449	1.051457783	1.009713669	1.013946964	0.970428918	1.017194244	1.050335461
Q00560	1.031810008	1.032506753	0.978781388	0.931145376	0.95818804	1.006358725	1.069982092	1.030387751	1.001222627	1.078137212	0.963830352	0.968114839
Q99MS8	0.979436534	1.130526695	0.974662023	0.950990561	1.060700241	0.94489105	0.917318415	1.025052892	0.920189838	1.130857551	1.055945074	0.99393686
O89017	1.093445036	1.006012055	1.017661171	0.951666191	0.966113015	0.992640609	1.033041069	1.001216086	0.977548975	1.003133591	1.003503648	0.982424955
Q8BHJ5	0.916347405	1.111105161	0.987420192	1.067676753	1.128021284	0.916843043	0.941737428	1.006086488	0.930260247	0.990065465	1.014948962	1.025444001
Q99LM2	0.997217662	1.137552856	1.018857186	1.007251308	0.958073301	0.938880195	0.99263441	1.005094827	0.999384099	1.048231087	0.986564577	0.952021958
Q9R0L7	0.98896332	0.958792294	0.958076496	0.967766957	0.960219726	1.045923935	1.088433537	1.020044699	0.933984414	1.259263489	0.994017836	0.951852968
Q9WTN0	0.993095885	0.999078437	1.004795485	1.04982217	0.993215375	1.019196118	0.947810327	0.998927553	0.985825831	0.940451477	1.009829369	1.068887224
Q9CQW0	0.927372657	1.120555274	0.991422652	1.048983586	0.9540337	0.864891921	0.914686184	0.992014115	1.073065245	0.804539212	1.121802598	1.100194779
A2AAE1	0.988136661	1.039478486	0.981335069	0.939559288	0.98645381	0.925417173	1.047523905	1.025691513	1.008751924	1.021609515	1.036870516	1.009766998
O89116	0.979142797	1.031016422	0.973228465	0.94569513	1.033369359	1.019703715	0.961925806	1.012832582	1.026786839	1.153376937	1.01811529	0.921855933
Q9DBS1	1.133932638	1.066117941	1.026311814	0.931537965	1.051169187	1.010710462	0.999988015	0.972704519	1.001034908	0.988767105	0.946134916	0.912800683
Q91W43	0.946828148	0.969624511	0.957868579	1.059172369	0.944500316	1.049173186	0.975808763	0.979027604	1.022206496	1.140974306	1.019455872	1.014683932
Q9CQQ8	0.992710848	0.980809073	1.027017845	1.07128895	0.905152619	1.002306833	1.00242877	0.977818194	1.086868751	0.807271056	0.978383209	1.106670715
Q9DBX6	1.023983611	0.958512254	0.976738799	1.036685177	0.92912612	0.930905252	1.015904939	1.016518919	1.066768933	0.920280271	1.039469058	1.059762831
Q9D1G3	1.051792744	0.99788225	0.959418521	0.984990496	1.100405752	0.942220321	0.987711604	0.965591332	0.928523666	1.175907782	1.07178191	0.941292679
Q8N7N5	1.005573376	0.978232924	1.03541859	0.967102699	0.974069948	0.948607154	1.082228952	1.005599254	1.081929078	0.94829348	0.967316482	0.984176193
Q9JJV5	0.85217076	1.036567006	0.898541491	0.976463444	1.045625945	0.949055592	1.027758742	0.965985469	0.955530653	1.026807895	1.185464165	1.078277553
Q3U2A8	1.001917372	0.97691979	0.99896207	0.954576718	0.979082956	1.027330072	1.009728348	0.959781337	1.057216351	1.042129176	0.95651211	1.077483165
Q3U487	0.96805924	0.964274981	0.987471719	1.044474577	1.004771108	0.968554217	1.003479691	0.987936285	0.986707789	1.036377184	1.037710398	1.04578172
Q9D5R2	0.945560396	0.949429264	1.002488422	1.022993458	1.05341561	0.937727207	0.938852873	0.937312547	1.027514721	0.997481331	1.107239188	1.087766115

Q6PCN3	0.954792669	1.017978442	1.031430398	0.994082085	1.043209112	0.868266211	0.976615875	1.131321714	0.954720449	1.112480646	1.040451835	0.923085876
Q9QY36	1.044206706	1.02286565	0.954492127	0.972736009	1.052624924	0.990368738	0.991683819	0.970942357	1.004835305	1.128174685	0.991376011	0.964882221
Q3V3V9	1.011671314	1.023875659	1.031687837	0.909420246	0.885949547	0.916004133	1.115243135	1.06472095	1.131841606	0.989120187	0.942336886	0.952823682
Q8VCN9	0.970907337	0.991300618	0.946306045	1.000655412	1.039645175	0.973914621	1.027728859	1.091351103	1.026746958	1.066465385	0.952785387	0.958589328
Q99P58	0.991372475	0.96768708	1.107756949	0.996925908	0.955398195	0.991275941	1.015508396	1.052045881	0.976562127	1.029919151	0.991438276	0.944409209
Q99MD9	1.005989926	0.937596253	0.947723414	0.95903407	0.981768196	1.029762987	1.076707357	1.005573341	1.010053266	1.133570927	0.970257128	1.017885453
Q6NTA4	0.98060904	0.996927396	1.032582825	1.002253306	1.010101227	1.00202294	0.974144981	0.959194315	0.917594622	1.107110196	1.08117676	1.003009195
Q8BYY4	1.095831611	1.038418628	1.0537242	0.949013376	0.949410677	0.937751952	1.088411826	1.023444078	1.020068807	0.959001582	0.922314173	0.968870962
Q9DBR0	0.988920451	0.97209382	1.054983284	0.95341582	1.22442627	1.056983819	0.971869238	0.933739627	0.882561365	1.156595681	1.018340349	0.895643645
Q62413	0.965523728	1.106614465	1.017412297	0.955114781	0.9274245	0.8907248	1.048074628	1.052236347	1.01266592	1.006613783	1.037714218	0.972413726
Q3UMT1	0.981518317	1.057990675	1.015299817	0.979358398	1.022631093	1.018599728	0.931707445	1.019676443	0.96718292	1.041055942	1.010843013	1.000588775
Q9Z2C9	0.945894528	0.939817078	0.98210352	0.998344254	0.975966956	0.942870072	1.027537561	0.998569372	1.053676149	1.037267279	1.0481682	1.06192115
Q6P5D8	0.938398005	0.984045935	1.009065338	1.037918151	0.998347264	0.937521449	1.025705663	1.013467158	0.979357819	1.009650578	1.05283644	1.024356996
P41242	0.905798089	1.19262282	0.970272111	1.010195071	1.051960808	0.983136416	0.994977518	0.976501608	0.931999439	1.051686544	1.034879391	0.941302926
Q80TE4	0.946152949	0.906402206	0.967866148	0.875399113	0.894916406	0.965328794	1.032691046	1.134006499	1.008364161	1.168290677	1.028104142	1.127580888
Q9R0P6	1.028973719	0.988681488	1.029807451	0.969417339	0.999922911	1.007601038	1.022267102	1.037471566	1.02716749	0.937731536	0.970378031	0.97270819
Q9CZG9	0.999173283	0.981315553	0.941323067	0.97599548	0.938037855	1.047080893	1.055216271	1.029105024	0.995129977	1.103027086	1.014495708	0.976184344
Q9CZN4	0.916324826	0.996176215	0.93970043	1.032744487	0.925323031	0.992217566	1.040424022	1.009169413	1.049902038	1.002707146	1.00770753	1.095212068
P23298	0.894399055	1.032910778	0.995018128	1.050536356	1.046005122	0.833415313	0.853311452	1.077573067	1.014025719	0.874361179	1.134520481	1.142184006
P97290	1.155711777	1.254080707	1.197766515	0.781487362	0.738958321	0.687381063	1.338689724	1.147044863	1.369309217	0.721863896	0.728305479	0.691992061
Q8JZM7	0.891759049	0.988808989	0.986043327	1.016077304	1.157340648	0.987478212	0.964898575	1.049968453	0.872563102	1.128895275	1.049281751	0.989402069
Q80U35	1.012449739	0.980998369	1.016842385	1.049779273	0.988310489	0.973882079	0.979602733	1.045412919	0.995651754	0.947151669	1.01494468	0.996156124

Q9JJZ4	0.993225028	0.974284054	1.012271478	0.970386514	1.007421803	0.995629225	0.997388765	1.063780922	0.987275824	1.069125708	1.001506283	0.971367479
Q924L1	0.943055135	1.027898778	0.976905715	1.080503277	1.024685285	0.977236106	0.963663003	0.986251284	0.992869408	0.965566668	1.039910413	1.033229159
Q9D0Q7	0.992360825	1.001456611	1.023781054	1.029095253	1.073900557	1.029726534	0.964491701	0.990127802	0.908472079	1.054368353	1.001239524	0.999593675
Q01815	0.996235809	1.012314158	1.052161201	0.976365763	0.927737864	0.923582409	0.937047179	1.017781235	1.021398529	0.958357817	1.052578154	1.114989397
Q8BG67	1.022395315	1.009836441	1.017790029	1.004509229	1.014413975	0.960998834	0.943169481	0.994079599	0.955731843	0.937610057	1.051995064	1.094118935
Q02257	1.591159221	0.92967975	1.041335032	1.591173753	0.7421078	0.990302527	0.772374895	0.80497039	0.802502259	1.427458136	0.872327928	0.829375382
P24457	1.096410015	1.096249689	1.077591203	0.911961213	0.979985211	0.913646801	1.059639206	1.054894894	1.122903109	0.921337737	0.902788582	0.833234028
Q8BGW1	1.042994643	0.991016654	1.038531759	1.029387975	1.053178391	0.93855759	0.940315518	0.980921423	0.921477621	1.159918859	1.047092628	0.961214401
Q3U319	1.021842896	1.083634698	0.989500571	0.978530862	1.04072817	0.992039882	0.971185513	0.983051244	0.94175625	1.015893587	1.035527199	0.987727553
Q9WTX8	1.008174464	1.025538543	1.008449981	1.011718129	1.028672679	0.936208583	0.969171488	1.026023501	1.000960477	0.963895673	1.04787771	0.974645934
Q9CQ80	1.023318736	1.01783212	1.028452476	1.007636297	1.004290977	1.021257637	1.02083116	0.977563631	0.971535688	1.005541803	0.974548795	0.983898503
Q2TA57	0.918132446	0.98223728	0.900817031	0.973521529	0.968501529	0.96921425	1.100587271	0.99843074	1.01468371	1.150088946	1.047552503	1.034420677
Q6NVG1	0.963766551	0.977096545	0.948813138	1.024554915	1.030034066	1.010445723	1.001875597	0.965589457	0.999390745	0.999595367	1.052752666	1.047367708
Q61161	0.994768246	1.046168546	1.002101728	1.011963304	0.982384027	0.99026811	1.027211543	0.961176052	1.008881512	0.996922176	1.00026326	0.998957453
Q62422	1.102076025	1.008897084	1.005411644	0.933049715	1.015050505	0.976968456	1.049794816	0.99789185	1.036985096	1.012443824	0.941117601	0.955341621
Q8R310	0.950617299	1.002161147	0.995795657	1.015108345	1.041700643	1.060141428	0.945910416	0.997311854	0.920795939	1.054243105	1.042660339	1.029933235
Q3UQ84	0.976324377	0.967511785	0.977963757	0.98124618	0.954455554	0.979721125	1.030537058	1.017701167	1.037028196	1.0323541	1.007603166	1.05919482
Q80SY4	1.093822799	1.085929655	0.943873691	0.985328052	1.059285424	1.07437335	0.973845229	1.015686877	1.024799565	0.999207872	0.896807409	0.906109775
Q2WF71	0.98531904	0.966477326	0.965476403	0.982878848	1.045227116	1.006037491	0.983677793	1.047150999	1.006767309	0.946195999	1.032312331	1.027625926
Q8C181	1.058290684	1.025240028	1.077778084	1.114343004	0.995039653	0.918797553	0.866875817	0.98234922	1.030811761	0.688072212	1.046111274	1.106043166
O88983	0.938907668	0.98312785	0.935206049	1.025078699	1.007224577	1.072884796	1.036754459	0.998431975	1.056952728	1.032490879	0.954958887	0.992925911
O89001	1.080131312	1.076188255	1.023342426	1.081712036	0.970248196	0.945614336	0.861066607	1.02446158	0.91781439	0.983434469	1.003797219	1.09043343

Q570Y9	1.023521536	1.009712512	0.992227865	0.993378807	1.039270889	0.918637793	1.03444121	1.090922118	0.980407713	0.963250889	1.035469263	0.912729221
Q3U1N2	1.076277443	1.021193677	1.029469563	0.856563553	1.118186015	0.931845745	0.96943428	0.988862187	0.855306802	1.057568306	1.134303013	1.00930329
Q9CR86	1.049992233	0.989992509	1.036484673	1.032746364	1.023835882	1.062240955	0.974997652	1.005887044	0.996244425	0.941643651	0.935551761	0.973127737
O88502	0.913953185	0.980689824	1.005016593	1.066047053	1.082052803	0.998813047	0.939522066	1.02205634	0.961600599	0.991483828	1.03710663	1.024294479
Q3U2I3	0.985847898	1.080155415	0.924921933	0.906040499	0.884048708	0.957316145	1.072783166	0.987625651	0.952689759	0.993561811	1.109594691	1.142684717
P17439	1.043699253	1.054666202	1.003546297	0.978072007	0.976708239	0.947489266	0.978356372	1.032965675	1.076120296	0.927778239	0.974996806	0.993837235
Q8K2H2	1.015823725	1.061868786	0.982161267	0.98818569	1.061600014	1.045208812	0.919645658	0.948163281	1.010184497	1.065944573	0.992846373	0.978546801
Q5SWP3	0.907685044	0.938431872	0.920400388	0.998647761	1.098425935	0.909644671	0.989671694	0.96907481	1.017520358	1.058697743	1.155367875	1.054011511
Q9JHJ0	1.12809375	1.078975625	1.017040428	0.97708301	0.944531981	0.988280988	1.014864959	0.983845072	0.989708167	0.914192768	0.999841873	0.963201181
P17047	0.993144219	1.163175051	1.006778712	0.940912191	0.973500966	0.924217826	0.993266104	1.024512924	0.998143946	0.939124286	1.045954181	0.978109745
Q99JX4	1.030542101	0.983374635	1.000653723	0.936618012	1.017391389	0.956492294	1.007051625	0.978870416	1.027443321	1.085371798	1.0182253	1.008452092
Q8BHE8	1.106953524	1.084361451	0.855272372	0.892272066	1.114536932	1.10407337	0.927089604	0.875283682	1.134118503	1.291199868	0.88048581	0.921032499
Q60751	1.019657925	0.999048799	1.009163166	0.991356085	1.127120359	1.02494891	0.995591291	0.959002004	0.906367075	1.083397284	1.010605702	0.954382224
Q8C996	1.0408223	1.0065834	1.005169008	1.015187663	0.936057137	0.946008011	1.022073386	1.021619108	1.037658539	0.952947047	1.002821665	1.009064039
Q6EDY6	1.040419255	1.017008154	1.043235616	0.935636302	1.085797641	0.981551491	0.975127723	1.018880936	1.026117331	0.938593378	1.005024762	0.924676191
L0N7N1	1.029690505	0.999452366	0.997927922	1.058933795	0.984998075	0.996219045	0.991459836	1.017789528	0.985311432	0.967888619	0.988797062	1.004346758
A2A7S8	0.97335675	0.944058976	0.960809248	0.979051116	0.991278638	1.010756403	1.037219067	1.003524253	1.026337628	1.032632557	1.029236462	1.033739388
Q63739	0.975105657	0.96282357	1.00143952	0.998946434	0.990097343	1.018805502	1.018018963	1.019735694	1.043918403	0.918624386	0.998449416	1.031872464
Q3UQ44	0.927572685	0.92461683	0.959932848	1.01164357	0.990346715	0.895761406	0.973980035	1.040630213	1.016228715	1.050271768	1.151863489	1.062807405
Q91WA3	1.205092386	1.102899855	0.838171076	0.834336024	1.198581799	1.169367231	0.822868359	0.854892078	1.145654159	1.432385691	0.832071867	0.840660516
Q924T2	0.953643869	0.971724227	0.986052534	0.984835553	0.961968736	0.989803852	1.095968594	1.045129827	1.03013178	1.170783486	0.929955649	0.959076183
Q9WV96	1.001296985	1.045167867	1.055952045	0.954683545	1.040822608	0.989784116	0.986828821	1.053552762	1.004373435	0.996340167	0.950507683	0.940796853

Q8C170	1.041845109	1.072627473	0.924351946	0.95296565	1.057861945	0.975600953	0.908297904	0.989726874	1.059071613	1.159585289	1.015238025	0.939373325
Q80YD1	0.964683677	0.993834193	1.034423292	0.869130576	0.871482823	0.862396055	1.145144392	1.153595313	1.15614311	0.893947196	1.010367385	0.940892534
Q9CZR3	0.944010073	1.006305573	0.959025098	1.045123838	0.998408563	0.92567912	0.93593581	1.057389057	1.010460634	0.960705579	1.108454523	1.035486651
Q3U0M1	1.005241843	0.980498186	0.971807187	0.99513583	0.992104047	1.041240074	1.025360732	0.95624901	0.994863291	1.0534311	0.998588726	1.037750763
Q91W34	1.058523458	1.057892725	1.024406276	0.985088674	1.091198688	1.037946464	0.993985281	0.966242635	0.980593219	0.868008469	0.977447671	0.947520111
Q91WG5	1.004493112	1.165009811	0.945933799	0.980535324	0.983863387	0.958738979	0.947151835	0.933723147	0.917406706	1.096395803	1.105901922	1.032820448
Q8K0C4	1.04479857	0.984907374	1.017545153	0.943978017	1.030845275	0.965969973	1.06104768	1.039617157	1.000036869	1.094827015	0.946209257	0.931188529
Q9DCV4	1.084407689	1.053982134	1.004575531	1.007065609	0.94590101	0.986484115	1.01483815	1.01440593	1.039660474	0.924901645	0.943671243	0.983116569
Q9WUB4	1.049747683	1.009928671	0.992727168	0.97873225	1.024554202	1.011811596	1.000935391	0.974112648	0.988741378	1.168169976	0.970366427	0.937040329
P50096	0.889677453	0.920516291	0.926459175	1.09649848	1.104978512	1.068012205	0.900950379	0.908020968	0.944125358	0.952868278	1.082390732	1.235578767
Q8K2Q7	0.992848957	0.966334431	0.984724163	1.00916522	0.973188746	1.015967632	1.017521318	1.026600457	1.023371602	1.015023882	1.028916761	0.961711392
Q8K2Q9	1.049394772	1.10291517	1.059417334	0.928941465	0.96643045	0.962321417	1.012182343	1.071431383	1.06593153	0.929927457	0.937211982	0.893958946
Q6PHS6	0.954808692	0.942925748	0.937112078	0.942974462	0.946962578	0.998816082	1.055421792	1.005801807	0.9926589	1.179668366	1.04455949	1.076593615
Q9DBN4	0.917179798	1.035127383	0.964832838	0.9866352	0.96414946	0.980668161	1.03611775	0.995509228	1.040616927	1.027597332	1.027450852	1.035189145
Q80X85	0.981565874	1.042185747	1.023580098	1.002540459	0.906106094	0.972770251	1.047529816	1.05955999	1.046701905	1.003293765	0.95760977	0.962646799
P70268	1.033195564	1.035360968	0.980101179	1.06822655	0.985110175	0.946461987	0.976936251	1.110035278	0.976911819	0.934775237	1.003523833	0.951153664
Q8K097	1.106087343	1.077644168	1.0262746	1.062562747	0.872872498	0.956504612	1.062789722	0.959273684	1.008877565	0.918432844	0.938093668	1.018928
O35166	1.03630448	1.029025285	1.087812989	0.999314017	0.956274924	0.934717888	1.017169512	1.016453044	1.048538729	0.87078567	0.967860896	0.995509606
Q99L27	1.004604034	0.969092636	0.969690979	0.967299897	0.994892844	1.001025057	1.01635935	1.003656067	1.021528313	1.045817961	1.020006398	1.020124573
Q99KK1	1.024446697	0.941438168	1.11190256	1.009059063	0.948202234	1.043926788	1.015036102	0.878849613	1.015316865	1.113509681	0.963867831	1.010178058
Q9DBH5	1.022929049	0.94797037	0.988616261	1.046338778	0.931601377	1.05483209	0.937799779	0.979639508	0.96605159	1.005819119	1.02287548	1.141280964
Q9JJG6	1.058652546	1.02254669	1.02802029	0.971779749	1.011597597	1.050423491	1.052459905	1.049770611	1.037664849	0.826861231	0.919219108	0.926208279

O89020	1.318845975	1.380379835	1.383293027	0.749086056	0.632821241	0.593091012	1.244322227	1.254470347	1.272211108	0.711507935	0.665830827	0.624624932
Q8C0I1	1.13377409	1.025615432	1.045599112	1.101650066	1.167844771	1.126363248	0.902885239	0.90505973	0.868940489	0.935272063	0.946168691	0.917157616
O55060	1.024374052	1.085464738	0.982046821	0.958171114	1.036819939	0.986969935	1.034348992	0.973210637	0.951862661	1.03967332	1.015154095	0.957401007
Q922H1	1.035946608	1.009221281	0.980645293	0.927982118	0.961248239	1.00665341	1.063456219	1.015434783	1.027520571	1.030648314	0.996915086	0.966654509
Q3U1V6	0.940927167	1.01324698	1.016484961	0.978571355	1.030158227	0.921189297	0.980165768	1.028692668	0.954880089	1.067854894	1.082789411	1.017254295
Q9CQT1	0.991658539	1.000810005	1.058859582	1.022287233	1.069114004	1.0789325	0.951923692	0.973688368	0.926280848	1.022520581	0.986288764	0.972344503
Q6A0D4	1.138360429	1.050008072	1.052094315	1.086979572	1.135529267	1.097663011	0.850661014	0.915484498	0.903381668	1.070814837	0.916270982	0.916501739
Q02013	1.044204154	1.015756355	1.076750837	0.93846813	0.922175464	0.850997134	1.025798827	1.069935614	1.101074557	0.999067071	1.024055276	0.909672947
P41234	1.043563045	1.037609435	1.000767423	1.03128273	1.082861956	1.018150208	0.931474498	0.913657563	0.962838853	1.037146377	1.009495702	1.001560023
P98203	1.041429847	1.006605277	0.9903501	1.002271081	0.972364581	1.037867833	0.99439986	0.989094068	1.003227311	1.092885382	0.940485539	1.009817971
Q8BP48	0.906905242	1.218614248	0.986923255	1.022889527	0.866159534	0.934673143	0.924628613	1.044535039	1.040519288	0.865049314	1.115126395	1.004464049
Q2TPA8	1.077767486	0.970772413	1.027877892	1.094057267	1.064431141	0.960206213	0.968627703	1.001283097	0.956560736	0.989365836	0.963267291	0.978560799
Q9R0X5	1.013998566	0.780685576	0.885186317	1.014334797	0.745921876	1.100147657	1.12549632	1.080920147	1.313781214	0.777414138	0.877971139	1.178220851
Q80ZJ6	0.97419714	0.953790429	0.96830019	0.987772083	0.979897929	0.962307497	0.977166987	1.016937703	0.937062938	1.12395807	1.130298285	1.046525701
Q6DG52	0.970327069	1.003155176	1.025607928	0.977884142	1.028010964	0.955911095	1.011855647	1.063668887	0.943658017	1.120060982	1.045434279	0.9105258
Q9ESN4	0.965023404	0.970075714	0.97092648	1.041810783	1.057163677	1.013697488	0.990654453	0.978675977	1.034728886	0.946148324	1.028935677	0.999987523
P70677	0.965007526	0.960331317	1.030555591	0.990959297	0.948284673	0.868391702	1.040606594	1.071545023	1.034720838	1.05302234	1.034378961	1.011955849
P61600	1.025473109	0.957728597	0.991335243	1.020606122	0.916815776	0.979469517	1.014716815	1.033011756	1.092534753	0.855768197	0.98486797	1.080152491
Q8R409	1.003852889	0.973030963	0.981077959	1.018081741	1.143650787	1.026880542	0.954168238	0.964702777	0.891748807	1.110817335	1.031585838	0.999222436
O35598	1.102533164	1.02155388	1.067923503	1.030346129	0.99422339	1.005720039	1.000633405	0.999478589	1.023148614	0.914586441	0.905435913	0.945690913
Q9D883	0.943014725	0.939996589	1.014370597	1.112140844	1.053227029	0.937931089	0.949308514	0.963789445	1.030863832	0.919429095	1.07395928	1.044704047
Q64314	0.948947589	0.899794651	0.966633195	1.047976326	0.956339022	0.884784914	0.968951741	1.006532573	1.11448178	0.852202777	1.157716105	1.111493176

P61804	1.211268724	1.157326228	1.124049563	0.785476276	0.92064036	0.933543163	1.062000034	1.059733615	1.175759994	1.07881276	0.779837864	0.757308127
Q8CJ61	0.924551767	0.994795319	1.09009026	0.973727991	1.019217262	1.006258171	1.02127659	1.060655222	0.900269448	0.983489317	0.975081187	1.06492666
Q8BH51	1.028687923	1.00973262	1.022959804	1.07955539	0.998236698	0.982263727	0.992728279	1.001224383	0.982357884	0.92341507	1.013297396	0.965494258
Q9D3B1	1.232007011	1.095053675	0.889232134	0.86395985	1.109333237	1.073955499	0.938654049	0.848251064	1.088310402	1.262396961	0.911456137	0.872335682
Q9WTU3	1.089911473	0.958524419	0.881739588	0.925789147	0.990049942	1.06013503	1.061383917	0.994773255	1.016676597	1.206538263	0.959602796	0.977786451
Q6NZN0	0.997167761	1.039295547	1.040524411	1.037504674	1.037291107	1.007260455	0.986267113	1.017368197	0.993670387	0.894859592	0.990280121	0.941889758
Q9QZB0	0.968618084	0.953572701	0.925497939	0.970652575	1.027220282	1.047983873	1.101097208	1.023315169	0.983323912	1.286288562	0.918485716	0.940844449
Q99M31	0.960277682	0.993032242	0.992970983	0.99568141	1.005668697	0.971350373	1.017401985	1.062335875	0.94068812	1.088973255	1.01574205	1.010731955
Q6P5G6	1.064814386	1.030165821	1.048493733	0.9040696	0.947072966	0.956407097	1.028436092	1.026821148	1.101840866	1.003677607	0.951110225	0.941535747
Q8BH24	0.971373976	0.973901424	1.018415033	0.960421211	1.01546207	0.899565482	1.037251577	1.051224294	1.038575878	0.944870853	1.04398297	1.013524048
Q8CGZ0	1.045117719	1.060431868	0.986124561	0.993167711	0.907798601	0.985343371	1.050674515	1.039549833	1.033355254	0.966737304	0.948048662	0.992145123
Q99NF3	0.963900347	1.000016744	0.907885703	0.981703527	1.108652821	1.03920292	1.005550807	1.068008738	0.91122196	1.188189205	1.032933503	0.899830663
Q8HW98	0.93275042	0.976929205	1.043002124	0.997815263	0.974975843	0.901598293	0.98758148	1.064525682	1.038002986	1.175078331	0.993285776	0.984634917
Q8BPB5	1.057595237	1.029007443	1.031858563	0.99980674	0.92791695	0.975100807	0.976417812	1.076323588	1.03879334	0.901359179	0.974065491	0.990952251
O08576	0.987434763	0.953999851	0.998394124	0.976713604	0.933538352	0.963597376	1.0065481	1.010310028	0.991972103	1.005774657	1.111579138	1.058738034
Q8K3E5	1.100791692	1.000522529	1.491701665	0.967648616	0.976828107	1.049193548	0.880889286	1.077914484	0.885277008	0.877868667	0.88947645	0.781451292
Q9CQM5	0.875637815	1.034492987	0.954968619	1.075389671	0.801940071	0.886808749	0.909984379	1.087257231	1.151342841	0.990466746	1.097017491	1.100785791
Q09200	0.958278333	0.950845167	0.971429588	0.920536356	0.927177497	0.92073823	1.080224172	1.046495555	1.067512155	1.087458737	1.048904925	1.033622254
Q8K2Q0	0.996469526	1.015691841	1.024891752	0.968201677	0.993962982	1.016346251	1.013471044	0.995210275	1.01472895	1.024824501	0.985345811	0.978816325
P46414	0.892097408	1.108687383	1.056091249	1.016299601	0.862023047	0.96440321	1.092574064	1.021358654	1.01773268	0.850771779	1.021714462	1.017804479
Q8JZS6	1.127583148	1.14848417	1.029913475	0.974174471	1.121674718	0.991238178	0.983832489	0.976907273	0.894733039	1.172422096	0.883918725	0.845502048
O88851	0.985765524	0.965899588	1.005049641	1.012775468	1.139990583	0.979308125	0.947626037	0.99149695	0.930709717	1.0209989	1.07850059	0.980302599

Q9D8V7	0.982255508	0.989156766	0.988125081	0.884000182	1.022978669	1.044504822	0.98438656	1.071310306	1.243611719	1.017765888	0.944889183	0.811180505
Q91V36	0.985546603	0.952272261	0.98515685	0.963888749	1.067260921	0.984329675	0.987532319	1.025743348	0.968880289	1.05629044	1.070553263	0.988407373
Q8BQM8	1.008579429	1.017758943	0.973713995	0.988298868	0.972402538	1.060193422	1.038941591	1.005542448	0.960763275	1.04713625	0.991378515	0.984064727
Q9JIK9	1.107755179	1.107723113	0.97450493	0.998937711	1.035197869	1.025919123	0.951944237	1.000286405	0.990544508	0.976102901	0.936899272	0.944468494
Q9DBX2	1.060342071	0.940216241	1.013916592	0.982411821	0.998104704	1.02865462	0.974811763	0.948670531	0.978955224	1.023374653	1.029346819	1.066478901
Q791V5	0.99035992	0.920166922	0.897144133	0.982052207	0.955270011	1.018629947	1.040270101	0.957799942	1.050335616	1.07362857	1.017166987	1.14807375
Q7TN79	1.097908976	1.006292355	0.930800433	0.890584947	0.948478607	0.946789764	1.213279265	1.086756989	0.992500927	1.030357957	0.923493416	0.959488625
Q9QXT0	0.951051923	1.004155674	1.059487397	0.945894438	0.889227365	0.961223783	1.094567649	1.093328822	1.051256866	1.025824849	0.97497621	0.938501723
O54916	1.101062077	1.075580218	1.065726977	0.952984108	0.991029917	1.010581719	1.025084511	1.018961232	1.008047212	0.905966541	0.928407455	0.911125322
Q6PFR5	0.966080672	0.977819436	0.985803915	1.080686385	1.007493439	1.049659922	0.997490347	0.986234832	0.950890188	0.995469122	1.001734968	1.037772635
Q9D1M4	1.005557357	1.031684565	1.031008995	1.000935752	1.023128936	1.013045282	0.985361218	1.028575107	1.005952032	0.934801492	0.962326707	0.979624696
Q9CWD8	0.959929403	1.000836677	0.876358656	1.012361328	0.99863682	1.316100935	1.066925145	0.937387133	1.003845428	1.042743966	0.89274203	0.965139702
Q3TCN2	1.102433593	1.032071733	1.011269767	1.045038753	1.100647758	1.053771384	0.938806139	0.966620223	0.932275189	1.028163626	0.952827857	0.919889221
Q91WG7	0.963005137	1.098279974	1.082219611	1.016520409	0.990262459	1.058161195	0.934505289	0.936558416	0.91508096	0.89553129	1.068093254	1.028055075
Q99LB7	1.10558933	1.011864851	0.996807435	1.022325594	0.993430314	0.98125221	0.951671668	1.055140425	0.990711343	1.02539931	0.970355286	0.950673641
P35441	1.343604067	1.161149082	1.299206733	0.836394692	0.904450394	0.868334985	1.035019365	1.106265136	1.010627532	0.936470671	0.780438674	0.732822231
Q99J10	0.97241582	1.034876268	1.035811589	1.039771507	1.05869501	0.985687666	0.899250323	0.982103547	0.946019793	0.958631311	1.070892628	1.030456811
Q9CZN8	0.997080856	1.0709632	1.029302533	0.953975059	1.024786349	0.937215453	1.038653899	1.006767109	0.980257934	1.134467424	0.933073467	0.976773947
Q8BJ05	0.938399826	0.932197492	0.95264174	0.947952408	1.566889549	0.905381694	0.931267341	0.923132765	0.974370832	0.9499483	1.127720394	0.857282299
O88939	0.956785634	1.002829922	0.906056103	0.953696217	0.996473056	0.956145699	1.078173291	1.033565281	0.998244836	1.122209355	1.01024509	1.046108302
Q9JKW0	1.022147399	1.062542716	1.061776704	1.039103836	0.992111522	0.989521298	0.925309077	0.977755644	1.021475409	0.760075098	1.005806089	1.076773918
Q8BXR1	1.083201657	1.046933018	1.092208302	0.983325846	1.001906559	0.951412529	0.981865959	0.963339607	0.978963761	0.920742497	1.001037298	0.994927114

Q3THK3	0.837304006	1.105600594	1.111215505	0.988108319	1.015083826	0.935969534	1.040914379	1.067123464	0.90455953	1.192878304	0.816183717	1.101530285
Q8BG89	1.731269278	1.268000732	0.63195275	0.604253987	1.413076818	1.375685044	0.645018478	0.711994107	1.270011822	1.825670544	0.578601671	0.513733192
Q8R4F1	1.135484636	1.041531289	0.909538862	0.934900711	1.055940405	0.98562309	0.882396941	0.929084696	1.198500473	1.215220936	0.929021143	0.915046149
Q6PGB6	1.003852368	0.964450446	0.966906798	0.991070353	0.98435428	1.054429658	1.035634222	1.044795543	1.04819812	0.946429251	0.969234091	0.985922347
Q3UHE1	0.990244349	1.010649334	1.006030712	0.968121329	1.016160362	1.003530034	0.991703475	0.989026112	0.976216578	1.055651984	1.039277702	0.994514455
Q99JW4	1.036996574	1.088074715	1.000694559	0.976982359	0.978784816	0.949585187	0.969337383	1.014272215	1.036181118	1.02464915	0.997521057	0.957846966
Q9CQF3	0.931923287	0.943774943	0.956562441	1.011793628	1.064482017	1.028696302	1.051814732	1.04498945	0.960110343	0.978285942	1.032661328	1.000036593
Q6A028	1.238708367	1.214939141	0.937926485	0.75405253	1.222505328	0.992391621	0.888910565	0.810369875	1.061604433	1.404234428	0.911945998	0.808036782
Q9JI19	1.016350167	1.032685998	0.981450876	0.943484762	1.026412173	0.979280142	1.020933268	1.026390675	1.009777746	1.053173838	0.982193683	0.970232405
Q9CQN7	1.055021282	1.119678892	1.009444047	0.890260125	1.004204843	1.090122158	1.013542437	1.056186981	1.12811411	1.035644525	0.782515561	0.865973507
Q5SUF2	0.966531083	0.918084697	0.992960592	1.027750413	1.04592755	1.053898062	0.98719159	0.965463091	1.00213472	1.046223403	1.02166571	1.016566252
Q9R0U0	1.049251043	0.956837307	0.928724806	0.899946389	0.981719292	0.960520038	1.062738911	1.048193331	0.878046025	1.312597698	1.099223692	0.964163871
Q8R092	0.931054682	1.386203694	1.013235373	0.990177322	1.044470733	0.898638494	0.952173088	0.950582102	0.926168706	1.076740712	1.073999348	0.804967301
Q99J31	1.005076838	1.010442451	0.929878993	0.939651774	0.992903728	1.003191735	0.9990564	1.017056049	0.998714125	1.174429735	0.996689877	1.03060995
Q8C460	0.869722403	0.973822609	0.968113593	1.082605339	1.056697437	1.03965302	0.931698228	1.03336186	0.964304343	0.836678034	1.107961906	1.081159353
P56716	1.003113342	1.257239167	1.041539517	0.990547125	1.101153233	1.0453838	0.94670983	0.948620786	0.839848318	0.947726931	1.029474077	0.881395776
Q9CQY1	1.008984859	0.978118419	0.989194132	0.977154871	1.020885933	1.043247343	1.01195583	1.023860887	0.976576813	0.977763341	1.026453424	0.977248161
Q6P1D5	1.042749299	1.037046449	0.975341321	0.922890838	1.042792744	0.941828452	1.08075953	0.980163108	0.982493121	1.205379752	0.970189186	0.9276049
Q8VCS3	0.990583075	1.080807032	1.012390759	0.91649009	0.986837153	0.94754134	1.011293119	0.994860001	1.0117997	1.112389219	1.012413754	0.976630929
Q3TRR0	1.207236001	1.314208386	1.172535266	1.044664527	0.996270395	0.992505458	0.9004909	0.827420745	0.875918861	0.933652982	0.893504529	0.918373557
Q6NXJ0	0.815617096	1.070871298	0.930982273	1.056136038	0.939054496	0.889389623	0.948906217	1.140494651	0.999855148	0.901251113	1.163037584	1.075610823
Q80Z10	0.919537732	0.951784319	0.970279294	0.866686992	0.944468339	0.911819444	1.113152314	1.025577502	1.065488433	1.21224159	1.02566431	1.052734677

Q6NSW3	0.978015872	0.978688599	0.977898857	0.961585362	0.987656535	0.966707382	1.051140638	1.032695256	0.971751192	1.095199246	1.015930321	1.034471304
P18531	1.529453963	1.319300457	0.890211817	0.79945231	1.203471833	0.911175394	0.81431493	0.904190211	0.878190868	1.803380707	0.74488972	0.711843539
Q8K1R7	1.063902516	1.216656697	0.978095211	0.966181776	0.992502926	0.972674889	0.948401895	0.982373758	0.984981652	1.027994328	0.983423065	0.937210636
P16390	0.962109151	0.996211967	1.007551963	1.046759522	1.153778974	1.05764334	0.991787973	0.988570764	0.923775964	0.979112131	1.012246096	0.911286056
Q68FH4	1.025218104	1.015252709	1.079948522	0.976070793	1.011439208	0.949861116	0.969678269	1.016595739	1.006029012	1.052255729	0.978404769	0.962113248
Q8BTY1	1.162936523	1.066932108	1.068716582	1.008927926	1.001783864	1.020441336	1.004957649	0.958278621	0.918222175	1.01292055	0.92149949	0.928690978
Q6DFV7	0.934010496	0.961165692	0.970721005	0.992553419	1.050238797	1.010404967	1.010718509	0.967249002	1.008828529	1.074264962	1.04397982	1.018351173
Q91VF2	1.000507751	0.929154446	0.995665469	0.995894406	1.007701142	1.101535656	0.974237663	0.945189657	1.005549757	1.006939573	0.998133485	1.077977495
Q61136	0.97052244	1.013856673	0.947145505	0.963441449	1.068421009	1.006033689	0.993532192	1.000123652	0.968252408	1.074328484	1.042495609	1.005363201
P09602	1.156118323	1.124217137	1.083311668	1.148099715	0.986946868	0.997687786	0.955265414	1.032427731	0.911200306	1.123205747	0.792284419	0.83953979
Q9Z239	1.043764475	1.048251798	1.127369254	1.047988893	1.23843896	1.310856451	0.857773976	0.780444423	0.867006738	0.784067109	0.941813671	0.970125031
Q3UN02	0.961974076	0.961215417	0.982883864	1.005589366	0.928114754	1.010082498	1.042902485	0.977337862	1.019532474	1.010175613	1.035571337	1.076304637
Q9ET22	1.010050993	1.064727137	1.053479967	0.999864574	1.06107856	0.982273473	0.99130607	0.967277084	0.909195449	0.991171357	1.027418986	0.973993909
Q8BM85	1.009009753	1.029858962	0.970243469	0.987011316	1.006837221	0.999896632	0.961283557	0.962668379	1.005875611	1.045146758	1.019324006	1.053014229
Q99JT9	1.075733173	0.987464262	0.971863152	0.911176619	0.991490766	1.214163938	1.134734387	0.977819277	0.906530509	1.255094972	0.864732945	0.870979184
Q9D2U5	0.937785339	0.945818936	0.950489774	0.971779347	1.039878335	1.041474553	0.967960325	1.024722649	0.987911781	1.090017022	1.02119839	1.080319728
P70288	1.004502555	0.956622533	0.976577886	1.01050676	0.969544583	0.998775172	1.012788263	0.998358505	1.019612673	1.006851688	1.033633931	1.030730753
Q99N95	1.228064531	1.179523283	1.291129077	0.762133909	0.737006604	0.78459002	1.2914181	1.265143769	1.155937117	0.705470914	0.717832194	0.720604324
Q9CPT3	1.021647383	1.032901551	1.042046736	1.023143057	1.08884728	1.031281542	0.92057307	0.950097355	0.954521456	0.935702206	1.023891168	0.994227993
O54962	0.878774328	1.20959927	1.122116412	1.248356398	1.118654452	0.896236312	0.673834803	0.914197816	0.998045139	0.549741912	1.155753398	1.089307366
Q60710	1.101727733	0.972446678	0.998999914	0.931533325	0.919910628	0.962749777	1.081306154	0.996786708	1.079760294	0.954308028	0.997460356	0.989624918
Q9CRB8	1.022010161	0.988436916	1.005542499	0.897975743	0.882967933	0.98272724	1.138794435	1.096096662	1.007012609	0.993586271	0.964301984	1.013313399

Q67BT3	1.09467975	0.984931041	1.053535511	0.988691172	1.039780189	1.015504054	0.991151964	0.945262915	0.971273608	1.001940554	0.983036274	0.975770164
Q8VCH8	1.038061946	1.06073751	0.994114373	0.970567815	1.051019156	1.003551479	0.975445613	0.938246444	1.002739085	1.033656131	1.008055761	0.972365306
P53995	0.979406852	0.993906202	0.964632854	1.027286556	0.961929002	1.036269517	1.01181405	1.024027162	0.975399012	1.030320381	1.004163488	1.029879389
Q8R3L2	0.977360466	0.951569738	0.946246992	0.940302955	1.028483736	0.93869407	0.961680604	0.943073654	0.95462758	1.133890746	1.183282067	1.099730315
P28184	2.183028375	2.037808706	1.331826196	1.001190207	0.631953587	0.667748994	0.552846024	0.871681205	0.837408511	0.544102968	0.697637622	0.718727904
Q8C050	0.981040601	0.970388643	1.078373295	1.06563613	1.129363341	0.985335388	0.952656069	1.163278662	0.937432036	0.971951356	0.952317659	0.831151015
O35114	1.0482737	1.023821929	1.034748621	1.026224203	1.070995423	1.036627473	0.982199945	0.961763065	0.975699177	1.014493506	0.929415092	0.956359286
O88736	0.99126085	0.948006402	0.978389333	0.958990614	0.989730203	1.052377988	1.054594295	0.916537285	1.027573246	0.971665919	1.00802379	1.111892874
Q8CB44	1.010467734	0.983091765	1.023603834	0.978552331	1.001342233	0.941564272	1.052686123	1.019305976	0.961363117	1.003706819	1.056108006	0.976801582
Q9R112	1.072872373	0.993916209	0.980783775	1.059349318	0.937100318	0.995030597	1.006733285	1.035978571	0.978229787	0.934319478	1.058708692	0.942958625
Q6ZQ88	0.949832101	0.964295922	1.05265947	0.976593052	0.962624221	0.976094255	1.11374434	1.074342561	1.028919819	0.932271662	0.964634905	0.967539199
Q62507	1.169833052	1.024858063	0.986759783	0.981334061	0.986402708	1.015457962	0.966906611	0.961971619	1.034929269	1.12535264	0.897337979	0.96493201
Q9WUR9	0.889286261	0.994864737	0.950911598	0.965087582	1.055260631	1.041894928	1.013275145	0.905257153	0.984536251	1.107779412	1.062378053	1.088486903
P53986	1.110743728	1.009162204	0.926652281	0.930416256	1.094666649	1.087317339	0.952378646	0.942591592	1.0260684	1.265982304	0.933765715	0.889154638
Q8K1C0	1.339583972	1.161553067	0.730088966	0.78115567	1.244892289	1.17845141	0.753083491	0.733573793	1.209815615	1.703267101	0.786686053	0.80563315
P24472	1.066506267	1.062226191	1.094840208	0.971776463	1.006923358	1.125968398	0.990780078	0.952976065	0.933149133	0.911586488	0.972091661	0.918899916
Q8BPM2	1.045280288	0.935662295	1.067722565	1.044220014	1.095853198	1.043230351	0.96599483	0.950618136	0.893991129	0.980417091	1.005605872	1.019623347
Q9CR84	1.001132734	0.961887563	0.985121874	1.047863532	0.887528506	0.993511563	0.989651446	1.00226808	1.004754995	1.024302334	1.005837629	1.133190157
Q9WVQ5	0.984379123	0.93162376	0.995605247	0.982112611	0.987223195	0.993610423	1.048789109	0.966433429	1.001119055	0.95790137	1.052368462	1.091386018
Q80YF9	1.035826421	1.042453473	0.9532996	0.972925528	0.957675783	1.004021359	0.961206419	0.956071625	0.982552847	1.044234745	1.174756095	0.936464589
P83870	0.988948976	1.032444965	0.964706083	1.022635679	1.028281356	0.976980448	0.927847636	0.9812106	1.048667718	0.952758329	1.028919918	1.053752822
P58466	1.172663951	1.04170368	1.033168189	1.015755126	1.082787172	1.074992033	0.955311823	0.963748415	0.925494437	1.032321289	0.919791121	0.876524803

P83887	0.987747699	0.991724603	0.995493636	0.96485744	1.019792922	1.055143424	1.034905877	0.992460172	0.955093727	1.136904556	0.965876659	0.988401183
Q921H9	0.968830776	0.986749408	1.016646607	1.028808453	1.036035602	0.988404737	0.944293715	0.968283834	1.01552292	0.905934881	1.049956611	1.073232992
O35900	0.953875729	0.936583586	0.945550707	1.027501586	1.022061281	1.12277974	0.976691419	0.932425516	0.955942679	1.034198049	1.063622976	1.076676946
Q8BGY9	1.039316424	1.015090719	0.991067942	1.122724003	0.99428025	1.052133633	0.924069061	1.043414305	0.980266195	0.94438863	0.971871791	0.950924343
Q5SUC9	1.034718247	0.955742594	0.98628767	1.02469228	0.942871231	1.04055092	0.989611879	0.944357316	1.077442534	1.19477717	0.911648019	1.017566441
Q8BVG8	0.947758828	0.940386999	0.967308083	1.01617722	0.960898736	1.043924668	1.003758355	1.025376494	0.942715733	1.218604859	1.011704762	1.036614063
Q9CZ83	0.984121775	0.992164477	0.981869753	0.936990781	0.933307026	0.993659898	1.033423105	0.950636236	1.013942909	1.08067602	1.130858742	0.988953137
P01843	2.276001824	1.607472504	1.344206714	0.814749908	0.750294784	0.775205493	0.786832801	0.899950009	0.821398239	0.741656329	0.666741782	0.649826145
Q8BH27	1.0856409	1.053525438	1.035044545	1.0066902	0.932777912	1.00146645	1.002673553	1.010402188	0.992652293	0.890835014	0.971489315	1.007607518
Q9CXX9	1.045706537	1.112465852	1.02354084	0.961179773	1.039778288	0.999584386	0.967968752	0.991585914	0.98797255	1.004295573	0.988624513	0.911843422
Q9QX66	0.969224992	0.945599599	0.930648401	0.952435724	1.060609262	1.001810428	0.975610113	0.984567498	1.051393277	1.205284086	1.003395963	1.023973822
P61460	0.948360041	0.927054862	0.976546903	1.047589418	1.083433837	0.959495687	0.936579443	1.033527449	0.978190932	1.019719316	1.068615104	1.050416057
Q9D1I5	0.931170891	1.037842736	1.027655028	1.036631648	0.933363263	1.006958567	1.009135106	1.00611845	1.026020019	0.929656847	1.00130191	1.034066335
P57080	1.122485539	1.040680943	0.97519733	0.714399704	0.849748903	1.012401697	0.944119056	0.9822614	1.111615752	1.238367531	1.055416894	1.043483636
P62080	0.929910867	1.003186804	1.047141117	0.98541896	1.131288022	1.001556933	0.984479967	1.006888019	0.945447454	1.030477964	1.037106445	0.925641213
Q9D0V7	0.945289467	0.979052388	1.00523477	0.967907684	0.984478152	1.055874092	1.012894855	1.020839418	0.97453113	1.114218945	1.023846654	0.972415063
Q8BIE6	1.03715159	1.023484435	0.959603439	0.993591287	1.016242737	0.97829334	0.965448801	1.009054706	1.023604042	1.169789238	0.962288432	0.968518988
Q9JM63	1.275267152	1.028325473	0.979396644	0.856193616	0.907463576	0.924018585	1.072911147	1.020284384	1.110655078	0.95285735	0.948443586	0.928084852
Q9D7I5	0.924451224	1.009181489	1.017995475	1.081555798	0.945380333	0.973903172	0.953938486	1.067390642	1.042279855	0.878261028	1.022948893	1.038970812
Q61578	1.073684028	0.922790246	0.973956017	1.091449172	0.950912374	1.01930258	1.042714999	0.960092764	0.955742393	1.006359659	0.998898386	1.054715899
Q8R4I7	0.938247691	0.989102497	0.946112975	0.970578622	0.983716185	1.008040461	1.052144572	0.987122134	1.069290028	0.985169567	1.012097269	1.054218295
Q9Z2C5	0.972974175	1.058436653	0.880616427	0.94001215	0.960025405	1.03757728	1.06003839	1.058094633	0.967999884	0.999955525	1.041447896	1.035708701

P84104	1.083901155	0.98696556	0.940017631	0.933462097	1.153386781	1.116946789	1.016648414	0.970953799	0.862739016	1.332311402	0.953180383	0.856162413
Q9CXI0	1.018871238	1.018666358	1.025522027	1.055450062	1.034431533	1.169308317	0.958434695	0.943603511	0.924088512	1.051790766	0.917851541	0.973951849
P12265	1.077283459	0.999602016	0.991605151	0.959536622	0.990508654	1.102363442	1.030729051	1.012383964	0.973746348	1.085066558	0.932589469	0.922836233
Q61503	1.030721493	0.996045321	1.042355362	0.96088658	1.047716326	1.096721211	1.055006351	0.945568951	0.950014029	1.112985311	0.918617356	0.935144829
Q922H9	1.073071622	0.953549685	0.943018692	1.057748688	0.877940546	1.070062529	1.139155175	1.108012615	1.087923263	0.943427346	0.828347352	0.925878238
Q3UUI3	0.885950201	0.928842939	0.940624338	0.995738944	0.954546981	0.986742179	1.107468195	1.080850328	0.989897694	1.043014151	1.039703034	1.053043726
O35316	1.448940515	1.100855663	1.132622375	0.920420982	0.92437375	1.073427892	0.909165876	0.96145405	0.953638863	0.922683841	0.850828043	0.876438986
Q8R059	0.948140886	0.947576063	0.962013932	1.060092516	0.969012658	0.91589017	0.935394021	1.053336002	1.160907811	1.124989536	0.998931952	0.972798705
P70295	1.137234524	1.076461481	1.176808567	0.941919504	0.862662451	0.892592659	1.039330597	1.021774606	1.091284407	0.910005543	0.925211165	0.890902381
Q8BHJ6	1.214412494	1.104060498	1.124460831	1.080308005	1.139726247	1.093921249	0.883485764	0.943196327	0.911415188	0.83036671	0.885845343	0.823102108
Q8BIQ5	1.016633022	1.04270705	1.00073932	1.045954436	0.971233896	0.985153307	1.026465754	0.999784676	0.99888732	0.992892531	0.982085868	0.962558693
Q7M757	1.082253697	1.073985201	0.868737426	0.928385682	1.127393352	1.018312803	0.927353024	0.944213287	1.019272462	1.356827224	0.897661616	0.977234244
Q9DC23	0.964603929	0.995650273	1.025307359	1.004503604	1.09092755	0.887032685	0.960197168	1.028340503	1.024473741	0.847265209	1.052320505	1.066195887
Q3U3E2	1.022616581	1.037881118	1.065425094	1.061082722	1.033826226	0.949626364	0.941251392	0.986763908	0.976262716	0.911531134	1.000891038	1.014935348
Q76LS9	1.027721766	1.010019952	1.089213492	1.097908695	1.133900821	0.965691104	0.903373416	0.952836476	0.96993068	0.921596789	1.007438844	0.936788574
P19324	1.138516122	0.972256044	0.994260353	0.918246444	0.947632276	0.979242974	1.026095514	1.032139808	1.030530405	1.033070569	0.996656712	0.966006203
Q9D0T1	0.990763474	0.968837748	1.060247694	1.015633424	0.83269775	1.058028064	1.086298316	0.943470427	1.082339215	0.832345602	0.975478223	1.087462436
Q9CQU3	1.04503328	1.085346746	0.987931326	1.036657797	0.928046439	1.029337489	1.056144558	1.027294303	1.010120121	1.064361582	0.897504424	0.896942582
Q8R5A3	1.042931708	1.019530897	0.996035062	0.933651613	0.946339227	0.941217131	1.088609636	1.129592362	1.020114223	1.146811294	0.908713099	0.897906193
Q9CYA0	0.808185779	0.977470844	1.000642119	1.242187671	0.864023765	0.664976287	0.875963128	1.059371712	1.264182286	0.86697713	1.129296663	1.143440404
Q8C0L6	1.014720777	1.026183813	1.032594811	1.052720606	1.059740181	1.042505024	0.961109811	0.965787903	0.934866926	1.000278573	0.992686667	0.965838544
Q9D517	1.015813599	0.926970532	0.929730108	0.934093843	1.02330432	1.130030472	1.118521919	0.965017089	0.974706102	1.371313384	0.89220375	0.912525713

A2AT37	0.997310072	1.006138601	0.987348673	0.957984568	0.955852818	0.996740982	1.076473579	1.034227708	1.017921976	1.070316856	0.966901135	0.972410562
Q91VC9	1.623624751	1.392726427	0.665319287	0.703405167	1.430165125	1.212949768	0.665575456	0.649432536	1.136641495	1.90032211	0.602094602	0.630856778
Q6ZPR4	1.57258962	1.163550224	0.772862436	0.705715876	1.267973018	1.082585056	0.744579681	0.823343652	1.20724641	1.924238396	0.662054593	0.621253052
P06797	0.967187411	0.954628999	0.964909175	0.951443603	0.986565781	1.024339384	0.997833282	0.979512243	0.992176669	1.177461269	1.081268603	1.001198314
Q8R2Y8	0.976247503	1.040725241	1.082186098	0.854661621	1.119026681	0.929937618	1.070066456	1.011910908	0.905187995	1.124631561	1.043461441	0.893637517
Q9WTX2	1.150501899	1.111156483	1.122884811	0.929393783	0.906440101	0.924527518	0.993554692	1.066992827	1.057767783	0.797599969	0.942754479	0.929652524
P03899	1.099878477	1.065282394	0.94764627	0.916017162	1.103082471	0.995599876	0.882800652	0.945567052	1.061510102	1.14755808	0.983667342	0.960630235
Q6IMP4	0.891675774	1.005653047	1.015693609	0.935371786	1.032014115	0.885648976	0.983269894	1.034177704	0.927652938	1.29376483	1.105929341	1.001825463
Q78IK4	1.059530847	1.007268925	1.034343961	1.091162604	1.034210296	1.045951247	0.965452415	0.931544688	0.975691808	0.881746996	0.970798989	1.011810831
Q9EQJ9	0.978138986	0.96806679	0.979643668	0.958710824	1.036306427	0.999022906	1.007828334	1.029697868	0.992762259	1.026371981	1.042896288	1.001947083
O70481	0.961544905	0.967969442	1.020608329	0.977327899	1.014455189	0.931914798	0.976497373	0.960957638	1.005040656	0.965379119	1.138758538	1.059615462
Q9DCL8	0.860118727	0.952381922	1.00505073	1.196944262	1.030183057	1.175010742	0.931554681	1.070205216	0.977794986	0.916752796	0.896190218	1.00642592
Q9CZ28	0.98241261	0.972751079	1.005480277	1.045628782	0.994381109	0.91467663	0.962783134	1.005176546	1.03488484	0.847987154	1.07660176	1.104145023
Q80X82	1.004651454	1.644881721	0.930808313	0.937457121	0.962476211	0.883931164	0.953361014	0.918114768	0.891763149	0.941511629	1.049458677	0.901563367
P51655	0.836854184	1.305298677	0.962632097	0.974817021	0.964160659	0.973998772	1.011521804	1.014735228	0.960975878	1.063708207	0.988016418	0.980555172
Q80U58	0.946084651	1.00552876	0.978492245	0.959738233	0.970786183	0.965609082	1.037490151	1.028528433	1.035418197	1.10453823	0.98510958	1.031122646
Q64008	1.054420432	1.087649191	0.974798626	0.925020925	1.021867024	0.984556435	0.987765877	1.03108597	1.020384698	1.183235986	0.950839718	0.882908775
Q8VBT0	1.129606109	1.045961923	0.946563158	0.92890978	1.071802739	1.137783841	0.899027729	0.894128218	1.064685861	1.340303418	0.891770481	0.860635
Q6P1I6	1.052338169	1.021807028	1.025310514	0.987673222	0.987950376	0.992808591	0.978521777	0.981622137	1.02083994	1.010008723	1	0.971574753
O08784	0.975392786	0.937014708	0.995903192	1.038095802	0.96363173	1.059675749	1.042896511	0.976249488	0.989730566	1.082301571	0.941279229	1.067626432
Q8C1D8	0.951339486	0.983135673	0.924723717	1.014009537	1.041076904	1.025974969	0.999638931	0.979145799	0.991780589	1.114068524	1.032330849	1.014728734
Q8BGE6	0.950362306	0.999483986	0.983696094	0.963733398	1.015889424	1.033029333	1.030087213	1.027904325	0.995372319	1.023829108	0.980682631	1.023240183

P01592	1.142255941	1.175788076	0.982138084	0.913235211	1.034384082	0.969553513	0.929376286	0.971894682	0.964201911	1.125210636	0.987626028	0.909536267
Q7TSG2	0.947400969	0.90787832	0.956664139	1.052813817	1.11112779	1.047854788	0.933985707	0.983220638	0.949179267	1.156993737	1.02451477	1.036466733
Q80YA3	0.987853599	0.980710532	0.990108822	1.005436345	0.93634829	0.911542838	0.974458203	0.970547323	1.074056613	0.888793657	1.14214289	1.079498
Q9WVI9	1.003059482	1.012505731	1.023986122	0.914049865	0.972154708	0.985529757	1.056153358	1.071692899	1.008975854	0.981636284	0.989404112	0.974942629
Q61625	0.951526233	0.939650353	0.972679239	0.929295279	0.89807545	1.012114674	1.068302512	1.01149245	0.992086474	1.069773699	1.103326099	1.064814682
P70444	1.015130291	1.026679439	1.100776469	1.092961554	0.932169913	0.896230205	0.960695002	1.182531122	1.037530099	0.862651508	0.975656224	0.863192621
Q0KK55	1.110284327	1.040958005	0.995898201	0.921081238	0.965451838	0.959192822	1.055865308	0.962379431	1.041332906	1.08284602	0.94833666	0.979714885
Q9DB75	1.204252217	1.17733174	1.105363281	0.986527809	0.983060352	0.955858738	0.924824642	0.927009267	0.918251754	0.939231167	0.968993373	0.955235987
Q6QD59	0.944156367	0.978961274	0.997830664	0.986763841	1.0572191	1.027844871	1.000231007	0.988322655	1.009793544	0.940706487	1.051281458	1.001346748
P03911	1.0548531	0.99682622	1.011658869	1.038464949	0.991048767	1.022468796	0.991692981	0.982485416	0.972897885	1.032786681	0.97307189	0.989697998
Q80YV2	0.898349734	1.008337246	0.95059346	0.971786033	1.096074645	1.012675221	1.022099521	1.039599501	0.918135715	1.162962686	1.048239358	0.952325191
Q9D287	0.984508143	0.998240804	1.049068038	0.96940719	0.967665314	0.991073091	1.012263237	0.991034038	1.005216071	1.001285529	1.030528393	1.006647358
Q61189	0.963924773	1.022872532	1.018058758	0.99599477	0.993593355	1.012491898	1.003201673	1.013654794	1.04746625	0.941600327	0.989335868	0.984276289
Q8R3Q0	0.990323846	0.986477114	0.975224695	1.025692618	1.001925908	1.027176152	0.988255021	0.994645956	0.970985734	1.102332258	0.978812625	1.039422101
Q6PGE7	1.039465405	1.116195075	1.012485463	1.02088816	0.886239618	0.993883336	0.97940106	1.019457653	1.008588231	0.954327485	0.983635268	0.994311448
Q69Z23	1.083318386	1.109571212	0.988708219	0.965358951	0.92516712	1.057222683	1.01114229	0.94048367	1.012167496	0.997184108	0.916418131	1.043314287
P05532	1.020907169	0.960579249	0.983226521	0.985242	1.011868882	0.973636741	1.021884778	1.024807922	0.99398944	0.975229487	1.054171683	0.996579792
Q8R313	0.915175711	1.027396981	0.965422545	1.074006818	0.968253184	1.012931132	0.984231946	1.006691735	0.993833012	1.002782625	1.00560256	1.069776676
Q9D1G2	0.995480091	1.005361941	1.043234161	0.9453598	0.988298288	0.966712483	1.012588367	1.019304246	1.03073017	1.00203441	0.999625651	0.99819682
Q3TKY6	0.990392526	1.11509683	1.060430618	1.033851007	1.072243906	1.003099454	0.952063321	0.990928155	0.904004306	0.924906218	0.988371317	0.981551479
Q8BXN7	0.989951293	0.972859133	0.998137186	0.982185778	1.017299651	1.004215684	1.027243434	1.018369198	0.954173903	1.024049489	1.023669897	1.018597372
Q6P3D0	1.031487223	1.12290685	1.147921815	1.019962015	0.923866705	0.953117538	1.063025736	1.100442484	1.072048203	0.766205877	0.824728611	0.895811399

Q9D4F2	0.951027704	1.050185001	1.036249452	1.060490327	1.131377292	0.907379844	0.894107068	1.04357795	0.98271403	0.757907901	1.119874964	0.976496285
Q8VD62	1.040138519	0.973705246	0.992491566	1.004579637	1.043633303	0.982557392	0.98266526	0.963897169	0.963798152	1.132858179	1.047170808	0.958181964
Q504M8	1.012336953	0.989250595	0.957545048	0.959309266	0.937033504	1.055382556	1.040963284	1.026279731	0.913749129	1.22178865	0.968758924	1.047384262
Q3TYD6	0.832033425	0.931180096	0.93282845	1.025706993	0.927232502	0.906967628	1.062897813	1.020712494	1.024621607	1.106577666	1.128042538	1.116046024
Q9CVD2	0.930307938	1.093095724	1.059203332	1.014782514	1.068423535	0.981891978	0.896830314	1.056605602	1.036758655	0.913151718	0.976908325	0.955111323
Q9ERG0	1.03654087	0.891379022	0.970689695	1.00376207	1.072069267	1.014758165	0.971699803	0.995288577	0.964616161	1.183332007	1.043764701	0.960242439
Q7TPW1	1.03794103	1.047371691	0.977604169	1.058109652	1.045673837	1.03244286	0.927494625	0.998367964	0.954068781	0.906784726	1.043514831	0.977320331
Q6NSR8	1.084210583	0.994377773	0.976602535	0.96217961	1.003741548	1.077676596	0.963698247	1.00169631	0.986360881	0.96596736	0.990482161	1.023200708
Q5NCI0	1.016949188	0.953039722	1.051677805	1.016320669	0.941290446	0.934176025	0.989237833	1.01995949	1.013645064	0.962942625	1.047265273	1.046116534
Q6PDC0	1.000039995	1.051011527	1.049076061	0.999767807	0.987349612	0.939637695	1.019469845	1.028194845	1.004162952	0.913705897	0.989148608	0.998239987
Q91XD6	1.016917184	1.019537505	1.001708341	1.056533446	1.022574851	0.97400116	0.968581985	1.036865545	0.972207275	0.944043183	1.008919946	0.989001616
Q3UNA4	0.98197859	0.986247767	1.019890785	1.053559319	1.059179829	0.968166141	0.930270774	0.993183753	0.996017642	0.885235666	1.059882923	1.044176083
Q5NCF2	1.000165302	0.994141508	0.993370704	0.931476491	0.953560543	0.933894894	1.055112072	1.081814649	1.052888252	0.876837017	1.087068455	0.965301645
Q80XU8	1.004772669	1.008925468	0.975216043	0.951984666	1.042469246	1.002751039	1.013949519	0.957305749	1.059324484	0.995933278	0.977877473	1.030816959
Q9DBE0	0.944713208	0.966575466	0.933092928	1.036022919	1.023911483	0.990327029	1.011433787	0.984909404	0.97596628	1.063549815	1.055633977	1.061345755
P69566	0.875078438	0.942884997	0.971659316	0.945262474	0.947481514	0.880018477	1.000517738	0.973659786	1.157052992	1.18237468	1.035724846	1.138345092
Q8BZZ3	1.014720945	0.96896504	0.984262685	1.016825378	0.96785451	0.997955562	0.994222995	1.015155715	0.98569874	1.03034527	1.001070266	1.065707359
Q8VE92	0.962537758	1.010089814	1.034292926	0.955364687	1.016248952	0.941399352	0.982352331	1.08746121	0.959400928	0.929329921	1.065926445	1.028073196
Q8CEI1	1.068078	1.011231015	0.999964747	0.981853075	0.993792657	1.040616071	1.003161815	0.96663073	0.998189076	1.007135262	1.002735259	0.962263602
Q8BP40	1.08852338	1.027861414	1.047794684	0.952633142	1.030166556	1.039743475	1.064102499	0.966947902	0.988477187	0.994729073	0.924924239	0.910812211
P14220	0.892275172	1.144577414	1.206187514	0.539092012	0.399164231	0.431204428	1.620388964	1.859583912	2.016731679	0.718019941	0.45600169	0.29887104
Q60766	0.950605706	0.974565112	1.058128103	0.99540003	1.023587448	0.907563556	0.979919843	1.178030961	1.009408641	1.000962717	0.94841042	0.980024296

Q9D6T0	0.970782735	0.976530687	0.929001083	0.982272907	0.885962326	0.976047284	1.108173075	1.040416453	1.046287449	1.01425317	1.002680074	1.072016854
Q9CQ79	1.012580212	0.961807013	0.981464073	0.975742046	1.039334908	0.979174979	1.027553455	1.006510738	0.970403955	1.102710837	1.024142265	0.982610822
Q8C2E7	0.98969008	0.921373289	0.971904096	1.010708516	1.058283559	1.014324689	0.998015484	1.015609133	1.052486539	1.086178856	1.011355422	0.91918913
Q61127	0.952566467	0.961248513	0.954224596	0.98521703	0.959088357	1.034534944	1.054362329	0.993940503	1.03084033	1.097298931	1.004766942	1.020645477
Q8BRN9	1.023904388	1.037820494	1.030243602	0.982633587	1.051562557	0.995543962	0.9808106	0.96042116	0.902247211	1.059343163	1.082164288	0.946205871
Q91YN9	1.038469032	1.039883312	1.037111014	0.939871125	1.0339062	0.938682107	1.003119843	1.033705906	0.981003933	1.057124528	0.9970576	0.943122036
Q00519	1.087800751	0.994760453	0.994996764	0.982717556	0.949313322	1.057389286	0.983155546	0.990958703	1.021293736	0.949185946	0.982547191	1.021407579
P18608	1.043692571	1.017633896	1.109132329	1.149575268	1.037108594	1.063876954	0.967867247	0.916617328	0.976375914	0.91124355	0.854059599	0.99420211
Q8R2Z5	1.010809037	0.974377754	0.968208255	1.033453916	1.067445271	1.076107692	0.960061051	0.930522028	0.927180396	1.067973912	0.996831642	1.075174864
Q9CPR7	1.037645432	1.020409454	0.95272484	1.031272612	0.940060464	1.03712753	1.032770871	1.03040961	1.037119511	0.86761011	0.945657092	1.046995372
Q8BX17	1.003020354	0.997052562	0.980656831	0.983260057	0.976196545	0.99222812	1.018529912	0.987082854	0.96464912	1.050135902	1.037150162	1.053198961
Q07797	1.083517197	0.982926618	0.987310914	0.927726562	0.907375363	0.984585856	1.037603956	1.000898757	1.116081997	1.013166197	0.943900498	1.034680005
Q8C4Q6	0.969301059	0.948805285	1.035060892	0.980926924	1.030365402	1.102398108	1.010102919	0.9714903	1.014651338	1.005375905	0.941474851	1.022227595
Q3UFS0	0.952628335	1.109432195	0.914846244	0.995264039	1.025680099	0.892560022	1.053952085	0.99722187	0.929976246	1.091542974	1.06052791	1.033602861
Q3UKC1	0.983413886	0.980595695	0.98776149	0.996522205	1.001411858	1.020286076	1.017308045	0.966441635	0.969130597	1.04157134	1.02728088	1.050568943
Q9DCR2	0.878331135	0.865644995	0.947265148	0.968628724	0.917727894	1.027392928	1.053228732	1.046698618	1.06272154	1.002294258	1.10803766	1.091853488
Q8BIV3	1.004938105	1.02636735	1.04673925	0.987749796	0.985379934	1.001412132	0.983989603	1.027352885	0.995355778	0.9858783	0.969639302	1.00484211
Q32NY4	1.21839976	1.265644671	0.811015414	0.864390362	1.112917533	1.028787956	0.790033838	0.839053262	1.267278328	1.33554793	0.847363669	0.840319433
Q9CY73	0.859193433	0.941130467	1.023920431	1.012172876	0.904563546	0.772245359	0.972788966	1.041110635	0.997033114	0.757653464	1.318157202	1.241990819
Q99M80	0.99574973	0.99871777	1.001728279	0.950331678	0.980281379	0.989606409	1.018839581	1.049248186	0.986262532	1.086404328	0.99085626	1.003393098
P29416	1.049657847	1.030959599	1.015173319	1.010894067	1.068424628	1.077458071	1.00414879	0.943779783	0.958767919	0.994511183	0.898733723	1.01119709
P22682	1.005045786	0.960773366	0.970170099	1.022805665	1.08081533	0.98823077	0.969847773	0.9815198	1.007293241	1.050800745	1.020651826	0.994364677

Q8C436	0.973271576	1.102388652	0.96068428	0.940192113	0.84059902	0.867377418	1.082666095	1.072383632	1.059769676	0.949788885	1.075814648	1.026012438
Q7TMC8	1.402786592	1.100185767	1.048035167	0.921993044	1.059837628	1.034360138	0.901507723	0.83666313	0.983302924	0.895482657	0.98531364	0.881847876
Q6NS46	0.954953986	0.799852035	0.91195832	1.025727555	1.043390648	0.769785893	1.058923911	1.122458515	1.032819111	1.278582921	1.061251224	1.044575166
Q9QXT8	0.885024427	0.957411647	1.040268688	0.983283397	1.116828905	0.950438097	0.953669897	1.073058393	0.926410447	1.149670782	1.052941589	0.980373618
P31809	1.021411449	1.019366008	0.984271139	0.855372263	0.910480731	0.798634876	0.993249096	0.946793498	1.154345624	1.166045416	1.091981909	1.099621988
Q9CPP0	1.021118467	1.048650529	1.034003376	0.989550298	0.964084226	0.924057441	0.983617799	1.082435736	1.055935869	1.014719015	0.968616289	0.928767489
Q7TQF2	0.962252818	0.950709881	1.052258075	1.019581883	1.08569558	0.996554473	0.94284983	0.995210315	0.97772547	0.952576793	1.062793671	0.999910463
Q61672	1.055394602	1.017581957	1.022238605	0.982443954	0.977818262	1.013373967	0.974482889	1.009784469	1.020821385	1.01455507	0.966034485	0.983114592
Q8C0M0	0.951181931	1.020440411	0.992659001	1.003830548	0.922987186	0.95473629	1.039269295	1.082219293	1.029054724	1.004431847	0.994898825	1.006037212
Q9CQ85	0.853056748	0.92019736	0.96895465	1.03923243	0.923586057	0.927605274	0.962794322	1.048550778	1.059873872	0.987902689	1.13473514	1.143128731
O70362	1.190878272	1.091731226	1.09549662	0.858157388	0.924961441	0.904056378	1.0966468	1.041689026	1.122558189	0.884764691	0.86552393	0.888554954
Q9D8L5	1.018704626	1.106524882	0.894797592	0.938301149	1.106791678	0.997985074	0.949988717	1.01544134	0.956610587	1.387825025	0.957802883	0.879569766
Q9CQE5	1.235291143	1.06321345	1.085801344	0.938469607	1.032440864	1.058120737	0.966254121	1.039827996	0.977792693	0.88798573	0.882939851	0.852098991
O35188	0.878471802	0.93771653	0.959244014	0.942090812	0.971580847	0.831289308	0.955867926	1.042117214	1.082344789	0.956248712	1.195055904	1.188428979
Q9DCD2	1.004327206	1.018897873	0.974266502	0.995828842	0.984034567	0.94363849	0.942164084	1.075269802	1.01927829	0.980126781	1.0689339	0.992964285
P51906	0.96519056	1.092875088	0.957171842	0.948986665	1.022204955	0.956804762	1.056916486	0.998912651	0.910332586	1.12486717	1.002439278	1.043810926
Q61823	1.077815985	1.021309078	1.090901764	1.045475099	0.944071598	0.957480876	0.994458753	1.056371895	1.028098747	0.894521123	0.933824285	0.940834931
Q64669	1.409664235	1.116083195	1.159968049	1.069081991	1.173792561	1.22365633	0.821370462	0.823451778	0.889485018	0.834677407	0.806436745	0.767252298
Q8BKC8	1.004996074	1.069523694	1.063688768	0.965751607	1.000965332	0.815140254	0.984683507	1.105310052	1.016859309	0.927064994	1.102244824	0.893195974
Q5DTM8	0.989331799	0.985666208	0.998432163	1.007460567	1.030612797	0.952031254	1.049250534	1.052176847	1.01320131	0.980899939	0.972555658	0.976878422
Q641K1	0.89720959	1.165753331	1.007686832	0.938007135	1.001847697	0.85325301	0.91342309	1.032264653	1.04475494	1.124093452	1.097089302	0.95838838
Q99M28	0.942620667	0.967172769	1.097342428	1.088253223	0.987137654	0.854560478	0.989429672	1.204695186	0.990926047	0.865294484	1.012725084	0.936653287

Q64520	1.000445943	1.036285337	0.974899134	0.997333114	1.069973274	1.040046726	0.977791364	0.941846486	0.962039381	1.026860766	1.012946041	1.012296989
Q8C561	0.969621027	0.967195741	0.968395721	0.967478165	0.990839121	1.02353854	1.022755645	1.053612203	0.942623186	1.157734647	1.021494685	0.997308681
Q571K4	1.000028849	1.102161827	1.02907356	0.958506687	0.916120138	0.871795818	1.048667805	0.964937658	0.985899498	1.117424047	1.005116977	1.06142375
Q5SXA9	0.992300913	1.009987541	1.009952211	1.0102631	1.069230451	0.952995017	0.955111255	1.002471127	0.934255783	1.043356014	1.060811887	1.005584411
Q61271	1.028354568	1.253257763	1.015820291	0.893516579	0.891831746	0.857227576	1.09091998	1.144237422	1.079132131	0.978224649	0.927784135	0.815977025
P0DN90	0.994447605	0.973058215	0.95352409	0.931199271	0.838009851	0.966550692	1.154855546	1.001556931	1.086248563	0.976673814	1.007017906	1.091757566
Q61107	1.008723754	1.032480406	1.037738693	0.959154839	0.967053422	0.939712777	1.058985098	1.062018093	1.026707133	1.036716866	0.952354762	0.940678702
Q9CQV7	1.034857681	1.048336145	0.994232268	1.023026153	1.014939103	1.055232066	0.99830562	1.020691902	0.945312581	0.969941333	0.963331481	0.96587758
Q6GQT5	0.964172683	0.96400515	0.992371293	1.044566899	0.913139586	0.93990431	1.010676217	1.041692021	1.081340246	1.093698101	0.989378978	1.00603818
Q9R099	0.959369726	0.891830268	1.013901042	0.99376508	0.930856886	0.977252721	1.045674014	1.04303861	1.081088329	0.940756697	1.014913907	1.073864233
Q8VC42	0.985060885	1.018813087	1.015788816	1.01341478	0.952330872	1.013835226	1.04351994	0.997732054	1.001530914	0.974743688	0.972516393	1.021862802
Q9D8Y1	0.974762898	1.120884196	0.988009611	1.028594154	0.950861787	1.095958684	1.00305014	1.032652271	0.990157002	0.870517182	0.953377158	0.970358976
Q62074	0.937643262	1.419477548	0.992235636	0.919859359	0.968432375	0.876632338	0.947372892	1.016580074	0.94122817	1.071130514	0.98016932	0.985749412
Q8C8T8	0.925020151	0.934331405	0.941584085	0.945688957	0.938863489	1.006419509	1.021922723	1.027340729	0.985048789	1.084636994	1.127737211	1.08378396
Q8CCP0	0.964711469	0.979572109	1.015090964	0.982959883	0.931382063	0.965172774	1.051862532	1.062628205	1.012028035	0.964323056	1.021676611	1.030776179
Q3TMX7	0.984698632	1.039469117	1.034529599	0.950007114	0.820951635	0.91716302	1.024357232	1.046349887	1.026834738	1.12736932	0.989284103	1.090635308
Q80W54	1.092647228	0.994063242	1.012813157	0.957430952	1.007956999	1.020263974	0.988271099	1.041743241	1.052552836	1.027944283	0.921768978	0.928001467
Q91ZH7	1.021594634	0.992956895	1.018116898	1.016931817	1.049927638	1.044624835	0.97194484	0.995042537	0.941594958	1.011082492	1.014015721	0.965281552
Q9D735	0.98378973	1.158569578	1.027936949	1.005505904	0.914638404	0.948153781	1.065951272	1.02647354	1.077240532	0.86026255	0.93221466	0.948379063
Q91ZV0	1.075146799	1.014567015	1.032114657	1.013081662	0.942839434	0.968313406	1.033941923	1.002584849	0.985906553	0.881660092	1.003264962	1.025091637
Q8BGA3	0.984883369	0.946344772	0.976723441	0.994672205	1.040433538	0.984230439	1.032191199	1.005134254	0.959298815	1.034892246	1.061401624	1.009641381
Q9Z0J0	1.00009433	0.97314762	1.046083151	1.018809537	0.965555702	0.940889285	0.930846747	0.975655441	1.042836899	0.834060047	1.107534199	1.099951258

Q9QXY9	1.028535385	1.167417508	1.119768865	0.992316577	0.870015254	0.882404888	1.031080863	1.106492856	1.005213227	0.947801435	0.944183413	0.886693775
Q9Z2X2	0.928580169	1.08912118	1.115603247	0.987620122	0.937175034	0.838098394	0.861246852	0.987278906	1.139932848	0.776593971	1.144489974	1.068978022
Q3TAS6	1.022476185	0.934792756	0.975372071	1.011464988	0.933884452	0.964437484	0.958791666	1.13595045	1.029386947	1.204149316	0.993347622	0.932215564
Q9CQL1	1.016704615	0.914955134	1.004372395	0.965937249	0.992699736	1.029895186	1.024919672	1.027597939	0.997691705	1.002148253	1.00313732	1.039374884
Q8R4G0	0.985820484	0.955378795	1.012819839	1.125850842	1.013997177	0.969076357	0.936024472	1.00117277	0.986977823	1.002514718	1.00509344	1.046441117
P08030	0.993419487	0.999801984	0.989374499	0.957908473	1.058303428	1.012954999	1.042361788	0.964767643	0.953441482	1.09549849	1.024995897	0.969778042
Q8R0X7	1.001386741	1.001635663	1.011175343	1.009016622	0.960075016	0.992670306	1.019988814	1.029488583	1.026600376	0.998898283	0.978092722	0.987425405
Q80ZM7	1.179352483	1.180420155	1.216704596	0.882951394	0.793953025	0.810297335	1.098441727	1.116251965	1.185136455	0.735881019	0.823740742	0.85414224
Q9QZD8	1.002171997	1.021203686	1.017982497	1.092923853	1.050899481	1.062010767	0.94385204	0.955417965	0.888610548	0.988583356	1.046930787	0.976513279
P12246	0.922234996	0.886539993	0.874128977	0.940603698	0.824231004	1.139702281	1.105305768	0.92342161	1.242028487	0.822655073	0.987148235	1.241385089
Q9EP72	1.011150984	0.949231671	0.979543012	0.953266908	0.940476887	1.047551361	1.069602998	0.987544646	1.090085968	0.886190313	1.003020952	1.036951445
Q920M5	1.085334025	0.955712331	0.915009928	0.896262359	1.042635855	1.044116169	0.977848054	0.998581491	1.136519422	1.246796096	0.949952569	0.874798021
Q9JMD0	0.994225785	1.12464584	1.040247841	0.952039044	0.976984841	0.923367849	1.013836669	1.011802242	1.032530003	1.045431322	0.994708071	0.913443888
P21300	1.166421486	1.002410548	0.99806214	0.970752979	1.026878402	1.057753873	1.001948957	0.904413291	0.979786038	0.93028157	0.970155457	1.025460959
Q6IFX2	0.903358067	1.05714027	0.98607802	0.998583897	0.879578664	0.906431543	0.967707725	0.970134837	1.122387714	1.46920062	1.004979169	0.914724885
Q8K296	0.87110977	1.012760183	1.027225433	0.859449821	1.015081368	0.964396109	0.970476382	0.927828424	1.171888847	1.263521467	1.013502253	0.983346624
Q9D2N9	0.965771634	0.999330004	0.943421576	1.047442857	0.962095845	1.055471873	0.973918086	1.008478169	1.022186048	0.954476842	0.994930028	1.084436893
Q8BH82	1.126231411	1.074658181	1.081071972	0.934841807	0.996597568	0.913886254	0.973650534	1.025961479	0.968946786	1.061397526	0.976621792	0.924743533
Q6P1H6	0.948407595	1.01862679	1.006149454	1.004312436	1.17222578	0.976453923	0.950418789	0.9701314	0.876801241	1.120082668	1.07413189	0.968199787
E9PZJ8	1.04646497	1.032058509	0.989016904	0.963545283	0.948831152	0.965037362	1.003772331	1.103541956	0.999839216	1.01376116	1.046304875	0.896908123
C0HKD8	1.000421959	0.964054296	0.986774629	1.019107364	0.993200722	1.007352669	1.044477014	1.050314808	0.990605818	0.995620584	0.969485122	1.001532682
B9EKI3	1.029560621	0.990598646	0.995123323	0.952576085	0.979523144	0.995776658	1.01558333	0.980338113	1.006083197	1.072588235	0.993639168	1.04311234

Q3U2S4	0.913333106	0.993248761	0.949714365	1.025168474	0.901495868	0.90433667	0.973438169	1.0873325	1.039235253	0.932813186	1.130149606	1.102512983
Q9ERA6	1.052229899	0.943156277	0.969166726	0.875344125	0.98322834	0.927886102	0.975533837	0.989455645	1.104934402	1.215427788	1.02934561	1.024406411
Q6V4S5	1.094142954	1.025618091	0.958820978	0.946725351	1.019332676	0.988448528	1.032448707	0.954184973	1.04308477	1.064913862	0.977176662	0.953912912
Q14CH0	0.946164307	0.951842698	0.977303921	1.019011604	1.001944666	1.008491613	1.037194356	1.007730094	1.024374893	0.948841927	1.020475956	1.045323863
Q9CZP5	0.902943507	0.940961079	1.021668664	1.047975369	1.0528203	1.035205251	1.014152893	1.019833886	0.952419843	1.00077477	1.018505424	1.010717476
Q99M04	1.10475342	1.025731895	0.924764819	0.934200759	1.073583508	1.056689408	0.972138851	0.93016939	1.030717304	1.244197728	0.916267738	0.948537998
Q8CAF4	1.046366001	1.026680849	1.01907632	0.911682007	0.976048064	0.925814714	0.98183653	1.064874892	1.02835648	1.006607646	0.980516106	1.050807272
P56393	0.958803676	0.91722229	1.006177248	0.99813321	0.986728362	1.096708953	1.059397823	1.035710178	0.947466146	0.891409596	1.082413301	0.976568042
E9PXF8	0.999648156	0.991088139	0.992366963	1.01444479	1.031783208	0.994624321	0.966409485	0.973514689	0.963662863	1.118914342	1.043457043	0.988483369
Q9CR59	0.98923305	0.955863133	0.913493548	1.061321399	0.903669806	1.073435023	0.972653374	0.947129583	1.179016537	0.974798999	0.932612836	1.11446323
Q8BH70	0.799894064	0.98763775	0.894041926	1.081462196	0.95331865	1.000008164	0.909630477	1.051190789	0.996672262	1.037901825	1.182296086	1.108347448
P97952	1.00344212	1.013547649	1.006542135	1.013595482	1.038083212	0.945314892	1.081828774	1.023201014	0.976083021	1.009327868	0.998106859	0.910601038
Q6ZWQ0	1.007736449	0.988429257	0.998756993	0.963216702	1.0688203	1.008613281	1.018685172	1.032792107	0.880133985	1.144258559	1.015987118	0.966991382
Q3USH1	0.987718726	1.090043574	1.007141292	0.963238446	1.015992547	0.962379578	0.99711074	1.087648096	0.956177614	1.101620458	0.977577211	0.916063429
Q8VI63	1.023900074	0.957894988	0.979015885	1.012418745	0.958974414	1.021715242	1.043500033	0.969483743	1.011938967	1.009137428	1.009782355	1.028989412
Q61923	0.918403227	0.853548893	0.983969	0.989980358	0.945442529	1.016597603	0.961180655	0.981253186	1.055564442	1.30680688	1.05647503	1.055763838
Q9QZ08	0.97327641	0.995685525	1.06056895	1.045542375	0.973369396	1.073794454	0.957005061	1.004331573	0.969984445	0.92123518	0.991407035	1.032955759
Q99KW9	0.928082204	0.947975703	0.911130592	1.004513743	0.872906083	0.946215659	1.079818355	1.090091401	1.061051661	1.043980892	1.061993295	1.049525
O35704	1.015624773	1.066566885	1.059720137	0.90525053	1.00325578	0.86621144	1.06859291	1.022593405	0.975411355	0.840899922	1.146220932	0.939328241
P05555	1.021340584	1.055783155	1.023928918	1.025076295	0.886540917	1.042227916	1.031995004	1.028884933	1.065944379	0.763071089	0.925296952	1.056089376
Q99MX7	0.935612212	0.914201811	1.007206168	1.011892224	0.99415305	1.039445455	1.039506467	0.952448432	1.007990965	1.070767201	0.996440471	1.077049888
Q9DAM5	0.980471902	1.078143782	0.935301252	0.955419337	0.955703219	0.948670031	1.123160315	1.013782081	0.972360637	1.217783751	0.946249158	0.982996629

Q8K0X8	0.941908826	0.968805174	1.016323815	0.998289378	0.998765807	1.00118429	1.02823864	1.053056563	1.050032404	0.924764918	0.994222338	0.993982072
Q61387	1.047958106	1.00096837	1.024413034	1.033843537	1.023961196	0.983621597	0.99805726	1.014678792	0.961023376	0.931256803	0.983295319	1.00882186
P61148	1.14648889	1.073013616	0.934985184	0.891652037	1.077898422	1.042545284	0.927524251	0.938648355	1.044446823	1.338481642	0.889592347	0.901805022
Q91Z49	1.064883145	0.984808559	1.051737381	0.900714342	0.966473445	0.935749118	1.127654377	1.090448206	1.06112385	0.840177399	0.946363741	0.958804617
Q91WE2	0.982373877	1.010245753	0.971215282	1.045338379	0.996837846	0.947643441	0.960171716	1.051032286	0.998677955	0.987728763	1.040430483	1.023167416
P06683	1.030253361	1.048901555	1.018654789	0.854815974	0.811288211	0.927386229	1.236424193	1.147349816	1.220932421	0.859518233	0.88212972	0.854468056
Q3UJD6	1.006792471	1.017250233	0.949652557	0.887836195	0.97025452	1.063326196	0.915233165	0.948731912	1.105975608	1.407184563	0.933262487	0.990840244
Q9Z2A5	1.102577251	1.034641358	1.068873384	1.06091089	0.998111233	1.137806638	0.992364204	0.971412336	0.958645283	1.015486503	0.825085821	0.920781542
Q9EPU4	1.203040939	1.077671599	1.081594095	1.078087991	0.965792331	1.002080069	1.007346688	0.990264714	1.087219396	0.811755488	0.815618159	0.867815281
Q9JIG4	0.907953808	0.895549497	1.020106121	1.032944151	1.023994471	1.0123438	0.964828387	1.050565523	1.010671015	0.958898304	1.064779878	1.040969773
Q9CWX2	0.98361136	1.054840112	0.985966343	0.887778032	1.014656658	0.936014378	0.980903788	0.960312829	0.971727308	1.046849641	1.210207338	0.967963914
Q9QXE0	0.965993191	1.054318542	1.017566916	1.044825275	1.100542182	0.973852972	0.982230304	1.041913848	0.922923944	1.014125821	0.996183396	0.926634512
P19973	1.260329839	1.189940829	1.194277917	0.97932298	1.027714769	0.933856059	1.129968304	0.947685265	0.98294511	0.717790656	0.788058164	0.799486831
A2AFR3	0.914049671	1.063257576	0.900869444	0.930071662	1.033390326	1.043516082	1.183677993	0.952670527	1.001409435	1.103395537	0.870091306	1.077255629
A2A6T1	0.979217494	1.02699493	1.013382303	1.011811563	1.016640267	0.983960531	0.974392495	0.957495527	1.015099323	0.851443616	0.989783759	1.151929824
Q9Z1M8	0.983311808	1.162832078	1.040093376	1.002052764	0.883711607	0.915727666	0.975386358	1.002420349	1.024054452	1.154589233	0.944888115	0.996210622
Q8BR70	0.946695933	0.989906677	1.05045772	1.041635197	0.945681037	0.985137369	1.022522249	1.083808718	0.909558393	1.105622058	1.051743022	0.915729523
Q9ER88	0.983450845	0.963912775	1.016762436	0.991568906	1.034382989	0.970428087	1.031989666	0.978166084	0.980043222	1.017347442	1.04079757	1.012506783
Q8R0P4	1.031467841	0.998371313	1.019354884	0.990490911	1.005933319	1.015834463	1.02702656	0.980457648	1.001943896	0.960063876	0.995192459	0.98234121
O54786	1.051388732	1.113854755	1.051931683	0.868874152	0.881124701	0.876407247	1.096799515	1.162242807	1.202864483	0.903963754	0.902697971	0.812985905
Q03717	0.979180342	0.872199217	0.904633606	1.194493129	0.965339713	1.020694017	0.855725566	0.85613865	0.856894674	1.919595713	0.991203444	1.056818987
Q8BU88	1.03010834	0.933425156	0.983944869	1.023113655	0.986157676	1.009326862	1.063120725	1.018296963	0.988833478	1.021193344	1.005104309	0.967267867

P15388	1.146448579	1.031201439	1.086176744	0.90489879	0.890160565	0.825332366	1.037372097	1.175654231	1.105217542	0.980253997	0.89952909	0.908845137
Q6RUT7	0.947805349	1.106878519	0.992102361	1.045570731	0.880259878	0.881549999	1.024300856	1.046777717	1.033821993	0.857770167	1.096058978	1.013382071
Q8C5L6	0.964635613	1.366248425	0.971589402	1.007722632	1.0387397	0.967279148	0.993155539	0.932191351	0.868556475	0.996788749	1.039067835	0.896361654
Q8BKG3	1.350659847	1.244749633	1.097472517	0.922756251	1.221402462	1.12531678	0.968565512	0.953061112	0.807258228	1.004197033	0.727816881	0.728899503
Q7TNG8	1.109937886	1.062878778	1.012050951	1.011431507	0.963065532	0.976848872	0.977186223	0.979835997	0.994995385	0.959070593	0.980535413	1.000108782
Q7TSK3	1.198378502	1.070190515	0.91211342	0.865453078	1.130031944	1.02114416	0.888344516	0.96469197	1.083124844	1.215959889	0.921171395	0.883800419
Q78J03	0.949283881	0.935321977	0.989851849	0.998737549	1.153148202	1.095224293	0.997088725	0.982634422	0.842313429	1.067206795	1.116676425	0.928825188
P19137	1.016598521	1.078379008	1.002919152	1.005745661	0.996673961	0.981855904	0.991688925	0.968838576	0.952178701	0.915127506	1.064448601	1.016428342
P11103	0.986895553	0.940751547	0.939784559	1.017794039	1.005452374	0.984058169	1.047755339	1.005585531	0.954465383	1.064841005	1.064584455	1.033033806
P97799	0.978461006	0.970092907	1.224432008	0.979467484	0.891045352	0.922060546	1.013212624	1.060379911	0.984998386	1.04001195	0.941384534	1.011189079
Q6P9J5	1.035807938	1.010938712	1.021463427	1.025880852	0.947990513	1.011621447	0.995555613	0.955827613	1.047680595	1.027724329	0.96921415	0.990509916
P21995	1.059528136	0.94159896	0.926143825	0.914558457	0.911405367	0.982883959	1.129726646	0.90735219	1.11847112	1.060770647	1.031436382	1.036840429
Q8K1S3	1.051260846	1.039805232	1.089789508	0.924676591	1.033609145	1.006593828	1.012276895	1.004356316	0.982289947	1.021207289	1.006072743	0.850111286
Q8BYZ1	0.899476293	1.042025339	1.059026775	1.076332323	0.957955079	0.913234034	0.949360923	1.034863036	1.069633672	0.758989364	1.062211321	1.068905648
Q9R1J0	0.998224196	1.064426372	1.015231795	0.911275213	0.908205727	0.911030328	1.052442143	1.122640073	1.068066321	1.055861583	0.984107763	0.914417039
Q8VHK9	0.961861709	0.968142829	0.955619385	0.987773543	0.917626213	1.020099983	1.014281021	1.001225718	1.108458556	0.995915285	0.969097787	1.107535716
Q6P5F6	0.936391188	1.015981547	0.914270768	0.966321894	1.00120116	1.009802306	1.074668806	0.980835486	0.973693345	1.086629894	1.048985927	1.037420202
Q3UN16	0.963455594	0.930184655	0.96752795	0.958912224	1.004919809	0.970505955	1.050306302	0.973919578	1.020816402	1.20223782	1.024342784	1.022769017
Q8VCG4	1.07927885	1.02634942	1.064388792	0.879711425	0.956649562	0.931467635	1.093031909	1.071895211	1.124519117	0.812943411	0.922376917	0.95377161
Q5DTN8	0.986753072	1.00279577	0.998201269	0.984678288	0.961686356	0.983672356	1.078762673	1.057906904	1.02093516	1.024786432	0.972079725	0.943879747
Q8BM65	1.007234809	1.010464942	1.01922335	1.05708782	0.959035394	1.00960408	0.953857006	1.012322176	0.983181056	0.88873199	1.02874602	1.05415503
P52332	0.940727546	1.035703976	1.011472027	0.963420961	1.043408544	0.934864616	1.056948539	1.09344346	1.009090661	0.999706091	0.996109624	0.910799156

P62309	1.134677549	1.17275173	1.17146003	0.744050776	0.67971104	0.690044456	1.408867554	1.288763727	1.362664147	0.715818278	0.727044319	0.689290213
Q9D7M1	0.982184345	0.963914303	0.977947245	0.947605501	0.958801477	0.92385516	0.998443387	1.005644554	0.967550099	1.069327387	1.130467092	1.10085431
A2AJT4	0.873733407	0.942019445	0.973809391	1.03047854	1.071599311	1.058417431	1.037730886	0.991010517	0.982715971	1.026365558	1.027958992	1.006240625
Q99JH7	1.086195915	1.11674704	1.062618348	0.933062075	0.884252421	0.890081659	1.103940896	1.091547445	1.134338401	0.765728213	0.909920945	0.917373959
B0F2B4	1.045499612	0.999878181	0.987085585	1.078663707	0.952979726	1.109184358	0.973929491	0.959880851	1.043078875	0.964431044	0.922859475	0.999917744
Q6IEE6	1.021838895	1.416964311	0.981449309	0.992537123	0.963400841	0.922408469	0.963226173	0.943057003	0.905040238	1.129767848	0.998513311	0.86280334
Q91WG8	1.130422833	1.06534152	0.949826352	0.910548703	1.139986142	0.987606966	0.917017025	1.017512094	1.019381284	1.156902829	0.951130076	0.872436158
Q9QYF9	0.970996513	0.906383144	0.953761338	0.99301435	0.953002517	0.950580194	1.055730086	1.006996443	1.074125722	0.982385009	1.068130894	1.067882365
Q60967	0.995243306	1.014171256	0.955878877	0.989282973	0.940004946	0.997352307	1.040606779	1.045205281	0.976362599	1.002102293	1.013136078	1.050005848
P97822	1.046383451	0.951001785	1.041574205	1.037656074	1.106167368	1.145478699	0.982223677	0.952245117	0.9433905	1.052376293	0.895789231	0.937224857
P43346	0.966002144	1.058978926	1.003474391	0.939546813	0.937219824	0.950587031	1.018464152	1.071820567	1.014455442	1.313207258	0.933561463	0.932218697
O88967	0.950833426	1.00647954	0.928451188	0.947198122	1.006034554	1.0594575	1.063048006	0.93692633	1.031102353	1.078978872	0.984775559	1.059147652
Q9D823	1.126322383	1.113196323	1.121184307	1.036326217	1.070049573	1.066588237	0.930752021	0.97907506	0.956289137	0.792921571	0.925290444	0.861373285
Q6P5D3	0.973562949	0.931184679	0.988469768	0.934012225	0.97870655	0.937664608	1.092066711	1.034264343	1.088033853	1.072234324	0.953766716	1.042402799
Q68FE6	0.943543957	1.026651163	0.973819452	0.998752701	1.05169168	1.009279972	1.001744997	0.979146988	1.011166787	1.016244522	1.028409887	0.98185729
Q3ULB5	1.035783357	1.003574747	0.998135356	0.988089463	1.001005462	0.980081623	1.014272096	1.00945607	1.029144671	0.98816914	0.975065791	0.997456126
Q8C0Y0	1.030605336	1.154245852	1.057700191	1.014265658	0.977095839	0.845520644	0.971924189	0.993596513	0.959165934	0.796019921	1.050139473	1.090374137
Q8BM72	1.024871488	1.178627784	0.909609819	0.868275954	1.098869693	0.986226495	0.849380809	1.072316164	0.993823935	0.878533511	1.12890924	0.976863169
Q3UQS2	1.079255041	0.943331103	1.011374426	1.08165005	0.950971589	1.069876255	0.95596115	0.958981791	0.99385451	0.948841148	0.996058556	1.038426454
P13439	0.990019953	1.124653416	1.026987522	0.939828605	0.969294422	1.002748608	1.10086081	0.980961879	0.986246664	0.979573797	0.942887545	0.965867488
Q8BW22	1.061385787	1.024861199	1.050302396	1.056141044	0.955630962	1.04934038	1.028699878	0.949099603	0.962533736	0.968156115	0.946481082	0.982372317
Q9D920	1.007291398	0.977731634	0.962842464	0.96854463	0.945526547	1.081953757	1.083336957	0.990297809	1.078659622	0.958997518	0.953442049	0.987415808

Q9D853	0.947495087	0.989293798	0.994325547	1.065335753	1.09902435	1.093421514	0.913709646	0.914185431	0.944441947	1.051130784	1.015779913	1.046483456
Q9QXW2	1.12979393	0.953236913	0.936675113	0.917489254	0.891629666	1.077568394	1.198900476	1.056046478	0.878260486	1.284598844	0.921496105	0.911606158
Q9Z2D3	1.065577807	1.018448999	1.087392878	0.786969864	0.83984932	0.846304109	1.108267795	1.434585458	1.204212759	0.819634487	0.830691101	0.82763322
P48024	0.977996776	1.079064708	0.991078602	0.926045398	1.191102516	1.054503439	0.90795319	0.878737844	0.959494806	1.09649915	1.091013111	0.918998576
Q91VU6	1.016244289	1.10192449	0.932418569	0.993888784	1.03446211	1.010303823	0.95860637	0.938935011	1.039315597	1.132762265	0.97671808	0.959545918
P07934	1.079322133	0.994566058	0.996735483	1.032530271	0.963569872	1.006770684	1.003223934	1.002213577	0.957803692	1.013559323	0.973287137	1.029553978
Q8K2T8	1.019224415	1.102950089	0.924462848	0.932989407	1.167005264	1.00190215	1.024743193	1.028049018	0.899112015	1.234030906	0.929618688	0.889047747
Q9D6W8	0.885878271	1.093592984	0.96788666	0.824762969	0.944214998	0.891605342	1.038105519	1.012000858	1.081308334	1.019961311	1.183766898	1.005708344
P0DP60	1.016037459	0.991178847	1.006274311	1.07203356	1.227959068	0.92050287	0.916186463	0.931682732	0.960999935	0.876881626	1.105771562	0.962982937
Q9R0D8	1.013724826	0.898156674	1.016538775	1.026202476	0.959300764	1.158460334	0.929085236	0.986496551	0.919632077	1.079965634	0.975758989	1.12723561
P62342	1.020611832	1.043792908	0.997735018	0.997328515	0.967491919	0.91600967	0.94766473	1.065807261	1.052208575	1.016042029	0.98259205	1.01868433
O08908	0.979668199	1.042677763	0.981261427	0.970649924	1.051924758	1.027359388	0.95796716	1.019297879	0.891329126	1.098498288	1.02471342	1.035801718
Q9JKF1	1.121165383	1.074937461	1.012662017	0.874175735	0.861012954	0.875418237	1.093212012	1.097765389	1.157398941	1.002177239	0.927943587	0.889284493
Q8VDU5	0.903763745	0.925566955	0.931179157	1.010681291	1.124339143	1.089649115	1.031038627	0.942153275	0.912649895	1.289302947	1.043505223	0.945494908
O70139	0.975597184	1.047402044	0.957460121	1.021406521	1.060575017	0.941444702	0.993775349	1.077537221	0.942955428	1.190176722	0.970303812	0.934275124
Q3UN04	1.041978414	1.015584298	0.986602263	0.98115541	1.021325683	1.033726385	0.954971447	0.943660742	1.004501934	0.937238309	1.037102011	1.050678721
P60041	1.047021502	1.108239224	1.01378747	1.092708681	1.044600142	1.139652342	0.92513712	0.9600555	0.917928875	0.826619037	0.940309772	0.991526793
Q8R1F1	0.998445942	0.979782553	1.007851616	0.971502476	1.052019121	1.030303506	1.03557349	1.022216731	0.995772376	0.992255837	0.967919637	0.967035193
Q9D554	0.859240072	1.023990115	0.99949289	1.067989296	1.043788729	0.902122701	0.884302172	0.996558034	1.008954816	0.840781152	1.172570023	1.129148966
Q8C5Q4	0.993361023	0.959952426	0.936593096	0.978817011	0.880908435	0.999181634	1.066434598	0.951472041	1.171236469	1.003584321	0.924409666	1.145928047
Q8BXA5	1.278373239	1.12599102	1.15577924	0.902569704	0.878871893	0.922538198	0.884684665	1.037836608	0.964320702	1.183937125	0.908848555	0.891503618
P97855	0.960449145	0.996174788	1.064985628	0.965653494	0.927639855	0.959979729	1.03665215	1.11593294	1.021237118	0.98507701	0.97930862	0.973188501

P25911	1.005288193	1.145863891	0.989879887	0.855194464	0.891185987	0.781885988	0.992657582	1.160297697	1.251706729	0.942287604	1.031415978	0.87084736
Q8BJ03	1.046393051	1.050672522	0.972744659	0.960093442	1.035206789	1.04142798	0.999912306	0.945726226	0.998017849	1.109429314	0.962959127	0.965135873
Q9ET26	1.112535221	0.994636123	0.962000709	0.970162085	0.963107805	1.073848358	1.10681857	0.996025391	1.012823012	1.001616246	0.914640324	0.932503296
Q9WVH9	0.906829501	0.857777905	0.91045257	1.254178959	0.879673782	0.893318239	0.890329744	0.988338441	0.97779883	1.647476941	1.062585008	1.032327832
Q62203	1.045997452	0.993417735	1.022802544	0.980427692	0.9923425	0.975111203	1.018087411	1.06095522	0.986358496	1.02298037	0.938608414	1.004695412
E1U8D0	1.110618942	1.015689616	1.023402053	1.073818579	1.12715452	1.020062427	0.929921337	1.000714693	0.974162778	1.003620315	0.92301964	0.86967775
O88874	0.992852102	1.029124318	0.972290087	1.020634538	1.00304323	0.991853547	1.042071241	1.018571806	0.990876774	1.115443016	0.953093176	0.947500794
Q5SZV5	0.845191569	1.080051741	0.987431443	0.95300686	1.041957059	0.987445336	1.080543606	0.989784389	1.009428963	1.065543765	1.006762961	0.972511535
Q91W96	1.000242094	1.112619094	0.924954098	0.846810357	0.961092109	1.160606426	1.032854706	0.938063563	1.044365915	1.405294676	0.900771389	0.869005107
O08581	0.789284209	1.05672824	1.050331115	1.112184072	0.724511474	0.8316648	1.014081213	1.198961213	1.031367455	1.249376134	1.107424271	0.873927056
S4R2P9	0.93732859	1.023575732	0.996102416	1.035032096	1.043997922	1.034593455	0.997301674	0.98157489	0.973900702	0.934277457	1.013058457	1.029263584
Q8CBG9	0.950130368	1.040743154	1.010411016	0.965286609	0.92630499	0.942584859	1.049030336	1.118889893	1.075707438	0.87082361	1.018727646	0.955900561
Q99ME9	1.051455384	1.02727114	1.011446626	0.954822621	0.992514057	1.044341419	1.002561873	0.975330147	0.981213791	1.009711577	1.026731479	0.951236063
Q8VE11	1.00337212	0.998761977	0.935668007	0.993197002	0.90320602	1.027663617	1.017033399	1.024769407	1.001968308	0.963415811	1.090084939	1.030922082
Q9JHW4	0.975301514	1.032316442	1.043342927	1.107883191	1.088895914	1.093518526	0.888812873	0.95826259	1.004482362	0.767409319	0.99423317	0.998803039
Q5FWH2	0.83631031	0.98241188	0.959476775	0.979213823	1.011021193	0.972828391	1.077336219	0.982408812	1.04934613	1.015128806	1.078025435	1.039207523
Q8BZH4	0.915626492	1.190710613	0.932821253	1.053427315	0.958448242	1.036485922	1.047904067	1.06649438	0.867032984	1.090230768	0.997292476	0.913067511
Q9QZN1	0.959298475	0.962064661	1.057596655	1.01708125	1.011272589	0.965523802	0.928276863	1.031661869	1.029424389	0.919638204	1.055684167	1.034935299
P36895	0.951692524	0.995755952	0.967439884	0.960155796	0.965235187	1.016665297	1.078925661	1.076280958	1.003866501	1.2066902	0.943357418	0.92792077
Q8R0G7	1.079092204	1.06887761	1.062355678	0.936935464	0.989421665	0.88862809	0.981327036	1.115088671	1.04546203	0.957187826	0.883173744	1.003904891
Q8VDP3	1.019805831	1.010146552	0.995466579	0.978191527	0.973307878	0.974606879	0.969374266	0.987047118	1.011582945	1.043698333	1.045195879	1.027488401
P49935	1.053437719	0.961840642	1.010079573	0.893492652	0.975524787	0.932687528	0.969103051	1.06389312	1.270011574	1.102844108	0.891529875	0.908806936

P35951	0.9738609	1.083049929	0.999895553	0.969611084	0.938342216	0.921651801	0.957473801	1.001101347	1.067483763	0.8881467	1.152554793	0.978878556
Q4U2R1	0.995618339	1.007336436	1.02775696	0.955886182	1.004147305	0.868383555	1.058509915	1.088725021	0.995366055	1.068392303	1.07097796	0.870199144
Q8CAK1	1.008581209	0.906784758	1.169967887	1.016100573	0.92959952	1.092707692	0.940706053	1.087853364	0.960357207	0.887627838	0.939863824	1.041873924
O08644	1.201379854	1.190577216	1.031710373	0.965566937	0.96280524	0.936355313	1.002251352	0.940850859	0.985703816	0.928754309	0.952803783	0.928056055
Q60805	0.990972493	1.044540224	1.019265621	0.986259014	0.950554434	0.991864936	1.074609723	0.983279163	1.005051281	0.956943449	1.009710216	0.977232201
Q62384	1.055224879	1.012715882	0.900779764	0.928177356	1.056875041	1.012556634	1.055085473	1.009983514	1.016547271	1.19085752	0.94691132	0.927684637
Q8C5P7	0.901664504	1.082673408	0.931679168	0.964850498	1.022638109	0.954465179	1.071982275	0.930385271	1.015517534	1.192507176	0.976612983	1.051269296
P52624	0.998279503	1.006301826	1.046859613	1.025867693	1.020122548	0.979546792	1.039923359	1.041235059	1.062948432	0.937155864	0.904953302	0.931789243
P41731	1.171465323	1.057747014	1.103252081	0.960184839	1.103788951	1.039947498	1.008779953	0.977112982	0.931845064	0.947193083	0.892664054	0.851491515
O08989	0.896498844	0.94170375	0.911170439	1.004430403	1.057293515	1.053874966	1.018463353	1.077707064	0.993959771	1.073680506	1.082379575	0.915170677
Q8BP00	1.003727069	0.958764282	1.037541225	1.061345186	1.010851969	0.986678221	0.986415336	1.043411543	0.988799493	0.950737657	0.979823802	1.001218887
Q9CXJ1	1.009759974	0.951044841	0.966014016	0.924011989	0.942045914	1.015711846	0.991539126	1.037548433	1.077569886	1.24042364	0.965112496	0.988682283
P29351	1.014323021	1.067220509	0.998340366	1.058089584	1.134050013	0.805519961	1.071902324	1.000979295	1.079620396	0.923096279	0.995960744	0.826611881
Q9CQ71	0.989518504	0.944919276	0.991549901	0.995738177	1.054629597	1.077271326	1.010945935	0.931645328	0.978120493	1.113943585	0.999174082	0.993948824
Q8K377	1.126116292	1.155388042	0.96434918	0.942953382	0.884380215	0.947266707	0.99826344	1.016967294	0.967782253	0.941105837	1.010229418	1.058285363
Q3UIA2	0.885414011	1.437287364	1.062743457	0.917394121	0.948207772	0.951817374	0.979084518	0.92209512	0.904981498	1.076283646	1.005721368	0.960041035
Q8CB27	0.992651129	0.971781819	0.980842456	1.078907916	0.925299462	0.94763424	1.034005055	1.073516767	1.077496528	0.963434867	0.976538491	0.97051922
Q9CQV4	1.041739684	1.013417172	0.927911104	0.954689112	1.086287819	1.0476752	0.939540248	0.945277163	0.984814781	1.107616546	1.002666491	1.044023999
Q8C079	1.028301663	1.01378396	1.061348968	1.027605001	0.964937535	0.970454913	1.016227732	1.019164215	1.019698974	0.857805435	0.990999679	0.988568328
Q78T81	1.173787686	1.117875296	0.972056563	0.938492715	0.600848238	0.930910065	1.171384349	0.887573907	1.485513553	0.671800114	0.801239957	1.09415534
O54784	1.001719851	0.853738533	0.938360307	1.040559332	0.939750645	0.968459568	1.168614782	1.071108789	1.15048643	1.140834448	1.039233438	0.70686863
Q8BTR5	1.010809338	1.018129469	1.082858945	0.98929863	0.91019407	0.962766125	1.004975583	1.047477021	1.062958239	0.936099573	0.971960033	0.978926803

Q9R062	1.022543128	1.031758802	1.056276421	0.933206745	0.922137178	0.972579353	1.041982393	0.956228655	1.134047308	0.98100423	1.004134695	0.923239359
Q8VE88	1.021091407	1.015875629	1.060265149	0.937160559	0.959135733	0.914459757	1.06343661	1.010924074	1.105135251	0.977430113	0.980157342	0.935344456
P49138	1.089706857	0.990684257	0.925123435	0.959399896	0.907093421	1.075155726	1.070890637	1.013603345	1.033834623	1.063275619	1.022702199	0.887930732
Q9DCU6	1.027380118	0.981836974	1.068775364	1.040647484	0.977250533	1.084390858	0.978296779	0.988398666	0.986662303	0.923728454	0.958966117	0.991018862
Q9QYF1	1.017185138	1.009521964	0.975891929	1.023785614	0.945349679	0.981606134	1.050318855	1.071785135	0.954089003	1.040259898	0.97979413	0.993796538
Q8BW41	1.052985626	1.009197597	1.006508978	0.995119023	0.972927133	1.027062502	0.978493689	1.039054511	0.974509269	0.929252167	0.981522481	1.041736038
Q91WE1	0.9664302	1.054382958	0.995440692	0.978561652	1.037034429	0.991619858	1.049977917	1.025433916	0.969613014	1.046850317	0.984502412	0.938088153
Q8K479	1.120794343	1.087273392	1.057843207	1.214980116	1.208857914	1.145474309	0.862782195	0.927352737	0.818463706	0.877605009	0.902082231	0.855679973
Q9JJF0	0.95144738	0.97901349	1.32201237	1.043748979	1.050676606	1.081242233	0.805113258	0.956507884	1.03892356	0.592079619	1.046852667	0.971187728
Q8VEB4	1.119055572	1.01306336	0.876711164	0.889696333	1.055064662	1.14557095	0.897932838	0.930986922	1.121028303	1.338556692	0.896108018	0.916617642
Q9JK81	1.115416366	1.095779727	0.946560098	1.036598042	1.078173807	1.103907092	0.923374214	1.082069927	0.922709336	1.110433786	0.808511525	0.923244629
Q91WF7	1.037725945	1.046260266	1.036395273	0.995314426	1.079655853	1.009867946	0.977277209	1.033980745	0.922359155	1.091197577	1.003383432	0.839235834
Q8BZ05	0.77824966	1.514892679	0.815700101	1.140803702	0.871519303	1.005070213	0.983792902	0.995476979	0.853855772	1.13332956	1.117080706	0.866230769
Q80Y55	1.112052147	1.069229235	1.041395615	1.117284857	1.203907739	1.081105871	0.91231057	0.919373508	0.907386257	1.007456894	0.896660076	0.836198356
Q99J95	0.937708678	1.112862595	1.062228397	1.066585571	0.837966337	0.845291238	0.939166086	1.128870595	1.059361285	0.86571909	1.041072592	1.033939251
Q99N87	1.015960275	0.952787404	0.981776826	1.001870348	1.024647426	1.032745471	1.06387221	1.011347124	1.026696026	0.974733573	0.980300969	0.941872076
A2RSQ0	0.977386974	0.978067821	0.93624334	0.975153089	0.950264794	0.995044006	1.025069589	0.967735186	1.037694848	1.122325754	1.047566925	1.045872894
Q8CB77	0.925860025	0.998483638	0.99492066	0.997929968	1.104146491	1.020312494	1.044877225	1.083916787	0.942473248	1.125660006	0.977769996	0.851962314
Q9JMA2	0.967004114	1.072131309	0.966841216	0.936678241	1.121669493	0.945322619	0.949728604	1.082709175	1.046434471	0.953103214	1.028961676	0.91570172
P97314	1.165168555	1.041625107	1.061194747	1.104275667	0.860157778	0.976990278	0.890498617	0.92189656	1.037400693	0.847184083	1.018195995	1.064989868
Q8BVU5	0.962637828	0.966979717	0.992529184	1.036318128	0.987101921	0.983099439	0.957735997	0.990933386	1.002726806	0.965675448	1.058565025	1.100122418
Q9CPV9	0.894976443	0.93590482	0.924803751	0.991066033	0.917305524	0.990843049	1.037500623	1.000058221	1.057785505	1.007275743	1.083525783	1.149597575

Q61103	1.010397828	1.162557289	1.034052187	0.992201876	0.866125264	0.923316983	1.003637493	1.044302969	1.027598883	1.051914657	0.981312139	0.931998171
Q9JMG3	1.079152457	1.026232107	1.022365323	1.006314536	0.965666713	1.063894396	0.963711699	1.006258956	0.995837324	0.974560684	0.93718215	0.997270721
P06327	2.655223901	2.148142253	1.845547666	0.726717132	0.57753498	0.557235737	0.60002785	0.872676666	0.667621962	0.500406045	0.468414825	0.484468743
Q9CQ06	1.02373954	1.039967192	1.075124883	0.990823685	1.047382289	0.95817481	0.977059659	0.999274875	0.937083246	1.083815919	0.962385789	0.979799607
Q9D1M0	0.889693664	1.005310577	0.99138219	1.037702203	1.108688558	0.88357575	0.97279914	1.030187403	1.009050006	0.82563651	1.158222656	1.000977764
Q78PG9	0.961341485	0.961421937	0.984567865	0.950536161	0.939608854	1.01072984	1.059179161	0.993850389	1.030219705	1.102185458	0.998965769	1.054131739
Q0V8T9	0.884484904	1.02326183	1.054080484	1.027452035	0.977556622	0.918007379	0.999197702	1.066154621	1.035939196	1.090503826	1.068674634	0.866931568
Q9JI11	1.079708714	0.978319763	1.045223139	0.815848956	0.921737719	0.970777751	1.01321474	0.920364069	1.130398246	1.097774142	1.054869465	0.995938129
Q9CXI3	0.948010036	1.03392034	1.014700854	1.010882758	1.032833713	0.947251668	1.000130039	1.006733785	0.920765623	1.084800593	1.086699536	0.959375443
Q9JII5	0.874846361	0.944171128	0.974062793	1.002233437	1.000268896	0.878621974	0.94804344	1.005156162	1.056976615	0.872716472	1.163402919	1.206135033
Q9CW46	1.038963237	0.925107832	0.962600265	0.962950507	1.02293358	1.006079715	1.045567511	1.012105086	1.012415191	1.071707464	1.018193846	0.96773242
Q923D5	0.900690527	0.930820754	0.946510728	0.945667026	1.047202681	0.970294267	1.060743078	1.028840464	1.035039561	1.143032182	1.027174076	1.015864507
P21952	1.252114236	1.170474937	1.117145741	1.013985481	1.048174231	0.91844633	0.969237606	1.000792908	0.912996008	0.942264315	0.885989563	0.826422771
P09925	1.10245099	1.040307436	1.017738995	0.932645914	0.986059744	1.052518673	1.123570422	1.008517836	1.009581367	1.065168605	0.858465443	0.867914297
Q9DBH0	0.999004726	1.029307901	1.009615351	1.030090517	1.070899206	1.011364645	1.010725476	0.988762572	0.971890242	0.95852816	0.997900975	0.936749183
P55772	0.934009268	1.018599865	1.046555678	0.979331043	0.979625192	0.987783956	1.024504786	1.028058267	0.982030456	1.138771236	0.985229744	0.960602067
Q8CD92	1.092248032	1.091281191	0.884262218	0.919593727	1.05040789	1.084570873	0.969452924	0.827180484	1.019128028	1.339886862	0.960191346	0.96652311
Q8CI78	0.96945958	0.965501436	1.025470024	0.997237618	0.909784287	0.97030088	1.11879081	1.02755882	1.042780571	1.059176782	0.926581444	1.01694984
P56818	1.053857372	1.108307903	1.009579453	0.853787279	0.892626679	0.901392629	1.16851408	1.215428027	0.898676123	1.139891009	0.963654704	0.845165989
Q8CGQ8	1.026951728	0.937071954	0.937153773	0.878067882	0.921288878	1.113551534	1.216132795	1.063747798	0.995482383	1.343749274	0.843356593	0.884118243
Q9Z2G9	0.914918044	0.985242973	0.956714481	0.890636411	0.985129224	0.938403725	0.962283796	0.933722878	0.929701965	2.095293675	0.982785475	0.899720835
Q9Z0Z4	1.012810704	1.133796624	1.049340643	0.934415895	0.961212679	0.979858538	0.977047521	1.101654956	0.966449247	1.09590835	0.970908393	0.871576989

P60762	1.016613979	1.070748077	1.034410773	0.983386392	0.942676578	0.900980668	1.023259173	1.023723319	1.004727646	1.035283634	1.011075424	0.976740959
Q9WTS5	0.933649316	0.942724303	0.991316495	1.022430786	0.934549804	1.015166819	0.979220989	1.021269814	1.059895301	0.975292798	1.045907242	1.067846624
Q8CIM7	1.125475182	1.192083965	1.064037023	1.044606251	1.278269022	1.004879244	0.906786331	0.926743703	0.854695812	0.962472965	0.928688161	0.794642854
Q8BHS6	0.980549891	0.982544865	0.922512352	0.982550259	1.00973376	0.955975801	0.996057672	1.016950119	1.084011962	1.017082168	1.039561881	1.024447708
Q9JHK5	1.057453983	0.991077034	1.065318129	0.967311006	0.995204711	0.921919254	0.985090783	1.029810285	1.01030216	0.966474304	1.019673509	0.990573318
Q8R3D1	0.968580305	0.985449874	1.012102274	0.987297388	1.056456609	0.980825484	0.957641284	0.986610995	0.927841261	1.024610662	1.093749661	1.049459626
Q9D9E0	1.191598499	1.014945643	0.92407628	0.988058834	0.980904586	0.931867488	0.966316564	1.044770147	0.983171653	0.977744078	1.010538214	1.029715947
Q9CQB5	0.957486958	1.208121602	0.974074402	1.078117161	1.025865231	0.831603311	0.882970313	0.994110369	1.019176253	0.807882601	1.060717764	1.10990903
Q9D1F4	0.959310511	0.993853054	0.976608226	1.016266566	1.016779847	1.039108682	1.044128789	0.983054718	0.976646792	1.016947197	1.027671888	0.974585008
Q8CDM8	0.969571233	0.960381634	0.997214073	0.975526587	0.988366938	0.965083268	1.010298834	1.043683507	0.977600955	1.106359959	1.042695863	1.014374957
Q62186	0.93963893	0.992096715	0.952260918	1.008067221	0.990274365	1.01530202	0.99592017	1.010348737	1.054932656	1.040287189	1.03200567	0.9908092
Q60928	1.251253834	1.250755837	1.040570669	0.926110445	0.954694103	0.974080009	0.98492564	0.976315419	1.100120519	1.060743998	0.769386765	0.807632856
Q922B9	1.116406207	1.136970458	1.080583068	0.859470401	0.85405102	0.78205901	1.115335462	1.180416448	1.138530521	0.974111297	0.908404467	0.814659687
Q9Z1S3	1.101439513	1.128269102	0.962868696	1.033768345	1.122613858	1.032863184	0.938179011	0.906966543	0.890190976	1.077430253	0.945710464	0.983661641
Q9EPK2	1.034058442	0.994049582	0.986233766	0.977403551	0.97669514	1.033435572	1.019981037	0.920048218	1.020572638	0.95500773	0.968052843	1.135393254
Q8BGX3	0.989088335	1.04096729	1.000738769	0.96916314	1.025972648	0.923552591	0.987683432	1.068563127	1.038629394	0.956230068	1.040839069	0.940059254
Q6PD24	1.054747909	0.986277451	1.043826505	0.99693285	0.987497421	0.972145579	0.966660754	0.96434739	0.979177854	1.029571655	1.035953176	1.023950252
Q8BFQ4	1.019405452	0.938396272	0.997111555	0.981623271	0.930155117	0.978359607	1.023490095	1.036137612	1.024084698	1.025367966	0.992594874	1.079207436
Q80UP8	1.11242904	1.114943791	1.075316462	0.967879683	0.998573373	0.917591859	0.986988208	1.008487293	0.968570071	0.997479108	0.970094682	0.917250682
Q8BKR5	0.876120066	0.983923588	1.011612261	0.848460968	0.943757879	1.051229535	1.207573319	1.069603245	0.865975723	1.05840209	1.035930739	1.052942645
Q07113	0.895551512	0.913404735	1.038272272	1.077402843	1.018866856	0.917961646	0.991536565	1.025133498	1.010403235	0.821419193	1.134022205	1.068216135
Q6WKZ8	0.971777753	1.070648259	0.942128696	1.040056062	0.996352009	0.928693034	0.926939716	1.022128776	0.905630657	1.033347328	1.221575638	0.958505614

Q9D074	0.824449845	0.989236614	0.941185536	0.961389207	1.048792432	1.017437327	1.024121955	0.993703349	0.999107266	1.27335775	1.037776096	0.998594615
Q9DBY0	0.949010072	0.995151373	0.863346678	0.980512017	1.019084183	1.014574739	1.060992145	1.017163621	1.130612141	1.161678465	0.904657808	0.987994453
Q8R1V4	1.018510805	0.955848905	0.998253033	0.957801313	0.957581579	0.925699203	1.01449182	0.996325397	0.939656445	1.234471762	1.087616794	1.020394952
P62313	0.950939723	0.975887181	1.005734487	1.004588966	0.923893142	0.999849248	1.059349555	0.976018526	1.013829773	0.988665947	1.050347611	1.045329243
Q6A026	1.01740423	0.906114461	0.926272765	0.931026611	1.112403593	1.002366296	1.073946314	1.024152375	0.938905439	1.121453911	0.9972988	1.030969257
Q3UQN2	1.045156962	0.939437991	0.958467661	1.177289242	0.997914531	1.148209514	0.96834802	0.932825067	0.95805913	0.99173711	0.970336503	0.980010731
Q9D2H5	0.916277745	0.957964785	0.978548401	1.073936478	1.037186152	1.030929592	1.016734703	1.047947277	0.987819273	0.978236837	0.981061025	1.007992014
Q810B7	0.855616075	0.94768322	1.034404906	0.921508424	0.912742561	0.859661601	1.020252001	1.032669645	1.147605118	1.002759962	1.117506128	1.089173119
Q61112	1.088625502	1.032142107	1.026389004	0.989583823	0.999298881	1.086122445	1.01478559	0.989518633	1.011440234	0.913184104	0.91553893	0.944145504
Q8K3G5	1.174265504	1.183418077	1.07657354	0.834823376	1.043481016	0.736808411	0.926036149	1.123608277	0.869580353	1.262512256	1.054427729	0.847725753
Q8BHH2	1.096845267	0.979764421	1.107584379	0.95041735	1.014376935	0.979478789	0.946939618	0.970482227	1.01137527	1.032236843	0.983722208	0.971076331
Q9D8B1	1.076401751	0.94095471	1.014321556	1.039119457	0.994122978	1.02500149	0.989197781	0.996274523	0.970103628	0.953375809	1.014507282	1.00629662
Q3V384	0.915513226	0.981578167	0.960288959	0.984711555	0.996422226	1.021137898	1.025045269	1.005512778	1.014429858	0.974358424	1.072153363	1.035957147
Q91WM3	1.013881612	0.99898667	1.055289457	1.001557124	1.063794846	1.009085049	0.957555541	1.024790276	0.922722698	1.117709521	0.975518326	0.94922574
Q9WV95	1.039111087	1.105162801	1.180734892	1.040249795	1.117107151	0.921613541	0.833312408	1.004434728	1.021897206	0.729077856	0.964996358	0.972404665
P50153	1.0063927	0.97931219	1.00483617	1.033943102	0.861886112	0.948124486	0.982503268	0.971738871	1.129947544	0.889651559	1.043540813	1.100785902
Q66JZ4	0.936624652	1.01939465	1.014368577	0.961462237	1.004772958	1.005246813	0.983002477	1.033247685	0.940247323	1.015904728	1.039981644	1.067613266
Q8VCA8	1.061413353	0.975315235	1.039860665	1.033880516	1.06787364	1.075611882	0.976909085	0.974017308	0.93408755	0.968358362	0.967527761	0.968148874
Q9JI78	1.00557876	1.031213144	1.034239722	1.017609174	0.961394527	1.002781098	0.969119147	0.971350077	0.963115046	1.037594124	1.005996142	1.049159056
Q3UUF8	1.08478285	1.015363317	1.009132834	0.985580029	0.919806754	1.028721027	0.971237419	1.005408023	1.001911341	0.995458272	0.967380738	1.053650075
Q9WTK3	0.981477036	0.972664316	0.993754827	0.989849533	0.94597198	0.978106516	1.042846525	1.010419639	1.01351785	1.001594714	1.023024602	1.055273232
O35972	0.997385679	0.995424109	1.001445356	0.951717485	1.082837459	1.016545131	1.020186704	1.033655729	0.990297532	1.236696322	0.949959663	0.845511473

O70480	0.883473372	0.988511401	0.974716172	1.021186549	1.020178163	1.023379544	0.982223484	1.086005729	0.95096493	0.954899295	1.095130693	0.999718488
Q9Z108	0.995804272	0.932494339	0.99446876	0.971545577	0.893165875	1.029621004	1.038027165	0.987026582	1.26591777	0.898021673	0.877885744	1.072007268
Q6NS65	0.667924027	0.666720183	0.730319833	0.926897978	0.754439036	0.719433577	1.028623883	1.280729227	1.286665818	0.819519219	1.466491045	1.408420621
Q8R1K1	0.916282171	0.955127356	0.989313819	1.068460059	1.237319633	1.073163611	1.013941066	0.924490185	0.891721391	1.031143608	0.988732193	0.978736877
Q8BGK5	1.00794008	0.919130994	1.074435386	0.999727684	1.087075068	1.075607167	0.955104918	0.946962757	0.975182633	1.051776141	0.981458504	0.986528611
Q9Z0W3	1.019444234	0.973669526	0.99516826	0.985500404	1.100824636	1.007571693	0.987494605	1.008429542	0.921292443	1.058792758	1.018196216	0.987073467
Q9CY57	1.022057213	1.026904402	1.05701747	0.963719302	0.925459745	0.986281729	0.932680991	1.104533735	0.908397197	1.098492447	1.084515191	0.940196397
Q8VEK2	0.95087993	0.978646601	1.048320919	1.046067288	1.007831045	1.026363061	0.974735653	1.024551408	0.946758116	0.939072151	1.051545114	0.997446744
Q64327	1.039130988	0.964969968	0.827777692	0.902437861	1.089526708	1.177462804	0.854900621	0.926195707	1.115163529	1.834516541	0.818970995	0.868724916
Q8BXK9	1.236081806	1.146058162	1.071438608	1.155006419	1.184630163	1.247395011	0.86888051	0.784293963	0.77390898	0.714411647	0.890456972	0.974814549
P46662	0.96415186	1.049035741	0.904180206	0.911438574	0.989457282	0.905644206	1.072607098	1.13320239	1.071689435	0.830105589	1.009236411	1.078966828
Q924Z4	1.106792763	1.093163857	0.976089386	0.885567707	0.96194513	1.286162202	0.869595382	0.980590972	1.15805162	0.971845573	0.880391757	0.863932679
Q91WN1	0.950380707	0.998604669	0.954353933	1.052254668	1.133693376	1.08717891	0.95214581	0.966504971	0.944763593	1.097800641	0.999463684	0.952369021
Q8K4F5	1.076785629	0.946830084	0.994858753	0.911616327	0.931806912	0.946267004	1.094405299	1.052978949	1.068772037	0.928283855	1.001065693	1.013622782
Q9QZN4	1.106348198	1.307796371	0.913438609	0.92233196	1.04272427	1.005977989	0.936309291	0.950909167	0.98014393	1.276524219	0.904393488	0.837335286
P50429	1.035072605	1.015705482	0.98072833	1.053759646	1.017098785	0.939343745	0.927827261	1.01255056	1.052332554	0.85108779	1.010389497	1.076122745
O35668	1.066037789	0.951407108	1.45923628	0.937261913	1.025685455	1.046668265	0.824567268	1.043377347	0.942801717	0.785473122	0.935616325	0.916861946
Q8K284	0.975439346	0.920421529	1.004092904	1.044656881	1.071919495	0.995599495	1.004719265	1.003312137	0.915981946	0.951331344	1.052951503	1.07088661
Q91VJ5	0.944723305	0.980208405	0.982346395	1.008857498	1.017417928	1.112371121	1.005781696	0.913416014	0.963797167	1.02970001	1.011129591	1.077867157
Q8BGR2	0.998230448	1.0386288	1.058374388	0.971106447	1.246598303	1.029777865	0.985880329	0.880009458	0.856853118	1.129031749	1.005068906	0.912261709
P97447	0.988896382	0.958689976	0.994435967	1.00716131	0.991635222	1.038284919	0.991019281	1.066910807	1.012881743	1.012188874	0.955384072	1.013817336
Q62415	0.889755296	0.85693804	0.978543477	1.018416675	1.031761347	1.086392534	1.034870622	0.968486083	1.021568548	1.051689064	1.005473662	1.090337648

A6H6A9	0.947734958	0.958048953	0.963342728	0.978926097	0.98411652	1.009178171	1.012408234	1.000779038	0.990158376	1.010125521	1.060760565	1.098286931
Q64471	1.124414513	1.068141307	1.118675939	1.077570789	1.016870936	1.143794129	0.920927858	0.913060537	0.92109279	0.822072028	0.941926952	0.936424199
P97789	0.979098747	1.051229856	0.951673832	0.944775903	1.026154241	1.063299937	0.981142673	0.987180205	1.007750239	1.272943891	0.915326127	0.972937946
Q8BHL8	1.054558571	1.043074143	1.015542787	0.996929341	1.073873131	1.003328942	0.975172469	0.953531512	0.967268779	1.065862446	0.973303931	0.952942954
Q8BG09	0.976610326	0.978982054	0.941032231	1.029637845	1.006170294	1.003764513	0.969947696	1.001868621	0.960934944	0.983812101	1.057636172	1.113751466
Q8QZY9	0.965034086	0.982738831	0.885788585	0.963878854	1.07039927	1.037677505	1.032502517	0.9714142	1.068963289	1.160040676	0.919385963	1.043456601
Q9D4F8	0.955190057	1.158594083	1.009906078	0.966895014	1.015389862	0.923742923	0.964053666	1.073352518	0.969827867	0.981201806	1.041775254	0.940211917
Q8K2F0	0.940219392	1.001410626	1.024589362	1.029185576	1.036249986	0.97808098	1.035644737	1.022110729	0.993479174	0.991339323	0.99209808	0.965643463
Q8K2V6	0.992586111	1.01218803	0.994834015	0.914214837	0.935057359	1.028291386	1.027857863	1.018388924	1.028161629	1.078777272	1.018778423	0.984297384
Q7TSS2	0.961213497	0.9348756	0.923606067	0.970883953	0.937194626	1.083850754	1.076012979	1.007541005	1.070850733	1.129274883	0.933467131	1.04029271
P56387	0.92331048	1.081499579	1.015213536	1.082552198	1.065693	0.953170966	0.944289058	0.933340236	1.087244414	0.704154729	1.072169011	1.023328075
Q9CY97	0.742886151	0.914726478	0.981182332	1.01330919	1.047629377	1.005829236	1.051563324	1.046315945	0.971854948	1.254457742	1.086310081	0.960995433
Q8R0F6	0.98843448	1.075625289	0.987524004	1.001220515	0.991207238	1.012571761	0.998479469	0.938919856	0.952631214	1.059963285	1.045013415	1.001397674
Q61554	1.100991373	1.259952823	1.240962028	0.807744276	0.772310589	0.784881932	0.88413173	1.247872665	1.24359686	0.837095705	0.902622028	0.803674042
Q9ESU6	0.929500216	0.966930944	1.016297164	0.99599128	1.2070973	0.940981902	0.939996419	0.998775769	0.921735503	1.262293883	1.035585245	0.922763254
Q920R0	1.001051951	0.956231714	1.021241139	0.989259948	1.069988749	1.006728624	1.00864316	0.956424407	0.989902079	1.013408045	1.019564625	0.997757329
O88307	0.902229172	1.032978639	1.026781575	0.906113207	1.226193526	0.883511597	1.037587779	1.044558036	1.059234117	1.171647984	0.880975839	0.911207727
Q8K093	0.910058054	1.323996415	0.904567116	0.887942401	0.8858932	0.861048626	1.082554584	1.025482801	0.994368305	1.035368783	1.136617124	0.934493912
Q8R1E7	1.023514613	1.013978684	0.992928113	0.961189674	0.969483215	0.959122939	0.987392245	1.028940835	1.038523487	0.994172286	1.021003462	1.018937101
Q8BGR9	1.107077322	0.991812804	1.081326809	1.011207188	1.055533918	1.108597632	1.026901992	0.944900816	0.994809631	0.867580024	0.874130939	0.943231652
Q99LG4	1.041070564	0.991140496	0.978457097	1.045986449	1.099057123	0.994866409	0.951948142	0.982863791	0.954461002	1.107663288	1.021028836	0.92164659
Q9Z2D8	0.943727264	0.926790541	0.810409133	0.951945943	1.28856367	0.925081953	0.937069158	1.003139419	0.947742533	1.280508453	0.925594493	1.252623686

Q6PIP5	1.036195942	0.885357204	1.037115954	0.873615602	0.929938052	1.060475693	1.011847553	1.065775863	1.066617105	1.091501594	1.014419698	0.954982932
Q8VD26	0.957702168	0.95638772	0.934389607	1.027553827	1.133535394	1.025275884	1.063815614	1.043165534	0.888494067	1.199030819	0.934015398	0.970115787
P62315	0.942306221	1.469238669	0.900821219	0.968790434	1.014083274	0.91192779	0.917583605	0.949494156	0.903924337	1.164147823	0.982693789	1.000308698
Q8K215	0.933375136	1.056360183	1.045463639	1.088338479	0.996738516	1.1247695	0.938975482	0.979047772	0.986964699	0.875389458	0.946114756	1.021402357
Q61818	0.984169813	1.032125117	1.029857078	0.970147292	0.969774829	0.836683284	1.007037029	1.084751251	0.963406852	1.173451678	1.04268005	0.977754758
Q6PHZ5	0.946092112	0.939140942	1.055754249	0.995415158	1.134956235	0.921926222	0.982475947	1.02037674	0.835135782	1.124321591	1.143620189	0.964534521
Q9CRT8	0.911921567	0.950892747	1.063269097	1.049539023	0.988806007	0.930736077	0.900239329	1.017854502	1.04832381	0.853955522	1.094969016	1.125925581
Q8CGA3	1.043050421	0.992635431	0.951681906	0.97295638	0.983147947	1.07581064	0.986393898	0.921252899	1.049643924	1.206852622	0.958983259	0.98207635
Q8R1G2	1.068186817	1.027615463	1.033278359	0.936964133	1.017102395	1.066074036	0.950549888	1.045221027	1.005411141	1.069904509	0.951172598	0.890809785
Q9D4H2	1.011556861	1.016646449	0.98214884	0.942725105	0.911960535	1.026956654	1.063482526	0.992868767	1.034812102	0.971512486	1.001813489	1.040227736
Q8K0V2	1.082889924	1.019313633	0.866340536	0.911059062	1.191936635	1.207097778	0.852285799	0.835658392	1.111343526	1.551308731	0.868775346	0.811499962
Q8BUV8	1.049270536	1.025354495	0.991460526	1.007370814	0.972740732	0.998023227	0.997375969	0.974877446	0.993257267	1.025825831	0.977977991	1.036429126
Q9DBE9	1.024693335	0.997484307	1.009930991	0.943606571	0.957804392	1.051614504	1.0581818	1.011238303	1.021466488	1.145196386	0.913101717	0.950576156
P97785	1.032618246	0.998607122	1.103007242	0.982388674	1.010701971	0.919957616	0.980201678	1.080456124	1.00959268	0.869379892	0.999646176	0.96911082
P22437	0.986987296	0.966963382	0.984360404	1.054366447	1.062127198	0.984110927	0.960473658	1.075968491	1.040321406	0.909950584	0.968746701	0.996619663
O70572	1.19732455	1.121974066	1.087523091	1.009518072	1.01438469	0.933978669	1.036426882	1.020799704	0.925856531	0.828817006	0.886812846	0.934513171
Q9EPL9	0.93404182	1.26292889	0.970140519	0.984479896	0.954947411	0.995727238	0.986105956	1.004942916	0.956485746	1.017778176	1.020917286	0.938325843
P02802	0.841779633	0.944472001	1.06554835	1.205479796	0.795383747	0.702915856	0.728850725	1.082669928	1.453458182	0.574347808	1.17607853	1.174892897
B9EHT4	1.026531185	1.062305307	1.060236261	0.997635922	0.956633315	0.922511322	1.003215463	1.068569771	1.089581259	0.882271659	0.936421388	0.955115139
Q61550	1.473878277	1.250076964	1.267769934	0.426845964	0.427908278	0.444334474	1.476597899	1.69945979	1.936702091	0.415630405	0.393248323	0.362776765
P50285	1.084967659	1.00944159	0.984833477	0.977595446	0.938840258	0.977693836	1.017715238	1.045738456	0.947824153	0.957029922	1.077119086	0.98141696
Q08943	0.976909011	1.011015006	0.931967893	1.017240372	1.057206076	1.035579026	0.973027277	0.95118499	1.005630585	1.009480028	1.05630377	1.005753354

Q9CR39	0.991995115	0.975280012	0.9948522	1.021445919	0.953233046	1.012156171	0.99198793	0.987704361	1.015221546	0.923412263	1.023796533	1.09884761
Q60759	0.931843466	1.097798102	0.976245538	0.986510147	1.054491715	1.00350558	0.975071174	0.907650417	0.924379151	1.009054624	1.101464283	1.060481791
Q9DCJ9	1.065751965	0.995919934	1.011834919	0.983044977	0.959524285	1.034556612	1.073941373	1.002558153	0.988682694	1.036543287	0.933160204	0.96348502
Q9R0C8	1.141248744	1.072282983	0.921098734	0.912757075	1.078557407	1.078991284	0.99111309	0.957772826	0.935287656	1.264545906	0.977026436	0.839059737
Q80V91	1.01223939	0.954378944	1.01647642	0.967652385	1.040647155	1.006572302	1.000191994	1.025770217	0.972737159	0.897310994	0.980730307	1.116212563
Q9QXQ1	0.916993444	0.991276591	0.990290949	1.074330179	1.056809897	1.034746337	0.952452247	1.003940238	0.93319279	0.990598396	1.060232596	1.019518973
Q922M7	1.000327181	0.987985293	0.995636987	0.994371007	1.058533515	1.027274405	0.986540795	0.959952577	1.022131258	1.064311595	0.985075361	0.97466314
Q9DC37	1.214121301	0.99367987	1.00601535	0.888149331	1.002472288	0.821309138	1.098285223	1.118540417	1.009560429	1.018000731	0.847104714	1.036012516
Q4VBD2	0.985934396	0.972323379	0.963474924	0.969020677	0.95906994	0.96100534	1.06828459	1.026290209	0.984272579	1.049733772	1.021248518	1.07070756
Q6ZWM4	0.992062621	0.949725276	0.995321279	1.000951913	1.00605456	1.013017015	1.018584325	1.001935376	1.001492771	0.965115599	1.021977902	1.037651082
Q8BYJ6	1.177028593	1.107244662	1.073832627	1.13848161	0.962229652	0.943042661	0.861756479	0.95074739	1.009554716	0.687679152	0.97716936	1.056129955
P16254	1.065935877	1.01911018	1.033142535	1.002155341	0.924714516	1.007314852	1.02977478	1.023124295	1.017096621	0.892001035	0.960948666	1.006873165
Q8BH10	0.962613775	0.965420527	0.932907646	0.979242065	0.999474781	0.926252499	1.002767358	1.208334299	1.004150071	0.925065333	1.053046618	1.003036231
P36552	0.928747886	1.050523024	0.983782402	1.01178924	1.109986743	1.006111739	0.886516871	1.059993848	0.944462252	0.90474439	1.089579804	1.006994243
P47867	0.943854525	1.129873042	1.076626836	0.913075429	0.902098781	0.950876671	1.067455861	1.067516436	1.122982599	0.904881484	0.9234812	0.937612981
Q3UZP4	1.029058652	1.112535613	1.098254337	1.047623949	1.110829781	1.084020759	0.902435249	0.983905199	0.912571738	0.969190341	0.838599887	0.986253014
Q9R1Q7	0.902686372	0.98666748	1.040139338	1.032813621	0.940957684	0.864837874	1.116220361	0.9687554	1.056584489	1.187458001	0.950966195	1.024777822
Q8BYM8	1.096879977	1.091051066	1.056515972	0.902448672	0.894807167	0.937599981	1.089441163	1.054805377	1.044290529	1.073144135	0.899802589	0.901847467
O88829	1.01968747	0.913270174	0.96299464	0.934801097	0.960190875	0.969836766	1.042472961	1.048883325	0.997882831	1.192173124	1.037426988	1.00448282
O35127	0.968313612	1.114884337	0.97823588	1.093682486	0.945894618	0.959935597	1.005672429	0.984088609	1.025655127	0.878496904	1.012489179	1.004556807
Q6P5H6	0.891393816	1.104192696	1.02384858	0.994897188	0.95498577	1.005502371	0.991256193	1.052155779	0.986895021	0.878758147	1.032313454	1.034912959
Q9D4C9	0.945792769	0.99041052	0.943432449	1.100213198	1.011911376	0.995585131	0.973591058	1.041056017	1.008162216	1.088089363	1.043422246	0.911675885

Q07456	1.153878911	1.233631212	1.051181601	0.833568989	0.769397957	0.872309766	1.30754862	1.091400499	0.968331665	1.061552874	0.865375892	0.815037549
Q8C650	1.060471953	1.036423717	0.973586101	0.926852091	0.956033857	0.93070289	1.003935517	1.022929485	0.947043247	1.066537112	1.156452473	0.944614982
F7BWT7	0.978930585	1.077821001	1.05954573	1.286922355	1.008393763	1.170923011	0.95776266	0.983885114	0.930959154	0.803119733	0.841909971	0.908437336
P0C913	0.939027805	0.997050768	1.001155594	0.881519045	1.036702199	0.954000222	1.083511389	1.017053532	0.962374853	1.042692479	1.078694706	1.011565081
P62274	1.006264062	1.082439067	1.035354301	1.070120806	0.759158401	1.047270415	1.140488901	1.097589686	1.139717365	0.608206895	0.845562227	1.010768584
Q8BGG7	1.018759554	1.007896877	1.027284648	1.097202943	0.989362654	1.065116322	0.968388073	1.008109685	0.965592481	0.909382812	0.97181592	0.979983947
Q8R1Y2	0.856589354	0.958971202	1.066081007	1.066104915	0.973700076	0.856806832	0.916918992	0.9960152	1.095901188	0.870976916	1.130868601	1.131893901
Q91W89	1.019593961	1.116125601	1.039770979	1.105067022	1.061569544	0.977375055	0.968106686	0.963624077	0.952330112	0.931962028	0.969827972	0.918886262
Q8BGT1	0.937246394	0.997065473	1.045199557	0.966901931	1.103492139	0.942401089	0.957002333	1.033346852	0.934040936	1.146873781	1.06506013	0.942021971
Q8K2F8	0.799523607	0.929190052	1.007142934	1.046015686	1.020403755	1.008462053	1.032251448	0.982744124	1.040200615	1.241502711	0.987376762	1.00042128
P48542	0.901295817	0.952964551	0.945221365	0.907500161	0.901390656	0.943740127	1.138890685	1.014018308	1.088055979	1.125739728	0.980677141	1.132653915
Q811S7	1.038128622	1.065839024	0.962453698	0.964810085	0.9374469	0.914618309	1.163477632	1.120729937	0.94117424	1.084358083	0.956087499	0.897007169
Q80TS7	1.065827716	1.291412702	0.884852209	0.819764617	1.041045988	0.931676076	0.956848748	0.941844668	0.987694836	1.513474505	0.941556304	0.883948455
Q9D0D3	0.911725693	0.934983724	0.975034683	1.045709363	1.058174055	1.027440092	0.925210374	0.950812362	1.008913916	0.993272314	1.110005951	1.069193448
Q8BWU5	1.003818119	1.017530856	1.002609701	1.023871867	1.02179821	1.010485586	0.993363145	0.984990176	0.988653089	0.995393204	1.014038015	0.969151587
Q6DFY8	1.021848965	0.993187513	0.951465395	0.931734856	0.902955251	0.922490693	1.015470879	1.0087053	1.039753861	1.072338289	1.087581413	1.077993784
Q9Z0S9	0.941676106	1.001273986	1.005571974	0.974351279	1.007952847	1.013012869	1.008591586	0.984870402	1.013578041	0.961920674	1.029685939	1.050923666
Q05A62	0.983990359	1.010274616	1.003242556	0.995280006	0.917041485	0.988412744	0.984439041	1.031012073	1.026356266	0.986821233	1.031733195	1.04356072
B9EJ80	0.984643995	0.999124886	0.937024724	0.969296505	0.955596088	0.896975611	1.06357922	1.147672901	1.051203444	0.97698134	1.000362458	1.001447648
Q3THJ3	0.983273149	0.965825879	1.006116412	0.970091014	1.032172519	0.929006867	1.017748	1.013202735	0.967008486	1.173519612	1.039161719	0.986318953
Q8VHQ4	0.955647738	0.988499543	0.940467619	0.924146148	1.005368101	0.98847057	0.950744861	1.009110002	1.010026101	1.127302715	1.065112644	1.098250737
Q8R1C3	0.957017638	1.11539705	1.110005861	1.12958945	0.747639521	0.682450702	0.75115393	1.042707074	1.282901888	0.507940765	1.163667295	1.265168698

O88492	1.009510035	1.030667322	1.01650453	1.031635868	1.065305417	1.071760232	0.953647853	0.983188281	0.953008062	0.938271525	1.000422044	0.964787445
Q9CQF0	1.007590874	0.921452134	0.946573212	0.956537657	1.08101656	0.971880765	1.087991955	1.053374859	0.992314625	1.010239445	0.993867576	0.998139068
O08545	0.947601423	1.020777093	0.922937006	0.981836413	0.928779382	0.994584705	1.072483594	0.993131588	1.055048101	1.148997915	0.97251206	1.032237007
Q91YQ3	0.975983433	0.954101051	0.981610515	1.024932018	1.004802215	1.023785284	0.996033121	0.987941976	0.948680011	1.101313618	1.041225469	1.028479543
Q8BH69	0.962991405	1.024943445	0.984851821	1.014853042	0.994776319	0.938738869	1.024476544	1.067540673	0.997040876	1.027678604	1.01411354	0.968144312
Q9D428	0.937581628	0.966146696	0.950083938	0.952871616	0.908091523	0.973211282	1.084694418	1.07080497	1.072159099	1.055940224	0.974483523	1.069186633
Q8CFJ9	1.005283944	1.024025988	1.006141945	0.974761336	0.96430827	0.97297938	1.041808968	1.086319266	1.012511182	0.969610127	0.982513927	0.955561691
Q3TY86	1.032279251	0.993488164	0.979361547	0.962002783	0.942750721	0.992260173	1.048193133	1.004470209	1.018377274	1.058435853	0.988474622	1.022164994
A2BH40	1.042392416	0.950377971	0.977315243	0.945233303	1.06405981	0.961027926	1.016801943	0.94316388	1.015779	1.246812898	0.992856321	0.974357471
Q7TSG1	0.986711826	1.008715385	1.039592113	0.752629881	0.996431445	0.868295031	0.984510836	1.028566425	1.052721378	1.209783562	1.089490355	1.03881643
Q8R4V2	1.166720266	1.075391626	1.074581277	0.913515835	1.201515523	1.018923903	0.978013869	0.950567422	1.036409494	0.844400579	0.923599241	0.803436508
Q5XKN4	0.933844382	1.130689972	1.008822269	1.074304841	0.997846549	0.951095255	0.92891007	1.007972256	0.907572209	1.059920848	1.089563079	0.957830185
Q2YDW2	1.037925926	1.045222616	1.0838724	0.933580751	0.998453224	0.971519795	1.067400695	0.953198445	0.980649751	0.9131899	0.955983654	1.049102648
Q9CPZ8	0.928700356	1.161312734	1.032338004	1.20214859	0.896466917	0.906768833	0.819664357	0.937000527	1.132308561	0.725852563	1.005561274	1.171430574
Q99J09	1.263107151	1.019845921	1.163218651	0.966931174	1.084480514	1.077646297	0.942480247	1.00604015	0.984085542	0.728177563	0.896645444	0.824362467
Q9R1Z7	0.905096212	0.930224453	0.980293727	0.976837982	0.970765024	1.052603164	1.041062703	1.073508589	0.956515739	1.005698361	1.006965685	1.112835615
Q8BUY9	1.180050772	1.094990707	0.947223152	0.898889696	1.109931683	1.164277203	0.937224451	0.927898212	1.021617155	1.088475707	0.88549087	0.859993091
P56213	1.148078581	1.06381682	1.004984097	1.037795735	1.054637216	1.031866491	0.911525738	1.055156528	0.934572319	0.925907379	0.997885713	0.863684279
B1AS29	0.913865369	1.004123571	0.956627491	1.03460873	0.986713612	1.043566318	1.033892327	0.992838017	0.974605332	0.970247329	1.032682915	1.061082258
P35294	0.998972228	1.064520147	1.080200253	1.078875221	0.954398743	0.917189846	0.871480946	1.021426173	1.024891315	0.894008617	1.060279926	1.00680252
Q61285	1.073070829	1.040641745	0.881392493	0.951993252	1.174528307	0.995975204	0.929997455	0.97573115	1.028688924	1.127320516	1.015788874	0.903695987
Q5PR69	1.002150556	1.042684876	1.076160669	0.994518614	0.974725421	0.98371927	1.007707078	1.026178215	0.961981105	0.912588348	1.028676439	0.965437894

Q5SVQ0	1.05485368	1.085989564	1.062915302	0.906333281	1.039966264	0.89237504	0.928031427	1.033445926	0.953123528	1.209255408	1.013334123	0.931351198
Q8BFY6	0.97755892	1.020761795	1.083789014	1.087041864	1.087202884	0.968113103	0.929536634	0.894062381	0.984507793	0.859002592	1.047682107	1.038269046
Q91YR5	0.952197522	1.02281322	1.018220773	1.007931878	1.057609954	0.925164034	1.029086952	1.003320564	0.956922286	1.063999211	1.041719198	0.959239784
P00416	0.932653706	0.93611661	0.99284595	1.038580539	1.055331345	1.007337143	0.980141061	1.005973027	1.026798669	0.941397004	1.037649883	1.033828126
Q8R1A4	0.998638898	0.944931644	0.995615681	1.040468915	1.112396179	1.045403647	0.984880653	0.989931786	0.964875725	0.919767833	1.02169872	0.985091844
Q9DCG9	1.027759866	1.088553928	1.026769476	1.010320229	0.965918924	1.008264073	1.016770084	1.015658968	0.976880205	0.960539181	0.973477644	0.942189231
Q8R2Q4	0.952631769	0.959761976	0.977228854	0.963589994	1.057581602	0.937440192	0.953131721	1.073853465	0.907533371	1.106477385	1.123531331	1.039592381
Q9ERR7	1.049260385	1.060608984	1.046416911	1.016308942	0.940896539	0.891762008	1.04966648	1.058658189	1.029461547	0.908899229	0.949427302	0.978210022
Q99LN9	1.556674504	1.329555264	0.820951345	0.743094685	1.344411979	1.207320293	0.709796504	0.698991591	1.135148067	1.642039921	0.656011437	0.620373019
Q9D7A6	1.011740546	0.975142549	1.013858443	0.999852357	0.947956573	0.948797563	0.948265254	1.07362104	1.165511763	0.818312681	0.946639273	1.082745477
Q149F1	1.013185609	0.968287132	0.997768927	1.010358351	0.975709569	1.011776734	0.988826923	1.010143491	1.032778948	1.03217646	0.997830375	0.995232121
Q8BHN0	1.088771959	0.989742347	0.987834343	0.980966119	1.011242938	1.0032718	0.993264124	0.977037412	1.027508737	1.062533984	0.962324222	0.98111921
Q63829	0.948856271	1.051147872	0.969092328	1.066130839	0.979406706	1.001227439	1.01577864	0.997302672	1.044872529	1.022154322	0.989666813	0.940055317
Q8BUY8	0.938183167	0.922983081	1.114827034	1.017874538	0.999038802	1.000013531	0.990783508	1.076581188	0.888308949	0.936047577	1.052368904	1.0474047
Q64282	1.245229593	1.159375157	1.003234738	0.907096712	1.112915982	0.972217396	0.971577333	0.863973546	0.945646917	1.182479915	0.936127727	0.858359926
Q505B7	0.985181491	0.974623754	0.964705113	1.071598909	1.175107211	1.133055976	0.950087954	0.95879869	0.937401455	0.94881373	0.997459693	0.941581019
Q8BHK1	1.008396763	1.145811425	0.910693199	1.002071604	0.966156254	0.809406709	1.050689505	1.324264752	0.901057422	0.883498057	1.062031823	0.884044881
Q9ERY9	0.981002918	1.147779336	1.075911039	0.839035342	0.994627805	0.918333988	1.059552449	1.135270332	0.921302287	0.9670539	1.010602137	0.927674027
Q6ZPK7	0.948248427	0.943891486	0.94543334	1.044757514	1.036256321	1.005232287	1.004578373	1.006736946	0.971541686	1.043211391	1.020497649	1.076526813
Q8BGT6	1.024930363	1.147079789	0.974459602	1.024522506	1.040609687	0.984694266	0.983829547	1.02716326	0.922315957	1.004747177	1.007822878	0.901090954
Q8CA71	0.953120526	0.921977192	0.926872194	0.990426864	1.059599198	0.991059334	1.045626119	1.012720877	1.031446939	0.912352648	1.073104727	1.047801741
P17095	1.204737828	1.079041412	1.143998352	0.993435924	1.008610533	1.003775328	0.984457875	0.925738828	1.024953469	1.128170067	0.764290425	0.874308016

Q7TPD3												
Q8K4L3	0.989924895	1.155089213	0.952464546	0.953099762	1.061671432	1.047330294	0.954302894	1.072609387	1.079266542	1.143659642	0.801636797	0.897943904
Q8CIB6	1.04147131	1.006542784	1.001658838	1.01172375	1.00022846	0.996542834	1.01166653	1.01020318	0.984810346	1.056380371	0.955071406	0.985259886
Q8CBX0	0.894533589	0.935292003	0.902439124	1.0871054	0.939399514	0.985104016	0.987114555	1.08054705	0.930364447	1.247368317	1.045929781	1.088396268
P54797	0.93873184	0.950816609	0.932501	0.950988801	1.064197206	0.97916895	1.044739222	1.031295465	1.028898357	1.166692799	0.948664768	1.051480266
O70145	1.167320915	0.956746477	0.95941434	0.712312836	0.987841151	0.81710508	1.043356158	0.992000004	1.189843825	1.291878024	0.970823959	1.022785145
O35604	1.0427324	1.197819614	1.133528286	0.861089781	1.049209153	0.922281477	0.953808065	1.048092425	0.954987048	0.909303702	0.981810765	0.926289
Q3TYD4	0.90918427	0.969311379	1.003140472	0.96812745	0.903747374	0.973559824	1.095790371	1.095643365	1.052486048	1.058861456	0.971178035	1.006652833
Q3UVU3	1.112689472	1.071663667	1.012647185	0.903272052	0.843777181	0.79696639	1.045088716	1.129037554	1.078639548	0.996656546	1.016539551	0.977346649
Q8C190	0.917604455	0.996894946	0.963625571	1.085005629	0.911102907	1.00501262	1.007525853	1.038271859	1.020881398	0.895255232	1.056684653	1.062852157
Q922V4	1.043646984	1.033721021	1.023102426	0.935876148	0.882908166	0.908833918	1.071452531	1.069313002	1.099645143	1.008764508	0.92504053	1.001587399
P47964	1.018857504	1.036716791	0.99015809	0.894389682	1.44729793	1.000472685	0.813162925	0.889603571	0.855812225	1.556746636	0.977851581	0.8374002
Q7TMS5	1.019221088	1.033790177	0.914251326	0.833192754	1.033378112	1.029889628	1.033012624	1.047228724	1.005704563	1.247756508	0.97272301	0.949487455
P50586	0.937886685	1.009220111	0.987558319	0.95087976	0.941907679	0.92419789	1.032007143	1.062819149	0.999440221	1.056690917	1.066067882	1.043497609
Q9D6U8	0.879303346	1.060405681	1.017673523	1.103248593	1.046309304	1.018731682	0.913953431	1.020102287	0.95759773	0.885451168	1.059400827	1.013177388
Q9JI10	1.184276516	1.170775646	1.119521027	1.029201866	1.163532366	1.101809947	0.876729972	0.911754866	0.904920661	0.868803966	0.899763786	0.814856958
P05366	1.018261869	1.688651889	0.921382298	0.79694376	0.83332083	0.75128591	1.061599578	1.406552585	1.081574497	0.860198351	0.800892567	0.708027655
Q8BKX6	0.922913397	1.132680186	0.941614688	0.960692584	1.025654093	0.907925607	1.074376863	1.020295311	0.968058119	1.140297046	1.034847997	0.930912438
Q8VHL0	1.308399668	1.077414113	1.151343923	0.894748858	1.011393728	0.913764268	0.983769061	1.052298692	1.065109735	0.796913657	0.883325454	0.822119883
Q91VR8	0.98938759	1.131382593	0.960847479	1.038263507	0.964449175	1.012225624	0.851364936	0.98366841	0.959369298	0.979786217	1.132232329	1.016706044
Q9ES56	1.055895335	1.016213129	1.01224556	0.962366503	0.927094023	1.04240062	1.006058317	0.964809349	1.046990188	0.933185337	0.971295252	1.06128114
Q9D3P8	0.8516104	0.942123124	0.937299049	0.976885367	1.006765938	0.963878735	1.072514997	1.069840145	1.044433327	1.074517828	1.080974966	0.980476886
Q8BZW8	1.2712	1.1226	1.0742	1.0114	0.9983	1.0981	0.9478	0.9158	0.9128	0.9114	0.9182	0.8754

	5312	56488	19896	11282	95414	90696	13856	47874	8633	39064	03592	13768
Q8CGV2	0.9483	0.9451	1.0167	1.0497	1.0249	0.9986	0.9997	0.9952	1.0181	0.9759	0.9459	1.1037
	22047	75662	19005	14391	87066	42378	31924	13125	73098	69966	88893	47078
Q925J9	0.9621	1.0408	1.0373	1.0399	1.0168	0.9419	0.9266	0.9946	0.9850	0.9449	1.0617	1.0445
	61418	98957	21078	85687	28538	99694	01879	83418	31471	98588	56569	00668
Q8K341	0.9643	1.0426	1.0354	1.0528	1.0589	1.0753	1.0005	0.9934	0.9029	0.8993	1.0248	0.9430
	04401	2389	00758	27388	86525	69335	08169	992	34687	34578	09872	53182
B2RR83	1.0562	0.9544	1.0086	0.9957	0.9703	1.0478	1.0344	0.9395	0.9780	1.0623	0.9800	1.0370
	20981	43553	15883	51536	21346	49809	31091	33598	1589	44004	27969	73489
O89103	0.9889	0.9802	0.9805	0.9326	1.1005	0.9950	0.9298	0.9703	0.9974	1.3238	1.0091	0.9539
	49224	91117	70082	08994	42873	58978	57311	83758	55621	22787	78377	98557
Q8CBY0	1.0406	1.0208	1.0157	1.0043	1.0608	0.9552	0.9636	1.1040	0.9958	1.0259	0.9413	0.9165
	2916	97507	04465	00986	57742	74859	7432	58866	73556	1942	79849	34545
Q8BYB9	1.0360	1.0807	0.9707	0.9505	0.9886	0.9811	1.0157	1.0532	1.0180	0.9769	0.9754	0.9647
	99895	4174	35701	94528	9005	08685	18187	35066	75022	56877	47401	33408
Q8BRU6	0.7790	0.8688	1.0060	1.1808	1.0685	1.0738	0.9585	0.9883	0.9364	1.0002	1.0385	1.1272
	57166	04069	08505	82638	67176	73552	25202	93231	20469	77197	2943	09009
P98156	0.9562	0.9708	0.9699	1.0294	1.0183	0.9889	0.9112	1.0566	0.9096	1.0743	1.1093	1.0592
	37071	25852	47582	99609	86249	02463	079	89857	68749	60461	02714	02988
Q9JKF7	0.9605	0.9541	1.0160	1.0358	0.9976	0.9596	0.9824	1.0507	0.9949	1.1453	0.9919	0.9889
	77861	22087	00187	04378	26167	54512	19355	60233	02397	30044	20109	85356
P11859	1.1854	1.1887	1.1426	0.8414	0.8397	0.9221	1.1266	1.0509	1.0459	1.0418	0.8239	0.8250
	91526	27031	01511	71932	27434	74683	92137	58902	73367	75077	80829	01572
Q7TSH8	0.9820	0.9992	1.0531	1.0112	1.0296	0.9807	1.0012	1.0324	0.8958	1.0584	1.0299	0.9757
	28556	76535	41307	22131	79086	45757	59768	09083	87217	94928	19148	8855
Q8BHT6	1.0170	0.9777	1.0105	0.9930	0.9638	1.0361	1.0333	0.9629	0.9822	1.0801	0.9594	1.0516
	90834	34161	83886	51743	40704	50437	79189	51419	03225	80151	63153	92651
P97770	1.0933	1.0177	1.0573	1.0238	1.0989	0.9534	0.9678	1.0167	0.9328	1.0002	0.9744	0.9141
	65235	8736	47746	56675	77983	52383	15131	66925	46563	51755	31876	58428
P54818	1.0870	1.0140	1.0181	0.9931	1.0703	1.0385	0.9762	0.9794	0.9664	0.9514	0.9736	0.9620
	71464	79442	70044	93341	14147	48259	05964	75345	15108	51095	26464	91734
O88653	0.8975	1.3392	1.0024	0.8698	1.2016	0.8620	0.9250	0.9991	0.8330	1.2835	1.0576	0.8739
	09852	45636	43674	21101	79247	44816	34231	6434	91779	42373	3319	53003
Q63918	1.1118	0.9327	1.0573	1.0047	1.0999	0.9835	0.9687	1.0670	0.9447	1.0649	0.9496	0.8906
	69382	96222	6696	34731	36231	22341	41578	78577	15293	23003	68018	6438
Q8R4R6	0.9349	1.0584	1.0101	0.9429	0.9664	0.9583	1.0034	1.0687	0.9636	1.1171	1.0625	0.9553
	36217	7419	72501	17026	41848	29965	48074	99525	85618	38037	88513	87305
Q3UHH1	0.9559	1.0366	0.9811	0.9779	1.0182	1.0123	0.9969	0.9795	0.9958	1.0582	0.9958	1.0396
	63459	03154	93835	81817	56001	74431	94468	48766	28561	97227	77794	63421
Q9DAF3	1.1833	1.1335	1.0950	0.8405	0.9145	0.9265	1.0427	1.0844	1.0463	1.0440	0.8782	0.8494
	56514	57955	49065	19613	16444	83824	44967	81194	46503	5376	24796	87665
Q9WVL2	1.1784	1.1365	1.0414	0.9640	1.0549	1.0068	1.0144	0.9711	0.9186	0.9339	0.9292	0.8909
	36824	32496	7454	18745	66695	14538	24884	34665	57757	91144	16546	9989
Q8CBH5	0.9745	1.0188	1.0018	0.9487	1.0314	0.9477	1.0050	1.0361	0.9396	1.1126	1.0442	0.9993

	41089	92387	0474	30454	92759	63239	41967	6913	39855	6982	96533	39428
Q60738	0.943832785	1.040171637	1.068933681	1.098097215	0.930965611	0.882416078	0.894313705	1.002952997	1.04924669	0.685098192	1.114378908	1.160595842
E9Q555	1.248572086	1.080049793	0.973373449	0.872534694	0.923694472	0.907740968	1.092974088	0.908497752	1.114294608	0.983390557	1.013306159	0.889956158
Q8VI51	0.962284355	0.992575763	1.016761062	1.009797217	0.972848806	0.893472267	0.924157869	1.05405543	1.023014819	0.999852524	1.132375221	1.007998315
O88662	1.015374532	0.996085035	0.985055197	0.942774274	0.95261458	0.918533958	1.039874212	1.06294317	1.074405692	0.933263844	1.037317679	1.004416258
Q9D7S7	1.021865832	0.970487751	1.102501932	0.972212696	0.929322387	1.022702038	1.030668736	1.102526635	1.022435891	0.984833931	0.901679494	0.946944184
Q61743	1.052540815	1.103530823	0.956813155	0.931349517	1.08553768	1.097568994	0.859080695	0.985569914	0.901563708	1.352367511	0.924682826	0.975615792
Q8R4V4												
Q8R0Y8	1.002429292	0.94675306	0.999236837	1.02169988	0.960640819	1.001380729	1.068061168	1.029776228	1.021812	1.028144017	0.9521704	0.999151719
Q8CHY3	1.054093124	0.99130839	0.944124656	0.96278602	1.096688265	1.037071271	0.955698364	0.953658393	1.025767341	1.143200008	0.975805947	0.964330011
Q8BX02	1.00844094	1.797101319	0.924675938	0.951248968	1.031759387	0.921912286	0.81475434	0.877639294	0.857049078	0.846535495	1.085954313	0.888231358
Q9D1K7	0.995803369	1.013578246	1.038031303	1.078404564	0.997198433	1.099837544	0.955733128	0.970845159	0.936359315	0.989993789	0.965923513	1.007263883
Q689Z5	0.952483439	0.976121476	0.994432562	0.97348641	0.951499492	1.026071063	0.959256277	0.949704107	1.11577412	0.921118171	1.034851556	1.114819737
Q9D8S9	0.965168172	0.9053881	0.993885599	1.033753975	1.051091458	1.067949123	0.973488594	1.044587224	1.010878858	0.958825942	1.068625195	0.916695749
Q9ES74	1.208709955	1.134857895	1.096934551	0.844032247	0.86009534	0.822939959	1.066380716	1.108790536	1.149048161	1.002642822	0.862547269	0.845295692
Q924D0	1.01269522	1.004440176	1.027797579	1.077963577	1.075756793	1.005901343	0.966685499	0.984253084	0.997790121	0.878997388	0.960748963	1.003619182
P70671	1.02062425	1.017715464	1.054258137	1.022631779	0.971910362	1.026263283	0.973395037	1.01560373	0.972474203	0.901484562	0.990866799	1.024183542
Q64213	1.032462606	1.001791077	1.002244524	1.064230722	0.941442698	1.068256618	1.064777864	0.929789586	1.027267499	1.003406449	0.898837155	1.013497811
Q62193	1.011402835	0.991682899	1.019139268	1.069180692	1.002508582	1.084671983	1.002217337	1.012686942	0.961122061	0.9739993	0.935733233	0.972879312
Q3UH99	0.990770461	1.107464458	0.983203255	0.990153384	1.036766174	1.017368718	0.988599149	0.978126288	0.992738849	0.953902693	0.988052619	0.986862053
Q9CXE2	0.95014212	0.944728707	0.982714387	0.999904614	1.012177624	1.043482741	1.048344703	0.976908918	1.012126657	1.041684015	1.011747226	1.006082303
Q3U1Y4	0.959869641	0.910628584	0.968975898	0.929779934	0.955997989	0.964444691	1.009593507	0.974518687	1.238741412	1.009868465	1.027114142	1.025470175
Q0PHV7	0.86962702	0.856350265	0.900917199	1.019451865	0.956441718	1.001143303	1.0390198	1.002263951	1.017004526	1.177804537	1.06201474	1.171857212

Q9JLI6	1.185891952	1.121361761	1.076248222	0.953214829	0.915769011	1.061913565	1.060681364	0.938517309	0.945612773	1.12771912	0.862462845	0.867844011
P70362	0.999068076	1.05794122	1.031665568	0.927537917	0.94444849	0.961697012	1.057460678	1.041775153	1.076429876	0.902116296	0.968184163	0.987979784
P58802	0.98868867	1.134322265	0.955802848	0.980807632	0.964268352	0.926353062	0.973184994	1.068199969	0.961997161	1.070905664	1.007064067	1.022992331
Q8R3N6	0.721816973	1.059300362	1.01964206	0.896752956	0.866435212	0.893165301	1.091605528	1.268127153	1.063702154	1.018900801	1.045987543	0.981568555
Q9QYK9	1.014047897	1.106875769	0.953956399	0.874494023	1.011416983	0.981228926	0.94853329	1.074342243	0.947571025	1.13790738	1.054455223	0.968511472
Q9QZR0	0.974971384	0.95927032	1.004958461	0.954575064	0.980614184	0.94479067	0.970890156	1.092558228	0.960841899	1.17134151	1.084045314	0.969614626
A2AR02	1.085125676	1.03394534	0.855812309	0.90693156	1.150673693	1.013994925	0.903663536	0.998767243	0.96551379	1.262449712	1.047911752	0.934447714
Q9CYW4	1.003743237	1.100445647	1.179886545	0.887386136	0.95365058	0.876541679	1.131660349	1.065860955	1.076433947	0.889001972	0.921264495	0.842310021
Q80U93	1.068860209	1.105459333	0.963016958	0.945344611	0.924381153	0.973889453	0.985045902	1.011002019	1.010509911	1.005564556	1.044847765	0.982477348
Q6NXI6	0.949051847	1.032076474	0.89498471	0.905199187	0.984838657	1.048321887	1.135463331	1.029717311	0.975086339	1.136803124	0.991303606	0.981034762
Q9D708	1.202439197	1.165970976	1.133804913	0.956524106	1.166522039	1.130541826	0.901562968	0.926490573	0.905075581	0.835158754	0.872468183	0.833591725
Q9CWT3	1.027255234	1.069101287	1.005760308	0.91532482	1.017435068	0.947954373	1.056341571	0.961122533	0.986947555	1.031660859	1.027908618	0.979532264
Q925N1	0.99091503	0.969792953	1.01378077	1.038087666	1.004358291	1.052088254	0.959306412	0.981622017	1.024930033	0.898609103	0.999277511	1.055638347
Q9DBG7	1.064178524	1.024559863	1.045090393	1.0162311	1.001229164	0.926776667	0.993948759	1.025354924	0.920140506	1.069184648	1.050871318	0.917101308
O55229	0.961829414	1.00445909	0.901568457	0.994879794	1.217246442	0.907837563	1.050086851	0.911701042	0.877276019	1.23918417	1.113329232	0.951411754
Q52KI8	1.013173607	0.963340539	0.97185349	0.966962193	1.010345946	1.002958326	1.093746322	1.050540226	0.953189615	1.210447281	0.956474135	0.917397149
E9PV86												
Q3ZK22	1.490503923	1.526459408	0.611192163	0.593054042	1.415061746	1.247578739	0.627946476	0.623962193	1.35362271	1.762314655	0.671874818	0.570001265
Q921L3	1.009021297	1.122898181	1.008599914	1.036851752	1.232505393	0.940854092	0.984212904	0.93851184	0.942418492	0.875686716	1.031588002	0.8693054
O88507	1.179011137	0.893269368	1.035522151	1.037743229	1.140019058	1.105990436	0.983832296	0.932419911	1.017286295	0.87004016	0.90678228	0.919100632
Q80ZJ8	1.030022838	1.035674851	1.074061223	0.940511918	1.022741882	0.979734109	1.009089676	1.001441201	0.955515002	0.949404524	0.979017888	1.030541205
Q8BYN3	1.09815972	1.053054139	0.977414235	0.92342417	1.057296702	1.126769885	0.932207385	0.922035925	1.079684362	1.230859862	0.9075964	0.837838969
Q8VD57	1.1359	1.0332	1.0142	1.1731	1.1602	1.1509	0.9368	0.8652	0.9262	0.9529	0.8963	0.8449

	82168	96953	67179	11327	35335	82104	07421	54818	44374	31784	95654	12632
Q91V24	0.941433347	1.008497028	0.953317669	0.989143075	1.096628441	1.005961453	0.98674308	1.018221811	1.011340496	1.06499588	0.968192868	1.011433623
P23475	1.081125682	1.01727541	1.003116742	0.988823273	1.047457435	1.096240061	1.003033611	0.943105229	0.949784386	1.064572096	0.948986491	0.940806719
Q8K1E0	1.074953944	1.04501611	0.998166874	0.948545688	1.032764329	0.987229952	0.969572057	1.079632528	1.005198115	1.059080372	0.927610637	0.935179839
Q9CZB0										20.17735755		
O35709	0.950501731	1.039631942	0.942271844	0.941227642	0.925482926	0.928159467	1.074835787	1.023155345	0.999153722	1.162336948	1.057783868	1.014594203
Q8CBC4	0.931778844	0.987454425	0.949101881	0.999461396	0.933542562	0.969417309	1.025913929	1.009395055	1.02985862	1.029032429	1.03425042	1.117651564
P47758	1.070693111	0.974004574	1.056968545	0.952637076	1.080990823	1.210492884	1.070446609	1.009429702	0.886617557	1.120147428	0.830833002	0.856060356
Q8BWW4	0.852986836	0.826589049	0.976572825	0.968856583	1.089370976	1.074579267	1.001817706	1.080882022	0.985902209	1.147452425	1.016283894	1.041928212
Q99KD5	0.996567112	0.952503214	0.97723624	0.876681727	1.148240952	0.991426942	1.064297414	1.152389126	0.945049191	1.313730061	0.840684028	0.908297416
Q8BTJ4	1.106175679	1.048191684	1.093130321	1.004441584	1.092359912	1.08546944	0.967062319	0.994434854	0.993318383	0.852590924	0.900863878	0.857464299
Q60739	1.094597875	0.956201086	0.97757057	0.960402634	0.968770157	1.068305968	1.0911887	1.020481666	1.035497136	1.099184135	0.861624232	0.949063925
Q5F2E7	0.980683079	1.003819296	0.972182274	0.984206225	0.967745118	1.068184819	1.040458317	1.024575847	0.967566655	1.002669733	1.006788099	1.002684192
O88455	1.010998565	1.039111883	1.051007362	1.064112711	0.948867345	0.832407849	0.881930519	1.061471108	1.051277184	0.762352737	1.099041265	1.104066507
Q91W82	0.892682896	0.968958114	1.005794275	1.018383166	1.055696038	1.079552301	0.910847753	1.002099481	0.956601945	1.242632062	1.009908776	0.985757976
Q8C3X4	1.07943729	1.100537736	0.956604253	0.982026108	1.043585217	1.000528572	0.998646667	0.974146376	1.00239035	1.084665125	0.955689406	0.902950746
Q91X58	0.934379789	1.084016983	0.940309437	0.751464732	0.995207711	0.948760965	0.908643467	0.820144553	1.037103764	1.235032036	1.366907594	1.022832252
P13808	1.186159276	1.122758319	1.008005061	0.932002057	0.826842583	0.931952092	1.00807232	1.069858473	1.084827738	1.053615413	0.946131244	0.870293493
Q9D2R6	1.056467458	1.237674509	1.030161848	1.226050156	0.756103312	0.930192508	0.805119102	1.136691242	1.025714602	0.892039309	0.946232397	0.964453605
Q3U5F4	1.012820393	1.037881607	0.952034065	0.983429014	1.024785127	0.971070082	1.058841631	1.015987193	1.046365893	1.030984061	0.973195244	0.922107212
O88522	0.964699627	0.987135846	1.035414521	1.040547651	0.905779461	1.061919816	1.027770954	1.004276137	1.008543169	0.921857371	0.996107542	1.027726782
Q3U3Q1	0.911758944	0.972243965	0.975411878	0.960466846	0.922387778	1.01293465	1.059651824	1.004080768	1.049529918	1.035745981	1.03217981	1.068014012
Q61687	0.8897	0.9669	0.9538	1.0035	1.0280	0.9278	1.0732	1.0838	0.9084	1.1198	1.1017	0.9850

	07562	08518	12577	76869	32381	97451	10323	02785	98662	91876	74052	41826
Q9WU63	1.1243	1.0094	0.9733	0.9472	1.0308	0.9986	0.9835	0.9170	1.0141	1.1941	1.0091	0.9185
	21826	22484	1074	61193	82934	30302	39583	45785	11024	40981	064	67861
Q8BVF2	1.0355	1.0526	1.0209	1.0213	0.9485	0.9871	1.0239	0.9812	1.0173	0.8967	1.0048	0.9885
	50704	93992	70059	09357	00139	32915	98568	25014	08911	23755	52234	34735
Q9D387	0.9238	0.9103	0.9834	0.9650	1.1248	0.9333	1.0172	1.0444	0.9406	1.1899	1.1175	0.9215
	28763	8473	86457	48624	19881	30514	38622	42494	92346	36956	4638	64272
Q8BND3	1.0743	0.9812	1.0152	1.0565	0.9404	0.8449	1.0303	1.1405	1.0290	0.9289	0.9648	0.9805
	99168	95578	37242	4932	9989	24004	94622	36054	8683	91628	57185	94565
Q9Z1T6	0.9211	0.9503	0.9883	0.9817	1.0815	0.9505	0.9683	1.0265	1.0244	0.9498	1.0790	1.0572
	88744	85192	77339	75321	10522	54814	73749	11089	00256	09572	19276	55229
P97454	0.9075	0.9556	0.9996	1.0360	0.9957	1.0007	0.9939	1.0347	0.9765	1.0622	1.0502	1.0194
	54836	24353	5452	10377	19657	98141	45919	54141	51104	78996	79284	11406
Q8C739	0.8628	0.8238	1.0567	1.0363	0.9891	0.9474	0.9943	1.2125	0.9817	1.0692	0.9927	1.0513
	6393	51812	99515	37265	96942	49643	35123	13857	9865	32362	77435	88964
Q99PL7	1.0100	1.0027	1.0168	0.9779	1.1878	1.0349	0.9439	0.9811	0.8417	1.0723	1.0723	0.9357
	24569	15973	50859	31803	53074	73484	49072	66601	22363	84981	20406	47048
Q8C5W3	1.0143	0.9723	0.9841	0.9919	1.0399	1.0602	1.0169	0.9754	1.0803	0.9751	0.9477	0.9570
	15202	29719	55967	53001	35981	46801	42818	11074	10913	89104	36164	31988
Q8BXN9	1.0470	1.0363	1.0615	1.0377	1.0810	0.9800	0.9237	0.9702	0.9037	1.0527	1.0182	0.9611
	47802	9733	05977	99133	92126	27199	31142	84037	56119	95865	28291	8588
Q99N84	0.9793	0.9793	0.9780	1.0457	0.9948	0.9834	0.9693	1.0004	0.9532	1.0516	1.0515	1.0630
	22474	4068	46417	36997	86386	00051	8586	54299	59632	32491	63858	6272
Q8CE33	0.9696	1.0041	0.9989	0.9034	0.9918	1.0350	0.9143	0.9917	0.9597	1.1355	1.1318	1.0235
	14158	5573	67115	35881	50228	9309	33346	75756	33292	18929	64839	81324
P58064	0.9787	0.9171	1.0338	1.0351	0.9966	1.0911	1.0628	0.9994	0.9527	0.9157	1.0005	1.0047
	49803	76858	68292	20457	90764	32172	79926	16135	39679	14732	2579	05965
Q9QYK4	0.8898	0.9174	0.9504	0.9952	0.9256	0.9616	1.0666	1.0691	1.1340	0.9427	1.0027	1.0986
	68559	03668	15439	81602	10573	81636	15566	16898	63253	31101	30576	70087
Q3UD01	1.1030	1.1800	0.9151	0.9322	1.0972	1.0666	0.9635	0.9408	1.0516	0.9907	0.9036	0.9146
	90839	76037	9962	36542	89643	38321	33345	16285	24971	01821	832	77534
Q8CF89	0.9624	1.0277	0.9915	1.0420	1.1221	1.0320	0.9428	0.9857	0.9200	1.0556	1.0257	0.9585
	61543	78953	34453	11746	33194	59964	45878	61522	03851	25554	86627	35694
Q8BJD1	1.0326	1.1833	0.9364	0.8317	1.0348	1.0761	0.8474	0.9486	0.8521	1.2131	1.2774	0.8643
	48715	01445	66566	04283	639	35276	08228	50523	84863	59048	05604	40283
Q91VJ2	1.0619	1.0454	1.0404	0.9711	1.0895	0.9904	0.9392	1.0747	0.9265	0.9887	1.0164	0.8858
	11935	96607	18844	55463	51032	18924	08344	2059	73606	50942	996	65275
Q9QZD4	0.6223	0.6877	1.0166	1.1049	1.0887	0.9641	1.0065	1.1134	1.0747	1.4946	1.0131	0.9733
	46328	47923	89928	95359	07273	57832	918	93408	19511	15039	79996	53872
Q8C6I2	0.9225	1.0176	1.0383	1.0382	0.9859	0.9137	0.9516	1.0140	0.9905	0.9238	1.1118	1.0575
	48392	04427	56385	83408	84999	8952	46661	76203	22095	90702	3293	83586
Q6P2L7	1.1096	1.0668	1.0390	0.9894	0.9612	0.9473	0.9942	1.0868	0.9853	0.8912	0.9871	0.9238
	82958	88692	42551	15111	02234	45491	60026	40406	51407	86441	02113	14275
Q9Z131	0.9816	0.9866	0.9997	0.9903	1.0093	1.0296	0.9687	1.0872	0.8929	1.1454	1.0136	0.9859

	36459	81043	58215	9791	32281	02478	12555	80226	95267	45466	67319	30804
O88559	1.0194	1.0268	0.9354	0.9956	1.0617	0.9845	1.0302	1.0872	0.9455	0.9408	1.0236	0.9509
	48641	32133	33291	07988	28574	93305	86044	57225	85342	62887	1944	4945
P30875	0.8456	0.9605	0.9467	0.9942	1.0995	0.9026	1.0766	1.0562	0.9137	1.0904	1.1188	1.0207
	41345	91051	98756	17918	45546	41817	73966	2295	35067	39042	2174	54227
Q3TIU4	0.8984	0.9318	0.9352	1.0148	1.0100	1.0051	1.0231	1.0247	0.9867	1.0925	1.0523	1.0704
	96161	96542	45247	60764	20051	73222	53622	43854	89884	20738	20895	70191
Q91WM6	0.9614	0.9310	0.9825	0.9956	1.0540	1.0100	0.9556	0.9789	1.0352	0.9792	1.0706	1.0481
	86138	95028	21408	75695	71397	79672	56599	14936	17616	46341	32872	58111
A2A8Z1	0.8931	0.9011	0.9295	1.0017	0.9349	1.0907	1.0474	1.0203	1.0385	1.0359	1.0192	1.1039
	48291	99905	17345	00468	23911	01088	62782	75007	7674	74916	13641	26625
P39087	1.2856	0.9905	1.0718	0.9877	1.0201	1.0393	1.0099	0.9161	1.0063	0.8925	0.8883	0.9256
	10985	36329	98549	39905	18761	23027	62502	37909	06589	44879	64472	71604
E9Q6B2	0.9548	1.0230	1.0163	1.0394	0.9104	0.8828	1.0115	1.1395	0.9933	0.9593	1.0344	1.0132
	7802	93878	95608	79132	57166	75512	46419	7002	84992	42469	48389	43888
Q3UTZ3	0.9613	0.8960	0.9703	0.9736	0.9921	1.0711	1.1042	1.0324	0.9755	1.0222	1.0111	1.0067
	02181	10811	12629	2791	65859	75129	91567	4577	49232	98672	29719	08625
P01723	1.9001	1.3600	1.2749	0.7991	0.7313	0.7725	0.8935	0.9777	0.8740	0.8848	0.7966	0.8369
	33892	32809	01989	77315	4133	6788	9252	20488	56785	96835	60791	68591
Q9D0I4	0.9507	0.9977	1.0549	1.0139	1.0354	1.0032	0.9561	0.9566	0.9805	1.0180	1.0060	1.0619
	83559	86083	79836	01524	53308	20576	05472	68391	72025	1146	6883	90921
P97412	0.9710	1.0264	1.0085	0.9483	0.9601	0.8332	0.9421	1.1224	1.0006	1.2278	1.0532	0.9944
	62863	12769	70096	16318	25684	53259	05125	79377	23011	46386	41827	38538
Q91W53	0.9666	1.0358	0.9936	0.9723	1.0112	0.9769	0.9862	1.0261	1.0190	1.0109	1.0168	1.0000
	39486	53486	75375	29404	67474	28877	28365	58412	94534	14669	52178	28083
Q80W00	1.0853	0.9525	0.9448	0.9400	0.8985	1.0049	1.0148	0.9493	1.0281	0.9148	1.0992	1.1458
	82913	12598	00048	94369	40254	14362	98537	53518	63065	94413	12017	05585
Q8K358	1.0592	0.9983	1.0200	0.9847	1.0054	1.0275	0.9641	0.9871	1.0325	0.9530	0.9912	0.9877
	60342	0738	90951	25924	61572	03425	64521	52182	01392	16387	53053	02839
Q9D924	0.9657	0.9197	0.9638	0.9297	1.0118	1.0183	1.0461	0.9802	1.0052	1.0169	1.0738	1.0764
	37897	75181	57598	8736	88472	26854	89426	20958	99969	08209	69737	72553
Q8K4M5	0.9398	1.0720	0.9821	1.1825	1.1510	0.8845	0.9161	0.8807	0.9816	1.0179	0.9551	1.1159
	49906	14881	15396	95358	24612	1029	07281	21229	20184	91833	13412	63389
Q99P47	1.0220	1.0022	1.0091	0.9579	1.0861	0.9538	0.9627	1.0694	0.9884	1.0489	1.0011	0.9410
	90853	53745	66853	9745	97601	55933	81773	68351	0909	3798	12525	75506
Q8QZV7	1.0774	1.2033	0.8925	1.1443	0.9230	0.9545	0.9656	0.8238	1.0724	1.0828	0.8438	1.1444
	24615	73468	31072	6986	12951	35174	21959	20962	29355	0811	35872	40464
Q9D4J7	1.0027	0.9731	1.0114	1.0131	1.0309	1.0005	0.9641	0.9695	0.9352	1.0476	1.0584	1.0448
	11508	81611	24879	98818	8347	70587	08783	28692	50638	05121	65942	21735
P0C7M9	1.0367	0.9793	0.9800	0.9896	0.9676	0.9820	0.9801	1.0196	1.0636	1.0251	0.9791	1.0298
	0663	63706	18564	71691	63649	67674	87105	63424	31149	55677	46488	45588
Q9D338	1.0351	1.0196	1.0246	1.0016	1.0559	0.9748	0.9682	0.9221	0.9381	1.0665	1.0387	1.0229
	02012	43663	94516	48168	10469	09901	44545	94263	59189	64017	41607	54302
P70697	0.9942	0.9548	1.0006	0.9508	1.0179	1.0125	1.0654	1.0431	1.0046	0.9810	0.9598	1.0241

	61811	96173	512	40123	24656	13322	30825	11101	73847	60004	7976	71639
Q8BGY3	0.9793	0.9763	1.0358	0.9918	0.9442	0.9617	1.0428	0.9973	1.0202	1.0593	1.0002	1.0217
	04291	24807	17704	45517	52497	48794	97525	2603	67824	87149	32461	74698
Q6P3A8	0.9534	0.9528	1.0000	1.0467	1.0116	0.9011	0.9810	0.9928	1.0632	0.8873	1.0707	1.0942
	37873	8733	94492	54722	49457	8567	72385	77079	05589	33576	60444	94248
P97401	0.9282	0.9075	0.9191	0.9780	0.9658	1.0146	1.1048	1.0078	1.0640	1.2200	0.9893	0.9880
	65614	17759	56343	52934	57821	39279	87907	5686	02041	61359	04215	86874
P58158	1.0303	1.0032	1.0028	1.0004	0.9507	1.0205	1.0740	1.0229	1.0013	0.9369	0.9719	0.9791
	1852	42412	53385	56931	22888	35303	79859	87007	38148	3667	8838	78576
Q91XE8	0.9897	0.9888	0.9977	0.9670	1.0177	1.0596	0.9978	1.0269	1.0144	1.0469	0.9731	0.9609
	374	11566	85916	0546	85492	20169	65936	87841	5813	59751	84171	92184
P59644	0.9881	1.0179	1.0199	1.0718	1.0215	0.7315	0.8489	1.1002	1.1580	0.6396	1.1265	1.1114
	80092	99652	65038	20197	44326	40697	20552	398	39314	80856	59899	96729
P05977	1.0372	1.0558	1.0519	1.0271	1.1357	1.0644	0.9651	0.9890	0.9209	0.9374	0.9365	0.9142
	24615	19243	16816	15527	73478	42282	99063	32237	9314	90246	45048	2115
Q8BP71	0.9334	0.9306	0.9554	0.9712	0.9989	0.9898	1.0049	1.0018	1.1162	1.2351	0.9424	1.0250
	41799	11807	83457	19603	58098	52935	77542	88301	64346	60773	08141	57598
Q3TWI9	1.0317	0.9635	0.9171	0.9936	0.9441	1.0273	1.0587	0.9185	1.0055	1.1391	1.0276	1.0582
	76494	57554	53917	42546	14941	27281	40858	03786	46273	98217	23259	86498
Q9QWH1	1.2675	1.0932	0.9078	0.8443	1.2044	1.1902	0.8555	0.8779	1.2030	1.4854	0.6986	0.6793
	66691	93383	11717	06372	62865	79464	78648	43775	60757	37036	54033	29145
Q3TRM4	1.4121	1.4180	1.3214	0.6556	0.7314	0.7519	1.2547	1.1786	1.2908	0.6390	0.6288	0.5429
	30347	96085	35888	88786	06478	11341	16113	8239	65411	94797	63419	69633
Q9ERB5	0.9065	0.9485	0.9242	1.0440	0.8975	0.9439	1.0127	1.0473	0.9967	1.0540	1.0921	1.1525
	78968	3811	72438	20622	87878	74059	04328	16229	28189	62947	79859	32176
Q9Z0H1	1.1184	1.1253	0.9715	0.9289	0.9614	0.9931	1.0548	0.9750	1.0620	1.0385	0.8931	0.9331
	73611	16117	36739	94788	03236	08625	11699	45168	47846	23384	14684	09624
B9EJI9	0.9460	0.9648	1.0133	1.0530	1.0490	0.9324	0.9744	1.0454	1.0086	1.0448	1.0402	0.9548
	80204	07834	16688	02639	72538	27219	13122	52621	94769	35211	79021	1175
Q9JKV5	0.9873	0.9661	1.0086	0.9683	1.0852	0.9789	1.0211	0.9773	0.9744	1.0357	0.9677	1.0782
	01938	00177	36577	56469	1177	32461	82027	93448	90407	80035	04849	97187
Q6P4T1	0.9446	1.0369	0.9494	0.8457	1.3425	0.9301	0.8677	0.8907	0.9151	1.2249	0.9572	1.2645
	10086	38867	64758	09881	71882	01893	18824	82067	47467	63888	05817	94338
P62965	1.0032	1.0461	0.9623	1.0283	1.0696	1.1011	0.8833	1.0599	0.9117	0.9996	1.0352	0.9482
	39199	46529	93368	10151	79268	04854	64003	87292	55792	7023	09211	07538
Q8CD91	0.9875	1.0887	1.0397	0.8884	1.0064	0.8332	1.0602	1.0503	1.0951	1.0019	1.0020	0.9251
	63501	30132	82029	58641	21596	27273	76184	86691	99584	37099	22658	59288
Q8BMB3	0.9232	0.9311	0.9413	0.9685	0.9448	0.9993	1.0221	1.0562	1.0108	1.1007	1.1221	1.0020
	0515	00172	97282	85372	2144	09905	14527	30615	10036	36263	44244	06543
P26262	1.2563	1.2212	1.2074	0.7837	0.7001	0.6689	1.3578	1.2046	1.2925	0.7771	0.7193	0.6609
	91593	10623	34363	30093	31325	88437	62404	37334	97555	92678	92247	61446
P58137	0.9981	1.0252	0.9285	0.9984	0.9959	1.0092	1.0330	1.0127	1.0092	0.9464	1.0094	1.0349
	36755	56678	49851	41403	78551	40029	76628	51432	72826	84775	92544	01809
Q9WUZ9	0.9534	0.9624	1.0481	1.0968	1.0299	0.9751	0.9906	1.0143	1.0606	0.9573	0.9812	0.9258

	18548	19385	15829	67822	10586	57931	90896	11455	8647	94281	12787	35951
Q8VHV1	0.8184	0.9269	0.8821	0.9157	0.9361	0.9388	1.1190	1.0723	1.0784	1.0890	1.1504	1.0495
	53726	66185	74486	32179	02224	72556	33147	14049	08737	95758	92765	80798
Q9D799	0.9759	1.0231	1.0026	1.0485	1.0794	0.9604	0.9400	0.9619	0.9683	1.0536	1.0523	0.9871
	66187	8322	3008	91815	28694	06449	21123	52316	48072	21752	0463	49729
Q9D1L9	1.0083	1.0244	1.0121	1.0162	0.9869	1.0120	1.0474	0.9289	0.9544	0.9719	1.0537	0.9942
	7329	93839	29597	91694	59764	73732	74789	85288	13422	42731	33196	1
Q9Z160	1.0584	1.0377	1.0742	1.0094	0.9445	0.9898	1.0366	0.9684	1.0494	0.8039	0.9384	1.0356
	77382	12009	58445	84566	37743	47297	4195	95515	42924	81734	44485	08957
Q80YE4	0.9734	1.0365	1.0101	1.0741	1.0244	0.9482	0.9467	0.9907	0.9961	0.9848	1.0120	1.0280
	95877	42877	00837	95462	61273	62341	38059	59637	56795	69521	0522	11251
P20491	1.1470	1.1781	1.0167	0.8376	0.9407	1.1617	1.1143	1.0550	1.0309	1.0937	0.7927	0.7089
	52099	1648	70435	08533	34978	24338	52158	38928	62891	57847	08074	37402
Q99N94	0.9934	0.9825	1.0330	1.0110	1.0377	1.0083	0.9775	1.0050	0.9837	1.0033	0.9952	0.9986
	06845	78435	10975	98242	93899	86634	37753	19407	67174	00688	39839	05286
Q9ERF3	1.0379	0.9946	0.9550	1.0290	0.9883	0.9234	0.9623	1.0264	1.0230	0.9113	1.0522	1.0833
	12251	23161	66402	61293	10857	79145	15247	86026	04724	22316	86385	27511
O88291	0.9859	1.0100	1.0247	1.0382	1.0031	0.9549	0.9738	0.9644	1.0066	0.9129	1.0977	0.9996
	59764	18281	11728	29292	6138	64691	42818	38142	84255	23818	70097	60631
Q8BKY8	0.9501	1.0692	1.0196	1.0599	1.0658	0.9614	0.8992	0.9989	1.0022	0.9907	1.0427	0.9606
	71473	23165	91697	09008	68765	83682	08654	38722	75922	3392	12815	76682
Q6RKD8	0.8910	1.0711	0.9444	0.9813	1.0767	0.9855	0.9786	0.9804	0.9248	1.1225	1.0797	1.0327
	80462	57327	41363	18908	76012	38594	24312	78288	29138	44883	66297	48477
P04940	2.9057	2.0088	1.6052	0.8283	0.6542	0.6309	0.7642	0.7162	0.6906	0.5286	0.4295	0.4141
	74478	52768	74243	27289	36326	44229	22073	22485	29937	16448	22849	17917
P34928	1.0896	1.1488	1.1718	0.8701	0.8184	0.7307	1.1701	1.2260	1.3267	0.8428	0.7815	0.6982
	18079	45612	60323	78608	29717	63582	72346	32088	13725	66327	01362	02214
Q80Y56	1.0831	1.0858	0.9811	0.9867	0.9916	1.0029	0.9752	0.9865	0.9900	0.9708	1.0146	0.9558
	65729	4794	35378	66545	01813	48209	20047	08873	36094	94415	82592	58575
P49070	0.8476	1.0651	1.0463	1.2207	1.0325	0.9566	0.8841	0.9780	0.9462	0.7643	1.0400	1.1619
	86883	60531	55952	14296	60159	44893	8909	68342	84173	90601	82041	41812
P51175	0.9799	0.9696	1.0584	1.0425	1.0590	0.9156	0.9546	1.0674	0.9356	1.0144	1.1537	0.8491
	87323	59671	76234	19899	66149	47798	89329	12927	71549	92769	74105	20633
Q80XS6	1.0741	1.0334	1.0126	0.9736	1.0107	0.9496	0.9721	1.0310	1.0292	0.9744	1.0022	0.9493
	91574	1796	46984	30972	48302	56787	06629	45589	80222	19405	64996	97465
Q6PGH1	1.0225	0.9590	0.9650	0.8423	1.0515	0.9612	0.9623	1.1300	1.0569	1.2140	0.9474	0.9869
	52583	93762	88279	86991	1665	45043	92328	42074	15309	87181	80704	73176
Q99MX0	0.8669	1.1619	0.8802	1.0211	1.0462	1.0982	1.0362	0.9323	0.9085	0.9765	1.0276	1.0734
	96	00355	37971	79437	42634	7892	26567	04658	55454	5141	92952	51613
Q64096	1.1269	1.0500	1.0160	0.8853	1.0129	0.9658	0.9819	1.0018	1.0420	1.0879	0.9569	0.9388
	85354	50142	68893	2909	39878	31989	51389	56658	94345	17769	7908	24205
O35739	0.9731	0.9441	0.9230	0.9152	0.9907	0.9337	0.9846	1.1578	1.2076	1.1134	0.9611	0.9200
	81527	16967	13054	07368	68229	38032	61965	21859	67242	42184	03339	93549
Q8BKI2	0.9746	0.9274	0.9559	1.0245	1.0599	1.0951	0.9762	0.9769	1.0423	1.1004	0.9783	0.9606

	40971	2696	91663	81455	43431	60307	21421	43344	70698	92084	44387	04875
Q9D1B9	1.0198	0.9934	0.9948	0.9960	0.9256	0.9868	1.0557	0.9928	1.0465	0.9145	1.0277	1.0159
	63197	98076	04629	58941	83735	31839	41222	85634	51904	42712	05939	88673
Q8C0J6	1.0149	0.9925	1.0413	0.8358	1.0573	1.0159	1.0607	0.9214	0.9046	1.2719	1.0795	0.9236
	67673	70133	91905	68971	75056	00966	7582	38283	7318	56154	96991	09598
Q8CE90	1.0839	1.0466	0.9165	0.9583	1.0165	1.1011	0.9278	0.9528	0.9956	1.1729	1.0044	0.9455
	71884	99448	49812	37214	49374	47289	04179	67039	4588	44193	99039	50212
Q8BI08	1.0314	1.0387	0.9648	1.0546	0.9070	1.0801	1.0431	0.9766	0.9985	0.8169	1.0043	1.0400
	37094	42872	68799	37286	79411	57298	03146	29726	95823	66664	85655	2593
Q31125	0.8993	0.9568	0.9911	1.1215	1.0000	1.0524	0.9677	1.0109	0.9284	1.0790	1.0375	1.0172
	16145	81327	01356	72803	22671	56048	43268	01706	78058	73019	8621	43375
P97291	0.8872	0.9158	0.9742	0.9825	0.9383	0.9683	1.0252	1.0050	1.0783	1.0825	1.0571	1.1019
	26837	22724	67974	02441	46444	4029	08235	46939	57339	79735	36429	63822
Q9JHI7	1.0180	0.9477	1.0175	1.0405	0.9960	1.0153	1.0214	0.9933	1.0469	0.9208	0.9766	0.9958
	16569	7351	79946	66031	60948	03363	2013	60372	88675	75826	23977	54513
Q68FF0	0.9578	1.0050	0.9983	1.0031	0.9593	0.9752	0.9999	1.0378	0.9648	1.1325	1.0070	1.0317
	53305	76412	77849	13155	2662	67259	00671	81406	76357	53448	94164	26888
Q80W37	1.0301	1.0206	1.0348	0.9420	1.0120	0.8430	1.0968	1.0644	0.9553	1.0484	1.0329	0.9387
	1007	75212	06162	60561	18891	04175	76767	29433	60771	33457	90323	74333
Q64704	1.0469	1.0793	0.9435	0.9877	1.0403	1.1060	0.9603	0.9498	0.9934	1.0914	0.9528	0.9410
	42995	11706	80074	234	71329	45574	8106	99	77561	33208	69973	9618
Q69Z66	1.1177	1.0342	1.0073	0.9248	1.0074	0.9905	1.0121	0.9742	1.0402	1.1640	0.9215	0.9117
	03405	27762	80138	12243	02527	98811	873	19391	7433	1206	82753	83221
Q8BGF6	0.9827	0.9852	1.0139	1.0175	1.0121	0.9650	1.0553	1.0811	0.9362	0.9783	0.9884	0.9958
	92137	51033	95343	3327	76815	3857	17041	18434	91293	76364	91398	73424
P03893	1.1138	0.9947	0.9991	0.9548	1.0958	1.0040	0.9467	0.9012	0.9841	1.2085	0.9861	0.9526
	89914	64883	00874	75652	61443	35261	57169	04597	63122	1336	13863	46291
Q5U4F6	0.9336	0.9885	1.0042	0.9932	0.9891	1.0424	0.9973	1.0306	0.9640	1.0595	1.0122	1.0264
	33005	12537	78975	10491	69909	17199	65433	3119	35429	26546	62266	67701
Q8K0G8	1.0417	1.0422	1.0122	0.9552	0.9290	1.0163	1.0017	1.0277	1.0693	0.9949	0.9309	0.9982
	7605	2885	67557	61172	74422	69171	00987	70686	25814	71721	22132	48486
Q6ZPS6	1.0734	1.1545	0.9137	0.8555	1.0440	0.8863	1.0867	1.1127	0.9629	0.9589	1.0182	0.9269
	98479	88902	66359	09969	36294	23883	706	8693	41924	69583	4794	17033
Q69Z38	1.0701	1.0619	0.8693	0.9241	1.0872	1.1100	0.9385	0.9047	1.0185	1.3899	0.9147	0.9417
	57786	53591	08671	32194	55963	29441	87872	06614	09807	47592	95115	6394
P22907	0.9245	0.9982	0.9941	0.9585	1.0303	0.9754	0.9849	1.0372	1.0668	0.9220	1.0496	1.0172
	35937	83729	99562	58237	83187	59981	86685	69454	79952	45544	44474	61366
Q6ZQH8	1.0183	1.0915	0.9741	0.9471	0.9643	1.0082	0.9457	1.0075	0.9452	1.2453	1.0382	0.9428
	34978	55065	59571	56439	35516	4246	0904	17123	49871	31511	06234	889
Q5U4D9	1.0280	1.0616	1.1230	1.0175	1.1300	0.9293	0.9046	1.0114	0.9471	0.8416	1.0306	0.9428
	07677	1742	6941	98217	51712	97185	19949	67083	70723	39971	31986	40404
Q8BG94	1.0442	0.9656	1.0171	1.0677	1.0894	1.0349	0.9294	1.0000	0.9847	0.9302	0.9726	0.9881
	15421	85039	85346	74809	71049	6507	27235	09814	61932	42955	05983	98947
Q8CFV9	1.1242	0.9204	0.9307	0.8456	0.9399	1.2585	1.2035	1.0489	0.8804	1.3383	0.8154	0.8929

	72007	65251	38269	94313	09719	40687	97201	84919	17218	39327	2635	75785
Q3UFY8	0.9383	1.0350	0.9950	1.0134	0.9814	0.9404	0.9788	1.0390	1.1056	0.9530	1.0107	0.9852
	3552	99174	33482	40169	68736	79919	22344	46981	40799	14012	27485	53552
O54949	0.8733	0.9645	0.9636	0.9840	1.0259	0.9404	1.0263	1.0027	1.0078	1.1005	1.0679	1.0781
	43046	64978	13723	77166	99646	9864	72443	86807	40845	15349	49612	39127
Q8C9B9	1.0322	1.0097	0.9885	0.9610	1.0192	0.9911	1.0673	1.0184	0.9764	0.8658	1.0210	1.0118
	5337	81678	22518	43728	75392	67501	22589	52672	82678	06902	74571	38813
Q3V1M1	1.0338	0.9627	0.9039	0.9529	1.0731	1.0249	0.9925	0.9044	1.0410	1.3670	0.9755	0.9599
	89205	24491	17507	89609	65872	52944	32732	64061	44173	98668	1545	01183
Q8C9S4	0.9921	1.1560	0.8447	0.8216	1.0725	0.8891	0.9230	1.0267	0.9438	1.1318	1.1449	1.1226
	52298	73882	80504	85862	85374	54387	5854	75223	29675	42178	31853	63087
P41241	1.0509	0.9939	0.9367	0.9234	0.9763	1.0010	0.9878	0.9825	1.0416	1.0119	1.0630	1.0494
	37557	15925	75909	86697	98775	71145	70795	81142	32268	6275	32212	04689
Q9CQS8	0.9994	0.9794	1.0005	0.9526	0.9260	1.0135	1.0680	0.9993	1.0618	0.8972	1.0196	1.0372
	95294	46477	01735	07884	60822	70825	13704	73294	18246	41999	43698	91302
Q8K1N1	1.0267	0.9176	0.9591	1.0328	0.9872	1.0214	1.0574	1.1413	0.9768	1.0147	0.9388	0.9605
	83867	08534	72844	82767	55609	94193	31564	74587	18145	64337	58218	52429
Q3UGP8	0.9687	0.9251	0.9792	0.9860	0.9569	0.9636	1.0054	1.1062	0.9444	1.0101	1.0779	1.0842
	76767	69353	45833	39755	1691	819	74924	90407	55479	91143	04727	89156
Q5Y5T1	0.9211	1.0397	1.0247	1.0520	1.0744	0.9659	0.9772	0.9928	0.9425	0.9817	1.0250	1.0224
	49005	39593	03916	0354	82332	36302	40098	02672	36345	80469	01846	6043
P70261	1.0003	1.1360	0.9692	0.9932	0.9888	0.9313	0.9717	1.0532	0.9949	0.8826	1.0327	1.0167
	6414	18061	7618	17873	06071	65265	98139	79995	24306	31503	09324	08796
Q09014	1.0976	1.0602	0.9747	0.9069	1.0898	0.9816	1.0113	0.8926	1.0340	1.2969	0.9192	0.9062
	26896	12822	79225	03602	02407	81642	07625	74712	21337	12614	63278	35929
Q99JN2	1.0344	1.0117	1.0065	0.9027	1.1019	0.9856	0.9109	0.9690	1.0054	1.1594	1.0414	0.9621
	84427	88044	7362	07278	27466	17508	7741	30711	18306	28079	5448	24632
O35387	0.9621	1.0295	1.0180	0.9908	1.0789	0.9792	0.9901	1.0145	0.9941	1.0244	0.9866	0.9636
	11379	95355	12304	67198	31831	66234	81191	68945	95736	62788	68568	77946
P00520	1.0652	1.0697	0.9909	0.9860	1.0693	1.1197	0.9818	0.9622	0.8742	1.0737	0.9557	0.9517
	11704	95112	5138	88102	95492	65872	91865	66067	04904	70699	42264	72563
Q9CQL5	1.0689	1.1074	1.1023	0.8415	0.8871	0.8923	1.0289	1.1487	1.0886	1.0017	0.8974	0.9273
	17801	9435	48569	44664	28652	85944	91613	61304	46539	56575	22757	8398
Q9WTX7	1.0134	1.3549	1.0252	0.6822	0.9191	0.9476	1.1850	1.1110	0.6733	1.3465	1.0542	0.8316
	33484	71235	46911	63115	62109	74511	14307	43964	71585	30852	68698	42009
Q9DCU2	0.9577	0.9393	1.0129	1.0848	1.0568	1.0525	0.9152	0.9877	0.9523	1.0106	1.0424	1.0299
	15709	8674	20063	13784	53324	46213	025	12889	02106	86124	40473	9598
Q8C2E4	0.9496	0.9272	0.9659	0.9828	0.9778	0.9204	1.1084	1.1642	1.0594	0.9949	1.0057	0.9179
	21365	80005	81239	58866	07673	65095	01663	20605	91793	57344	72509	82001
Q9CQ10	1.0947	1.0577	1.0115	1.0552	1.1450	1.0881	0.9240	0.9324	0.9160	0.8526	0.9641	0.9800
	98734	00626	39024	18594	55092	15233	08015	8914	53058	95964	93262	47192
Q91WM1	1.0193	1.0960	1.1650	0.7872	0.8894	0.7690	1.1314	1.2154	1.2167	0.9850	0.8651	0.7914
	84529	98597	17772	36437	09883	40481	97958	87243	30319	37437	76698	30322
Q9DB32	1.0082	1.2020	1.0433	1.0341	1.0242	0.9343	0.9255	0.9866	0.9230	0.9024	1.0270	0.9903

	34694	71865	03072	16532	59972	02463	74191	75068	94623	63944	64868	34434
Q8BR07	1.0657	1.0294	0.9301	0.9492	1.1078	1.0058	0.9482	0.9810	1.0061	1.1580	1.0369	0.8805
	40206	59359	86142	40392	39473	74663	23803	14445	06044	69342	76801	32671
B1AUE5	0.9425	1.0696	1.0363	1.0569	0.9624	0.8902	0.9179	1.1218	1.0837	0.8854	1.0312	0.9467
	26584	99042	83448	39985	47982	38799	97525	77634	17293	8777	39476	42179
Q9CRB0	1.0359	1.0665	1.0336	1.0510	1.0678	0.9551	0.9625	1.0184	0.8802	0.9732	1.0821	0.8967
	06042	53933	38956	4036	74155	47147	45622	16579	52698	65168	14299	23627
Q8BG16	0.8711	1.0048	1.0359	0.9650	1.0137	0.8703	0.9658	0.9450	0.9725	0.9427	1.2268	1.1350
	36303	06362	91526	45445	51197	46634	5219	06711	03587	564	7578	40037
B2RWJ3	0.8110	0.8926	0.9521	1.0841	0.8593	0.8429	0.8773	1.0295	1.1757	0.6695	1.3438	1.2510
	48459	72746	62636	95068	87653	2363	32664	75596	24333	36596	01204	24251
Q3UBX0	1.0258	1.0428	1.0281	1.0799	0.8907	1.0388	0.9589	1.0354	0.9955	0.8809	0.9964	1.0083
	84974	4133	14446	49645	55118	62684	00881	47983	47175	34732	78635	09545
Q8BVU0	1.4441	1.0010	0.8782	0.8184	1.0273	1.0820	0.7953	0.8328	0.9792	1.2339	1.1467	0.9438
	24557	09093	85293	10145	04764	75277	93982	90691	10233	03793	64391	22047
Q9ES89	0.9945	1.0652	1.0012	0.9670	0.9200	0.9706	1.1121	1.0835	1.0117	0.9667	0.9245	0.9787
	19127	75452	9694	11338	28639	52355	02599	84727	67856	2819	01961	89842
Q9CZV5	0.9035	0.9246	1.0172	0.9416	0.9597	0.9389	0.9902	1.0823	1.0750	1.0809	1.0833	1.0028
	35362	87324	86517	52891	51957	85336	91616	48632	61148	92103	9871	9609
Q8CGA0	0.9908	0.9939	1.0538	1.0599	0.9727	1.1027	0.9209	0.9250	1.0355	1.0398	0.9533	1.0101
	07605	777	26075	5621	78818	77095	23842	12445	33878	02091	52384	83177
Q99K95	1.0556	1.0856	1.0355	0.9036	0.8835	0.8588	1.0568	1.1270	1.1199	0.9825	0.9755	0.8853
	02567	82676	07688	89485	13051	52805	09657	23172	4448	63223	60558	83378
Q64689	0.9936	1.0065	0.9675	0.9556	0.9701	1.0313	1.0516	1.0375	1.0250	1.0169	0.9381	1.0353
	43717	30374	26078	86823	73148	01688	78667	08768	25254	25853	24611	10297
Q9D0B0	0.9052	1.0423	1.0328	1.0119	1.1687	0.9728	0.8875	1.0164	0.9059	1.0863	1.1127	0.9109
	19594	82813	00086	00253	17861	90134	8493	33654	2707	05094	73724	37964
Q9D5J6	0.8591	0.8087	0.9420	1.0037	0.9198	1.0391	0.9631	0.9475	0.9729	1.2138	1.2193	1.1810
	66604	08368	38343	53299	3675	36978	85147	38797	6723	6491	62126	32338
Q8R3P6	0.9437	1.0299	0.9618	1.0457	0.9647	0.9604	0.9034	0.8740	0.9584	0.9734	1.1538	1.2471
	44284	9118	31408	28848	07326	47829	32078	50932	47677	23049	65563	49676
Q8R121	0.9560	1.2640	1.1160	1.0142	1.0139	0.9797	0.9886	1.0189	0.8989	0.8615	0.8977	0.9809
	52751	04614	30688	38274	70096	10403	28375	00142	04909	4656	63658	79188
Q60603	1.0342	1.0910	0.9845	0.9809	0.9822	0.8788	0.9714	1.1133	1.0876	0.9478	0.9969	0.9120
	92732	06626	10952	96769	915	83029	95589	94945	09313	28501	87352	38347
Q9D7B1	0.9218	1.0773	0.9944	1.0374	0.9773	0.9139	1.0029	0.9995	0.9594	1.1092	1.0268	1.0420
	63471	22782	33896	44898	72461	39513	42119	1572	30529	78216	73505	55825
Q8R1B5	0.7662	0.9735	0.8628	0.9355	0.8793	0.9176	1.1704	1.1265	1.0180	0.9626	1.1447	1.1665
	98971	69544	18974	04185	72271	80084	32905	62318	89276	62653	53184	56369
Q8R4X3	1.0339	1.0091	1.0069	0.9534	0.9691	0.9889	1.0468	1.0556	1.0700	1.0686	0.9243	0.9142
	12749	89583	43715	72512	1795	99102	29926	55926	15843	31252	56354	0659
Q8VE96	1.0094	0.9587	0.9989	0.9387	1.2250	1.0098	0.9493	1.0500	0.9070	1.1348	1.0430	0.8637
	65312	45502	40503	15045	06209	69326	20088	66202	42331	65194	23588	11531
Q4VBE8	0.9703	0.9693	1.0007	1.0321	1.0293	0.9314	1.0350	1.0262	0.9626	0.9807	1.0346	1.0360

	57887	91909	37414	52966	46678	49797	64546	15848	3171	09277	47004	48206
P70193	1.2173	1.1833	1.2100	0.8106	0.8877	0.7605	1.1052	1.1996	1.0161	0.9920	0.8513	0.7612
	23411	89988	78632	87116	01069	14228	41409	53896	02114	41128	45499	56724
Q9D8X2	1.0517	1.0476	0.9725	0.9853	0.9581	0.9883	0.9813	1.0570	1.0385	1.0160	0.9476	0.9930
	96896	80855	5722	26607	82872	64094	35179	53296	48689	8758	82563	54981
Q5HZI2	1.0684	0.9878	1.1049	0.8616	0.9231	0.8715	1.0845	1.1781	1.2619	1.0318	0.8833	0.7082
	25323	99237	24397	30793	84317	98619	40365	03015	93753	00171	0534	05429
Q80WV3	1.0139	1.1271	0.9888	0.8974	0.9707	0.9117	0.9890	1.0793	0.9953	1.0999	1.0221	0.9509
	43269	20888	11197	75066	46297	50302	81884	19969	12514	43317	15184	23129
Q3TGF2	1.0014	1.1626	1.0950	0.9079	1.0153	0.9238	1.0058	1.0330	0.9399	0.9442	1.0681	0.8773
	39807	48899	42526	65421	56169	52128	20152	48914	52498	73944	52767	42843
Q99MR3	1.0871	1.0100	0.9824	0.9867	0.9664	1.0246	0.9731	1.0237	1.0204	1.0715	0.9609	0.9603
	11805	33945	73414	6865	4648	58241	44996	2533	93506	49274	3419	43912
A2AB59	0.8619	0.8956	0.9395	1.0669	1.0615	1.0550	0.9495	0.9894	0.9929	1.0617	1.0916	1.0720
	10925	07918	0264	44672	9952	45015	10102	18833	42314	65609	18621	42495
Q8BH15	1.0124	1.0075	1.0254	0.9752	1.1022	0.9966	0.9833	0.9945	0.9938	1.0012	0.9947	0.9413
	2853	87117	59422	93798	6611	5321	36978	5796	98373	94197	39351	00024
Q499E0	0.8798	1.0395	0.9690	1.0331	1.0905	0.9148	0.9572	1.0772	0.9357	1.1028	1.1492	0.8851
	30744	00125	69281	32008	56689	24905	3513	12403	52945	84591	65877	98992
P03958	1.1489	1.0830	1.0437	0.9690	0.9955	0.9836	1.0134	1.0458	0.9903	0.9077	0.8627	0.9768
	34833	84946	218	06658	23593	06029	80818	54567	99402	92237	47346	35824
P09242	1.0273	1.0475	0.9454	1.0151	1.0247	0.8752	0.9947	0.9936	0.9821	1.0207	1.1186	0.9748
	97778	73802	40238	91152	10655	56481	9143	86992	20002	89451	81489	78556
P32211	1.0612	1.0280	0.9355	1.0172	1.0512	1.1072	0.9027	0.9233	1.0086	1.0040	0.9510	1.0830
	06597	28229	74325	77433	8617	09334	41181	31664	06371	05698	95569	98846
P58929	0.9990	1.1476	1.0103	0.8525	0.8860	0.9283	1.0822	1.1634	0.9916	1.2439	0.9677	0.8109
	70859	56206	79425	73544	08417	99633	42166	00155	70217	37298	6823	88275
Q66L42	0.9393	0.9750	0.9923	1.0383	1.0272	0.9860	0.9651	1.0171	0.9454	1.0009	1.0558	1.0813
	29548	71404	15147	61337	09044	04559	96518	33169	4308	04895	22834	39807
Q64345	1.2985	1.0492	0.9955	1.0029	1.0849	1.0740	0.9211	0.8880	1.0357	0.9738	0.8855	0.8780
	06259	74303	60079	51159	3262	32274	1161	77049	68442	00399	55398	71904
Q8VE95	0.9178	0.9307	0.9446	0.9929	0.9667	1.1118	1.0772	1.0093	1.0299	1.2377	0.9964	0.8830
	41464	1492	83248	57933	92867	20148	81596	31383	01509	1405	04907	83609
P42227	1.0529	0.9329	1.0454	0.9541	0.9341	0.9144	1.0762	1.0709	1.0979	0.9724	0.9670	0.9624
	45679	0051	70313	77109	69069	41728	83863	22709	35019	34746	69603	99737
Q9JJ69	0.8679	1.1375	0.9538	1.0653	1.0191	1.0090	0.9645	0.9723	0.9493	1.0600	1.0032	1.0570
	44901	18571	80989	47411	20091	97786	74295	04847	8409	13151	22092	0548
Q8BFR4	1.0503	0.9788	1.0904	0.9406	1.0770	0.9101	1.0273	1.0193	1.0070	0.8662	0.9870	1.0065
	43057	53139	79298	63761	13024	44165	0933	57026	57742	80224	20913	53896
P42128	0.8923	0.9554	0.9716	1.0347	0.9709	1.0158	0.9955	1.0339	0.9725	1.0442	1.0755	1.0589
	79225	61355	47073	01603	61651	92256	28555	49149	27063	91807	59826	77945
Q9CQ02	0.9935	1.0428	1.0069	0.9551	1.0054	0.9940	0.9971	1.0396	0.9610	1.0005	1.0716	0.9387
	34578	09821	58937	61089	83674	51613	92638	46134	46875	92674	56816	99587
B8ZXI1	1.0165	1.1172	0.9840	1.0916	0.9920	0.9241	0.9438	0.9818	0.9726	1.1057	1.0361	0.9130

	44732	19529	46158	75887	48183	83416	61117	38321	44465	95026	58634	29584
Q8CAL5	0.9271	0.9031	0.9973	1.0114	1.0210	1.0187	0.9795	1.0001	1.0025	1.0313	1.0620	1.0666
	49158	16085	84297	01063	96541	33463	83035	87019	88025	93065	29741	15095
P14847	1.1209	1.2043	1.1823	0.9102	0.8621	0.8070	1.0372	1.1607	1.1308	0.9008	0.8225	0.8172
	82305	65088	67124	52224	02974	47935	0128	79375	05614	63728	63281	06356
Q99LH2	1.0498	0.9808	1.0510	0.9727	0.9689	1.0634	0.9951	0.9957	0.9806	0.9578	0.9897	1.0064
	64803	37089	37844	05304	45469	00485	47135	60577	86589	37954	0818	14553
P58242	1.1546	1.0603	0.9814	0.9945	1.1945	1.1007	0.9120	0.9333	0.9486	1.2538	0.8102	0.8716
	64724	24941	44171	95154	22371	18883	86376	27942	42911	32901	75502	68922
Q3V2J0	0.8284	0.8961	0.9331	0.9773	0.9565	0.9418	1.0668	0.9949	0.9583	1.2084	1.1510	1.1496
	56433	2137	90113	18168	91384	25592	67537	33675	47659	90481	55195	7759
Q8CD54	0.7684	1.0078	1.0419	1.0507	1.1509	0.9460	0.9077	0.9767	0.8150	1.2521	1.1414	1.0611
	00662	03359	5971	36718	83782	00217	73472	45249	31557	17941	22172	91263
Q9QXW9	0.8150	0.9083	0.9993	1.1592	1.0225	0.7969	0.8144	0.8517	1.2414	0.6398	1.2522	1.3162
	02469	06503	10279	42208	12909	04735	43439	80439	99806	84536	00679	89028
P03975	0.9952	1.1064	1.0218	0.9790	0.8924	0.9118	1.0014	1.0359	1.0557	1.0136	0.9930	1.0018
	64786	68486	7793	74816	53369	67891	35947	77034	99381	70276	27512	87851
Q3U0D9	0.8732	0.9700	0.8641	1.0620	1.1623	0.9715	0.8807	0.9121	0.8940	1.3317	1.2156	1.0253
	26072	96156	55762	58783	83966	92541	40277	53495	91082	08768	4378	09188
P03921	0.9852	0.9589	0.9923	1.0098	0.9616	1.0292	1.0292	0.9663	1.0196	1.0607	0.9874	1.0468
	35814	7384	70376	68533	17325	09454	22054	65058	06479	31401	15631	91218
Q3U213	1.0602	0.9976	0.9810	1.0192	0.8820	0.9348	1.0772	1.1760	0.9907	0.9145	0.9551	0.9908
	70393	78034	02249	45625	88184	10175	28451	94706	48598	04684	57918	2935
Q8BH43	1.1058	1.0155	0.9378	1.0092	1.0207	1.0476	1.0345	1.0156	0.9929	1.0937	0.9115	0.9088
	86659	63992	24248	63991	76816	6492	55357	47435	20026	47214	1482	82442
O70200	1.0054	1.0141	1.0141	1.0339	0.9920	1.0120	1.0122	1.0237	0.9471	0.9913	0.9956	0.9862
	04842	93639	61294	81659	01728	16206	89211	15322	22327	66487	33168	9715
P97490	1.0919	1.0833	1.0645	1.0100	0.9128	0.8221	1.0790	1.2086	1.0095	0.8659	0.9251	0.8781
	94497	65833	90214	25594	30807	25928	35989	20184	23706	02481	46512	95842
Q7TSK2	1.0547	1.0066	1.1209	1.1356	1.0170	1.0459	0.9362	0.9574	0.9922	0.8398	0.9577	0.9202
	8719	27525	05804	31962	10838	28673	59696	74648	10055	94297	95777	35365
Q3TC33	0.8485	0.8418	0.9538	1.0303	0.9980	0.9914	1.0402	0.9966	1.1107	0.9210	1.0642	1.1515
	72168	37968	10078	10144	00324	02676	11789	73517	02988	7187	64885	09241
Q8R3K3	0.9137	0.9551	0.9580	1.0320	1.0093	1.0013	1.0538	1.0007	0.9442	1.1164	1.0491	1.0272
	84147	36434	37205	52904	17208	5485	35167	8169	74876	07445	92329	39653
Q9Z224	0.9618	1.0115	1.0339	0.9452	0.9842	0.9558	1.0361	1.0122	1.0549	1.0266	0.9869	1.0002
	98169	63854	51039	25574	53341	47251	67407	24365	31045	62121	43033	92458
Q8CDD9	1.0176	1.1130	0.9244	0.8817	0.9901	0.8744	1.0247	0.9779	1.0378	1.1489	1.0660	1.0052
	43409	44964	62613	23215	91928	28472	69264	16963	98418	52037	54673	72672
Q80XD1	1.0555	0.9877	1.0726	0.9528	0.9575	1.0155	1.0752	1.0477	0.9641	1.0066	0.9625	0.9216
	04753	5045	70303	90841	56342	89027	36951	89527	83581	68318	01368	09404
Q8JZX4	0.9338	0.8995	0.9778	0.9923	1.0428	1.1442	1.0267	1.0095	0.8810	1.2557	1.0272	0.9429
	5418	98482	16549	73607	81697	33643	54485	57328	61187	17924	64301	05763
Q9EST4	0.9154	1.0215	0.9944	1.0317	1.0487	0.9319	0.9393	1.0427	1.0368	0.8448	1.0812	1.0479

	28881	56789	35336	64202	93672	0003	55262	26979	8442	56085	49244	14561
Q6NZL6	1.0161	1.0813	1.0650	0.9766	0.9806	0.9711	1.0127	0.8709	1.0131	1.0389	0.9837	1.0345
	03493	02093	68565	51055	5208	85624	20795	58059	59284	96011	76326	75658
Q9CR30	1.0604	1.0031	0.9749	1.0340	1.0166	1.0596	1.0227	0.9649	1.0620	0.9035	0.9210	0.9814
	91854	45439	40345	63393	80286	00182	19911	31496	01712	71284	8579	09393
P54254	0.8917	0.9154	0.9879	0.9681	0.9713	0.9991	1.0520	0.9978	0.9579	1.0674	1.1162	1.0905
	95233	78132	54549	50323	6113	72335	98701	39478	25416	40158	60917	28314
P24452	1.2357	1.0485	1.0718	0.9327	1.0175	1.0732	1.0065	0.9683	0.9639	0.8871	0.8882	0.9317
	22319	06508	54626	35368	69476	10971	76353	4421	91266	38484	42136	73951
P52432	1.0781	0.8492	1.0017	0.9970	1.0180	1.1374	1.0575	1.1335	1.0885	0.9215	0.8439	0.8716
	69187	44906	33408	45871	53569	34983	9109	55704	0177	55042	72525	61312
Q8R3C0	0.9973	1.0182	1.0209	0.9078	0.9608	0.9811	1.0356	0.9987	1.0971	1.0092	1.0423	0.9160
	98371	63852	09993	175	72104	86433	43511	94113	67994	58891	98297	98813
Q8R0K9	0.9913	1.0184	0.9946	0.9967	0.9861	0.9509	1.0020	1.0419	1.0376	1.0425	0.9977	0.9682
	20943	60738	91275	87692	67491	00957	46606	46016	7305	47703	20292	9792
P61211	1.0078	1.0544	0.9899	0.9191	0.9123	0.9472	1.1249	1.0079	1.0343	1.0820	0.9586	0.9976
	24529	15552	4732	41499	1146	81272	24657	07046	89998	41013	31002	43391
O35684	0.9532	1.0330	0.9977	0.9744	1.0422	1.0575	1.0636	0.9882	0.9919	0.9940	0.9294	1.0009
	34085	86548	80277	97647	64521	61713	77547	72816	2643	49873	0119	54286
O70400	1.0895	1.1166	0.9897	1.0549	1.1587	1.0394	0.8898	0.9105	0.9025	1.0111	0.9833	0.9439
	82727	16179	98194	56108	12763	99423	91942	79762	91509	34445	50641	81893
Q9CQ89	1.0293	1.0034	0.9598	1.0133	1.0358	1.0349	0.9991	0.9459	0.9616	1.0513	1.0149	1.0141
	89645	89126	66324	90768	1127	57975	62992	21223	57327	24782	1836	4621
Q99M51	1.0446	1.0281	0.9992	0.9656	0.9423	0.9507	1.0334	1.0541	1.0000	0.8960	1.0299	1.0231
	35804	89252	13642	58649	40802	6173	17891	34386	69867	14469	96581	85005
P47879	0.9989	0.9744	1.0213	1.0204	1.0164	0.9917	0.9972	0.9795	1.0267	0.9368	1.0001	1.0331
	24076	22764	63556	7346	06212	27777	61531	03765	04893	6879	60556	32563
Q5FW52	0.8849	0.9215	0.8824	0.9825	0.9407	0.9989	1.1030	1.0096	1.0235	1.1755	1.0510	1.0902
	08519	66402	28867	83826	50245	02554	03334	29222	84029	76363	28327	75607
Q9R1E6	1.0097	1.0939	1.0638	0.8996	1.1497	0.8870	1.0027	0.9626	0.9601	1.0967	1.0608	0.8615
	74525	69338	43469	38798	58893	71057	71912	48703	87962	67846	45764	18485
O35963	1.0907	1.0222	1.0210	0.8788	1.0135	1.0216	1.0175	1.0251	0.9781	1.0509	0.9615	0.9703
	75253	61901	03173	52232	95474	6824	06748	73516	75156	84453	43114	68855
Q91X52	0.9045	0.8963	1.0274	1.0392	0.9716	1.1324	0.9848	0.9303	0.9315	1.0847	1.0595	1.0955
	40746	07321	21198	08292	09486	66994	25154	73214	92657	56882	95037	22901
Q5DU41	1.1856	1.1101	0.8066	0.8618	1.0505	1.1031	0.8134	0.8839	1.3748	1.4998	0.7964	0.7793
	36563	47312	21221	21508	21725	32335	94789	23317	38378	65623	21589	94699
Q8BK63	0.9603	0.9354	1.0014	0.9761	0.9518	0.9606	1.0551	1.0882	1.0488	1.1062	0.9723	0.9837
	78565	46901	06351	78377	79976	39185	27741	72805	08091	12179	00107	32709
Q8K2M0	0.9250	0.9563	0.9157	0.9930	1.0455	1.0284	0.9705	1.0224	0.9815	1.0989	1.0597	1.0615
	25784	91691	24155	86905	59195	10589	75896	88163	26958	57611	65169	10466
Q8K2C8	0.8909	1.1255	1.0597	0.9001	1.0241	0.8304	1.0102	1.0848	0.9072	1.0654	1.1116	0.9965
	40699	42189	10946	21581	78478	95786	14987	28025	12941	40353	96147	21627
Q6P8M1	0.9455	0.9827	0.9876	1.0030	1.0698	1.0762	0.9428	0.9549	1.0924	0.9421	0.9630	1.0430

	38095	67001	50845	52435	64123	50746	69981	34546	66275	12877	5223	96128
Q8R143	1.1908	1.2237	1.1378	1.1792	0.9401	0.7478	0.7124	0.9809	1.2708	0.6349	0.8985	0.9857
	15803	17256	18466	73423	91423	68128	60678	31761	59307	09245	80336	84692
Q3TPE9	1.0322	0.9292	1.0178	0.9689	1.0547	0.9840	1.0376	1.0036	0.9422	1.2063	0.9636	0.9801
	59654	68262	97386	32423	13248	37226	89188	21974	49611	75815	9609	62842
Q8BQU6	1.2434	1.0913	1.0006	1.0619	1.2027	1.1273	0.8962	0.9079	0.9425	0.8640	0.8871	0.8360
	11803	30805	94451	71886	99451	89628	30722	42817	27783	64638	09565	40164
A3KGB4	0.9478	0.9685	1.0613	1.0484	0.9734	1.0577	1.0334	1.0259	0.9772	0.8275	1.0237	0.9928
	94625	41466	10378	92928	07467	83665	52353	43596	96011	9101	19856	88314
Q9Z1R3	1.0477	1.2201	1.1649	0.5652	0.4161	0.4183	1.8757	1.5974	1.8142	0.5210	0.4955	0.4137
	06156	84384	79185	01162	06107	02002	7046	76735	65727	20712	8665	7469
P01674	1.3057	1.3319	1.2248	0.9020	0.9376	0.8179	0.8506	1.0256	0.9227	1.0578	0.8563	0.8706
	57845	30669	89485	29294	86454	5131	17387	00311	75952	36134	59899	97997
Q8C0S1	1.0628	1.0917	1.0291	0.9963	1.0191	1.0338	0.9444	0.9715	0.9081	1.0171	1.0098	0.9744
	53223	61712	81879	74129	90632	85787	44967	05276	35309	88812	37343	916
Q6GQW0	1.0600	1.0590	0.9738	1.0825	0.9642	0.9956	0.9741	0.9931	0.9914	0.9779	0.9852	0.9799
	3203	31342	9598	52045	45036	31165	30661	01051	32665	26239	42611	63495
Q149G0	1.1368	1.1608	1.0355	0.9263	1.0587	0.9968	0.8934	1.0789	1.0161	1.0558	0.8757	0.8452
	8626	27874	3108	64649	51984	31817	80687	07908	33161	38956	43119	18766
Q99JG2	0.9110	0.9710	1.0389	1.0795	1.1176	0.8944	0.8685	0.9406	1.0496	0.7287	1.1612	1.1301
	58658	54972	56792	14783	09059	37077	74168	76636	7589	86321	96737	86055
Q61474	0.9596	1.2698	0.9958	0.9965	0.9910	0.9771	0.9704	1.0213	0.9195	1.0941	0.9796	0.8989
	15566	83535	49673	52462	08354	87812	85793	07812	79307	32793	06405	1651
Q8C6G8	0.9701	1.0383	1.0341	0.9956	1.0732	0.9483	1.0220	0.9553	0.9553	1.0626	1.0025	0.9948
	66229	89002	67558	59546	24503	86718	44836	37226	40083	69313	82928	31014
Q9WUH1	1.1074	1.2436	0.9400	1.0223	0.9206	1.0398	0.9717	0.9999	0.9668	0.9894	0.9216	0.9377
	95574	41153	60661	7632	99609	29519	27782	77307	48186	94459	34037	24685
Q9ESP1	0.9393	0.8242	1.0567	0.9803	0.8732	0.8363	1.0085	1.1714	1.1057	0.8701	1.0619	1.1791
	43281	50054	93297	26303	63751	03889	36001	03808	3386	39003	80315	01746
Q8BGN5	1.0048	0.9546	0.9623	0.9445	0.9134	0.9208	1.0929	1.0560	1.0210	1.0586	1.0657	1.0154
	7747	83657	45784	09644	207	20799	74606	50698	9338	60897	78779	79821
Q8K448	1.1117	1.0283	1.0804	1.0335	1.0304	0.9412	1.0057	0.9524	1.0454	0.8848	0.9336	0.9460
	42755	52368	25763	65079	71539	45584	62558	81264	43361	22361	00307	04317
P98191	1.1349	1.1169	1.0526	0.8436	1.0355	0.8561	0.9222	1.0063	0.8657	1.1817	1.1430	0.9376
	96842	31324	43105	39379	36396	56983	02178	02097	2941	37433	59297	00473
Q3UP75	1.1129	1.1877	1.0636	0.9997	0.9976	0.9421	1.0334	0.9342	1.1053	0.7121	0.9012	0.9266
	80255	11218	95059	2315	36069	18346	96906	10527	52331	78592	6881	69588
P09470	1.3123	0.9900	1.2654	0.7159	0.7680	0.8202	1.1427	1.1477	1.2474	0.7870	0.9099	0.7494
	10633	51379	41388	82189	85429	31751	18598	05767	66024	0572	30514	96686
Q8CG03	1.0222	0.9902	0.9483	0.9104	0.9962	0.9537	1.0974	1.0339	1.1053	1.1025	0.9388	0.9448
	28519	65807	93136	91117	70256	72669	97849	66599	49787	85972	83533	67978
O09117	1.0260	1.0398	1.0324	1.0936	1.0993	1.0224	0.9381	0.9369	0.9885	0.9276	0.9212	1.0108
	18222	7389	50079	76481	63631	80929	64549	95789	34314	4979	31991	98185
Q9D071	1.0867	1.1349	1.1293	0.9106	0.8553	0.8903	1.0627	1.0653	1.1039	0.8374	0.9199	0.9294

	29577	78547	00038	58948	68949	90558	50588	02271	88459	79893	40314	1889
Q6PDN3	1.2164	1.0255	1.0488	0.9417	1.0136	1.0038	1.0102	1.0212	0.8834	1.0054	0.9738	0.9186
	19664	44086	22623	67946	81763	96978	76813	70781	86592	35113	76274	84271
P70170	0.9402	1.0329	0.9572	0.9649	0.8980	0.9066	1.0269	1.0417	1.0218	1.0327	1.1345	1.0312
	73735	71946	26734	21706	83932	34099	50716	67397	78044	79099	71126	06686
Q924Z6	0.9459	0.9793	1.0706	0.9870	1.0165	0.9454	0.9734	1.0030	0.9695	0.9727	1.0880	1.0378
	03219	99664	663	14658	45458	76419	36693	89423	27799	36028	01861	67575
P28662	0.9526	0.9586	0.9866	1.0051	1.0440	1.0134	1.0747	0.9654	0.9825	0.9753	1.0361	1.0073
	34915	93745	27803	97878	53607	09501	51962	68329	70579	67933	84555	20542
O35657	0.9784	1.0650	1.0043	1.0230	1.1171	0.9416	0.9463	0.9667	1.0439	1.0299	1.0355	0.8778
	0675	87837	6987	01116	95892	06096	41992	83953	69854	93851	75323	7163
Q99MU3	0.9948	1.0015	0.9583	0.9634	1.0078	1.0351	1.0210	1.0106	1.0208	1.0996	0.9822	0.9662
	18314	58794	48924	3258	6484	87902	12602	00644	44395	64845	78794	80857
Q9CRC0	0.9612	0.9679	0.9939	1.0294	1.0671	0.9653	0.9700	0.9995	0.9630	1.0825	1.0898	0.9578
	94694	49335	72315	66666	3365	5889	81625	18338	1909	04015	07557	53446
Q8CI59	0.9773	0.9662	0.9268	0.8948	0.9588	0.9094	1.0630	1.1038	1.0750	1.0871	1.0726	0.9725
	41399	38174	79172	63523	64147	04016	10539	40409	62921	38292	72053	72309
Q3V4B5	1.0038	1.0764	0.9403	0.8867	1.2048	0.8285	0.9761	1.0047	0.8050	1.5742	1.1574	0.7986
	9854	11984	24757	44514	15285	42544	60107	80753	34456	64507	97257	85014
O70209	1.0528	1.0499	1.0437	1.0660	1.0261	1.0085	1.0307	0.8906	0.9228	0.9491	0.9865	1.0066
	51444	76196	80803	76812	60189	09663	59772	57387	66249	19686	8603	59136
Q60953	0.9972	1.0281	0.9637	0.9263	1.0960	0.9995	1.0328	0.9894	1.0133	1.0608	1.0023	0.9331
	245	84167	05925	45038	62841	31781	06128	7614	23729	87059	26525	35735
Q3UZ39	1.0471	0.9940	0.9434	0.9943	1.1889	1.0628	0.9667	1.0009	0.9792	1.1725	0.8857	0.9049
	25589	72752	64051	48414	1191	59682	08502	81442	69418	59651	46508	23799
P70158	0.8667	0.9162	0.9040	0.9629	0.9462	0.9819	1.0902	1.0580	1.0330	1.0362	1.0877	1.1070
	93689	77239	65326	2574	34584	54403	39069	75612	20657	01876	17448	82544
Q8BYK5	0.9953	0.9740	0.9915	1.0351	1.0263	0.9896	0.9844	1.0568	0.9814	1.0503	1.0152	0.9428
	32398	84328	58011	36594	80083	02314	75045	28468	13184	75263	77892	14138
Q8VHI3	1.0076	1.0106	0.9857	0.8789	0.9267	0.9694	1.0558	1.0666	0.9909	1.2257	0.9583	1.0267
	22833	65186	83743	42581	78419	94708	12144	85777	62228	39757	49902	84923
Q8R554	0.9841	1.0024	0.9879	1.0237	0.9872	0.9726	1.0360	1.0025	1.0199	0.9338	1.0160	1.0203
	89226	83931	29038	22646	10214	47677	25183	86998	3674	23297	55249	84234
A6H5Y3	1.0261	1.2375	1.0503	1.0687	0.9291	0.8640	0.9768	1.0922	0.8957	1.0128	1.0692	0.7977
	82115	22921	23101	01492	5506	20857	44199	23663	17448	70226	89962	35643
Q6ZQA6	0.8581	0.8382	0.9860	1.0119	0.9102	1.0024	1.0279	1.0616	1.0628	1.1096	1.0552	1.0976
	34341	1895	00886	22344	46189	73633	08504	06903	95015	83597	86809	14973
Q920N2	0.9661	1.0303	0.9748	0.9939	1.0301	0.9624	0.9608	1.0836	1.0296	1.0935	0.9813	0.9479
	54433	83175	37887	16654	77076	54958	99148	33377	09634	43646	91478	98552
Q8BWU8	0.9800	1.0521	0.9261	0.9542	1.0231	1.0525	1.0525	1.0383	1.0379	1.1380	0.9039	0.9257
	08336	6956	84405	96791	25245	1891	19284	75245	04043	84528	9778	67794
P62838	0.9433	1.1094	1.1077	1.1770	0.9971	1.0928	0.8956	0.9392	0.8587	0.9172	1.0936	0.8753
	31107	32733	48839	02224	63883	66675	98472	02292	41999	14109	56601	29001
Q9CWU9	0.9485	1.0178	0.9924	1.0474	0.9901	1.0610	0.9897	0.9503	0.9513	1.0163	1.0147	1.0622

	04788	12918	47802	02438	71994	0046	54136	02534	57401	65438	89066	38448
Q8BJ64	0.9180	0.8783	0.9064	0.9417	0.9707	0.9501	0.7223	0.9177	0.7985	0.8045	2.1399	0.8259
	31541	54587	48668	51808	71222	56014	95328	35533	07644	8316	14784	34291
Q61409	1.1064	1.0022	1.0753	1.0386	1.0772	1.0989	0.9858	0.9119	0.9590	0.8320	0.9640	0.9393
	86923	74221	92458	07795	41158	22434	38457	65539	85199	76787	93669	43757
P61971	1.0496	0.9842	1.0292	1.0068	0.9806	0.9952	1.0193	1.0041	1.0114	0.9780	0.9541	1.0100
	0066	23939	43491	98057	49781	2149	31521	9132	12465	55467	79384	10167
Q8K353	1.0324	1.0087	1.0046	1.0588	1.0373	0.9897	0.8844	1.1671	0.9426	0.9501	1.0065	0.9349
	72882	35808	5796	58999	84729	88191	97678	5577	47886	12982	26127	86507
Q61660	1.1659	1.1130	1.0480	0.9623	0.9716	1.0197	1.0423	1.0273	1.0248	0.8978	0.8659	0.8675
	42607	7426	47504	74129	01485	58558	93259	89262	90858	01008	66643	28896
Q8BH50	0.9843	0.9359	1.0022	0.9950	1.0531	0.9384	1.0316	1.0263	1.0699	0.9377	1.0165	0.9844
	11117	53766	33977	87287	90381	243	01669	73147	23944	00737	38379	63364
Q6P2K6	0.9367	0.9280	0.9614	0.9907	1.0636	0.9907	1.0419	0.9818	0.9615	1.1985	1.0540	0.9847
	11025	34919	19583	49454	44426	63533	789	31213	30853	16052	319	01638
Q6P5B0	0.9471	0.9723	1.0041	1.0039	0.9675	0.9403	0.9805	0.9987	0.9626	1.0201	1.0556	1.1722
	68855	42092	15227	88152	78004	02834	626	41873	25238	28034	91178	74805
P70274	1.1191	1.2154	1.3502	0.8324	0.8467	0.7239	1.0536	1.1673	1.2228	0.7297	0.7808	0.8113
	46348	58661	34808	74162	01906	23093	84971	5792	02174	14592	27863	2107
P27048	0.9535	0.9752	0.9834	0.9798	0.9799	1.0440	1.0141	1.0856	1.0108	0.9199	1.0109	1.0156
	48164	2463	57852	89641	26948	55341	69735	12937	29079	13416	7199	22545
Q5PR68	1.1147	1.1025	1.0715	0.9612	0.9653	0.8747	1.0634	0.9368	1.0191	1.0131	0.9511	0.9602
	53208	43369	70969	89283	03351	19213	15258	53363	22264	08502	93869	79288
Q8C0Q2	1.0069	0.9914	1.0242	0.9878	1.0576	0.8769	0.9943	1.1724	0.9277	1.0651	1.0057	0.9317
	4372	1912	25852	42799	72836	93125	7894	73283	20683	67113	44984	56794
Q9DBU3	0.9774	0.8979	0.9902	1.0001	0.9869	1.0332	1.0699	0.9408	1.1380	1.0447	0.8511	1.1168
	26041	51407	63729	32788	77892	90464	96073	71034	94295	71994	9785	48397
Q8BR65	0.9348	0.9574	0.9567	0.9579	0.9812	1.0880	1.0707	0.9795	1.0504	0.9877	0.9796	1.0597
	96039	48587	50778	03642	67801	29201	04757	2304	54031	6027	27212	78264
O35955	0.9791	0.9961	0.9723	0.9374	0.9426	0.9567	1.0896	1.0715	1.0015	1.0996	1.0058	0.9855
	87772	53195	25974	21362	37644	57743	80545	05531	2365	63479	81728	54191
A2AJL3	1.0788	1.0210	1.0318	1.0471	1.0976	1.0708	0.9909	0.8977	0.9593	0.8836	0.9861	0.9453
	72426	12107	00527	11041	25585	49222	83524	79082	06083	36058	48302	17145
Q8CGE9	0.9242	1.0071	0.9903	0.9495	0.9719	0.9330	1.1085	1.0839	0.9976	1.0755	0.9899	0.9907
	56944	16049	91485	32817	96812	18754	66745	93443	20031	86933	20205	01373
P62843	0.9827	0.9644	0.9891	0.9965	1.0485	1.0283	1.0370	1.0304	1.0175	1.0358	0.9502	0.9577
	55797	1972	14535	7955	62099	0968	84993	91544	26833	17122	33364	60497
Q571F8	0.8760	1.1494	0.9843	1.0739	1.0818	0.8614	0.8537	1.0273	1.0667	0.7831	1.0865	1.0732
	54041	50085	7738	12598	20099	37038	17995	11692	11591	8791	49006	90165
O54990	1.0220	0.9868	1.0085	1.0044	1.0060	1.0186	1.0009	1.0380	0.9949	1.0227	0.9785	0.9545
	00574	90564	11106	69089	80746	36505	90232	05594	30541	93364	73348	53228
Q925T6	1.4527	1.2324	0.8867	0.8100	1.3445	1.1502	0.7388	0.7666	1.0677	1.2955	0.7929	0.7479
	4516	92446	57362	93126	25147	57757	04549	86656	57912	05147	39773	94664
Q61739	1.0923	1.0705	1.1345	0.9903	0.9556	0.8300	0.9360	1.0608	1.0955	0.8333	0.9808	0.9583

	62847	2081	06803	15885	25609	88564	11243	62248	37499	54246	17746	29148
P41778	0.9949	0.9506	0.9914	0.9608	1.0391	1.0779	1.0259	0.9453	1.0064	0.9597	1.0101	1.0454
	16577	34704	05396	29244	57147	59323	34317	45638	40541	03017	4667	67605
Q9CZ69	0.9172	1.1825	0.9695	0.9417	0.9956	0.9932	1.0466	0.9549	0.9631	1.0262	1.0186	1.0158
	31599	50554	48413	57228	38635	27641	72633	17293	33423	45833	50176	16138
Q60857	1.0353	0.9832	1.0508	0.9831	0.9650	0.9653	1.0436	1.0585	0.9393	1.0700	1.0068	0.9439
	61371	82809	19676	4621	68729	23185	33285	3387	44369	78622	58016	56981
Q8VI59	0.7680	0.8976	0.9571	0.8974	1.6973	1.0153	0.7642	0.8818	0.8559	0.7857	1.1767	1.2698
	80813	46247	54767	96131	3518	85523	37062	84927	50678	4433	1181	70923
Q9CWW6	1.1069	1.1097	1.0800	1.0904	0.9989	1.0768	0.9771	1.0006	0.9779	0.8323	0.8614	0.8886
	14609	66197	96285	80727	15622	1593	21751	87295	12238	6733	055	17863
P42337	0.9588	0.9746	0.9744	1.0237	0.9357	0.9795	1.0026	0.9601	1.0047	1.0113	1.0790	1.1094
	8278	82216	93254	64566	58427	74857	34567	19238	16457	72051	56763	91383
Q6NS82	1.4275	1.1077	0.5952	0.5879	1.1879	1.9293	0.5705	0.6311	1.4323	2.1375	0.5330	0.5197
	1874	08164	93365	35959	03203	69912	66899	52394	18314	88357	8339	78626
Q3U1V8	1.0089	1.0065	0.9570	0.9997	0.8898	0.9899	1.0114	1.0691	0.9877	0.9854	1.0682	1.0259
	27661	83177	91662	57294	03209	63617	69664	02726	71279	70833	94307	84496
Q3U308	0.9525	0.9758	0.9524	0.9649	0.9698	1.0032	1.0424	1.0010	1.0151	1.1275	1.0216	1.0329
	70336	16963	72057	37697	74202	19379	93958	88783	37623	75311	92429	58821
Q80WV7	1.0254	1.0852	0.9088	0.9198	1.0547	0.9097	0.8745	0.8552	0.9594	1.1056	1.3942	0.9331
	60163	70287	08657	5253	39374	5478	28899	9051	61609	93146	12517	04078
Q9WV68	0.9791	0.9858	1.0316	1.0294	0.9794	1.0359	1.0303	1.0050	1.0082	1.0234	0.9443	0.9826
	63764	00196	53667	96152	36696	59429	36159	32681	43247	03258	80402	42872
Q9D2R0	1.1008	0.9562	0.9672	0.8114	1.1016	0.9197	1.1583	1.0673	0.9094	0.9658	1.1124	0.9086
	43314	68395	9215	7635	26515	0047	78471	10174	38628	1125	57196	44084
Q3UGR5	1.1138	1.0101	1.0147	1.0169	1.0563	1.0881	1.0096	0.9959	0.9957	0.8915	0.8947	0.9290
	34011	57467	51922	11966	12563	48156	23015	7216	79451	39061	36679	30522
Q62179	1.2863	1.0941	0.8896	0.8934	1.1230	1.1127	0.8453	0.8531	1.1416	1.4093	0.8418	0.7765
	20291	81327	59617	5867	50188	90599	39107	16119	38427	30104	02903	39277
P62257	0.9062	0.9450	0.9687	0.9864	0.9437	0.9883	1.0300	1.0301	1.0429	0.9422	1.0517	1.1331
	39067	08293	33089	95254	01646	11309	03498	66483	20871	39768	69313	86845
Q6P6M7	1.0756	1.0453	1.0412	0.9749	1.1728	0.9363	0.9940	1.0486	0.8771	1.0044	1.0046	0.8721
	06186	78876	75659	61324	11098	84114	28221	00153	39912	59238	40359	0832
Q8BG21	0.9929	1.1181	1.0234	0.8244	0.8958	0.9100	1.0528	0.9658	0.9887	1.3401	1.0654	0.9466
	49176	95834	6592	47997	01726	30953	11267	32655	1766	97452	74516	60582
Q8BTW3	0.8691	1.1969	1.0086	1.0086	0.8319	1.0295	0.9932	0.9768	0.9752	0.9431	1.0566	1.0878
	7757	82114	40974	63792	86764	1477	57384	27136	23181	96722	07453	78259
Q78RX3	0.9543	0.9427	1.0305	1.0881	1.0134	1.0343	0.9898	1.0127	0.9803	0.9415	1.0136	0.9985
	31187	21841	44278	05796	20318	48692	74282	44518	44735	35	07096	3202
Q9QY33	1.0574	1.0180	1.0386	0.9742	1.0249	1.0045	1.0074	1.0382	1.0291	0.9590	0.9256	0.9366
	00939	87788	84883	22983	33719	40631	33746	6681	02016	8547	73469	77
Q80YE7	0.9838	1.1135	0.9423	0.9941	0.9735	0.9977	0.9727	0.9816	0.9522	1.0557	1.0734	1.0061
	60423	80762	25498	34657	48659	90307	25666	40713	44144	66034	75912	74783
Q99KN2	1.4942	1.1597	0.7632	0.8234	1.3637	1.1173	0.7631	0.7481	1.1253	1.6239	0.7446	0.7080

	67281	4879	81379	21181	04299	49179	08204	2765	68338	67251	86903	737
Q6PDJ6	0.9890	0.9741	0.9855	0.9800	1.0511	1.0206	0.9707	1.0253	0.9875	1.0518	1.0166	0.9928
	47799	74214	93935	4885	0995	54703	24988	66986	78566	02009	43613	73743
P63166	0.9553	0.9924	0.9832	0.9791	1.0355	1.0212	1.0333	1.0359	0.9476	1.0717	1.0327	0.9548
	74077	30534	83619	17096	91435	69985	94989	67195	96812	70008	36495	13514
P39429	0.8304	0.7922	1.0554	1.0604	1.0435	0.9449	1.0191	0.9293	1.0958	1.1843	1.0427	1.0604
	78663	73011	20165	4075	34241	4501	41885	37099	7342	09374	25739	99653
Q80W47	1.0092	0.9699	1.0217	0.9455	1.0030	1.0071	0.9811	0.9848	0.9584	1.0512	1.0481	1.0619
	79071	06737	73896	09225	01734	76123	14105	15257	42669	78021	97348	39866
Q6PFX7	0.9635	0.9371	0.9591	1.0560	0.9872	1.0276	1.0219	1.0472	0.9989	1.1496	0.9464	0.9973
	3288	00658	8737	51358	74638	42831	38419	83632	23419	9869	2511	41913
Q9Z1X2	1.0360	1.0685	0.9895	0.9759	0.9663	0.9784	0.9987	1.0146	1.0425	1.0466	0.9772	0.9453
	16451	38191	16233	48522	00915	7518	06905	71703	29733	23019	04604	88459
A2AHL1	0.9889	1.0208	0.8814	0.9552	1.1101	1.1102	0.9445	0.9406	1.0742	1.1277	0.9696	0.9697
	96893	89652	62067	31598	83308	73998	68407	94862	70588	39906	07453	63251
Q8K2G4	0.8385	0.8667	0.9610	1.0910	1.1572	0.9439	1.0100	1.1939	0.9343	0.8565	1.1369	0.9342
	41146	01039	88711	95669	80501	62227	78266	55624	7036	21832	7255	58868
P50428	1.0592	1.0353	1.0016	0.9195	0.9970	0.9994	1.0083	0.9883	0.9751	1.1144	1.0033	0.9723
	59645	4708	67793	58987	42953	30521	35815	46911	39565	19513	31252	02189
E9Q9D5	1.0862	1.0586	1.0640	0.9803	0.9408	0.9731	0.9849	1.0462	0.9699	1.0257	0.9502	0.9658
	90216	6278	78931	02704	65126	31156	03277	98378	29465	6897	98231	46986
Q9CU65	1.2729	1.0745	0.8610	0.8175	1.0713	1.0930	0.8946	0.9038	1.1404	1.4767	0.8487	0.8224
	51417	69833	26815	63868	21415	25683	71976	21224	77807	20486	48795	01504
Q923D4	0.9650	1.0121	0.9829	1.0161	0.9950	1.0067	1.0211	0.9697	0.9850	1.0474	1.0411	0.9934
	85836	90969	78887	93432	40689	06702	46089	28617	94849	56736	48269	87543
P49817	1.2344	1.0730	0.8637	0.9486	1.1437	1.1238	0.8806	0.9260	1.0358	1.2865	0.8613	0.8458
	49375	66438	22588	30537	29412	38863	81684	19192	94006	02223	43579	77925
P62305	0.9810	0.9554	1.0274	1.0586	1.0094	0.9894	0.9900	1.0046	1.0101	0.9054	1.0284	1.0190
	13258	62096	613	17736	15508	80951	26511	32616	84971	26288	34825	00555
Q3V140	0.8587	0.9208	0.9511	0.9891	1.0076	1.0559	1.0621	0.9280	0.9691	1.0003	1.0922	1.1673
	17694	08259	68744	58686	61352	93914	41133	24767	71374	02882	19506	12795
Q3TZX3	1.1438	0.7943	0.9437	0.9229	0.9047	1.0172	1.2400	1.0854	1.0318	1.1221	0.8825	0.9796
	9492	45345	04172	05799	14265	27084	97604	41589	25032	81394	27631	96973
O88427	0.9395	0.9572	0.9030	1.0084	1.1468	1.0389	1.0142	0.9444	0.9693	1.0561	1.1054	0.9580
	41326	46825	54667	36718	78671	77661	6328	63307	43364	65394	88436	25197
P97930	1.0382	1.0773	0.9598	0.9620	1.1221	1.1022	0.8778	0.9862	0.9118	1.1218	1.0052	0.9485
	47698	56631	59016	78712	25071	21026	85624	94904	18518	82192	75675	55328
O70404	1.1289	0.9794	1.0184	0.8560	1.0178	1.1168	1.1359	1.1509	0.9675	1.0236	0.8599	0.7820
	15866	6629	16261	92057	9025	74353	07244	78503	82393	17818	14634	98729
Q3URS9	1.0206	1.1648	1.0695	0.9570	1.0402	0.8560	0.9576	1.1560	0.9871	0.9543	1.0188	0.7991
	59987	07376	8431	45802	2611	31076	16492	57878	47303	99854	92817	4452
Q8K4L6	0.9631	0.9205	0.9808	0.9556	0.9982	0.9719	1.1036	0.9265	1.0629	1.1952	1.0350	0.9584
	2597	76127	78515	5898	64815	53394	99887	23378	33449	48347	32177	79711
Q9DC04	1.0714	1.0137	1.0194	0.9832	0.9376	0.9463	1.0961	1.0287	1.0335	0.9824	0.9168	0.9865

	35668	58504	85036	61988	01831	14835	16095	97574	83546	91231	21113	81473
Q5ND34	1.0927	1.0431	1.0051	1.0426	1.0762	1.0047	0.9320	0.9955	0.9622	1.0565	0.9150	0.9686
	52423	11604	36456	49473	86243	46983	44815	7511	94788	67553	97314	77609
Q8K3A9	0.9142	0.9590	1.0506	1.1634	0.8957	0.8822	1.0619	1.2567	1.0184	0.9723	0.8962	0.9177
	98458	82355	50371	67244	60197	39047	08769	94833	45358	67865	82426	88298
Q08879	0.9879	0.9483	1.0039	1.0184	1.1299	0.9696	1.0552	1.0248	0.9897	0.9787	0.9948	0.9095
	53426	3137	44888	21717	68539	21684	39586	60231	13339	05141	88767	09635
Q7TMY7	1.0242	1.0051	1.0034	0.9779	0.9391	1.0065	1.0633	1.0109	1.0203	1.0359	0.9613	0.9844
	8487	99826	05076	65874	88866	86374	48143	98254	55581	73786	28385	20183
Q8BGN2	1.0263	0.9783	1.0579	1.0331	1.0435	1.1287	0.9579	0.9930	0.9571	0.9314	0.9395	0.9773
	9612	3994	03605	97976	78226	3699	71428	61158	52636	68219	38569	32093
Q8BIL5	0.9830	1.0266	0.9406	1.0044	1.0994	1.0457	0.9460	0.9913	0.9855	1.0780	0.9908	0.9835
	85228	0428	68722	79419	058	51039	40917	73283	37739	31489	81082	70454
Q9CWG8	0.9453	1.0716	0.9964	0.9551	1.0204	0.9861	1.0394	1.0741	0.9114	1.1886	0.9634	0.9491
	39503	4568	32905	32051	32506	04062	30646	92975	05596	52017	67116	74429
P42866	1.0200	1.0514	1.0346	0.9828	1.0800	0.9122	0.9007	1.0227	0.9308	1.1106	1.0863	0.9378
	50826	404	25409	06066	2989	01535	71875	50376	42258	455	0391	59098
Q8VDZ4	1.0285	0.9598	0.9913	1.0191	0.9151	1.0151	0.9971	0.9853	1.1278	1.0093	0.9529	1.0189
	00984	2025	93618	66495	01735	71213	08391	56332	9839	55248	10008	34044
Q8CB67	0.9534	0.9383	0.9943	0.8968	0.9579	0.9045	1.0475	1.2051	1.0757	1.0207	0.9784	1.0113
	16507	28898	23912	17905	93837	09016	47344	17856	38961	98517	30843	63403
Q8K2P7	1.0038	0.9490	0.9649	1.0416	0.8929	1.0957	1.0462	0.9833	1.0086	1.1661	0.9582	0.9875
	70426	64208	69949	64727	63441	94654	11456	17679	5073	1963	09592	59605
Q9JLK7	0.9187	1.0435	0.9748	0.9785	1.1565	1.0257	1.0151	0.9998	0.8330	1.2337	1.0622	0.8850
	60969	88519	81934	15353	5452	79199	81597	1839	16238	32714	55682	0488
Q61214	0.8677	0.9644	1.0330	1.0660	0.9477	0.8802	1.0015	1.1400	1.0421	1.0055	0.9917	1.0510
	53121	48997	96568	07684	08471	66788	20572	84789	42429	5091	16428	57918
Q6PE01	0.9941	0.9276	1.0177	0.9539	1.0484	0.9432	0.9557	1.0521	0.9553	1.0877	1.0841	1.0257
	3214	1227	88016	79888	8356	2833	01098	83843	5019	60455	13334	02552
O54940	1.0537	1.0435	1.0060	1.0076	0.9910	0.9474	0.9729	1.0692	0.9854	0.9305	1.0380	0.9456
	76058	63799	67576	46921	12485	71323	69675	78106	58895	06746	00269	27791
P97372	1.0128	0.9377	1.0365	1.0630	1.0639	0.9629	1.0237	1.0134	0.9991	0.9658	0.9769	0.9582
	21794	24132	62755	3399	60823	47985	80362	40269	02688	77166	28526	42409
Q61205	1.0403	1.0615	1.0879	1.0010	0.9946	1.0191	0.9891	1.0097	0.9939	0.9720	0.9369	0.9148
	59236	94909	7592	9759	01666	9221	03008	42024	84962	76716	49556	3402
Q922J9	1.0156	0.9768	1.0239	0.9780	0.9657	0.9594	0.9940	1.0206	1.0379	1.0208	0.9974	1.0313
	30502	65078	86807	8311	8651	74018	95079	11387	88155	32899	83461	91271
P56671	1.2019	1.1328	0.9097	0.9016	1.1596	1.2095	0.7874	0.8890	1.1146	1.4112	0.7885	0.7724
	45718	3333	15597	80138	50153	52127	22638	07932	1788	17689	16256	80615
Q61210	0.8652	0.9689	1.0238	1.0045	1.0872	1.0225	1.0051	0.9551	0.9674	1.1070	1.0322	1.0165
	06889	3099	36917	72413	21471	93965	71267	95155	88703	60753	71623	21406
Q8CDV6	1.0661	1.3265	1.1116	0.7637	0.8144	0.9045	1.0062	1.0517	1.2637	0.9795	0.8316	0.8482
	63015	85809	54673	50316	51318	55294	00208	61952	2628	69617	45002	59726
Q61239	1.0465	1.1505	0.9272	0.9788	0.8512	0.9786	1.0527	0.9953	0.9551	1.0304	1.0770	0.9834

	75083	41267	82691	9692	75905	90324	23101	52624	46176	77167	26806	08937
P61963	1.0102	1.0106	1.0259	0.9628	0.9974	0.9188	1.0055	1.0981	0.9269	1.0201	1.0572	0.9834
	13399	67777	94208	18997	72283	38495	39968	63116	12524	34747	09574	49482
Q99NH2	1.0126	0.9787	1.0402	0.9182	1.0192	0.7524	1.0331	1.4973	1.0265	1.1150	0.8458	0.7914
	41926	09782	98382	06188	11495	94917	73159	25494	86574	64203	62142	57474
Q8CDG1	0.9357	1.0887	1.0925	1.1504	1.0371	0.9209	0.8746	0.9956	1.0365	0.6891	1.0309	1.0446
	25553	20594	65025	44842	73226	32105	12537	70359	01543	72996	87334	90481
P60853	0.9837	1.0092	0.9339	0.9764	1.1123	1.0237	0.9173	0.9879	0.9865	1.0491	1.0417	1.0345
	62797	88549	63216	76676	98691	91771	31504	86338	09933	82649	62746	48681
Q78T54	1.0089	0.9740	1.0613	1.0158	1.1829	0.9641	0.9269	0.9698	0.8971	1.0503	1.0475	0.9660
	57762	46187	50285	52445	40308	37443	45847	72505	10248	94782	48412	78214
Q8VDB8	1.0117	0.8928	1.0803	0.9318	1.1296	0.9569	0.9919	0.9788	1.0061	1.1052	0.9775	1.0027
	65919	22341	09661	03122	99671	52833	50678	62418	72637	13799	65541	21702
Q91X51	0.8894	1.1676	0.9044	0.9017	1.1806	0.8869	1.0265	0.9660	0.8906	1.5179	0.9285	1.0004
	53352	55681	84509	50801	93766	58858	05774	58777	06746	31088	79881	03788
Q5SSH7	1.0968	1.1066	1.0288	1.0037	1.0477	1.0186	0.9270	0.9765	0.9760	0.9090	0.9885	0.9341
	30829	84195	73994	69655	63274	90196	87247	76671	54139	36775	67549	02311
Q9CQC8	0.9595	1.0337	0.9684	1.0113	0.9458	0.9585	1.0025	1.1022	1.0823	1.0253	0.9981	0.9173
	50765	83777	7346	10692	49126	53895	65177	55457	11303	9819	95365	30012
Q9R0S3	0.9735	1.0103	1.0513	1.0476	1.0052	0.9223	1.0178	1.0026	0.9657	1.0146	1.0185	0.9939
	00206	55632	56349	97848	23044	36886	28345	84937	74869	52699	66953	43012
O70496	1.0881	1.0201	0.9691	0.9247	1.0673	1.0694	0.9071	0.9889	1.0800	1.1571	0.9152	0.9254
	74272	59328	04735	86499	65151	28614	33035	49704	80981	84913	91419	78212
Q03958	0.9692	1.1921	1.0672	1.1327	0.8580	1.0309	0.8683	0.9285	0.9822	0.7300	1.0955	1.0622
	41166	32243	54112	40651	0937	44645	15498	30186	19111	47086	86107	30125
Q9CWQ0	0.9801	1.0071	0.9923	0.9515	1.0129	0.9814	1.0792	1.0506	0.9299	1.1355	1.0149	0.9310
	27656	61987	28082	1582	51341	73379	94114	72024	04713	13365	89845	72814
Q8BHY8	1.0371	0.9537	1.0068	0.9991	1.0054	0.9592	1.0717	0.9777	0.9520	1.0559	1.0107	1.0201
	11722	21944	43446	07284	38703	48488	54273	94176	38853	08271	27856	75994
Q9QXM1	0.9185	1.0871	0.9421	1.0339	0.9733	0.9572	0.9276	1.1601	1.0250	1.2090	0.9337	0.9391
	90009	96525	92352	17708	8791	54763	22359	44711	35002	04835	24463	14086
O55028	1.0336	0.9212	0.9835	0.9889	1.0276	1.0819	1.0761	0.9373	1.0535	1.0311	0.9163	0.9970
	68369	92403	5527	68181	4195	61838	15968	70694	04992	11596	52777	04847
Q00899	1.0086	1.0100	0.9903	1.0245	0.9816	1.0451	0.9870	1.0247	0.9652	1.1443	0.9413	0.9797
	45768	39009	75982	47859	64352	10039	90886	05142	5929	72917	1917	49911
Q8VEB1	1.4966	1.4159	0.6556	0.6415	1.4881	1.1840	0.6688	0.6700	1.3236	1.5258	0.6851	0.6390
	01322	79695	76455	34877	08364	19262	90902	50753	62006	03932	93379	36977
Q640M6	0.8841	0.9569	0.9746	0.9696	0.9483	0.9108	0.9636	1.1431	1.1023	1.3283	1.0399	0.8805
	50766	29503	59977	16894	82479	08395	66478	56745	15748	69362	57087	43629
Q80V26	1.0149	1.0137	0.9547	0.9738	1.0628	0.9438	1.0007	1.0305	0.9983	1.2070	0.9752	0.9386
	19889	44003	18585	52874	1849	07648	21718	05646	56998	69848	62393	92516
P48543	0.8966	1.0332	0.9509	0.9778	1.0945	0.9416	1.0393	1.0752	0.9605	1.1312	1.0236	0.9345
	55851	658	64243	53696	32621	62268	28322	89085	75447	71853	48343	03159
Q9D1E8	1.0034	0.9609	1.0350	0.9141	0.9565	0.9024	1.0912	1.0939	1.0402	1.0639	1.0170	0.9269

	34891	36903	87115	21382	41958	62888	18997	33183	09417	61104	49016	73864
Q80W22	1.105154784	1.103688175	0.990689031	1.08123365	1.074337015	0.98822576	0.895083494	0.87008558	1.007488348	0.837312312	1.003596457	1.047129284
Q61247	1.148664815	1.229777786	1.245498442	0.763035907	0.861986884	0.762093953	1.130646652	1.08761487	1.373746786	0.782802801	0.795704012	0.66945701
E9Q942	0.890940017	1.024058899	0.934332665	1.041955255	1.018593957	1.00643596	1.109382434	1.095652065	0.946694525	0.997652661	0.99883415	0.942296053
Q9JJI8	0.966129093	1.082501715	1.019464873	1.025923254	0.992056386	1.029343941	1.001496437	1.031917697	0.987645818	0.796005853	1.007220144	0.995847986
Q9JK23	0.974166556	0.99574095	1.044693327	0.984076155	0.998903894	0.977714032	1.050441981	1.010791748	0.922143544	0.997786574	1.026842608	1.032603949
Q8BTN6	0.958560069	0.968102057	1.022634119	0.933660927	1.000725566	0.97512948	1.069580768	0.990885303	1.142254333	1.008746914	0.981136554	0.934823975
Q99PV8	0.888196309	0.89638451	0.981616474	1.004927359	0.955299568	0.994773489	0.981805429	0.99922489	1.042842981	0.941069111	1.126388586	1.147811253
P14069	1.133432519	1.316223066	1.333546813	1.105769076	0.958573159	1.214586476	0.744050669	0.947274738	0.884620676	0.618399204	0.904167111	0.77096365
Q80W03												
Q9QYM9	1.042444684	1.036366585	1.025173588	1.056472816	0.997526198	0.938514187	0.99966923	1.036273255	1.059273905	0.907832898	0.945227189	0.944314581
Q6QI06	0.982593366	0.954269517	0.936090036	1.049790126	0.970151039	1.025366932	1.080922929	0.996292054	1.012420541	0.978065424	0.985606041	1.043540473
Q62010	1.187176308	1.006572481	1.056553576	0.908250836	1.080699374	0.999652368	1.07074621	1.045484133	1.082925206	0.877372193	0.86817327	0.799249687
Q69ZP3	1.007939904	1.114746215	1.106059675	0.983281308	0.99233395	0.767757944	0.897663083	0.919010725	1.167555204	0.925899174	1.091370174	0.978228053
Q80ZK0	1.091814994	0.99955672	1.023761614	0.982204355	0.892876974	1.035728673	0.983220791	1.01306354	1.011832339	1.00081687	1.013130871	0.975619736
Q9QWK4	1.186140215	1.581142756	1.359625344	0.839332583	0.842993286	0.742145939	0.878868257	0.953983261	1.075954381	0.765670139	0.849786426	0.852805451
Q9CR27	1.032358479	0.96959815	1.050553706	1.067881567	1.043578296	0.9364759	0.966263451	0.989326277	1.041425455	0.824859671	1.022931267	1.006027849
Q8K182	1.103017059	1.032630293	1.10561522	0.898598185	0.809816888	0.81831152	1.239585391	1.146767511	1.233001136	0.739218849	0.838447929	0.887393169
O35304	1.035301447	1.086541667	0.919596098	1.061684958	0.958635962	1.058886	0.953840225	0.976235195	0.975463447	1.004992047	0.974365189	1.055098925
Q9D7H3	0.973626635	0.941905682	1.002347994	1.05864936	1.062479166	1.032452149	0.979499582	0.993336497	0.981528572	1.003059858	0.971158469	1.042250473
Q9QUM7	0.95105338	0.923148008	1.058090025	1.000914976	0.971143909	1.041503289	1.04832428	0.912443183	1.052720477	1.171682757	0.980435641	0.966769604
Q9Z1M0	1.073116241	1.003195116	1.01586374	0.924692199	0.9444866	0.916019365	1.104208717	1.084664025	1.066172809	0.985872712	0.965926835	0.905703085
Q8CAE9	1.09893234	0.913445903	0.989600961	1.000816735	0.851345068	1.184372284	1.031796358	0.952873389	1.07522139	1.007861029	0.841991567	1.112056245

P51569	1.040729112	1.005905474	1.023034022	1.017998121	0.918295199	0.997880094	1.053428445	1.011451814	1.030594269	0.940537799	1.006539147	0.939079375
O35386	1.08088097	1.209552489	0.947595877	0.751883365	0.923358191	0.976749368	1.045689278	1.196919211	1.058211884	1.000245398	0.799031361	1.036665931
Q99JP6	1.021875814	1.103921303	0.908002372	0.99462142	1.036916451	1.05462509	1.011881982	1.041065896	0.910634018	1.068738564	1.012218921	0.906049567
O08992	0.986922424	0.879602206	0.973817979	1.004960026	1.077427626	1.079024349	1.051465831	1.023378055	1.034920778	0.947644967	0.959280571	0.983772384
Q5S006	0.980520433	1.028267133	0.966874691	0.987836639	0.939219963	0.960193268	0.981606717	1.061481625	1.027964	1.087403857	1.021391318	1.001334037
Q03249	1.005987319	0.992293051	0.984370233	0.949168459	0.861798443	0.848684085	1.180866312	1.099101681	1.079520324	0.95530209	0.95107498	1.056467158
Q8BUE4	1.066099049	1.012248536	0.953604113	0.969961499	0.958558812	1.023618701	0.988877326	0.970323211	0.985116204	1.073726841	1.016571169	1.048584172
Q9CWL8	1.182783685	0.959471679	1.089174179	0.917808244	1.055557827	1.06006511	0.992767932	0.95277817	0.943419735	0.976669067	0.997792958	0.909105704
Q8R516	0.962196131	0.935216036	0.930467211	0.969034043	0.810478821	0.964542479	1.068607403	1.010070227	1.19177835	0.973369135	0.990562849	1.164095942
Q9CYU6	0.870334483	1.059262591	0.883040296	0.946785377	1.055509214	0.889202413	1.053600792	0.889455541	0.879219017	1.232223946	1.23656159	1.093768463
Q80TT8	0.967367771	1.005251778	0.867760975	0.979864823	1.052203878	1.011674419	0.968094829	0.955584861	1.074335983	1.248769708	0.992535363	1.008025012
Q76M72	0.994345921	0.961688059	1.029322272	0.994801874	1.028337788	0.98884948	0.969844471	0.989039051	0.962274886	1.093585609	1.018979342	1.03580493
Q8K157	1.002551788	0.966248552	1.00864876	1.046755045	1.030111695	1.000623108	0.991728209	1.040150706	0.918220861	1.089415655	1.027930057	0.945816492
O89050	0.896326409	1.548628173	0.720921381	0.858526066	0.840354317	1.03805921	1.243025845	1.256857515	0.733306999	1.75837395	0.677798408	0.812586064
Q8BGV7	0.980561886	0.996988576	0.986751319	0.944953638	1.040811914	0.954783505	1.045535298	1.042302927	0.979153045	1.060132876	1.050014287	0.944923095
Q91ZV7	0.971666448	0.950202607	0.991987025	0.985797599	0.973951695	1.016100585	1.041838425	1.074690148	0.952407218	1.341675339	0.933531312	0.932004491
Q8BXV2	1.305652672	1.246728055	0.767378651	0.806013539	1.235232082	1.137153132	0.748491024	0.823580823	1.153644532	1.673676234	0.783803818	0.728145789
Q91VU7	0.887265145	1.124745309	0.984412824	0.895747925	0.990536775	0.909709231	0.981396392	0.974001016	1.004811645	1.128023161	1.136373524	1.014247792
Q3U0S6	1.034819799	1.034501927	1.010014432	0.942872582	0.94068301	0.965684126	1.10236631	1.028473074	0.960973149	1.072180195	0.976466725	0.976663664
Q9CQA1	1.304010208	1.366347972	1.159012212	0.700186619	0.687905289	0.686288455	1.269117797	1.215821974	1.239807651	0.739332096	0.745744294	0.740991259
Q9Z0S6	1.049682879	1.058444012	1.02973087	1.004922996	1.120587564	1.065081129	0.910295696	0.956782451	0.947707423	1.033605326	0.994209799	0.897663086
P19182	1.462582989	1.152712505	1.364083598	0.749808511	0.920620828	0.917928944	1.070156841	0.798764839	0.891697033	1.347866721	0.773401659	0.780950294

Q9WVD4	0.95412514	1.002524895	1.023500459	0.993027618	1.178969046	0.937228939	0.974452211	1.035560259	0.832420212	1.139660639	1.090772681	0.923434877
E9Q414	1.005529259	1.044497252	0.994593217	0.885843732	0.960012413	0.926508722	1.062207598	1.052494517	1.081971673	1.054470219	0.968686324	0.979554657
P06802	0.967779604	0.949182842	0.926042997	1.073055167	1.063416215	1.096914283	0.838486281	0.795047374	1.321505794	1.331312177	0.911456599	0.895455757
Q91YN0	0.997640028	0.996676676	1.008737294	1.022962788	0.995274049	1.010176508	0.969568226	0.961097504	1.04601593	0.982696669	1.008406699	1.01657748
Q9D7V9	1.085993342	1.069928267	1.10141974	1.003045371	0.983246653	0.96331779	0.946452397	1.015692657	1.093452646	0.941716199	0.925188119	0.871658786
Q8CEC5	0.951702749	1.010555867	1.033435889	0.93443233	1.042401303	0.896754106	1.01638332	1.052276862	0.948442816	1.003491801	1.16190194	0.927035718
Q3ZT31	0.971180901	1.011733666	0.996613221	0.994400939	0.923651298	0.967482116	1.01427965	1.041657679	0.902213552	1.030010972	1.139620618	1.019489631
O88630	1.352677643	1.052891 7	0.928444694	0.780415195	1.087271764	1.093435678	0.860328449	0.82865928	1.019391867	1.479156381	0.894927486	0.925941051
A3KGF9	1.117503472	0.859177728	0.911536514	0.841524661	1.098952099	0.925237421	1.025155674	0.858656295	1.085510101	1.290477125	1.077371924	1.044482583
Q8R332	1.018689251	1.01391107	0.992353693	1.002883579	1.047386308	1.105486924	0.913302208	0.950879883	0.955735499	1.079175411	0.981209742	1.028601002
Q5XG73	0.990214008	1.113884431	1.034060466	0.956110632	1.029798758	0.989214314	0.984792702	1.048359156	0.986455744	0.96606119	0.970846209	0.938238205
Q923M0	1.215671438	1.163707274	1.09558194	0.96685538	1.150404121	1.102241968	0.940718069	0.874142585	0.90017429	0.91772226	0.891341999	0.847414161
Q91YT2	1.080003246	0.987313642	1.040814013	1.025114768	0.955053876	0.967453799	1.011612532	1.095216728	1.051186243	0.921543072	0.884331241	0.982315968
P52795	1.090076726	1.021700591	1.070449823	1.079431515	1.132859018	0.990041073	0.924993886	0.980410926	0.998550219	0.841302297	0.945461082	0.919220832
Q8BQ48	1.05950316	1.036914858	0.990422463	0.981935125	0.903828298	0.956938111	1.043386868	1.023006031	1.003414081	1.008883204	0.99581342	1.019623197
Q9CVI2	1.197155832	1.15995733	0.847162195	0.910126314	0.955149655	1.309941416	0.841564062	0.927420979	0.950147338	1.495386704	0.798012016	0.940856147
Q5U464	0.970311938	1.019028117	0.988308109	1.069546761	0.99917966	1.082803571	0.970225175	0.934937985	0.938691491	1.011117108	0.982750713	1.091561751
Q8K135	0.953113243	0.95272535	0.987554065	0.998934417	0.971631819	0.996371782	1.030520671	0.998124771	1.004419419	1.032314859	1.037381904	1.058232856
Q920L1	1.262899909	1.083181748	0.945658129	0.937804684	1.005436803	1.068415093	0.961955134	0.95241676	1.097558334	0.971865018	0.870272654	0.904965648
Q91ZU1	0.947552177	1.05596508	1.0414909	0.980115557	0.962632263	0.940565027	1.042899599	0.985184951	1.012681845	0.948226038	1.033047184	1.027140129
C3VPR6	0.905255962	0.982701872	0.99493607	1.039306699	0.991382858	1.026371692	1.029989072	0.959713467	0.999669648	1.041877609	0.978590271	1.090568663
Q9D009	0.959724974	0.987273224	1.058620163	0.998474743	1.222140819	0.930342717	0.90868225	1.005924014	0.853019372	1.122855805	1.12289205	0.908093226

Q9CW07	0.854928437	0.961871398	0.944249124	1.006700415	1.002088274	0.961159274	1.047064678	0.993062847	1.033613091	1.137015319	1.037879027	1.071699134
Q91ZW2	1.008322411	0.93077306	0.991840471	0.991512762	0.970996844	1.040106806	0.966449639	0.960287808	1.004515705	0.976923331	1.091133335	1.072605882
Q8R1Q9	1.073154893	1.139001006	1.072956357	0.921329672	0.982897559	1.061575036	1.09091176	0.975215949	0.932609264	1.051949297	0.848686945	0.923233507
Q8K3I4	1.186734573	1.189274318	0.908466824	0.898069982	1.00415619	0.971685424	0.947044855	0.877808643	1.001437473	1.119213616	1.085885592	0.903205655
Q5GH67	0.823234838	0.91116335	0.928172296	0.847555524	0.888111612	0.897841155	1.004708248	1.00598652	1.063954357	1.316005689	1.205361844	1.179483958
Q6PAV2	0.975842119	1.014265197	0.971328676	1.065346072	1.113463846	0.994730557	0.867878499	0.969911627	1.026331199	0.905738767	1.050463483	1.044494476
Q8VDP6	0.923985094	0.954325191	0.983775941	1.033758809	0.939127281	0.968324798	1.018207341	1.023636524	1.071333855	0.87865779	1.042501064	1.107473282
Q6PE87	1.129788231	1.053061943	0.932595524	1.054795042	0.989614005	1.108983821	0.974407845	0.987880303	1.050281047	0.929777534	0.873361744	0.95904327
O35710	0.739494002	0.955854519	1.083663354	1.138035569	1.02655498	0.710919615	0.733603094	1.108685128	1.169293901	0.682817597	1.237755454	1.22700344
Q69ZT1	0.824583975	0.921185396	0.882626485	0.979162385	0.913389378	0.965962287	1.163785891	1.006071298	1.069305916	1.049265801	1.123077047	1.076239453
P06328	1.055229143	1.12743193	1.045992967	0.97675763	0.921009727	0.975131113	0.980551763	1.051943462	0.980985152	0.951470388	0.975777133	0.964235632
P59823	0.924344657	0.968913764	0.942941251	0.978990738	0.932579985	1.001196166	1.066006215	1.056235064	1.015852011	1.124634061	1.028517005	1.005539642
Q61324	0.974196371	0.930108684	1.03299115	0.970091844	1.043439305	0.989243382	1.023932959	1.051145717	0.955211648	1.128123342	0.984364965	0.988963264
Q99PW4	0.982635924	1.468030301	0.999108336	1.004969049	0.988849708	0.939241948	0.94173653	1.058865672	0.867280425	0.804800049	1.062481641	0.832044444
Q9JHZ2	1.118822736	1.117137441	1.064702431	0.998508044	1.111543508	0.953872604	0.960952561	1.039006284	0.961869241	0.927138196	0.934656991	0.834393476
Q8R480	0.899137467	1.024320444	0.980975196	1.000945753	1.018032111	1.018298074	0.943173645	0.896100621	0.857031001	1.057789595	1.129351084	1.233399955
Q8K2W3	0.969734719	1.015192756	1.055333329	0.956416736	1.043369007	0.935021742	0.936509047	1.162273109	0.967324989	0.990616768	0.994445566	0.981413093
E9Q1P8	0.867664951	1.280595678	0.991520152	1.039342403	0.963616815	0.925694464	0.962248465	1.021979833	0.933032769	0.977719008	1.037758014	1.006344333
Q9JJA9	1.042530102	0.990521667	0.971905152	1.029371517	0.953496655	1.024909785	1.004724097	0.982390573	1.006580898	0.986535584	0.974068372	1.067190491
Q9ERN0	0.986855104	0.92620208	0.975465179	1.069514213	1.002554254	1.081860939	1.038628293	0.967883322	0.976457108	0.964034823	0.986766514	1.046907695
Q60611	1.115282906	1.041643891	1.060491223	1.060429474	1.040739538	1.049235931	0.969232526	0.96695433	0.970651234	0.769886855	0.957548346	0.962974875
P61329	1.079528875	0.999406126	1.044752304	1.003261094	0.997530617	0.976756505	1.042268317	1.05209996	0.965349308	0.969040437	1.005468161	0.872637829

Q80V03	0.950617322	0.915842044	0.965590328	0.986176938	1.016233624	1.048570039	1.031566836	1.007434523	1.026759904	1.020610278	1.003287407	1.049290127
Q9DBL2	1.150430729	1.205528153	1.032805609	1.120650733	0.880506838	1.087817989	0.848308415	0.997651491	1.025092256	0.868084939	0.886301638	0.923382053
P47759	0.944810284	1.013486886	1.113891502	1.016531065	1.144756762	0.974482353	0.969734553	0.985594051	0.903447543	0.949915877	1.025822849	0.96526392
Q9D9H8	1.043461214	0.919192509	0.97565434	1.01485838	1.236439944	1.09634013	0.937575543	0.952493486	0.960591127	0.86475681	1.060893769	0.930349021
Q99J27	0.944946641	0.986547761	1.000452481	0.950203857	0.993450638	0.920633764	1.039271903	1.033418142	1.046440842	0.974091229	1.023699481	1.070340144
Q9DB98	0.845879093	0.891292015	0.876676987	1.010429518	0.850966791	0.996805613	1.101658163	0.928304212	1.058474362	1.032818486	1.181884901	1.20376587
O88904	1.005294941	0.928585446	0.916705792	1.008982116	0.986400971	1.043135387	0.956199618	0.959072273	1.00540353	1.162668061	1.051712126	1.074472268
Q9Z2V6	1.003324975	0.984054646	1.026653481	0.938603232	1.018506108	0.99213748	1.06939824	1.022276951	1.000566814	0.99289616	0.99534481	0.961079092
P56528	1.052153922	0.961770449	0.988187406	0.933825021	1.006471894	0.985249458	1.048162875	1.053892403	1.060788554	0.985914536	0.982502751	0.94321094
Q3UHF7	1.062306218	0.97793266	0.897243269	0.837439808	0.983816392	0.965293495	1.066022646	0.971331299	1.269422187	1.258968238	0.873548751	0.943596638
Q99KR3	1.012433397	1.012491079	0.998425733	0.996556491	1.036328758	0.967910265	1.042837528	0.986925726	0.965208573	0.951962284	1.050883986	0.97608298
Q61235	0.962684949	0.981486239	1.006433247	1.002311045	1.006433606	1.085222458	0.95496265	1.016105997	1.020282278	0.976776387	0.981328304	1.02231979
Q91YI1	1.018529905	1.096101319	1.067067805	1.083027919	1.083203203	0.953629133	0.866321703	1.009810972	0.948882398	0.782801535	1.006320113	1.048504483
Q61206	1.035118652	0.978564558	0.99589079	0.910456074	1.019259611	1.038566643	1.027557624	0.940684886	1.043869711	0.947139565	0.995252157	1.067332707
P70271	1.074080549	1.102233889	1.088181374	1.051057972	0.9278513	0.966160846	0.979146845	1.00620877	0.996477824	0.856814852	0.951169265	0.979678387
Q8VCE6	0.995977881	1.055604446	1.048873114	1.02523285	0.977344256	0.976046617	0.9124981	1.033133732	1.001603685	0.797727321	1.063365649	1.045751481
Q8CFI2	1.004716747	0.992577237	1.041391743	0.907488435	0.995077981	0.957961611	0.97825033	1.133804654	1.002482876	1.001225742	1.042361047	0.935761188
P52187	0.966046827	1.406246211	0.940735888	1.088786762	0.929909677	0.9907749	0.956034581	0.915970462	0.910690985	0.963497554	1.098333191	0.85272081
Q8C570	1.028450916	0.992928391	1.02547091	1.023844012	0.9354114	1.036893424	1.015555363	0.966290665	1.015514077	0.981357945	0.956142934	1.048399464
Q60722	1.006920052	1.032702669	1.03404992	0.964592202	1.006820151	0.952307308	0.999672619	1.054995356	0.944830416	1.038424334	1.018145896	0.98081053
Q811C2	1.590184375	1.224718482	0.71819037	0.688433114	1.414915022	1.332578563	0.723483802	0.716702966	1.185339874	1.472504932	0.681929555	0.644974347
Q9Z1R4	1.032261031	0.980923231	0.960608266	0.981845351	0.89573054	1.101135877	1.059289954	0.951354569	1.158080034	0.870209	0.902271692	1.074756078

Q9CRA8	0.992383911	1.072625297	1.031510689	1.01072922	1.073155612	0.918433964	0.928878069	0.930133851	0.883795	1.052868429	1.112736203	1.049334371
Q91XB0	1.079682795	1.064871363	0.974959118	0.922363932	0.988381703	0.921523252	1.083513425	1.031040149	1.043906615	1.066789263	0.937293154	0.932431282
Q9JHR9	0.973827011	0.868196377	0.966851434	1.024309436	1.031816849	1.097982203	0.978516002	1.015320566	1.021637704	0.982036305	1.02263208	1.031899477
Q8CGS6	0.968578529	0.962368189	0.941307568	0.938086309	0.972216676	0.979397175	1.054291068	1.035691557	0.973563379	1.134229465	1.085966925	1.006100577
B2RRE7	1.08686489	1.059174738	1.008427951	0.939287882	0.937095162	0.898973124	1.014206451	1.018080645	0.964575124	1.15136221	1.044399546	0.956355066
Q8R3H7	1.036079391	0.959661658	1.081813873	0.977955779	0.981839691	0.968251729	0.996141083	1.110730125	1.016669761	1.059175449	0.963207198	0.881881512
Q99LI7	0.883513599	0.905704385	1.050071832	0.94331471	1.047058116	1.026274756	0.938526984	1.007662992	0.991956134	1.08913763	1.084930852	1.06352723
Q8CHE4	0.948297159	0.978301938	0.931577144	0.996261429	1.16820753	0.988080644	0.99120496	0.976113853	0.943362904	1.026554914	1.040711743	1.058715114
Q8K0V4	1.080144071	1.073536755	0.948860665	0.982253957	0.893748182	0.938162981	1.07684712	1.011103754	0.988412454	1.030546899	0.99078008	1.024756445
Q91VM3	0.939803983	1.015794454	1.042150879	0.977924133	0.964243234	0.96430949	0.897487053	1.007635206	1.052856517	0.92599433	1.070363684	1.111997063
Q6PFE7	0.86793393	0.939777574	0.93702518	0.896329375	0.966020563	0.910318738	1.014409234	1.089679259	0.986710018	1.166459848	1.153670197	1.1069095
Q91ZI0	0.840452188	0.940350528	1.026021366	0.938852542	1.140999997	0.999518227	0.957808464	0.956144264	0.990281978	1.135734541	1.171571099	0.934632863
P97499	1.144034147	1.035334782	1.03360922	0.947895764	0.932854171	1.015330874	1.061605376	1.020447875	1.006450597	0.93051753	0.906547455	0.977441984
Q8JZN7	0.920931367	0.98162163	0.943131311	0.983662428	1.025678759	0.918748991	1.042316639	1.043410061	0.982778243	1.073892985	1.082112118	1.028311237
Q5BL07	1.014059894	1.070263791	1.084482062	0.949049793	0.989458909	0.97036561	1.030794188	1.002122048	1.046130362	0.960872523	0.968056693	0.904197168
Q3URU2	0.915393276	0.901943268	0.982804445	0.884018571	1.109380948	0.999302532	0.955292603	0.85074165	1.07386047	1.109674074	1.006334994	1.279476213
Q3TQB2	0.992013374	1.034575055	1.023851637	1.132633579	1.035255238	1.098855798	1.003416841	0.996824598	0.899465601	1.068713115	0.890928253	0.921968605
Q9CR25	0.991147946	0.891687885	1.002230986	0.948833822	1.039909185	1.082823378	1.006913171	1.015001192	1.004852092	1.026518453	1.022840231	0.991889658
Q9QXA5	1.010859399	0.981192819	1.071658661	1.041981811	1.044544447	0.979832339	0.926123231	0.984034014	0.990991026	0.870394004	1.054940931	1.014797559
P07361	1.1290298	1.506445552	1.137344715	0.70795082	0.717046383	0.912147134	1.305074401	1.221273432	1.192546389	0.713331661	0.673690018	0.630495415
A6PWD2	1.357066482	1.414084963	0.788655311	0.666816119	1.099146056	0.967708114	0.874560838	1.037661975	1.193879443	1.326752549	0.741991912	0.755307572
Q9Z0U0	1.006602463	0.966641996	1.03713742	0.981380631	1.040862657	0.953979589	1.008433477	1.03170141	0.995977713	0.957406357	1.023955513	0.991104769

Q9D8M3	1.122410115	1.121367762	0.951350146	0.986789436	0.925289288	1.020802048	1.056711082	0.946533183	1.005600389	1.290870048	0.881337163	0.866636746
P0C605	1.086491521	1.006509074	1.139619572	1.002310416	0.935386496	0.985330652	0.919680542	0.926184242	0.850106509	1.179938414	1.107730645	0.970301486
Q8VBW1	1.177739938	1.004116073	1.021146895	0.990295115	0.963944088	1.072382925	0.962836482	0.966782115	0.991234892	0.885415263	0.96433165	1.012096579
Q9D4V0	1.075154017	1.048184742	0.955789457	0.951055692	0.956292555	1.041016347	1.026675199	0.986307491	0.999888666	1.036586186	1.01701735	0.946936387
Q9CQ40	1.049445081	1.090914432	1.130225077	0.888513615	0.841177454	0.813590019	1.09252906	1.268187128	1.286339486	0.757380088	0.90813495	0.703224055
Q9CY45	0.986981926	0.980779906	0.982442714	0.97301225	0.978961065	1.074933579	0.999393643	1.002625172	0.944627208	1.042479057	1.023544547	1.055937611
P70694	1.027831781	1.072091168	1.005581106	0.996378077	0.997929572	0.945945767	1.000303648	1.029782847	1.019975708	0.955025341	1.006861672	0.940912328
Q64373	1.062969402	0.981134213	1.00364762	0.875880997	0.98958303	0.987069155	1.108043552	1.02577247	1.123599943	0.958863228	0.8975536	0.975241765
Q8BFR6	0.996616619	1.144928228	0.895139965	0.999086961	0.917716281	1.083024561	0.976851807	0.990848055	0.975668819	1.02369762	1.007750937	1.037730374
P63032	0.935287605	0.974796639	1.039879731	1.047380649	1.059390807	0.90401295	0.867105854	1.165890293	0.907856355	0.929826713	1.127064366	1.020423141
Q9ES34	0.980555329	0.947493534	0.949851022	0.983765519	1.057216435	1.033836817	0.970362239	0.919660752	1.008610952	1.055407678	1.049861629	1.095671265
Q91V64	0.935007568	0.987954814	1.043203557	1.031972182	0.918408029	0.974224696	0.996905076	0.994249546	0.970946429	1.037080177	1.079147532	1.047833698
Q8VBX0	0.933661154	0.960173464	0.976684948	0.987627868	1.012578871	1.016401824	1.035041933	1.011576298	0.96500743	1.060438189	0.978860965	1.112756922
Q9QZI9	1.045822474	0.976868663	1.07656911	1.019439535	1.096386347	1.009105697	1.045159866	0.946510449	0.97908917	1.065236069	0.919596072	0.890646197
Q62132	1.027195599	0.984316174	1.023169539	0.97260148	1.026129933	0.965711594	1.030831209	0.991017666	0.957015167	1.023429598	1.040755569	0.986597163
Q9EPQ2	1.119233979	0.952296844	0.921012704	0.976085916	1.137689108	0.993401784	0.900962865	0.99470601	0.905042146	1.132416652	1.129204617	0.940760161
Q9DCT5	1.001201533	1.032563581	0.998678383	0.974870868	0.921831656	0.945059263	0.966766174	0.957496562	1.039747777	1.172112877	1.114182836	0.940018305
Q52KG4	0.991416245	1.044623768	0.994750315	1.101467586	0.94353697	1.006536812	0.995412583	0.941670253	1.025042485	0.928178282	1.040799757	0.979477186
Q91ZR2	0.957818504	1.066928878	0.998214237	1.025188887	1.051481384	0.953560036	0.939871633	0.950953977	1.022526845	0.991739028	1.022826092	1.042375922
Q80XQ2	1.045020299	1.0285501	1.021527891	1.039427393	1.003970603	1.001514969	1.009548381	0.930669339	1.032679033	0.938603428	0.949287565	1.015420448
Q9WUD8	0.932386818	1.018343105	1.057928369	0.876534247	1.078562397	0.876359446	1.070186693	0.964031083	1.062484434	1.106979084	1.060563432	0.911367038
Q6TDU8	1.1585137	1.051562936	1.158406088	0.969846518	0.895829978	0.903123934	0.977901411	1.045988063	0.960138402	0.942381129	1.022798905	0.908044252

Q8CHK4	1.107969294	1.024399086	1.007329567	0.99215734	1.014383132	0.882184619	1.037039086	1.090800186	0.960730837	0.94673538	0.99464083	0.948077451
Q7TSH7	0.892445641	1.005007162	0.964917252	0.961519609	1.109915646	0.922278697	0.945567229	1.113942835	1.000522799	1.078456247	1.03558016	1.005367681
Q03391	1.218937078	1.044445656	1.137561256	0.993903585	0.989725717	1.054151762	0.922402869	1.062706397	0.954799043	1.033013592	0.836241317	0.842507866
O09106	0.965040701	1.015020242	1.003120577	0.975289613	0.978457886	0.983429474	1.026920802	0.965269271	0.987901753	1.090996864	1.021596098	1.039306798
P97364	0.847478713	0.897161565	0.955031356	1.059280916	1.013878653	1.050807807	0.982043564	1.089191571	1.021432038	0.984910046	1.048372438	1.041899418
Q9JI59	1.041749508	0.963599326	1.024535601	1.037856375	1.005403611	1.052779661	0.936005476	0.957584982	0.979436327	1.038884632	1.009509174	1.012053217
Q9R1S8	0.9950498	1.070672566	1.073218575	1.007297485	0.948848043	0.926342884	0.969633193	1.042755279	0.939995824	1.066037929	1.016020468	0.991080332
O70451	0.967622754	0.917184126	0.978192134	1.006523301	0.953344976	0.889663623	1.051957235	1.133771009	1.089680115	1.033747233	0.951290418	1.037320002
Q9CR89	1.086431451	1.021842276	1.030897341	1.0057223	0.912928544	1.033831136	1.065422476	1.03306579	1.012255611	1.006237885	0.889646497	0.941531424
Q3V1L4	0.931167617	0.941833582	1.005500897	1.040313705	0.982308531	1.048989042	0.981812081	0.983491132	1.020557915	0.979049462	1.029308976	1.061486774
Q9DA08	0.993493323	1.013233408	0.987507148	0.995949434	0.971886936	1.00004452	1.035861705	1.028376579	1.018433775	0.96255828	0.969578238	1.028288644
Q5XPI3	0.970016864	1.013095741	0.996093831	0.968431732	0.949811587	1.024529132	1.034015966	1.008724384	1.110721191	0.99282415	0.946445706	0.986808078
Q9D6G9	0.992587815	0.836444133	1.028603842	0.963364341	1.111571345	1.088480095	1.097379908	1.033448256	0.99922681	0.930898365	1.006744457	0.887865933
Q8K1C9	1.124591149	1.021289581	0.999985103	1.130801025	0.914606208	0.940106959	1.096905238	0.985190353	0.984260515	1.270975761	0.845614274	0.859877582
Q3TUF7	1.368420892	1.132715284	1.040583497	1.20377108	1.276270689	1.232582753	0.783223173	0.80350294	0.820887967	0.745547216	0.857604173	0.823343306
Q80Y19	0.906006864	1.03616218	0.919684353	0.994205152	0.923853434	0.977262845	1.072296062	1.098958345	0.986329424	1.211914563	0.975225328	0.990582265
Q8C0D4	0.833115407	0.970309179	0.969874061	0.97553982	0.916266144	1.025384302	0.939303329	0.93636898	1.044813713	1.064004998	1.110330046	1.232304028
O35671	1.020672249	1.12659571	1.070404543	1.008650662	1.053045294	1.034060925	0.909043431	0.949079729	0.901013063	0.961047352	1.045964363	0.949277954
P63147	1.074276973	1.093613736	0.953571813	0.900143703	1.057257136	1.132419184	0.949842062	0.944359006	1.070423672	1.189601639	0.867031005	0.902555982
Q8BYI6	1.205480602	1.144585731	0.907628483	0.892389172	1.182133882	1.104130948	0.928025831	0.851991369	0.968252689	1.34908235	0.907649396	0.799177378
Q8BS40	0.909260051	0.994914293	1.152502022	1.146553534	1.067389634	0.872721699	0.868209312	1.082108589	1.076091837	0.609721532	0.983462928	1.09016815
Q9D7E3	1.094291403	1.068295223	1.072626395	0.957364865	1.0227581	0.927906026	0.948686094	1.007890962	0.981615063	0.98992583	1.015267539	0.93792751

Q8JZS9	1.102383114	1.165061083	1.264025463	0.951757471	0.895963144	0.830740821	1.013679197	1.095989047	1.090951018	0.806323525	0.862422325	0.841450908
Q99LJ7	0.981665151	1.037143105	1.023246938	1.001024589	0.974568269	0.982177758	1.006599409	1.061685845	1.030818337	0.997275373	0.954956698	0.963036149
O08747	1.440088284	1.042949709	1.002554431	0.963022713	1.025668426	0.906384832	0.828720564	0.909982765	0.822075676	0.838514903	1.30211495	0.91964336
Q7TPQ3	1.00206393	0.977928788	0.936458996	0.892506413	1.083855113	1.023201447	0.951436995	0.83730803	0.977134847	1.373593946	1.073377657	1.059386697
Q9Z2B2	0.999656996	0.951995998	0.979971323	1.03964086	0.961410109	0.999548105	1.031962875	0.998788192	1.005890304	1.008472172	1.016018005	1.030334385
Q9Z1B5	0.968135659	1.008227638	1.037993508	0.93636277	1.038556551	0.984435308	0.975060721	1.036796445	0.932858563	1.130101925	1.055238505	0.960686325
Q8BJE2	0.961215672	0.992196586	0.949740482	1.004433615	0.779330762	1.078305515	1.111376663	1.038724592	1.125158788	0.885335216	0.947348682	1.073101775
O89023	1.141383365	1.087355684	1.035196667	1.041979398	1.110210093	1.131442864	0.885728019	0.914661568	0.880544323	0.91998209	0.963469072	0.950270756
E9Q7D5	0.892593201	1.808463267	0.865377307	1.131333634	0.841650122	0.892582563	0.934617474	0.929523028	1.053314358	0.606876244	1.015714033	0.896676822
Q6Y5D8	1.08411969	1.146505177	0.897249278	0.854798465	1.070429094	0.977130292	0.90656033	0.826176205	0.980069968	1.09988833	1.167476755	1.06376584
Q80U57	1.037707411	1.081318235	1.011600557	1.012578955	0.991646561	1.027247427	0.945491457	0.978670961	1.028681852	0.825661658	1.00356412	1.017372263
Q5FW53	1.179148735	1.203418837	1.113753898	0.976600477	0.783771496	0.846222997	1.141161874	1.061912591	1.092853435	0.929710946	0.767498934	0.904913039
P70303	1.17090549	1.020509995	1.09851253	0.898049308	1.175630331	1.111572073	1.022627859	0.919189392	0.818339583	1.006028149	1.005454756	0.821056291
Q9D023	1.168773077	1.336034365	1.207344523	0.88221282	0.811308059	0.949251261	1.104197984	1.151954591	1.009135284	0.660344431	0.843000533	0.744996619
Q8VDS8	0.924489456	0.921091891	0.967025348	1.002981139	1.024257641	1.05941415	1.016998563	1.017046107	0.973807131	1.066032595	1.060604096	1.001877896
Q8BWB6	0.846403604	0.902495172	0.97528689	0.891042618	1.393823515	1.047802569	1.04870162	1.125446742	1.023495855	0.926912953	0.984011197	0.79812606
P46412	1.140466243	1.380363761	1.006672523	0.818070775	0.870393789	0.878670065	1.15120418	1.021323066	1.1055066	1.009455835	0.862290241	0.764687932
P62046	0.932896705	1.054354128	0.944656501	1.018899427	1.074623874	0.964479941	0.937048907	0.942391328	1.003791094	1.094858618	1.097962081	0.989556024
P50608	1.615649611	0.94561222	0.951583135	0.782069164	0.989551953	1.314644145	0.86630438	1.033868369	0.8874075	1.023231049	0.958590699	0.764668185
P19785	1.065730014	1.03093573	1.007462433	1.078527823	1.016533383	1.014409213	0.995586149	0.952137358	1.007247425	0.862918898	0.95693519	1.006728136
O70361	1.071824991	0.994632878	1.032764287	0.951594713	0.969037336	0.986577236	1.011549869	1.007304211	1.003413492	1.024042764	0.98515753	0.99666152
Q80WP8	1.074746083	1.026887485	1.068017497	0.972900003	0.938420235	1.009749753	1.00418841	1.070302112	1.008691775	0.9572512	0.924724691	0.954915812

Q5U4C9	1.183849958	1.027577571	0.957565489	0.954931646	1.080158235	1.07215018	0.939998602	0.929120024	1.025907743	0.965879527	0.965122429	0.949904309
P61961	1.134151924	1.170113969	1.207888635	0.79448046	0.797269942	0.741068925	1.150648089	1.272995664	1.228828876	0.867163323	0.791138262	0.738414049
Q6DFW0	1.08100477	0.979764584	0.978695656	1.010966418	1.020010967	1.057798538	0.968197174	0.973761902	0.928267345	1.084030226	0.992756903	1.017075324
Q9Z0L0	0.995656011	1.225524812	0.864560736	0.992421748	1.206211608	1.035909845	0.897062296	0.963374319	1.000728371	1.092669058	0.949738733	0.881386999
Q3UM29	0.998675861	1.000184934	0.97993102	0.951010335	0.920710315	1.013725153	1.05656434	1.015546805	1.002647622	0.956687492	1.054556402	1.033042007
O08648	0.963308267	1.01022847	0.947600014	1.104048711	0.905044039	1.040164192	0.987793518	1.181514117	0.975885832	1.008746809	0.999953216	0.894936484
C0HK79	0.891896982	1.657009782	0.883789488	0.949309553	0.912941847	0.945031859	0.969339955	0.948829839	0.913401515	1.006146281	1.051472498	0.899412933
P70403	1.236937949	1.028708833	0.894955094	0.87625593	1.077868715	0.979223537	0.848316625	0.9013192	0.952916178	1.175645259	1.140027097	1.021412618
Q9QXA7	0.937655536	1.00663379	1.043427707	1.075330785	1.019402858	1.028407997	0.960370054	1.00063858	0.890577087	1.111636984	0.980074777	1.038403337
Q9JKQ4	1.029292309	0.965339875	0.965761762	1.004986744	1.056805865	1.002150041	1.006449681	0.951905801	1.027206068	1.180937854	0.954050407	0.969338391
Q9D552	0.902027466	0.891072745	0.984173481	0.963283566	1.070052297	1.032188254	1.02571373	0.971852665	1.009532516	1.092527025	1.039053584	1.061982348
Q8BFX3	0.976795646	1.044307774	1.06967601	0.999316935	0.966421701	0.829475934	0.887584954	1.074349194	1.067864524	0.800344188	1.129311473	1.054502434
Q9JMG1	1.020867403	0.985624356	0.973916807	0.956762627	0.998828811	1.041155911	1.066329796	0.999885075	1.089067144	0.888085118	0.952826092	0.99478806
Q9D9Z5	1.03251169	1.022903378	1.05499749	1.062865495	0.965335007	0.871973067	0.985646841	1.236907092	0.928430957	1.014763833	1.004277478	0.836098706
Q80XI6												
Q91WP0	1.235820753	1.175392798	1.030887258	0.77638093	0.801745425	0.778940569	1.20643909	1.16324655	1.221002346	0.937522912	0.829726283	0.79244963
Q61043	1.234023586	1.146898067	1.330911288	0.825933209	0.740715387	0.721016062	1.14660844	1.104955465	1.285102517	0.761177239	0.801793427	0.756455948
P97479	0.98689773	0.97043282	1.007239715	1.029293791	0.955077602	1.056632026	1.020294968	0.994802569	0.972086041	0.970520628	0.992382932	1.06159938
Q3V0M2	1.351137467	1.310249404	1.601135113	0.676179783	0.561545301	0.378756823	1.413740788	1.325500474	1.562617789	0.646918729	0.502049695	0.397787134
Q61084	1.165136398	1.101717566	1.026669447	0.832162132	1.215522387	1.131227901	0.838982894	0.905306284	0.9873589	1.136907206	0.886978376	0.917425371
Q8CIH5	1.061492865	1.058497917	1.140622905	0.961461006	1.012351412	0.890932636	0.995207106	1.094891192	0.976719035	0.942508468	0.959948437	0.897220584
Q61333	0.977264	0.988840094	0.982783253	1.066834324	0.989826524	0.978472045	1.001828573	1.139471689	1.018157722	0.987800424	0.983761876	0.89175508
Q9JJI6	0.9216	0.9294	0.9280	1.0790	1.0312	1.0715	0.9615	0.9947	1.0060	0.9318	1.1023	1.0286

	70508	08034	52897	557	86502	41649	46964	43694	7519	21156	20839	5607
Q8R5K4	1.0296	1.0274	0.9444	0.9739	0.9380	0.9650	1.0915	0.9886	1.0813	0.9718	0.9892	0.9945
	27625	76686	01977	32003	11601	18685	70521	2261	45787	39569	07589	16188
Q91VH6	1.0594	0.9608	1.0057	0.9779	0.9503	1.0423	1.0127	1.0808	1.0061	1.0089	0.9649	0.9570
	33875	00972	92632	59711	16191	98896	9686	5808	16448	32364	26223	71843
B1AZA5	0.8838	0.9868	0.9593	0.9663	0.9669	0.9807	1.0351	1.0032	0.9417	1.0261	1.0684	1.1987
	28275	93412	02647	45182	57149	77004	55679	2221	48525	1024	34082	71708
Q7TS63	0.9474	1.2520	1.0677	0.9261	0.9322	0.9018	0.9617	1.0923	1.0527	1.0060	0.9989	0.8491
	38682	06095	05982	09862	57769	07472	91545	62233	33387	29638	16997	37298
Q8VCM3	1.0336	1.0548	1.0493	0.9428	0.9789	1.0637	0.9780	0.9648	0.9302	1.0075	0.9984	1.0389
	80553	0381	06809	68793	25067	60428	00434	47217	82612	21916	91149	71025
Q91Z22	0.9117	1.1431	0.9317	0.9313	1.0443	0.8204	0.9035	1.1099	0.9655	1.0925	1.0795	1.1117
	00964	57086	124	2312	17754	82276	81304	16163	46977	66086	46819	77222
Q9QY01	1.0243	0.9136	1.1143	1.1247	0.9849	0.9170	1.0937	1.0655	0.9855	1.0291	0.8949	0.8904
	95733	62998	8901	62113	82916	09363	90592	9755	45017	98253	36542	90978
P49446	0.9810	1.1212	0.9678	0.9568	1.0371	0.9309	0.9654	0.9929	0.8808	1.1763	1.0626	1.0330
	26411	63608	87315	95061	97239	86153	41074	45251	99446	42966	2686	66542
Q9D8C2	1.0877	1.0977	1.0396	1.0551	1.0576	0.9740	0.9258	0.9519	0.9664	0.9006	0.8479	1.1452
	11352	04484	83257	90032	86902	11136	57987	9024	41347	6055	73418	92479
Q3TZA2	0.9524	1.0897	1.0159	1.0192	0.9917	0.8959	0.9812	1.0135	1.0418	0.7807	1.0725	1.0533
	89872	89006	15961	94755	84221	33721	11899	487	80087	33994	72213	95887
P70445	1.0297	1.1346	0.9886	0.8420	0.8873	0.8174	1.0309	0.9178	1.0725	1.1700	1.1127	1.0447
	47393	67019	16988	00699	87616	88228	1017	29757	78884	73252	3772	65838
P58059	0.9809	1.2553	0.9812	0.9881	1.0573	0.9151	0.9014	0.9369	0.9980	0.8969	1.0476	1.0322
	52121	31088	02999	71561	01384	52549	61993	97091	82192	77471	25165	25866
Q80YQ8	1.0064	0.9237	0.9586	1.0070	1.0156	0.9726	1.0446	1.0401	0.9826	1.0641	1.0009	1.0331
	42298	30923	98017	96131	68907	83484	32967	98789	94585	21514	33864	15868
Q9DBB8	0.9153	0.8789	0.9906	0.9933	1.0178	0.9836	1.0381	1.0054	0.9568	1.0801	1.0751	1.1011
	13266	39584	87431	01053	83589	53939	89213	85733	10766	61118	21551	89106
Q810C1	0.9933	0.9436	0.9315	0.9386	1.0755	1.0359	1.0204	0.9723	0.9781	0.9942	1.1010	1.0253
	61624	93034	68239	35989	76195	47262	8471	32379	31756	4934	90666	35678
Q8K2A1	0.9627	1.0018	0.9873	0.9659	0.9706	0.9905	1.0053	1.0001	0.9875	1.0333	1.0876	1.0218
	17522	00215	51624	76441	01849	06826	1219	31729	70174	93705	3854	68239
Q66T02	0.9386	0.9821	1.0174	1.0562	0.9765	1.0292	0.9879	1.0214	0.9996	0.9926	1.0181	0.9914
	57411	64712	5751	03697	71034	12691	50358	23005	25486	6757	5749	39191
Q80VM8	1.0395	1.0616	0.9587	0.8854	1.0295	0.9130	1.1442	1.0089	0.9093	1.0263	1.0668	0.9744
	11432	49837	67088	27646	05898	95818	64614	38945	11871	03516	90575	75641
Q9D486	0.9690	0.9672	0.9680	0.9909	1.0320	1.0978	0.9678	0.9971	1.0239	1.0074	0.9623	1.0537
	61505	93521	95232	36776	51233	5356	90647	42076	53717	03814	9517	36584
Q8K4K6	1.0275	0.9778	1.0262	1.0447	1.0463	0.9816	0.9620	1.0561	0.9936	0.9525	0.9818	0.9629
	21553	68931	78856	86355	41681	04357	38168	59686	92165	05931	00161	01049
Q9R1Z8	0.9883	1.0677	0.9724	1.0220	1.0989	1.0175	0.9712	0.9483	0.9232	0.9541	1.0616	0.9956
	12997	27479	02558	07954	82674	16321	40284	29013	72092	22227	12761	02211
Q99PP7	0.8906	0.9770	1.0245	1.1287	1.0852	0.9566	0.9109	1.0127	1.0576	0.7776	1.0252	1.0803

	33126	59054	67253	83777	07606	56124	36603	32354	62973	61911	64252	74073
Q8K2C7	0.9044	0.9058	0.9916	0.9200	1.1348	0.9142	0.9733	1.0226	0.9200	1.2397	1.1238	1.0481
	2679	33711	81863	27469	45546	62928	04981	49224	31876	79699	06901	67956
Q3TBW2	0.9968	1.0775	0.9838	1.0355	1.0144	1.0017	1.0121	0.9826	0.9984	1.0381	0.9559	0.9550
	99003	98217	68242	29491	53618	94519	62742	03699	01463	12495	80817	09144
Q810C0	0.8388	0.8478	0.9025	0.9883	1.0541	1.0042	0.9165	0.9083	1.0234	1.0885	1.2181	1.2360
	86933	4687	8916	79883	91601	55239	87761	3752	08621	3342	51532	52287
Q8BR90	1.2645	1.1356	1.1070	1.0095	1.0512	1.0148	0.9091	0.9344	0.9162	0.8591	0.9200	0.9140
	57121	78881	92826	57959	68963	40564	64429	06966	72436	32621	40591	75882
Q5DU56	1.0675	1.0003	1.0237	0.9724	1.0408	0.9932	0.9873	1.0212	1.0558	0.9763	0.9110	0.9775
	19437	6519	14097	00914	96557	86173	36121	24053	06274	61514	04736	64781
Q9JLV1	1.0422	1.0311	1.0270	1.0445	0.9475	1.0278	1.0032	1.0162	0.9903	0.9193	0.9798	0.9694
	06255	0475	17452	62656	47527	17213	42046	0206	86838	7366	76536	48794
Q04207	0.9861	0.9018	1.0387	1.0262	0.9569	0.9615	1.0949	1.2190	0.9750	1.0843	0.8689	0.9365
	17655	10336	9786	01818	64738	10434	58872	16364	81358	9877	84387	45916
O70422	1.5759	1.2299	0.6946	0.7862	1.1326	1.3227	0.7355	0.6206	1.2704	1.5081	0.7637	0.7432
	54967	67714	37553	68505	14634	04588	71609	3693	91172	4819	24725	93058
Q8K004	0.9434	0.9771	1.0333	1.0081	1.0067	1.0005	0.9567	0.9860	0.9925	1.0607	1.0469	1.0258
	09253	93654	81705	99021	32496	59123	39563	75839	46217	45898	39078	11998
A7XV04	0.9268	0.9312	0.9625	0.9771	1.0304	1.0364	1.0722	1.0168	0.9991	1.0489	1.0271	0.9943
	78861	57092	92944	24868	22922	7262	69092	46224	08688	38227	1907	25635
Q9JI39	0.9751	0.9181	0.9489	0.9803	1.0340	1.0769	1.0006	0.9594	1.0579	1.0937	1.0224	0.9859
	90463	04883	0998	99048	94648	26121	92236	61932	8888	67624	98346	21518
Q9ET54	1.0661	1.0344	1.0133	0.9230	0.9454	0.9768	1.0240	1.0328	0.8946	1.0941	1.0554	1.0007
	45684	81654	15698	68209	23064	64776	74224	91957	29728	16883	95513	52242
A2A6Q5	1.0461	1.0668	0.9570	0.9638	1.0295	1.0883	0.9622	0.9365	1.0266	1.1714	0.9569	0.9095
	88303	44664	15038	63928	9916	91403	77787	75039	9946	2454	1341	48443
Q8BIP0	0.9561	0.9597	1.0919	0.9429	1.1374	0.9004	0.9172	1.1016	0.8696	1.0137	1.1835	0.9233
	80391	32771	15202	99537	83155	13394	1504	34767	30563	11455	97139	40844
Q6ZQF0	0.9537	0.9518	0.9882	1.0194	1.0760	1.0830	0.9824	0.9418	1.0177	0.9831	0.9929	1.0349
	8575	76661	56801	36512	0837	843	55942	0928	04839	20604	23043	65614
Q9D9Q6	0.7895	1.0550	0.9264	1.0355	0.8027	0.9648	0.9937	1.2100	0.9908	1.0456	1.1343	1.0270
	91414	36976	67242	20658	13679	5109	54243	81926	33163	7693	16954	16831
Q9WU60	0.9798	1.0567	0.9794	0.9707	1.0509	0.9653	0.9627	1.0435	0.9620	1.1361	1.0228	0.9485
	13073	20211	76157	62707	09505	76347	93345	83065	49586	13143	22712	17516
P52430	1.1708	1.2221	1.1171	0.7849	0.6744	0.8141	1.3038	1.1611	1.3338	0.7953	0.6982	0.7929
	83119	69664	72747	41309	54035	61283	63368	98292	22035	57423	55822	12645
Q8BTI9	0.9417	0.8739	1.0370	1.0043	1.0033	0.9501	0.9678	1.1144	1.0782	1.1474	1.0100	0.9199
	56512	22433	38949	11769	62988	43473	27427	74401	88436	97576	59909	3359
Q9Z2R6	1.0662	1.1239	0.9319	0.9701	0.9816	1.0239	1.0641	1.0114	0.9400	1.1647	0.9177	0.9232
	22564	03095	91089	79299	48504	70733	37171	47408	55509	3458	38654	36404
Q8CIR4	1.5124	1.3120	0.8711	0.6706	0.6976	0.9032	1.7723	1.1000	0.7728	1.2018	0.7165	0.5972
	67867	356	09849	75465	1873	09119	06585	75945	27743	38032	23832	14106
Q8BGC9	0.9343	1.0189	1.0233	0.8888	1.0730	0.8930	1.0824	1.1131	1.0126	1.0582	0.9899	0.9205

	59063	08584	83986	04628	25038	61691	81117	51234	68515	32352	26866	18644
Q9JJ26	0.7665	1.1124	0.7981	1.2903	1.1619	0.7206	1.1696	1.2131	0.9504	1.2675	0.8838	0.8001
	77877	98379	39632	7353	44944	38527	04775	44435	87854	96263	62333	31412
P0C7Q1	0.8621	0.9297	0.9930	1.0377	1.0500	1.0069	0.8885	0.8921	1.0566	1.5890	1.0216	0.9308
	27807	45726	13474	09077	00537	89136	8412	18749	72498	51901	05496	47954
Q6PFX9	1.0606	1.0002	1.0683	0.8976	0.8944	0.8404	1.0843	1.0968	1.1293	0.9905	1.0013	0.8989
	48467	73151	31926	8669	91936	38053	5241	35021	72696	56444	75258	13606
Q62441	0.9900	0.9384	0.9932	0.9848	1.0127	0.9826	1.0324	0.9774	1.0191	0.9747	1.0328	1.0636
	81283	47104	2576	13337	58843	24431	82691	88483	34382	52243	98709	93705
Q8C2B3	0.9865	0.9816	0.9805	0.9986	1.0517	0.9922	1.0530	1.0035	0.9991	0.9524	0.9866	1.0175
	53206	5959	47504	35886	70174	81037	18361	37714	27637	44615	71297	41518
Q3UZZ6	1.1126	0.9457	0.9568	0.9539	1.0382	0.8089	0.9188	0.8373	1.0012	0.8414	1.3207	1.2002
	29515	44505	73122	79193	08285	56525	23798	98541	27394	82264	73394	09455
Q9D5Y1	0.9511	1.0588	0.9073	0.9892	0.9357	0.9945	1.0727	0.8675	1.0200	1.2179	1.0577	1.0284
	03266	16963	98063	98076	59604	41113	43516	51295	89006	32377	39826	72126
Q14AT5	1.1393	1.0101	1.0213	0.9786	0.9309	1.0180	1.0369	1.0002	0.9836	0.9828	0.9360	1.0008
	65139	5123	01763	99735	72961	34883	69695	95956	48697	74849	06029	72438
Q91YK0	0.9696	0.9428	0.9669	0.9411	0.9454	0.9972	1.0368	1.0757	1.0930	1.0288	0.9769	1.0328
	41564	64723	13317	51392	1418	05443	81947	71667	36773	93809	75511	06372
Q99LB2	0.9801	0.9454	0.9948	1.0310	0.9815	0.9849	1.0172	0.9496	0.9616	1.0401	1.0952	1.0485
	31292	38483	82935	52314	63131	34821	7418	8057	64672	48454	40946	75869
Q8K327	0.9152	1.2533	0.9300	0.8870	0.8719	1.2558	1.1727	1.0896	0.6822	1.1863	0.9148	0.9701
	14586	96876	84116	79826	37245	16561	86259	83562	27035	42097	53012	05853
Q9DB00	0.9943	0.9572	0.9370	0.9879	1.0262	1.0157	1.0481	0.9947	0.9781	1.1082	1.0086	1.0142
	33313	82465	44978	03169	57065	06472	71793	07741	47212	88937	939	31233
Q03146	1.2618	1.1834	0.8076	0.7814	1.1531	1.2700	0.8417	0.7963	1.2263	1.5320	0.7424	0.7314
	57832	05292	89232	10933	6742	91268	39361	95767	72275	7801	76046	60966
P51954	0.9185	0.9176	0.9904	1.0208	0.9857	1.0494	1.0173	0.9548	1.0402	0.9433	1.0449	1.0996
	52115	02419	1473	33452	58012	41372	92805	16173	51113	24743	22689	63227
O54943	1.1194	1.0618	1.0976	1.0898	1.1132	0.9769	1.0162	0.9896	0.8774	0.9412	0.8897	0.8815
	24821	39698	17647	34992	96805	30783	50733	44095	13465	91569	81178	13907
Q99KL7	0.9478	0.9738	1.0709	1.0762	0.9996	0.9844	1.0232	1.0736	0.9721	0.9672	0.9873	0.9223
	92373	42147	25214	14836	48911	08083	73165	65343	39619	32407	45439	48864
Q9CPX7	1.0154	0.9927	0.9398	1.0821	0.8755	1.1567	0.9873	0.9174	0.9553	0.8997	0.9714	1.2295
	71816	93612	98301	53316	49798	33302	44265	42154	14369	64027	60219	77721
Q05860	1.0900	0.9815	1.0576	1.0171	0.9718	0.9512	1.0113	0.9939	1.0164	0.9393	0.9878	0.9841
	06835	60067	07984	97208	15223	88043	138	79331	08893	44003	50264	69761
Q9CWV1	1.0153	1.1351	0.9985	1.1684	1.0406	1.0573	0.8842	0.9783	0.8636	0.8673	1.0058	1.0102
	57975	58357	12942	41902	14693	86813	9479	30073	74945	17393	56579	76742
Q9CQ82	0.9900	0.9682	0.9845	0.9227	1.1221	1.0788	0.9847	0.9503	1.0015	1.1399	1.0400	0.8932
	7616	26421	07096	76998	19527	09006	34853	44756	85535	77957	30499	56873
P08121	1.0939	0.9283	1.0341	1.2394	0.9715	1.0381	0.9680	0.8725	0.9228	1.0363	0.9879	1.0010
	07248	83219	28892	02708	81887	49823	62837	1255	17116	54715	85998	63201
O08665	1.2133	1.1241	1.1048	0.8745	0.8935	0.8922	1.1465	1.0516	1.0953	0.8035	0.8539	0.8800

	20866	42169	49785	39011	10977	88681	09113	23906	78016	01669	9588	81108
Q8R5L3	0.9195	1.0257	1.0675	1.0910	0.9856	0.8406	0.9750	1.0777	1.0655	0.9611	1.0059	0.9629
	88326	22981	28421	10357	24817	79762	0922	9558	67984	14202	6796	91893
O35855	0.9996	0.9636	0.9619	0.9196	0.9449	0.9764	1.0510	1.0802	0.9991	1.0395	1.0542	1.0211
	52615	30726	86386	53823	94464	92516	11326	01572	15933	70769	27743	09905
Q9WUU9	0.9932	0.9447	1.1768	1.0001	1.1083	0.9257	0.9622	0.9361	1.0012	0.9661	1.0347	0.9474
	96581	12428	32925	33214	26594	97676	7481	01397	10062	71099	07705	54574
Q6PDM1	1.6024	1.3137	1.1409	1.0282	1.0069	1.1426	0.8629	0.7916	0.8759	0.8014	0.7708	0.7656
	10366	80776	2229	59601	03444	85908	25378	50528	215	38909	2067	04627
Q91VX2	0.8873	0.9430	0.9781	1.0442	0.9015	1.0486	1.0666	0.9267	0.9661	1.1244	1.0157	1.1664
	80136	34613	31181	77295	20793	92921	38586	39103	87857	29534	04301	41998
A8E0Y8	1.0040	0.9665	1.0112	0.9929	0.9466	0.9435	1.0870	1.0411	1.0518	0.9616	0.9849	0.9931
	38084	26824	28	35422	55322	62095	75601	54491	2414	15188	63186	03542
Q8BLY3	1.0412	0.9852	1.0567	0.9775	0.9254	0.8849	1.0174	1.1099	1.0958	0.9445	0.9789	0.9515
	33192	08725	30416	76956	84979	34307	1279	73311	44075	47933	49427	9904
Q9CXB8	1.2021	1.0737	0.8930	0.9664	1.1727	0.9962	0.9592	0.9319	0.9625	1.3078	0.8784	0.8816
	85058	18817	55428	18505	36109	67171	76561	6261	95634	56321	20577	04521
Q5SS80	0.9769	0.9719	0.9482	0.9711	0.9738	0.9390	0.9624	1.0059	1.0547	1.0003	1.0727	1.1281
	03487	52023	71148	44839	69128	66297	2319	69652	17953	6656	5122	57941
Q8CG64	2.4783	1.9578	0.4090	0.4308	1.4819	1.3946	0.3695	0.3288	1.3398	2.1742	0.2714	0.2874
	67352	17557	65271	76157	38666	03568	85446	61661	40226	94384	24011	42827
Q5D525	1.2077	1.0193	0.9836	0.9141	0.9588	1.0146	0.9709	0.9905	1.1197	1.2087	0.8568	0.8922
	84885	69601	86438	90498	93592	13608	07862	39063	12278	84893	32154	66955
Q99MY0	1.0843	0.9926	0.9631	1.0157	1.0708	1.0721	0.9888	0.9395	1.0204	0.9600	0.9803	0.9444
	0287	30079	29037	05765	32733	58964	39214	5886	94306	45048	59756	69669
Q9D2Y4	0.9749	1.0344	0.9995	1.0161	1.0983	0.9913	0.9733	1.0309	0.8862	1.0888	1.0340	0.9449
	45429	2367	40711	65894	63952	38211	27876	33021	68051	31679	60096	12779
Q60665	0.8138	0.7561	0.9962	1.0579	0.9288	1.0176	0.9918	1.0359	1.1376	1.1108	1.0566	1.1107
	84233	25678	57749	43208	91682	90968	52938	43249	2721	69316	17705	71799
Q9D5H4	1.0436	0.9698	0.9837	0.9858	0.9815	0.9593	0.9974	1.0282	1.0617	0.9745	1.0041	1.0149
	66021	59175	94646	23366	9341	03017	2455	02442	0584	1603	67209	31662
E9PVX6	0.9748	0.9624	1.0318	1.0314	0.9534	1.0434	0.9886	1.0170	0.9858	1.2642	0.9033	0.9911
	73903	63949	60859	04381	20341	7281	15162	06157	07193	6729	75385	57503
Q8R4X1	1.0699	0.9426	0.9107	0.9778	0.9914	1.0043	1.0111	1.0082	1.0070	1.1002	0.9713	1.0894
	08027	96018	18159	39777	7581	2336	39837	74125	42425	07111	9283	62661
Q9WUS4	0.9082	1.0030	1.0184	1.1320	1.1826	1.1437	0.9527	1.0602	0.9356	0.7570	0.9819	0.8674
	98565	66616	28159	47939	49068	85805	82702	92192	97376	69264	97316	1041
Q8CGC6	1.0038	1.0203	0.9975	1.1197	0.9622	1.0687	0.9472	0.9709	1.0349	0.8392	0.9573	1.0592
	37596	0131	58981	93298	66706	80086	79476	37699	67009	62309	49342	96731
P21183	0.9660	0.9768	1.0165	0.9859	0.9243	0.9334	1.0144	1.2126	1.0506	0.9235	1.0259	0.9148
	86138	43009	78489	3587	64157	52862	74831	32776	15391	42171	54417	99092
E9PVD1	0.9548	0.9400	0.9543	1.0121	1.1395	0.9988	1.0614	1.0221	1.0207	1.0261	0.9555	0.9489
	4203	67085	96058	51565	39087	33297	96405	97263	92639	02734	57729	65428
Q9CVW4	0.9546	1.0033	0.9605	0.9381	1.0211	0.9966	1.0566	1.0280	0.9718	1.2181	0.9877	0.9672

	03034	21081	76332	35857	63123	89771	42155	71414	29806	20451	94471	70131
Q8BXX9	1.0668	1.1479	1.0361	1.1265	1.0158	1.1113	0.8838	0.9511	0.9500	0.8461	0.9306	0.9512
	31891	3364	4184	2417	57069	39633	54425	38309	74972	66606	7375	72706
Q920F6	1.7330	1.5302	1.3207	0.8895	1.1274	1.1270	0.7975	0.8337	0.8122	0.6464	0.6601	0.5875
	59781	55587	09198	9599	60424	94284	7535	54743	19456	46991	54862	73419
A2AJX4	0.9300	0.9554	0.9949	1.0183	1.0469	1.0313	1.0067	0.9652	0.9794	1.0573	1.0123	1.0495
	10106	40508	70553	5035	12489	12415	76806	50834	90184	04317	52955	88828
Q9Z2R9	0.8847	0.9280	0.9630	0.8739	0.8681	0.8742	1.1796	1.0812	1.1677	1.0051	1.0279	1.0857
	23333	14488	23623	76977	13673	67723	46985	16612	13363	93488	7557	36218
Q3UEI1	0.9813	0.9346	1.0270	0.9373	1.0597	1.0111	1.0439	0.9950	0.9379	1.1706	1.0098	0.9806
	34513	66986	41746	63019	84285	65765	07645	16095	04636	97653	23898	25106
Q80XE1	1.0491	1.0551	1.0909	1.0203	0.8678	1.0247	0.9812	1.0142	0.9438	0.9944	0.9945	0.9904
	91032	83706	97402	34786	24785	35815	39842	39465	4509	29618	4615	76716
O70370	0.9801	0.9926	0.9962	0.9806	1.1058	0.9741	1.0164	0.9311	0.9306	1.0067	1.0285	1.0965
	95794	34664	41395	72696	15643	73238	76179	54026	53269	86701	75516	40032
Q9D4H9	0.9114	0.9784	1.0092	1.0174	0.9830	0.8876	1.0507	1.0919	1.0153	1.0886	0.9982	0.9985
	48435	34455	74704	0237	91561	86797	46112	74796	07355	42608	40372	39026
Q8R1B0	0.9278	1.1538	0.9477	0.8645	0.9133	0.8875	1.0528	0.9453	1.0064	1.1649	1.1855	0.9821
	16977	10439	62831	74495	00763	33664	15902	51624	23462	23471	43408	96281
O70340	1.0601	1.0646	0.9817	0.9576	1.0114	1.0189	0.9990	1.0471	1.0366	1.0227	0.9401	0.8989
	35157	43274	0928	88514	20184	78217	47525	30244	52793	3378	69162	35881
P49891	1.0232	1.0052	1.1126	0.9718	0.9868	0.9797	1.0180	1.0619	0.9396	1.0314	0.9660	0.9367
	62217	05624	15174	27792	50221	22127	90718	90502	09085	65499	9036	39519
P43883	0.8787	0.9636	0.9601	1.0634	0.9936	1.0231	0.9785	1.0050	0.9166	1.0790	1.0756	1.1162
	05367	26478	7763	56489	60609	71543	45657	95545	35445	13066	93333	55636
Q8K0D2	0.9940	1.0003	1.0171	1.0136	1.0319	1.0290	0.9948	0.9821	0.9749	0.9402	1.0175	1.0075
	03033	41119	50417	08301	99418	42081	79966	30572	66967	82918	41703	0471
Q9D0I6	1.0999	1.0875	1.1139	0.8937	0.9459	0.9123	1.0602	1.0114	1.1267	0.9428	0.8862	0.9008
	15289	96151	70449	43535	86071	74818	36016	64269	51753	14075	65418	157
Q8K007	0.7495	0.9353	0.9433	1.0146	0.9498	1.0993	1.0324	0.9723	1.0333	0.9997	1.0912	1.1581
	85285	00762	74056	49758	0921	23301	56791	65716	2173	82539	11458	78664
Q64104	1.0148	0.9367	0.9903	0.9767	1.0251	0.9702	0.9782	1.0054	0.9942	1.0556	1.0905	0.9941
	69713	14751	02615	04036	83566	8779	6283	29107	60215	57818	24343	54537
Q3U962	1.2575	1.0966	1.0698	1.0114	1.0559	1.0202	0.9266	0.9427	0.9598	0.9417	0.9195	0.8589
	46334	30967	1157	38571	2178	13737	67149	86267	64354	09151	2409	46237
P81117	0.9282	1.0157	1.0328	0.9079	0.9124	0.8898	1.1260	1.1351	1.1199	0.8665	1.0126	0.9479
	91437	14334	74938	6108	96963	75624	40448	8516	32618	82224	05763	57837
Q8BL06	1.0719	1.0695	1.1007	1.0486	0.9716	1.0123	0.9669	1.0037	0.9780	0.9029	0.9400	0.9377
	34147	07058	72366	29186	64599	29222	50206	35159	57415	31612	54436	70623
Q7TSH4	0.9918	1.0060	1.0895	1.0832	1.0634	0.8978	0.9631	1.0856	0.9353	0.9454	1.0278	0.9092
	51459	64041	27963	14264	26617	12016	20688	7869	67332	54604	95573	26312
Q9D3A8	0.9509	1.0264	0.9018	0.9447	1.0115	0.9282	0.9963	0.9776	1.1165	1.1837	1.0225	1.0155
	43204	20132	04285	61982	33806	96402	71847	52799	07457	74642	85137	032
O54879	0.9565	0.9970	1.0082	1.0252	0.9390	0.9711	0.9698	1.0649	1.0184	0.9962	1.0516	1.0011

	97414	29315	35446	08558	4001	78389	73853	86767	61214	26581	99519	71052
Q9DAW6	0.8699	0.9902	1.0001	0.9942	1.0124	1.0168	1.0062	0.9894	1.0135	0.9650	1.0905	1.0264
	54641	01855	50693	77966	78365	60851	12072	3628	73219	36227	98315	11568
Q8BGX0	1.0410	1.0193	0.9470	1.0380	0.8699	0.9240	0.9487	1.0088	1.0077	0.8983	1.0926	1.1836
	37453	28388	67731	39909	48253	95958	6713	02228	61984	307	15089	22412
Q9R269	0.9998	0.8684	0.9378	0.9331	1.0103	1.0950	1.0508	0.9463	1.0916	1.1934	0.9095	1.0725
	78947	62126	84258	29975	31624	27042	12333	65752	2201	82635	16707	40972
Q8CIM1	1.2544	1.2346	1.2771	0.7942	0.7416	0.7387	1.2236	1.2021	1.2436	0.7993	0.7029	0.6713
	66801	34057	40344	40425	04868	21964	75883	17469	46435	047	48088	48493
O54931	1.0749	1.1971	1.0160	0.9673	0.9301	0.9227	0.9918	1.0227	1.0141	0.9560	0.9688	0.9473
	43498	16002	09294	26895	08386	68326	47099	95507	15487	55889	53412	7749
Q6PB90	1.0294	0.9750	1.0645	0.9695	0.9906	0.9679	1.0276	1.0055	1.0951	0.9774	0.9442	0.9520
	64719	61482	4953	52402	26161	94532	79594	89245	77915	56649	8091	43309
P11416	0.9700	0.9474	0.9644	0.9937	0.9797	1.0359	1.0194	0.9594	1.0185	1.0296	1.0237	1.0886
	6534	71916	34635	96159	72143	69845	55411	62415	87339	89899	81566	18053
Q99NG0	1.0263	1.0359	1.0680	1.0454	1.0878	1.0513	1.0869	0.9999	0.9997	0.9474	0.8424	0.8332
	07192	38169	0261	50198	79995	94501	30129	27536	48699	15866	969	63077
P15307	1.0027	0.9453	1.0103	1.0502	0.9984	1.0246	1.0639	1.0116	0.9612	0.9392	1.0091	0.9815
	54199	10146	1489	84074	42299	27338	10085	61628	15797	81209	31828	38139
Q8R2M2	0.9962	0.9594	1.0063	1.0053	1.1162	1.0272	0.9907	0.9007	1.0244	1.0036	0.9802	1.0298
	92298	72955	02768	94181	20698	13913	66346	46527	77478	81687	91984	49227
Q76HP3	0.9530	1.0335	1.0151	0.9453	1.1377	0.8875	1.0342	1.0065	0.9480	1.2728	1.0395	0.8454
	81075	74704	20316	20666	09792	04017	00504	64171	38644	74545	96231	14365
Q3UV71	1.1719	1.2481	1.2540	0.8249	0.7285	0.7271	1.1818	1.1531	1.2234	0.7916	0.8127	0.7497
	06211	50845	53565	56544	43499	03317	09705	59456	54174	07132	33607	79693
Q8BZH1		1.4066	0.8100	1.5113	0.9414	0.6173	1.2803	1.5127	0.8917	1.0257	0.7234	1.2456
		72308	17214	047	2646	13438	66462	69891	6113	43802	82873	81705
O88428	0.8592	1.0125	0.9403	0.9310	1.1679	1.0538	0.9830	1.0515	0.9961	0.8999	1.0566	1.0093
	77318	81678	00516	70358	9308	80288	7828	10117	96169	5542	94009	65863
Q64676	1.0323	0.9308	0.9364	0.9895	0.9331	1.0341	1.0155	0.9671	1.0862	0.9941	0.9995	1.0989
	30559	81587	58174	92661	54346	93558	31966	21774	19972	85109	65677	87465
Q4QRL3	0.9564	0.9505	0.8833	0.9595	1.1916	1.1661	0.9431	0.9131	0.9337	1.2096	1.0283	1.0064
	73017	12801	29837	43715	89254	59139	99857	32471	73174	15113	8931	04503
O08850	0.9643	0.9530	0.9449	1.0186	1.1588	1.1267	1.0029	0.9870	0.9535	1.1204	0.9482	0.9218
	34984	08253	33077	4287	36566	73096	76665	00044	5174	19089	60977	69302
Q4VK74	1.0294	1.0174	1.0295	1.0484	1.0783	0.9908	0.9973	0.9751	1.0029	0.9101	0.9727	0.9488
	15363	93608	78263	11334	05159	11578	44963	72949	09496	32179	69059	89587
Q3UPF5	1.1016	0.9873	1.1912	0.9859	1.2287	1.0894	0.9255	0.8956	0.9242	0.7880	0.9418	0.9222
	56116	72281	80042	25312	67161	04781	97191	58827	2299	65451	60985	41685
Q80VW7	0.9835	0.9259	0.9722	1.0174	1.0416	0.9791	0.9749	1.0477	0.9535	1.0231	1.0895	1.0161
	74392	84222	44635	16664	35088	28099	60894	30655	37518	86696	97028	58942

Supplementary Table 3 | The full protein names of DEPs in all heatmaps.

Gene Name	Protein Name
Rad21	Double-strand-break repair protein rad21 homolog
Gypa	Glycophorin-A
ApoM	Apolipoprotein M
Alb	Serum albumin
Lrrc36	Leucine-rich repeat-containing protein 36
Serpina1e	Alpha-1-antitrypsin 1-5
S100a9	Protein S100-A9
Pzp	Pregnancy zone protein
Vtn	Vitronectin
Pnpla6	Neuropathy target esterase
Apoa1	Apolipoprotein A-I
Mug1	Murinoglobulin-1
S100a8	Protein S100-A8
Serpina1b	Alpha-1-antitrypsin 1-2
Hp	Haptoglobin
Ngp	Neutrophilic granule protein
Fgb	Fibrinogen beta chain
Tf	Serotransferrin
Kng1	Kininogen-1
Ces1c	Carboxylesterase 1C
Snrpg	Small nuclear ribonucleoprotein G
Afm	Afamin
Serpina3k	Serine protease inhibitor A3K
Apoa2	Apolipoprotein A-II
Orm2	Alpha-1-acid glycoprotein 2
Serpina1a	Alpha-1-antitrypsin 1-1
Serping1	Plasma protease C1 inhibitor
Gc	Vitamin D-binding protein
Klkb1	Plasma kallikrein
C3	Complement C3
Itih1	Inter-alpha-trypsin inhibitor heavy chain H1
Serpina3n	Serine protease inhibitor A3N
Mrpl3	39S ribosomal protein L3, mitochondrial
Hpx	Hemopexin
Lrrc45	Leucine-rich repeat-containing protein 45
C8b	Complement component C8 beta chain
Trappc5	Trafficking protein particle complex subunit 5
Pon1	Serum paraoxonase/arylesterase 1
Apoa4	Apolipoprotein A-IV
Ltf	Lactotransferrin
Apoh	Beta-2-glycoprotein 1

Ttr	Transthyretin
Ahsg	Alpha-2-HS-glycoprotein
Camp	Cathelicidin antimicrobial peptide
Serpinc1	Antithrombin-III
Cfh	Complement factor H
Igkc	Immunoglobulin kappa constant
C4b	Complement C4-B
Ufm1	Ubiquitin-fold modifier 1
Tmtc1	Protein O-mannosyl-transferase TMTC1
Plg	Plasminogen
C5	Complement C5
Itih4	Inter alpha-trypsin inhibitor, heavy chain 4
Tbr1	T-box brain protein 1
Cfi	Complement factor I
Mbl2	Mannose-binding protein C
Erlin1	Erlin-1
Ak2	Adenylate kinase 2, mitochondrial
C9	Complement component C9
Ica	Inhibitor of carbonic anhydrase
Qsox1	Sulfhydryl oxidase 1
Mybbp1a	Myb-binding protein 1A
F2	Prothrombin
Blvrb	Flavin reductase (NADPH)
Sdc4	Syndecan-4
Fmr1	Synaptic functional regulator FMR1
Rps13	40S ribosomal protein S13
Pmm1	Phosphomannomutase 1
Psmb2	Proteasome subunit beta type-2
Ovgp1	Oviduct-specific glycoprotein
Edil3	EGF-like repeat and discoidin I-like domain-containing protein 3
Gpld1	Phosphatidylinositol-glycan-specific phospholipase D
Epb41	Protein 4.1
Shpk	Sedoheptulokinase
Plcb1	1-phosphatidylinositol 4,5-bisphosphate phosphodiesterase beta-1
Ank3	Ankyrin-3
Phactr1	Phosphatase and actin regulator 1
Clu	Clusterin
Gk	Glycerol kinase
Rpl7a	60S ribosomal protein L7a
Fkbp8	Peptidyl-prolyl cis-trans isomerase FKBP8
Ndufb11	NADH dehydrogenase [ubiquinone] 1 beta subcomplex subunit 11, mitochondrial
Iqsec3	IQ motif and SEC7 domain-containing protein 3

Ig alpha chain C region	Ig alpha chain C region
Rida	2-iminobutanoate/2-iminopropanoate deaminase
Rbm14	RNA-binding protein 14
Srrm2	Serine/arginine repetitive matrix protein 2
Chp1	Calcineurin B homologous protein 1
Exoc3	Exocyst complex component 3
Rpl21	60S ribosomal protein L21
Stim1	Stromal interaction molecule 1
Crip2	Cysteine-rich protein 2
Sbds	Ribosome maturation protein SBDS
Gatm	Glycine amidinotransferase, mitochondrial
Bzw2	Basic leucine zipper and W2 domain-containing protein 2
Supt16h	FACT complex subunit SPT16
Ddx39b	Spliceosome RNA helicase Ddx39b
Maea	E3 ubiquitin-protein transferase MAEA
Tsta3	GDP-L-fucose synthase
Grm7	Metabotropic glutamate receptor 7
Reps1	RalBP1-associated Eps domain-containing protein 1
Upp1	Uridine phosphorylase 1
Ankrd13d	Ankyrin repeat domain-containing protein 13D
Scg3	Secretogranin-3
Phf6	PHD finger protein 6
Stat3	Signal transducer and activator of transcription 3
Foxj1	Forkhead box protein J1
Wipi2	WD repeat domain phosphoinositide-interacting protein 2
Cldn11	Claudin-11
Htra1	Serine protease HTRA1
Hapln2	Hyaluronan and proteoglycan link protein 2
Plp1	Myelin proteolipid protein
Mog	Myelin-oligodendrocyte glycoprotein
Fth1	Ferritin heavy chain
Yeats2	YEATS domain-containing protein 2
Abcb8	Mitochondrial potassium channel ATP-binding subunit
Ccdc8	Coiled-coil domain-containing protein 8 homolog
Nmral1	NmrA-like family domain-containing protein 1
C1qtnf5	Complement C1q tumor necrosis factor-related protein 5
Vcan	Versican core protein
Serinc5	Serine incorporator 5
Ass1	Argininosuccinate synthase
Folh1	Glutamate carboxypeptidase 2
Nefh	Neurofilament heavy polypeptide
Cd81	CD81 antigen
Gja1	Gap junction alpha-1 protein

Ndufb4	NADH dehydrogenase [ubiquinone] 1 beta subcomplex subunit 4
Agps	Alkyldihydroxyacetonephosphate synthase, peroxisomal
Fam92b	Protein FAM92B
Dpysl3	Dihydropyrimidinase-related protein 3
Rras2	Ras-related protein R-Ras2
Srcin1	SRC kinase signaling inhibitor 1
Shank2	SH3 and multiple ankyrin repeat domains protein 2
Srgap3	SLIT-ROBO Rho GTPase-activating protein 3
Slc12a2	Solute carrier family 12 member 2
Prdx1	Peroxiredoxin-1
Glod4	Glyoxalase domain-containing protein 4
Mecp2	Methyl-CpG-binding protein 2
Astn1	Astrotactin-1
Bsg	Basigin
Mapk3	Mitogen-activated protein kinase 3
Vars	Valine--tRNA ligase
Acadl	Long-chain specific acyl-CoA dehydrogenase, mitochondrial
Ccdc177	Coiled-coil domain-containing protein 177
Chl1	Neural cell adhesion molecule L1-like protein
Gnpda2	Glucosamine-6-phosphate isomerase 2
Ndufa13	NADH dehydrogenase [ubiquinone] 1 alpha subcomplex subunit 13
Ajm1	Apical junction component 1 homolog
Selenbp1	Methanethiol oxidase
Slc6a9	Sodium- and chloride-dependent glycine transporter 1
Snrpd2	Small nuclear ribonucleoprotein Sm D2
Spart	Spartin
Pygl	Glycogen phosphorylase, liver form
Eef1b	Elongation factor 1-beta
Psme1	Proteasome activator complex subunit 1
Cyth2	Cytohesin-2
Slc23a2	Solute carrier family 23 member 2
Entpd2	Ectonucleoside triphosphate diphosphohydrolase 2
Ptgfrn	Prostaglandin F2 receptor negative regulator
Fen1	Flap endonuclease 1
Gemin5	Gem-associated protein 5
Mical1	[F-actin]-monooxygenase MICAL1
Brinp2	BMP/retinoic acid-inducible neural-specific protein 2
Tub	Tubby protein
Hba	Hemoglobin subunit alpha
Hbb-b1	Hemoglobin subunit beta-1
Hbb-b2	Hemoglobin subunit beta-2
Ca1	Carbonic anhydrase 1
Hrg	Histidine-rich glycoprotein
Fgg	Fibrinogen gamma chain

Ptx3	Pentraxin-related protein PTX3
Fga	Fibrinogen alpha chain
Nin	Ninein
Ubqln1	Ubiquilin-1
Slc4a1	Band 3 anion transport protein
Mapk1	Mitogen-activated protein kinase 1
Fn1	Fibronectin
Cfb	Complement factor B
Fbn1	Fibrillin-1
Serpinf2	Alpha-2-antiplasmin
Lrig1	Leucine-rich repeats and immunoglobulin-like domains protein 1
Ig kappa chain V-III region PC 2154	Ig kappa chain V-III region PC 2154
Gtf2a2	Transcription initiation factor IIA subunit 2
H2-D1	H-2 class I histocompatibility antigen, D-B alpha chain
Dguok	Deoxyguanosine kinase, mitochondrial
Cp	Ceruloplasmin
Nek7	Serine/threonine-protein kinase Nek7
Crp	C-reactive protein
Agt	Angiotensinogen
Itprid2	Protein ITPRID2
Spta1	Spectrin alpha chain, erythrocytic 1
Bpgm	Bisphosphoglycerate mutase
B2m	Beta-2-microglobulin
Cops7a	COP9 signalosome complex subunit 7a
Smarcd1	SWI/SNF-related matrix-associated actin-dependent regulator of chromatin subfamily D member 1
Sema3a	Semaphorin-3A
Mrpl49	39S ribosomal protein L49, mitochondrial
Gsdme	Gasdermin-E
Cnn2	Calponin-2
Mms19	MMS19 nucleotide excision repair protein homolog
Mrpl18	39S ribosomal protein L18, mitochondrial
Rasal1	RasGAP-activating-like protein 1
Egfr	Epidermal growth factor receptor
Prkra	Interferon-inducible double-stranded RNA-dependent protein kinase activator A
Dffa	DNA fragmentation factor subunit alpha
Fam169a	Soluble lamin-associated protein of 75 kDa
Clstn3	Calsyntenin-3
Rtf2	Replication termination factor 2
Map1a	Microtubule-associated protein 1A
Atp1a1	Sodium/potassium-transporting ATPase subunit alpha-1

Pgk1	Phosphoglycerate kinase 1
Bin1	Myc box-dependent-interacting protein 1
Scrn1	Secernin-1
Pfkl	ATP-dependent 6-phosphofructokinase, liver type
Kif5a	Kinesin heavy chain isoform 5A
Hip1r	Huntingtin-interacting protein 1-related protein
Pip5k1c	Phosphatidylinositol 4-phosphate 5-kinase type-1 gamma
Lrrc7	Leucine-rich repeat-containing protein 7
Dpp3	Dipeptidyl peptidase 3
Ank1	Ankyrin-1
Vdac3	Voltage-dependent anion-selective channel protein 3
Coro7	Coronin-7
Ctnnd1	Catenin delta-1
Clstn1	Calsyntenin-1
Me3	NADP-dependent malic enzyme, mitochondrial
Smpd3	Sphingomyelin phosphodiesterase 3
Psmb3	Proteasome subunit beta type-3
Camkk2	Calcium/calmodulin-dependent protein kinase kinase 2
Reps2	RalBP1-associated Eps domain-containing protein 2
Osbp2	Oxysterol-binding protein 2
Il1rap	Interleukin-1 receptor accessory protein
Cpne1	Copine-1
Eps8	Epidermal growth factor receptor kinase substrate 8
Smap1	Stromal membrane-associated protein 1
Fundc1	FUN14 domain-containing protein 1
F13a1	Coagulation factor XIII A chain
Lrrfip2	Leucine-rich repeat flightless-interacting protein 2
Acbd6	Acyl-CoA-binding domain-containing protein 6
Fermt3	Fermitin family homolog 3
Rprd1b	Regulation of nuclear pre-mRNA domain-containing protein 1B
Rbfox3	RNA binding protein fox-1 homolog 3
Carmil2	Capping protein, Arp2/3 and myosin-I linker protein 2
Impdh1	Inosine-5'-monophosphate dehydrogenase 1
Trappc1	Trafficking protein particle complex subunit 1
Cars2	Probable cysteine--tRNA ligase, mitochondrial
Plrg1	Pleiotropic regulator 1
Pde12	2',5'-phosphodiesterase 12
Arhgap27	Rho GTPase-activating protein 27
Rabl2	Rab-like protein 2A
Enpp1	Ectonucleotide pyrophosphatase/phosphodiesterase family member 1
Pigo	GPI ethanolamine phosphate transferase 3
Wdsub1	WD repeat, SAM and U-box domain-containing protein 1

Supplementary Table 4 | The function and subcellular localization of common and specific DEPs (FC>2, FDR<0.05) between aged ICH and young ICH mice compared with their corresponding controls.

Protein name	Gene name	Foldchange	Subcellular localization	Biological function
**Common DEPs**				
Apolipoprotein M	ApoM	3.697 (young)<br>2.453 (aged)	Extracellular	Lipid transport; lipoprotein metabolic process; negative regulation of plasma lipoprotein oxidation
Alpha-1-antitrypsin 1-5	Serpina1e	2.666 (young)<br>2.218 (aged)	Extracellular	Protease binding activity;serine-type endopeptidase inhibitor activity; response to cytokine
Protein S100-A8	S100a8	2.045 (young)<br>2.368 (aged)	Cytoplasmic; Nuclear	A calcium- and zinc-binding protein; positive regulation of inflammatory response; autophagy
Protein S100-A9	S100a9	2.132 (young)<br>2.217 (aged)	Nuclear	A calcium- and zinc-binding protein; positive regulation of inflammatory response; autophagy
Double-strand-break repair protein rad21 homolog	Rad21	4.364 (young)<br>3.073 (aged)	Nuclear	Double-strand-break repair protein rad21 homolog; apoptotic process; cell division
Serum albumin	Alb	3.295 (young)<br>3.374 (aged)	Extracellular	The regulation of the colloidal osmotic pressure of blood; negative regulation of apoptotic process
Vitronectin	Vtn	2.079 (young)<br>2.051 (aged)	Extracellular	A cell adhesion and spreading factor; oligodendrocyte differentiation; positive regulation of receptor-mediated endocytosis

Pregnancy zone protein	Pzp	2.108 (young) 2.163 (aged)	Plasma membrane	A proteinase inhibitor
**Aged specific DEPs**				
Afamin	Afm	2.067	Extracellular	Protein stabilization; protein transport within extracellular region; vitamin transport
Carbonic anhydrase 1	Ca1	2.14	Cytoplasmic	One-carbon metabolic process
Kininogen-1	Kng1	2.024	Extracellular	angiogenesis; inflammatory response; positive regulation of cytosolic calcium ion concentration
Histidine-rich glycoprotein	Hrg	2.033	Nuclear	angiogenesis; blood vessel remodeling; regulation of transcription from RNA polymerase II promoter in response to iron
Fibrinogen beta chain	Fgb	2.095	Nuclear	blood coagulation, fibrin clot formation; cellular response to interleukin-1;
Hemoglobin subunit alpha	Hba	5.455	Extracellular; Mitochondria l	oxygen transport; cellular oxidant detoxification
Hemoglobin subunit beta-1	Hbb-b1	4.29	Extracellular; Cytoplasmic	oxygen transport; cellular oxidant detoxification
Hemoglobin subunit beta-2	Hbb-b2	2.478	Extracellular; Cytoplasmic	oxygen transport; cellular oxidant detoxification
**Young specific DEPs**				
Murinoglobulin-1	Mug1	2.045	Plasma membrane; Nuclear	A proteinase activates the inhibitor
Glycophorin-A	Gypa	3.732	Extracellular; Plasma Membrane; Nuclear	Regulation of response to osmotic stress

Leucine-rich repeat-containing protein 36	Lrrc36	2.781	Nuclear	-
Neuropathy target esterase	Pnpla6	2.057	Nuclear	angiogenesis; lipid catabolic process; phosphatidylcholine metabolic proces
Apolipoprotein A-I	Apoa1	2.051	Extracellular	lipoprotein metabolic process; Reverse cholesterol transport; vitamin transport

Supplementary Table 5 | Common and specific DEPs in PPI networks.

Module	Molecules	Nodes	Score	Edges
	**Common DEPs**			
1	Fgb, Klkb1, Pzp, ApoM, Plg, Ahsg, Ces1c, Serpina1b, Kng1, F2, Serpina1a, Pon1, Mbl2, Alb, Afm, Cfi, Itih1, Apoh, Ttr, Gc, Serping1, C3, Serpinc1, Vtn, Apoa4, Mug1	26	21.44	268
	**Aged specific DEPs**			
1	Fn1, Crp, Serpinf2, Fga, Fgg, Cp, Agt, Mapk1, F13a1, Cfb, Hrg	11	7.4	37
2	Bpgm, Pgk1, Pfkl, Me3	4	3.33	5
3	Spta1, Slc4a1, Ank1	3	3	3
	**Young specific DEPs**			
1	Apoa1, Apoa2, C4b, C9, Clu, Hp, Hpx, Orm2	8	7.143	25
2	Rpl21, Rpl7a, Rps13	3	3	3
	**Aged sham vs. Young sham**			
1	Cldn11, Plp1, Mog, Hapln2	4	3.333	5
2	Nmral1, Ndufa13, Ndufb4	3	3	3

Supplementary Table 6 | IPA pathway enrichment results for the up- or down- regulated DEPs following ICH.

Ingenuity Canonical Pathways	-log(P-value)	Molecules
		**Common upregulated DEPs**

LXR/RXR Activation	18.90	AHSG, ALB, APOA4, APOH, APOM, C3, GC, KNG1, PON1, S100A8, SERPINA1, TTR, VTN
FXR/RXR Activation	16.90	AHSG, ALB, APOA4, APOH, APOM, C3, GC, KNG1, PON1, SERPINA1, TTR, VTN
Acute Phase Response Signaling	16.60	AHSG, ALB, APOH, C3, F2, FGB, KLKB1, MBL2, PLG, SERPINA1, SERPINA3, SERPING1, TTR
Coagulation System	12.30	F2, FGB, KLKB1 ,KNG1, PLG, SERPINA1, SERPINC1
Complement System	8.00	C3, CFH ,CFI, MBL2, SERPING1
Intrinsic Prothrombin Activation Pathway	7.71	F2, FGB, KLKB1, KNG1, SERPINC1
Clathrin-mediated Endocytosis Signaling	7.00	ALB, APOA4, APOM, F2, PON1, S100A8, SERPINA1
Atherosclerosis Signaling	6.65	ALB, APOA4, APOM, PON1, S100A8, SERPINA1

IL-12 Signaling and Production in Macrophages	6.56	ALB, APOA4, APOM, PON1, S100A8, SERPINA1
Production of Nitric Oxide and Reactive Oxygen Species in Macrophages	5.69	ALB, APOA4, APOM, PON1, S100A8, SERPINA1
Extrinsic Prothrombin Activation Pathway	5.40	F2, FGB, SERPINC1
Role of IL-17A in Psoriasis	3.47	S100A8, S100A9
Maturity Onset Diabetes of Young (MODY) Signaling	3.31	APOA4, APOH, APOM
Neuroprotective Role of THOP1 in Alzheimer's Disease	2.76	KNG1, PLG, SERPINA3
Heme Degradation	2.10	BLVRB
Glioma Invasiveness Signaling	2.04	PLG, VTN
Osteoarthritis Pathway	1.95	MYBBP1A, S100A8, S100A9
Role of Tissue Factor in Cancer	1.66	F2, FGB
Role of Pattern Recognition Receptors in Recognition of Bacteria and Viruses	1.42	C3, MBL2
	**Aged specific upregulated DEPs**	
Acute Phase Response Signaling	10.40	AGT, CFB, CP, CRP, FGA, FGG, FN1, IL1RAP, MAPK1, SERPINF2

**Iron homeostasis signaling pathway**	5.82	CP, EGFR, Hbb-b1, Hbb-b2, MAPK1, MMS19
Coagulation System	5.71	F13A1, FGA, FGG, SERPINF2
Extrinsic Prothrombin Activation Pathway	5.06	F13A1, FGA, FGG
Glucocorticoid Receptor Signaling	3.94	AGT, B2M, EGFR, FGG, GTF2A2,<br>HLA-A, MAPK1, SMARCD1
Intrinsic Prothrombin Activation Pathway	3.77	F13A1, FGA, FGG
Role of Tissue Factor in Cancer	3.65	EGFR, FGA, FGG, MAPK1
LXR/RXR Activation	3.55	AGT, FGA, IL1RAP, SERPINF2
Caveolar-mediated Endocytosis Signaling	3.02	B2M, EGFR, HLA-A
Hepatic Fibrosis / Hepatic Stellate Cell Activation	2.81	AGT, EGFR, FN1, IL1RAP
Pyroptosis Signaling Pathway	2.75	GSDME, MAPK1, NEK7
IL-17A Signaling in Gastric Cells	2.69	EGFR, MAPK1
Wound Healing Signaling Pathway	2.40	EGFR, FN1, IL1RAP, MAPK1
FXR/RXR Activation	2.38	AGT, FGA, SERPINF2
IL-6 Signaling	2.36	CRP, IL1RAP, MAPK1

tRNA Charging	2.35	CARS2, VARS1
Antigen Presentation Pathway	2.35	B2M, HLA-A
p70S6K Signaling	2.32	AGT, EGFR, MAPK1
UVC-Induced MAPK Signaling	2.12	EGFR, MAPK1
UVB-Induced MAPK Signaling	2.10	EGFR, MAPK1
EGF Signaling	2.06	EGFR, MAPK1
Neuroinflammation Signaling Pathway	2.05	B2M, CRP, HLA-A, MAPK1
Erythropoietin Signaling Pathway	1.97	Hbb-b1, Hbb-b2, MAPK1
Tumor Microenvironment Pathway	1.96	FN1, HLA-A, MAPK1
Semaphorin Signaling in Neurons	1.95	MAPK1, SEMA3A
SPINK1 General Cancer Pathway	1.87	EGFR, MAPK1
Agrin Interactions at Neuromuscular Junction	1.85	EGFR, MAPK1
Natural Killer Cell Signaling	1.84	B2M, HLA-A, MAPK1
ID1 Signaling Pathway	1.83	EGFR, FN1, MAPK1
IL-10 Signaling	1.83	IL1RAP, MAPK1
Rapoport-Luebering Glycolytic Shunt	1.82	BPGM
Estrogen-Dependent Breast Cancer Signaling	1.74	EGFR, MAPK1
BAG2 Signaling Pathway	1.71	MAPK1, PSMB3

Regulation of Cellular Mechanics by Calpain Protease	1.66	EGFR, MAPK1
Non-Small Cell Lung Cancer Signaling	1.61	EGFR, MAPK1
ERBB Signaling	1.61	EGFR, MAPK1
IL-1 Signaling	1.60	IL1RAP, MAPK1
UVA-Induced MAPK Signaling	1.58	EGFR, MAPK1
Virus Entry via Endocytic Pathways	1.53	B2M, HLA-A
Apoptosis Signaling	1.53	DFFA, MAPK1
PD-1, PD-L1 cancer immunotherapy pathway	1.52	B2M, HLA-A
Telomerase Signaling	1.51	EGFR, MAPK1
PPAR Signaling	1.51	IL1RAP, MAPK1
Protein Ubiquitination Pathway	1.47	B2M, HLA-A, PSMB3
Bladder Cancer Signaling	1.45	EGFR, MAPK1
**Huntington's Disease Signaling**	1.44	EGFR, MAPK1, PSMB3
Neuregulin Signaling	1.44	EGFR, MAPK1
Cholecystokinin/Gastrin-mediated Signaling	1.43	EGFR, MAPK1
**Neuroprotective Role of THOP1 in Alzheimer's Disease**	1.42	AGT, HLA-A
Renin-Angiotensin Signaling	1.41	AGT, MAPK1

**Parkinson's Signaling**	1.39	MAPK1
Glioma Signaling	1.39	EGFR, MAPK1
Pancreatic Adenocarcinoma Signaling	1.38	EGFR, MAPK1
GP6 Signaling Pathway	1.37	FGA, FGG
G Beta Gamma Signaling	1.36	EGFR, MAPK1
		**Young specific upregulated DEPs**
Acute Phase Response Signaling	9.2400	APOA1, APOA2, C4A/C4B, C9, HP, HPX, ITIH4, STAT3
LXR/RXR Activation	8.9500	APOA1, APOA2, C4A/C4B, C9, CLU, HPX, ITIH4
FXR/RXR Activation	8.8800	APOA1, APOA2, C4A/C4B, C9, CLU, HPX, ITIH4
Complement System	4.5200	C4A/C4B, C8B, C9
Atherosclerosis Signaling	2.8900	APOA1, APOA2, CLU
IL-12 Signaling and Production in Macrophages	2.8400	APOA1, APOA2, CLU
**Iron homeostasis signaling pathway**	2.8200	HP,HPX, STAT3
Glycine Degradation (Creatine Biosynthesis)	2.4800	GATM
Production of Nitric Oxide and Reactive Oxygen Species in Macrophages	2.4200	APOA1, APOA2, CLU

Clathrin-mediated Endocytosis Signaling	2.4100	APOA1, APOA2, CLU
EIF2 Signaling	2.2300	RPL21, RPL7A, RPS13
Maturity Onset Diabetes of Young (MODY) Signaling	2.1400	APOA1, APOA2
GDP-mannose Biosynthesis	2.0100	PMM1
Salvage Pathways of Pyrimidine Deoxyribonucleotides	1.8300	UPP1
Pancreatic Adenocarcinoma Signaling	1.7400	GPLD1, STAT3
Gαi Signaling	1.6600	GRM7, STAT3
Colanic Acid Building Blocks Biosynthesis	1.6400	PMM1
Choline Biosynthesis III	1.6100	GPLD1
IL-22 Signaling	1.4100	STAT3
Role of JAK family kinases in IL-6-type Cytokine Signaling	1.4000	STAT3
PPARα/RXRα Activation	1.3900	APOA1, APOA2
Role of JAK1, JAK2 and TYK2 in Interferon Signaling	1.3800	STAT3
Coronavirus Pathogenesis Pathway	1.3600	RPS13, STAT3
**Aged specific downregulated DEPs**		
Gluconeogenesis I	3.5100	ME3, PGK1
Glycolysis I	3.4700	PFKL, PGK1

Sphingomyelin Metabolism	2.0900	SMPD3
Endocannabinoid Cancer Inhibition Pathway	2.0200	CAMKK2, SMPD3
Purine Nucleotides De Novo Biosynthesis II	1.9600	IMPDH1
Urate Biosynthesis/Inosine 5'-phosphate Degradation	1.8500	IMPDH1
3-phosphoinositide Biosynthesis	1.7400	ATP1A1, PIP5K1C
Purine Nucleotides Degradation II (Aerobic)	1.7200	IMPDH1
**Autophagy**	1.7100	CAMKK2, FUNDC1
Superpathway of Inositol Phosphate Compounds	1.6400	ATP1A1, PIP5K1C
AMPK Signaling	1.6000	CAMKK2, PFKL
**D-myo-inositol (1,4,5)-Trisphosphate Biosynthesis**	1.5900	PIP5K1C
Oxytocin In Spinal Neurons Signaling Pathway	1.4600	CAMKK2
Sirtuin Signaling Pathway	1.4600	PGK1, VDAC3
**Young specific downregulated DEPs**		
Synaptic Long Term Potentiation	2.72	CHP1, PLCB1
fMLP Signaling in Neutrophils	2.72	CHP1, PLCB1

PI3K Signaling in B Lymphocytes	2.64	CHP1, PLCB1
Endocannabinoid Neuronal Synapse Pathway	2.61	CHP1, PLCB1
Glycerol Degradation I	2.52	Gk
Gαq Signaling	2.49	CHP1, PLCB1
Dopamine-DARPP32 Feedback in cAMP Signaling	2.43	CHP1, PLCB1
**Autophagy**	2.30	CHP1, WIPI2
Role of NFAT in Cardiac Hypertrophy	2.27	CHP1, PLCB1
Cardiac Hypertrophy Signaling	2.14	CHP1, PLCB1
Oxytocin Signaling Pathway	2.07	CHP1, PLCB1
Role of Macrophages, Fibroblasts and Endothelial Cells in Rheumatoid Arthritis	1.94	CHP1, PLCB1
**D-myo-inositol (1,4,5)-Trisphosphate Biosynthesis**	1.89	PLCB1
Protein Kinase A Signaling	1.76	CHP1, PLCB1
G Protein Signaling Mediated by Tubby	1.66	PLCB1
nNOS Signaling in Neurons	1.63	CHP1
Axonal Guidance Signaling	1.58	CHP1, PLCB1

Cardiac Hypertrophy Signaling (Enhanced)	1.53	CHP1, PLCB1
WNT/Ca+ pathway	1.48	PLCB1
Phospholipases	1.48	PLCB1
Role of NFAT in Regulation of the Immune Response	1.46	CHP1, PLCB1
Melatonin Signaling	1.45	PLCB1
Netrin Signaling	1.45	CHP1
GPCR-Mediated Integration of Enteroendocrine Signaling Exemplified by an L Cell	1.43	PLCB1
Leptin Signaling in Obesity	1.42	PLCB1
IL-3 Signaling	1.41	CHP1
Chemokine Signaling	1.40	PLCB1
Phospholipase C Signaling	1.35	CHP1, PLCB1
RANK Signaling in Osteoclasts	1.35	CHP1

Supplementary Table 7 | GO and KEGG enrichment results for upregulated DEPs in the aged sham group versus the young sham group.

Ontology	ID	Description	Gene Ratio	BgRatio	P-value	P.adjust	qvalue
BP	GO:0006575	cellular modified amino acid metabolic process	4/35	165/23328	0.000106488	0.094363062	0.069831676
BP	GO:1902513	regulation of organelle transport along microtubule	2/35	12/23328	0.000142974	0.094363062	0.069831676

BP	GO:0048708	astrocyte differentiation	3/35	86/23328	0.00029072	0.12791663	0.094662386
BP	GO:0009636	response to toxic substance	3/35	116/23328	0.000698188	0.143316824	0.106059022
BP	GO:0010038	response to metal ion	4/35	274/23328	0.00073194	0.143316824	0.106059022
CC	GO:0043209	myelin sheath	7/38	213/23271	4.83695E-08	7.01357E-06	4.27688E-06
CC	GO:0016328	lateral plasma membrane	4/38	66/23271	4.05033E-06	0.000293649	0.000179067
CC	GO:0045178	basal part of cell	4/38	83/23271	1.0124E-05	0.000489327	0.000298392
CC	GO:0009925	basal plasma membrane	3/38	60/23271	0.000128904	0.00410301	0.002502017
CC	GO:0005883	neurofilament	2/38	11/23271	0.000141483	0.00410301	0.002502017
MF	GO:0015643	toxic substance binding	2/35	14/22669	0.000208298	0.024003644	0.015463953
MF	GO:0031406	carboxylic acid binding	4/35	226/22669	0.000395179	0.024003644	0.015463953
MF	GO:0043177	organic acid binding	4/35	241/22669	0.000503546	0.024003644	0.015463953
MF	GO:0005540	hyaluronic acid binding	2/35	22/22669	0.00052467	0.024003644	0.015463953
MF	GO:0015370	solute:sodium symporter activity	2/35	73/22669	0.0056817	0.119558587	0.077023651
KEGG	mmu00250	Alanine, aspartate and glutamate metabolism	2/26	39/9040	0.005521319	0.446654962	0.437839404
KEGG	mmu04723	Retrograde endocannabinoid signaling	3/26	148/9040	0.008484064	0.446654962	0.437839404
KEGG	mmu05160	Hepatitis C	3/26	165/9040	0.011407263	0.446654962	0.437839404
KEGG	mmu04662	B cell receptor signaling pathway	2/26	83/9040	0.023469948	0.446654962	0.437839404
KEGG	mmu05208	Chemical carcinogenesis - reactive oxygen species	3/26	222/9040	0.025059446	0.446654962	0.437839404

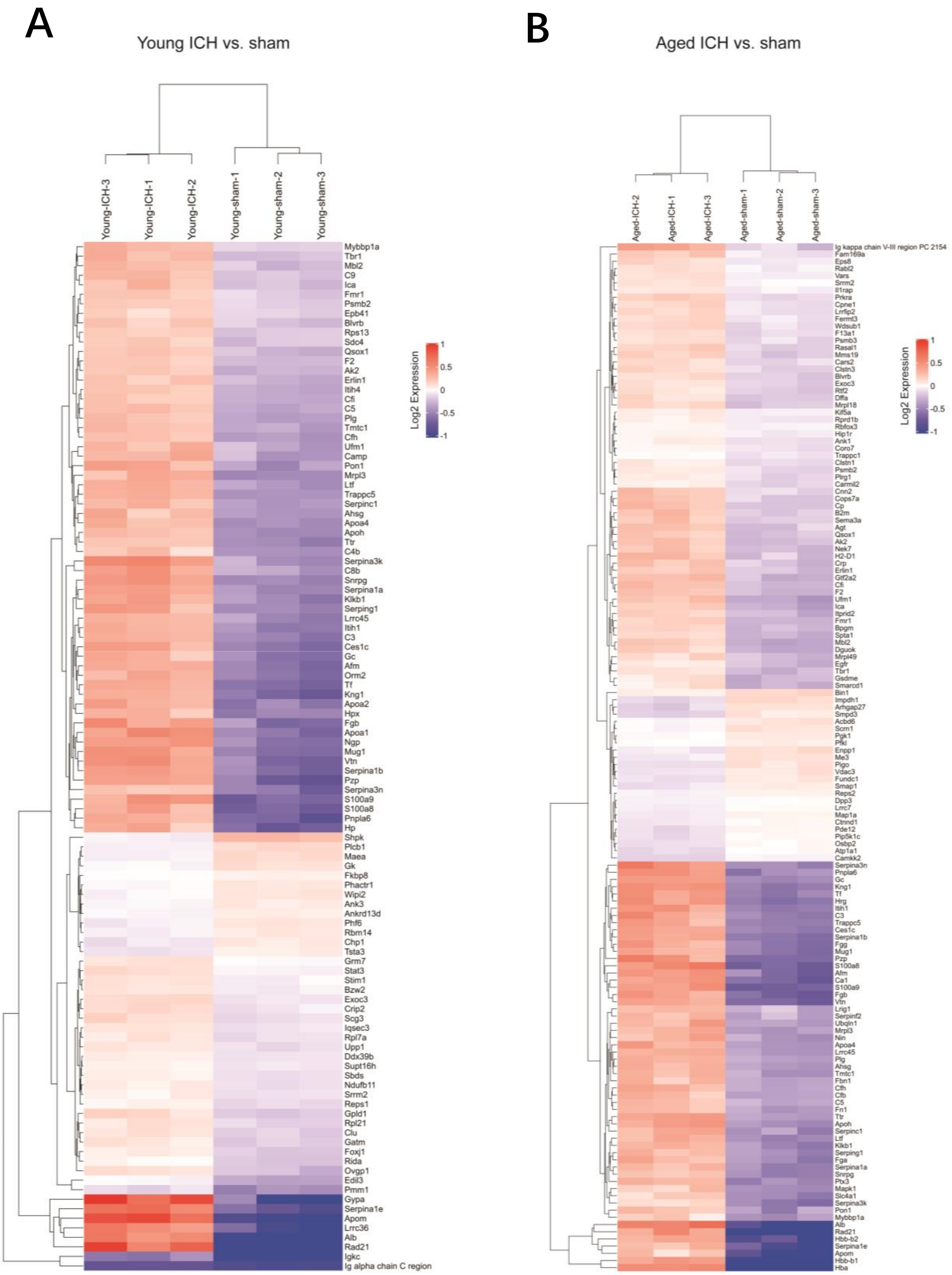

Supplementary Figure 1 | Hierarchical clustering heatmaps of DEPs between the ICH and sham controls in young and aged mice. (A) Young ICH vs. Young sham. (B) Aged ICH vs. Aged sham.

A

Young ICH vs. sham

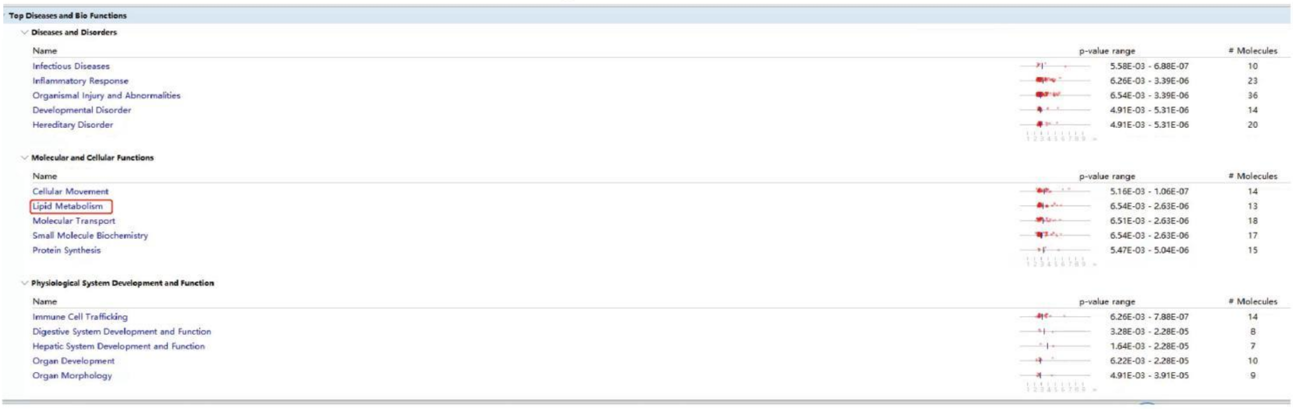

Top Diseases and Bio Functions

Diseases and Disorders

Name	p-value range	# Molecules
Infectious Diseases	5.58E-03 - 6.88E-07	10
Inflammatory Response	6.26E-03 - 3.39E-06	23
Organismal Injury and Abnormalities	6.54E-03 - 3.39E-06	36
Developmental Disorder	4.91E-03 - 5.31E-06	14
Hereditary Disorder	4.91E-03 - 5.31E-06	20

Molecular and Cellular Functions

Name	p-value range	# Molecules
Cellular Movement	5.16E-03 - 1.06E-07	14
Lipid Metabolism	6.54E-03 - 2.63E-06	13
Molecular Transport	6.51E-03 - 2.63E-06	18
Small Molecule Biochemistry	6.54E-03 - 2.63E-06	17
Protein Synthesis	5.47E-03 - 5.04E-06	15

Physiological System Development and Function

Name	p-value range	# Molecules
Immune Cell Trafficking	6.26E-03 - 7.88E-07	14
Digestive System Development and Function	3.28E-03 - 2.28E-05	8
Hepatic System Development and Function	1.64E-03 - 2.28E-05	7
Organ Development	6.22E-03 - 2.28E-05	10
Organ Morphology	4.91E-03 - 3.91E-05	9

B

Aged ICH vs. sham

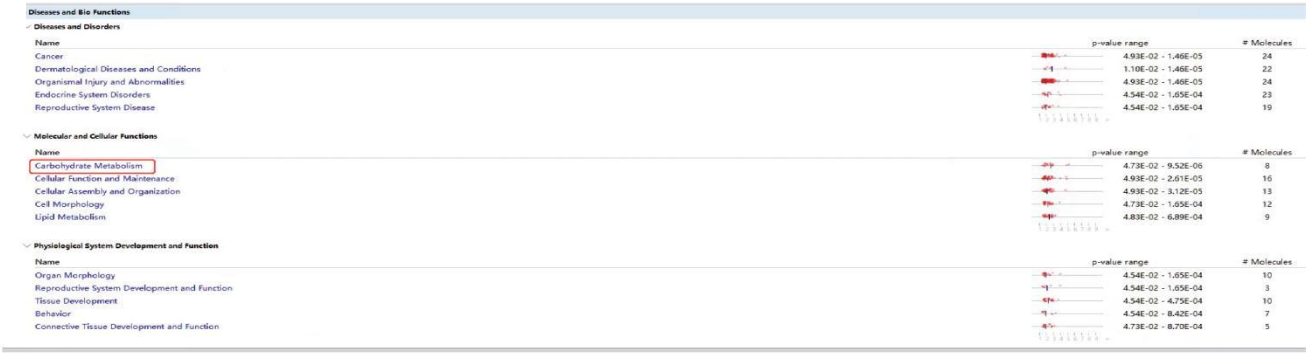

Diseases and Bio Functions

Diseases and Disorders

Name	p-value range	# Molecules
Cancer	4.93E-02 - 1.46E-05	24
Dermatological Diseases and Conditions	1.10E-02 - 1.46E-05	22
Organismal Injury and Abnormalities	4.93E-02 - 1.46E-05	24
Endocrine System Disorders	4.54E-02 - 1.65E-04	23
Reproductive System Disease	4.54E-02 - 1.65E-04	19

Molecular and Cellular Functions

Name	p-value range	# Molecules
Carbohydrate Metabolism	4.73E-02 - 9.52E-06	8
Cellular Function and Maintenance	4.93E-02 - 2.61E-05	16
Cellular Assembly and Organization	4.93E-02 - 3.12E-05	13
Cell Morphology	4.73E-02 - 1.65E-04	12
Lipid Metabolism	4.83E-02 - 6.89E-04	9

Physiological System Development and Function

Name	p-value range	# Molecules
Organ Morphology	4.54E-02 - 1.65E-04	10
Reproductive System Development and Function	4.54E-02 - 1.65E-04	3
Tissue Development	4.54E-02 - 4.75E-04	10
Behavior	4.54E-02 - 8.42E-04	7
Connective Tissue Development and Function	4.73E-02 - 8.70E-04	5

Supplementary Figure 2 | Top diseases and biofunctional enrichment results with the IPA software. (A) Young ICH vs. Young sham. (B) Aged ICH vs. Aged sham.

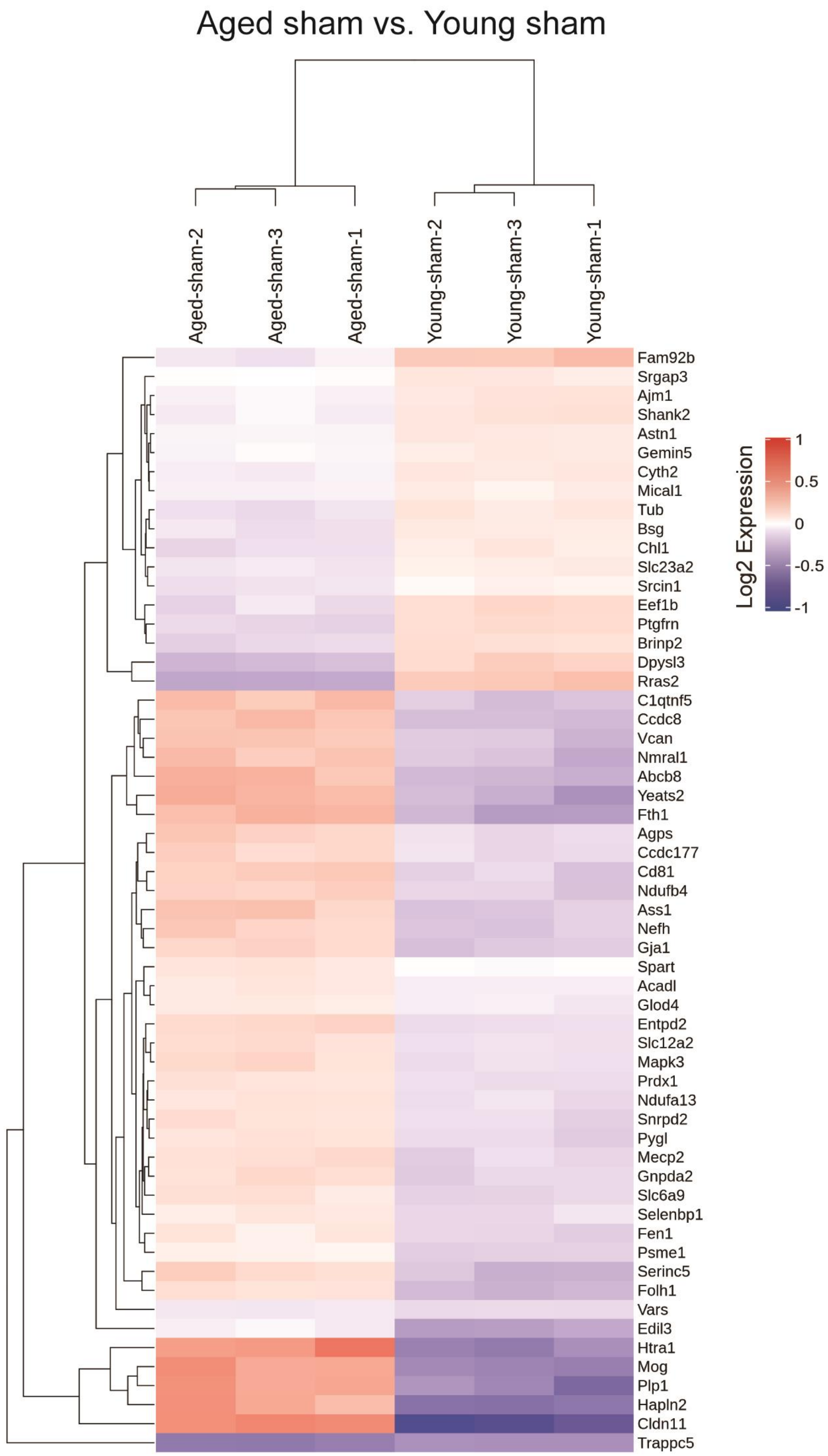

Supplementary Figure 3 | The hierarchical clustering heatmap of DEPs in the aged sham group versus the young sham group.
